# Ni-Catalyzed Enantioselective Intramolecular Mizoroki–Heck Reaction for the Synthesis of Phenanthridinone Derivatives

**DOI:** 10.1021/acs.joc.3c00202

**Published:** 2023-06-15

**Authors:** Diana Rachii, Dana J. Caldwell, Yui Kosukegawa, Mary Sexton, Paul R. Rablen, William P. Malachowski

**Affiliations:** † Chemistry Department, Bryn Mawr College, Bryn Mawr, Pennsylvania 19010, United States; ‡ Chemistry Department, Swarthmore College, Swarthmore, Pennsylvania 19081, United States

## Abstract

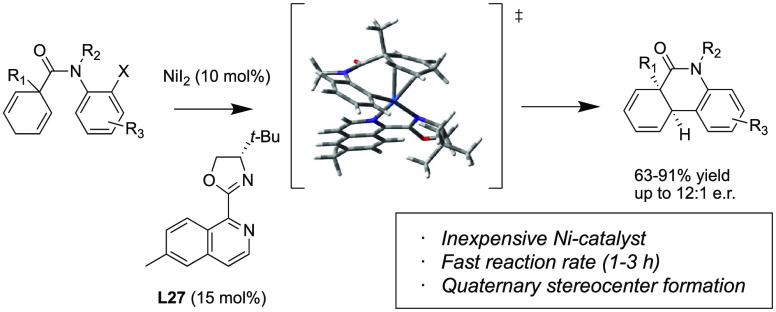

A Ni-catalyzed enantioselective intramolecular Mizoroki–Heck reaction has been developed to transform symmetrical 1,4-cyclohexadienes with attached aryl halides into phenanthridinone analogues containing quaternary stereocenters. Herein, we report important advances in reaction optimization enabling control of unwanted proto-dehalogenation and alkene reduction side products. Moreover, this approach provides direct access to six-membered ring heterocyclic systems bearing all-carbon quaternary stereocenters, which have been much more challenging to form enantioselectively with nickel-catalyzed Heck reactions. A wide range of substrates were demonstrated to work in good to excellent yields. Good enantioselectivity was demonstrated using a new synthesized chiral *i*Quinox-type bidentate ligand (**L27**). The sustainability, low price of nickel catalysts, and significantly faster reaction rate (1 h) versus that of a recently reported palladium-catalyzed reaction (20 h) make this process an attractive alternative.

## Introduction

Palladium has a privileged status in the world of cross-coupling chemistry, having demonstrated a unique versatility and efficiency in a range of synthetic organometallic transformations.^1−4^ Nevertheless, palladium’s cheaper, more earth abundant, and environmentally friendly group 10 relative nickel has recently attracted significant attention as a worthy replacement or, more frequently, as a complementary transition metal catalyst with unique reactivity.^5−9^ Nickel’s reactivity, especially its greater range of oxidation states,^9,10^ creates significant challenges in developing efficient and useful transformations. Despite this, and following the seminal nickel reports of Watson and Jacobsen^11^ and Nakao,^12^ there have been a flood of recent reports^5−7^ with applications of nickel-catalyzed alkene functionalization and other Heck-type reactions illustrating an impressive array of reactivity. In the specific and most challenging realm of intramolecular enantioselective reactions to form quaternary stereocenters, there has also been a variety of impressive reports.^13^ However, most of these communications report new alkene reaction manifolds such as dicarbofunctionalization;^14−16^ therefore, although there was early work demonstrating Ni-catalyzed Heck reactions,^17,18^ more recently there have been many fewer reports of traditional intramolecular^19,20^ or intermolecular^21−24^ Heck reaction transformations likely due to the difficulty in achieving selective reactions with nickel, the difficulty of nickel complexes undergoing β-hydride elimination, and the challenges in regenerating Ni(0) from Ni(II) in the Mizoroki–Heck reaction catalytic cycle.

The more popular Ni-catalyzed Heck-type difunctionalization reactions provide a complementary pathway to traditional palladium-catalyzed Heck reactions and overcome the much slower tendency of nickel complexes to undergo β-hydride elimination. Perhaps predictably then, many fewer examples have offered a direct replacement of nickel for palladium in a traditional enantioselective intramolecular Heck reaction. A second perhaps more surprising deficiency in the recent reports of intramolecular nickel-catalyzed Heck reactions, including alkene difunctionalization, is an example of six-membered ring formation with high levels of enantioselectivity. There is an abundance of reports with excellent enantioselectivity to form five-membered rings,^25−51^ with indolinone or oxindole structures being the most common product formed by far. In contrast, there is only one recent report with a collection of six-membered substrates with excellent enantioselectivity^52^ and two additional reports citing just one successful enantioselective six-membered ring example.^53,54^ Otherwise, communications to date have reported modest enantioselectivities below 50% enantiomeric excess (e.r. = 3:1) or no enantioselectivity achieved for six-membered ring examples.^26,29,55,56^ Given the importance of six-membered rings in organic chemistry and drug development,^57,58^ the scarcity of examples of enantioselective six-membered ring formation seems a serious omission. Herein, we demonstrate conditions for a new nickel-catalyzed enantioselective intramolecular Heck reaction that substitutes nickel for palladium in an identical Heck transformation that was recently reported by our group.^59^ In addition to contrasting the nickel-catalyzed conditions for the identical palladium Heck reaction, it also illustrates a rare example of an enantioselective nickel-catalyzed Heck reaction to form a wide selection of six-membered ring structures. The structures formed are quaternary stereocenter-containing analogues of the highly bioactive phenanthridinone structure and therefore should have interest to the drug development community due to the benefits of three-dimensional structures in successful drug architectures.^60−62^ As with our first report, the process uses a desymmetrization strategy^63,64^ to generate a chiral quaternary stereocenter. Importantly, the nickel Heck reaction occurs considerably faster than the palladium-catalyzed reaction, illustrating another important benefit of the nickel process consistent with other recent reports highlighting lower catalyst loadings and lower temperatures in a Suzuki–Miyaura reaction.^65^

## Results and Discussion

The substrates **1** for the Ni-catalyzed Heck reactions were synthesized using the previously reported^59^ three-step sequence that involves a Birch reduction–alkylation process followed by coupling with a corresponding primary or secondary 2-haloaniline derivative (SI-Table 1). As with the Pd-catalyzed case, tertiary amides must be used and *N*-methyl and *N*-methoxymethyl (N-MOM) amides were chosen for this work. The N-MOM amides allow subsequent deprotection to reveal the important H-bond donor in the secondary amide of the parent phenanthridinone (SI-Table 2).

In our previous work with Pd-catalyzed enantioselective desymmetrizing Heck reaction, the more sterically demanding alkyl groups at the quaternary center (e.g., -*i*Pr, -*i*Bu, and -Cy) resulted in lower enantioselectivities. Thus, we initially explored Ni as an alternative catalyst choice to achieve higher levels of enantioselectivity with these more sterically demanding substrates. Our preliminary screening of reaction parameters with tertiary aryl bromide **1d** in dimethylformamide (DMF) at 80 °C provided promising results using NiCl_2_ as the catalyst, bipyridine (bipy) as the ligand, and Zn as the reducing agent (Table 1, entry 1). The reaction was completed in an impressively short time (10 min) versus the palladium-catalyzed reaction (24 h), but with significant formation of two major side products: a protodehalogenated **2**-**1** species and a cyclized alkene **2**-**2** species. Both, we suspected, were the result of Ni–H intermediates. Further optimization using aryl bromide **1d** as the model substrate was performed to increase product yield and decrease the amount of the major side products.

**Table 1:**
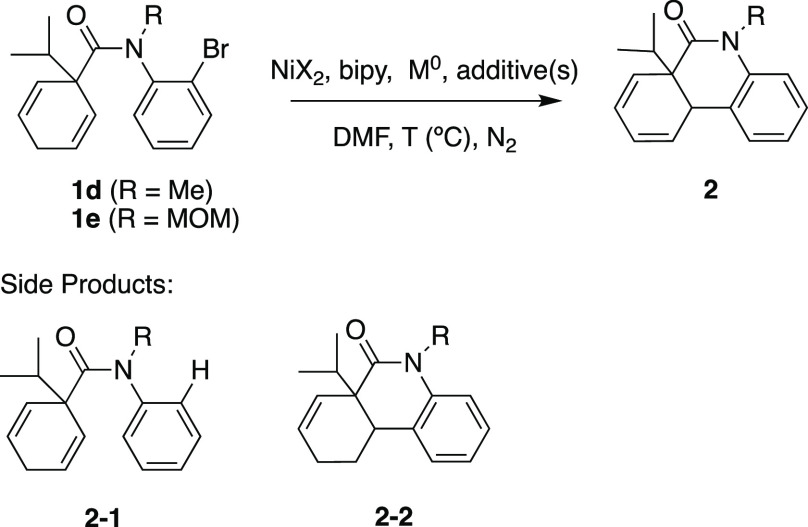
Optimization of the Heck Reaction^a^

entry	R	time	*T* (°C)	NiX_2_ (mol %)	M^0^ (equiv)	additive(s) (equiv)	**2** (%)^b^	**2-1** (%)^b^	**2-2** (%)^b^
1	Me	10 min	80	NiCl_2_ (20)	Zn (3.0)	–	45	49	6
2	Me	10 min	80	NiCl_2_ (20)	Zn (3.0)	LiI (1.0)	56	40	5
3	Me	10 min	80	NiCl_2_ (20)	Zn (3.0)	KI (1.0)	67	33	5
4	Me	1.5 h	80	NiCl_2_ (20)	Zn (3.0)	K_3_PO_4_ (1.0)	59	33	8
5	Me	1 h	80	NiCl_2_ (20)	Zn (1.5)	KI (1.0)	63	29	9
6	Me	10 min	120	NiCl_2_ (20)	Zn (3.0)	KI (1.0)	62	28	11
7	Me	2 h	60	NiCl_2_ (20)	Zn (3.0)	KI (1.0)	57	36	7
8	Me	20 min	80	NiCl_2_ (20)	Zn (3.0)	KI (1.0), 2-cyclohexenone (2.0)	85	9	6
9	Me	18 h	80	NiCl_2_ (20)	TDAE (3.0)	KI (1.0), 2-cyclohexenone (2.0)	80	4	10
10	MOM	1.5 h	80	NiCl_2_ (20)	Zn (3.0)	KI (1.0), 2-cyclohexenone (2.0)	72	0.2	22
11	MOM	3 h	80	NiCl_2_ (20)	Mn (3.0)	KI (1.0), 2-cyclohexenone (2.0)	73	0.3	25
12	MOM	24 h	80	Ni(acac)_2_ (20)	Mn (3.0)	KI (1.0), 2-cyclohexenone (3.0)	79	7	14
13	MOM	1 h	80	NiBr_2_ (20)	Mn (3.0)	KI (1.0), 2-cyclohexenone (3.0)	73	–	27
14	MOM	1.5 h	80	NiI_2_ (10)	Mn (3.0)	KI (1.0), 2-cyclohexenone (3.0)	93	–	7

a Conditions: NiX_2_ (10–20 mol %), bipy (15–20 mol %), M^0^ (3 equiv), additives (1–3 equiv), DMF (12 mL/mmol of ArX).

b Determined by GC analysis.

We first improved the catalytic system outcome with the addition of 1 equiv of LiI (entry 2). Iodide has been shown to improve the reactivity of Ni-catalyzed transformations, although the exact reason for the benefit is not definitively known. It has been proposed that the iodide either facilitates the transfer of electrons from the reducing agent (Zn) to the Ni catalytic complex,^66−68^ promotes formation of a beneficial nickelate complex,^69,70^ or facilitates β-hydride elimination.^71,72^ Alternatively, a nickel-catalyzed halogen exchange^73^ of the aryl bromide for iodide with the excess iodide can facilitate substrate reactivity toward oxidative addition of the nickel catalyst. Replacing LiI with KI as an external iodide source resulted in a slight decrease in the amount of unwanted protodebromination product **2**-**1** (entry 3). To test whether the KI improvement was more the result of the potassium cation than the iodide anion, we used K_3_PO_4_ as the additive (entry 4). The absence of iodide significantly prolonged the reaction time and increased the amount of cyclized alkene (**2**-**2**) versus the desired cyclized diene. Reducing the amount of Zn led to a decreased catalyst turnover rate (entry 5). Increasing or decreasing the temperature had a minimal impact on product yield (entry 3 vs entries 6 and 7). Because both side products, **2**-**1** and **2**-**2**, are likely the result of Ni–H intermediates, we explored the use of acceptor olefin additives to consume the hydride species. Prior work by Lv^74^ elegantly demonstrated that the addition of a sacrificial acceptor olefin facilitated the Ni(0)-catalyzed oxidative Heck arylation via a transfer hydrogenation process. To our delight, the reaction efficiency was significantly improved after the addition of 2 equiv of 2-cyclohexen-1-one as a sacrificial alkene (entry 8). Replacing Zn with tetrakis(dimethylamino)ethylene (TDAE) as an organic reductant resulted in a significantly longer reaction time (entry 9). Unfortunately, after employing our initially optimized conditions to a MOM-protected tertiary amide **1e** (entry 10), we observed an increased level of formation of cyclized alkene side product **2**-**2**, so further optimization through additional screening of the Ni source, reductants, and additives was performed (entries 11–14). The reaction efficiency was dramatically improved with NiI_2_ (10 mol %) as the catalyst and Mn as the reductant (entry 14). Because Mn is a stronger two-electron reductant [*E*° = −1.18 V vs the standard hydrogen electrode (SHE) in water] than Zn (*E*° = −0.76 V vs SHE in water), it likely accelerates catalyst turnover by reducing the Ni(II)-H species faster, if the Ni(II)-H is not consumed by the sacrificial alkene.

**Scheme 1:**
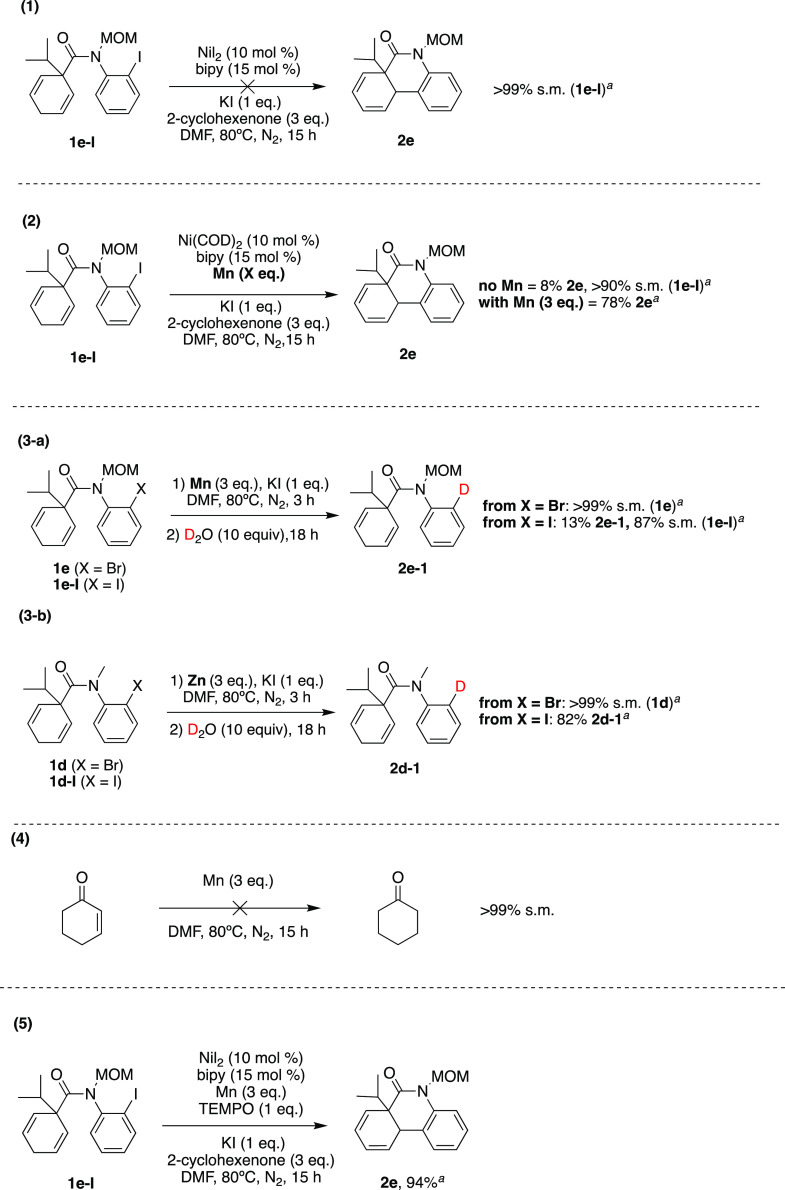
Mechanistic Control Studies Reactions were run on a 20–25 mg scale of aryl halide **1** or **1-I** and analyzed by GC-MS. s.m. = starting material.

Initially, we explored an enantioselective version of this reaction with a survey of common chiral ligands (**L1–18**) used in Ni-catalyzed cross-coupling reactions (SI-Table 3). Unfortunately, an array of commercially available bidentate and tridentate N- or P,N-ligands commonly reported with asymmetric Ni-catalyzed reactions^75−78^ (e.g., pyox, box, pybox, biox, diamine, PHOX, Quinap, and PINAP) did not provide optimal enantioselective results. However, we observed that *t*Bu-pyox- and *t*Bu-*i*Quinox-type ligands were more enantioselective, and therefore, subsequent studies focused on these ligand types. Thus, we began to synthesize and test novel functionalized *t*Bu-pyox^79,80^ and *t*Bu*-i*Quinox (SI-Scheme 1)^81,82^ chiral ligands (**L19–27**) (SI-Table 3). To our delight, the enantioselectivity was improved to 9:1 e.r. with (*S*)-*t*Bu-*i*Quinox **L27**, presumably by favorable π-stacking of the substrate aryl group and the isoqunoline ring of the *i*Quinox ligand.

After establishing the optimal reaction conditions (Table 1, entry 14) and achieving the enantioselectivity with (*S*)-*t*Bu-*i*Quinox **L27** (SI-Table 3), we proceeded to evaluate the substrate scope of the enantioselective intramolecular Ni-catalyzed Heck reaction with variations at the quaternary center (R_1_), the amide nitrogen (R_2_), and the aryl halide (R_3_) (Table 2). We were pleased to find that a variety of aryl bromides and aryl iodides could undergo this Ni-catalyzed Heck cyclization to furnish the phenanthridinone derivatives in good to excellent yields with good enantioselectivities. Moreover, most reactions were completed within 3 h, which is roughly 7 times faster than with the Pd catalyst.^59^ Both aryl halides demonstrated compatibility with a simple achiral ligand such as bipy; however, aryl iodides showed better reactivity when employing chiral ligand **L27**. The higher reactivity of aryl iodides is consistent with the weaker C–I bond being more susceptible to oxidative addition than the C–Br bond of the aryl bromides.^83^ In addition, we believe it correlates to the complex nature of the iodide effect that seems to increase substrate reactivity.^66−68^

**Table 2:**
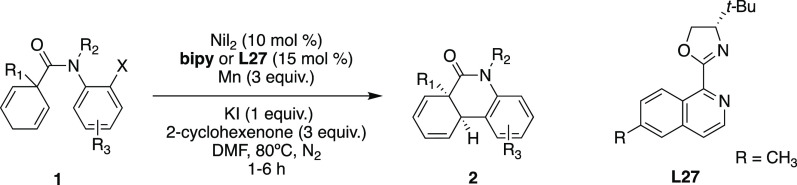

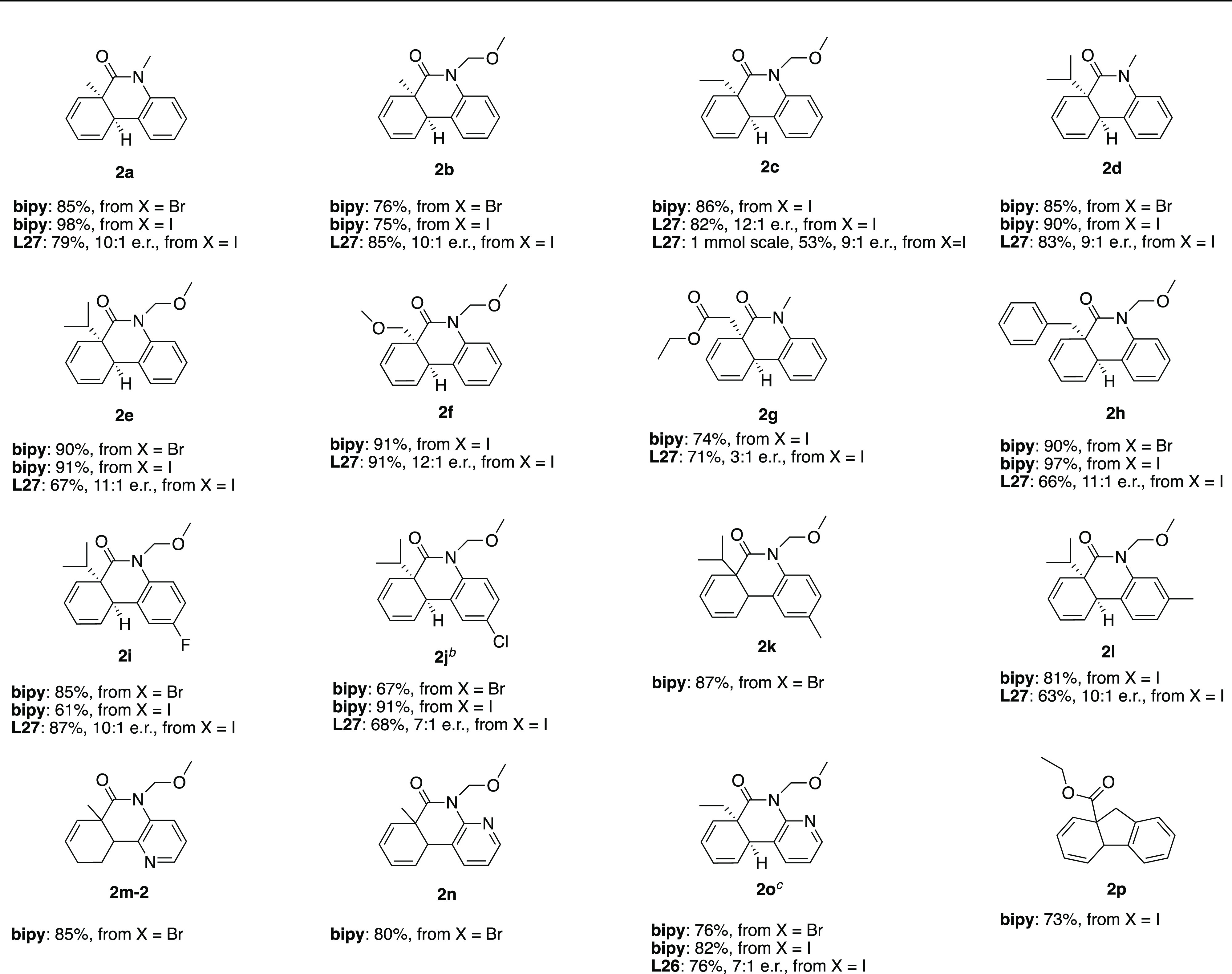
Substrate Scope^a^

a Unless otherwise noted, the reaction of aryl halide **1** or **1-I** (1 equiv) was carried out with NiI_2_ (10 mol %), bipy or **L27** (15 mol %), Mn (3 equiv), KI (1 equiv), 2-cyclohexenone (3 equiv), and DMF (0.08 M) at 80 °C. The yield of **2** is the isolated yield.

b At 65 °C.

c With **L26** (R = F).

A range of alkyl (R_1_) groups were well-tolerated and showed reactivity comparable to that of the the analogous Pd version,^59^ including methyl (**2a**, **2b**, **2m**, and **2n**), ethyl (**2c** and **2o**), and isopropyl (**2d**, **2e**, and **2i**–**l**) groups. A larger scale (1 mmol) enantioselective Heck reaction converting **1c-I** to **2c** demonstrated a slight reduction in reaction yield and a modest decrease in enantioselectivity. We were particularly delighted to achieve higher enantioselectivity for the isopropyl (**2e**) derivative (11:1) than with our previous Pd-catalyzed method (7:1). Functional groups such as ether (**2f**) and ester (**2g** and **2p**) and large benzyl groups (**2h**) were generally well-tolerated. As with the Pd catalyst report,^59^ only tertiary amides (R_2_ = Me or MOM) were successful in this intramolecular Heck reaction. Our previous experimental work with palladium^59^ suggests the secondary amides experience an *ortho* effect^65,84,85^ with a stable six-membered ring chelation event between the amide oxygen and the metal after oxidative addition to the aryl halide. Next, we examined aryl halide substitution (R_3_) and found a variety of aryl ring substituents were tolerated, including the 2-F, 2-Cl, 2-Me, and 3-Me derivatives (**2i**–**l**, respectively). The formation of a protodechlorinated side product was observed with the chloro analogue (**2j**) in 22% yield for X = Br, which results from oxidative addition of nickel into the C–Cl bond. Formation of this side product was decreased by decreasing the reaction temperature to 65 °C. When X = Br for **2j**, a 67% yield was obtained at the lower temperature, and when X = I for **2j**, a 91% yield was achieved. The higher yield of the iodo derivative is likely due to the greater difference in reactivity of the Ar–I and Ar–Cl bonds versus the Ar–Br and Ar–Cl bonds. The enantiomeric ratio for **2j** was lower, but a similar lower enantiomeric ratio was seen in our studies of the same enantioselective desymmetrizing Heck reaction with palladium.^59^ Noteworthy was the tolerance of pyridine rings, which afforded **2m**–**o** derivatives in very good yields, albeit with lower er (**2o**). Interestingly, pyridine bromide **1m** delivered exclusively the cyclized alkene product (**2m-2**). We speculate this may be the result of the initial 1,3-diene product isomerizing to the 1,4-diene product and the resulting conjugation with the pyridine ring promoting reduction with the Ni–H complex before reaction with the cyclohexenone sacrificial alkene. It must be noted that, in our phenanthridinone substrate, the presence of heterocyclic rings was not compatible with the Pd-catalyzed conditions,^59^ thereby illustrating another benefit of the Ni-catalyzed Heck version. Although enantioselectivity was not studied with bromopyridine derivatives **2m-2** and **2n**, iodopyridine **2o** did afford enantioselectivity. Lastly, tricyclic 6-5-6 ring system **2p** was efficiently formed in good yield.^86^ Consistent with our previous report,^59^ removal of the MOM amide protecting group was achieved with TMS-I generated in situ^87^ for most Heck products, except for pyridine analogues for which BCl_3_ was used to deprotect the MOM group (SI-Table 4).

### Mechanistic Studies

We performed mechanistic studies to shed light on the reaction process (Scheme 1). We first analyzed the role of the reducing metal, Mn primarily, but also the Zn used in the early reaction development. When manganese was removed from the reaction mixture, the reaction was completely hindered, suggesting the catalytic cycle is not initiated by a Ni(II) catalyst (eq 1). Using 10 mol % Ni(COD)_2_ in the absence of Mn afforded the cyclized diene in low yield (8%) (eq 2), the expected result for one round of catalyst activity. Under identical reaction conditions with the addition of 3 equiv of Mn, 78% of product was formed, which indicates the importance of Mn to turn over the catalytic cycle. In the context of this catalytic process, we speculated that an arylmanganese intermediate (ArMnBr or ArMnI), if formed,^88,89^ could create an aryl–Ni intermediate via nucleophilic addition instead of direct oxidative addition of Ni into the aryl halide. In addition, insertion of Mn into the Ar–X bond could facilitate the formation of protodehalogenated species. To assess the possibility of ArMnX intermediates, both aryl bromide **1e** and aryl iodide **1e-I** were used as substrates for stoichiometric reactions with Mn powder (eq 3a). Both reactions were quenched with D_2_O after 3 h, and the mixtures allowed to stir for an additional 18 h. Deuterated dehalogenated side product **D**_**1**_**2e-1** was formed in only 13% yield and presumably from an ArMnI intermediate. When stoichiometric reactions were performed with Zn, we observed deuterated side product **D**_**1**_**2e-I** in 82% yield (eq 3b). The additional unknown side product was presumed to be the cyclized alkene, based on the GC retention time and the MS data (Supporting Information). The basis for this cyclization is unclear but may be the result of minor Ni or Pd contamination of the commercial Zn reagent. Overall, these results illustrate protodehalogenation reactions are likely with the aryl iodide substrates, but less relevant to the aryl bromide reactions. Given the short reaction times, it is likely the Mn oxidative addition has a limited impact in hampering the desired Heck product formation. However, insertion of Zn into the Ar–I reaction is likely contributing to some of the protodeiodination side product, **2e-1**.^90−92^ Therefore, the main function of Mn in our reaction is to create a reducing environment for Ni(II) salts to form the necessary zerovalent nickel species, thus making it a better reductant.

**Scheme 2:**
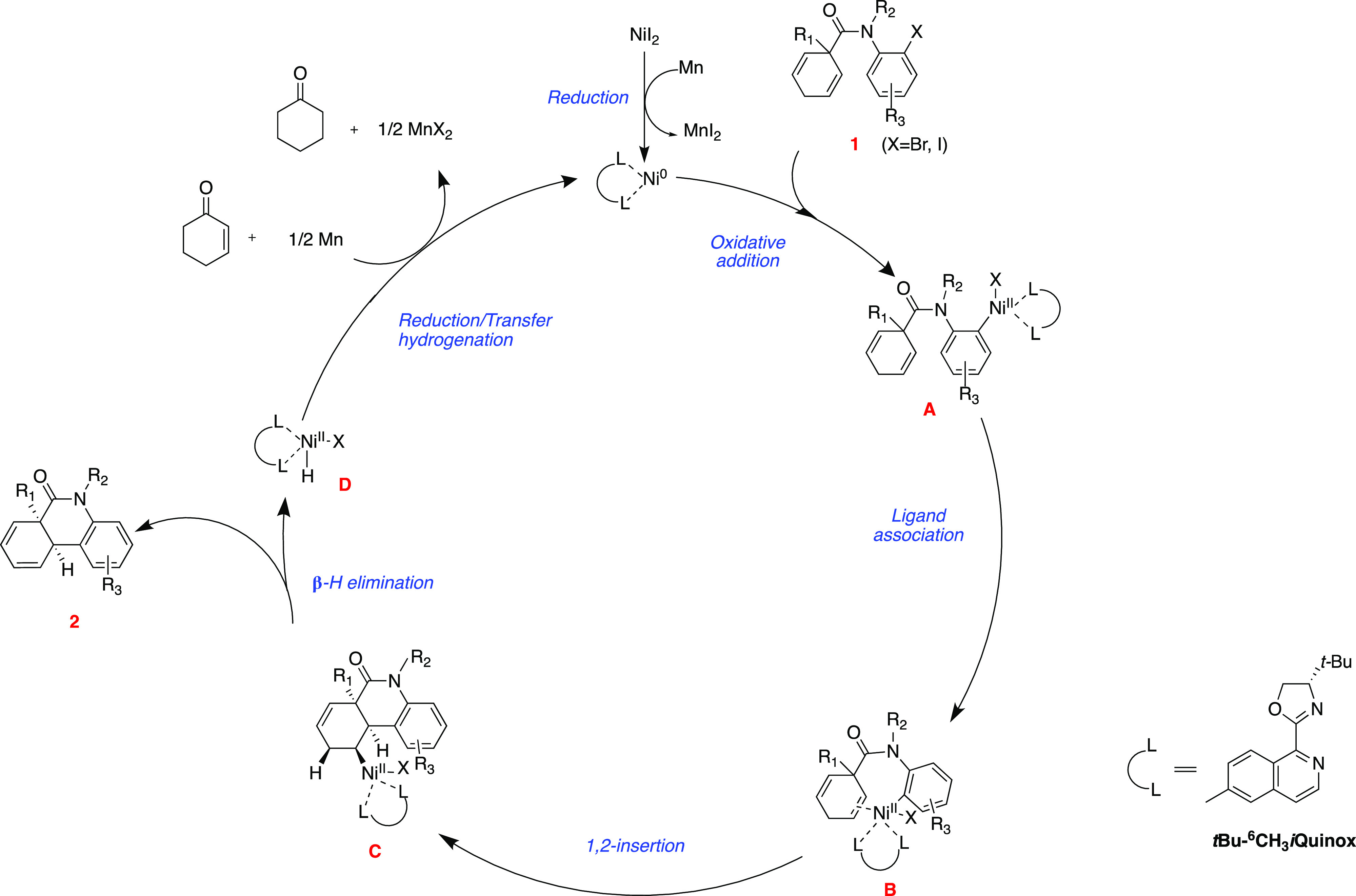
Proposed Reaction Mechanism

The role of 2-cyclohexenone as a sacrificial alkene was the next reaction component to be analyzed. As noted earlier, it was introduced to remove Ni–H intermediates and reduce the amount of protodehalogenation (**2**-**1**) and alkene (**2**-**1**) side products. Cyclohexanone was detected and quantified (1.2 equiv) in the gas chromatographic analysis of the reactions, demonstrating that the enone system was reduced. To test for a potential competing reduction of the sacrificial alkene by Mn,^93^ 2-cyclohexenone was stirred and heated at 80 °C with Mn in DMF (eq 4). The formation of cyclohexanone was not observed, which confirms that the Mn is not responsible for the enone reduction. Finally, adding TEMPO (1 equiv) did not impact the reaction, suggesting that the formation of cyclized diene product **2e** occurs through a nonradical two-electron pathway (eq 5). Therefore, we propose a traditional two-electron Heck reaction mechanism (Scheme 2). The low-valent Ni(0) species generated under reductive conditions undergoes oxidative addition with aryl halide **1** to afford Ni(II) intermediate **A**. Ligand association of intermediate **A** followed by an intramolecular migratory insertion produces alkyl-Ni(II)-Ar species **C**. A *syn*-coplanar position of nickel with a β-hydrogen allows for efficient β-hydride elimination to afford product **2** and N(II)–XH species **D**. The Ni(0) catalyst is regenerated upon Mn reduction. Some of the Ni(II)–H is presumably converted to a Ni–enolate by reaction with 2-cyclohexenone and then subsequently reduced with Mn to Ni(0). Formation of the cyclohexanone, which was detected in our mechanistic studies, could occur by protonation of the Ni–enolate intermediate via a Ni(II)–H complex also present in solution.^94^

### Computational Study

An understanding of the basis for the enantioselectivity in the catalytic cycle was developed through a computational study of the key 1,2-migratory insertion event taking proposed complex **B** to **C** (Scheme 2) for substrate **2a** (R_1_ and R_2_ = Me; R_3_ = H). We initially assumed this step would be rate-determining and stereodetermining. The Gibbs free energy of activation for this step with bipyridine ligand was calculated to be quite low at 1.9 kcal/mol, and the reaction is very exothermic (17.5 kcal/mol) and is unlikely to be reversible.

**Figure 1:**
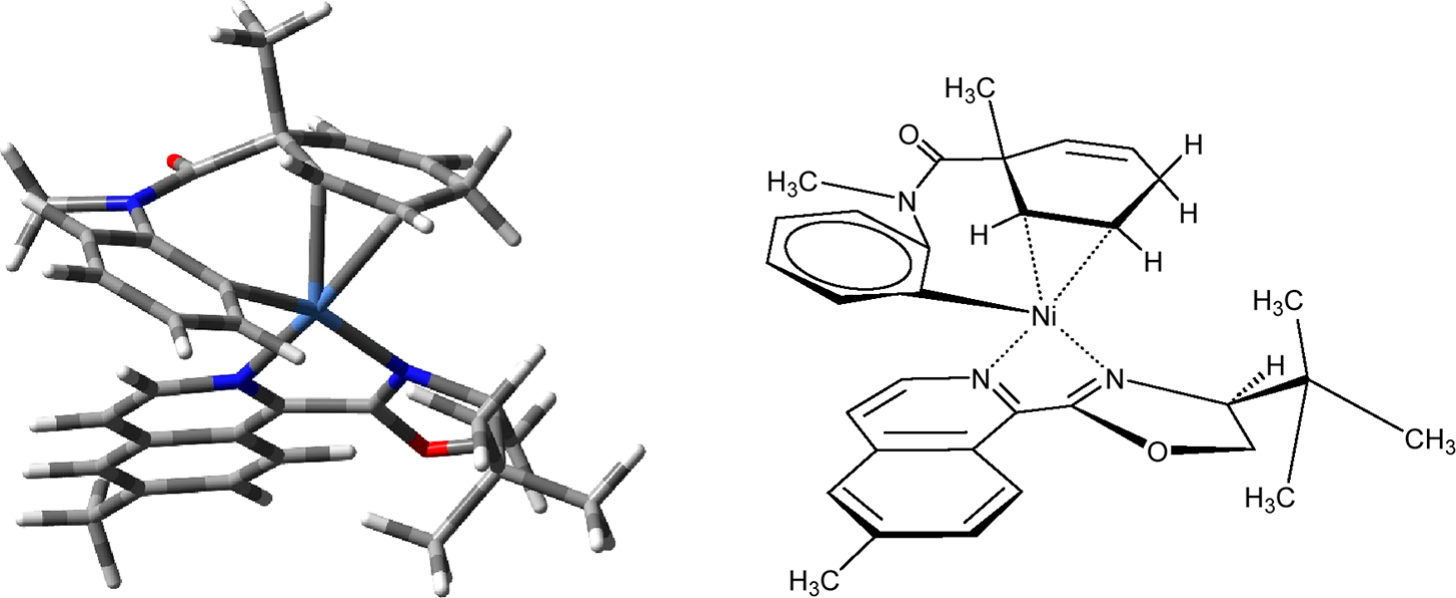
Lowest-energy transition state for 1,2-migratory insertion of **B** to **C**.

To better understand the asymmetric induction, a study of the transition state of the 1,2-migratory insertion with **L27** was conducted considering eight possible geometries: the bidentate chiral ligand in either of two possible binding positions (180° rotated), addition of the aryl group to either alkene of the cyclohexadiene (*pro-R* or *pro-S*), and either possible helical twist of the amide in the transition state. These variations were explored for intermediates **B** and **C** as well as the transition structure between them. The results are shown in SI-Table 5 and SI-Figure 1.

The lowest-energy structures for intermediate **B** led to high energy barriers. In fact, of the eight stereoisomeric transition structures, only two had energies of <24 kcal/mol above the global minimum. These two transition structures lay 13.4 and 17.2 kcal/mol above the global minimum for intermediate **B**, although only 2.2 and 6.0 kcal/mol, respectively, above the corresponding stereoisomers of intermediate **B**, which were both (coincidentally) 11.2 kcal/mol above the global minimum. These two pathways differed by only a 180° flip of the bidentate chiral ligand and thus corresponded to the same stereochemical outcome of the reaction. The exothermicity (16.3 kcal/mol) closely matched that of the achiral bipyridine case.

Assuming that the barriers for the reaction steps leading up to intermediates **B** are low, and that the migratory insertion is rate-determining, the pathway having the lowest overall barrier to migratory insertion should dominate. Even if the pathway with the second highest barrier were to contribute, the stereochemical outcome would not be affected. The remaining pathways, leading to different stereochemical outcomes, all involve much higher overall barriers.

The predicted stereochemical outcome from the computational analysis with **2a**, R for the quaternary carbon and R for the tertiary bridgehead carbon, with the (*S*)-**L27** ligand (Figure 1) matches in a relative sense the stereochemistry seen from our previous Pd-catalyzed Heck intramolecular desymmetrizing reactions.^59^ Products generated for both reactions did have the same major enantiomer formed on the basis of chiral HPLC retention times.

However, the energy difference between the favored pathway yielding the observed major product and lowest-energy alternate pathway yielding the opposite stereochemistry is notably large (10.9 kcal/mol). If the 1,2-migratory insertion (**B** to **C** in Scheme 2) solely controls the stereochemical outcome, as we had supposed, then the selectivity would be far greater than observed. We are thus forced to conclude that this simple explanation of the stereochemical outcome is inadequate, and in fact, there is good reason to suspect as much.

The foregoing analysis presumes that interconversion of the different possible conformations of the initial amide **1** is rapid compared to the rate of reaction (the typical Curtin–Hammett condition). However, this hindered amide is twisted, and axially chiral, although presumably racemic. Furthermore, interconversion of the enantiomers is likely to have a fairly higher barrier, quite likely in the neighborhood of 20 kcal/mol.^95^ Consequently, the reaction is likely to experience some degree of dynamic kinetic resolution, as detailed below, and has been previously observed for similar Heck reactions.^96^

**Scheme 3:**
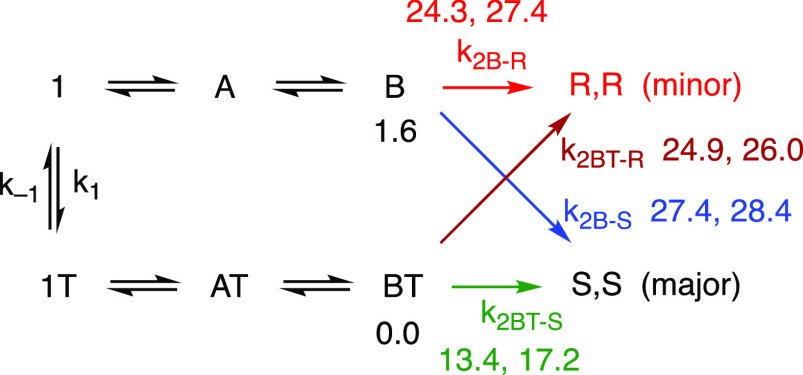
Kinetics of the Reaction in Scheme 2, Assuming That Isomerization of the Amide (*k*_1_/*k*_–1_ process) Can Be Slow Calculated energies are shown in kilocalories per mole (transition state energies for *k*_2B-R_, *k*_2BT-S_, *k*_2B-S_, and *k*_2BT-R_, minima for **B** and **BT**).

The eight aforementioned conformations of intermediate **B** differ by the orientation of the bidentate ligand, by which alkene the nickel complexes, and by the twist of the amide. If we assume that isomerization of the former two parameters is rapid, but that amide twist interconversion is potentially slow, we arrive at Scheme 3.

For the sake of simplicity, we do not specify whether the amide twist isomerization occurs in free amide **1**, in intermediate **A**, and/or in intermediate **B**, because it does not matter for this purpose. The series of structures **1**, **A**, and **B** have one twist of the amide, and this “**B**” represents the four isomers of **B** that were calculated and have this twist (**R**, **RA**, **RF**, and **RAF** in SI-Table 5 and SI-Figure 1). The series of structures **1T**, **AT**, and **BT** represent the corresponding structures with the opposite amide twist (including **RT**, **RAT**, **RFT**, and **RAFT** in SI-Table 5 and SI-Figure 1). The lowest energy of the four “**BT**” structures is the global minimum, while the lowest energy of the four “**B**” structures is 1.6 kcal/mol higher.

The **BT** structures lead via low barriers (13.4 or 17.2 kcal/mol) to the major observed stereochemistry of the product (*S,S* in this case). Much higher barriers (24.9 or 26.0 kcal/mol) lead to the unobserved product. The **B** structures, on the contrary, lead preferentially to the minor observed stereochemistry via barriers that are generally fairly high (24.3 or 27.4 kcal/mol above the global minimum), although significantly lower than the barriers leading to the major product stereochemistry (27.4 or 28.4 kcal/mol).

If the interconversion of the different amide twists (*k*_1_ and *k*_–1_) is rapid compared to *k*_2R_, i.e., if the *k*_1_ process has a barrier significantly below 24 kcal/mol, then the Curtin–Hammett condition applies, and the reaction would be expected to funnel essentially exclusively through the lowest overall barrier, shown in green, leading exclusively to one stereoisomer product. This is the condition we initially had assumed. On the contrary, if the amide twist interconversion is slow, having a barrier significantly higher than 24 kcal/mol, then one would expect the 50% that had the “desired” twist at the beginning (**1T**) would proceed to the (*S,S*) stereochemistry of the product, while the other 50%, having the opposite twist (**1**), would preferentially yield the (*R,R*) product. Little if any stereoselectivity would be observed overall. If the rate of the amide twist interconversion is competitive with that of the reaction, i.e., the *k*_1_ barrier is in the neighborhood of 24 kcal/mol, then one would expect reduced enantioselectivity, to the extent that **1** cannot isomerize to **1T** before reacting through **B** to yield the (*R,R*) product. This analysis offers two insights into the reaction process. First, it provides an explanation for why higher enantiomeric ratios are not achieved with the large calculated energy difference (∼11 kcal/mol) between the two diastereomeric transition state complexes in the 1,2-migratory insertion, which might otherwise be expected to afford enantiomeric ratios of >99:1. Second, it leads to the interesting notion that the stereoselectivity of this reaction could be improved by an increase in the barriers for the migratory insertion or, alternatively, a decrease in the barriers for the amide bond rotation. A simultaneous and equivalent change in both the amide rotamer barrier and the migratory insertion barrier would not help, so merely decreasing the temperature is unlikely to improve enantioselectivity. To date, experimental work concurs; attempts to improve the enantioselectivity by decreasing the reaction temperature have failed (data not shown)

## Conclusion

In summary, we have developed the first example of an enantioselective intramolecular Ni-catalyzed synthesis of a heterocyclic system with a quaternary stereocenter using the Birch–Heck sequence. This work represents a rare example of a Ni-catalyzed intramolecular Heck reaction to form a six-membered ring with an all-carbon quaternary center and demonstrates the broadest substrate scope to date with good to very good levels of enantioselectivity. In comparison with the Pd-catalyzed version of the reaction, the alternative Ni-catalyzed desymmetrization reaction is much faster (1 h vs 20 h) than the analogous Pd version and can be applied to a wide range of substrates giving good to excellent yields. It affords enantioselectivities comparable to that of the Pd/BINAP Heck reaction^59^ with the use of a newly synthesized chiral *i*Quinox-type bidentate ligand **L27**. Notably, during reaction optimization, we were able to control unwanted Ni–H reductions, including a protodehalogenation side reaction. As such, this work presents a direct and valuable comparison of the performance of nickel and palladium catalysts, which should facilitate the application of Ni catalysis to traditional Heck transformations.

## Experimental Section

### General Procedures

All reactants and reagents were commercially available and used without further purification unless otherwise indicated. Anhydrous tetrahydrofuran (THF) was obtained by distillation from benzophenone-sodium under argon. All reactions were carried out under an inert atmosphere of argon in flame-dried glassware unless otherwise indicated. Concentrated refers to the removal of solvent with a rotary evaporator at normal water aspirator pressure. Concentrated under high vacuum refers to removal of solvent with a direct-drive rotary vane vacuum pump. Thin layer chromatography (TLC) was performed using silica gel 60 Å precoated aluminum-backed plates (0.25 mm thickness) with a fluorescent indicator. Developed TLC plates were visualized with UV light (254 nm) and KMnO_4_ spray. Flash column chromatography was conducted with the indicated solvent system using normal phase silica gel (60 Å, 230–400 mesh). Yields refer to chromatographically and spectroscopically pure (>95%) compounds, except as otherwise indicated.

^1^H and ^13^C NMR spectra were recorded on a Bruker Avance III 400 instrument at 400 and 100 MHz, respectively. Chemical shifts are reported in δ values (parts per million) relative to an internal reference [0.05% (v/v)] of tetramethylsilane (TMS) for ^1^H NMR or the solvent signal, chloroform (CDCl_3_) or DMSO-*d*_6_, for ^13^C NMR. NMR analysis of the tertiary amides was conducted at increased temperatures (77–100 °C) in DMSO-*d*_6_ due to the presence of atropisomers. Peak splitting patterns in the ^1^H NMR spectra are reported as follows: s, singlet; bs, broad singlet; d, doublet; t, triplet; q, quartet; hept, heptet; dd, doublet of doublets; ddd, doublet of doublets of doublets; dt, doublet of triplets; dq, doublet of quartets; m, multiplet. ^13^C NMR experiments were conducted with the attached proton test (APT) pulse sequence. ^13^C multiplicities are reported as δ_u_ (up) for methyl and methine and δ_d_ (down) for methylene and quaternary carbons.

GC-MS analyses were performed with an Agilent 6890 GC instrument and a Hewlett-Packard 5973 EI-MS detector fitted with a 30 m × 0.25 mm column filled with cross-linked 5% PH ME siloxane (0.25 μm film thickness); the gas pressure was 7.63 psi of He. Analysis of samples involved either heating from 70 to 250 °C (10 °C/min) and then being held at 250 °C for 5 min (method A) or heating from 175 to 250 °C (25 °C/min) and then being held at 250 °C for 2 min (method B). Melting points were measured on a Stanford Research Systems MPA160 melting point apparatus and are uncorrected. HPLC analysis was conducted using an Agilent 1100 instrument fitted with a DAD at 254 nm using a CHIRACEL OD-H 4.6 mm × 250 mm, 5 μm column, run under the specified conditions. HRMS were collected at the University of Delaware using a Q-Exactive Orbitrap instrument with an ESI source in positive mode or a Waters GCT Premier instrument equipped with a LIFDI (liquid field desorption ionization). Optical rotations were determined on a PerkinElmer 341 polarimeter at Villanova University at 589 nm and 20.0 °C.

### Computational Methods

All calculations were carried out with Gaussian 16^97^ and using density functional theory (DFT) in the gas phase. We chose the specific configuration applied successfully by Houk, Chen, and co-workers to a related Ni(II) migratory insertion.^40^ This procedure involves geometry optimization using the B3LYP hybrid functional,^98^ the def2-SVP basis set,^99,100^ and the Grimme D3 empirical dispersion correction^101^ with Becke–Johnson damping,^102^ followed by a single-point energy correction using the TZVPP basis set. After each optimization [conducted with fopt = (calcfc,tight) or fopt = (calcfc,ts,tight)], a subsequent frequency calculation was performed to confirm the nature of the stationary point as a minimum (NImag = 0) or a transition structure (NImag = 1) and to obtain thermodynamic corrections. Gibbs free energies at 298 K were used for analysis, but essentially the same picture would be obtained using either enthalpies or electronic energies, as the thermodynamic corrections to the energy barriers and differences were small, as would be expected.

The structures computed were those of intermediates **B** and **C** in Scheme 2, and the transition structure between them, for the case in which R_1_ = R_2_ = CH_3_, R_3_ = H, and L–L = (*R*)-*t*Bu-6-CH_3_-*i*Quinox. Eight major stereoisomers were computed, corresponding to the three major factors that could be varied one way or the other: (1) the orientation of the (*R*)-*t*Bu-6-CH_3_-*i*Quinox ligand (a given orientation, or flipped 180° to transpose the positions of the two coordinating nitrogen atoms), (2) which of the two alkenes in the cyclohexadiene unit coordinates to nickel, and (3) whether to twist the amide out of plane in one sense or the other. In a few cases, there were additional orientations of the *i*Quinox ligand, leading to similar energies. In addition, of course, there would be another eight equivalent structures with the *S* configuration at the carbon bearing the *tert*-butyl group, but these would have the same energies and were not computed. The amide functionality was in all cases restricted to the conformation illustrated in Scheme 2. Conformational depictions and calculation details are available in the Supporting Information.

### Birch Reduction/Alkylation

#### General Procedure A

A flame-dried three-necked round-bottom flask with a stir bar, connected to a Dewar condenser, under argon, was charged with benzoic acid (1.0 mmol, 1.0 equiv) that was dissolved in THF (0.4 mL, 2.5 M) and cooled to −78 °C. Ammonia (7 mL, 0.14 M) was distilled into the flask, and lithium (4.0 mmol, 4.0 equiv) was added in small pieces until a dark blue color was maintained for 30 min. Isoprene was added dropwise to quench the excess lithium and produce a bright yellow opaque solution. An alkylating agent (2.0 mmol, 2.0 equiv) was added slowly dropwise. When the addition was complete, the reaction mixture was maintained at −78 °C while the color faded to white/off-white over 1 h. The reaction mixture was then warmed to room temperature, and the ammonia was allowed to evaporate. Once the ammonia had evaporated, the reaction was quenched with water and the mixture washed with diethyl ether. The aqueous layer was acidified with 6 N HCl (until the pH reached ∼1) and then extracted with diethyl ether. The combined organic layers were washed with Na_2_S_2_O_4_ and brine, dried with MgSO_4_, and concentrated in vacuo.

#### General Procedure B

A flame-dried flask with a stir bar, connected to a Dewar condenser, under argon, was charged with benzoate ester (1.0 mmol, 1.0 equiv), THF (0.4 mL, 2.5 M), and *t*BuOH (1.1 mmol, 1.1 equiv) and cooled to −78 °C. Ammonia (7 mL, 0.14 M) was distilled into the flask, and lithium (2.0 mmol, 2.0 equiv) was added in small pieces until a dark blue color was maintained for 30 min. Isoprene was added dropwise to quench the lithium and produce a bright yellow opaque solution. An alkylating agent (1.1 mmol, 1.1 equiv) in THF (0.4 mL, 2.5 M) was added slowly dropwise. When the addition was complete, the reaction mixture was maintained at −78 °C while the color faded to white/off-white over 1 h. The reaction mixture was then warmed to room temperature, and the ammonia was allowed to evaporate under a stream of argon. Once the ammonia had evaporated, the reaction was quenched with water and the mixture extracted with Et_2_O (5 × 7 mL/mmol). The combined organic layers were washed with brine, dried with MgSO_4_, concentrated in vacuo, and purified with flash chromatography on silica gel.

*1-Methylcyclohexa-2,5-diene-1-carboxylic acid* (**S1a**). Using Birch reduction/alkylation procedure A with benzoic acid (3.00 g, 24.6 mmol, 1.0 equiv) and iodomethane (3.06 mL, 49.1 mmol, 2.0 equiv) in THF (9.8 mL, 2.5 M) afforded **S1a** (3.18 g, 23.0 mmol) in 94% yield as a white solid: mp 31.2–33.4 °C. Spectral data were in accordance with the literature.^103^

*1-Ethylcyclohexa-2,5-diene-1-carboxylic acid* (**S1b**). Using Birch reduction/alkylation procedure A with benzoic acid (3.03 g, 24.6 mmol, 1.0 equiv) and bromoethane (3.70 mL, 49.6 mmol, 2.0 equiv) in THF (9.8 mL, 2.5 M) provided **S1b** (3.74 g, 24.6 mmol) in 100% yield as a clear colorless oil. Spectral data were in accordance with the literature.^104^

*1-Isopropylcyclohexa-2,5-diene-1-carboxylic acid* (**S1c**). Using Birch reduction/alkylation procedure A with benzoic acid (3.01 g, 24.6 mmol, 1.0 equiv) and 2-iodopropane (4.82 mL, 49.2 mmol, 2.0 equiv) in THF (9.8 mL, 2.5 M) afforded isopropyl diene acid **S1c** (7.07 g, 42.5 mmol) in 96% yield as a white solid: mp 74.0–76.0 °C. Spectral data were in accordance with the literature.^59^

*1-(Methoxymethyl)cyclohexa-2,5-diene-1-carboxylic acid* (**S1d**). Using Birch reduction/alkylation procedure A with benzoic acid (3.02 g, 24.76 mmol, 1.0 equiv) and chloromethyl methyl ether (3.76 mL, 49.5 mmol, 2.0 equiv) in THF (9.9 mL, 2.5 M) afforded **S1d** (3.74 g, 22.2 mmol) in 90% yield as a white solid: mp 66.9–70.5 °C. Spectral data were in accordance with the literature.^59^

*1-(2-Ethoxy-2-oxoethyl)cyclohexa-2,5-diene-1-carboxylic acid* (**S1e**). Using Birch reduction/alkylation procedure A with benzoic acid (2.00 g, 16.4 mmol, 1.0 equiv) and ethyl 2-chloroacetate (3.51 mL, 32.8 mmol, 2.0 equiv) in THF (6.6 mL, 2.5 M) afforded **S1e** (3.41 g, 16.2 mmol) in 99% yield as a pale-yellow liquid that solidified to white crystals upon cooling: mp 63.5–66 °C. Spectral data were in accordance with the literature.^59^

*1-Benzylcyclohexa-2,5-diene-1-carboxylic acid* (**S1f**). Using Birch reduction/alkylation procedure A (2.33 g, 19.1 mmol, 1.0 equiv) and benzyl chloride (4.38 mL, 38.1 mmol, 2.0 equiv) in THF (7.6 mL, 2.5 M) afforded **S1f** (3.83 g, 17.9 mmol) in 94% yield as a white crystalline solid: mp 71.9–75.0 °C. Spectral data were in accordance with the literature.^104^

*Ethyl-1-(2-iodobenzyl)cyclohexa-2,5-diene-1-carboxylate* (**S1g**). Using Birch reduction/alkylation procedure B with ethyl benzoate (5.25 g, 35.0 mmol, 1.0 equiv) and 2-iodobenzyl bromide (11.4 g, 38.5 mmol, 1.1 equiv) in THF (14 mL, 2.5 M) afforded **S1g** (10.98 g, 29.82 mmol) in 85% yield as a clear colorless oil. Spectral data were in accordance with the literature.^86^

#### General Aniline Methylation Procedure A

A flame-dried round-bottom flask with a stir bar, under argon, was charged with aniline (1.0 mmol, 1.0 equiv) dissolved in THF (2 mL, 0.5 M). The solution was cooled to −78 °C, and then *n*BuLi (2.5 M in hexanes, 0.3 mL, 0.7 mmol, 0.7 equiv) was added slowly dropwise over 15 min. After the reaction mixture was stirred at −78 °C for 30 min, iodomethane (1.1 mmol, 1.1 equiv) was added over 5 min. The solution was stirred at −78 °C for 1 h and then at room temperature overnight. The reaction was quenched with water, and the mixture extracted with Et_2_O (3 × 5 mL/mmol). The organic layers were combined and washed with brine, dried over MgSO_4_, and concentrated in vacuo. The crude products were purified by column chromatography.

#### General Aniline Methylation Procedure B

A flame-dried round-bottom flask with a stir bar, under argon, was charged with aniline (1.0 mmol, 1.0 equiv) dissolved in THF (2 mL, 0.5 M) and cooled to −78 °C. Using an automated syringe pump, MeLi (1.6 M in Et_2_O, 0.6 mL, 1.0 mmol, 1.0 equiv) was added dropwise over 45 min. After the reaction mixture was stirred at −78 °C for 30 min, iodomethane (1.1 mmol, 1.1 equiv) was dissolved in THF (0.3 mL, 3.3 M) and added dropwise to the flask over 10 min at −78 °C. The solution was stirred at −78 °C for 1 h and then warmed to room temperature overnight. The reaction was quenched with saturated aqueous NH_4_Cl (1.6 mL/mmol), and the mixture extracted with Et_2_O (3 × 1.6 mL/mmol). The organic layers were combined and washed with brine, dried over MgSO_4_, and concentrated in vacuo. The crude products were purified by column chromatography.

*2-Bromo-N-methylaniline.* Using general aniline methylation procedure A with 2-bromoaniline (3.04 g, 17.7 mmol, 1.0 equiv) in THF (35 mL, 0.5 M) afforded the crude product that was purified by column chromatography (silica, 10:1 hexanes/EtOAc) to afford pure 2-bromo-*N*-methylaniline (1.98 g, 10.6 mmol) in 60% yield as a clear yellow oil. Spectral data were in accordance with the literature.^105^

*2-Iodo-N-methylaniline.* Using general aniline methylation procedure B with 2-iodoaniline (4.0 g, 18.3 mmol, 1.0 equiv) in THF (37 mL, 0.5 M) afforded the crude product that was purified by column chromatography (silica, 60:1 hexanes/EtOAc) to afford pure 2-iodo-*N*-methylaniline (3.56 g, 15.3 mmol) in 83% yield as a yellow-orange oil. Spectral data were in accordance with the literature.^105^

#### General Benzamide Synthesis Procedure

In a round-bottom flask with a stir bar, oxalyl chloride (2.2 mmol, 2.2 equiv) was dissolved in DCM (5.5 mL, 0.4 M) and a catalytic amount of DMF (1.4 μL, 0.02 equiv) was added. The Birch product (1.0 mmol, 1.0 equiv) was dissolved in DCM (2.5 mL, 0.4 M) and added dropwise to the flask. The reaction mixture was refluxed under argon for 1 h until it turned deep yellow. Once the reaction had reached completion, as judged by GC-MS analysis, the mixture was concentrated under vacuum to remove excess oxalyl chloride.

In a round-bottom flask with a stir bar, 2-haloaniline or purified 2-halo-*N*-methylaniline (1.05–1.3 mmol, 1.05–1.3 equiv) was dissolved in DCM (2.5 mL, 0.4 M) and cooled to 0 °C. Triethylamine (2.5 mmol, 2.5 equiv) was added dropwise followed shortly thereafter by the addition of the acid chloride (1.0 mmol, 1.0 equiv) in DCM (2.5 mL, 0.4 M). The mixture was allowed to warm to room temperature and react overnight. The reaction mixture was diluted with DCM, washed with saturated NaHCO_3_, 1 N HCl (not used for pyridine- and pyrimidine-containing substrates), and brine, and then dried with MgSO_4_. The crude product was concentrated under vacuum and purified by column chromatography.

*N-(2-Bromophenyl)-N,1-dimethylcyclohexa-2,5-diene-1-carboxamide* (**1a**). Using the general benzamide synthesis procedure, diene acid **S1a** (1.15g, 7.33 mmol, 1.0 equiv) in DCM (18.3 mL, 0.4 M) reacted with 2-bromo-*N*-methylaniline (1.77 g, 9.53 mmol, 1.3 equiv) in DCM (23.8 mL, 0.4 M) to afford the crude product that was purified by column chromatography (silica, 4:1 hexanes/EtOAc) to afford pure **1a** (2.08 g, 6.79 mmol) in 93% yield as a white solid: mp 42.2–44.4 °C; ^1^H NMR (400 MHz, CDCl_3_) δ 7.46 (d, *J* = 7.5 Hz, 1H), 7.23–7.17 (m, 1H), 7.13–7.02 (m, 2H), 5.59 (d, *J* = 10.1 Hz, 1H), 5.48–5.38 (m, 2H), 4.94 (d, *J* = 10.1 Hz, 1H), 3.08 (s, 3H), 2.22 (d, *J* = 23.1 Hz, 1H), 1.83 (d, *J* = 23.0 Hz, 1H), 1.26 (s, 3H); ^13^C{^1^H} NMR (101 MHz, CDCl_3_) δ_u_ 133.1, 131.2, 131.1, 128.9, 128.3, 127.8, 123.5, 121.7, 39.0, 29.3; δ_d_ 174.2, 143.3, 124.6, 45.8, 25.9; GC (method B) *t*_R_ = 2.272 min; EI-MS *m*/*z* (%) 305 (M+, 1), 214 (45), 185 (18), 134 (100), 105 (13), 93 (53), 77 (46), 65 (8), 51 (8); HRMS (ESI) calcd for C_15_H_17_ONBr [M + H]^+^ 306.0494, found 306.0493.

*N-(2-Iodophenyl)-N,1-dimethylcyclohexa-2,5-diene-1-carboxamide* (**1a-I**). Using the general benzamide synthesis procedure, diene acid **S1a** (1.16 g, 7.42 mmol, 1.0 equiv) in DCM (18.6 mL, 0.4 M) reacted with 2-iodo-*N*-methylaniline (2.25 g, 9.65 mmol, 1.3 equiv) in DCM (24.1 mL, 0.4 M) to afford the crude product that was purified by column chromatography (silica, 4:1 hexanes/EtOAc) to afford pure **1a-I** (2.07 g, 5.86 mmol) in 79% yield as a white solid: mp 35.8–38.2 °C; ^1^H NMR (400 MHz, CDCl_3_) δ 7.80 (d, *J* = 7.9 Hz, 1H), 7.27–7.18 (m, 2H), 6.96 (t, *J* = 7.9 Hz, 1H), 5.64 (s, 1H), 5.56 (d, *J* = 9.9 Hz, 1H), 5.49 (s, 1H), 5.01 (s, 1H), 3.14 (s, 3H), 2.30 (d, *J* = 23.2 Hz, 1H), 1.94 (d, *J* = 23.2 Hz, 1H), 1.35 (s, 3H); ^13^C{^1^H} NMR (101 MHz, CDCl_3_) δ_u_ 139.4, 131.2, 130.2, 128.9, 128.8, 123.6, 121.6, 39.4, 29.4; δ_d_ 174.2, 146.6, 101.8, 45.8, 26.0; GC (method B) *t*_R_ = 2.608 min; EI-MS *m*/*z* (%) 353.0 (M+, 1), 260.9 (92), 243.9 (19), 230.9 (20), 202.8 (7), 182.1 (1), 134.0 (100), 105.0 (30), 91.0 (47), 77.0 (47), 64.0 (11), 51.0 (12); HRMS (ESI) calcd for C_15_H_17_ONI [M + H]^+^ 354.0355, found 354.0350.

*N-(2-Bromophenyl)-1-methylcyclohexa-2,5-diene-1-carboxamide* (**S2b**). Using the general benzamide synthesis procedure, diene acid **S1a** (0.505 g, 3.62 mmol, 1.0 equiv) in DCM (9.0 mL, 0.4 M) reacted with 2-bromoaniline (0.44 mL, 3.87 mmol, 1.1 equiv) in DCM (9.7 mL, 0.4 M) to afford the crude product that was purified by column chromatography (silica, 10:1 hexanes/EtOAc) to afford pure **S2b** (1.01 g, 3.46 mmol) in 95% yield as a clear colorless oil: ^1^H NMR (400 MHz, CDCl_3_) δ 8.32 (dd, *J* = 8.3, 1.6 Hz, 1H), 8.28 (s, 1H), 7.41 (dd, *J* = 8.1, 1.5 Hz, 1H), 7.25–7.16 (m, 1H), 6.89–6.80 (m, 1H), 5.97–5.87 (m, 2H), 5.76–5.67 (m, 2H), 2.86–2.65 (m, 2H), 1.34 (s, 3H); ^13^C{^1^H} NMR (101 MHz, CDCl_3_) δ_u_ 132.2, 129.7, 128.4, 126.1, 124.8, 120.9, 24.7; δ_d_ 172.8, 136.1, 113.1, 46.4, 26.0; GC (method B) *tR* = 2.373 min; EI-MS *m*/*z* (%) 291.1 (M + 1^+^, 5), 276.0 (4), 198.9 (9), 171.0 (11), 120.0 (26), 93.1 (100), 77.1(35), 65.1 (10), 51.1 (5); HRMS (ESI) calcd for C_14_H_15_ONBr [M + H]^+^ 292.0337, found 292.0331.

*N-(2-Iodophenyl)-1-methylcyclohexa-2,5-diene-1-carboxamide* (**S2b-I**). Using the general benzamide synthesis procedure, diene acid **S1a** (0.299 g, 2.16 mmol, 1.0 equiv) in DCM (6.5 mL, 0.4 M) reacted with 2-iodoaniline (0.615 g, 2.81 mmol, 1.3 equiv) in DCM (7 mL, 0.4 M) to afford the crude product that was purified by column chromatography (silica, 9:1 hexanes/EtOAc) to afford pure **S2b-I** (0.694 g, 2.05 mmol) in 99% yield as an off-white solid: mp 72.7–75.0 °C; ^1^H NMR (400 MHz, CDCl_3_) δ 8.33 (dd, *J* = 8.2, 1.6 Hz, 1H), 8.17 (s, 1H), 7.76 (dd, *J* = 8.0, 1.5 Hz, 1H), 7.39–7.30 (m, 1H), 6.82 (td, *J* = 7.6, 1.6 Hz, 1H), 6.09–5.99 (m, 2H), 5.87–5.78 (m, 2H), 3.00–2.76 (m, 2H), 1.44 (s, 3H); ^13^C{^1^H} NMR (101 MHz, CDCl_3_) δ_u_ 138.8, 129.7, 129.3, 126.1, 125.5, 120.9, 24.8; δ_d_ 173.0, 138.6, 89.0, 46.3, 26.2; GC (method B) *t*_R_ = 2.792 min; EI-MS *m*/*z* (%) 339.0 (M+, 13), 324.0 (9), 246.9 (35), 218.9 (20), 202.9 (4), 120.0 (39), 93.0 (100), 77.0 (39), 64.0 (8), 51.0 (5); HRMS (ESI) calcd for C_14_H_15_ONI [M + H]^+^ 340.0198, found 340.0197.

*1-Ethyl-N-(2-iodophenyl)cyclohexa-2,5-diene-1-carboxamide* (**S2c-I**). Using the general benzamide synthesis procedure, diene acid **S1b** (0.501 g, 3.26 mmol, 1.0 equiv) in DCM (8.2 mL, 0.4 M) reacted with 2-iodoaniline (0.935 g, 4.27 mmol, 1.3 equiv) in DCM (10.7 mL, 0.4 M) to afford the crude product that was purified by column chromatography (silica, 9:1 hexanes/EtOAc) to afford pure **S2c-I** (1.10 g, 3.11 mmol) in 95% yield as a white solid: ^1^H NMR (400 MHz, CDCl_3_) δ 8.31 (dd, *J* = 8.3, 1.6 Hz, 1H), 8.13 (s, 1H), 7.74 (dd, *J* = 7.9, 1.5 Hz, 1H), 7.32 (ddd, *J* = 8.5, 7.2, 1.5 Hz, 1H), 6.80 (td, *J* = 7.6, 1.6 Hz, 1H), 6.16–6.06 (m, 2H), 5.72 (dt, *J* = 10.4, 2.1 Hz, 2H), 2.96–2.73 (m, 2H), 1.87 (q, *J* = 7.5 Hz, 2H), 0.86 (t, *J* = 7.5 Hz, 3H); ^13^C{^1^H} NMR (101 MHz, CDCl_3_) δ_u_ 138.8, 129.2, 128.1, 127.8, 125.6, 121.1, 8.9; δ_d_ 172.8, 138.6, 89.1, 50.8, 29.5, 26.5; GC (method B) *t*_R_ = 3.101 min; EI-MS *m*/*z* (%) 353.1 (M+, 16), 324 (19), 246.9 (50), 218.9 (29), 202.9 (4), 120 (35), 107.0 (64), 91.0 (33), 79.0 (100), 65.0 (8).

*N-(2-Bromophenyl)-1-isopropyl-N-methylcyclohexa-2,5-diene-1-carboxamide* (**1d**). Using the general benzamide synthesis procedure, diene acid **S1c** (0.501 g, 3.00 mmol, 1.0 equiv) in DCM (7.5 mL, 0.4 M) reacted with 2-bromo-*N*-methylaniline (0.587 g, 3.153 mmol, 1.05 equiv) in DCM (7.9 mL, 0.4 M) to afford the crude product that was purified by column chromatography (silica, 20:1 hexanes/EtOAc) to afford pure **1d** (0.897 g, 2.70 mmol) in 90% yield as a white solid: mp 85.4–86.1 °C; ^1^H NMR (400 MHz, CDCl_3_) δ 7.50–7.43 (m, 1H), 7.15–7.01 (m, 3H), 5.54 (d, *J* = 10.3 Hz, 1H), 5.43 (d, *J* = 10.4 Hz, 1H), 5.25 (d, *J* = 10.3 Hz, 1H), 5.02 (d, *J* = 10.7 Hz, 1H), 3.09 (s, 3H), 2.45 (hept, *J* = 6.8 Hz, 1H), 2.25 (d, *J* = 23.2 Hz, 1H), 2.01 (d, *J* = 20.1 Hz, 1H), 0.80 (d, *J* = 6.7 Hz, 3H), 0.61 (d, *J* = 7.0 Hz, 3H); ^13^C{^1^H} NMR (101 MHz, CDCl_3_) δ_u_ 133.0, 130.4, 130.1, 128.9, 128.9, 127.9, 124.9, 123.9, 38.9, 35.9, 17.8, 17.1; δ_d_ 174.7, 143.8, 124.3, 54.0, 26.6; GC (method B) *t*_R_ = 2.692 min; EI-MS *m*/*z* (%) 333.1 (M – 1^+^, 2), 290.0 (23), 214.0 (55), 185.0 (16), 134.0 (100), 121.1 (9), 105.0 (72), 91.0 (9), 77.1 (78), 51.0 (8); HRMS (ESI) calcd for C_17_H_21_ONBr [M + H]^+^ 334.0807, found 334.0806.

*N-(2-Iodophenyl)-1-isopropyl-N-methylcyclohexa-2,5-diene-1-carboxamide* (**1d-I**). Using the general benzamide synthesis procedure, diene acid **S1c** (0.340 g, 2.05 mmol, 1.0 equiv) in DCM (5.1 mL, 0.4 M) reacted with 2-iodo-*N*-methylaniline (0.620 g, 2.66 mmol, 1.3 equiv) in DCM (6.7 mL, 0.4 M) to afford the crude product that was purified by column chromatography (silica, 9:1 hexanes/EtOAc) to afford pure **1d-I** (0.267 g, 1.64 mmol) in 84% yield as a white solid: mp 89.6–90.8 °C; ^1^H NMR (400 MHz, CDCl_3_) δ 7.74 (d, *J* = 7.9 Hz, 1H), 7.11 (d, *J* = 3.9 Hz, 2H), 6.93–6.84 (m, 1H), 5.66–5.34 (m, 2H), 5.27 (d, *J* = 10.4 Hz, 1H), 5.01 (s, 1H), 3.08 (s, 3H), 2.46 (hept, *J* = 6.9 Hz, 1H), 2.31–1.94 (m, 2H), 0.83 (d, *J* = 6.8 Hz, 3H), 0.62 (d, *J* = 6.9 Hz, 2H); ^13^C{^1^H} NMR (101 MHz, CDCl_3_) δ_u_ 139.4, 130.3, 129.6, 128.8, 128.8, 125.0, 124.3, 123.8, 39.4, 36.0, 18.1, 17.1; δ_d_ 174.8, 147.1, 101.5, 54.0, 26.7; GC (method B) *t*_R_ = 3.051 min; EI-MS *m*/*z* (%) 381.0 (M+, 2), 338.0 (20), 260.9 (75), 244.9 (5), 230.9 (15), 210.1 (14), 134.0 (100), 105.0 (80), 91.0 (9), 77.0 (39), 51.0 (8); HRMS (ESI) calcd for C_17_H_21_ONI [M + H]^+^ 382.0668, found 382.0664.

*N-(2-Bromophenyl)-1-isopropylcyclohexa-2,5-diene-1-carboxamide* (**S2e**). Using the general benzamide synthesis procedure, diene acid **S1c** (0.800 g, 4.81 mmol, 1.0 equiv) in DCM (12.0 mL, 0.4 M) reacted with 2-bromoaniline (1.08 g, 6.25 mmol, 1.3 equiv) in DCM (15.6 mL, 0.4 M) to afford the crude product that was purified by column chromatography (silica, 9:1 hexanes/EtOAc) to afford pure **S2e** (1.18 g, 3.68 mmol) in 79% yield as a yellow oil: ^1^H NMR (400 MHz, CDCl_3_) δ 8.42 (dd, *J* = 8.3, 1.6 Hz, 1H), 8.35 (s, 1H), 7.50 (dd, *J* = 8.0, 1.5 Hz, 1H), 7.29 (ddd, *J* = 8.6, 7.4, 1.5 Hz, 1H), 6.94 (td, *J* = 7.7, 1.6 Hz, 1H), 6.17–6.07 (m, 2H), 5.78 (dp, *J* = 10.6, 2.1 Hz, 2H), 2.81 (qd, *J* = 3.3, 1.8 Hz, 2H), 2.48 (hept, *J* = 6.9 Hz, 1H), 0.90 (d, *J* = 6.9 Hz, 6H); ^13^C{^1^H} NMR (101 MHz, CDCl_3_) δ_u_ 132.2, 128.5, 128.4, 126.7, 124.9, 121.3, 33.4, 17.7; δ_d_ 172.5, 136.1, 113.4, 54.4, 26.8; GC (method B) *t*_R_ = 2.897 min; EI-MS *m*/*z* (%) 321.1 (M + 1^+^, 8), 277.9 (14), 198.9 (21), 170.9 (19), 121.0 (55), 105.0 (85), 91.0 (25), 79.0 (100), 65.1 (8), 51.0 (7).

*N-(2-Iodophenyl)-1-isopropylcyclohexa-2,5-diene-1-carboxamide* (**S2e-I**). Using the general benzamide synthesis procedure, diene acid **S1c** (0.497 g, 2.99 mmol, 1.0 equiv) in DCM (7.5 mL) reacted with 2-iodoaniline (0.863 g, 3.94 mmol, 1.3 equiv) in DCM (9.9 mL, 0.4 M) to afford the crude product that was purified by column chromatography (silica, 4:1 hexanes/EtOAc) to afford pure **S2e-I** (0.93 g, 2.53 mmol) in 84% yield as a yellow oil: ^1^H NMR (400 MHz, DMSO-*d*_6_) δ 8.22 (s, 1H), 7.90–7.78 (m, 2H), 7.34 (t, *J* = 7.5 Hz, 1H), 6.93–6.84 (m, 1H), 6.09 (dt, *J* = 10.7, 3.4 Hz, 2H), 5.82–5.74 (m, 2H), 2.87–2.67 (m, 2H), 2.33 (m, 1H), 0.91–0.76 (d, 6H); ^13^C{^1^H} NMR (101 MHz, DMSO-*d*_6_) δ_u_ 139.3, 129.3, 128.4, 127.2, 126.7, 124.2, 33.9, 18.0; δ_d_ 172.5, 139.3, 93.9, 53.5, 26.9; GC (method B) *t*_R_ = 3.671 min; EI-MS *m*/*z* (%) 367.1 (M^+^, 14), 324.0 (16), 247.0 (53), 218.9 (32), 197.1 (11), 121.1 (64), 105.1 (65), 91.0 (35), 79.1 (100), 65.1 (11), 51.0 (7).

*N-(2-Iodophenyl)-1-(methoxymethyl)cyclohexa-2,5-diene-1-carboxamide* (**S2f-I**). Using the general benzamide synthesis procedure, diene acid **S1d** (0.500 g, 2.97 mmol, 1.0 equiv) in DCM (7.4 mL, 0.4 M) reacted with 2-iodoaniline (0.781 g, 3.57 mmol, 1.2 equiv) in DCM (8.9 mL, 0.4 M) to afford the crude product that was purified by column chromatography (silica, 7:1 hexanes/EtOAc) to afford pure **S2f-I** (1.06 g, 2.87 mmol) in 97% yield as a yellow oil: **^1^**H NMR (400 MHz, CDCl_3_) δ 8.25 (dd, *J* = 8.2, 1.6 Hz, 1H), 8.21 (s, 1H), 7.67 (dd, *J* = 8.0, 1.5 Hz, 1H), 7.28–7.20 (m, 1H), 6.73 (td, *J* = 7.6, 1.6 Hz, 1H), 6.11–6.01 (m, 2H), 5.89–5.79 (m, 2H), 3.62 (s, 2H), 3.33 (s, 3H), 2.92–2.68 (m, 2H); ^13^C{^1^H} NMR (101 MHz, CDCl_3_) δ_u_ 138.8, 129.2, 128.4, 125.9, 125.7, 121.3, 59.6; δ_d_ 171.1, 138.6, 89.1, 77.0, 51.1, 26.7; GC (method B) *t*_R_ = 3.353 min; EI-MS *m*/*z* (%) 369 (M+, 2), 336.0 (5), 227.9 (4), 262.9 (7), 244.9 (35), 232.0 (26), 218.8 (10), 210.0 (5), 196.0 (8), 150.9 (10), 118.9 (14), 105.0 (26), 92.0 (100), 77.0 (13), 63.0 (6); HRMS (ESI) calcd for C_15_H_17_INO_2_ [M + H]^+^ 370.0304, found 370.0294.

*Ethyl 2-{1-[(2-Iodophenyl)(methyl)carbamoyl]cyclohexa-2,5-dien-1-yl}acetate* (**1g-I**). Using the general benzamide synthesis procedure, diene acid **S1e** (0.502 g, 2.38 mmol, 1.0 equiv) in DCM (6 mL, 0.4 M) reacted with 2-iodo-*N*-methylaniline (0.721 g, 3.09 mmol, 1.3 equiv) in DCM (7.7 mL, 0.4 M) to afford the crude product that was purified by column chromatography (silica, 4:1 hexanes/EtOAc) to afford pure **1g-I** (0.84 g, 1.97 mmol) in 83% yield as a tan solid: mp 63.7–64.8 °C; ^1^H NMR (400 MHz, CDCl_3_) δ 7.74 (d, *J* = 7.8 Hz, 1H), 7.23 (d, *J* = 7.7 Hz, 1H), 7.14 (d, *J* = 7.4 Hz, 1H), 6.89 (td, *J* = 7.6, 1.7 Hz, 1H), 5.78 (d, *J* = 10.3 Hz, 1H), 5.58 (d, *J* = 10.2 Hz, 1H), 5.41 (d, *J* = 10.1 Hz, 1H), 5.10 (d, *J* = 10.3 Hz, 1H), 4.05 (q, *J* = 7.1 Hz, 2H), 3.11 (s, 3H), 2.85 (d, *J* = 16.0 Hz, 1H), 2.52 (d, *J* = 16.1 Hz, 1H), 2.25 (d, *J* = 23.3 Hz, 1H), 1.94 (d, *J* = 22.8 Hz, 1H), 1.17 (t, *J* = 7.2 Hz, 3H); ^13^C{^1^H} NMR (101 MHz, CDCl_3_) δ_u_ 139.5, 130.5, 129.0, 129.0, 128.6, 126.9, 124.6, 123.7, 39.5, 14.3; δ_d_ 171.1, 146.4, 101.2, 60.2, 47.7, 46.6, 26.1; GC (method B) *t*_R_ = 4.145 min; EI-MS *m*/*z* (%) 380.1 (M+, 8), 260.0 (45), 232.9 (14), 210.1 (9), 165.1 (62), 134.0 (81), 119.0 (24), 105.0 (35), 91.1 (100), 77.0 (14); HRMS (ESI) calcd for C_18_H_21_O_3_NI [M + H]^+^ 426.0566, found 426.0580.

*1-Benzyl-N-(2-bromophenyl)cyclohexa-2,5-diene-1-carboxamide* (**S2h**). Using the general benzamide synthesis procedure, diene acid **S1f** (1.01 g, 4.71 mmol, 1.0 equiv) in DCM (11.8 mL, 0.4 M) reacted with 2-bromoaniline (1.05 g, 6.12 mmol, 1.3 equiv) in DCM (15.3 mL, 0.4 M) to afford the crude product that was purified by column chromatography (silica, 9:1 hexanes/EtOAc) to afford pure **S2h** (1.53 g, 4.15 mmol) in 88% yield as a yellow oil that solidified to white-yellow crystals upon cooling: mp 70.2–72.0 °C; ^1^H NMR (400 MHz, CDCl_3_) δ 8.43 (dd, *J* = 8.3, 1.6 Hz, 1H), 8.31 (s, 1H), 7.48 (dd, *J* = 8.0, 1.5 Hz, 1H), 7.31 (ddd, *J* = 8.5, 7.5, 1.5 Hz, 1H), 7.27–7.14 (m, 4H), 6.95 (td, *J* = 7.7, 1.6 Hz, 1H), 5.99 (dt, *J* = 10.3, 3.3 Hz, 2H), 5.82 (dt, *J* = 10.4, 2.0 Hz, 2H), 3.21 (s, 2H), 2.73 (dddd, *J* = 23.4, 5.5, 3.1, 2.3 Hz, 1H), 2.56 (dtt, *J* = 23.4, 3.6, 1.8 Hz, 1H); ^13^C{^1^H} NMR (101 MHz, CDCl_3_) δ_u_ 132.2, 130.7, 128.4, 127.9, 127.8, 127.6, 126.3, 125.0, 121.2; δ_d_ 172.0, 137.4, 136.0, 113.3, 51.3, 43.4, 26.2; GC (method A) *t*_R_ = 19.251 min; EI-MS *m*/*z* (%) 367.1 (M+, 7), 276.0 (15), 196.1 (25), 170.1 (12), 152.1 (4), 120.0 (4), 105.0 (29), 91.1 (100), 77.1 (10), 63.1 (10); HRMS (ESI) calcd for C_20_H_19_ONBr [M + H]^+^ 368.0650, found 368.0657.

*1-Benzyl-N-(2-iodophenyl)cyclohexa-2,5-diene-1-carboxamide* (**S2h-I**). Using the general benzamide synthesis procedure, diene acid **S1f** (0.501 g, 2.33 mmol, 1.0 equiv) in DCM (5.8 mL, 0.4 M) reacted with 2-iodoaniline (0.665 g, 3.03 mmol, 1.3 equiv) in DCM (7.6 mL, 0.4 M) to afford the crude product that was purified by column chromatography (silica, 7:1 hexanes/EtOAc) to afford pure **S2h-I** (0.868 g, 2.09 mmol) in 90% yield as a yellow solid: mp 68.6–70.4 °C; ^1^H NMR (400 MHz, CDCl_3_) δ 8.35 (dd, *J* = 8.2, 1.6 Hz, 1H), 8.11 (s, 1H), 7.75 (dd, *J* = 8.0, 1.5 Hz, 1H), 7.41–7.32 (m, 1H), 7.27–7.16 (m, 5H), 6.84 (td, *J* = 7.6, 1.6 Hz, 1H), 6.03 (dt, *J* = 10.2, 3.4 Hz, 2H), 5.85 (dt, *J* = 10.4, 2.0 Hz, 2H), 3.23 (s, 2H), 2.85–2.73 (m, 1H), 2.66–2.53 (m, 1H); ^13^C{^1^H} NMR (101 MHz, CDCl_3_) δ_u_ 138.8, 130.7, 129.2, 127.9, 127.8, 127.6, 126.3, 125.8, 121.2; δ_d_ 172.2, 138.5, 137.4, 89.3, 51.2, 43.4, 26.3; GC (method B) *t*_R_ = 19.751 min; EI-MS *m*/*z* (%) 415.1 (M+, 7), 324.0 (14), 245.9 (10), 218.9 (8), 196.0 (40), 170.0 (14), 119.0 (11), 105.0 (32), 91.0 (100), 77.1 (11), 65.0 (8); HRMS (ESI) calcd for C_20_H_19_ONI [M + H]^+^ 416.0511, found 416.0508.

*N-(2-Bromo-4-fluorophenyl)-1-isopropylcyclohexa-2,5-diene-1-carboxamide* (**S2i**). Using the general benzamide synthesis procedure, diene acid **S1c** (0.499 g, 3.00 mmol, 1.0 equiv) in DCM (7.5 mL, 0.4 M) reacted with 2-bromo-4-fluoroaniline (0.44 mL, 3.90 mmol, 1.3 equiv) in DCM (9.8 mL, 0.4 M) to afford the crude product that was purified by column chromatography (silica, 20:1 hexanes/EtOAc) to afford pure **S2i** (0.907 g, 2.68 mmol) in 90% yield as a clear colorless oil: ^1^H NMR (400 MHz, CDCl_3_) δ 8.37 (dd, *J* = 9.2, 5.6 Hz, 1H), 8.23 (s, 1H), 7.26 (dd, *J* = 7.9, 2.9 Hz, 1H), 7.03 (ddd, *J* = 9.2, 7.8, 2.9 Hz, 1H), 6.12 (dtd, *J* = 10.7, 3.4, 1.7 Hz, 2H), 5.77 (dt, *J* = 10.5, 2.0 Hz, 2H), 2.84–2.76 (m, 2H), 2.47 (hept, *J* = 6.9 Hz, 1H), 0.90 (d, *J* = 6.9 Hz, 6H); ^13^C{^1^H} NMR (101 MHz, CDCl_3_) δ_u_ 128.5, 126.6, 122.3 (d, ^3^*J*_C–F_ = 8.1 Hz), 119.2 (d, ^2^*J*_C–F_ = 26.3 Hz), 115.1 (d, ^2^*J*_C–F_ = 22.2 Hz), 33.4, 17.7; δ_d_ 172.5, 158.3 (d, ^1^*J*_C–F_ = 248.5 Hz), 132.6, 54.3, 26.8; ^19^F NMR (376 MHz, CDCl_3_) δ −116.66; GC (method B) *t*_R_ = 2.741 min; EI-MS *m*/*z* (%) 337.1 (M – 1^+^, 7), 294.0 (15), 216.9 (18), 188.9 (17), 138.0 (15), 121.1 (80), 105.0 (58), 79.1 (100), 51.0 (6); HRMS (ESI) calcd for C_16_H_18_ONBrF [M + H]^+^ 338.0556, found 338.0560.

*N-(4-Fluoro-2-iodophenyl)-1-isopropylcyclohexa-2,5-diene-1-carboxamide* (**S2i-I**). Using the general benzamide synthesis procedure, diene acid **S1c** (0.501 g, 3.01 mmol, 1.0 equiv) in DCM (7.5 mL, 0.4 M) reacted with 4-fluoro-2-iodoaniline (0.37 mL, 3.16 mmol, 1.05 equiv) in DCM (7.9 mL, 0.4 M) to afford the crude product that was purified by column chromatography (silica, 20:1 hexanes/EtOAc) to afford pure **S2i-I** (1.06 g, 2.74 mmol) in 91% yield as an orange solid: mp 62.0–62.5 °C; ^1^H NMR (400 MHz, CDCl_3_) δ 8.16 (dd, *J* = 9.1, 5.5 Hz, 1H), 7.95 (s, 1H), 7.40 (dd, *J* = 7.7, 2.9 Hz, 1H), 7.00 (ddd, *J* = 9.1, 7.8, 2.9 Hz, 1H), 6.06 (dt, *J* = 10.3, 3.3 Hz, 2H), 5.72 (dt, *J* = 10.5, 2.1 Hz, 2H), 2.84–2.65 (m, 2H), 2.40 (hept, *J* = 6.9 Hz, 1H), 0.83 (d, *J* = 6.9 Hz, 6H); ^13^C{^1^H} NMR (101 MHz, CDCl_3_) δ_u_ 128.6, 126.7, 125.3 (d, ^2^*J*_C–F_ = 24.9 Hz), 122.2 (d, ^3^*J*_C–F_ = 7.8 Hz), 115.9 (d, ^2^*J*_C–F_ = 21.7 Hz), 33.4, 17.7; δ_d_ 172.6, 158.4 (d, ^1^*J*_C–F_ = 248.6 Hz), 135.1, 88.7, 54.2, 26.9; ^19^F NMR (376 MHz, CDCl_3_) δ −116.70; GC (method B) *t*_R_ = 3.152 min; EI-MS *m*/*z* (%) 385.1 (M^+^, 18), 342.0 (21), 264.9 (54), 236.9 (29), 215.0 (10), 138.0 (26), 121.1 (100), 105.0 (65), 85.1 (17), 79.0 (99) 51.0 (7); HRMS (ESI) calcd for C_16_H_18_ONFI [M + H]^+^ 386.0417, found 386.0408.

*N-(2-Bromo-4-chlorophenyl)-1-isopropylcyclohexa-2,5-diene-1-carboxamide* (**S2j**). Using the general benzamide synthesis procedure, diene acid **S1c** (0.502 g, 3.02 mmol, 1.0 equiv) in DCM (7.6 mL, 0.4 M) reacted with 2-bromo-4-chloroaniline (0.804 g, 3.90 mmol, 1.3 equiv) in DCM (9.8 mL, 0.4 M) to afford the crude product that was purified by column chromatography (silica, 20:1 hexanes/EtOAc) to afford pure **S2j** (0.99 g, 2.79 mmol) in 93% yield as a yellow oil: ^1^H NMR (400 MHz, CDCl_3_) δ 8.39 (d, *J* = 8.9 Hz, 1H), 8.32 (s, 1H), 7.50 (d, *J* = 2.4 Hz, 1H), 7.27 (dd, 1H), 6.15–6.09 (m, 2H), 5.77 (dt, *J* = 10.5, 2.0 Hz, 2H), 2.82–2.78 (m, 2H), 2.46 (hept, *J* = 6.9 Hz, 1H), 0.89 (d, *J* = 6.9 Hz, 6H); ^13^C{^1^H} NMR (101 MHz, CDCl_3_) δ_u_ 131.6, 128.6, 128.4, 126.5, 121.8, 116.2, 33.4, 17.7; δ_d_ 172.5, 134.9, 129.0, 113.4, 54.4, 26.8; GC (method B) *t*_R_ = 3.515 min; EI-MS *m*/*z* (%) 355.1 (M + 1^+^, 6), 312.0 (13), 234.9 (16), 206.9 (16), 154.0 (11), 121.1 (90), 105.0 (51), 91.0 (12), 79.1 (100), 51.0 (6).

*N-(4-Chloro-2-iodophenyl)-1-isopropylcyclohexa-2,5-diene-1-carboxamide* (**S2j-I**). Using the general benzamide synthesis procedure, diene acid **S1c** (0.505 g, 3.04 mmol, 1.0 equiv) in DCM (7.6 mL, 0.4 M) reacted with 4-chloro-2-iodoaniline (0.798 g, 3.15 mmol, 1.04 equiv) in DCM (7.9 mL, 0.4 M) to afford the crude product that was purified by column chromatography (silica, 20:1 hexanes/EtOAc) to afford pure **S2j-I** (0.998 g, 2.48 mmol) in 82% yield as a white solid: mp 85.3–86.5 °C; ^1^H NMR (400 MHz, CDCl_3_) δ 8.20 (d, *J* = 8.9 Hz, 1H), 8.04 (s, 1H), 7.66 (d, *J* = 2.4 Hz, 1H), 7.23 (dd, *J* = 8.9, 2.4 Hz, 1H), 6.06 (dt, *J* = 10.3, 3.4 Hz, 2H), 5.71 (dt, *J* = 10.5, 2.0 Hz, 2H), 2.84–2.65 (m, 2H), 2.39 (hept, *J* = 6.9 Hz, 1H), 0.83 (d, *J* = 6.9 Hz, 6H); ^13^C{^1^H} NMR (101 MHz, CDCl_3_) δ_u_ 137.8, 129.2, 128.7, 126.6, 121.6, 33.4, 17.7; δ_d_ 172.7, 137.5, 129.5, 89.0, 54.4, 26.9; GC (method B) *t*_R_ = 4.148 min; EI-MS *m*/*z* (%) 401.1 (M^+^, 13), 358.0 (13), 280.9 (40), 252.9 (23), 231.0 (9), 154.0 (20), 121.1 (100), 105.0 (56), 91.0 (15), 79.0 (90), 63.0 (6), 51.0 (5); HRMS (ESI) calcd for C_16_H_18_ONClI [M + H]^+^ 402.0122, found 402.0119.

*N-(2-Bromo-4-methylphenyl)-1-isopropylcyclohexa-2,5-diene-1-carboxamide* (**S2k**). Using the general benzamide synthesis procedure, diene acid **S1c** (0.504 g, 3.03 mmol, 1.0 equiv) in DCM (7.6 mL, 0.4 M) reacted with 2-bromo-4-methylaniline (0.48 mL, 3.94 mmol, 1.3 equiv) in DCM (9.9 mL, 0.4 M) to afford the crude product that was purified by column chromatography (silica, 10:1 hexanes/EtOAc) to afford pure **S2k** (0.966 g, 2.89 mmol) in 96% yield as a yellow oil: ^1^H NMR (400 MHz, CDCl_3_) δ 8.26 (d, *J* = 8.3 Hz, 1H), 7.32 (dd, *J* = 2.0, 0.9 Hz, 1H), 7.09 (dd, *J* = 8.4, 2.0 Hz, 1H), 6.15–6.05 (m, 2H), 5.78 (dt, *J* = 10.5, 2.0 Hz, 2H), 2.80 (dp, *J* = 5.4, 1.9 Hz, 2H), 2.46 (h, *J* = 7.0 Hz, 1H), 2.28 (s, 3H), 0.90 (d, *J* = 6.9 Hz, 7H); ^13^C{^1^H} NMR (101 MHz, CDCl_3_) δ_u_ 132.4, 128.9, 128.3, 126.7, 121.2, 33.4, 20.5, 17.7; δ_d_ 172.4, 134.9, 133.5, 113.3, 54.3, 26.8; GC (method B) *t*_R_ = 3.296 min; EI-MS *m*/*z* (%) 333.1 (M – 1^+^, 13), 290.0 (19), 212.9 (22), 185.9 (24), 134.1 (60), 122.1 (62), 105.0 (65), 91.0 (15), 79.1 (100), 51.0 (8).

*N-(2-Iodo-5-methylphenyl)-1-isopropylcyclohexa-2,5-diene-1-carboxamide* (**S2l-I**). Using the general benzamide synthesis procedure, diene acid **S1c** (0.501 g, 3.00 mmol, 1.0 equiv) in DCM (7.5 mL, 0.4 M) reacted with 2-iodo-5-methylaniline (0.735 g, 3.15 mmol, 1.05 equiv) in DCM (7.9 mL, 0.4 M) to afford the crude product that was purified by column chromatography (silica, 10:1 hexanes/EtOAc) to afford pure **S2l-I** (1.13 g, 2.96 mmol) in 99% yield as a white solid: mp 78.2–79.1 °C; ^1^H NMR (400 MHz, CDCl_3_) δ 8.11 (d, *J* = 2.1 Hz, 1H), 8.01 (s, 1H), 7.52 (d, *J* = 8.1 Hz, 1H), 6.57 (dd, *J* = 8.1, 1.5 Hz, 1H), 6.05 (dt, *J* = 10.3, 3.3 Hz, 2H), 5.72 (dt, *J* = 10.5, 2.0 Hz, 2H), 2.85–2.64 (m, 2H), 2.40 (hept, *J* = 6.9 Hz, 1H), 2.24 (s, 3H), 0.84 (d, *J* = 6.9 Hz, 6H); ^13^C{^1^H} NMR (101 MHz, CDCl_3_) δ_u_ 138.3, 129.50, 128.5, 126.7, 126.7, 122.0, 33.4, 21.3, 17.8; δ_d_ 172.8, 139.6, 138.3, 54.3, 26.9; GC (method B) *t*_R_ = 3.659 min; EI-MS *m*/*z* (%) 381.1 (M^+^, 26), 338.0 (196), 260.9 (60), 232.9 (50), 211.0 (18), 134.0 (99), 121.0 (81), 105.0 (76), 85 (18), 79.0 (100), 65.0 (5), 51.0 (10); HRMS (ESI) calcd for C_17_H_21_ONI [M + H]^+^ 382.0668, found 382.0658.

*N-(2-Bromopyridin-3-yl)-1-methylcyclohexa-2,5-diene-1-carboxamide* (**S2m**). Using the general benzamide synthesis procedure, diene acid **S1a** (0.501 g, 3.62 mmol, 1.0 equiv) in DCM (9.1 mL, 0.4 M) reacted with 2-bromopyridin-3-amine (0.814 g, 4.71 mmol, 1.3 equiv) in DCM (11.8 mL, 0.4 M) to afford the crude product that was purified by column chromatography (silica, 4:1 hexanes/EtOAc) to afford pure **S2m** (0.899 g, 3.07 mmol) in 85% yield as a white-tan solid: mp 96.0–97.8 °C; ^1^H NMR (400 MHz, CDCl_3_) δ 8.69 (dd, *J* = 8.1, 1.8 Hz, 1H), 8.40 (s, 1H), 8.05 (dd, *J* = 4.6, 1.8 Hz, 1H), 7.25 (dd, *J* = 8.2, 4.7 Hz, 1H), 6.05 (dt, *J* = 10.2, 3.4 Hz, 2H), 5.78 (dt, *J* = 10.3, 2.1 Hz, 2H), 2.96–2.75 (m, 2H), 1.42 (s, 3H); ^13^C{^1^H} NMR (101 MHz, CDCl_3_) δ_u_ 144.2, 129.3, 127.8, 126.5, 123.6, 24.5; δ_d_ 173.4, 133.9, 132.9, 46.5, 26.0; GC (method B) *t*_R_ = 2.454 min; EI-MS *m*/*z* (%) 292.0 (M – 1^+^, 1), 199.0 (8), 171.9 (5), 119.0 (6), 93.0 (100), 77.0 (31), 65.0 (8), 51.0 (4).

*N-(3-Bromopyridin-2-yl)-1-methylcyclohexa-2,5-diene-1-carboxamide* (**S2n**). Using the general benzamide synthesis procedure, diene acid **S1a** (0.501 g, 3.62 mmol, 1.0 equiv) in DCM (9.1 mL, 0.4 M) reacted with 2-amino-3-bromopyridine (0.814 g, 4.71 mmol, 1.3 equiv) in DCM (11.8 mL, 0.4 M) to afford the crude product that was purified by column chromatography (silica, 4:1 hexanes/EtOAc) to afford pure **S2n** (0.858 g, 2.93 mmol) in 81% yield as a white solid: mp 96.0–97.8 °C; ^1^H NMR (400 MHz, CDCl_3_) δ 8.47 (s, 1H), 8.43 (dd, *J* = 4.8, 1.6 Hz, 1H), 7.83 (dd, *J* = 7.9, 1.6 Hz, 1H), 6.94 (dd, *J* = 7.9, 4.7 Hz, 1H), 6.06–5.96 (m, 2H), 5.82 (dt, *J* = 10.4, 2.0 Hz, 2H), 2.94–2.75 (m, 2H), 1.43 (s, 3H); ^13^C{^1^H} NMR (101 MHz, CDCl_3_) δ_u_ 147.6, 141.1, 129.7, 126.1, 120.8, 24.6; δ_d_ 171.7, 148.6, 111.0, 46.6, 26.0; GC (method B) *t*_R_ = 2.695 min; EI-MS *m*/*z* (%) 293.0 (M^+^, 12), 200.9 (42), 171.9 (83), 157.9 (16), 119.0 (6), 93.1 (100), 77.0 (54), 65.0 (14), 51.0 (7); HRMS (ESI) calcd for C_13_H_14_ON_2_Br [M + H]^+^ 293.0289, found 293.0287.

*N-(3-Bromopyridin-2-yl)-1-ethylcyclohexa-2,5-diene-1-carboxamide* (**S2o**). Using the general benzamide synthesis procedure, diene acid **S1b** (1.11 g, 6.50 mmol, 1.0 equiv) in DCM (16.3 mL, 0.4 M) reacted with 2-amino-3-bromopyridine (1.71 g, 9.88 mmol, 1.5 equiv) in DCM (24.7 mL, 0.4 M) to afford the crude product that was purified by column chromatography (silica, 3:2 hexanes/EtOAc) to afford pure **S2o** (1.575 g, 5.13 mmol) in 78% yield as a white solid: mp 81.3–84.5 °C; ^1^H NMR (400 MHz, CDCl_3_) δ 8.48 (s, 1H), 8.43 (dd, *J* = 4.7, 1.6 Hz, 1H), 7.83 (dd, *J* = 7.9, 1.6 Hz, 1H), 6.94 (dd, *J* = 7.9, 4.7 Hz, 1H), 6.11 (dt, *J* = 10.3, 3.4 Hz, 2H), 5.74 (dt, *J* = 10.4, 2.1 Hz, 2H), 2.87–2.79 (m, 2H), 1.91 (q, *J* = 7.5 Hz, 2H), 0.86 (t, *J* = 7.5 Hz, 3H); ^13^C{^1^H} NMR (101 MHz, CDCl_3_) δ_u_ 147.6, 141.1, 128.0, 127.8, 120.8, 8.8; δ_d_ 171.6, 148.6, 111.1, 51.1, 29.3, 26.3; GC (method B) *t*_R_ = 3.072 min; EI-MS *m*/*z* (%) 307.0 (M^+^, 5), 200.9 (18), 171.9 (49), 157.9 (8), 108.1 (40), 91.1 (30), 79.1 (100), 65.1 (7), 51.1 (5); HRMS (ESI) calcd for C_14_H_16_ON_2_Br [M + H]^+^ 307.0446, found 307.0447.

*N-(3-Iodopyridin-2-yl)-1-ethylcyclohexa-2,5-diene-1-carboxamide* (**S2o-I**). Using the general benzamide synthesis procedure, diene acid **S1b** (0.561 g, 3.29 mmol, 1.0 equiv) in DCM (8.2 mL, 0.4 M) reacted with 2-amino-3-iodopyridine (0.759 g, 3.45 mmol, 1.05 equiv) in DCM (8.6 mL, 0.4 M) to afford the crude product that was purified by column chromatography (silica, 3:2 hexanes/EtOAc) to afford pure **S2o-I** (1.04 g, 2.94 mmol) in 89% yield as a white solid: mp 101.3–101.9 °C; ^1^H NMR (400 MHz, CDCl_3_) δ 8.38 (dd, *J* = 4.7, 1.7 Hz, 1H), 8.26 (s, 1H), 7.99 (dd, *J* = 7.9, 1.6 Hz, 1H), 6.72 (dd, *J* = 7.9, 4.7 Hz, 1H), 6.09–5.99 (m, 2H), 5.72–5.63 (m, 2H), 2.88–2.67 (m, 2H), 1.84 (q, *J* = 7.5 Hz, 2H), 0.79 (t, *J* = 7.5 Hz, 3H); ^13^C{^1^H} NMR (101 MHz, CDCl_3_) δ_u_ 148.4, 147.8, 128.1, 127.8, 121.3, 8.8; δ_d_ 171.8, 150.9, 86.0, 51.0, 29.3, 26.4; GC (method B) *t*_R_ = 3.444 min; EI-MS *m*/*z* (%) 353.1 (M – 1^+^, 9), 246.9 (39), 219.9 (100), 203.9 (12), 108.0 (33), 93.0 (21), 79.0 (60), 65.0 (5), 51.0 (4); HRMS (ESI) calcd for C_14_H_16_ON_2_I [M + H]^+^ 355.0307, found 355.0299.

#### General Procedure for the Methoxymethyl (MOM) Group Protection of Amide N

A secondary amide (1.0 mmol, 1.0 equiv) was dissolved in THF (7 mL, 0.14 M) and cooled to 0 °C. LiHMDS (1.0 M in hexanes, 1.2 mmol, 1.2 equiv) was added dropwise, and the solution stirred for 10 min. Chloro- or bromomethyl methyl ether (2.0–5.0 mmol, 2.0–5.0 equiv) was added dropwise to the reaction solution, and the mixture was left stirring while slowly warming to rt. Upon completion, the reaction was quenched with saturated NH_4_Cl, and the mixture diluted with EtOAc. The aqueous layer was extracted twice with EtOAc. The combined organic layers were washed with brine, dried with MgSO_4_, filtered, and concentrated. The crude product was purified by column chromatography.

*N-(2-Bromophenyl)-N-(methoxymethyl)-1-methylcyclohexa-2,5-diene-1-carboxamide* (**1b**). Using the general procedure for MOM group protection, secondary amide **S2b** (0.444 g, 1.52 mmol, 1.0 equiv) in THF (10.6 mL, 0.14 M) was alkylated with chloromethyl methyl ether (0.248 mL, 3.04 mmol, 2.0 equiv). The crude product was purified by column chromatography (silica, 9:1 hexanes/EtOAc) to afford **1b** (0.389 g, 1.16 mmol) in 76% yield as a clear colorless oil: ^1^H NMR (400 MHz, DMSO-*d*_6_) δ 7.62 (d, *J* = 7.6 Hz, 1H), 7.34–7.21 (m, 3H), 5.58 (s, 2H), 5.46 (s, 1H), 5.25 (s, 1H), 4.25 (s, 1H), 3.25 (s, 3H), 2.45–2.05 (m, 2H), 1.22 (s, 3H); ^13^C{^1^H} NMR (101 MHz, DMSO-*d*_6_) δ_u_ 133.2, 130.5, 130.1, 128.3, 124.4, 122.7, 56.4, 29.4; δ_d_ 174.1, 140.6, 121.4, 80.2, 46.0, 25.9; GC (method B) *t*_R_ = 2.578 min; EI-MS *m*/*z* (%) 335.1 (M + 1^+^, 1), 305.1 (5), 244.0 (4), 224.1 (19), 211.9 (8), 198.9 (8), 184.9 (70), 164.0 (58), 152.0 (5), 134.0 (6), 105.0 (7), 93.0 (100), 77.0 (39), 65.0 (7), 51.0 (5); HRMS (ESI) calcd for C_16_H_19_O_2_NBr [M + H]^+^ 336.0599, found 336.0590.

*N-(2-Iodophenyl)-N-(methoxymethyl)-1-methylcyclohexa-2,5-diene-1-carboxamide* (**1b-I**). Using the general procedure for MOM group protection, secondary amide **S2b-I** (0.998 g, 2.95 mmol, 1.0 equiv) in THF (20.7 mL, 0.14 M) was alkylated with chloromethyl methyl ether (0.90 mL, 11.8 mmol, 4.0 equiv). The crude product was purified by column chromatography (silica, 9:1 hexanes/EtOAc) to afford **1b-I** (0.990 g, 2.58 mmol) in 88% yield as a yellow oil: ^1^H NMR (400 MHz, DMSO-*d*_6_) δ 7.90–7.86 (m, 1H), 7.36 (td, *J* = 7.6, 1.6 Hz, 1H), 7.28 (dd, *J* = 7.8, 1.8 Hz, 1H), 7.09 (td, *J* = 7.5, 1.8 Hz, 1H), 5.72–5.55 (m, 3H), 5.50 (d, *J* = 9.9 Hz, 1H), 5.27 (s, 1H), 4.22 (d, *J* = 10.1 Hz, 1H), 3.26 (s, 3H), 2.48–2.14 (m, 2H), 1.26 (s, 3H); ^13^C{^1^H} NMR (101 MHz, DMSO-*d*_6_) δ_u_ 139. 9, 139.5, 132.4, 130.8, 129.9, 129.0, 124.2, 122.5, 56.5, 29.5, 26.8; δ_d_ 174.1, 150.7, 144.0, 80.6, 46.1, 25.9; GC (method B) *t*_R_ = 2.935 min; EI-MS *m*/*z* (%) 383.1 (M^+^, 1), 292.0 (4), 259.9 (6), 244.9 (11), 230.9 (100), 224.1 (18), 202.9 (5), 164.0 (28), 134.0 (4), 93.0 (50), 77.0 (24), 65.0 (5), 51.0 (5); HRMS (ESI) calcd for C_16_H_19_O_2_NI [M + H]^+^ 384.0461, found 384.0447.

*1-Ethyl-N-(2-iodophenyl)-N-(methoxymethyl)cyclohexa-2,5-diene-1-carboxamide* (**1c-I**). Using the general procedure for MOM group protection, secondary amide **S2c-I** (1.10 g, 3.12 mmol, 1.0 equiv) in THF (21.8 mL, 0.14 M) was alkylated with chloromethyl methyl ether (0.47 mL, 6.24 mmol, 2.0 equiv). The crude product was purified by column chromatography (silica, 9:1 hexanes/EtOAc) to afford **1c-I** (0.978 g, 2.46 mmol) in 79% yield as an orange solid: mp 64.9–66.0 °C; ^1^H NMR (400 MHz, DMSO-*d*_6_) δ 7.84 (dd, *J* = 7.8, 2.3 Hz, 1H), 7.30 (t, *J* = 7.6 Hz, 1H), 7.21 (d, *J* = 7.7 Hz, 1H), 7.09–7.00 (m, 1H), 5.66 (d, *J* = 10.0 Hz, 1H), 5.52–5.39 (m, 3H), 5.27 (s, 1H), 4.17 (d, *J* = 9.8 Hz, 1H), 3.22 (s, 3H), 2.37 (d, *J* = 23.2 Hz, 1H), 2.18 (d, *J* = 23.3 Hz, 1H), 1.69 (q, *J* = 6.9 Hz, 2H), 0.70 (q, *J* = 0.7 Hz, 3H); ^13^C{^1^H} NMR (101 MHz, DMSO-*d*_6_) δ_u_ 139.5, 132.4, 129.8, 129.2, 128.9, 127.2, 125.6, 123.9, 56.6, 8.7; δ_d_ 174.0, 144.1, 117.5, 80.5, 50.4, 33.2, 26.3; GC (method B) *t*_R_ = 3.233 min; EI-MS *m*/*z* (%) 397.1 (M^+^, 1), 365.0 (5), 292.0 (7), 259.9 (13), 239.0 (16), 230.9 (100), 202.9 (4), 164.0 (25), 134.0 (4), 107.0 (26), 90.0 (17), 79.0 (43); HRMS (ESI) calcd for C_17_H_21_O_2_NI [M + H]^+^ 398.0617, found 398.0611.

*N-(2-Bromophenyl)-1-isopropyl-N-(methoxymethyl)cyclohexa-2,5-diene-1-carboxamide* (**1e**). Using the general procedure for MOM group protection, secondary amide **S2e** (1.10 g, 3.43 mmol, 1.0 equiv) in THF (24 mL, 0.14 M) was alkylated with chloromethyl methyl ether (0.51 mL, 6.86 mmol, 2.0 equiv). The crude product was purified by column chromatography (silica, 9:1 hexanes/EtOAc) to afford **1e** (1.01 g, 2.77 mmol) in 81% yield as an orange solid: mp 70.2–71.5 °C; ^1^H NMR (400 MHz, DMSO-*d*_6_) δ 7.61 (dd, *J* = 7.6, 1.8 Hz, 1H), 7.33–7.19 (m, 3H), 5.62 (s, 1H), 5.46 (s, 1H), 5.40–5.20 (m, 3H), 4.27 (s, 1H), 3.28 (s, 3H), 2.44–2.34 (m, 1H), 2.33–2.12 (m, 2H), 0.75 (s, 6H); ^13^C{^1^H} NMR (101 MHz, DMSO-*d*_6_) δ_u_ 133.2, 133.1, 130.0, 128.3, 124.5, 56.8, 35.09, 17.8; δ_d_ 174.4, 141.1, 124.5, 80.6, 54.4, 26.7; GC (method B) *t*_R_ = 3.046 min; EI-MS *m*/*z* (%) 363.1 (M – 1^+^, 1), 331.1 (7), 252.1 (10), 213.9 (15), 183.0 (68), 164.1 (64), 121.1 (21), 105.1 (100), 91.0 (15), 79.1 (47), 51.1 (6); HRMS (ESI) calcd for C_18_H_23_O_2_NBr [M + H]^+^ 364.0912, found 364.0907.

*N-(2-Iodophenyl)-1-isopropyl-N-(methoxymethyl)cyclohexa-2,5-diene-1-carboxamide* (**1e-I**). Using the general procedure for MOM group protection, secondary amide **S2e-I** (0.359 g, 0.978 mmol, 1.0 equiv) in THF (6.8 mL, 0.14 M) was alkylated with bromomethyl methyl ether (0.16 mL, 1.96 mmol, 2.0 equiv). The crude product was purified by column chromatography (silica, 9:1 hexanes/EtOAc) to afford **1e-I** (0.289 g, 0.703 mmol) in 72% yield as a light orange solid: mp 76.9–79.8 °C; ^1^H NMR (400 MHz, DMSO-*d*_6_) δ 7.90 (d, *J* = 7.9 Hz, 1H), 7.35 (t, *J* = 7.6 Hz, 1H), 7.24–7.18 (m, 1H), 7.11 (td, *J* = 7.7, 1.7 Hz, 1H), 5.70 (d, *J* = 10.2 Hz, 1H), 5.56 (d, *J* = 9.8 Hz, 1H), 5.41 (m, 2H), 5.27 (d, *J* = 10.3 Hz, 1H), 4.23 (d, *J* = 9.9 Hz, 1H), 3.31 (s, 3H), 2.43 (hept, *J* = 6.7 Hz, 1H), 2.38–2.18 (m, 2H), 0.88 (d, *J* = 6.6 Hz, 3H), 0.73 (d, *J* = 6.6 Hz, 3H); ^13^C{^1^H} NMR (101 MHz, DMSO-*d*_6_) δ_u_ 139.5, 132.3, 129.9, 129.6, 129.1, 128.9, 125.9, 124.7, 124.6, 56.8, 35.8, 26.7, 18.3, 17.6; δ_d_ 174.3, 144.1, 102.7, 80.6, 54.3, 26.7; GC (method B) *t*_R_ = 3.451 min; EI-MS *m*/*z* (%) 411.1 (M^+^, 1), 379.1 (4), 292.0 (10), 260.1 (10), 252.1 (12), 230.9 (100), 202.9 (5), 164.0 (34), 121.1 (15), 105.0 (55), 90.0 (10), 79.0 (26), 51.0 (4); HRMS (ESI) calcd for C_18_H_23_O_2_NI [M + H]^+^ 412.0773, found 412.0767.

*N-(2-Iodophenyl)-N,1-bis(methoxymethyl)cyclohexa-2,5-diene-1-carboxamide* (**1f-I**). Using the general procedure for MOM group protection, secondary amide **S2f-I** (0.503 g, 1.36 mmol, 1.0 equiv) in THF (9.5 mL, 0.14 M) was alkylated with chloromethyl methyl ether (0.41 mL, 5.45 mmol, 4.0 equiv). The crude product was purified by column chromatography (silica, 7:1 hexanes/EtOAc) to afford **1f-I** (0.506 g, 1.22 mmol) in 90% yield as a dark yellow oil: ^1^H NMR (400 MHz, DMSO-*d*_6_) δ 7.88 (dt, *J* = 8.2, 2.0 Hz, 1H), 7.37–7.31 (m, 1H), 7.27–7.20 (m, 1H), 7.12–7.05 (m, 1H), 5.66–5.57 (m, 2H), 5.56–5.42 (m, 2H), 5.32 (s, 1H), 4.21 (d, *J* = 9.9 Hz, 1H), 3.60 (d, *J* = 8.7 Hz, 1H), 3.35 (d, *J* = 8.8 Hz, 1H), 3.28 (s, 3H), 3.24 (s, 3H), 2.49–2.19 (m, 2H); ^13^C{^1^H} NMR (101 MHz, DMSO-*d*_6_) δ_u_ 139.5, 132.6, 129.9, 128.9, 126.9, 126.5, 126.3, 124.3, 59.3, 56.6; δ_d_ 172.6, 143.6, 102.6, 80.3, 80.0, 51.3, 26.4; GC (method B) *t*_R_ 3.399 min; EI-MS *m*/*z* (%) 413.1 (M^+^, 1), 337.0 (7), 289.9 (9), 259.9 (6), 245.1 (9), 231.9 (100), 202.9 (4), 180.0 (4), 164.0 (9), 121.0 (6), 105.0 (44), 90.0 (34), 77.0 (16), 65.0 (4), 51.0 (4); HRMS (ESI) calcd for C_17_H_21_O_3_NI [M + H]^+^ 414.0566, found 414.0557.

*1-Benzyl-N-(2-bromophenyl)-N-(methoxymethyl)cyclohexa-2,5-diene-1-carboxamide* (**1h**). Using the general procedure for MOM group protection, secondary amide **S2h** (0.996 g, 2.71 mmol, 1.0 equiv) in THF (19 mL, 0.14 M) was alkylated with bromomethyl methyl ether (0.44 mL, 5.41 mmol, 2.0 equiv). The crude product was purified by column chromatography (silica, 9:1 hexanes/EtOAc) to afford **1h** (1.03 g, 2.49 mmol) in 92% yield as a yellow oil: ^1^H NMR (400 MHz, CDCl_3_) δ 7.48 (s, 1H), 7.23–7.16 (m, 1H), 7.17–7.11 (m, 3H), 7.11–7.05 (m, 4H), 5.69 (d, *J* = 10.2 Hz, 1H), 5.63 (s, 1H), 5.52 (s, 1H), 5.41 (s, 1H), 5.01 (s, 1H), 4.28 (d, *J* = 10.0 Hz, 1H), 3.43 (s, 3H), 3.19 (d, *J* = 13.1 Hz, 1H), 3.07 (d, *J* = 13.2 Hz, 1H), 1.93 (s, 2H); ^13^C{^1^H} NMR (101 MHz, CDCl_3_) δ_u_ 132.8, 131.4, 129.3, 129.1, 127.3, 126.3, 125.9, 125.5, 123.4, 57.1; δ_d_ 174.7, 140.3, 137.4, 124.0, 80.2, 51.4, 46.6, 26.0; GC (method A) *t*_R_ = 18.724 min; EI-MS *m*/*z* (%) 413.2 (M + 1^+^, 1), 379.1 (4), 288.0 (9), 240.1 (26), 213.9 (8), 184.9 (30), 168.0 (32), 152.0 (6), 105.0 (99), 91.0 (100), 79.0 (16), 65.0 (7); HRMS (ESI) calcd for C_22_H_23_O_2_NBr [M + H]^+^ 412.0774, found 412.0767.

*1-Benzyl-N-(2-iodophenyl)-N-(methoxymethyl)cyclohexa-2,5-diene-1-carboxamide* (**1h-I**). Using the general procedure for MOM group protection, secondary amide **S2h-I** (0. 501 g, 1.204 mmol, 1.0 equiv) in THF (8.4 mL, 0.14 M) was alkylated with chloromethyl methyl ether (0.37 mL, 4.82 mmol, 4.0 equiv). The crude product was purified by column chromatography (silica, 9:1 hexanes/EtOAc) to afford **1h-I** (0.470 g, 1.02 mmol) in 85% yield as a yellow oil: ^1^H NMR (400 MHz, DMSO-*d*_6_) δ 7.80 (d, *J* = 8.2 Hz, 1H), 7.24 (t, *J* = 7.6 Hz, 1H), 7.19–6.97 (m, 7H), 5.59–5.40 (m, 4H), 5.13 (s, 1H), 4.19 (d, *J* = 9.9 Hz, 1H), 3.24 (s, 3H), 3.02–2.90 (m, 2H), 2.10–1.93 (m, 2H); ^13^C{^1^H} NMR (101 MHz, DMSO-*d*_6_) δ_u_ 139.5, 132.4, 131.5, 129.9, 129.2, 128.8, 127.7, 127.6, 126.3, 125.5, 123.8, 56.7; δ_d_ 173.8, 143.9, 137.6, 115.1, 80.7, 60.1, 51.3, 46.8, 26.1; GC (method A) *t*_R_ = 19.957 min; EI-MS *m*/*z* (%) 459 (M^+^, 1), 427.1 (6), 336.0 (15), 300.0 (6), 292.0 (10), 259.9 (10), 259.9 (20), 240.1 (42), 230.9 (70), 213.1 (4), 196.0 (5), 168.0 (31), 150.9 (7), 105.0 (98), 91.0 (100), 79.0 (18), 65.0 (8); HRMS (ESI) calcd for C_22_H_23_O_2_NI [M + H]^+^ 459.0695, found 459.0696.

*N-(2-Bromo-4-fluorophenyl)-1-isopropyl-N-(methoxymethyl)cyclohexa-2,5-diene-1-carboxamide* (**1i**). Using the general procedure for MOM group protection, secondary amide **S2i** (0.8434 g, 2.49 mmol, 1.0 equiv) in THF (17.4 mL, 0.14 M) was alkylated with chloromethyl methyl ether (0.38 mL, 4.98 mmol, 2.0 equiv). The crude product was purified by column chromatography (silica, 9:1 hexanes/EtOAc) to afford **1i** (0.828 g, 2.17 mmol) in 87% yield as a white solid: mp 66.8–69.9 °C; ^1^H NMR (400 MHz, DMSO-*d*_6_) δ 7.58 (dd, *J* = 8.3, 2.9 Hz, 1H), 7.26 (dd, *J* = 8.9, 5.7 Hz, 1H), 7.19 (td, *J* = 8.4, 2.9 Hz, 1H), 5.69 (s, 1H), 5.49 (d, *J* = 9.7 Hz, 1H), 5.43–5.35 (m, 3H), 4.26 (d, *J* = 9.9 Hz, 1H), 3.30 (s, 3H), 2.47–2.35 (m, 2H), 2.24 (d, *J* = 23.5 Hz, 1H), 0.82 (d, *J* = 7.1 Hz, 3H), 0.72 (d, *J* = 6.9 Hz, 3H); ^13^C{^1^H} NMR (101 MHz, DMSO-*d*_6_) δ_u_ 134.0 (d, ^3^*J*_C–F_ = 9.4 Hz), 128.8, 126.0, 125.0, 124.6, 120.2 (d, ^2^*J*_C–F_ = 25.7 Hz), 115.3 (d, ^2^*J*_C–F_ = 22.1 Hz), 56.8, 35.8, 18.1, 17.5; δ_d_ 174.4, 161.7 (d, ^1^*J*_C–F_ = 250 Hz), 137.6, 125.1, 80.3, 54.3, 26.7; ^19^F NMR (376 MHz, DMSO-*d*_6_) δ −111.69; GC (method B) *t*_R_ = 2.815 min; EI-MS *m*/*z* (%) 381.1 (M + 1^+^, 1), 349.1 (5), 262.0 (6), 246.0 (4), 232.0 (10), 201.0 (57), 182.1 (37), 121.1 (26), 105.1 (100), 90.0 (12), 105.1 (100), 51.1 (4); HRMS (ESI) calcd for C_18_H_22_O_2_NBrF [M + H]^+^ 382.0818, found 382.0813.

*N-(4-Fluoro-2-iodophenyl)-1-isopropyl-N-(methoxymethyl)cyclohexa-2,5-diene-1-carboxamide* (**1i-I**). Using the general procedure for MOM group protection, secondary amide **S2i-I** (0.201 g, 0.519 mmol, 1.0 equiv) in THF (3.6 mL, 0.14 M) was alkylated with chloromethyl methyl ether (0.16 mL, 2.08 mmol, 4.0 equiv). The crude product was purified by column chromatography (silica, 9:1 hexanes/EtOAc) to afford **1i-I** (0.214 g, 0.499 mmol) in 96% yield as a solid: mp 65.1–66.6 °C; ^1^H NMR (400 MHz, DMSO-*d*_6_) δ 7.73 (dd, *J* = 8.2, 1.9 Hz, 1H), 7.22–7.15 (m, 2H), 5.71 (d, *J* = 9.7 Hz, 1H), 5.53 (d, *J* = 9.6 Hz, 1H), 5.38 (dd, *J* = 19.5, 10.4 Hz, 2H), 4.19 (d, *J* = 9.6 Hz, 1H), 3.29 (s, 3H), 2.46–2.40 (m, 1H), 2.39–2.19 (m, 2H), 0.86 (d, *J* = 6.7 Hz, 3H), 0.72 (d, *J* = 6.9 Hz, 3H); ^13^C{^1^H} NMR (101 MHz, DMSO-*d*_6_) δ_u_ 133.0 (d, ^3^*J*_C–F_ = 9.0 Hz), 129.0, 125.9 (d, ^2^*J*_C–F_ = 24.9 Hz), 124.8, 124.7, 115.8 (d, ^2^*J*_C–F_ = 22.3 Hz), 56.8, 35.8, 18.3, 17.5; δ_d_ 174.3, 161.3 (d, ^1^*J*_C–F_ = 251 Hz), 140.8, 100.0, 80.5, 54.3, 26.7; ^19^F NMR (376 MHz, DMSO-*d*_6_) δ −113.19; GC (method B) *t*_R_ = 3.193 min; EI-MS *m*/*z* (%) 429.1 (M^+^, 1), 397.1 (7), 310.0 (9), 278.0 (12), 265.0 (7), 262.0 (4), 248.9 (100), 182.0 (27), 121.1 (18), 105.0 (55), 79.0 (28); HRMS (ESI) calcd for C_18_H_22_O_2_NFI [M + H]^+^ 430.0679, found 430.0675.

*N-(2-Bromo-4-chlorophenyl)-1-isopropyl-N-(methoxymethyl)cyclohexa-2,5-diene-1-carboxamide* (**1j**). Using the general procedure for MOM group protection, secondary amide **S2j** (0.969 g, 2.73 mmol, 1.0 equiv) in THF (19.1 mL, 0.14 M) was alkylated with chloromethyl methyl ether (0.42 mL, 5.50 mmol, 2.0 equiv). The crude product was purified by column chromatography (silica, 9:1 hexanes/EtOAc) to afford **1j** (0.855 g, 2.14 mmol) in 79% yield as a low-melting point orange solid: ^1^H NMR (400 MHz, CDCl_3_) δ 7.57 (t, *J* = 1.3 Hz, 1H), 7.16 (d, *J* = 1.3 Hz, 2H), 5.70–5.62 (m, 2H), 5.49 (d, *J* = 10.2 Hz, 1H), 5.38 (d, *J* = 10.4 Hz, 1H), 5.25 (s, 1H), 4.24 (d, *J* = 10.0 Hz, 1H), 3.40 (s, 3H), 2.51 (hept, *J* = 6.8 Hz, 1H), 2.40 (d, *J* = 23.4 Hz, 1H), 2.16 (d, *J* = 23.6 Hz, 1H), 0.87 (d, *J* = 6.8 Hz, 3H), 0.71 (d, *J* = 6.9 Hz, 3H); ^13^C{^1^H} NMR (101 MHz, CDCl_3_) δ_u_ 133.1, 132.4, 129.6, 127.7, 125.7, 124.9, 79.9, 57.1, 35.7, 17.8, 17.1; δ_d_ 175.2, 139.5, 134.4, 124.9, 79.9, 54.4, 26.7; GC (method B) *t*_R_ = 3.480 min; EI-MS *m*/*z* (%) 399.1 (M + 1^+^, 1), 367.1 (4), 280.0 (5), 263.9 (4), 247.9 (8), 218.9 (65), 198.0 (42), 121.1 (28), 105.1 (100), 90.0 (12), 79.1 (50), 51.1 (4); HRMS (ESI) calcd for C_18_H_22_O_2_NBrCl [M + H]^+^ 398.0522, found 398.0515.

*N-(4-Chloro-2-iodophenyl)-1-isopropyl-N-(methoxymethyl)cyclohexa-2,5-diene-1-carboxamide* (**1j-I**). Using the general procedure for MOM group protection, secondary amide **S2j-I** (0.500 g, 1.25 mmol, 1.0 equiv) in THF (8.8 mL, 0.14 M) was alkylated with chloromethyl methyl ether (0.38 mL, 4.98 mmol, 4.0 equiv). The crude product was purified by column chromatography (silica, 10:1 hexanes/EtOAc) to afford **1j-I** (0.483 g, 1.08 mmol) in 87% yield as a low-melting point tan solid: ^1^H NMR (400 MHz, DMSO-*d*_6_) δ 7.92 (d, *J* = 2.4 Hz, 1H), 7.40 (dd, *J* = 8.4, 2.4 Hz, 1H), 7.17 (dd, *J* = 8.4, 2.0 Hz, 1H), 5.72–5.65 (m, 1H), 5.51 (d, *J* = 9.8 Hz, 1H), 5.45–5.30 (m, 3H), 4.23 (d, *J* = 9.8 Hz, 1H), 3.29 (s, 3H), 2.49–2.36 (m, 2H), 2.27 (d, *J* = 23.4 Hz, 1H), 0.86 (d, *J* = 6.9 Hz, 3H), 0.74 (d, *J* = 7.1 Hz, 2H); ^13^C{^1^H} NMR (101 MHz, DMSO-*d*_6_) δ_u_ 138.3, 133.0, 129.0, 128.9, 126.2, 125.2, 124.8, 56.9, 35.8, 18.3, 17.6; δ_d_ 174.2, 143.3, 133.4, 103.7, 80.4, 54.3, 26.8; GC (method B) *t*_R_ = 4.011 min; EI-MS *m*/*z* (%) 445.0 (M^+^, 1), 413.1 (5), 326.0 (6), 293.9 (8), 290.1 (7), 264.9 (100), 198.0 (34), 121.1 (25), 105.1 (80), 90.0 (10), 79.1 (45), 51.0 (4); HRMS (ESI) calcd for C_18_H_22_O_2_NClI [M + H]^+^ 446.0384, found 446.0373.

*N-(2-Bromo-4-methylphenyl)-1-isopropyl-N-(methoxymethyl)cyclohexa-2,5-diene-1-carboxamide* (**1k**). Using the general procedure for MOM group protection, secondary amide **S2k** (0.99 g, 2.96 mmol, 1.0 equiv) in THF (20.7 mL, 0.14 M) was alkylated with chloromethyl methyl ether (0.45 mL, 5.92 mmol, 2.0 equiv). The crude product was purified by column chromatography (silica, 9:1 hexanes/EtOAc) to afford **1k** (0.857 g, 2.2 mmol) in 77% yield as a tan solid: mp 66.2–68.8 °C; ^1^H NMR (400 MHz, DMSO-*d*_6_) δ 7.48 (s, 1H), 7.11 (s, 2H), 5.67 (d, *J* = 19.2 Hz, 1H), 5.49 (d, *J* = 9.9 Hz, 1H), 5.37 (d, *J* = 10.5 Hz, 2H), 5.30 (s, 1H), 4.24 (d, *J* = 9.8 Hz, 1H), 3.29 (s, 3H), 2.45–2.36 (m, 1H), 2.36–2.32 (m, 4H), 2.23 (d, *J* = 23.0 Hz, 1H), 0.82 (d, *J* = 7.0 Hz, 3H), 0.72 (d, *J* = 7.0 Hz, 3H); ^13^C{^1^H} NMR (101 MHz, DMSO-*d*_6_) δ_u_ 133.3, 132.6, 130.3, 128.8, 128.0, 126.4, 125.0, 56.8, 35.8, 20.6, 17.9; δ_d_ 174.5, 140.2, 138.4, 124.1, 80.6, 54.4, 26.7; GC (method B) *t*_R_ = 3.480 min; EI-MS *m*/*z* (%) 399.1 (M + 1^+^, 1), 367.1 (4), 280.0 (5), 263.9 (4), 247.9 (8), 218.9 (65), 198.0 (42), 121.1 (28), 105.1 (100), 90.0 (12), 79.1 (50), 51.1 (4); HRMS (ESI) calcd for C_19_H_25_O_2_NBr [M + H]^+^ 378.1069, found 378.1063.

*N-(2-Iodo-5-methylphenyl)-1-isopropyl-N-(methoxymethyl)cyclohexa-2,5-diene-1-carboxamide* (**1l-I**). Using the general procedure for MOM group protection, secondary amide **S2l-I** (0.883 g, 2.32 mmol, 1.0 equiv) in THF (16.2 mL, 0.14 M) was alkylated with chloromethyl methyl ether (0.53 mL, 6.95 mmol, 3.0 equiv). The crude product was purified by column chromatography (silica, 9:1 hexanes/EtOAc) to afford **1l-I** (0.832 g, 1.95 mmol) in 84% yield as a yellow oil that solidified to yellow crystals upon cooling: mp 71.6–72.3 °C; ^1^H NMR (400 MHz, DMSO-*d*_6_) δ 7.73 (d, *J* = 8.0 Hz, 1H), 7.00 (d, *J* = 2.2 Hz, 1H), 6.92 (dd, *J* = 8.1, 2.3 Hz, 1H), 5.72 (d, *J* = 10.1 Hz, 1H), 5.49 (d, *J* = 9.6 Hz, 1H), 5.47–5.36 (m, 2H), 5.25 (d, *J* = 10.1 Hz, 1H), 4.24 (d, *J* = 9.8 Hz, 1H), 3.30 (s, 3H), 2.47–2.39 (m, 1H), 2.37 (s, 1H), 2.24 (s, 3H), 2.16 (d, *J* = 23.5 Hz, 1H), 0.87 (d, *J* = 6.7 Hz, 3H), 0.73 (d, *J* = 6.7 Hz, 3H); ^13^C{^1^H} NMR (101 MHz, DMSO-*d*_6_) δ_u_ 139.1, 133.1, 130.8, 129.5, 125.7, 124.2, 124.1, 56.9, 35.8, 20.5, 18.3, 17.5; δ_d_ 174.4, 143.8, 138.6, 98.6, 80.5, 54.3, 26.7; GC (method B) *t*_R_ = 3.610 min; EI-MS *m*/*z* (%) 425.1 (M^+^, 1), 266.1 (22), 259.1 (7), 245.0 (100), 178.0 (55), 148.0 (6), 121.1 (11), 105.0 (40), 90.0 (12), 79.0 (22); HRMS (ESI) calcd for C_19_H_25_O_2_NI [M + H]^+^ 426.0930, found 426.0919.

*N-(2-Bromopyridin-3-yl)-N-(methoxymethyl)-1-methylcyclohexa-2,5-diene-1-carboxamide* (**1m**). Using the general procedure for MOM group protection, secondary amide **S2m** (0.856 g, 2.92 mmol, 1.0 equiv) in THF (20.4 mL, 0.14 M) was alkylated with chloromethyl methyl ether (0.44 mL, 5.84 mmol, 2.0 equiv). The crude product was purified by column chromatography (silica, 4:1 hexanes/EtOAc) to afford **1m** (0.822 g, 2.45 mmol) in 84% yield as a yellow solid: mp 59.0–61.3 °C; ^1^H NMR (400 MHz, DMSO*-d*_6_) δ 8.29 (dd, *J* = 4.7, 1.9 Hz, 1H), 7.68 (dd, *J* = 7.7, 1.9 Hz, 1H), 7.38 (dd, *J* = 7.8, 4.7 Hz, 1H), 5.60 (s, 2H), 5.47 (s, 1H), 5.32 (s, 1H), 4.30 (s, 1H), 3.25 (s, 3H), 2.41 (d, *J* = 24.3 Hz, 1H), 2.07 (d, *J* = 23.3 Hz, 1H), 1.21 (s, 3H); ^13^C{^1^H} NMR (101 MHz, DMSO-*d*_6_) δ_u_ 149.2, 141.2, 123.8, 56.6, 29.1; δ_d_ 173.9, 144.3, 138.0, 80.2, 46.1, 25.9; GC (method B) *t*_R_ = 2.786 min; EI-MS *m*/*z* (%) 339.1 (M + 2^+^, 1), 306.0 (4), 245.0 (9), 216.0 (32), 200.9 (26), 184.9 (44), 165.0 (25), 137.0 (8), 119.0 (11), 106.0 (8), 93.1 (100), 77.1 (50), 64.0 (10), 51.0 (7); HRMS (ESI) calcd for C_15_H_18_O_2_N_2_Br [M + H]^+^ 337.0552, found 337.0555.

*N-(3-Bromopyridin-2-yl)-N-(methoxymethyl)-1-methylcyclohexa-2,5-diene-1-carboxamide* (**1n**). Using the general procedure for MOM group protection, secondary amide **S2n** (0.737 g, 2.51 mmol, 1.0 equiv) in THF (17.6 mL, 0.14 M) was alkylated with bromomethyl methyl ether (0.82 mL, 10.0 mmol, 4.0 equiv). The crude product was purified by column chromatography (silica, 4:1 hexanes/EtOAc) to afford **1n** (0.547 g, 1.62 mmol) in 65% yield as a yellow oil: ^1^H NMR (400 MHz, DMSO-*d*_6_) δ 8.43 (dd, *J* = 4.6, 1.7 Hz, 1H), 8.11 (dd, *J* = 8.0, 1.7 Hz, 1H), 7.33 (dd, *J* = 7.9, 4.6 Hz, 1H), 5.60–5.42 (m, 4H), 5.05 (s, 1H), 3.27 (s, 3H), 2.49–2.30 (m, 2H), 1.28 (s, 3H); ^13^C{^1^H} NMR (101 MHz, DMSO-*d*_6_) δ_u_ 148.1, 142.5, 129.8, 125.4, 123.5, 56.7, 29.4; δ_d_ 175.0, 153.0, 121.0, 79.6, 46.3, 25.9; GC (method B) *t*_R_ = 2.740 min; EI-MS *m*/*z* (%) 338.1 (M + 1^+^, 1), 304.0 (6), 245.0 (20), 214.9 (21), 200.9 (41), 185.9 (66), 156.0 (33), 135.0 (3), 120.0 (9), 106.0 (6), 93.1 (100), 77.1 (50), 65.1 (11), 51.1 (8); HRMS (ESI) calcd for C_15_H_18_O_2_N_2_Br [M + H]^+^ 337.0552, found 337.0547.

*N-(3-Bromopyridin-2-yl)-1-ethyl-N-(methoxymethyl)cyclohexa-2,5-diene-1-carboxamide* (**1o**). Using the general procedure for MOM group protection, secondary amide **S2o** (1.43 g, 4.66 mmol, 1.0 equiv) in THF (32.6 mL, 0.14 M) was alkylated with bromomethyl methyl ether (2.3 mL, 27.9 mmol, 6.0 equiv). The crude product was purified by column chromatography (silica, 3:2 hexanes/EtOAc) to afford **1o** (1.28 g, 3.64 mmol) in 78% yield as a yellow solid: mp 41.9–44.4 °C; ^1^H NMR (400 MHz, DMSO-*d*_6_) δ 8.42 (d, *J* = 4.6 Hz, 1H), 8.10 (d, *J* = 8.0 Hz, 1H), 7.32 (dd, *J* = 8.0, 4.7 Hz, 1H), 5.56 (d, *J* = 10.2 Hz, 2H), 5.39 (d, *J* = 10.3 Hz, 2H), 5.05 (s, 2H), 3.28 (s, 3H), 2.45–2.36 (m, 2H), 1.74 (q, *J* = 7.5 Hz, 2H), 0.74 (t, *J* = 7.5 Hz, 3H); ^13^C{^1^H} NMR (101 MHz, DMSO-*d*_6_) δ_u_ 148.1, 142.5, 128.0, 125.4, 125.0, 56.8, 8.6; δ_d_ 176.1, 153.0, 121.1, 79.5, 50.7, 33.4, 26.3; GC (method B) *t*_R_ = 3.027 min; EI-MS *m*/*z* (%) 350.1 (M – 1^+^, 1), 320.1 (6), 245.0 (15), 215.0 (19), 200.9 (33), 186.0 (61), 156.9 (29), 134.0 (12), 107.1 (39), 91.1 (50), 79.1 (100), 65.1 (5), 51.1 (6); HRMS (ESI) calcd for C_16_H_20_O_2_N_2_Br [M + H]^+^ 351.0708, found 351.0706.

*1-Ethyl-N-(2-iodopyridin-3-yl)cyclohexa-2,5-diene-1-carboxamide* (**1o-I**). Using the general procedure for MOM group protection, secondary amide **S2o-I** (0.497 g, 1.41 mmol, 1.0 equiv) in THF (9.9 mL, 0.14 M) was alkylated with bromomethyl methyl ether (0.46 mL, 5.65 mmol, 4.0 equiv). The crude product was purified by column chromatography (silica, 4:1 hexanes/EtOAc) to afford **1o-I** (0.283 g, 0.711 mmol) in 50% yield as a tan solid: mp 71.3–72.1 °C; ^1^H NMR (400 MHz, DMSO-*d*_6_) δ 8.45–8.38 (m, 1H), 8.28 (dd, *J* = 7.8, 1.7 Hz, 1H), 7.12 (dd, *J* = 7.9, 4.6 Hz, 1H), 5.56 (d, *J* = 10.7 Hz, 2H), 5.42 (d, *J* = 10.0 Hz, 2H), 5.04 (s, 2H), 3.28 (s, 3H), 2.43 (s, 2H), 1.76 (q, *J* = 7.7 Hz, 2H), 0.76 (t, *J* = 7.4 Hz, 3H); ^13^C{^1^H} NMR (101 MHz, DMSO-*d*_6_) δ_u_ 148.8, 148.6, 128.1, 124.9, 56.8, 8.7; δ_d_ 176.2, 155.9, 97.9, 79.9, 50.7, 33.5, 26.3; GC (method B) *t*_R_ = 3.496 min; EI-MS *m*/*z* (%) 398.1 (M^+^, 1), 366.1 (5), 291.0 (20), 264.0 (30), 246.9 (36), 231.9 (100), 204.9 (37), 165 (6), 134.0 (10), 107.1 (25), 91.1 (25), 79.0 (58), 51.0 (4); HRMS (ESI) calcd for C_16_H_20_O_2_N_2_I [M + H]^+^ 399.0570, found 399.0567.

#### Mizoroki–Heck General Procedure

A vial with a stir bar was either flame-dried under argon or oven-dried, charged with the aryl halide diene (0.1 mmol, 1.0 equiv), and then placed in a glovebox. Anhydrous nickel salt (0.01 mmol, 10 mol %), a ligand (bipy or **L27**) (0.015 mmol, 15 mol %), Mn (0.3 mmol, 3.0 equiv), KI (0.1 mmol, 1.0 equiv), 2-cyclohexenone (0.3 mmol, 3.0 equiv), and DMF (1.2 mL, 0.08 M) were added, and the vial was sealed with a pressure relief cap and removed from the glovebox. The contents of the vial were stirred at 80 °C in a pie reactor until the reaction was determined to be complete by GC-MS analysis (typically 1–6 h). GC-MS analysis was optimal because the reactant and the product have quite similar TLC properties. Upon completion, the reaction mixture was filtered through an aluminum oxide plug to remove Ni and Mn metals and the plug was washed with EtOAc. The resulting organic solution was washed with 1 N HCl (twice) or water (for pyridine- and pyrimidine-containing substrates) and brine, dried with MgSO_4_, and filtered. The crude product was concentrated *in vacuo* and purified by column chromatography.

*(6aR,10aR)-5,6a-Dimethyl-6a,10a-dihydrophenanthridin-6(5H)-one* (**2a**). Racemic Procedure. Using the general procedure detailed above, diene bromide **1a** (0.0995 g, 0.327 mmol, 1.0 equiv) in DMF (3.9 mL, 0.08M) or diene iodide **1a-I** (0.101 g, 0.283 mmol, 1.0 equiv) in DMF (3.4 mL, 0.08 M) was subjected to the Heck reaction conditions with 10 mol % NiI_2_/15 mol % 2,2′-bipyridine for 1.5 or 3 h, respectively. The crude products were purified by column chromatography (silica, 4:1 hexanes/EtOAc) to afford **2a** (0.0626 g, 0.278 mmol) in 85% yield from **1a** or in 98% yield (0.0631 g, 0.280 mmol) from **1a-I** as a yellow oil.

Enantioselective Procedure. Using the general procedure detailed above, diene iodide **1a-I** (0.0449 g, 0.127 mmol, 1.0 equiv) in DMF (1.5 mL, 0.08 M) was subjected to the Heck reaction conditions with 10 mol % NiI_2_/15 mol % *t*Bu-^6^CH_3_*i*Quinox (**L27**) for 1.5 h. The crude products were purified by column chromatography (silica, 4:1 hexanes/EtOAc) to afford **2a** (0.0226 g, 0.100 mmol) with an enantiomeric ratio of 10:1 (82% ee) in 79% yield as a yellow oil. Spectral data were in accordance with the literature:^59^ [α]_D_^20^ = +146 (*c* 0.95, CHCl_3_); ^1^H NMR (400 MHz, CDCl_3_) δ 7.30 (td, *J* = 8.0, 1.6 Hz, 1H), 7.23 (dd, *J* = 7.5, 1.6 Hz, 1H), 7.08 (td, *J* = 7.5, 1.1 Hz, 1H), 6.99 (dd, *J* = 8.2, 1.1 Hz, 1H), 6.12–6.07 (m, 1H), 6.05–5.99 (m, 1H), 5.91–5.87 (m, 1H), 5.58 (ddt, *J* = 9.3, 3.0, 1.0 Hz, 1H), 3.52–3.45 (m, 1H), 3.35 (s, 3H), 1.23 (s, 3H); ^13^C{^1^H} NMR (101 MHz, CDCl_3_) δ_u_ 131. 8, 128. 6, 127.8, 125.2, 124.2, 123.3, 114.3, 44.3, 29.9, 23.4; δ_d_ 173.4, 139.0, 126.6, 40.8; GC (method B) *t*_R_ = 1.940 min; EI-MS *m*/*z* (%) 224.1 (M – 1^+^, 79), 210.1 (100), 195.1 (23), 182.1 (28), 167.1 (30), 152.1 (9), 139.0 (5), 128.0 (5), 115.0 (8), 105.0 (4), 90.9 (7), 77.0 (9), 63.0 (6), 51.1 (6); HPLC (Chiralcel OD-H, 97.5:2.5 *n*-hexane/isopropanol, flow rate of 1 mL/min, λ = 254 nm) *t*_R1_ = 11.39 min (major), *t*_R2_ = 12.87 min (minor).

*(6aR,10aR)-5-(Methoxymethyl)-6a-methyl-6a,10a-dihydrophenanthridin-6(5H)-one* (**2b**). Racemic Procedure. Using the general procedure detailed above, diene bromide **1b** (0.0797 g, 0.237 mmol, 1.0 equiv) in DMF (2.8 mL, 0.08 M) or diene iodide **1b-I** (0.0404 g, 0.104 mmol, 1.0 equiv) in DMF (1.2 mL, 0.08 M) was subjected to the Heck reaction conditions with 10 mol % NiI_2_/15 mol % 2,2′-bipyridine for 2 or 4 h, respectively. The crude product was purified by column chromatography (silica, 10:1 hexanes/EtOAc) to afford **2b** (0.0459 g, 0.179 mmol) in 76% yield from **1b** or in 75% yield (0.0201 g, 0.0787 mmol) from **1b-I** as a white solid: mp 136.5–138.3 °C.

Enantioselective Procedure. Using the general procedure detailed above, diene iodide **1b-I** (0.0504 g, 0.130 mmol, 1.0 equiv) in DMF (1.6 mL, 0.08 M) was subjected to the Heck reaction conditions with 10 mol % NiI_2_/15 mol % *t*Bu-^6^CH_3_*i*Quinox (**L27**) for 2.5 h. The crude product was purified by column chromatography (silica, 10:1 hexanes/EtOAc) to afford **2b** (0.0281 g, 0.110 mmol) with an enantiomeric ratio of 10:1 (82% ee) in 85% yield as a white solid. Spectral data were in accordance with the literature:^59^ [α]_D_^20^ = +312 (*c* 0.74, CHCl_3_); ^1^H NMR (400 MHz, CDCl_3_) δ 7.23–7.19 (m, 2H), 7.15 (dd, *J* = 7.4, 1.3 Hz, 1H), 7.07–7.01 (m, 1H), 6.09–6.02 (m, 1H), 5.99–5.92 (m, 1H), 5.86–5.81 (m, 1H), 5.55–5.49 (m, 2H), 5.03 (d, *J* = 10.7 Hz, 1H), 3.46–3.39 (m, 1H), 3.27 (s, 3H), 1.21 (s, 3H); GC (method B) *t*_R_ = 2.128; EI-MS *m*/*z* (%) 255.1 (35, M^+^), 240.0 (5), 223.0 (23), 210.0 (100), 192.0 (92), 180.0 (43), 165.0 (41), 152.0 (24), 139.0 (5), 128.0 (6), 115.0 (8), 91.0 (10), 77.0 (8); ^13^C{^1^H} NMR (101 MHz, CDCl_3_) δ_u_ 131.6, 128.8, 128.7, 128.0, 125.3, 124.5, 124.0, 115.6, 56.1, 44.5, 23.3; δ_d_ 174.8, 137.6, 126.3, 73.7, 40.9; HPLC (Chiralcel OD-H, 97.5:2.5 *n*-hexane/isopropanol, flow rate of 1 mL/min, λ = 254 nm) *t*_R1_ = 9.54 min (major), *t*_R2_ = 14.52 min (minor).

*(6aR,10aR)-6a-Ethyl-5-(methoxymethyl)-6a,10a-dihydrophenanthridin-6(5H)-one* (**2c**). Racemic Procedure. Using the general procedure detailed above, diene iodide **1c-I** (0.135 g, 0.336 mmol, 1.0 equiv) in DMF (4 mL, 0.08 M) was subjected to the Heck reaction conditions with 10 mol % NiI_2_/15 mol % 2,2′-bipyridine for 2 h. The crude product was purified by column chromatography (silica, 10:1 hexanes/EtOAc) to afford **2c** (0.0786 g, 0.292 mmol) in 87% yield as a clear colorless oil.

Enantioselective Procedure. Using the general procedure detailed above, diene iodide **1c-I** (0.100 g, 0.252 mmol, 1.0 equiv) in DMF (3 mL, 0.08 M) was subjected to the Heck reaction conditions with 10 mol % NiI_2_/15 mol % *t*Bu-^6^CH_3_*i*Quinox (**L27**) for 3 h. The crude product was purified by column chromatography (silica, 10:1 hexanes/EtOAc) to afford **2c** (0.0559 g, 0.208 mmol) with an enantiomeric ratio of 12:1 (84% ee) in 82% yield as a clear colorless oil.

The 1 mmol Scale Enantioselective Procedure. Using the general procedure detailed above, diene iodide **1c-I** (0.397 g, 1.0 mmol, 1.0 equiv) in DMF (12 mL, 0.08 M) was subjected to the Heck reaction conditions with 10 mol % NiI_2_/15 mol % *t*Bu-^6^CH_3_*i*Quinox (**L27**) for 6 h. The crude product was purified by column chromatography (silica, 10:1 hexanes/EtOAc) to afford **2c** (0.142 g, 0.527 mmol) with an enantiomeric ratio of 9:1 (80% ee) in 53% yield as a clear colorless oil. Spectral data were in accordance with the literature:^59^ [α]_D_^20^ = +157 (*c* 0.35, CHCl_3_); ^1^H NMR (400 MHz, CDCl_3_) δ 7.31–7.24 (m, 2H), 7.21 (d, 1H), 7.10 (td, *J* = 6.9, 2.0 Hz, 1H), 6.22–6.14 (m, 1H), 6.04–5.99 (m, 1H), 5.95 (d, *J* = 9.1 Hz, 1H), 5.63 (d, *J* = 10.6 Hz, 1H), 5.55 (dd, *J* = 9.4, 2.5 Hz, 1H), 5.05 (d, *J* = 10.6 Hz, 1H), 3.70–3.64 (m, 1H), 3.34 (s, 3H), 1.75–1.64 (m, 1H), 1.59–1.46 (m, 1H), 0.91 (t, *J* = 7.4 Hz, 3H); ^13^C{^1^H} NMR (101 MHz, CDCl_3_) δ_u_ 129.8, 128.9, 128.6, 128.0, 125.2, 125.2, 124.0, 115.5, 56.2, 41.3, 9.1; δ_d_ 174.4, 137.8, 126.2, 73.8, 45.2, 27.9; GC (method B) *t*_R_ = 2.380; EI-MS *m*/*z* (%) 269.1 (36, M^+^), 237.0 (28), 224.0 (100), 208.0 (91), 196.0 (64), 178.0 (82), 167.0 (28), 152.0 (26), 139.0 (5), 115.0 (5), 91.0 (8), 77.0 (8); HPLC (Chiralcel OD-H, 97.5:2.5 *n*-hexane/isopropanol, flow rate of 1 mL/min, λ = 254 nm) *t*_R1_ = 8.27 min (major), *t*_R2_ = 14.30 min (minor).

*(6aR,10aR)-6a-Isopropyl-5-methyl-6a,10a-dihydrophenanthridin-6(5H)-one* (**2d**). Racemic Procedure. Using the general procedure detailed above, diene bromide **1d** (0.0954 g, 0.285 mmol, 1.0 equiv) in DMF (3.4 mL, 0.08 M) or diene iodide **1d-I** (0.0993 g, 0.262 mmol, 1.0 equiv) in DMF (3.1 mL, 0.08 M) was subjected to the Heck reaction conditions with 10 mol % NiI_2_/15 mol % 2,2′-bipyridine for 1.5 or 3 h, respectively. The crude products were purified by column chromatography (silica, 10:1 hexanes/EtOAc) to afford **2d** (0.0613 g, 0.242 mmol) in 85% yield from **1d** or in 90% yield (0.0595 g, 0.235 mmol) from **1d-I** as a clear colorless oil.

Enantioselective Procedure. Using the general procedure detailed above, diene iodide **1d-I** (0.0499 g, 0.131 mmol, 1.0 equiv) in DMF (1.6 mL, 0.08 M) was subjected to the Heck reaction conditions with 10 mol % NiI_2_/15 mol % *t*Bu-^6^CH_3_*i*Quinox (**L27**) for 2.5 h. The crude product was purified by column chromatography (silica, 10:1 hexanes/EtOAc) to afford **2d** (0.0273 g, 0.108 mmol) with an enantiomeric ratio of 9:1 (80% ee) in 83% yield as a clear colorless oil. Spectral data were in accordance with the literature:^59^ [α]_D_^20^ = +270 (*c* 0.5, CHCl_3_); ^1^H NMR (400 MHz, CDCl_3_) δ 7.23 (ddd, *J* = 8.1, 7.5, 1.6 Hz, 1H), 7.13 (dd, *J* = 7.3, 1.6 Hz, 1H), 7.00 (td, *J* = 7.4, 1.1 Hz, 1H), 6.90 (dd, *J* = 8.2, 1.1 Hz, 1H), 6.17–6.08 (m, 1H), 6.00–5.86 (m, 2H), 5.48–5.40 (m, 1H), 3.74 (s, 1H), 3.29 (s, 3H), 1.73–1.58 (m, 1H), 0.86 (d, *J* = 6.9 Hz, 3H), 0.74 (d, *J* = 6.8 Hz, 3H); ^13^C{^1^H} NMR (101 MHz, CDCl_3_) δ_u_ 129.2, 128.5, 127.8, 127.2, 125.4, 125.2, 123.3, 114.2, 41.09, 29.9, 29.8, 18.5, 18.0; δ_d_ 172.6, 139.3, 126.9, 48.5; GC (method B) *t*_R_ = 2.294; EI-MS *m*/*z* (%) 253.1 (12, M^+^), 210.1 (100), 195.0 (23), 180.0 (7), 167.0 (10), 152.0 (7); HPLC (Chiralcel OD-H, 97.5:2.5 *n*-hexane/isopropanol, flow rate of 1 mL/min, λ = 254 nm) *t*_R1_ = 9.96 min (major), *t*_R2_ = 11.76 min (minor).

*(6aR,10aR)-6a-Isopropyl-5-(methoxymethyl)-6a,10a-dihydrophenanthridin-6(5H)-one* (**2e**). Racemic Procedure. Using the general procedure detailed above, diene bromide **1e** (0.0988 g, 0.275 mmol, 1.0 equiv) in DMF (3.3 mL, 0.08 M) or diene iodide **1e-I** (0.103 g, 0.243 mmol, 1.0 equiv) in DMF (2.9 mL, 0.08 M) was subjected to the Heck reaction conditions with 10 mol % NiI_2_/15 mol % 2,2′-bipyridine for 2 or 2.5 h, respectively. The crude products were purified by column chromatography (silica, 10:1 hexanes/EtOAc) to afford **2e** (0.0708 g, 0.24 mmol) in 90% yield from **1e** or in 91% yield (0.0625 g, 0.221 mmol) from **1e-I** as a white solid: mp 104.4–106.5 °C.

Chiral Procedure. Using the general procedure detailed above, diene iodide **1e-I** (0.103 g, 0.243 mmol, 1.0 equiv) in DMF (2.9 mL, 0.08 M) was subjected to the Heck reaction conditions with 10 mol % NiI_2_/15 mol % *t*Bu-^6^CH_3_*i*Quinox (**L27**) for 3 h. The crude product was purified by column chromatography (silica, 10:1 hexanes/EtOAc) to afford **2e** (0.0460 g, 0.162 mmol) with an enantiomeric ratio of 11:1 (84% ee) in 67% yield as a tan oil that solidified to white crystals upon cooling: [α]_D_^20^ = +160 (*c* 0.65, CHCl_3_); ^1^H NMR (400 MHz, CDCl_3_) δ 7.32–7.22 (m, 2H), 7.19 (dd, *J* = 7.4, 1.5 Hz, 1H), 7.10 (td, *J* = 7.2, 1.8 Hz, 1H), 6.22 (ddd, *J* = 9.6, 5.2, 1.0 Hz, 1H), 6.07–5.95 (m, 2H), 5.66 (d, *J* = 10.6 Hz, 1H), 5.53 (ddt, *J* = 9.3, 2.2, 1.0 Hz, 1H), 5.02 (d, *J* = 10.5 Hz, 1H), 3.81 (t, *J* = 3.2 Hz, 1H), 3.36 (s, 3H), 1.79 (hept, *J* = 6.9 Hz, 1H), 0.98 (d, *J* = 6.9 Hz, 3H), 0.84 (d, *J* = 6.8 Hz, 3H); ^13^C{^1^H} NMR (101 MHz, CDCl_3_) δ_u_ 129.2, 128.4, 128.0, 126.7, 125.8, 125.4, 124.0, 115.5, 74.1, 56.3, 48.7, 41.3, 40.7, 29.3, 18.5, 17.7; δ_d_ 173.8, 138.2, 126.6, 74.1, 48.7; GC (method B) *t*_R_ = 2.560 min; EI-MS *m*/*z* (%) 283.1 (M^+^, 17), 251.0 (12), 240.0 (17), 224 (10), 208.0 (100), 196.0 (99), 178 (67), 167 (14), 152.0 (17), 139 (4), 115.0 (4), 105.0 (4), 77.0 (5); HRMS (ESI) calcd for C_18_H_22_O_2_N [M + H]^+^ 284.1651, found 284.1646; HPLC )Chiralcel OD-H, 97.5:2.5 *n*-hexane/isopropanol, flow rate of 1 mL/min, λ = 254 nm) *t*_R1_ = 7.80 min (major), *t*_R2_ = 13.46 min (minor).

*(6aR,10aR)-5,6a-Bis(methoxymethyl)-6a,10a-dihydrophenanthridin-6(5H)-one* (**2f**). Racemic Procedure. Using the general procedure detailed above, diene iodide **1f-I** (0.100 g, 0.242 mmol, 1.0 equiv) in DMF (2.9 mL, 0.08 M) was subjected to the Heck reaction conditions with 10 mol % NiI_2_/15 mol % 2,2′-bipyridine for 3 h. The crude product was purified by column chromatography (silica, 7:1 hexanes/EtOAc) to afford **2f** (0.0630 g, 0.221 mmol) in 91% yield as a yellow oil.

Enantioselective Procedure. Using the general procedure detailed above, diene iodide **1f-I** (0.0500 g, 0.121 mmol, 1.0 equiv) in DMF (1.5 mL, 0.08 M) was subjected to the Heck reaction conditions with 10 mol % NiI_2_/15 mol % *t*Bu-^6^CH_3_*i*Quinox (**L27**) for 4 h. The crude product was purified by column chromatography (silica, 7:1 hexanes/EtOAc) to afford **2f** (0.0313 g, 0.110 mmol) with an enantiomeric ratio of 12:1 (85% ee) in 91% yield as a yellow oil: [α]_D_^20^ = +126 (*c* 0.21, CHCl_3_); ^1^H NMR (400 MHz, CDCl_3_) δ 7.22–7.18 (m, 2H), 7.18–7.14 (m, 1H), 7.06–7.00 (m, 1H), 6.12–6.04 (m, 1H), 6.02–5.92 (m, 1H), 5.86–5.79 (m, 1H), 5.71–5.63 (m, 1H), 5.40 (d, *J* = 10.6 Hz, 1H), 5.17 (d, *J* = 10.6 Hz, 1H), 3.88–3.82 (m, 1H), 3.46–3.36 (m, 2H), 3.29 (s, 3H), 3.23 (s, 3H); ^13^C{^1^H} NMR (101 MHz, CDCl_3_) δ_u_ 128.2, 127.9, 127.9, 127.5, 125.8, 124.8, 124.0, 115.5, 59.5, 56.2, 37.6; δ_d_ 171.9, 137.8, 125.8, 74.1, 73.5, 47.1; GC (method B) *t*_R_ = 2.597 min; EI-MS *m*/*z* (%) 285.1 (M^+^, 1), 240.0 (5), 221.0 (5), 209.0 (100), 195.0 (11), 180.0 (45), 165.0 (10), 152.0 (10); HRMS (ESI) calcd for C_17_H_20_O_3_N [M + H]^+^ 286.1443, found 286.1442; HPLC (Chiralcel OD-H, 97.5:2.5 *n*-hexane/isopropanol, flow rate of 1 mL/min, λ = 254 nm) *t*_R1_ = 18.33 min (major), *t*_R2_ = 24.17 min (minor).

*Ethyl 2-[(6aR,10aR)-5-Methyl-6-oxo-5,10a-dihydrophenanthridin-6a(6H)-yl]acetate* (**2g**). Racemic Procedure. Using the general procedure detailed above, diene iodide **1g-I** (0.202 g, 0.475 mmol, 1.0 equiv) in DMF (5.7 mL, 0.08 M) was subjected to the Heck reaction conditions with 10 mol % NiI_2_/15 mol % 2,2′-bipyridine for 2 h. The crude product was purified by column chromatography (silica, 10:1 hexanes/EtOAc) to afford **2g** (0.104 g, 0.349 mmol) in 74% yield as a clear colorless oil.

Enantioselective Procedure. Using the general procedure detailed above, diene iodide **1g-I** (0.0501 g, 0.118 mmol, 1.0 equiv) in DMF (1.4 mL, 0.08 M) was subjected to the Heck reaction conditions with 10 mol % NiI_2_/15 mol % *t*Bu-^6^CH_3_*i*Quinox (**L27**) for 2 h. The crude product was purified by column chromatography (silica, 10:1 hexanes/EtOAc) to afford **2g** (0.0249 g, 0.0837 mmol) with an enantiomeric ratio of 3:1 (49% ee) in 71% yield as a clear colorless oil. Spectral data were in accordance with the literature:^59^ [α]_D_^20^ = +209 (*c* 0.86, CHCl_3_); ^1^H NMR (400 MHz, CDCl_3_) δ 7.31–7.21 (m, 2H), 7.05 (td, *J* = 7.5, 1.1 Hz, 1H), 6.97 (dd, *J* = 8.1, 1.1 Hz, 1H), 6.14–5.99 (m, 2H), 5.95–5.85 (m, 1H), 5.85–5.75 (m, 1H), 4.17–4.01 (m, 3H), 3.38 (s, 3H), 2.70 (q, 2H), 1.23 (t, *J* = 7.1 Hz, 3H); ^13^C{^1^H} NMR (101 MHz, CDCl_3_) δ_u_ 128.5, 127.9, 127.8, 127.7, 125.2, 125.0, 123.3, 114.2, 38.5, 30.2, 14.2; δ_d_ 171.4, 170.9, 139.4, 125.8, 60.6, 44.3, 37.8; GC (method B) *t*_R_ = 3.162 min; EI-MS *m*/*z* (%) 296.1 (M^+^, 1), 252.1 (5), 210.0 (100), 195.0 (10), 180.0 (15), 165.0 (7), 152.0 (8); HPLC (Chiralcel OD-H, 60:40 *n*-hexane/isopropanol, flow rate of 1 mL/min, λ = 254 nm) *t*_R1_ = 18.93 min (major), *t*_R2_ = 24.37 min (minor).

*(6aR,10aR)-6a-Benzyl-5-(methoxymethyl)-6a,10a-dihydrophenanthridin-6(5H)-one* (**2h**). Racemic Procedure. Using the general procedure detailed above, diene bromide **1h** (0.0990 g, 0.241 mmol, 1.0 equiv) in DMF (2.9 mL, 0.08 M) or diene iodide **1h-I** (0.0504 g, 0.109 mmol, 1.0 equiv) in DMF (1.3 mL, 0.08M) was subjected to the Heck reaction conditions with 10 mol % NiI_2_/15 mol % 2,2′-bipyridine for 2 or 4 h, respectively. The crude product was purified by column chromatography (silica, 10:1 hexanes/EtOAc) to afford **2h** (0.0721 g, 0.218 mmol) in 90% yield from **1h** or in 97% yield (0.0625 g, 0.105 mmol) from **1h-I** as a yellow oil.

Chiral Procedure. Using the general procedure detailed above, diene iodide **1h-I** (0.0503 g, 0.109 mmol, 1.0 equiv) in DMF (1.3 mL, 0.08M) was subjected to the Heck reaction conditions with 10 mol % NiI_2_/15 mol % *t*Bu-^6^CH_3_*i*Quinox (**L27**) for 3 h. The crude product was purified by column chromatography (silica, 10:1 hexanes/EtOAc) to afford **2h** (0.0237 g, 0.0715 mmol) with an enantiomeric ratio of 11:1 (83% ee) in 66% yield as a tan oil. Spectral data were in accordance with the literature:^59^ [α]_D_^20^ = +50 (*c* 0.23, CHCl_3_); ^1^H NMR (400 MHz, CDCl_3_) δ 7.33–7.30 (m, 2H), 7.30–7.26 (m, 1H), 7.25–7.19 (m, 2H), 7.18–7.14 (m, 2H), 7.04–6.99 (m, 2H), 6.10–6.04 (m, 2H), 5.98–5.89 (m, 1H), 5.59 (d, *J* = 10.6 Hz, 1H), 5.55 (dd, *J* = 9.4, 2.9 Hz, 1H), 5.18 (d, *J* = 10.6 Hz, 1H), 3.62–3.58 (m, 1H), 3.39 (s, 3H), 3.01 (d, *J* = 13.3 Hz, 1H), 2.86 (d, *J* = 13.3 Hz, 1H); ^13^C{^1^H} NMR (101 MHz, CDCl_3_) δ_u_ 130.7, 130.2, 128.7, 128.4, 128.3, 128.1, 126.8, 125.3, 124.7, 124.2, 115.7, 56.4, 40.2; δ_d_ 174.1, 137.9, 136.3, 125.9, 74.0, 46.8, 40.9; GC (method A) *t*_R_ = 17.679 min; EI-MS *m*/*z* (%) 331.1 (M^+^, 1), 239.0 (10), 224.0 (19), 208.0 (100), 196.0 (15), 178.0 (26), 165.0 (6), 152.0 (7), 91.0 (40); HPLC (Chiralcel OD-H, 97.5:2.5 *n*-hexane/isopropanol, flow rate of 1 mL/min, λ = 254 nm) *t*_R1_ = 9.21 min (major), *t*_R2_ = 15.29 min (minor).

*(6aR,10aR)-2-Fluoro-6a-isopropyl-5-(methoxymethyl)-6a,10a-dihydrophenanthridin-6(5H)-one* (**2i**). Racemic Procedure. Using the general procedure detailed above, diene bromide **1i** (0.101 g, 0.262 mmol, 1.0 equiv) in DMF (3.1 mL, 0.08 M) or diene iodide **1i-I** (0.0501 g, 0.117 mmol, 1.0 equiv) in DMF (1.4 mL, 0.08 M) was subjected to the Heck reaction conditions with 10 mol % NiI_2_/15 mol % 2,2′-bipyridine for 4 h. The crude product was purified by column chromatography (silica, 10:1 hexanes/EtOAc) to afford **2i** (0.0669 g, 0.222 mmol) in 85% yield from **1i** or in 61% yield (0.0214 g, 0.0710 mmol) from **1i-I** as a white solid: mp 130.3–132.0 °C.

Enantioselective Procedure. Using the general procedure detailed above, diene iodide **1i-I** (0.0502 g, 0.117 mmol, 1.0 equiv) in DMF (1.4 mL, 0.08M) was subjected to the Heck reaction conditions with 10 mol % NiI_2_/15 mol % *t*Bu-^6^CH_3_*i*Quinox (**L27**) for 6 h. The crude product was purified by column chromatography (silica, 10:1 hexanes/EtOAc) to afford **2i** (0.0307 g, 0.102 mmol) with an enantiomeric ratio of 10:1 (81% ee) in 87% yield as a white oil that solidified to white crystals upon cooling: [α]_D_^20^ = +152 (*c* 0.21, CHCl_3_); ^1^H NMR (400 MHz, CDCl_3_) δ 7.23 (dd, *J* = 8.9, 4.8 Hz, 1H), 6.97 (td, *J* = 8.6, 3.0 Hz, 1H), 6.92 (dd, *J* = 8.2, 2.9 Hz, 1H), 6.26–6.17 (m, 1H), 6.07–5.94 (m, 2H), 5.64 (d, *J* = 10.7 Hz, 1H), 5.51 (ddt, *J* = 9.4, 2.2, 1.0 Hz, 1H), 4.97 (d, *J* = 10.8 Hz, 1H), 3.78 (s, 1H), 3.35 (s, 3H), 1.77 (hept, *J* = 6.9 Hz, 1H), 0.99 (d, *J* = 6.9 Hz, 3H), 0.85 (d, *J* = 6.8 Hz, 3H); ^13^C{^1^H} NMR (101 MHz, CDCl_3_) δ_u_ 128.2, 126.7, 125.8 (d, ^3^*J*_C–F_ = 12.1 Hz), 117.0, 116.9, 115.2 (d, ^2^*J*_C–F_ = 23.2 Hz), 114.4 (d, ^2^*J*_C–F_ = 23.2 Hz), 56.3, 41.4, 29.3, 18.5, 17.9; δ_d_ 173.3, 160.5 (d, ^1^*J*_C–F_ = 245.5 Hz), 134.4, 128.6, 74.4, 48.5; ^19^F NMR (376 MHz, CDCl_3_) δ −119.34; GC (method B) *t*_R_ = 2.490 min; EI-MS *m*/*z* (%) 301.1 (M^+^, 32), 269 (14), 258.0 (7), 242.0 (9), 226.0 (77), 214.0 (100), 196.0 (58), 185.0 (14), 170.0 (10), 105.0 (5); HRMS (ESI) calcd for C_18_H_21_O_2_NF [M + H]^+^ 302.1556, found 302.1550; HPLC (Chiralcel OD-H, 97.5:2.5 *n*-hexane/isopropanol, flow rate of 1 mL/min, λ = 254 nm) *t*_R1_ = 9.53 min (major), *t*_R2_ = 16.15 min (minor).

*(6aR,10aR)-2-Chloro-6a-isopropyl-5-(methoxymethyl)-6a,10a-dihydrophenanthridin-6(5H)-one* (**2j**). Racemic Procedure. Using the general procedure detailed above, diene bromide **1j** (0.0999 g, 0.251 mmol, 1.0 equiv) in DMF (3 mL, 0.08 M) or diene iodide **1j-I** (0.0502 g, 0.112 mmol, 1.0 equiv) in DMF (1.3 mL, 0.08 M) was subjected to the Heck reaction conditions with 10 mol % NiI_2_/15 mol % 2,2′-bipyridine for 6 h at 65 °C. The crude product was purified by column chromatography (silica, 10:1 hexanes/EtOAc) to afford **2j** (0.0531 g, 0.167 mmol) in 67% yield from **1j** or in 91% yield (0.0324 g, 0.102 mmol) from **1j-I** as a tan solid: mp 101.1–102.9 °C.

Enantioselective Procedure. Using the general procedure detailed above, diene iodide **1j-I** (0.0508 g, 0.114 mmol, 1.0 equiv) in DMF (1.4 mL, 0.08 M) was subjected to the Heck reaction conditions with 10 mol % NiI_2_/15 mol % *t*Bu-^6^CH_3_*i*Quinox (**L27**) for 6 h at 65 °C. The crude product was purified by column chromatography (silica, 10:1 hexanes/EtOAc) to afford **2j** (0.0242 g, 0.0761 mmol) with an enantiomeric ratio of 7:1 (74% ee) in 68% yield as a clear oil that solidified to tan crystals on cooling: [α]_D_^20^ = +99 (*c* 0.22, CHCl_3_); ^1^H NMR (400 MHz, CDCl_3_) δ 7.20–7.16 (m, 1H), 7.14 (s, 1H), 7.11 (d, *J* = 2.3 Hz, 1H), 6.19–6.10 (m, 1H), 5.99–5.93 (m, 1H), 5.90 (d, *J* = 10.7 Hz, 1H), 5.56 (d, *J* = 10.7 Hz, 1H), 5.47–5.39 (m, 1H), 4.91 (d, *J* = 10.6 Hz, 1H), 3.74–3.68 (m, 1H), 3.27 (s, 3H), 1.70 (hept, *J* = 6.7 Hz, 1H), 0.91 (d, *J* = 6.9 Hz, 3H), 0.79 (d, *J* = 6.8 Hz, 3H); ^13^C{^1^H} NMR (101 MHz, CDCl_3_) δ_u_ 128.3, 128.2, 127.9, 126.6, 125.9, 125.8, 116.9, 56.3, 41.2, 29.3, 18.5, 17.8; δ_d_ 173.4, 136.9, 129.0, 128.5, 74.2, 48.6; GC (method B) *t*_R_ = 3.135 min; EI-MS *m*/*z* (%) 317.1 (M^+^, 29), 285.1 (10), 272.0 (9), 258.0 (10), 242.0 (86), 230.0 (100), 214.0 (61), 195.0 (12), 180.0 (13), 166.0 (13), 152.0 (14), 139.0 (6), 105.0 (7), 77.0 (5); HRMS (ESI) calcd for C_18_H_21_O_2_NCl [M + H]^+^ 318.1261, found 318.1260; HPLC (Chiralcel OD-H, 97.5:2.5 *n*-hexane/isopropanol, flow rate of 1 mL/min, λ = 254 nm) *t*_R1_ = 8.98 min (major), *t*_R2_ = 13.42 min (minor).

*6a-Isopropyl-5-(methoxymethyl)-2-methyl-6a,10a-dihydrophenanthridin-6(5H)-one* (**2k**). Using the general procedure detailed above, diene bromide **1k** (0.101 g, 0.264 mmol, 1.0 equiv) in DMF (3.2 mL, 0.08 M) was subjected to the Heck reaction conditions with 10 mol % NiI_2_/15 mol % 2,2′-bipyridine for 3 h. The crude product was purified by column chromatography (silica, 10:1 hexanes/EtOAc) to afford **2k** (0.0684 g, 0.229 mmol) in 87% yield as a clear colorless oil: ^1^H NMR (400 MHz, CDCl_3_) δ 7.14 (d, *J* = 8.3 Hz, 1H), 7.08 (dd, *J* = 8.1, 2.3 Hz, 1H), 6.99 (d, *J* = 2.1 Hz, 1H), 6.25–6.16 (m, 1H), 6.06–5.94 (m, 2H), 5.63 (d, *J* = 10.6 Hz, 1H), 5.52 (ddt, *J* = 9.3, 2.2, 1.1 Hz, 1H), 5.01 (d, *J* = 10.6 Hz, 1H), 3.76 (d, *J* = 3.3 Hz, 1H), 3.34 (s, 3H), 2.34 (s, 3H), 1.78 (hept, *J* = 7.0 Hz, 1H), 0.98 (d, *J* = 6.9 Hz, 3H), 0.84 (d, *J* = 6.8 Hz, 3H); ^13^C{^1^H} NMR (101 MHz, CDCl_3_) δ_u_ 129.3, 129.1, 128.4, 126.8, 125.8, 125.3, 115.4, 56.3, 41.3, 29.2, 20.7, 18.5, 17.9; δ_d_ 173.7, 135.7, 133.6, 126.4, 74.1, 48.7; GC (method B) *t*_R_ = 2.836 min; EI-MS *m*/*z* (%) 297.1 (M^+^, 28), 265.1 (5), 254.1 (8), 238.0 (6), 222.0 (98), 210.0 (100), 192.0 (53), 180.0 (14), 165.0 (16), 152.0 (11), 77.0 (4); HRMS (ESI) calcd for C_19_H_24_O_2_N [M + H]^+^ 298.1807, found 298.1803.

*(6aR,10aR)-6a-Isopropyl-5-(methoxymethyl)-3-methyl-6a,10a-dihydrophenanthridin-6(5H)-one* (**2l**). Racemic Procedure. Using the general procedure detailed above, diene iodide **1l-I** (0.101 g, 0.235 mmol, 1.0 equiv) in DMF (2.8 mL, 0.08 M) was subjected to the Heck reaction conditions with 10 mol % NiI_2_/15 mol % 2,2′-bipyridine for 2 h. The crude product was purified by column chromatography (silica, 10:1 hexanes/EtOAc) to afford **2l** (0.0562 g, 0.189 mmol) in 81% yield as a white cloudy oil.

Enantioselective Procedure. Using the general procedure detailed above, diene iodide **1l-l** (0.0501 g, 0.118 mmol, 1.0 equiv) in DMF (1.4 mL, 0.08 M) was subjected to the Heck reaction conditions with 10 mol % NiI_2_/15 mol % *t*Bu-^6^CH_3_*i*Quinox (**L27**) for 1.5 h. The crude product was purified by column chromatography (silica, 10:1 hexanes/EtOAc) to afford **2l** (0.0222 g, 0.0746 mmol) with an enantiomeric ratio of 10:1 (81% ee) in 63% yield as a white cloudy oil: [α]_D_^20^ = +130 (*c* 0.39, CHCl_3_); ^1^H NMR (400 MHz, CDCl_3_) δ 7.03–6.97 (m, 2H), 6.88–6.81 (m, 1H), 6.17–6.09 (m, 1H), 5.96–5.88 (m, 2H), 5.58 (d, *J* = 10.6 Hz, 1H), 5.47–5.43 (m, 1H), 4.93 (d, *J* = 10.6 Hz, 1H), 3.73–3.65 (m, 1H), 3.30 (s, 3H), 2.30 (s, 3H), 1.72 (hept, *J* = 6.9 Hz, 1H), 0.90 (d, *J* = 6.9 Hz, 3H), 0.76 (d, *J* = 6.8 Hz, 3H); ^13^C{^1^H} NMR (101 MHz, CDCl_3_) δ_u_ 129.6, 128.3, 126.7, 125.8, 125.2, 124.7, 116.2, 56.4, 40.9, 29.2, 21.6, 18.5, 17.9; δ_d_ 174.0, 138.1, 137.9, 123.6, 74.1, 48.6; GC (method B) *t*_R_ = 2.775 min; EI-MS *m*/*z* (%) 297.1 (M^+^, 18), 266.1 (4), 254.1 (22), 238.0 (6), 222.0 (100), 210.0 (68), 192.0 (45), 180.0 (10), 165.0 (12), 152.0 (8), 105.0 (5); HRMS (ESI) calcd for C_19_H_24_O_2_N [M + H]^+^ 298.1807, found 298.1800; HPLC (Chiralcel OD-H, 97.5:2.5 *n*-hexane/isopropanol, flow rate of 1 mL/min, λ = 254 nm) *t*_R1_ = 7.32 min (major), *t*_R2_ = 12.13 min (minor).

*5-(Methoxymethyl)-6a-methyl-6a,9,10,10a-tetrahydrobenzo[c][1,5]naphthyridin-6(5H)-one* (**2m-2**). Using the general procedure detailed above, diene bromide **1m** (0.100 g, 0.297 mmol, 1.0 equiv) in DMF (3.6 mL, 0.08 M) was subjected to the Heck reaction conditions with 10 mol % NiI_2_/15 mol % 2,2′-bipyridine for 6 h. The crude product was purified by column chromatography (silica, 10:1 hexanes/EtOAc) to afford **2m-2** (0.0635 g, 0.246 mmol) in 85% yield as a yellow oil: ^1^H NMR (400 MHz, CDCl_3_) δ 8.30 (dd, *J* = 4.8, 1.4 Hz, 1H), 7.54 (dd, *J* = 8.3, 1.4 Hz, 1H), 7.19 (dd, *J* = 8.3, 4.8 Hz, 1H), 5.91 (d, *J* = 1.5 Hz, 2H), 5.62 (d, *J* = 10.8 Hz, 1H), 5.04 (d, *J* = 10.8 Hz, 1H), 3.37 (s, 3H), 2.91 (dd, *J* = 12.9, 3.0 Hz, 1H), 2.26–2.20 (m, 1H), 2.19–2.14 (m, 1H), 1.98–1.91 (m, 1H), 1.69–1.62 (m, 1H), 1.27 (s, 3H); ^13^C{^1^H} NMR (101 MHz, CDCl_3_) δ_u_ 144.4, 129.8, 128.0, 122.4, 56.3, 47.0, 24.9; δ_d_ 174.8, 148.5, 133.5, 73.6, 42.5, 26.0, 24.7; GC (method B) *t*_R_ = 2.260 min; EI-MS *m*/*z* (%) 258.1 (M^+^, 100), 243.0 (99), 227.0 (6), 211.0 (21), 205.0 (13), 197.0 (23), 185.0 (85), 171.0 (25), 156.0 (5), 144.0 (4), 131.0 (5), 91.0 (6), 77.0 (6); HRMS (ESI) calcd for C_15_H_19_O_2_N_2_ [M + H]^+^ 259.1447, found 259.1447.

*5-(Methoxymethyl)-6a-methyl-6a,10a-dihydrobenzo[c][1,8]naphthyridin-6(5H)-one* (**2n**). Using the general procedure detailed above, diene bromide **1n** (0.0895 g, 0.267 mmol, 1.0 equiv) in DMF (3.2 mL, 0.08 M) was subjected to the Heck reaction conditions with 10 mol % NiI_2_/15 mol % 2,2′-bipyridine for 5 h. The crude product was purified by column chromatography (silica, 4:1 hexanes/EtOAc) to afford **2n** (0.0542 g, 0.211 mmol) in 80% yield as a tan solid: mp 124.9–126.0 °C; ^1^H NMR (400 MHz, CDCl_3_) δ 8.37 (dd, *J* = 5.0, 1.8 Hz, 1H), 7.60–7.57 (m, 1H), 7.05 (dd, *J* = 7.4, 4.9 Hz, 1H), 6.17–6.07 (m, 2H), 5.92–5.88 (m, 1H), 5.73 (d, *J* = 9.6 Hz, 1H), 5.65–5.58 (m, 1H), 5.56 (d, *J* = 9.7 Hz, 1H), 3.51 (t, *J* = 3.1 Hz, 1H), 3.41 (s, 3H), 1.33 (s, 3H); ^13^C{^1^H} NMR (101 MHz, CDCl_3_) δ_u_ 147.0, 136.9, 131.3, 127.4, 125.9, 124.6, 119.3, 57.1, 42.7, 23.3; δ_d_ 175.1, 150.0, 121.3, 71.4, 41.2; GC (method B) *t*_R_ = 2.290 min; EI-MS *m*/*z* (%) 256.1 (M^+^, 13), 241.0 (46), 224.0 (82), 211.0 (71), 195.0 (25), 181.0 (24), 167.0 (100), 153.0 (8), 142.0 (10), 133.0 (18), 129.1 (7), 121.0 (11), 115.0 (12), 109.0 (4), 91.0 (15), 79.0 (6), 65.0 (4); HRMS (ESI) calcd for C_15_H_17_O_2_N_2_ [M + H]^+^ 257.1290, found 257.1289.

*(6aR,10aR)-6a-Ethyl-5-(methoxymethyl)-6a,10a-dihydrobenzo[c][1,8]naphthyridin-6(5H)-one* (**2o**). Racemic Procedure. Using the general procedure detailed above, diene bromide **1o** (0.151 g, 0.427 mmol, 1.0 equiv) in DMF (5.1 mL, 0.08 M) or diene iodide **1o-I** (0.100 g, 0.251 mmol, 1.0 equiv) in DMF (3 mL, 0.08 M) was subjected to the Heck reaction conditions with 10 mol % NiI_2_/15 mol % 2,2′-bipyridine for 3 or 4 h, respectively. The crude product was purified by column chromatography (silica, 4:1 hexanes/EtOAc) to afford **2o** (0.0873 g, 0.323 mmol) in 76% yield from **1o** or in 82% yield (0.0557 g, 0.206 mmol) from **1o-I** as a clear colorless oil.

Enantioselective Procedure. Using the general procedure detailed above, diene iodide **1o-I** (0.0198 g, 0.0502 mmol, 1.0 equiv) in DMF (0.6 mL, 0.08 M) was subjected to the Heck reaction conditions with 10 mol % NiI_2_/15 mol % *t*Bu-^6^F*i*Quinox (**L26**) for 2.5 h. The crude product was purified by column chromatography (silica, 10:1 hexanes/EtOAc) to afford **2o** (0.0103 g, 0.0381 mmol) with an enantiomeric ratio of 7:1 (76% ee) in 76% yield as a clear colorless oil: [α]_D_^20^ = +73 (*c* 0.16, CHCl_3_); ^1^H NMR (400 MHz, CDCl_3_) δ 8.27 (dd, *J* = 4.9, 1.8 Hz, 1H), 7.48 (dd, *J* = 7.3, 0.6 Hz, 1H), 6.96 (dd, *J* = 7.4, 4.9 Hz, 1H), 6.16–6.08 (m, 1H), 6.05–5.95 (m, 1H), 5.91–5.83 (m, 1H), 5.65 (d, *J* = 9.7 Hz, 1H), 5.49–5.40 (m, 2H), 3.62–3.56 (m, 1H), 3.31 (s, 3H), 1.65–1.59 (m, 1H), 1.49–1.43 (m, 1H), 0.85 (t, *J* = 7.5 Hz, 3H); ^13^C{^1^H} NMR (101 MHz, CDCl_3_) δ_u_ 146.9, 136.9, 129.6, 127.6, 125.9, 125.3, 119.3, 57.1, 39.7, 9.1; δ_d_ 174.8, 150.1, 121.3, 71.4, 45.4, 28.1; GC (method B) *t*_R_ = 2.626 min; EI-MS *m*/*z* (%) 270.1 (M^+^, 10), 255.1 (24), 238.1 (100), 225.1 (53), 209.1 (25), 197.0 (52), 181.1 (36), 167.1 (50), 154.0 (16), 140.0 (8), 132.0 (14), 127.0 (19), 121.0 (19), 115.0 (6), 91.0 (10), 77.0 (7); HRMS (ESI) calcd for C_16_H_19_O_2_N_2_ [M + H]^+^ 271.1447, found 271.1445; HPLC (Chiralcel OD-H, 85:15 *n*-hexane/isopropanol, flow rate of 1 mL/min, λ = 254 nm) *t*_R1_ = 11.17 min (minor), *t*_R2_ = 12.18 min (major).

*Ethyl 4b,9-Dihydro-8aH-fluorene-8a-carboxylate* (**2p**). Using the general procedure detailed above diene iodide **S1g** (0.199 g, 0.543 mmol, 1.0 equiv) in DMF (6.5 mL, 0.08 M) was subjected to the Heck reaction conditions with 10 mol % NiI_2_/15 mol % 2,2′-bipyridine for 3 h. The crude product was purified by column chromatography (silica, 4:1 hexanes/EtOAc) to afford **2p** (0.0947 g, 0.394 mmol) in 73% yield as a clear colorless oil. Spectral data were in accordance with the literature:^86^^1^H NMR (400 MHz, CDCl_3_) δ 7.24–7.15 (m, 4H), 6.07–5.98 (m, 1H), 5.95–5.86 (m, 2H), 5.80–5.72 (m, 1H), 4.48 (d, *J* = 4.9 Hz, 1H), 4.18 (q, *J* = 7.1 Hz, 2H), 3.58 (d, *J* = 15.9 Hz, 1H), 3.15 (d, *J* = 15.9 Hz, 1H), 1.26 (t, *J* = 7.1 Hz, 4H); ^13^C{^1^H} NMR (101 MHz, CDCl_3_) δ_u_ 128.2, 127.1, 127.0, 126.8, 124.0, 123.7, 123.2, 121.1, 46.2, 14.2; δ_d_ 175.2, 144.3, 139.8, 61.2, 46.0; GC (method B) *t*_R_ = 1.676 min; EI-MS *m*/*z* (%) 240.1 (M+, 12), 167.1 (100), 152.0 (13), 139.0 (4), 115.0 (4).

### MOM (methoxymethyl) Group Deprotection

#### General Procedure A

A flame-dried round-bottom flask with a stir bar, under argon, was charged with chlorotrimethylsilane (0.45 mmol, 4.5 equiv) dissolved in CH_3_CN (1.7 mL, 0.06 M). After NaI (0.45 mmol, 4.5 equiv) was added, the resulting heterogeneous solution was stirred for 15 min at room temperature. In a second round-bottom flask, the MOM-protected Heck product (0.1 mmol, 1.0 equiv) was dissolved in CH_3_CN (1.7 mL, 0.06 M) and cooled to 0 °C. The TMS-Cl/NaI solution was added to the Heck product by syringe. The resulting mixture was stirred at 0 °C for 1 h. The reaction progress was monitored by TLC and GC-MS. Upon completion, the reaction was quenched with 1 M NaOH (17 mL/mmol), and the mixture stirred overnight. The aqueous layer was extracted thrice with Et_2_O. The combined organic layers were washed with brine, dried over MgSO_4_, and concentrated under reduced pressure. The crude products were purified by column chromatography.

#### General Procedure B

A flame-dried round-bottom flask with a stir bar, under argon, was charged with the MOM-protected Heck product (0.1 mmol, 1.0 equiv) dissolved in DCM (5 mL, 0.02 M). After the solution was cooled to −78 °C, BCl_3_ (1.0 M in DCM, 1 mmol, 10 equiv) was added, and the reaction mixture was stirred for 2–3 h. The reaction progress was monitored by TLC and GC-MS. Upon starting material consumption, the reaction was quenched with saturated NaHCO_3_ (50 mL/mmol), and the mixture extracted thrice with DCM. The combined organic layers were washed with brine, dried over MgSO_4_, and concentrated under reduced pressure. The crude intermediate was placed in a vial and redissolved in THF (5 mL, 0.02 M). Then, 1 M NaOH (5 mL, 5 mmol, 50 equiv) was added, and the vial was sealed with a pressure relief cap; the contents were stirred at 65 °C in a pie reactor until the reaction was determined to be complete by GC-MS analysis (typically 1 h). Upon completion of the reaction, the mixture was extracted thrice with Et_2_O. The combined organic layers were washed with brine, dried over MgSO_4_, and concentrated under reduced pressure. The crude products were purified by column chromatography.

*(6aR,10aR)-6a-Ethyl-6a,10a-dihydrophenanthridin-6(5H)-one* (**3c**). Using general MOM group deprotection procedure A with MOM-protected Heck product **2c** (0.0368 g, 0.137 mmol, 1.0 equiv) in CH_3_CN (4.7 mL, 0.03 M) afforded the crude deprotected product that was purified by column chromatography (silica, 4:1 hexanes/EtOAc) to give pure **3c** (0.0253 g, 0.112 mmol) in 82% yield as a white solid: mp 127.4–129.2 °C; ^1^H NMR (400 MHz, CDCl_3_) δ 8.22 (s, 1H), 7.17–7.10 (m, 2H), 6.97 (td, *J* = 7.4, 1.2 Hz, 1H), 6.67 (dd, *J* = 7.8, 1.4 Hz, 1H), 6.14–6.06 (m, 1H), 5.99–5.90 (m, 1H), 5.87–5.80 (m, 1H), 5.52–5.44 (m, 1H), 3.69–3.63 (m, 1H), 1.71–1.59 (m, 1H), 1.55–1.45 (m, 1H), 0.86 (t, *J* = 7.5 Hz, 3H); ^13^C{^1^H} NMR (101 MHz, CDCl_3_) δ_u_ 128.9, 128.8, 128.5, 127.8, 125.4, 125.0, 124.0, 115.0, 41.2, 9.2; δ_d_ 174.5, 135.9, 124.8, 45.2, 28.1; GC (method B) *t*_R_ = 2.352 min; EI-MS *m*/*z* (%) 224.1 (M – 1^+^,14), 196 (100), 178.0 (49), 167 (17), 152.0 (11), 139.0 (4), 115.0 (4), 77.0 (4); HRMS (ESI) calcd for C_15_H_16_ON [M + H]^+^ 226.1232, found 226.1232.

*(6aR,10aR)-6a-Isopropyl-6a,10a-dihydrophenanthridin-6(5H)-one* (**3e**). Using general MOM group deprotection procedure A with MOM-protected Heck product **2e** (0.100 g, 0.353 mmol, 1.0 equiv) in CH_3_CN (12 mL, 0.03 M) afforded the crude deprotected product that was purified by column chromatography (silica, 3:2 hexanes/EtOAc) to give pure **3e** (0.0681 g, 0.284 mmol) in 81% yield as a white solid: mp 169.9–172.4 °C; ^1^H NMR (400 MHz, CDCl_3_) δ 7.45 (s, 1H), 7.25–7.16 (m, 2H), 7.05 (td, *J* = 7.5, 1.2 Hz, 1H), 6.68 (dd, *J* = 7.9, 1.1 Hz, 1H), 6.22 (dd, *J* = 9.7, 5.0 Hz, 1H), 6.06–5.97 (m, 1H), 5.94 (d, *J* = 9.2 Hz, 1H), 5.54–5.47 (m, 1H), 3.89 (s, 1H), 1.84 (hept, *J* = 6.8 Hz, 1H), 1.01 (d, *J* = 6.8 Hz, 3H), 0.86 (s, 3H); ^13^C{^1^H} NMR (101 MHz, CDCl_3_) δ_u_ 129.3, 128.6, 127.8, 126.0, 125.9, 125.2, 123.8, 114.7, 41.3, 29.9, 18.5, 17.9; δ_d_ 173.6, 136.0, 125.3, 48.7; GC (method B) *t*_R_ = 2.523 min; EI-MS *m*/*z* (%) 239.1 (M^+^, 6), 196 (100), 178.0 (42), 167 (12), 152.0 (9), 139.0 (4); HRMS (ESI) calcd for C_16_H_18_ON [M + H]^+^ 240.1388, found 240.1383.

*(6aR,10aR)-6a-(Methoxymethyl)-6a,10a-dihydrophenanthridin-6(5H)-one* (**3f**). Using general MOM group deprotection procedure A with MOM-protected Heck product **2f** (0.0326 g, 0.114 mmol, 1.0 equiv) in CH_3_CN (3.9 mL, 0.03 M) afforded the crude deprotected product that was purified by column chromatography (silica, 4:1 hexanes/EtOAc) to give pure **3f** (0.0268 g, 0.111 mmol) in 97% yield as a clear colorless oil: ^1^H NMR (400 MHz, CDCl_3_) δ 8.05 (s, 1H), 7.16 (d, *J* = 7.5 Hz, 1H), 7.12 (td, *J* = 7.7, 1.5 Hz, 1H), 6.96 (td, *J* = 7.5, 1.2 Hz, 1H), 6.64 (dd, *J* = 7.8, 1.2 Hz, 1H), 6.06 (dd, *J* = 9.5, 5.1 Hz, 1H), 6.03–5.95 (m, 1H), 5.78 (dd, *J* = 9.4, 4.3 Hz, 1H), 5.68 (d, *J* = 9.4 Hz, 1H), 3.98 (dd, *J* = 4.6, 2.1 Hz, 1H), 3.55 (d, *J* = 8.9 Hz, 1H), 3.39 (d, *J* = 8.9 Hz, 1H), 3.27 (s, 3H); ^13^C{^1^H} NMR (101 MHz, CDCl_3_) δ_u_ 128.0, 127.7, 127.3, 126.2, 126.2, 124.5, 124.5, 123.6, 114.8, 59.5, 36.6; δ_d_ 171.8, 135.8, 124.5, 72.8, 47.3; GC (method B) *t*_R_ = 2.603 min; EI-MS *m*/*z* (%) 240.1 (M – 1^+^, 21), 210.0 (20), 196.0 (100), 177.9 (64), 167.0 (15), 152.0 (16), 138.9 (6), 76.9 (4); HRMS (ESI) calcd for C_15_H_16_NO_2_ [M + H]^+^ 242.1181, found 242.1174.

*(6aR,10aR)-2-Fluoro-6a-isopropyl-6a,10a-dihydrophenanthridin-6(5H)-one* (**3i**). Using general MOM group deprotection procedure A with MOM-protected Heck product **2i** (0.0793 g, 0.265 mmol, 1.0 equiv) in CH_3_CN (9 mL, 0.03 M) afforded the crude deprotected product that was purified by column chromatography (silica, 4:1 hexanes/EtOAc) to give pure **3i** (0.0355 g, 0.138 mmol) in 52% yield as a white solid: mp 178.0–180.9 °C; ^1^H NMR (400 MHz, CDCl_3_) δ 8.64 (s, 1H), 6.94–6.89 (m, 2H), 6.74–6.70 (m, 1H), 6.26–6.17 (m, 1H), 6.06–5.97 (m, 1H), 5.97–5.90 (m, 1H), 5.51–5.43 (m, 1H), 3.87–3.81 (m, 1H), 1.83 (hept, *J* = 6.9 Hz, 1H), 1.00 (d, *J* = 6.9 Hz, 3H), 0.87 (d, *J* = 6.8 Hz, 3H); ^13^C{^1^H} NMR (101 MHz, CDCl_3_) δ_u_ 128.4, 125.9 (d, ^3^*J*_C–F_ = 8.6 Hz), 125.4, 116.2, 116.1, 115.3 (d, ^2^*J*_C–F_ = 23.0 Hz), 114.4 (d, ^2^*J*_C–F_ = 22.8 Hz), 41.2, 30.0, 18.5, 17.9; δ_d_ 173.9, 159.0 (d, ^1^*J*_C–F_ = 242.2 Hz) 132.3, 126.9 (d, ^3^*J*_C–F_ = 7.3 Hz), 48.3; ^19^F NMR (376 MHz, CDCl_3_) δ −119.38; GC (method B) *t*_R_ = 2.527 min; EI-MS *m*/*z* (%) 257.1 (M^+^, 10), 214.0 (100), 196.0 (43), 185.0 (12), 169.9 (10); HRMS (ESI) calcd for C_16_H_17_ONF [M + H]^+^ 258.1294, found 258.1298.

*(6aR,10aR)-2-Chloro-6a-isopropyl-6a,10a-dihydrophenanthridin-6(5H)-oneone* (**3j**). Using general MOM group deprotection procedure A with MOM-protected Heck product **2j** (0.0161 g, 0.0507 mmol, 1.0 equiv) in CH_3_CN (1.7 mL, 0.03 M) afforded the crude deprotected product that was purified by column chromatography (silica, 4:1 hexanes/EtOAc) to give pure **3j** (0.0130 g, 0.0475 mmol) in 94% yield as a white solid: mp 182.5–184.4 °C; ^1^H NMR (400 MHz, CDCl_3_) δ 8.18 (s, 1H), 7.20–7.15 (m, 2H), 6.67 (d, *J* = 8.2 Hz, 1H), 6.22 (m, 1H), 6.07–5.98 (m, 1H), 5.93 (m, 1H), 5.51–5.43 (m, 1H), 3.87–3.82 (m, 1H), 1.81 (hept, *J* = 6.9 Hz, 1H), 1.00 (d, *J* = 6.9 Hz, 3H), 0.87 (d, *J* = 6.8 Hz, 3H); ^13^C{^1^H} NMR (101 MHz, CDCl_3_) δ_u_ 128.4, 128.4, 127.8, 127.0, 126.0, 125.8, 125.5, 116.1, 41.1, 30.0, 18.5, 17.9; δ_d_ 173.7, 134.8, 128.4, 127.0, 48.5; GC (method B) *t*_R_ = 3.215 min; EI-MS *m*/*z* (%) 273.0 (M^+^, 10), 230.0 (100), 211.9 (26), 194.9 (12), 177.0 (12), 167.0 (14), 151.9 (6), 138.9 (6); HRMS (ESI) calcd for C_16_H_17_ONCl [M + H]^+^ 274.0999, found 274.0995.

*6a-Isopropyl-2-methyl-6a,10a-dihydrophenanthridin-6(5H)-one* (**3k**). Using general MOM group deprotection procedure A with MOM-protected Heck product **2k** (0.100 g, 0.336 mmol, 1.0 equiv) in CH_3_CN (11.4 mL, 0.03 M) afforded the crude deprotected product that was purified by column chromatography (silica, 3:2 hexanes/EtOAc) to give pure **3k** (0.0724 g, 0.285 mmol) in 85% yield as a white solid: mp 193.3–194.4 °C; ^1^H NMR (400 MHz, CDCl_3_) δ 7.41 (s, 1H), 7.02–6.98 (m, 2H), 6.57 (d, *J* = 8.5 Hz, 1H), 6.21 (dd, *J* = 9.7, 5.1 Hz, 1H), 6.05–5.96 (m, 1H), 5.93 (d, *J* = 9.1 Hz, 1H), 5.50 (ddt, *J* = 9.3, 1.9, 0.9 Hz, 1H), 3.83 (s, 1H), 2.32 (s, 3H), 1.83 (hept, *J* = 6.9 Hz, 1H), 1.00 (d, *J* = 6.9 Hz, 3H), 0.87 (d, *J* = 6.8 Hz, 3H); ^13^C{^1^H} NMR (101 MHz, CDCl_3_) δ_u_ 129.4, 129.2, 128.3, 126.0, 125.9, 125.1, 114.6, 41.3, 29.9, 20.9, 18.5, 17.9; δ_d_ 173.5, 133.5, 133.3, 125.1, 48.7; GC (method B) *t*_R_ = 2.844 min; EI-MS *m*/*z* (%) 253.2 (M^+^, 18), 210.1 (100), 192.1 (46), 180.1 (10), 165.1 (15), 152.1 (10), 77.1 (4); HRMS (ESI) calcd for C_17_H_20_ON [M + H]^+^ 254.1545, found 254.1545.

*6a-Methyl-6a,9,10,10a-tetrahydrobenzo[c][1,5]naphthyridin-6(5H)-one* (**3m-2**). Using general MOM group deprotection procedure B with MOM-protected Heck product **2m-2** (0.0323, 0.125 mmol, 1.0 equiv) in CH_3_CN (4.3 mL, 0.03 M) afforded the crude deprotected product that was purified by column chromatography (silica, 3:2 hexanes/EtOAc) to give pure **3m-2** (0.0169 g, 0.0789 mmol) in 63% yield as a yellow solid: mp 134.9–137.4 °C; ^1^H NMR (400 MHz, CDCl_3_) δ 8.83 (s, 1H), 8.27 (dd, *J* = 3.8, 2.5 Hz, 1H), 7.15–7.12 (m, 2H), 5.95–5.85 (m, 2H), 2.92 (dd, *J* = 12.7, 3.1 Hz, 1H), 2.20–2.15 (m, 1H), 1.94–1.83 (m, 1H), 1.80–1.69 (m, 2H), 1.31 (s, 1H), 1.30 (s, 3H); ^13^C{^1^H} NMR (101 MHz, CDCl_3_) δ_u_ 144.3, 129.2, 128.3, 122.4, 121.9, 47.0, 42.3, 25.2; δ_d_ 175.6, 147.6, 131.5, 42.3, 25.7, 24.5; GC (method B) *t*_R_ = 2.294 min; EI-MS *m*/*z* (%) 214.0 (M^+^, 62), 199.0 (100), 185.0 (13), 171.0 (13), 161.0 (14), 156.0 (6), 131.0 (4), 91.0 (4), 77.0 (4); HRMS (ESI) calcd for C_13_H_15_ON_2_ [M + H]^+^ 215.1184, found 215.1182.

*(6aR,10aR)-6a-Ethyl-6a,10a-dihydrobenzo[c][1,8]naphthyridin-6(5H)-one* (**3o**). Using general MOM group deprotection procedure B with MOM-protected Heck product **2o** (0.0156, 0.0577 mmol, 1.0 equiv) in CH_3_CN (1.9 mL, 0.03 M) afforded the crude deprotected product that was purified by column chromatography (silica, 3:2 hexanes/EtOAc) to give pure **3o** (0.0102 g, 0.0451 mmol) in 79% yield as a white solid: mp 163.9–165.5 °C; ^1^H NMR (400 MHz, CDCl_3_) δ 9.08 (s, 1H), 8.26 (dd, *J* = 5.0, 1.7 Hz, 1H), 7.60–7.54 (m, 1H), 7.00 (dd, *J* = 7.4, 5.0 Hz, 1H), 6.16 (m, 1H), 6.06 (m, 1H), 5.91–5.87 (m, 1H), 5.55 (m,1H), 3.77–3.71 (m, 1H), 1.70–1.61 (m, 2H), 0.95 (t, *J* = 7.5 Hz, 3H); ^13^C{^1^H} NMR (101 MHz, CDCl_3_) δ_u_ 146.8, 136.8, 128.9, 127.2, 125.6, 125.3, 119.2, 39.8, 9.2; δ_d_ 174.3, 149.7, 120.2, 45.6, 28.2; GC (method B) *t*_R_ = 2.237 min; EI-MS *m*/*z* (%) 225.0 (M – 1^+^, 20), 211.0 (4), 197.0 (100), 179.0 (6), 169.0 (11), 154.0 (15), 127.0 (8), 115.0 (4), 77.0 (4); HRMS (ESI) calcd for C_13_H_15_ON_2_ [M + H]^+^ 227.1184, found 227.1183.

### Synthesis of Chiral *t*Bu-*i*Quinox Ligands (**L26** and **L27**)

#### General Procedure for the Synthesis of Isoquinoline *N*-Oxides

In a round-bottom flask with a stir bar, isoquinoline (1.0 mmol, 1.0 equiv) was dissolved in DCM (0.65 mL, 10.8 M) and the mixture cooled to 0 °C. *m*-Chloroperbenzoic acid (mCPBA, 2.0 mmol, 2.0 equiv) was dissolved in DCM (0.65 mL, 10.8 M), and the mixture added dropwise to the reaction mixture. The mixture was allowed to warm to room temperature and react overnight. The reaction mixture was diluted with DCM, washed with 3 N NaOH and brine, and then dried with MgSO_4_. The crude product was concentrated under vacuum and purified by column chromatography.

*6-Fluoroisoquinoline 2-Oxide* (**4a**). Using the general procedure for the synthesis of isoquinoline *N*-oxides, 6-fluoroisoquinoline (0.300 g, 2.04 mmol, 1.0 equiv) in DCM (1.3 mL, 10.8 M) reacted with mCPBA (1.01 g, 4.08 mmol, 2.0 equiv) in DCM (2.7 mL, 10.8 M) to afford the crude product that was purified by column chromatography (silica, 10:1 EtOAc/MeOH) to give pure **4a** (0.288 g, 1.76 mmol) in 86% yield as a tan solid: mp 225.1–225.6 °C; ^1^H NMR (400 MHz, CDCl_3_) δ 8.76 (d, *J* = 4.0 Hz, 1H), 8.20–8.13 (m, 1H), 7.75 (dd, *J* = 9.0, 5.2 Hz, 1H), 7.64 (d, *J* = 7.2 Hz, 1H), 7.48–7.37 (m, 2H); ^13^C{^1^H} NMR (101 MHz, CDCl_3_) δ_u_ 137.8, 136.1, 127.7 (d, ^3^*J*_C–F_ = 9.2 Hz), 123.6 (d, ^4^*J*_C–F_ = 5.5 Hz), 120.2 (d, ^2^*J*_C–F_ = 25.7 Hz), 110.9 (d, ^2^*J*_C–F_ = 22.1 Hz); δ_d_ 162.3 (d, ^1^*J*_C–F_ = 253.1 Hz), 130.0 (d, ^3^*J*_C–F_ = 9.9 Hz), 126.7; ^19^F NMR (376 MHz, CDCl_3_) δ −108.08; HRMS (ESI) calcd for C_9_H_7_ONF [M + H]^+^ 164.0512, found 164.0504.

*6-Methylisoquinoline 2-Oxide* (**4b**). Using the general procedure for the synthesis of isoquinoline *N*-oxides, 6-methylisoquinoline (0.200 g, 1.40 mmol, 1.0 equiv) in DCM (0.9 mL, 10.8 M) reacted with mCPBA (0.688 g, 2.79 mmol, 2.0 equiv) in DCM (1.8 mL, 10.8 M) to afford the crude product that was purified by column chromatography (silica, 10:1 EtOAc/MeOH) to give pure **4b** (0.180 g, 1.13 mmol) in 81% yield as a white solid: mp 135.5–135.8 °C; ^1^H NMR (400 MHz, CDCl_3_) δ 8.72 (d, *J* = 1.8 Hz, 1H), 8.11 (dd, *J* = 7.1, 1.8 Hz, 1H), 7.63 (d, *J* = 8.5 Hz, 1H), 7.61–7.54 (m, 2H), 7.46 (dd, *J* = 8.4, 1.6 Hz, 1H), 2.52 (s, 3H); ^13^C{^1^H} NMR (101 MHz, CDCl_3_) δ_u_ 136.7, 136.0, 131.8, 125.8, 124.8, 123.6; 21.8; δ_d_ 139.6, 129.1, 127.7; HRMS (ESI) calcd for C_10_H_10_ON [M + H]^+^ 160.0762, found 160.0757.

#### General Procedure for the Cyanation of Isoquinoline *N*-Oxides

A vial with a stir bar was either flame-dried under argon or oven-dried and charged with isoquinoline *N*-oxide (1.0 mmol, 1.0 equiv) and trimethylsilyl cyanide (2.2 mmol, 2.2 equiv). The vial was sealed with a pressure relief cap, and its contents were stirred at 130 °C in a pie reactor for 1 h. Upon completion of the reaction, the crude reaction mixture was concentrated under vacuum and purified by column chromatography.

*6-Fluoroisoquinoline-1-carbonitrile* (**5a**). Using the general procedure for cyanation of isoquinoline *N*-oxides, *N*-oxide **4a** (0.412 g, 2.53 mmol, 1.0 equiv) reacted with trimethylsilyl cyanide (0.741 mL, 5.56 mmol, 2.2 equiv) to afford the crude product that was purified by column chromatography (silica, 10:1 hexanes/EtOAc) to give pure **5a** (0.410 g, 2.38 mmol) in 94% yield as a white solid: mp 123.3–124.0 °C; ^1^H NMR (400 MHz, CDCl_3_) δ 8.50 (dd, *J* = 5.7, 0.8 Hz, 1H), 8.24 (ddt, *J* = 8.8, 5.2, 0.9 Hz, 1H), 7.71 (dd, *J* = 5.7, 0.9 Hz, 1H), 7.47–7.37 (m, 2H); ^13^C{^1^H} NMR (101 MHz, CDCl_3_) δ_u_ 144.1, 128.8 (d, ^3^*J*_C–F_ = 9.9 Hz), 123.9 (d, ^4^*J*_C–F_ = 5.6 Hz), 120.8 (d, ^2^*J*_C–F_ = 26.2 Hz), 110.8 (d, ^2^*J*_C–F_ = 21.4 Hz); δ_d_ 163.8 (d, ^1^*J*_C–F_ = 256.7 Hz), 137.5 (d, ^3^*J*_C–F_ = 10.7 Hz), 126.6, 115.6; ^19^F NMR (376 MHz, CDCl_3_) δ −103.47; GC (method A) *t*_R_ = 8.084 min; EI-MS *m*/*z* (%) 171.9 (M^+^, 100), 144.9 (36), 177.8 (4), 99.8 (4), 93.9 (4), 85.8 (4), 73.9 (3); HRMS (ESI) calcd for C_10_H_6_N_2_F [M + H]^+^ 173.0515, found 173.0509.

*6-Methylisoquinoline-1-carbonitrile* (**5b**). Using the general procedure for cyanation of isoquinoline *N*-oxides, *N*-oxide **4b** (0.159 g, 1.00 mmol, 1.0 equiv) reacted with trimethylsilyl cyanide (0.744 mL, 2.20 mmol, 2.2 equiv) to afford the crude product that was purified by column chromatography (silica, 10:1 hexanes/EtOAc) to give pure **5b** (0.155 g, 0.922 mmol) in 92% yield as a white solid: mp 103.9–104.4 °C; ^1^H NMR (400 MHz, CDCl_3_) δ 8.60 (d, *J* = 5.6 Hz, 1H), 8.24 (d, *J* = 8.6 Hz, 1H), 7.80 (dd, *J* = 5.7, 0.9 Hz, 1H), 7.70 (s, 1H), 7.63 (dd, *J* = 8.5, 1.6 Hz, 1H), 2.60 (s, 3H); ^13^C{^1^H} NMR (101 MHz, CDCl_3_) δ_u_ 143.4, 132.2, 126.1, 125.1, 123.8, 22.1; δ_d_ 142.6, 136.2, 134.4, 128.0, 116.0; GC (method B) *t*_R_ = 1.413 min; EI-MS *m*/*z* (%) 168.0 (M^+^, 100), 140.0 (17), 114.0 (9), 63.0 (4); HRMS (ESI) calcd for C_11_H_9_N_2_ [M + H]^+^ 169.0766, found 169.0759.

#### General Procedure for the Synthesis of (*S*)-4-(*tert*-Butyl)-2-(isoquinol-1-yl)-4,5-dihydrooxazole [(*S*)-*tert*-butyl *i*Quinox]

A two-necked round-bottom flask with a stir bar was equipped with a reflux condenser and charged with zinc triflate (0.2 mmol, 20 mol %). The flask was placed under high vacuum and heated to 90 °C for 1 h using a mineral oil bath. In a separate flame-dried round-bottom flask with a stir bar, under argon, isoquinoline-1-carbonitrile (1.0 mmol, 1.0 equiv) and (*S*)-*tert*-leucinol (1.0 mmol, 1.0 equiv) were dissolved in toluene (4 mL, 0.25 M). After the flask had been cooled with zinc triflate to room temperature, the mixture was added to it, and the new mixture refluxed under argon for 15–24 h. Once the reaction had reached completion, as judged by GC-MS or TLC analysis, the reaction mixture was allowed to cool to room temperature, diluted with EtOAc, washed with saturated NaHCO_3_ and brine, and then dried with MgSO_4_. The crude product was concentrated under vacuum and purified by column chromatography.

*(S)-4-(tert-Butyl)-2-(6-fluoroisoquinolin-1-yl)-4,5-dihydrooxazole* (*t*Bu-^6^F*i*Quinox, **L26**). Using the general procedure for the synthesis of (*S*)-*tert*-butyl *i*Quinox, **5a** (0.154 g, 0.893 mmol, 1.0 equiv) reacted with (*S*)-*tert*-leucinol (0.105 g, 0.893 mmol, 1.0 equiv) and zinc triflate (0.065 g, 0.179 mmol, 20 mol %) in toluene (3.6 mL, 0.25 M) to afford the crude product that was purified column chromatography (silica, 3:2 hexanes/EtOAc) to give pure **L26** (0.154 g, 0.566 mmol) in 63% yield as a white solid: mp 86.9–87.2 °C; [α]_D_^20^ = −41 (*c* 0.40, CHCl_3_); ^1^H NMR (400 MHz, CDCl_3_) δ 9.41–9.32 (m, 1H), 8.54 (d, *J* = 5.6 Hz, 1H), 7.63 (d, *J* = 5.6 Hz, 1H), 7.41–7.31 (m, 2H), 4.48–4.36 (m, 1H), 4.30–4.18 (m, 2H), 0.98 (s, 9H); ^13^C{^1^H} NMR (101 MHz, CDCl_3_) δ_u_ 142.6, 131.2 (d, ^3^*J*_C–F_ = 9.4 Hz), 122.8 (d, ^4^*J*_C–F_ = 5.2 Hz), 119.0 (d, ^2^*J*_C–F_ = 24.7 Hz), 110.2 (d, ^2^*J*_C–F_ = 20.8 Hz), 77.6, 26.1; δ_d_ 163.0 (d, ^1^*J*_C–F_ = 254.1 Hz), 161.6, 146.3 (d, ^4^*J*_C–F_ = 1.6 Hz), 138.5 (d, ^3^*J*_C–F_ = 10.4 Hz), 124.7, 68.2, 34.1; ^19^F NMR (376 MHz, CDCl_3_) δ −107.09; GC (method B) *t*_R_ = 2.604 min; EI-MS *m*/*z* (%) 272.0 (M^+^, 12), 257.0 (3), 216.0 (100), 186.0 (61), 172.9 (17), 159.9 (49), 145.9 (81), 132.9 (18), 126.0 (17), 118.9 (8), 98.9 (6), 57.0 (12); HRMS (ESI) calcd for C_16_H_18_ON_2_F [M + H]^+^ 273.1403, found 273.1397.

*(S)-4-(tert-Butyl)-2-(6-methylisoquinolin-1-yl)-4,5-dihydrooxazole* (*t*Bu-^6^CH_3_*i*Quinox, **L27**). Using the general procedure for the synthesis of (*S*)-*tert*-butyl *i*Quinox, **5b** (0.124 g, 0.737 mmol, 1.0 equiv) reacted with (*S*)-*tert*-leucinol (0.0860 g, 0.737 mmol, 1.0 equiv) and zinc triflate (0.054 g, 0.147 mmol, 20 mol %) in toluene (2.9 mL, 0.25 M) to afford the crude product that was purified column chromatography (silica, 3:2 hexanes/EtOAc) to give pure **L27** (0.168 g, 0.626 mmol) in 85% yield as a yellow oil: [α]_D_^20^ = −32 (*c* 0.34, CHCl_3_); ^1^H NMR (400 MHz, CDCl_3_) δ 9.19 (d, *J* = 8.8 Hz, 1H), 8.58 (d, *J* = 5.6 Hz, 1H), 7.66 (d, *J* = 5.6 Hz, 1H), 7.62 (s, 1H), 7.51 (dd, *J* = 8.8, 1.8 Hz, 1H), 4.55–4.43 (m, 1H), 4.37–4.25 (m, 2H), 2.55 (s, 3H), 1.06 (s, 9H); ^13^C{^1^H} NMR (101 MHz, CDCl_3_) δ_u_ 141.9, 130.8, 127.2, 125.9, 122.8, 77.5, 26.1, 21.9; δ_d_ 161.9, 146.1, 140.7, 137.1, 125.9, 68.1, 34.1; GC (method B) *t*_R_ = 3.187 min; EI-MS *m*/*z* (%) 268.1 (M^+^, 28), 253.1 (4), 211.1 (100), 197.0 (4), 183.1 (58), 169.0 (13), 156.0 (48), 142.0 (50), 129.0 (12), 115 (17), 89.0 (4), 57.1 (5); HRMS (ESI) calcd for C_17_H_21_ON_2_ [M + H]^+^ 269.1654, found 269.1646.

#### Control Experiment in the Absence of Mn

An oven-dried screw-cap vial, equipped with a magnetic stir bar, was charged with the aryl iodide diene **1e-I** (0.020 g, 0.049 mmol, 1.0 equiv) and then placed in a glovebox. Anhydrous NiI_2_ (0.0015 g, 0.0049 mmol, 10 mol %), bipy (0.0011 g, 0.0074 mmol, 15 mol %), KI (0.0081 g, 0.049 mmol, 1.0 equiv), 2-cyclohexenone (14 μL, 0.0141 g, 0.147 mmol, 3.0 equiv), and DMF (0.6 mL, 0.08 M) were added, and the vial was sealed with a pressure relief cap and removed from the glovebox. The contents of the vial were stirred at 80 °C in a pie reactor for 15 h, filtered through an aluminum oxide plug, diluted with EtOAc, and analyzed by TLC/GC-MS. However, the formation of desired product **2e** was not observed, and only starting material **1e-I** (>99% as determined by GC) was present in the reaction mixture: GC (method B) *t*_R_ = 3.438 min; EI-MS *m*/*z* (%) 411.1 (M^+^, 1), 379.1 (4), 292.0 (8), 260.1 (10), 252.1 (8), 230.9 (100), 202.9 (5), 164.0 (34), 121.1 (10), 105.0 (50), 90.0 (7), 79.0 (26), 51.0 (4).

#### Control Experiment Using Ni(COD)_2_ in the Absence of Mn

An oven-dried screw-cap vial, equipped with a magnetic stir bar, was charged with aryl iodide diene **1e-I** (0.020 g, 0.049 mmol, 1.0 equiv) and then placed in a glovebox. Anhydrous Ni(COD)_2_ (0.0013 g, 0.0049 mmol, 10 mol %), bipy (0.0011 g, 0.0074 mmol, 15 mol %), KI (0.0081 g, 0.049 mmol, 1.0 equiv), 2-cyclohexenone (14 μL, 0.0141 g, 0.147 mmol, 3.0 equiv), and DMF (0.6 mL, 0.08 M) were added, and the vial was sealed with a pressure relief cap and removed from the glovebox. The contents of the vial were stirred at 80 °C in a pie reactor for 15 h, filtered through an aluminum oxide plug, diluted with EtOAc, and analyzed by TLC/GC-MS. The reaction mixture contained the desired product **2e** (8% as determined by GC) and starting material **1e-I** (>90% as determined by GC). **2e**: GC (method B) *t*_R_ = 2.546 min; EI-MS *m*/*z* (%) 283.1 (M^+^, 17), 251.0 (12), 240.0 (15), 224 (10), 208.0 (100), 196.0 (99), 178 (67), 167 (10), 152.0 (12), 139 (4), 115.0 (4), 105.0 (4), 77.0 (6). **1e-I**: GC (method B) *t*_R_ = 3.439 min; EI-MS *m*/*z* (%) 411.1 (M^+^, 1), 379.1 (4), 292.0 (8), 260.1 (10), 252.1 (8), 230.9 (100), 202.9 (5), 164.0 (34), 121.1 (10), 105.0 (50), 90.0 (7), 79.0 (26), 51.0 (4).

#### Control Experiment Using Ni(COD)_2_ in the Presence of Mn

An oven-dried screw-cap vial, equipped with a magnetic stir bar, was charged with aryl iodide diene **1e-I** (0.020 g, 0.049 mmol, 1.0 equiv) and then placed in a glovebox. Anhydrous Ni(COD)_2_ (0.0013 g, 0.0049 mmol, 10 mol %), bipy (0.0011 g, 0.0074 mmol, 15 mol %), Mn (0.0080 g, 0.147 mmol, 3.0 equiv), KI (0.0081 g, 0.049 mmol, 1.0 equiv), 2-cyclohexenone (14 μL, 0.0141 g, 0.147 mmol, 3.0 equiv), and DMF (0.6 mL, 0.08 M) were added, and the vial was sealed with a pressure relief cap and removed from the glovebox. The contents of the vial were stirred at 80 °C in a pie reactor for 15 h. After the reaction had reached completion, the reaction mixture was filtered through an aluminum oxide plug, diluted with EtOAc, and analyzed by TLC/GC-MS. The reaction mixture contained the desired product **2e** (78% as determined by GC): GC (method B) *t*_R_ = 2.546 min; EI-MS *m*/*z* (%) 283.1 (M^+^, 17), 251.0 (12), 240.0 (15), 224 (10), 208.0 (100), 196.0 (99), 178 (67), 167 (10), 152.0 (12), 139 (4), 115.0 (4), 105.0 (4), 77.0 (6).

#### Control Experiment Using D_2_O and Mn with the Aryl Bromide Diene

An oven-dried screw-cap vial, equipped with a magnetic stir bar, was charged with aryl bromide diene **1e** (0.020 g, 0.055 mmol, 1.0 equiv) and then placed in a glovebox. Mn (0.0091 g, 0.165 mmol, 3.0 equiv), KI (0.0091 g, 0.055 mmol, 1.0 equiv), and DMF (0.66 mL, 0.08 M) were added, and the vial was sealed with a pressure relief cap and removed from the glovebox. The contents of the vial were stirred at 80 °C. After 3 h, D_2_O (0.01 mL, 0.55 mmol, 10.0 equiv) was added and the mixture was heated at 80 °C for additional 18 h. The reaction mixture was filtered through an aluminum oxide plug, diluted with EtOAc, and analyzed by TLC/GC-MS. The reaction mixture contained only the starting material **1e** (>99% as determined by GC): GC (method B) *t*_R_ = 3.032 min; EI-MS *m*/*z* (%) 363.1 (M – 1^+^, 1), 331.1 (7), 243.9 (10), 227.9 (4), 213.9 (15), 183.0 (68), 164.1 (64), 121.1 (21), 105.1 (100), 91.0 (12), 79.1 (47), 51.1 (5).

#### Control Experiment Using D_2_O and Mn with the Aryl Iodide Diene

An oven-dried screw-cap vial, equipped with a magnetic stir bar, was charged with aryl bromide diene **1e-I** (0.020 g, 0.049 mmol, 1.0 equiv) and then placed in a glovebox. Mn (0.0080 g, 0.147 mmol, 3.0 equiv), KI (0.0081 g, 0.049 mmol, 1.0 equiv), and DMF (0.6 mL, 0.08 M) were added, and the vial was sealed with a pressure relief cap and removed from the glovebox. The contents of the vial were stirred at 80 °C. After 3 h, D_2_O (0.01 mL, 0.49 mmol, 10.0 equiv) was added and the mixture was heated at 80 °C for additional 18 h. The reaction mixture was filtered through an aluminum oxide plug, diluted with EtOAc, and analyzed by TLC/GC-MS. The reaction mixture contained deuterated dehalogenated side product-D_1_**2e-1** (13% as determined by GC) and starting material **1e-I** (87% as determined by GC): **2e-1**: GC (method B) *t*_R_ = 2.323 min; EI-MS *m*/*z* (%) 254.0 (M – 32^+^, 21), 211.0 (5), 166.0 (30), 150.9 (23), 135.0 (5), 121.0 (20), 106.0 (100), 91.0 (6), 79.0 (36). 51.0 (5). **1e-I**: GC (method B) *t*_R_ = 3.439 min; EI-MS *m*/*z* (%) 379.1 (M – 32^+^, 4), 291.9 (7), 259.9 (12) 252.1 (0), 244.8 (4), 230.9 (100), 202.9 (4), 164.0 (33), 121.1 (12), 105.0 (50), 79.0 (26), 51.0 (4).

#### Control Experiment Using D_2_O and Zn with the Aryl Bromide Diene

An oven-dried screw-cap vial, equipped with a magnetic stir bar, was charged with aryl bromide diene **1d** (0.025 g, 0.075 mmol, 1.0 equiv) and then placed in a glovebox. Zn (0.0146 g, 0.224 mmol, 3.0 equiv), KI (0.0124 g, 0.075 mmol, 1.0 equiv), and DMF (0.9 mL, 0.08 M) were added, and the vial was sealed with a pressure relief cap and removed from the glovebox. The contents of the vial were stirred at 80 °C. After 3 h, D_2_O (0.014 mL, 0.75 mmol, 10 equiv) was added and the mixture was heated at 80 °C for additional 18 h. The reaction mixture was filtered through an aluminum oxide plug, diluted with EtOAc, and analyzed by TLC/GC-MS. The reaction mixture contained only starting material **1d** (>99% as determined by GC): GC (method B) *t*_R_ = 2.677 min; EI-MS *m*/*z* (%) 333.1 (M – 1^+^, 3), 290.0 (23), 214.0 (63), 197 (5), 185.0 (17), 134.0 (100), 121.1 (10), 105.0 (74), 91.0 (9), 77.1 (39), 51.0 (6).

#### Control Experiment Using D_2_O and Zn with the Aryl Iodide Diene

An oven-dried screw-cap vial, equipped with a magnetic stir bar, was charged with aryl iodide diene **1d-I** (0.025 g, 0.066 mmol, 1.0 equiv) and then placed in a glovebox. Zn (0.0129 g, 0.197 mmol, 3.0 equiv), KI (0.0111 g, 0.066 mmol, 1.0 equiv), and DMF (0.8 mL, 0.08 M) were added, and the vial was sealed with a pressure relief cap and removed from the glovebox. The contents of the vial were stirred at 80 °C. After 3 h, D_2_O (0.012 mL, 0.66 mmol, 10 equiv) was added and the mixture was heated at 80 °C for additional 18 h. The reaction mixture was filtered through an aluminum oxide plug, diluted with EtOAc, and analyzed by TLC/GC-MS. The reaction mixture contained deuterated dehalogenated side product-D_1_**2d-1** (82% as determined by GC). The organic layer was washed twice with 1 N HCl and once withbrine, dried with MgSO_4_, and filtered and concentrated *in vacuo.* The crude residue was purified by column chromatography (silica, 4:1 hexanes/EtOAc) to afford **2d-1** in 65% yield (0.011 g, 0.043 mmol) as a yellow oil: ^1^H NMR (400 MHz, CDCl_3_) δ 7.28–7.20 (m, 3H), 7.15–7.09 (m, 1H), 5.35 (s, 4H), 3.23 (s, 3H), 2.49 (hept, *J* = 6.9 Hz, 1H), 2.37–2.07 (m, 2H), 0.76 (d, *J* = 6.9 Hz, 6H); ^13^C{^1^H} NMR (101 MHz, CDCl_3_) δ_u_ 128.8, 128.7, 128.4, 127.9, 127.0, 124.1, 40.9, 35.9, 17.5; δ_d_ 174.9, 145.0, 128.1, 53.9, 26.3; GC (mthod B) *t*_R_ = 1.951 min; EI-MS *m*/*z* (%) 256.0 (M^+^, 6), 213.0 (28), 136.0 (100), 121.0 (14), 105.0 (54), 95.0 (10), 79.0 (45), 51.0 (6).

#### Control Experiment Using 2-Cyclohexenone and Mn

An oven-dried screw-cap vial, equipped with a magnetic stir bar, was charged with 2-cyclohexenone (50 μL, 0.05 g, 0.52 mmol, 1.0 equiv), Mn (0.057 g, 1.04 mmol, 2.0 equiv), and DMF (2 mL, 0.26 M) in a glovebox. The vial was sealed with a pressure relief cap, removed from the glovebox, and stirred at 80 °C for 15 h. The reaction mixture was filtered through an aluminum oxide plug, diluted with EtOAc, and analyzed by TLC/GC-MS. The reaction mixture contained only starting material 2-cyclohexenone (>99% as determined by GC): GC (method B) *t*_R_ = 1.970 min; EI-MS *m*/*z* (%) 96.0 (M^+^, 41), 73.0 (4), 68.0 (100), 55.0 (5).

#### Control Experiment Using TEMPO

An oven-dried screw-cap vial, equipped with a magnetic stir bar, was charged with aryl iodide diene **1e-I** (0.020 g, 0.049 mmol, 1.0 equiv) and then placed in a glovebox. Anhydrous NiI_2_ (0.0015 g, 0.0049 mmol, 10 mol %), bipy (0.0011 g, 0.0074 mmol, 15 mol %), Mn (0.0080 g, 0.147 mmol, 3.0 equiv), KI (0.0081 g, 0.049 mmol, 1.0 equiv), 2-cyclohexenone (14 μL, 0.0141 g, 0.147 mmol, 3.0 equiv), TEMPO (0.0077 g, 0.049 mmol, 1.0 equiv), and DMF (0.6 mL, 0.08 M) were added. The vial was sealed with a pressure relief cap and removed from the glovebox. The contents of the vial were stirred at 80 °C in a pie reactor for 15 h. After the reaction had reached completion, the reaction mixture was filtered through an aluminum oxide plug, diluted with EtOAc, and analyzed by TLC/GC-MS. The reaction mixture contained desired product **2e** (94% as determined by GC): GC (method B) *t*_R_ = 2.546 min; EI-MS *m*/*z* (%) 283.1 (M^+^, 17), 251.0 (12), 240.0 (15), 224 (10), 208.0 (100), 196.0 (99), 178 (67), 167 (10), 152.0 (12), 139 (4), 115.0 (4), 105.0 (4), 77.0 (6).

## Data Availability

The data underlying this study are available in the published article and its Supporting Information.

## Supporting Information Available

NMR spectra for all new compounds, chiral LC traces for enantioselective Mizoroki–Heck reactions, a table of amide formation (SI-Table 1), secondary amide protection (SI-Table 2), chiral ligand screening (SI-Table 3), MOM group deprotection (SI-Table 4), a scheme for the synthesis of chiral *t*Bu-*i*Quinox ligands (SI-Scheme 1), GC-MS and NMR data for mechanistic control studies, computational data, including conformation images (SI-Figure 1), and calculation details (SI-Table 5) (PDF)
FAIR data, including the primary NMR FID files, for compounds **1a**, **1a-I**, **S2b**, **S2b-I**, **S2c-I**, **1d**, **1d-I**, **S2e**, **S2e-I**, **S2f-I**, **1g-I**, **S2h**, **S2h-I**, **S2i**, **S2i-I**, **S2j**, **S2j-I**, **S2k**, **S2l-I**, **S2m–o**, **S2o-I**, **1b**, **1b-I**, **1c-I**, **1e**, **1e-I**, **1f-I**, **1h**, **1h-I**, **1i**, **1i-I**, **1j**, **1j-I**, **1k**, **1l-I**, **1m–o**, **1o-I**, **2a–d**, **2d-1**, **2e–l**, **2m-2**, **2n–p**, **3c**, **3e**, **3f**, **3i–k**, **3m-2**, **3o**, **4a**, **4b**, **5a**, **5b**, **L26**, and **L27** (ZIP)
