# Supplementary material for: Ni-Catalyzed Enantioselective Intramolecular Mizoroki–Heck Reaction for the Synthesis of Phenanthridinone Derivatives
Source: J Org Chem. 2023 Jun 15;88(13):8203–26. doi: 10.1021/acs.joc.3c00202 (PMC10337041; doi:10.1021/acs.joc.3c00202)
Supplement: Supplementary file 1 — jo3c00202_si_001.pdf [file jo3c00202_si_001.pdf]

Supporting Information

Ni-catalyzed Enantioselective Intramolecular Mizoroki-Heck Reaction for the Synthesis of Phenanthridinone Derivatives

Authors: Diana Rachii[a], Dana J. Caldwell[a], Yui Kosukegawa[a], Mary Sexton[a], Paul Rablen[b], William P. Malachowski*[a].

[a]*Bryn Mawr College, Chemistry Department, Bryn Mawr, Pennsylvania 19010*
[b]*Swarthmore College, Chemistry Department, Swarthmore, Pennsylvania 19081*

***Corresponding author e-mail:** wmalacho@brynmawr.edu

Table of Contents

SI-Table 1. Amide Formation. 3
NMR spectra for N-Me and N-H amide products 4
SI-Table 2: Secondary Amide Protection. 28
NMR spectra for N-methoxymethyl (MOM) amide products 29
SI-Table 3: Chiral Ligand Screening. 48
Chiral LC's, GCMS data, and NMR spectra for Mizoroki-Heck reaction products 49
SI-Table 4: MOM Group Deprotection. 84
NMR spectra for MOM group deprotection products 85
SI-Scheme 1. Synthesis of chiral tBu-iQuinox ligands. 93
NMR spectra for isoquinoline N-oxides 94
NMR spectra for isoquinoline carbonitrile products 96
GCMS and NMR data for Mechanistic Control Studies (Scheme 1). 102
Computational Data 122
SI-Figure 1. Conformation Images and Relative Energy. 122
SI-Table 5. Calculations. 123

SI-Table 1. Amide Formation.

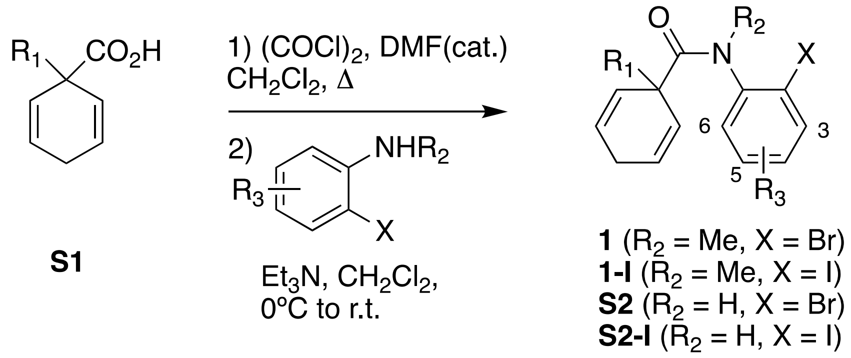

entry	R_1	R_2	R_3	X	yield (%)	compd
1	Me, **S1a**	Me	H	Br	93	**1a**
2	Me, **S1a**	Me	H	I	79	**1a-I**
3	Me, **S1a**	H	H	Br	95	**S2b**
4	Me, **S1a**	H	H	I	99	**S2b-I**
5	Et, **S1b**	H	H	I	95	**S2c-I**
6	*i*-Pr, **S1c**	Me	H	Br	90	**1d**
7	*i*-Pr, **S1c**	Me	H	I	84	**1d-I**
8	*i*-Pr, **S1c**	H	H	Br	79	**S2e**
9	*i*-Pr, **S1c**	H	H	I	84	**S2e-I**
10	$-CH_2OCH_3$, **S1d**	H	H	I	97	**S2f-I**
11	$-CH_2CO_2Et$, **S1e**	Me	H	I	83	**1g-I**
12	- CH_2Ph, **S1f**	H	H	Br	88	**S2h**
13	$-CH_2Ph$, **S1f**	H	H	I	90	**S2h-I**
14	*i*-Pr, **S1c**	H	4-F	Br	90	**S2i**
15	*i*-Pr, **S1c**	H	4-F	I	91	**S2i-I**
16	*i*-Pr, **S1c**	H	4-Cl	Br	93	**S2j**
17	*i*-Pr, **S1c**	H	4-Cl	I	82	**S2j-I**
18	*i*-Pr, **S1c**	H	4-Me	Br	96	**S2k**
19	*i*-Pr, **S1c**	H	5-Me	I	99	**S2l-I**
20	Me, **S1a**	H	C_3=N	Br	85	**S2m**
21	Me, **S1a**	H	C_6=N	Br	81	**S2n**
22	Et, **S1b**	H	C_6=N	Br	78	**S2o**
23	Et, **S1b**	H	C_6=N	I	89	**S2o-I**

NMR spectra for N-Me and N-H amide products

N-(2-Bromophenyl)-N,1-dimethylcyclohexa-2,5-diene-1-carboxamide (1a).

^{1}H NMR (400 MHz), **1a**

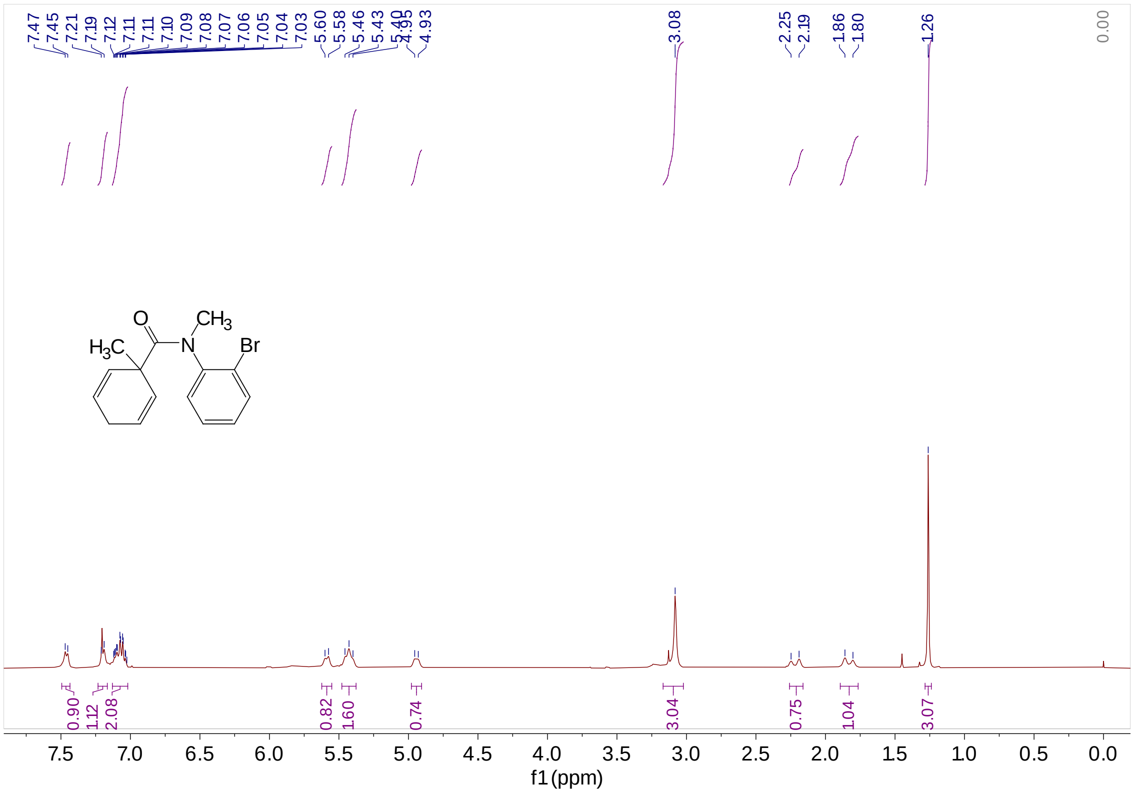

$^{13}C\{^1H\}$ NMR (101 MHz, $CDCl_3$), **1a**

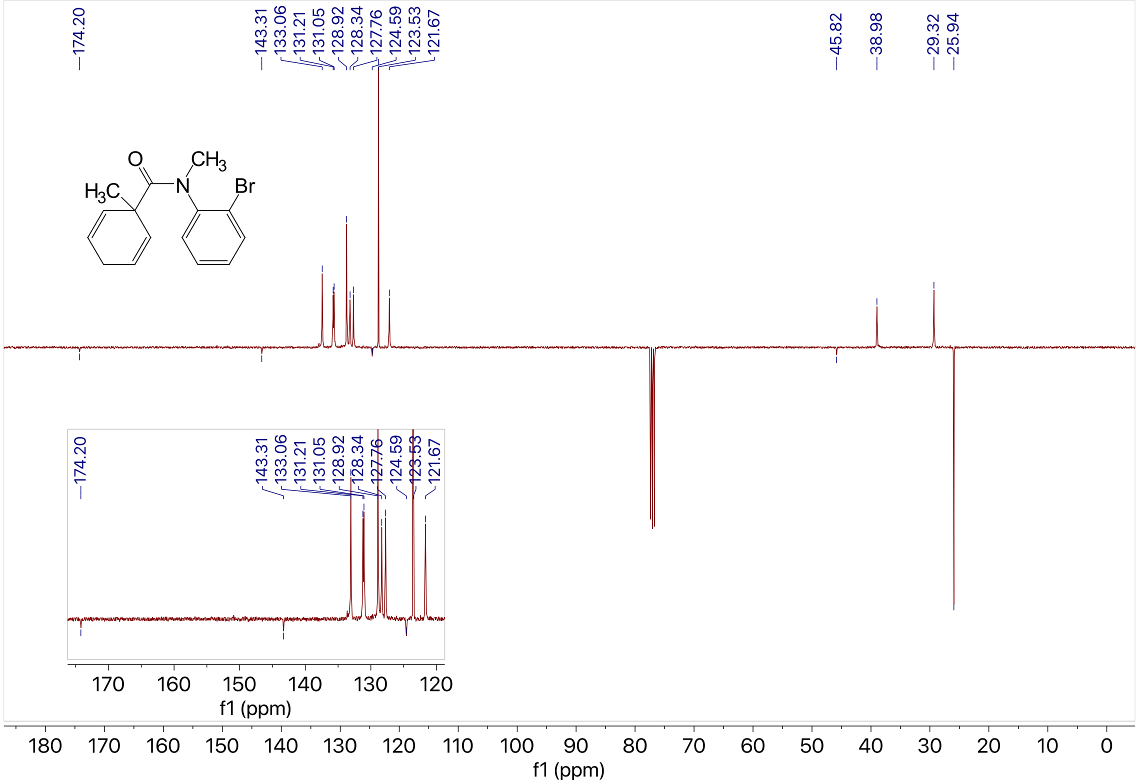

***N*-(2-Iodophenyl)-*N*,1-dimethylcyclohexa-2,5-diene-1-carboxamide (1a-I).**

^{1}H NMR (400 MHz), **1a-I**

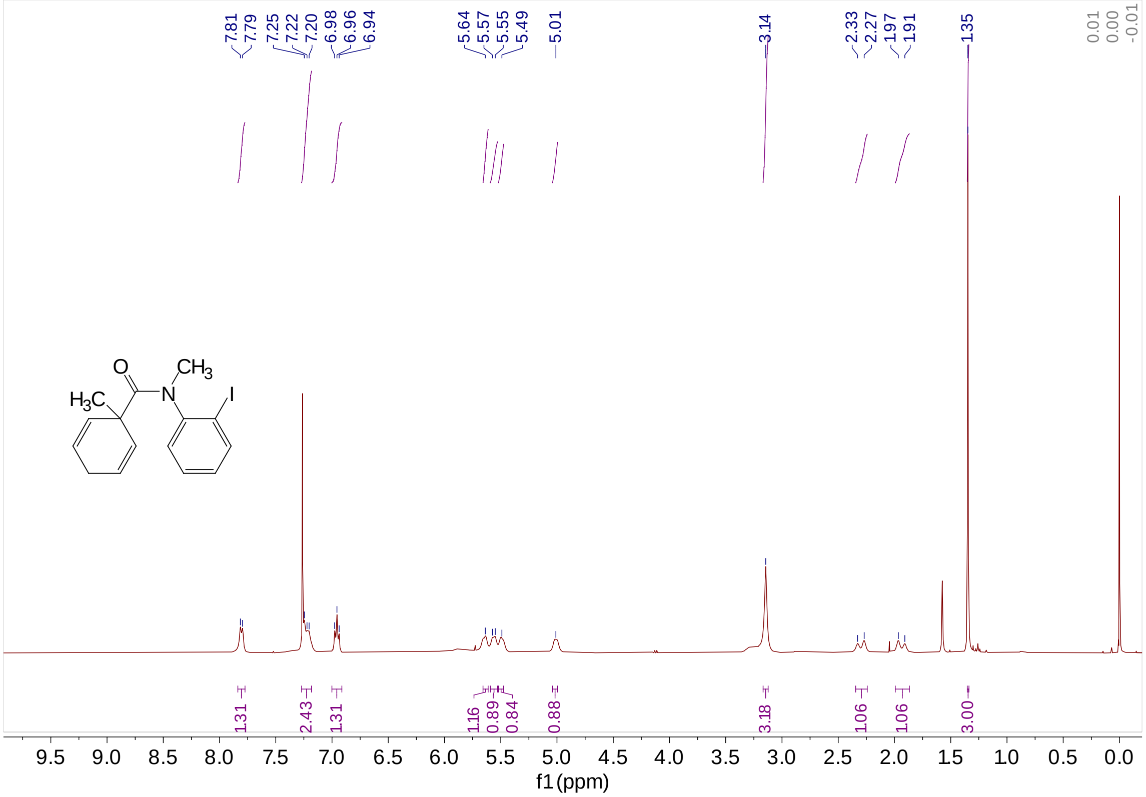

^{13}C{^{1}H} NMR (101 MHz, CDCl$_3$), **1a-I**

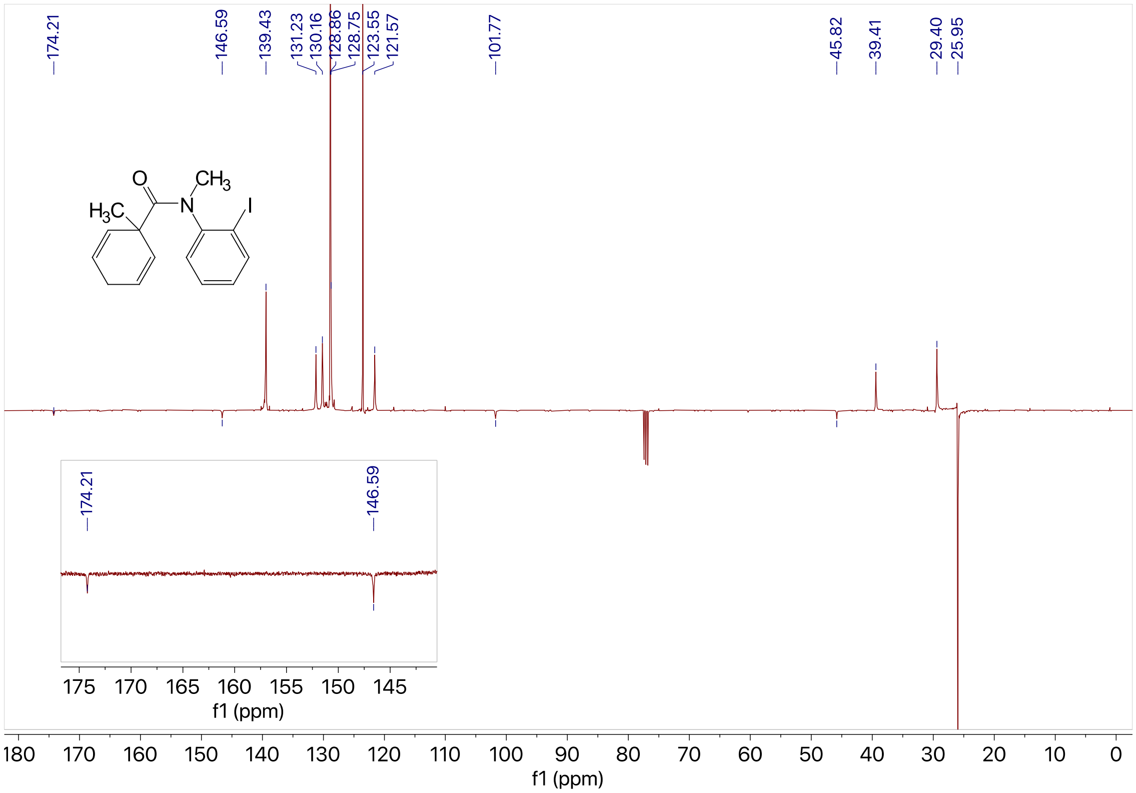

_N_-(2-Bromophenyl)-1-methylcyclohexa-2,5-diene-1-carboxamide (S2b).

^{1}H NMR (400 MHz), **S2b**

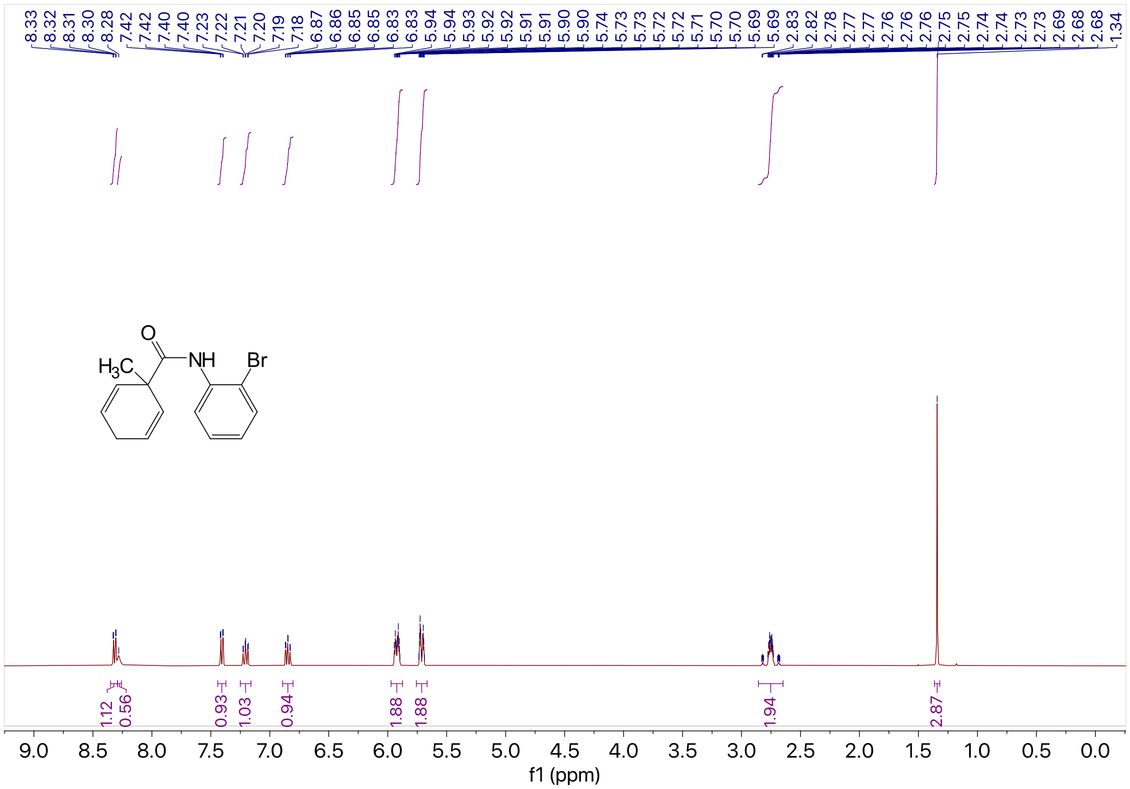

^{13}C{^{1}H} NMR (101 MHz, $CDCl_3$), **S2b**

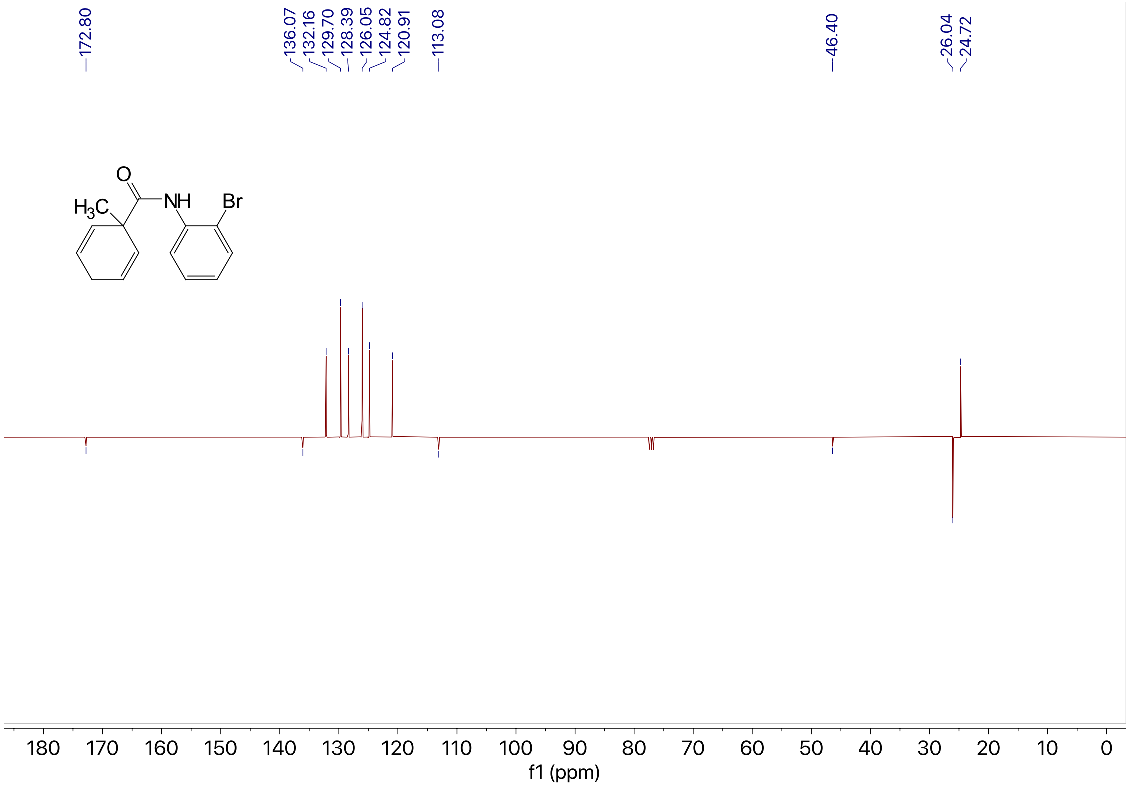

_N_-(2-Iodophenyl)-1-methylcyclohexa-2,5-diene-1-carboxamide (S2b-I).

^{1}H NMR (400 MHz), **S2b-I**

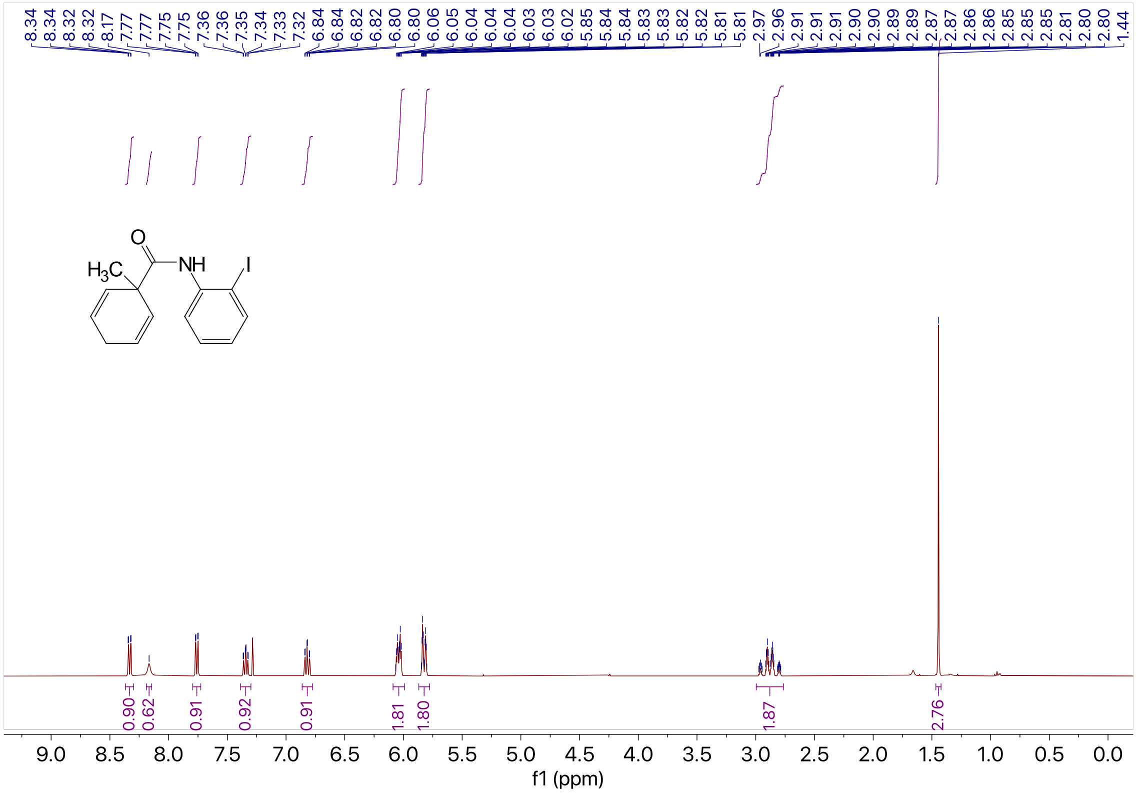

$^{13}C\{^{1}H\}$ NMR (101 MHz, $CDCl_3$), **S2b-I**

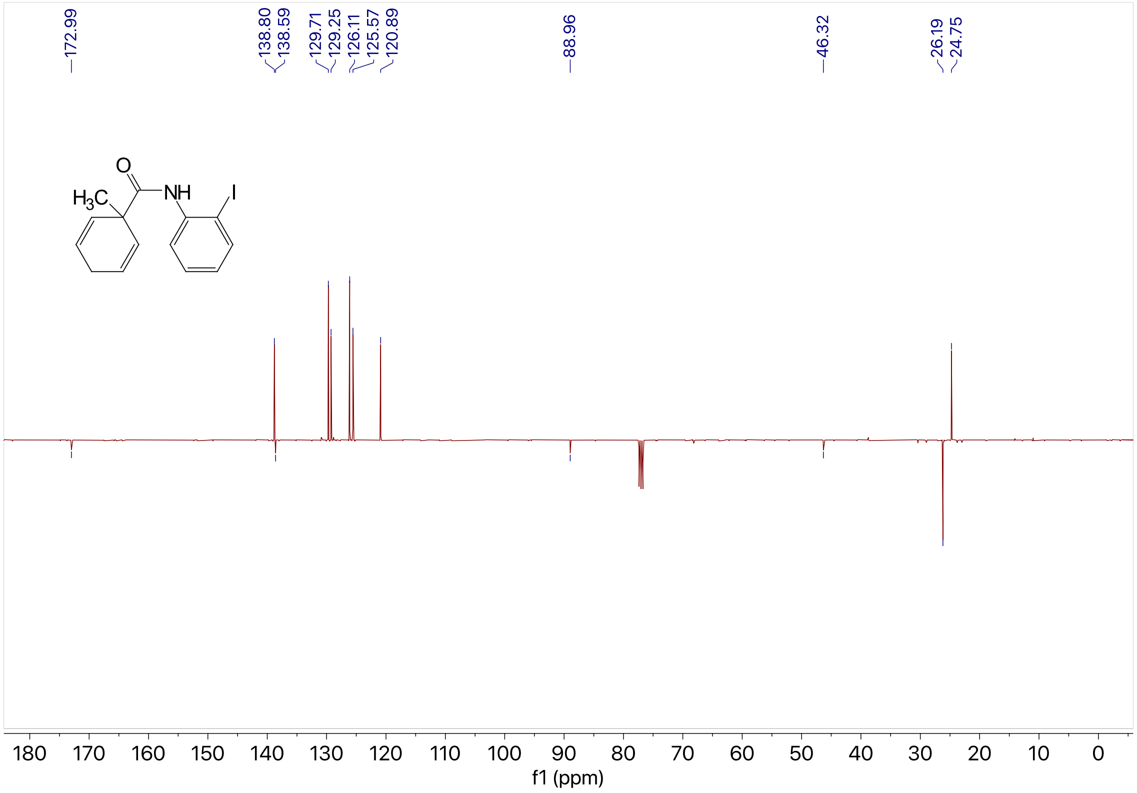

1-Ethyl-*N*-(2-iodophenyl)cyclohexa-2,5-diene-1-carboxamide (S2c-I).

^{1}H NMR (400 MHz), **S2c-I**

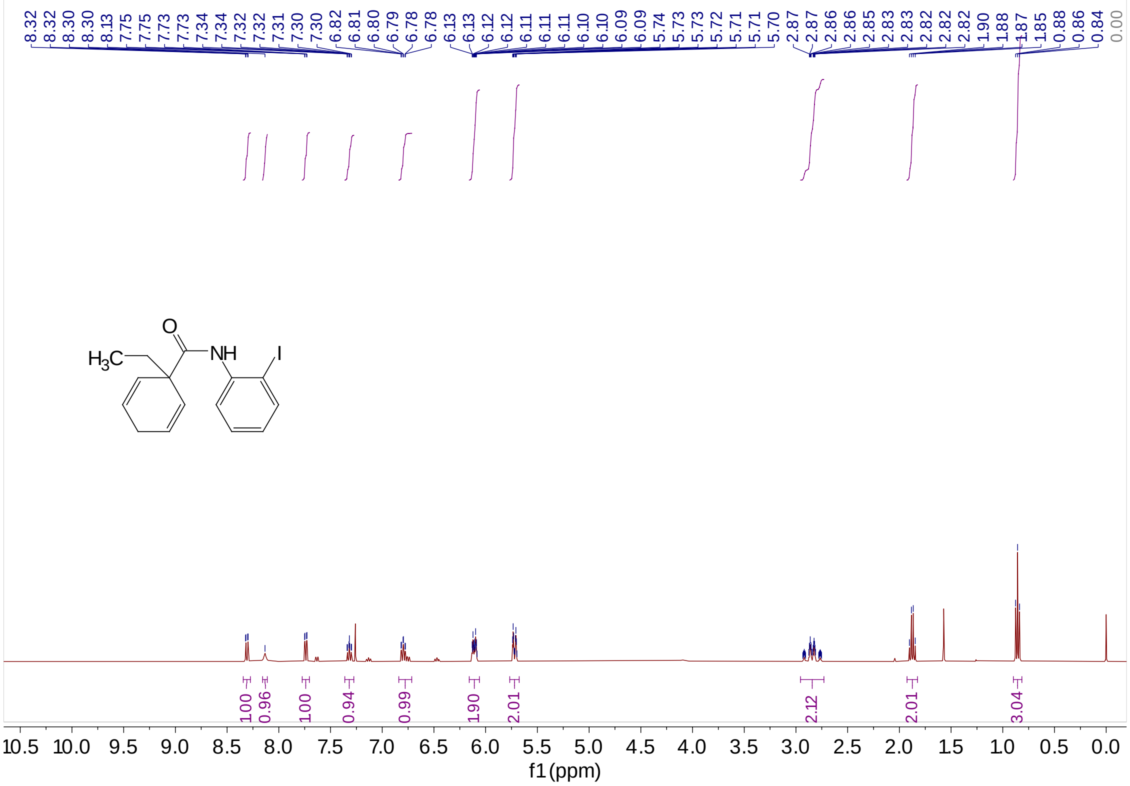

^{13}C{^{1}H} NMR (101 MHz, $CDCl_3$), **S2c-I**

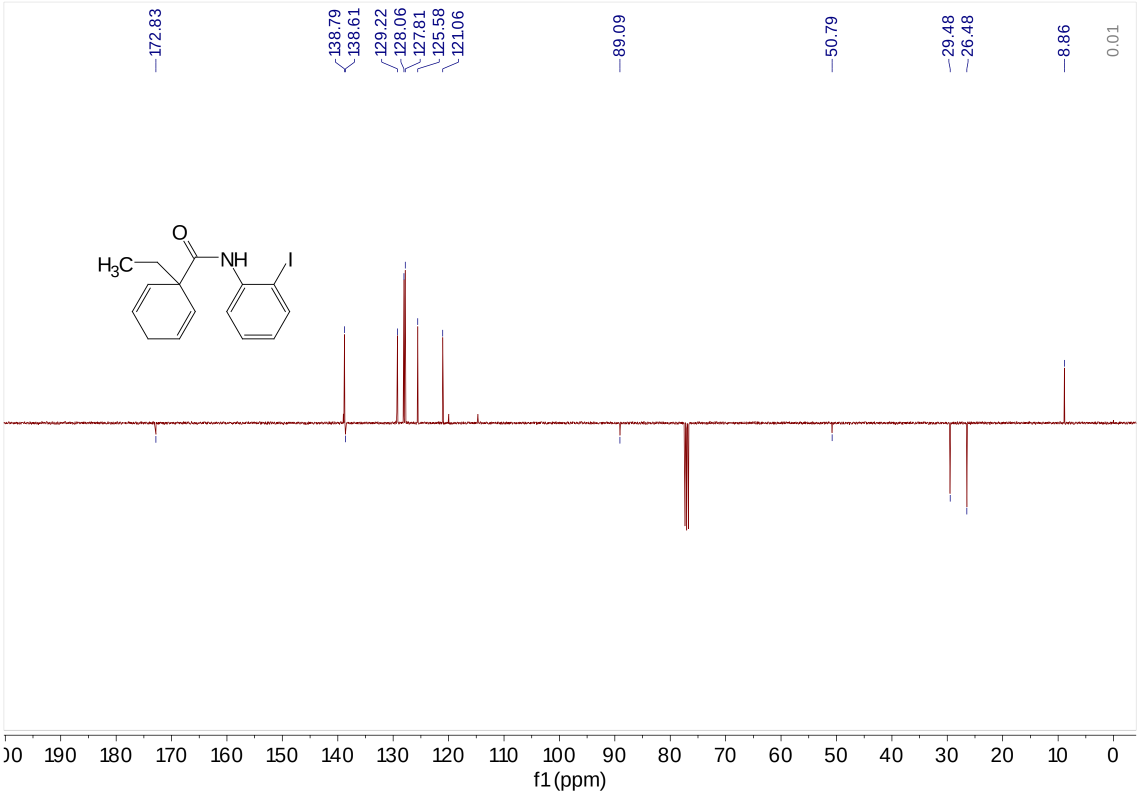

N-(2-Bromophenyl)-1-isopropyl-N-methylcyclohexa-2,5-diene-1-carboxamide (1d).

¹H NMR (400 MHz), **1d**

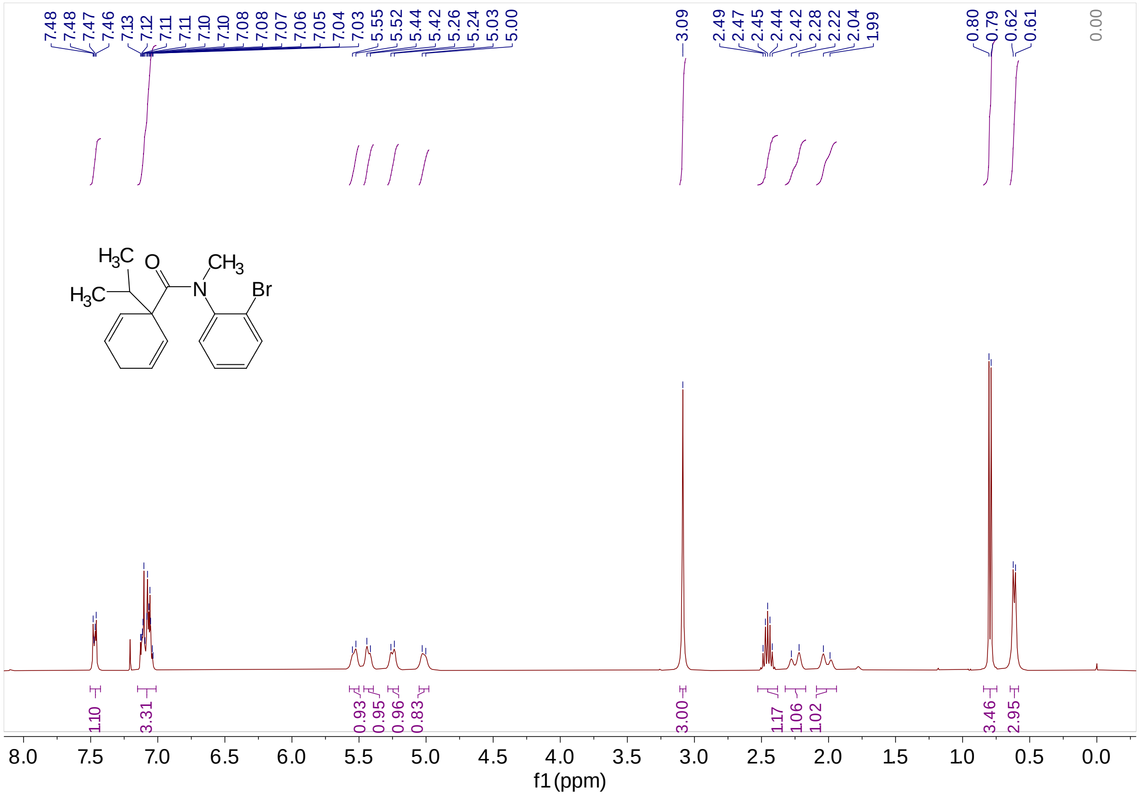

¹³C{¹H} NMR (101 MHz, CDCl₃), **1d**

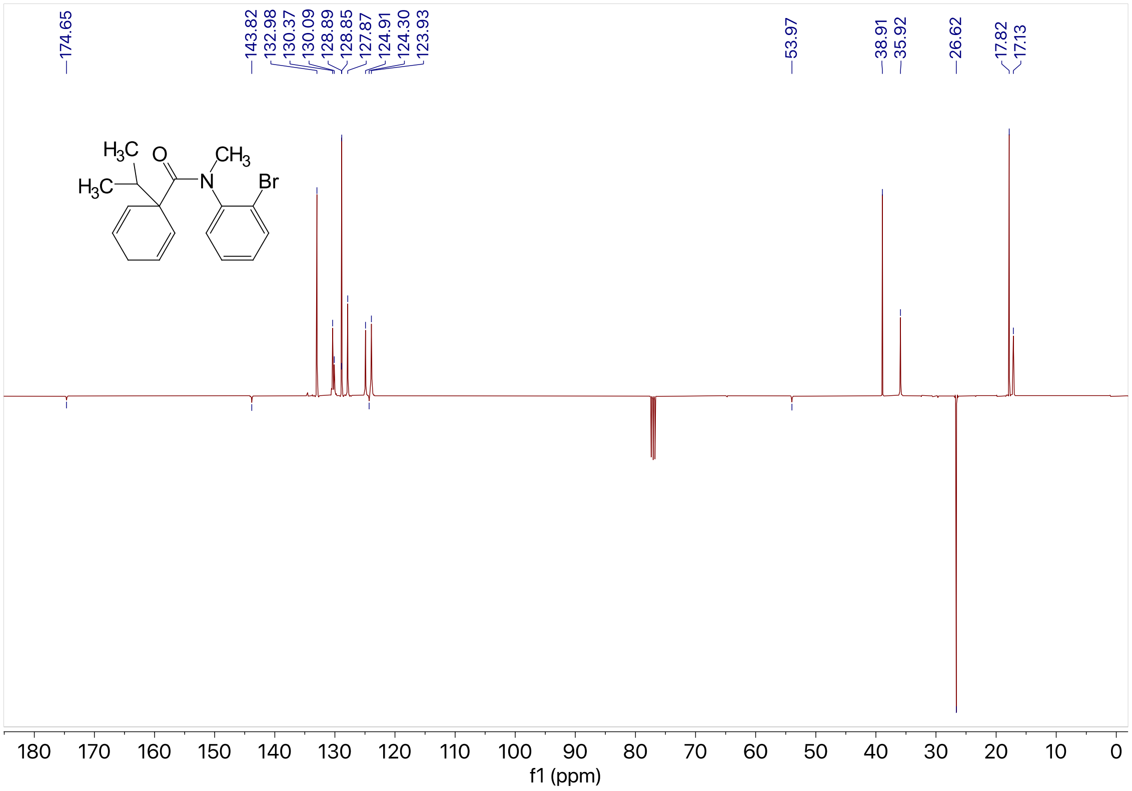

N-(2-Iodophenyl)-1-isopropyl-N-methylcyclohexa-2,5-diene-1-carboxamide (1d-I).

^{1}H NMR (400 MHz), **1d-I**

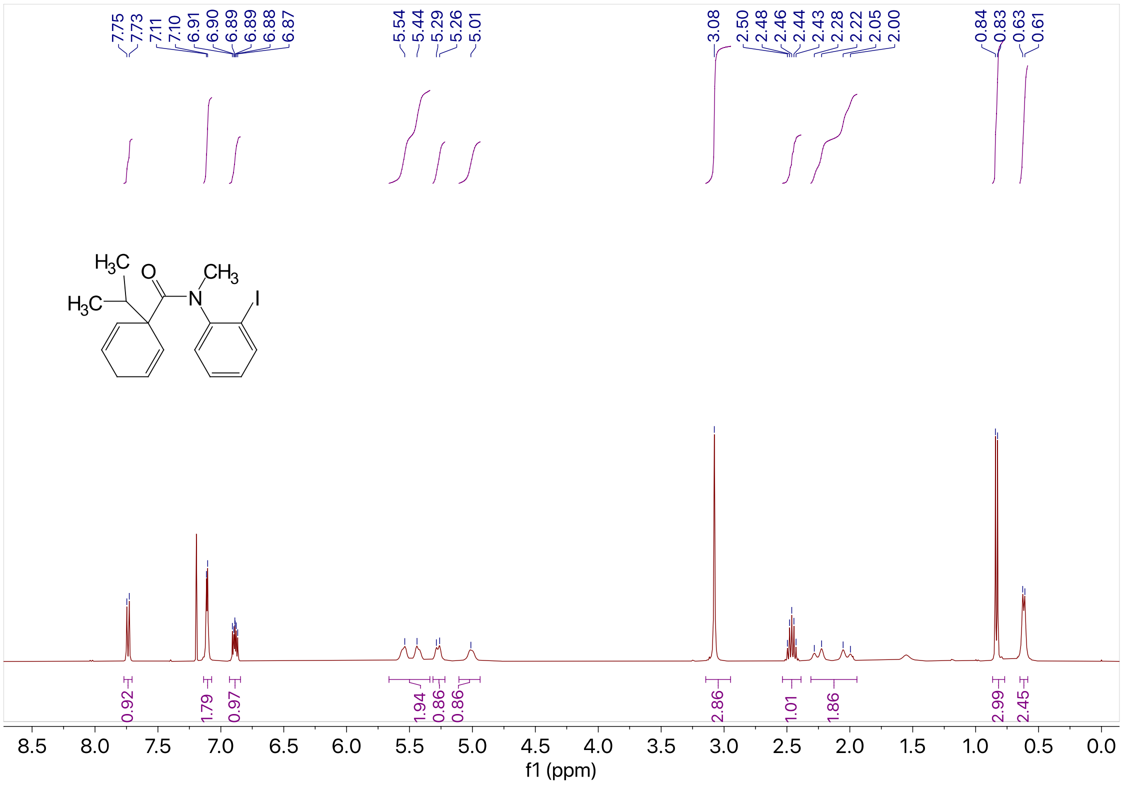

$^{13}C\{^{1}H\}$ NMR (101 MHz, $CDCl_3$), **1d-I**

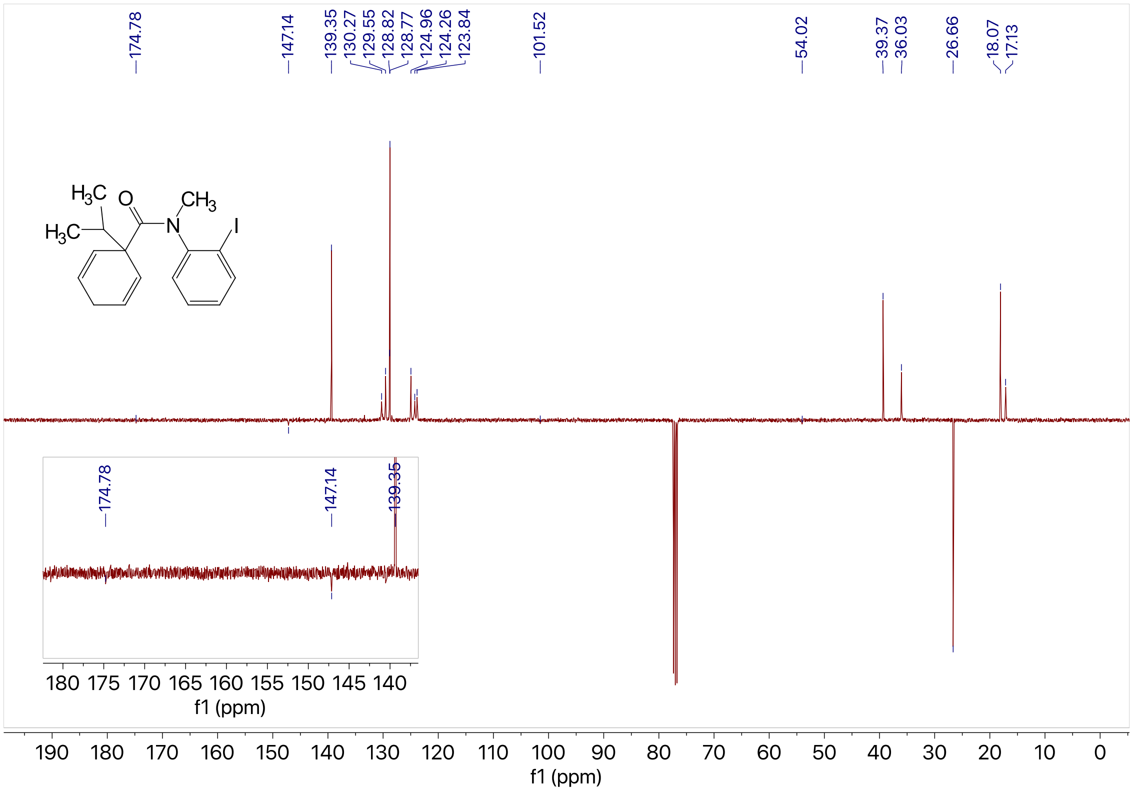

***N*-(2-Bromophenyl)-1-isopropylcyclohexa-2,5-diene-1-carboxamide (S2e).**

^{1}H NMR (400 MHz), **S2e**

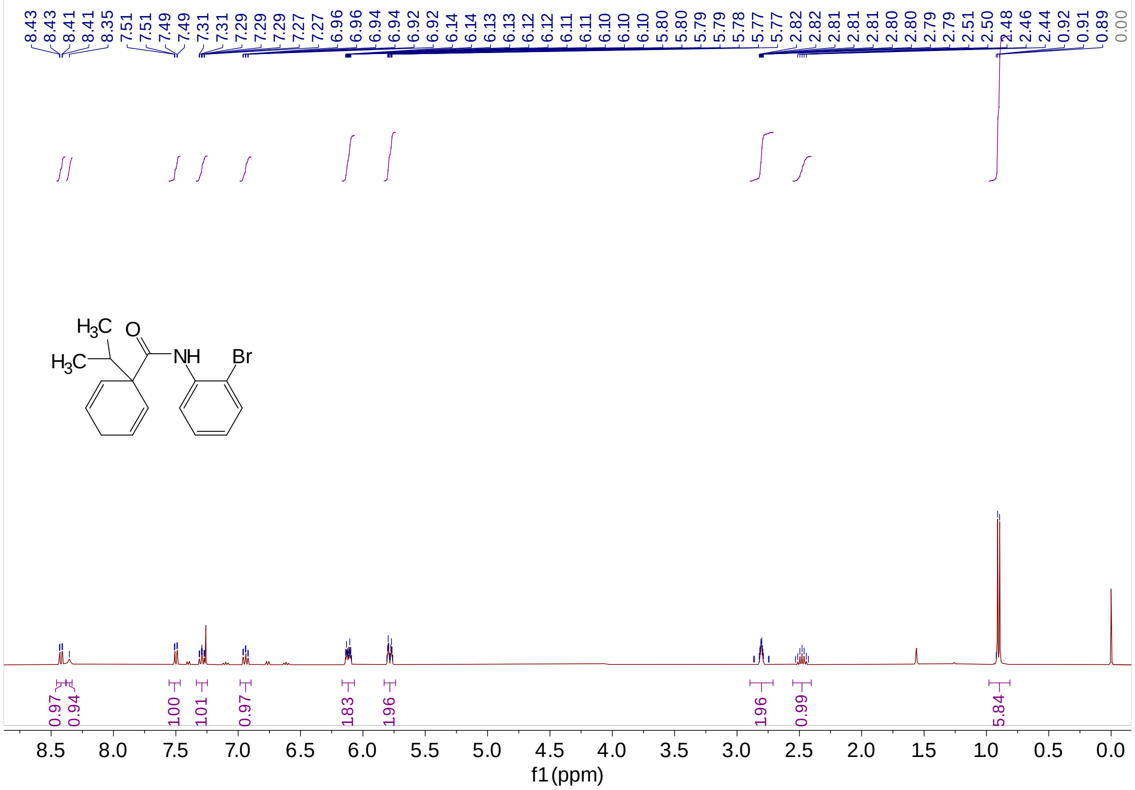

^{13}C{^{1}H} NMR (101 MHz, $CDCl_3$), **S2e**

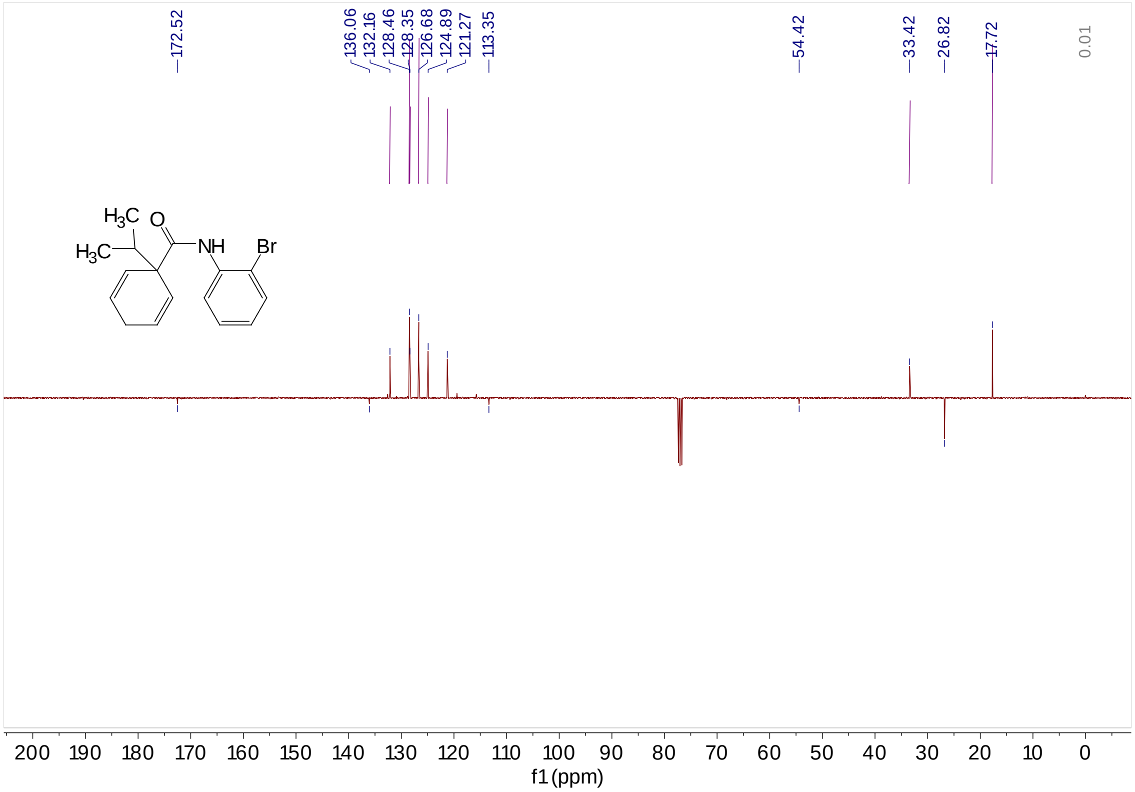

_N_-(2-Iodophenyl)-1-isopropylcyclohexa-2,5-diene-1-carboxamide (S2e-I).
^{1}H NMR (400 MHz), **S2e-I**

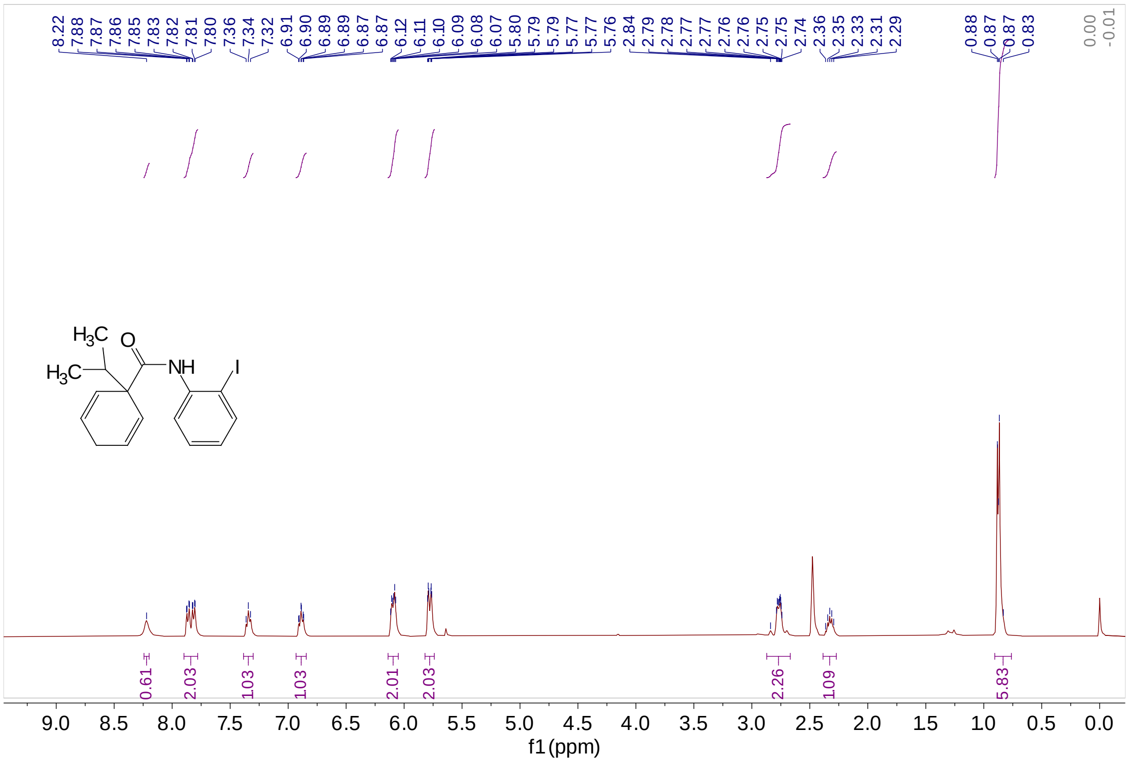

$^{13}C\{^{1}H\}$ NMR (101 MHz, $CDCl_3$), **S2e-I**

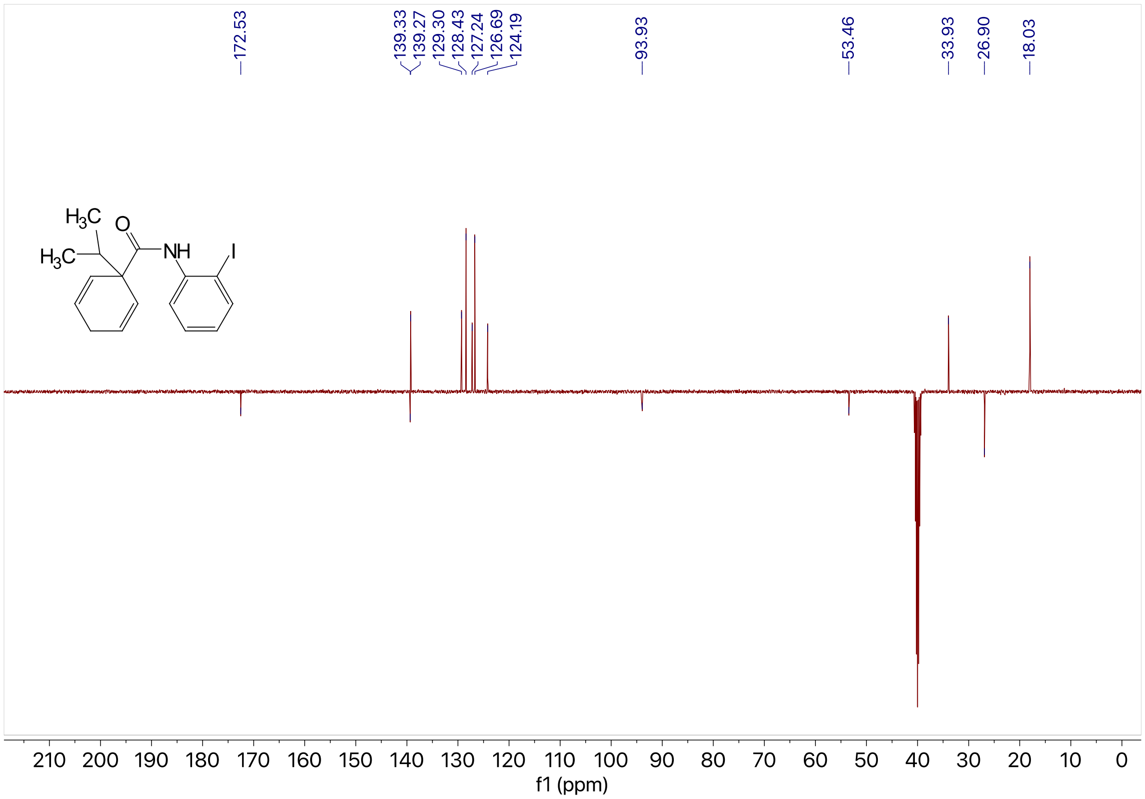

_N_-(2-Iodophenyl)-1-(methoxymethyl)cyclohexa-2,5-diene-1-carboxamide (S2f-I).

^{1}H NMR (400 MHz), **S2f-I**

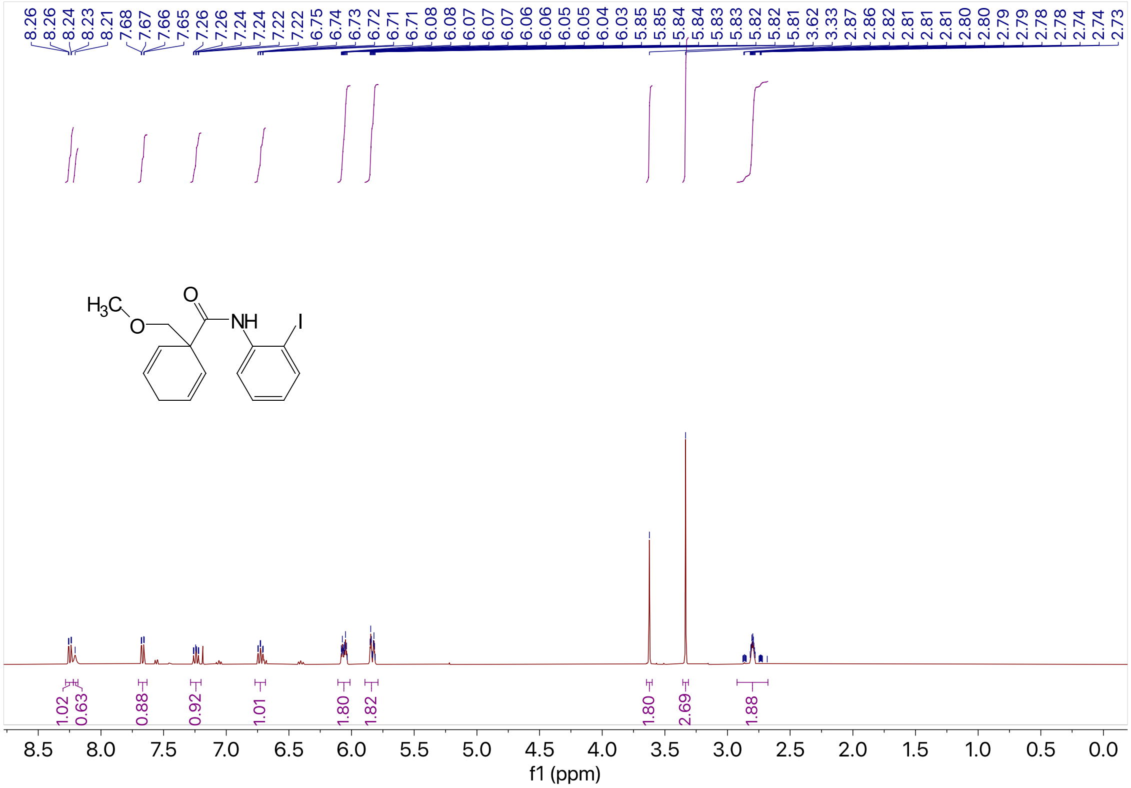

^{13}C{^{1}H} NMR (101 MHz, $CDCl_3$), **S2f-I**

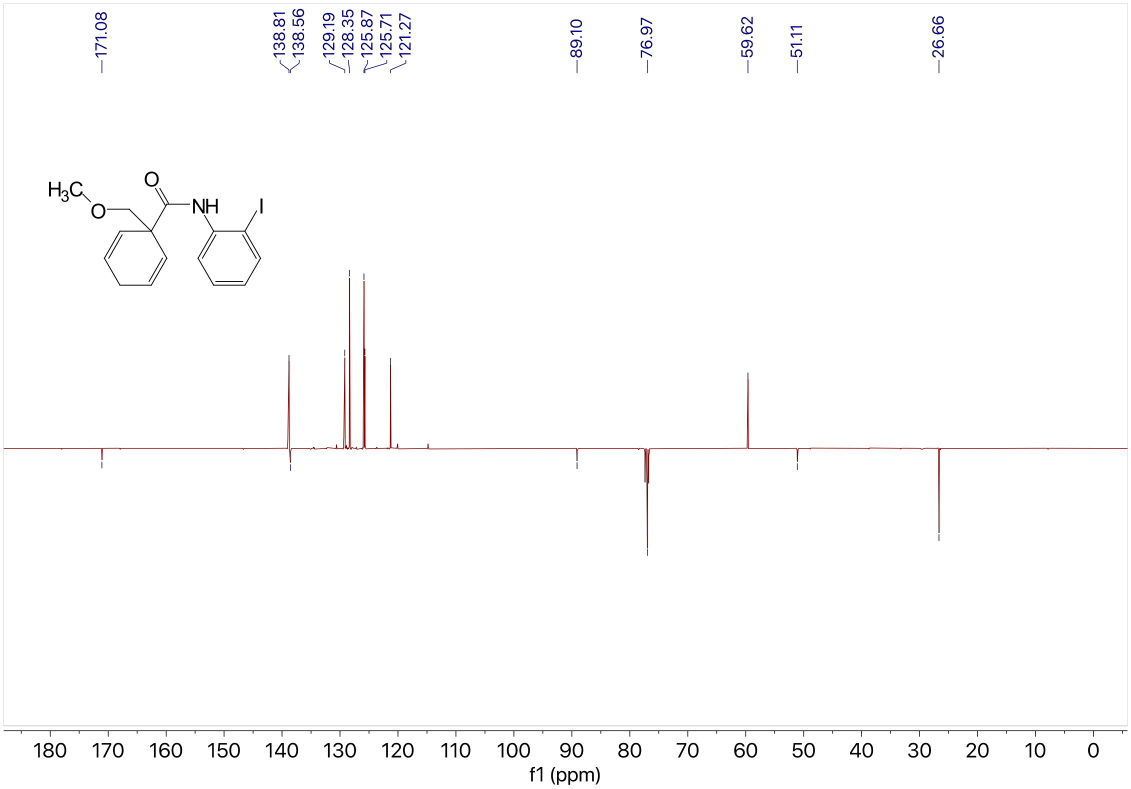

Ethyl 2-(1-((2-iodophenyl)(methyl)carbamoyl)cyclohexa-2,5-dien-1-yl)acetate (1g-I).

^{1}H NMR (400 MHz), **1g-I**

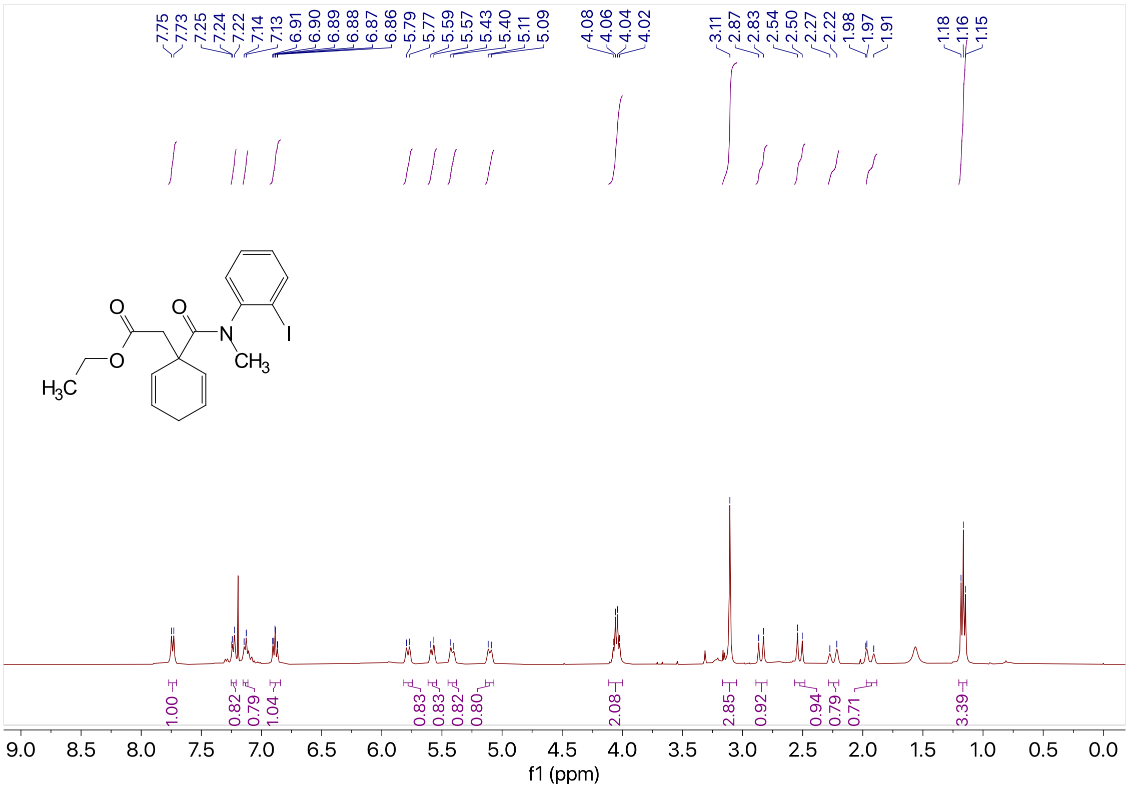

^{13}C{^{1}H} NMR (101 MHz, $CDCl_3$), **1g-I**

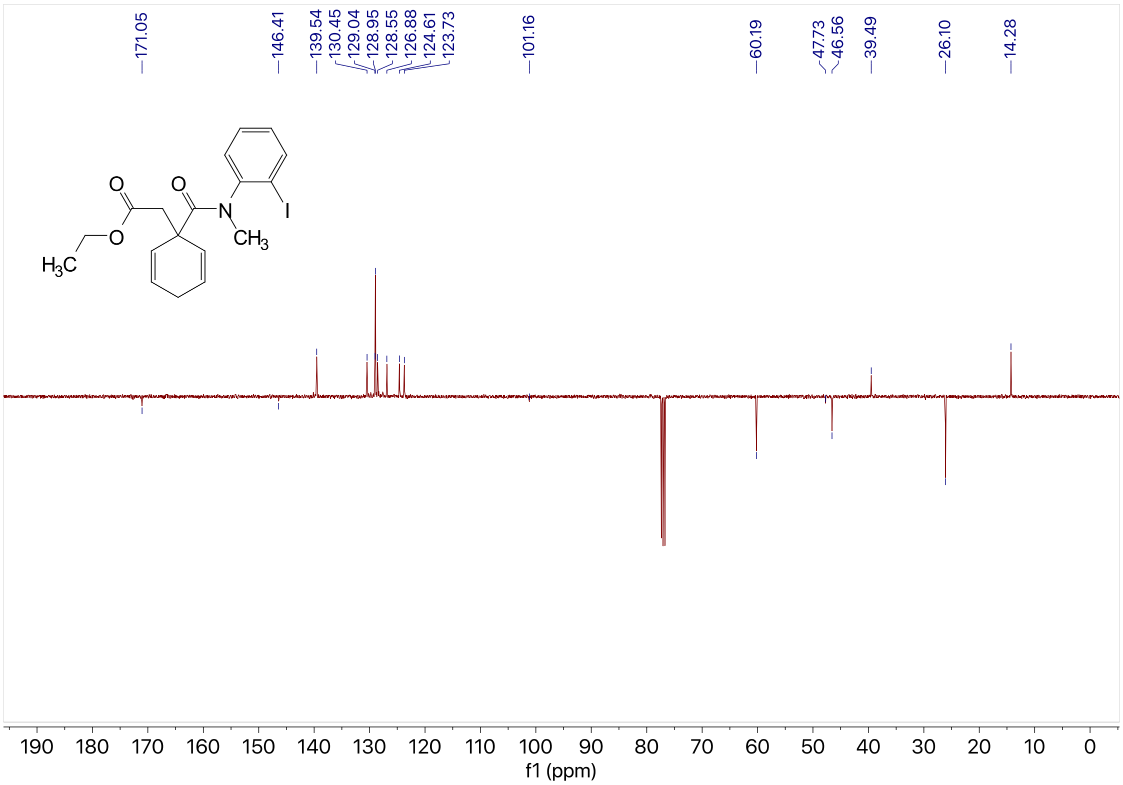

1-Benzyl-N-(2-bromophenyl)cyclohexa-2,5-diene-1-carboxamide (S2h).

^{1}H NMR (400 MHz), **S2h**

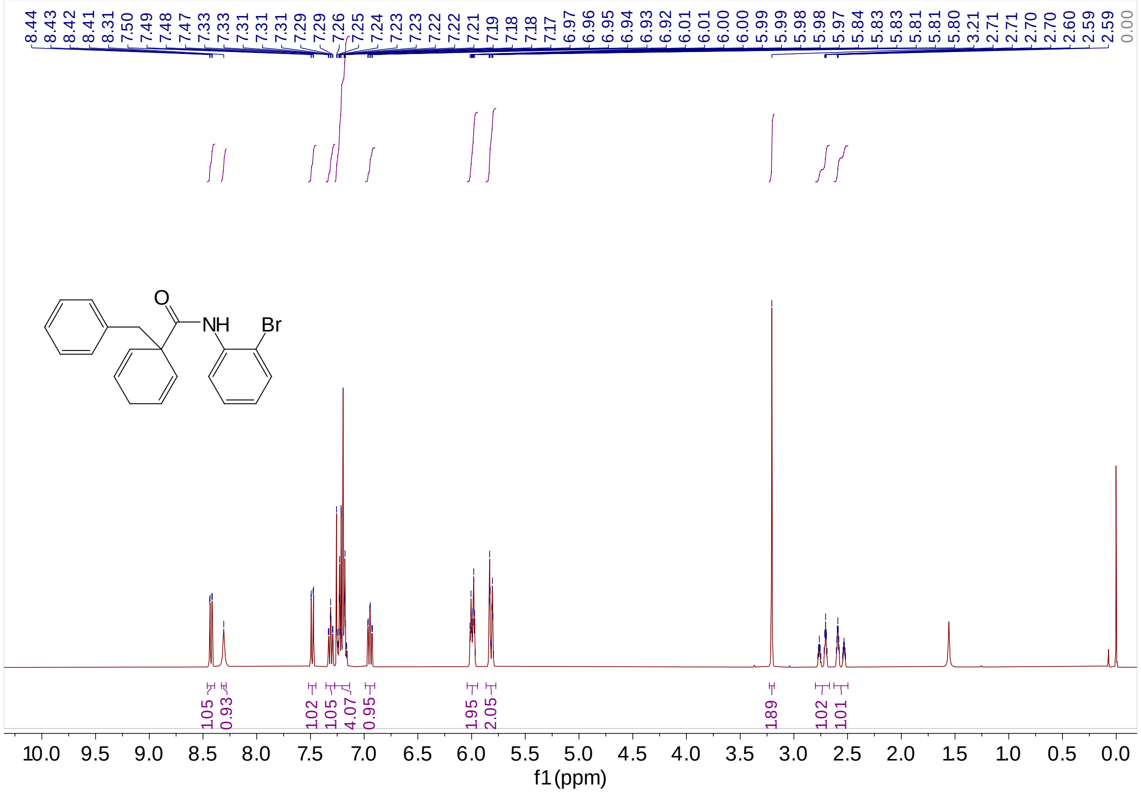

$^{13}C\{^{1}H\}$ NMR (101 MHz, $CDCl_3$), **S2h**

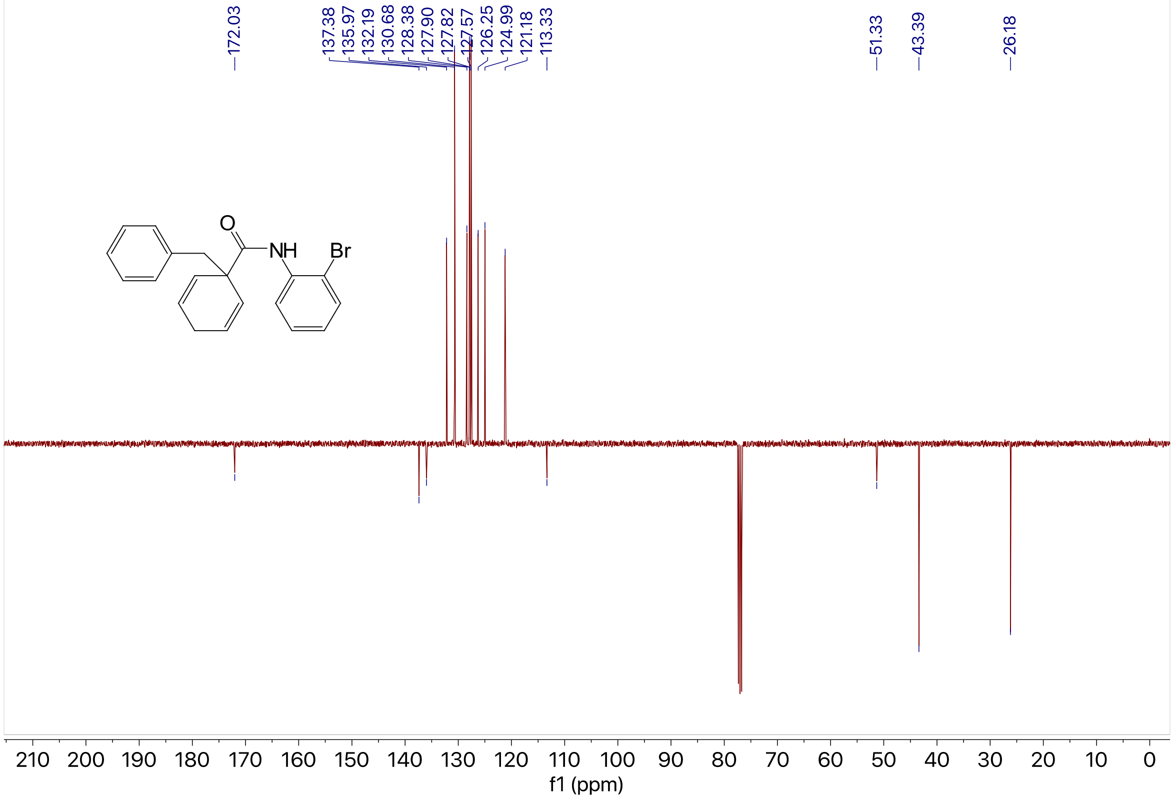

1-Benzyl-*N*-(2-iodophenyl)cyclohexa-2,5-diene-1-carboxamide (S2h-I).

^{1}H NMR (400 MHz), **S2h-I**

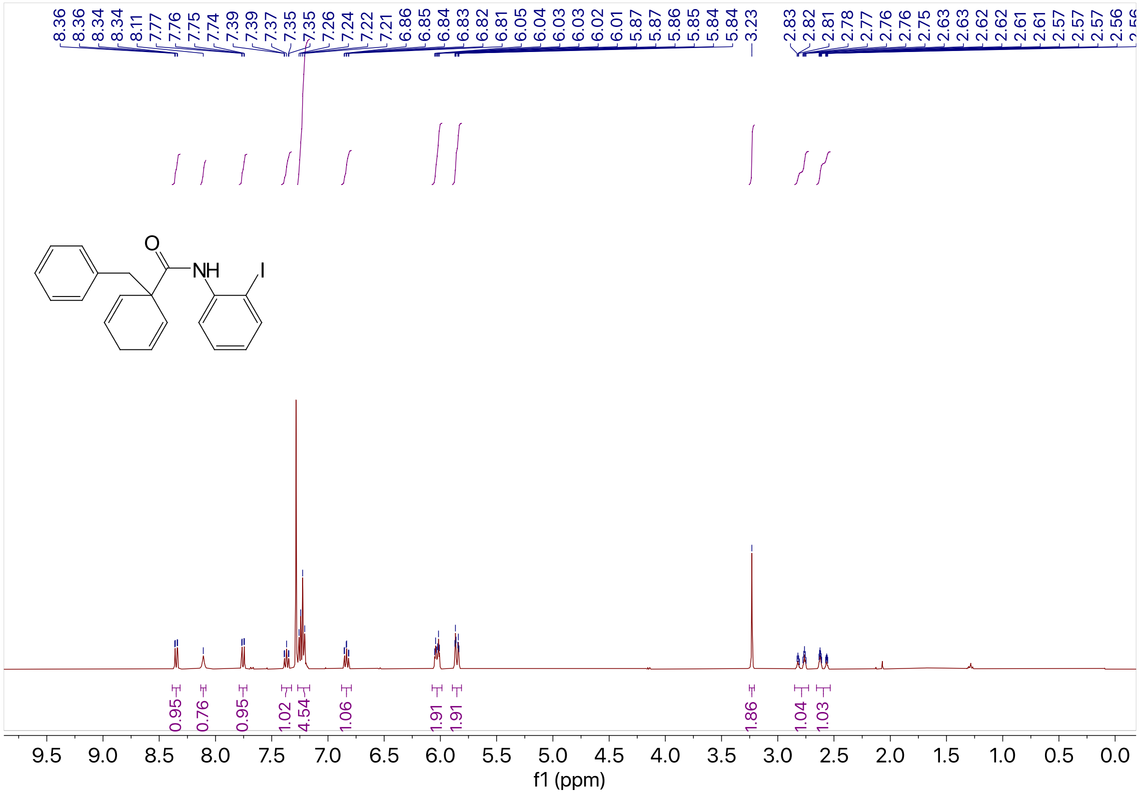

^{13}C{^{1}H} NMR (101 MHz, $CDCl_3$), **S2h-I**

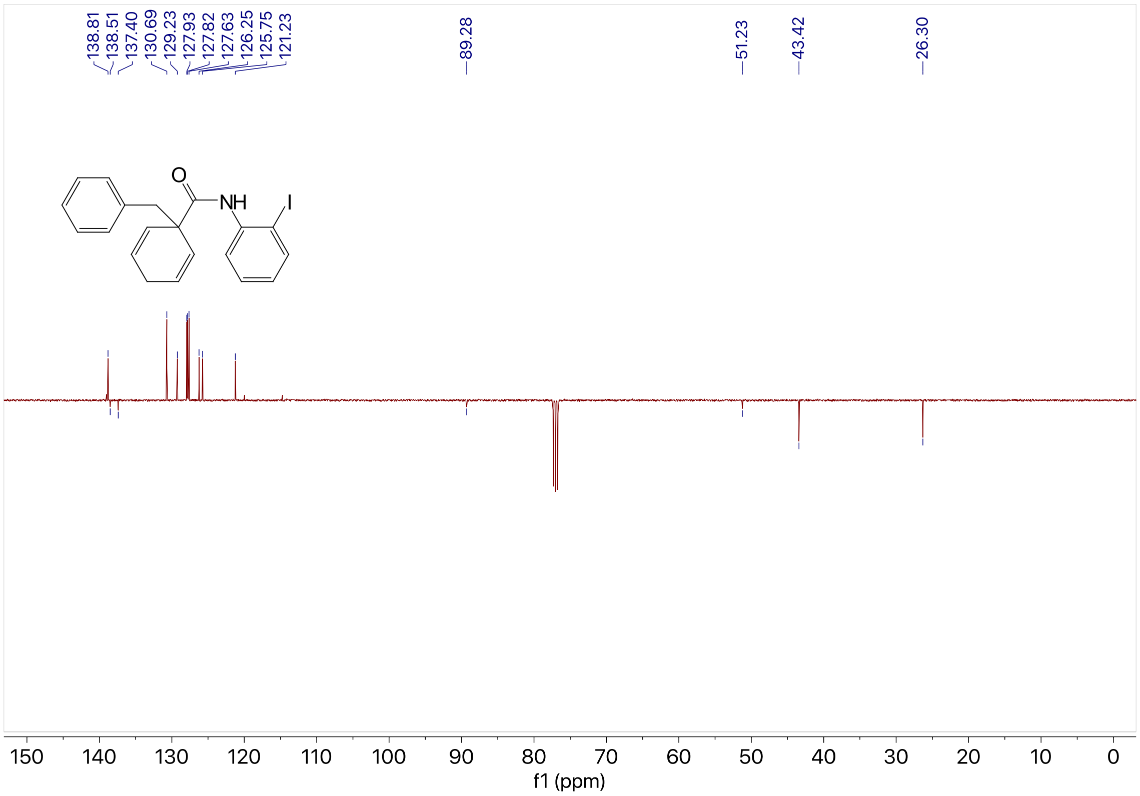

N-(2-Bromo-4-fluorophenyl)-1-isopropylcyclohexa-2,5-diene-1-carboxamide (S2i).

^{1}H NMR (400 MHz), **S2i**

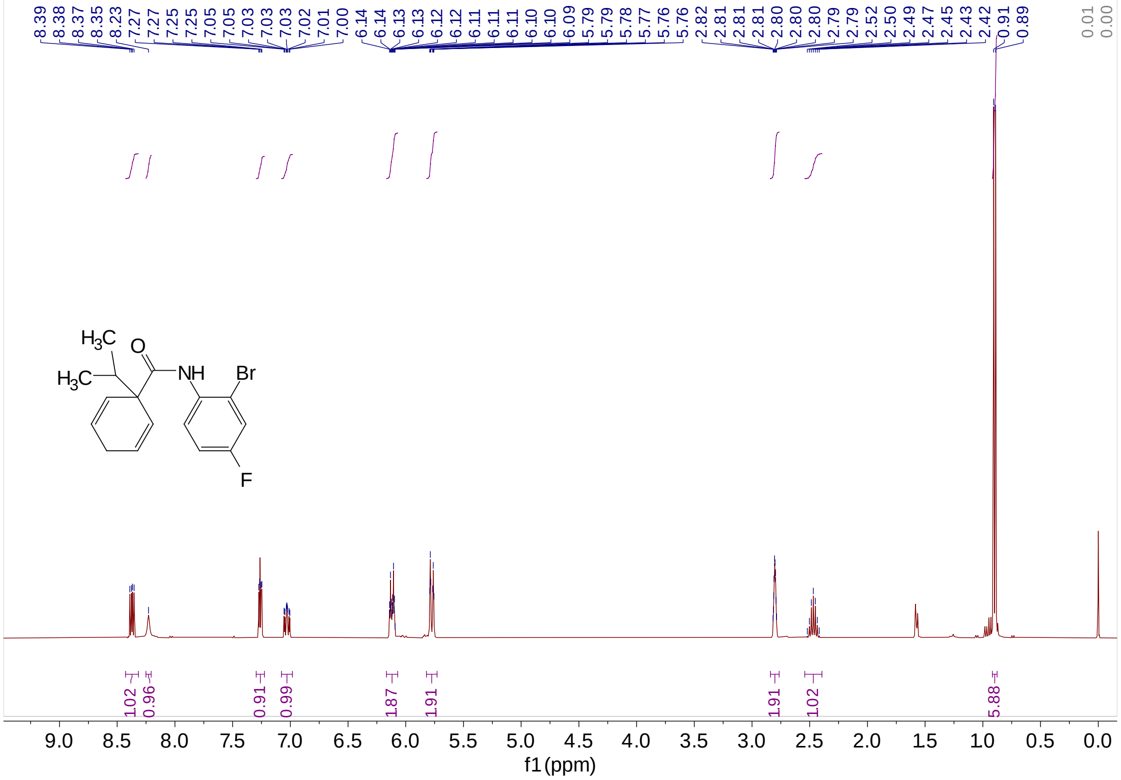

^{13}C{^{1}H} NMR (101 MHz, $CDCl_3$), **S2i**

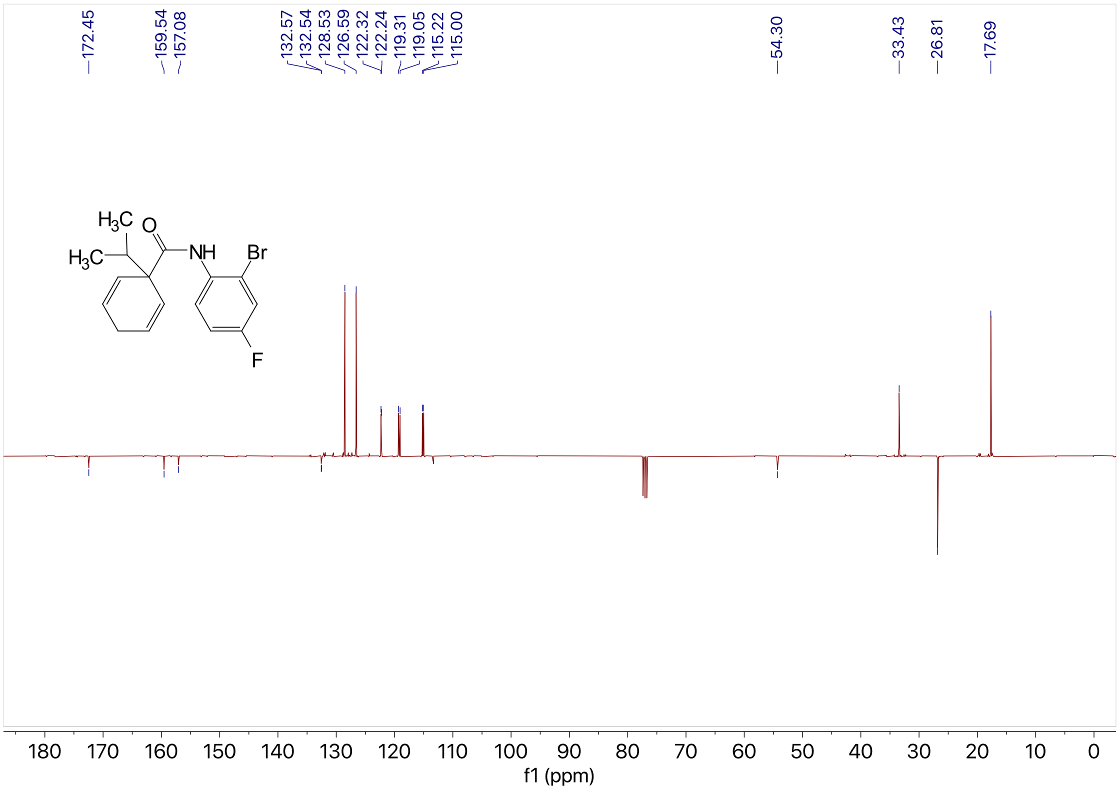

^{19}*F NMR (376 MHz),* **S2i**

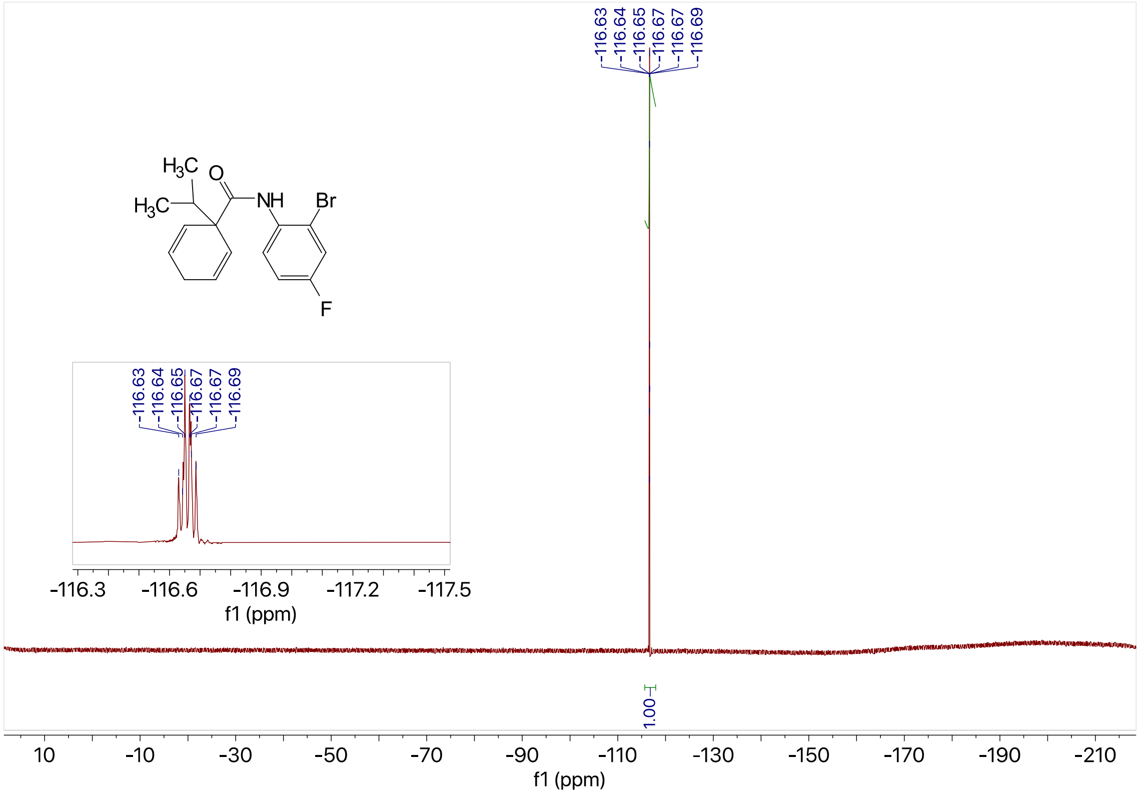

***N*-(4-Fluoro-2-iodophenyl)-1-isopropylcyclohexa-2,5-diene-1-carboxamide (S2i-I).**

^{1}*H NMR (400 MHz),* **S2i-I**

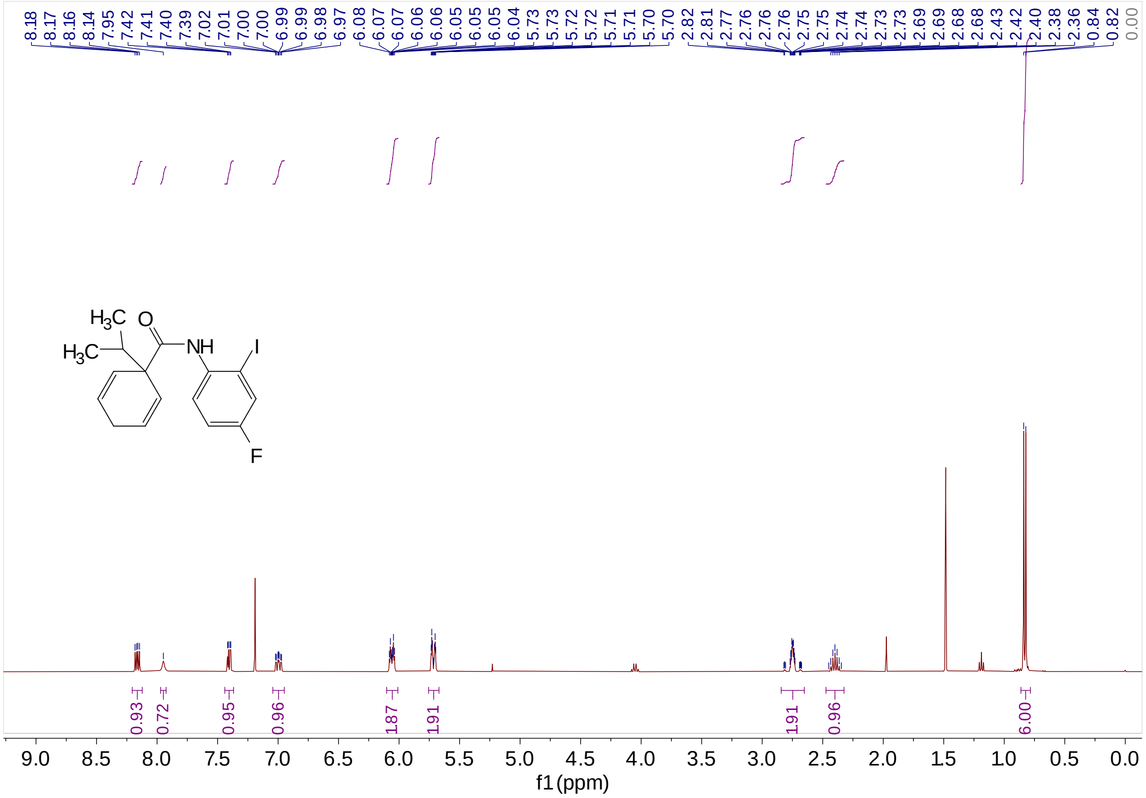

$^{13}C\{^{1}H\}$ NMR (101 MHz, $CDCl_3$), **S2i-I**

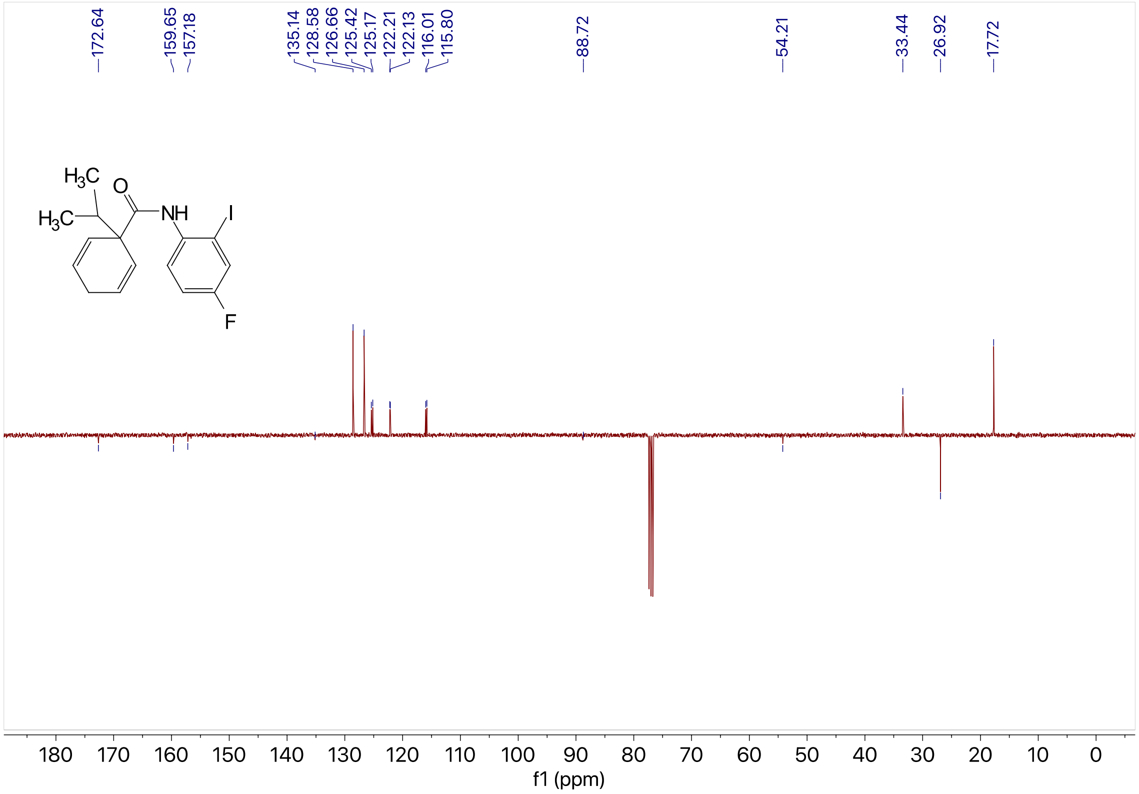

^{19}F NMR (376 MHz), **S2i-I**

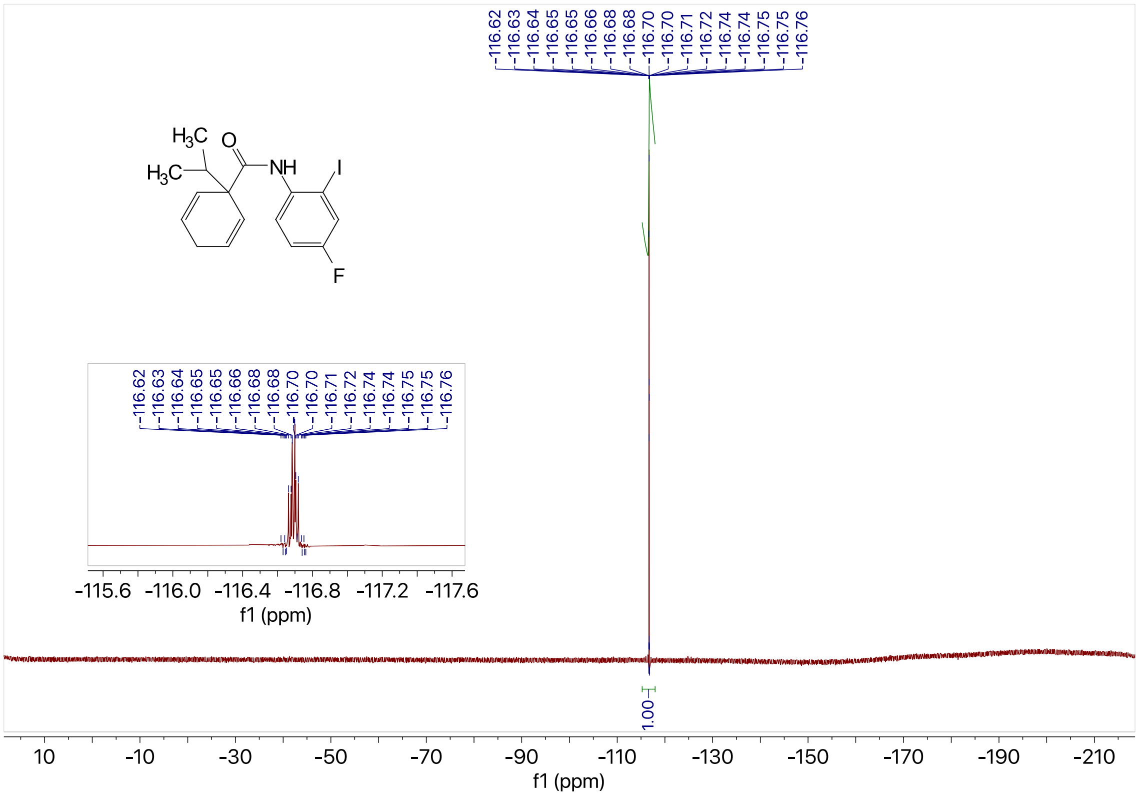

N-(2-Bromo-4-chlorophenyl)-1-isopropylcyclohexa-2,5-diene-1-carboxamide (S2j).

^{1}H NMR (400 MHz), **S2j**

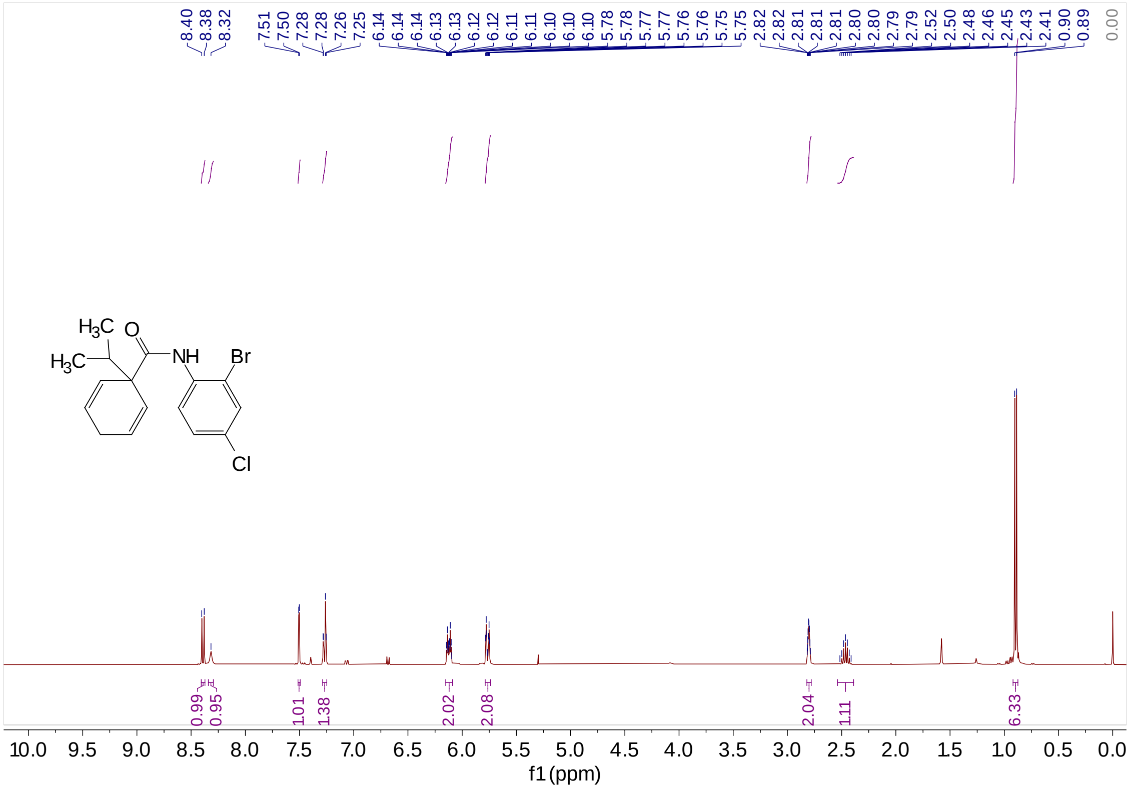

^{13}C{^{1}H} NMR (101 MHz, $CDCl_3$), **S2j**

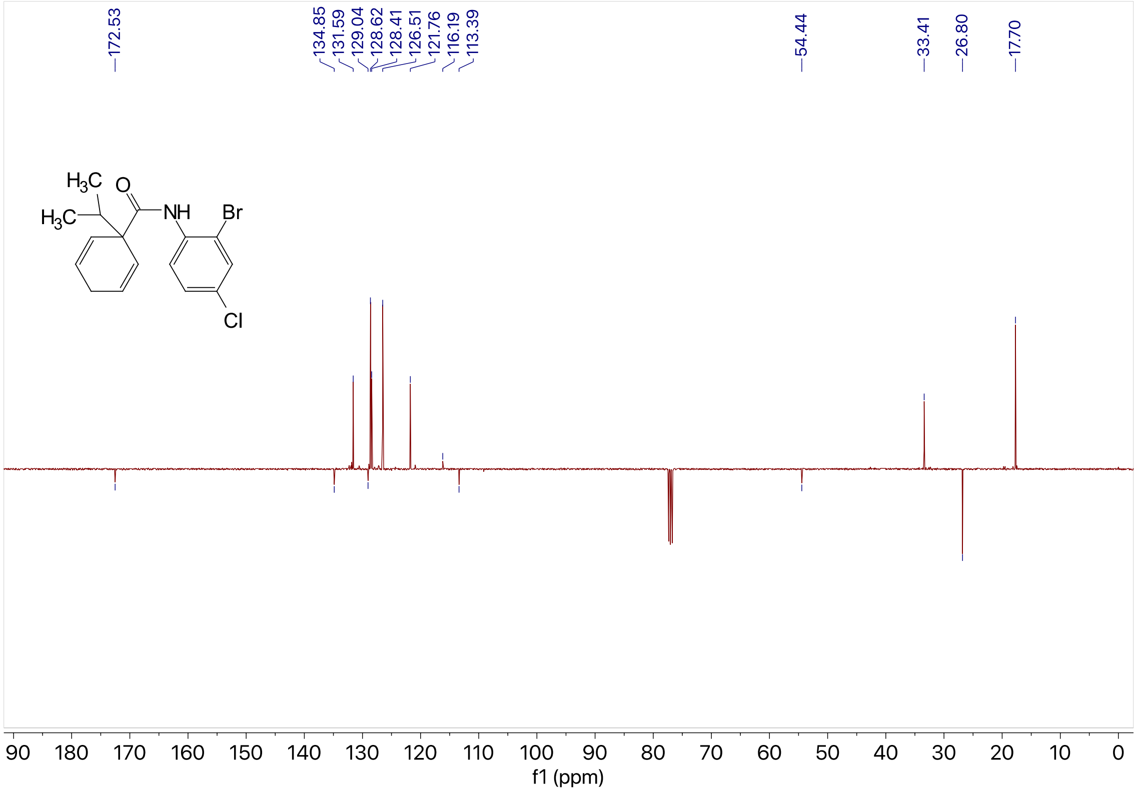

_N_-(4-Chloro-2-iodophenyl)-1-isopropylcyclohexa-2,5-diene-1-carboxamide (S2j-I).

^{1}H NMR (400 MHz), **S2j-I**

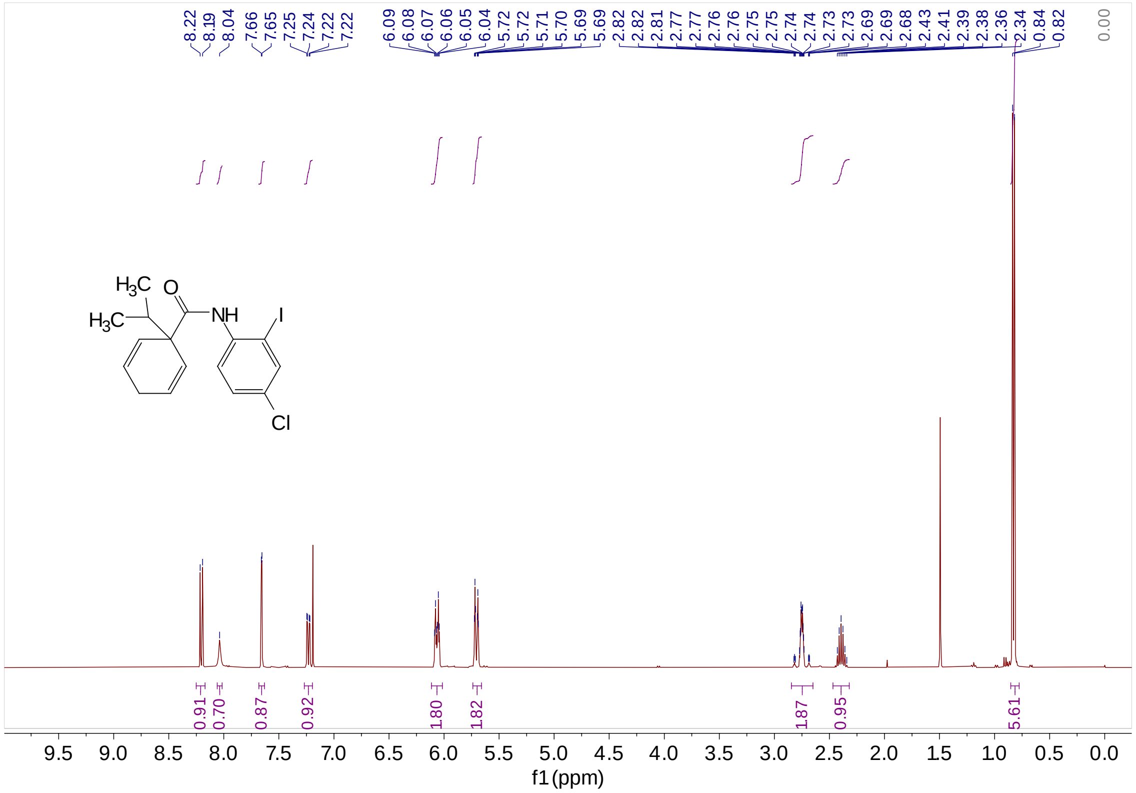

$^{13}C\{^{1}H\}$ NMR (101 MHz, $CDCl_3$), **S2j-I**

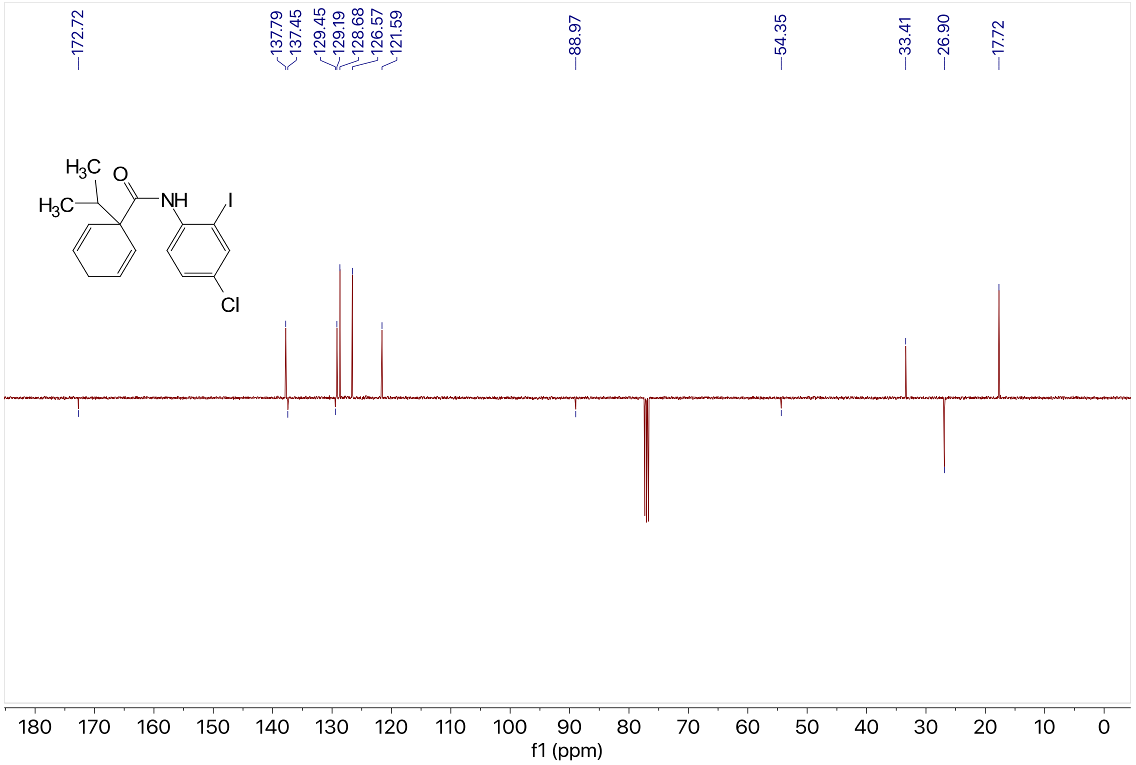

***N*-(2-Bromo-4-methylphenyl)-1-isopropylcyclohexa-2,5-diene-1-carboxamide (S2k).**

^{1}H NMR (400 MHz), **S2k**

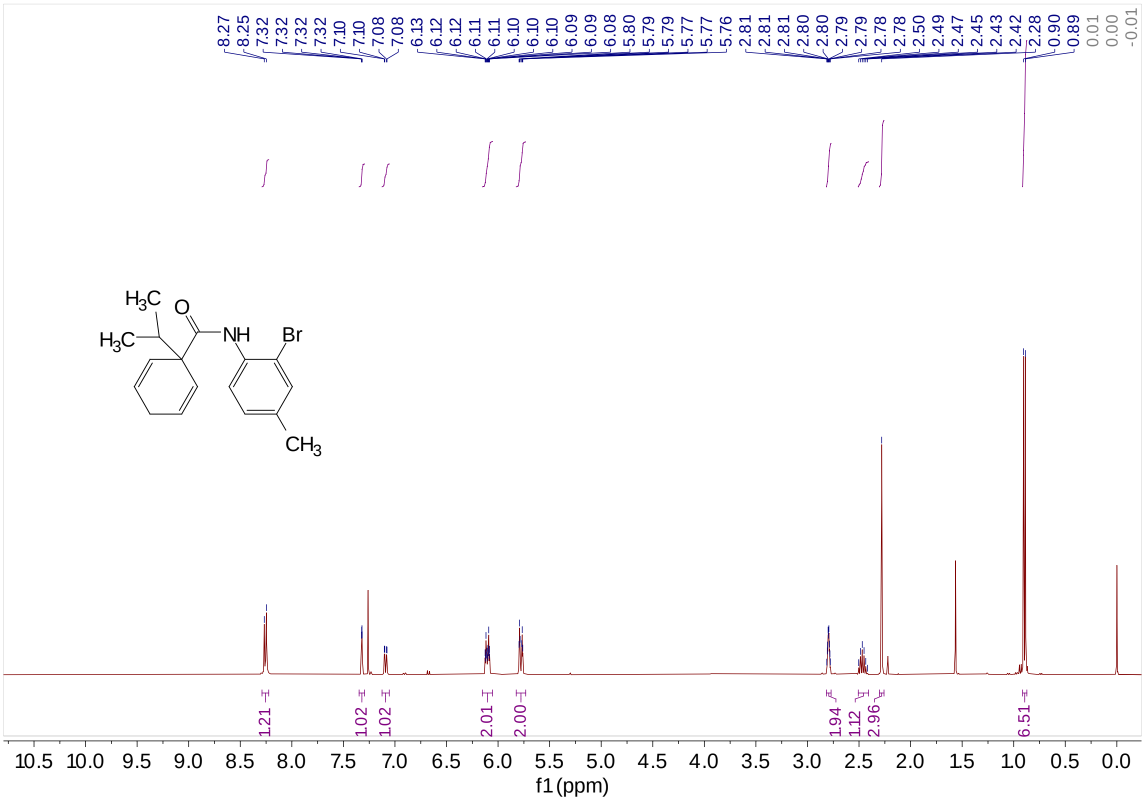

^{13}C{^{1}H} NMR (101 MHz, $CDCl_3$), **S2k**

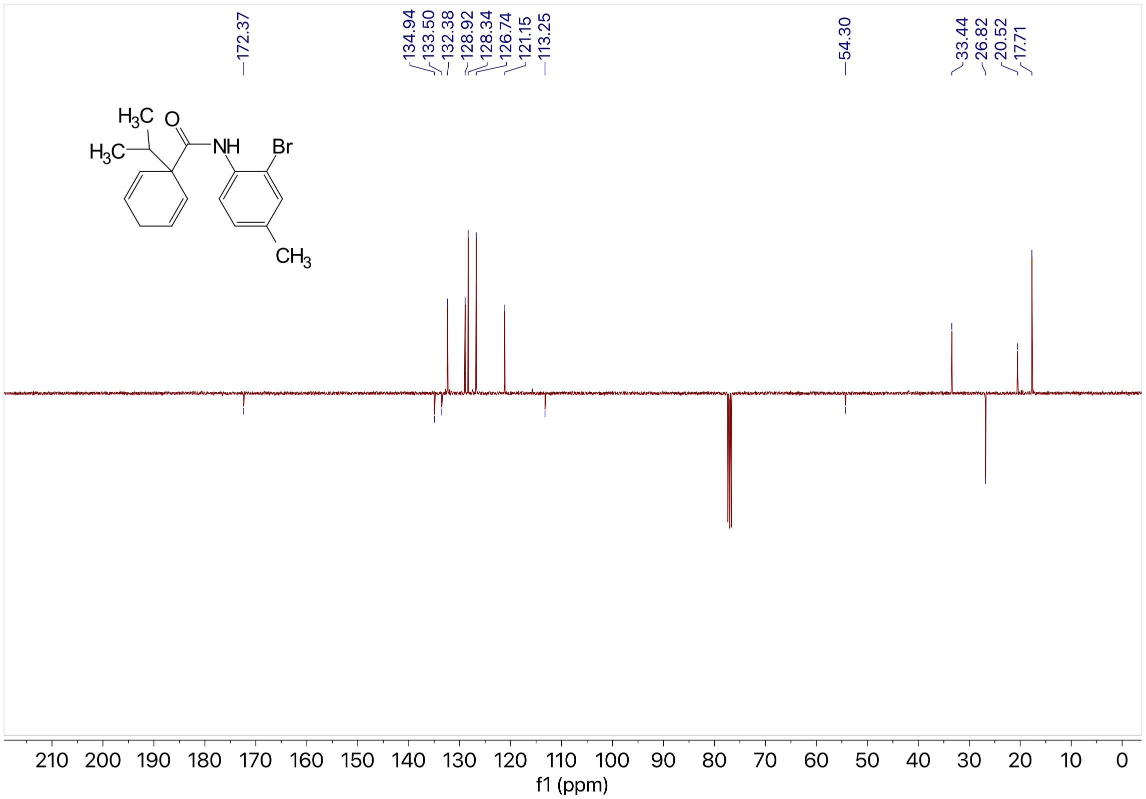

***N*-(2-Iodo-5-methylphenyl)-1-isopropylcyclohexa-2,5-diene-1-carboxamide (S2l-I).**

^{1}H NMR (400 MHz), **S2l-I**

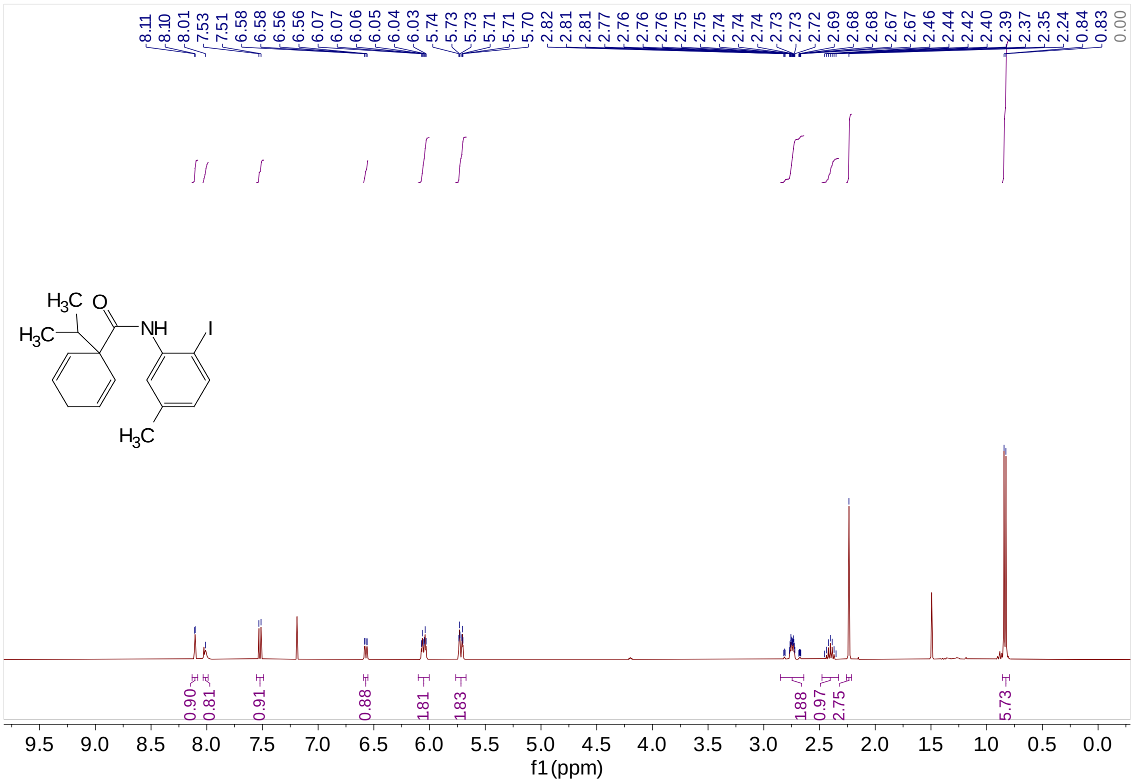

^{13}C{^{1}H} NMR (101 MHz, $CDCl_3$), **S2l-I**

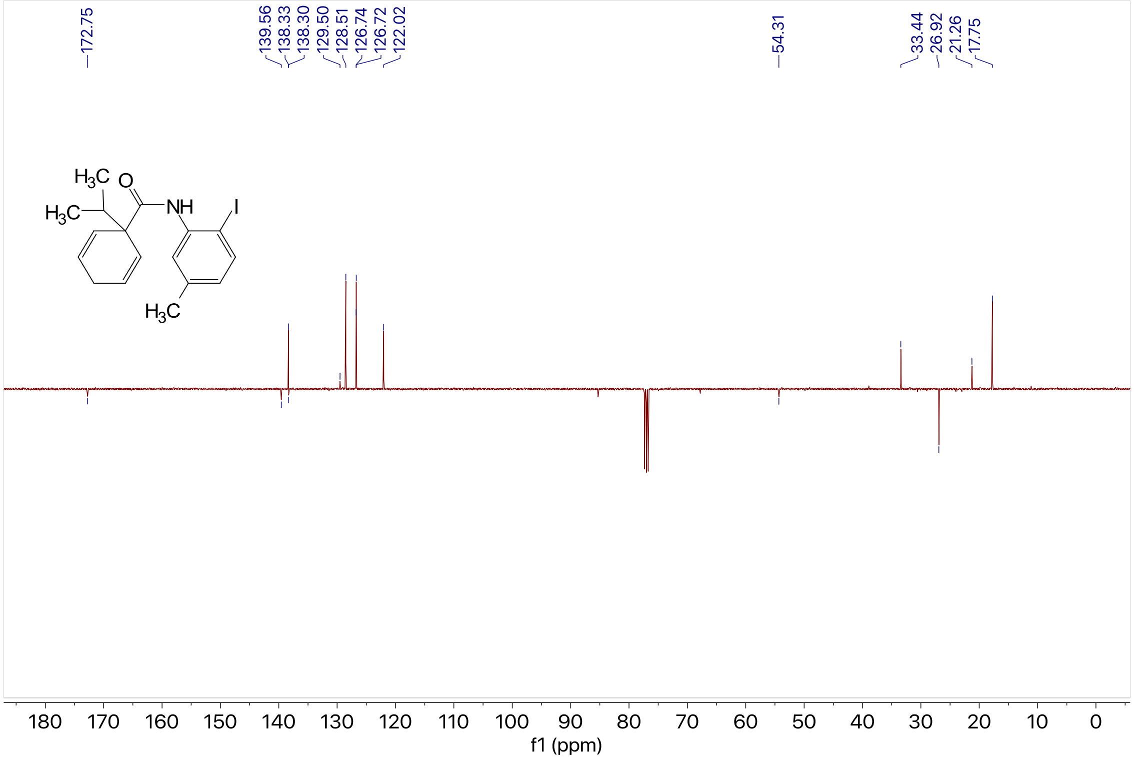

***N*-(2-Bromopyridin-3-yl)-1-methylcyclohexa-2,5-diene-1-carboxamide (S2m).**

^{1}*H NMR (400 MHz),* **S2m**

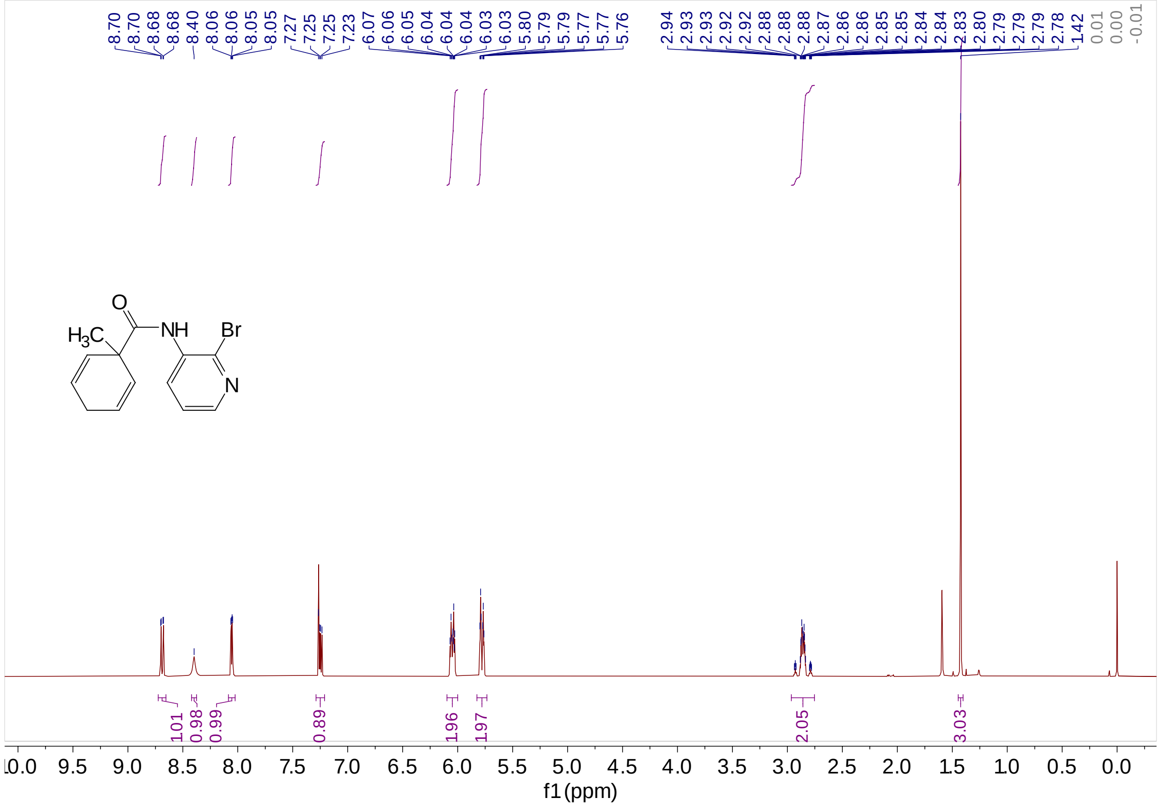

^{13}*C{^{1}H} NMR (101 MHz, $CDCl_3$),* **S2m**

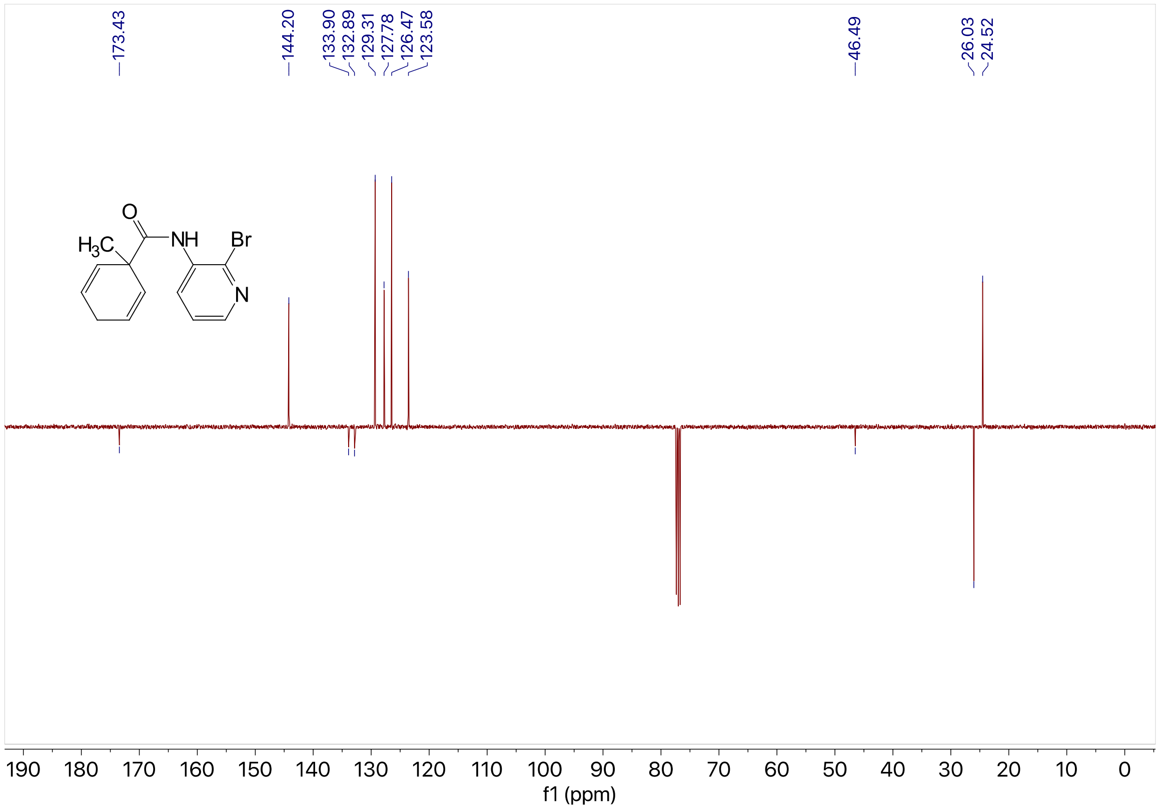

N-(3-Bromopyridin-2-yl)-1-methylcyclohexa-2,5-diene-1-carboxamide (S2n).

^{1}H NMR (400 MHz), **S2n**

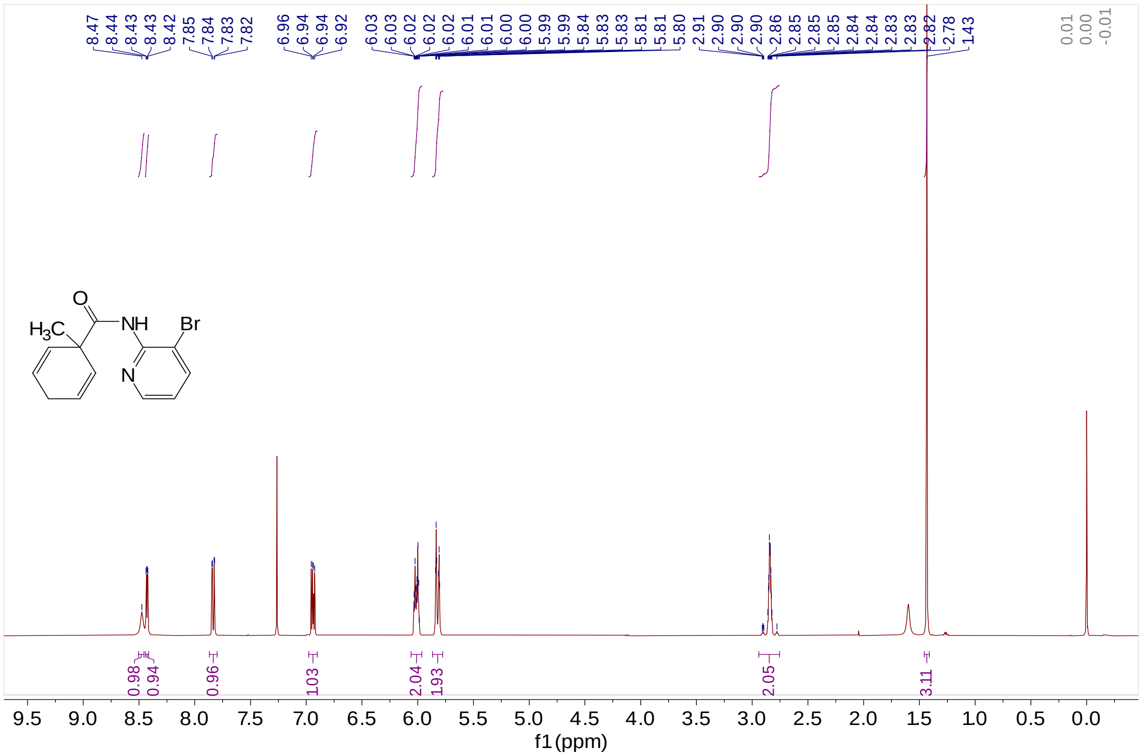

$^{13}C\{^{1}H\}$ NMR (101 MHz, $CDCl_3$), **S2n**

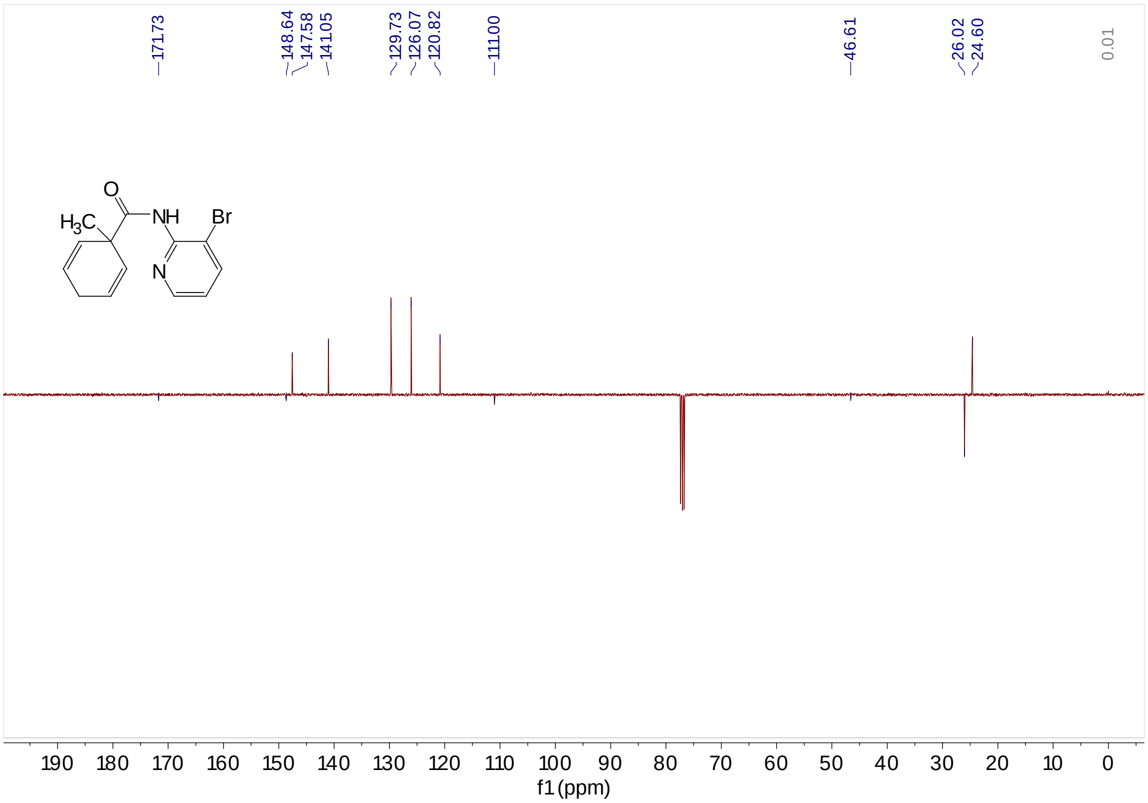

***N*-(3-Bromopyridin-2-yl)-1-ethylcyclohexa-2,5-diene-1-carboxamide (S2o).**

^{1}H NMR (400 MHz), **S2o**

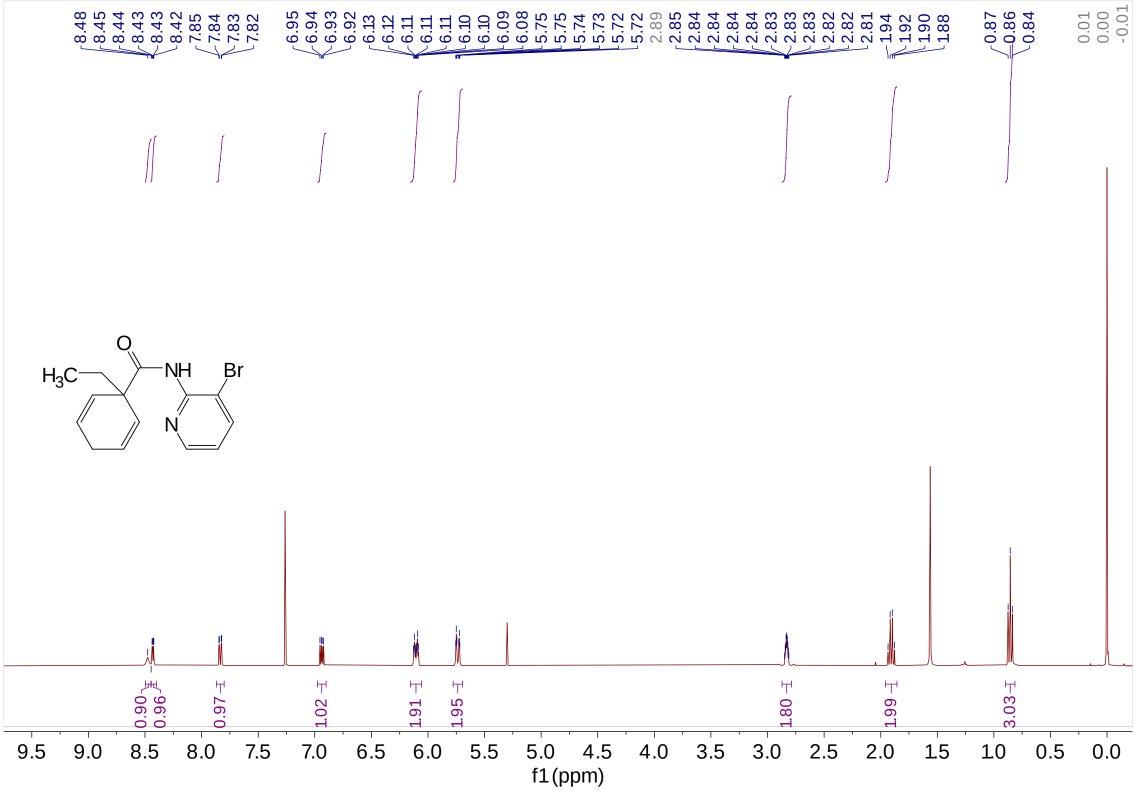

^{13}C{^{1}H} NMR (101 MHz, $CDCl_3$), **S2o**

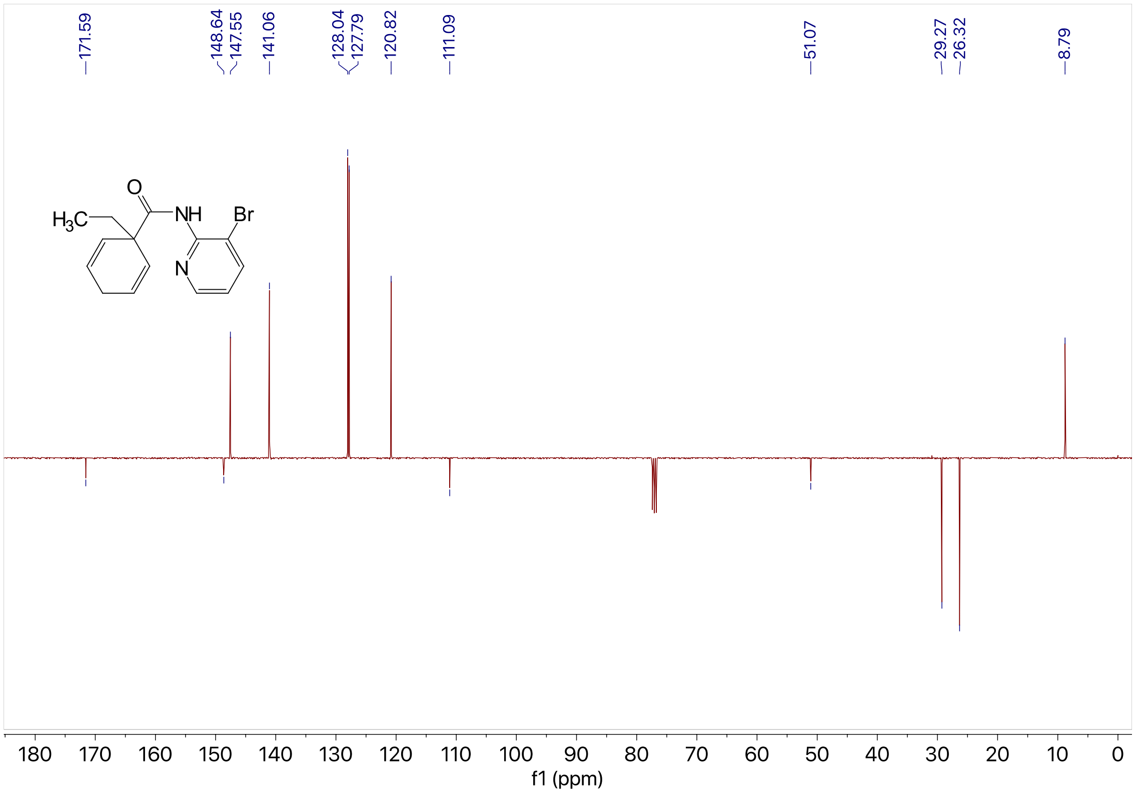

***N*-(3-Iodopyridin-2-yl)-1-ethylcyclohexa-2,5-diene-1-carboxamide (S2o-I).**

^{1}H NMR (400 MHz), **S2o-I**

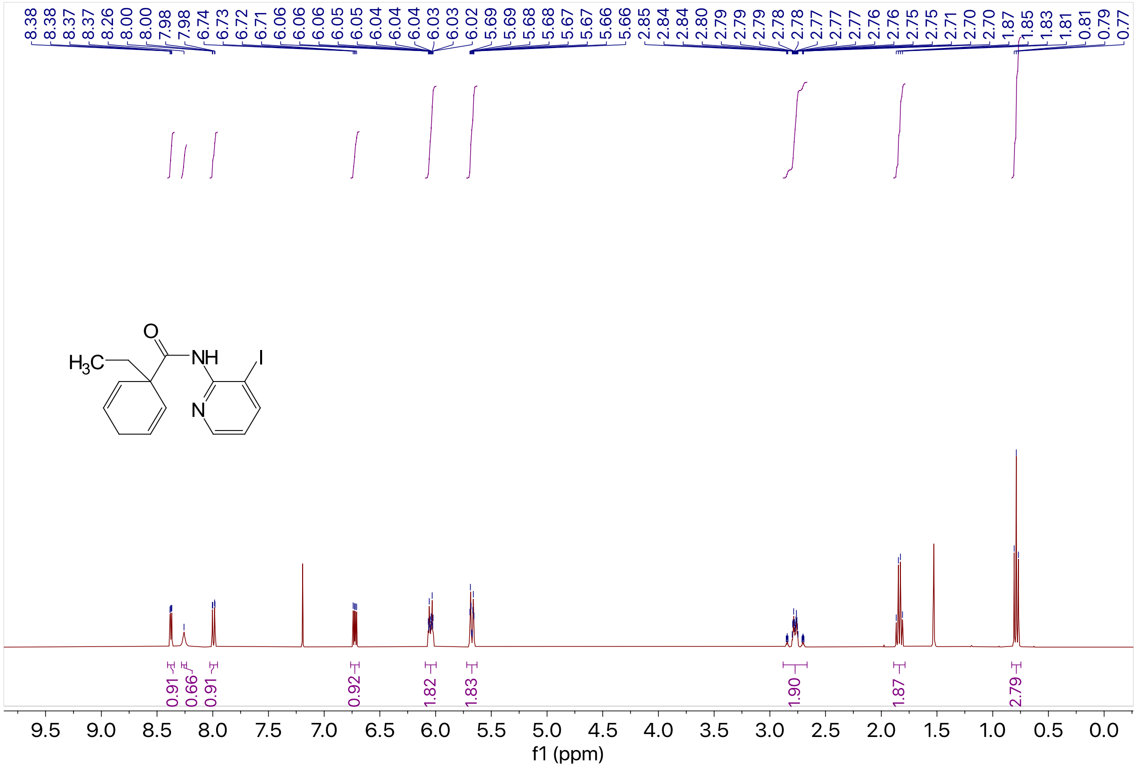

^{13}C{^{1}H} NMR (101 MHz, $CDCl_3$), **S2o-I**

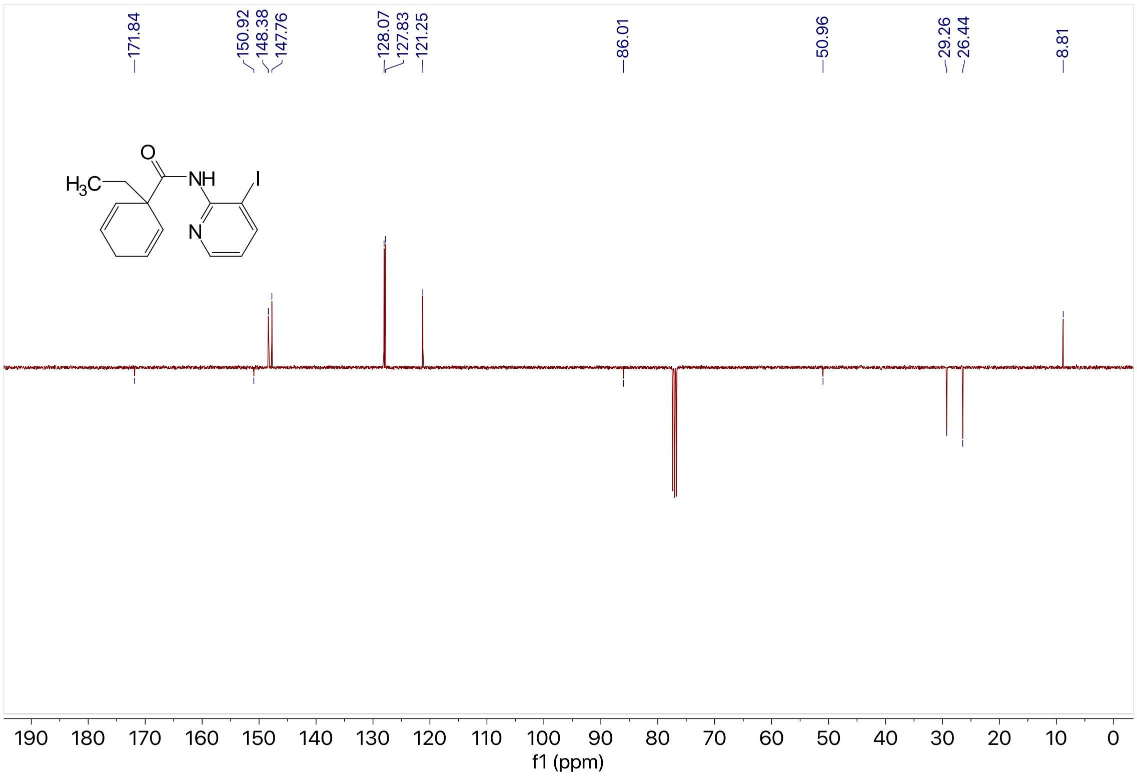

SI-Table 2: Secondary Amide Protection.

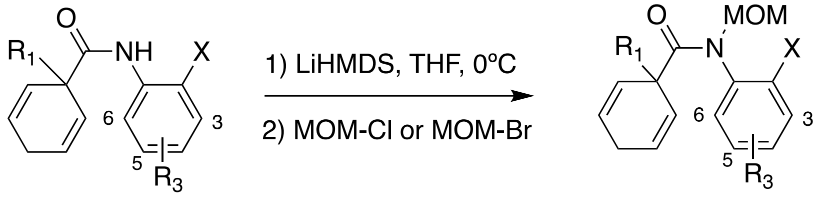

entry	R_1	R_3	X	yield (%)	compd
1	Me, **S2b**	H	Br	76	**1b**
2	Me, **S2b-I**	H	I	88	**1b-I**
3	Et, **S2c-I**	H	I	79	**1c-I**
4	*i*-Pr, **S2e**	H	Br	81	**1e**
5	*i*-Pr, **S2e-I**	H	I	72	**1e-I**
6	$-CH_2OCH_3$, **S2f-I**	H	I	90	**1f-I**
7	$-CH_2Ph$, **S2h**	H	Br	92	**1h**
8	$-CH_2Ph$, **S2h-I**	H	I	85	**1h-I**
9	*i*-Pr, **S2i**	4-F	Br	87	**1i**
10	*i*-Pr, **S2i-I**	4-F	I	96	**1i-I**
11	*i*-Pr, **S2j**	4-Cl	Br	79	**1j**
12	*i*-Pr, **S2j-I**	4-Cl	I	87	**1j-I**
13	*i*-Pr, **S2k**	4-Me	Br	77	**1k**
14	*i*-Pr, **S2l-I**	5-Me	I	84	**1l-I**
15	Me, **S2m**	C_3=N	Br	84	**1m**
16	Me, **S2n**	C_6=N	Br	65	**1n**
17	Et, **S2o**	C_6=N	Br	78	**1o**
18	Et, **S2o-I**	C_6=N	I	50	**1o-I**

NMR spectra for N-methoxymethyl (MOM) amide products

_N_-(2-Bromophenyl)-_N_-(methoxymethyl)-1-methylcyclohexa-2,5-diene-1-carboxamide (1b).

^{1}H NMR (400 MHz), **1b**

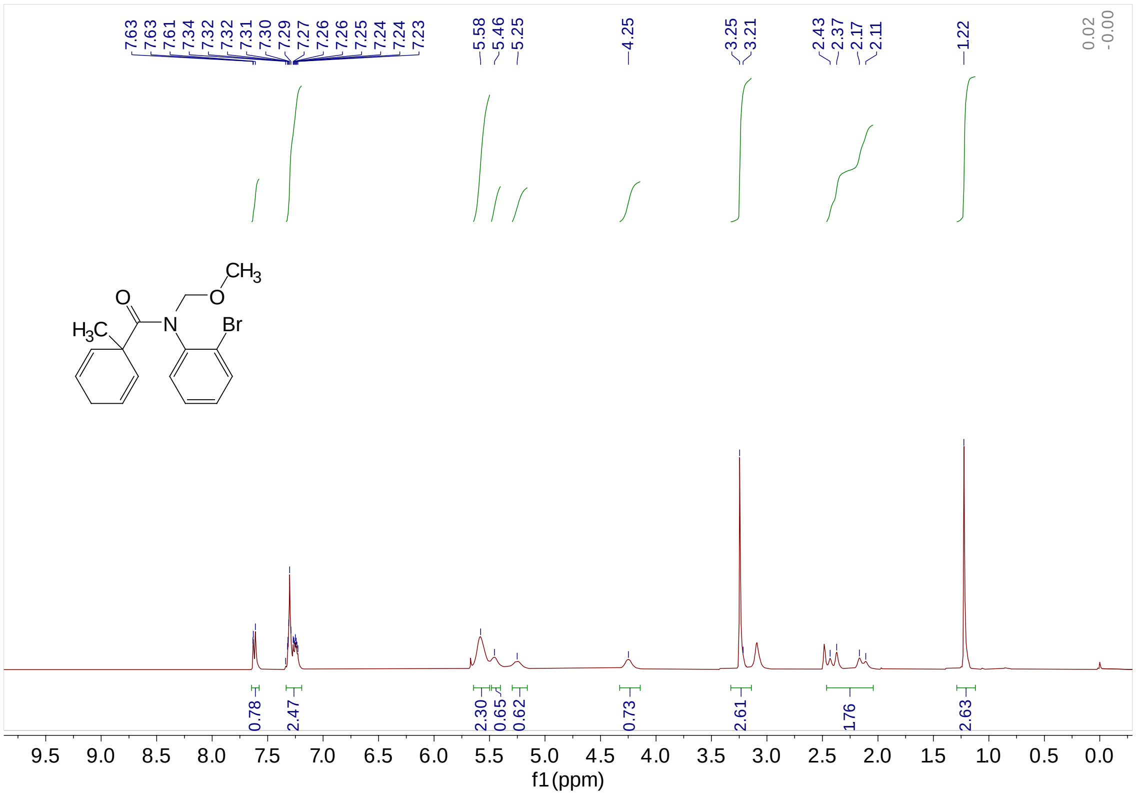

$^{13}C\{^1H\}$ NMR (101 MHz, $CDCl_3$), **1b**

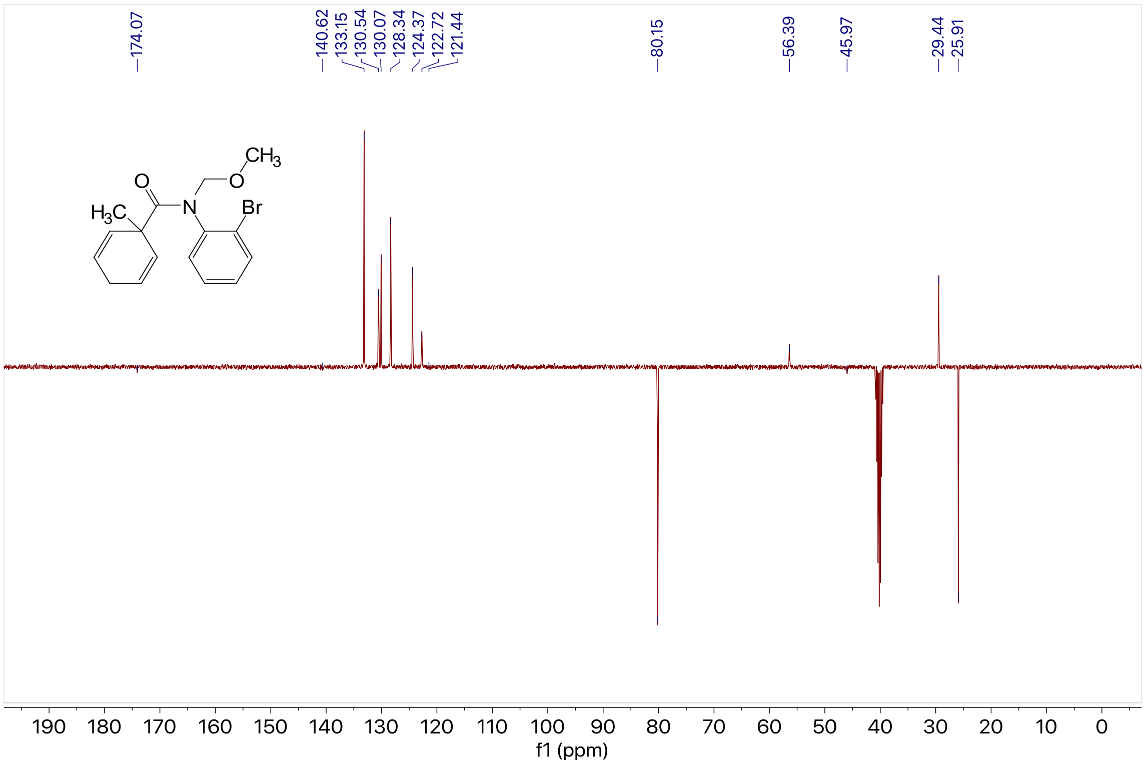

N-(2-Iodophenyl)-N-(methoxymethyl)-1-methylcyclohexa-2,5-diene-1-carboxamide (1b-I).

^{1}H NMR (400 MHz), **1b-I**

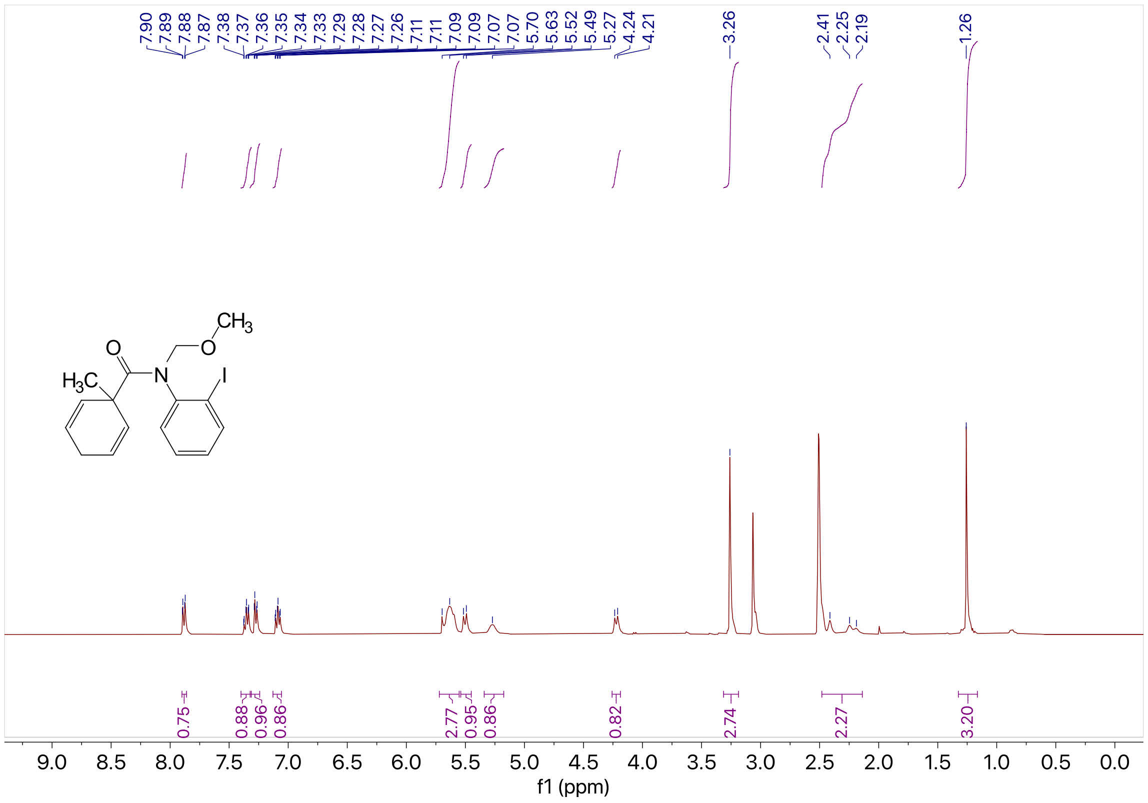

$^{13}C\{^{1}H\}$ NMR (101 MHz, $CDCl_3$), **1b-I**

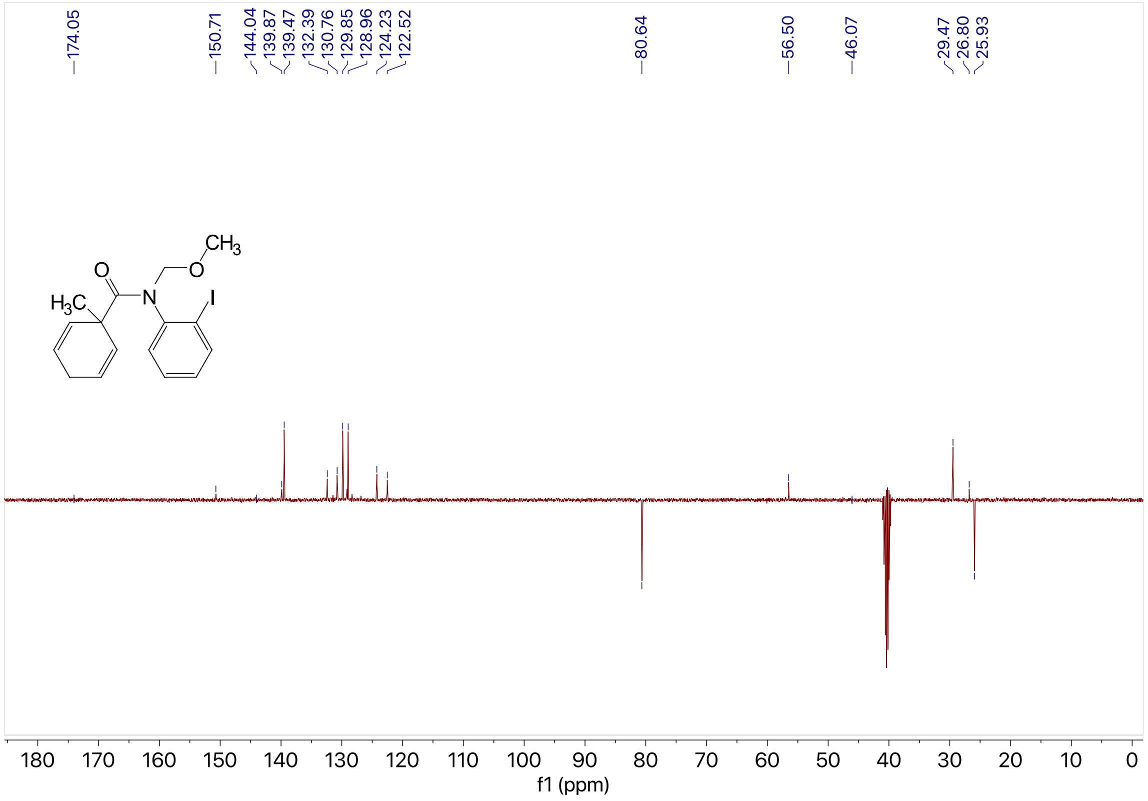

1-Ethyl-*N*-(2-iodophenyl)-*N*-(methoxymethyl)cyclohexa-2,5-diene-1-carboxamide (1c-I).

^{1}H NMR (400 MHz), **1c-I**

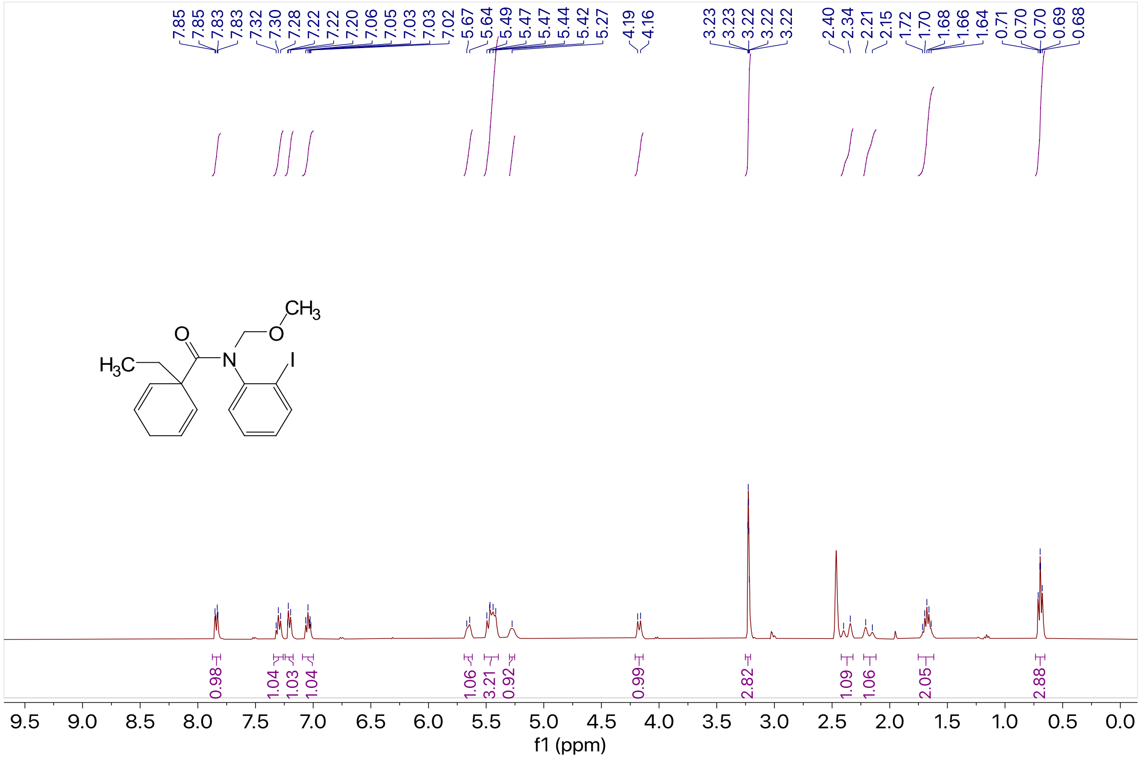

^{13}C{^{1}H} NMR (101 MHz, $CDCl_3$), **1c-I**

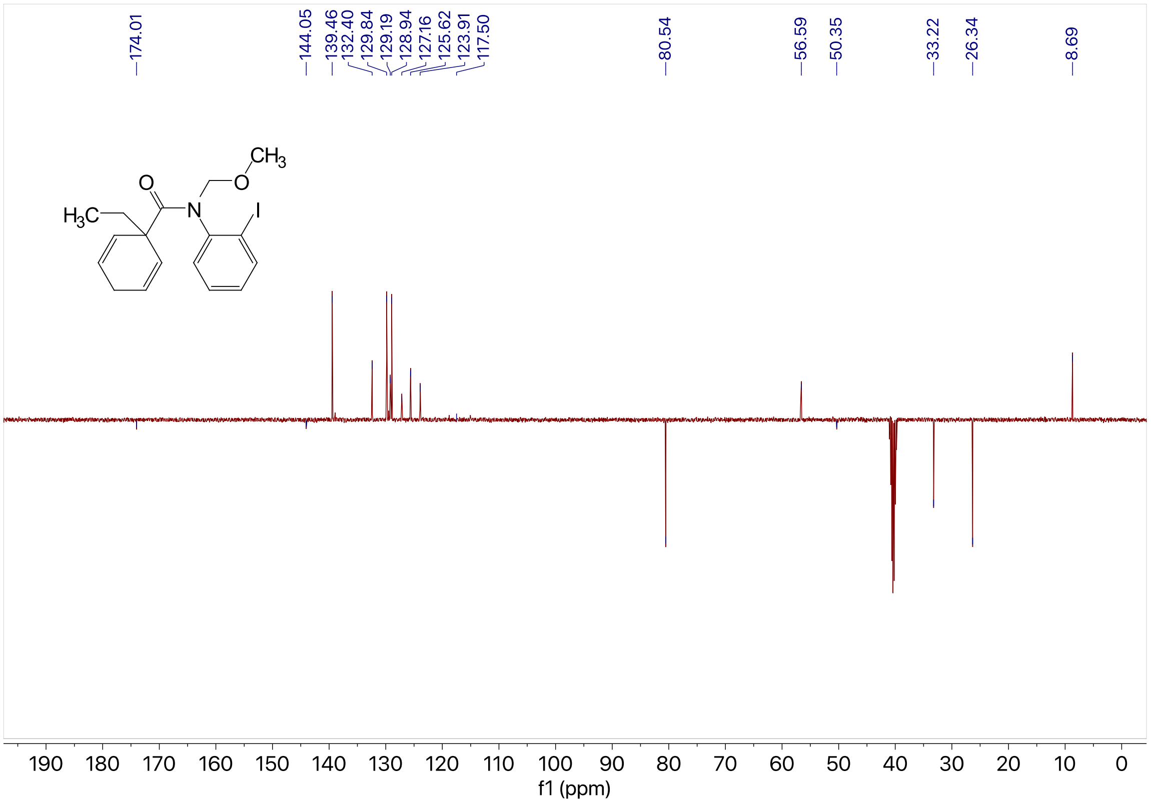

N-(2-Bromophenyl)-1-isopropyl-N-(methoxymethyl)cyclohexa-2,5-diene-1-carboxamide (1e).

^{1}H NMR (400 MHz), **1e**

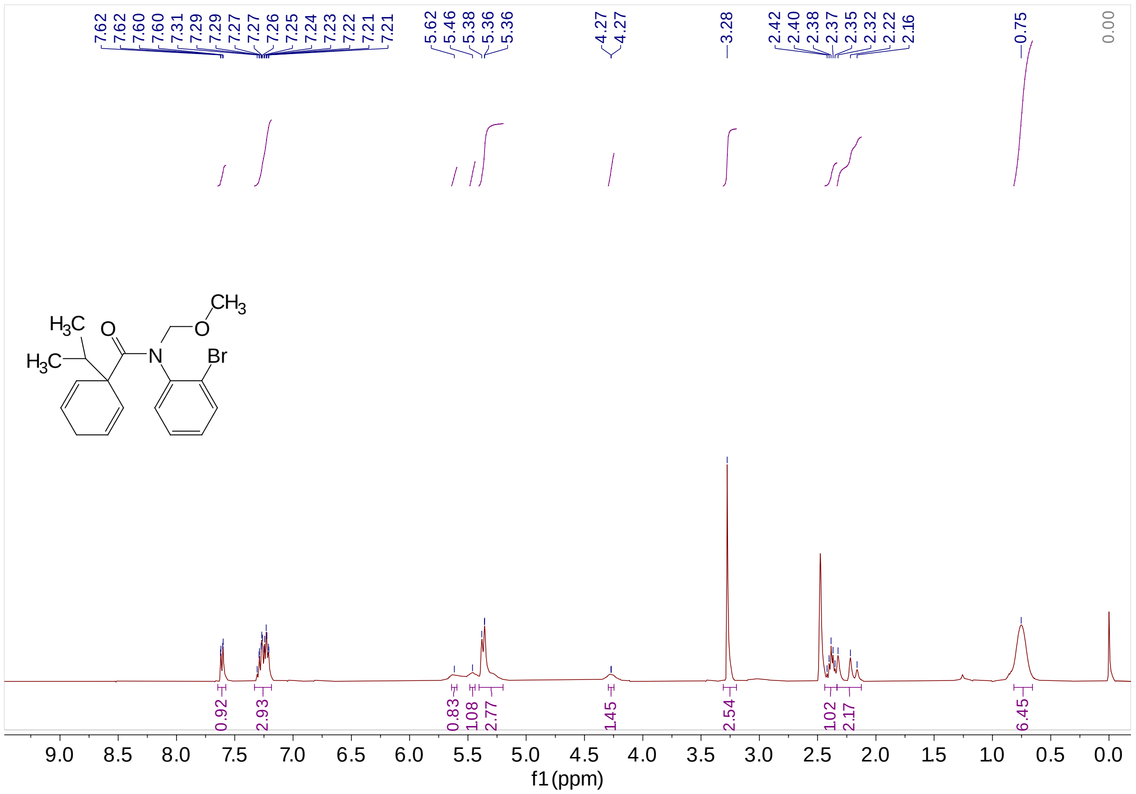

$^{13}C\{^{1}H\}$ NMR (101 MHz, $CDCl_3$), **1e**

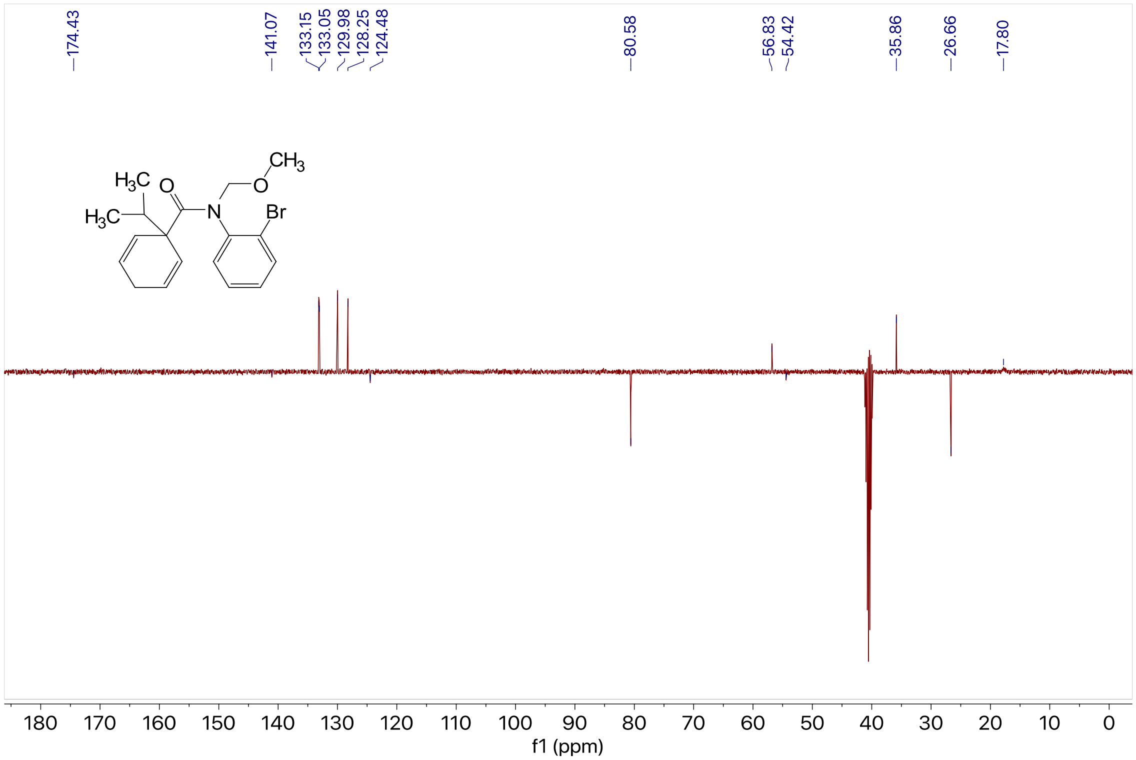

***N*-(2-Iodophenyl)-1-isopropyl-*N*-(methoxymethyl)cyclohexa-2,5-diene-1-carboxamide (1e-I).**

^{1}H NMR (400 MHz), **1e-I**

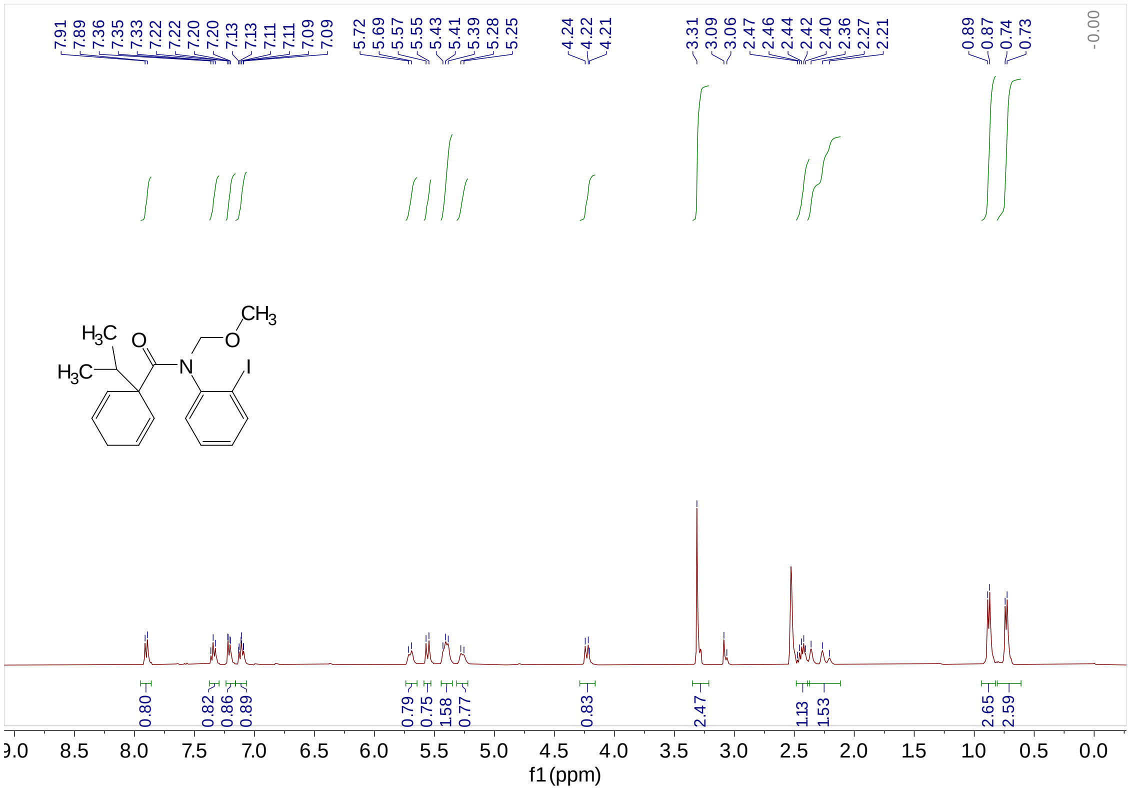

^{13}C{^{1}H} NMR (101 MHz, $CDCl_3$), **1e-I**

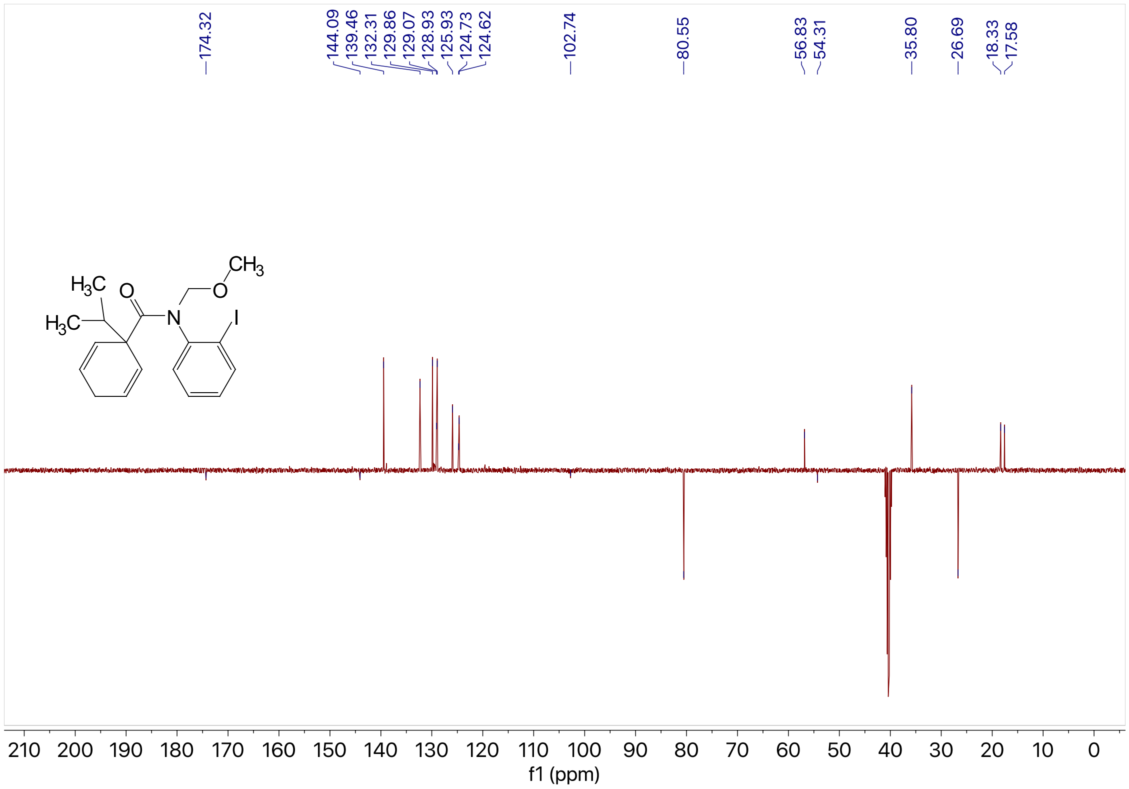

***N*-(2-Iodophenyl)-*N*,1-bis(methoxymethyl)cyclohexa-2,5-diene-1-carboxamide (1f-I).**
^{1}H NMR (400 MHz), **1f-I**

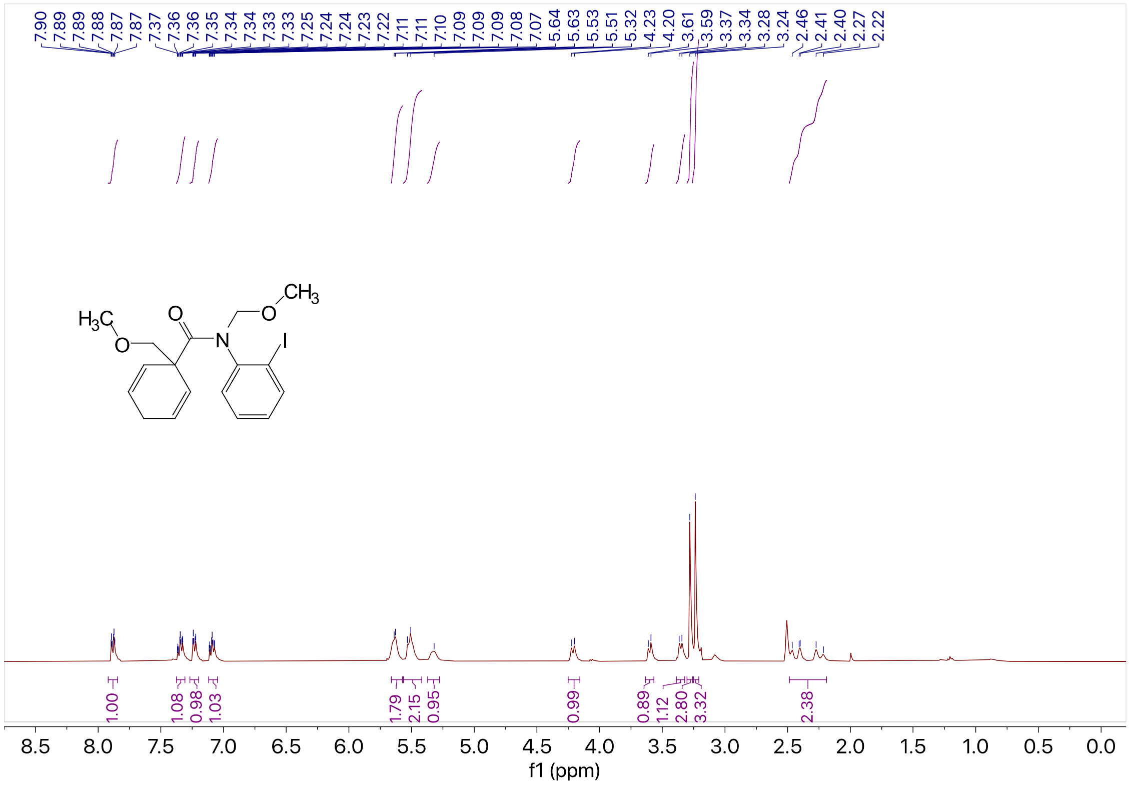

^{13}C{^{1}H} NMR (101 MHz, $CDCl_3$), **1f-I**

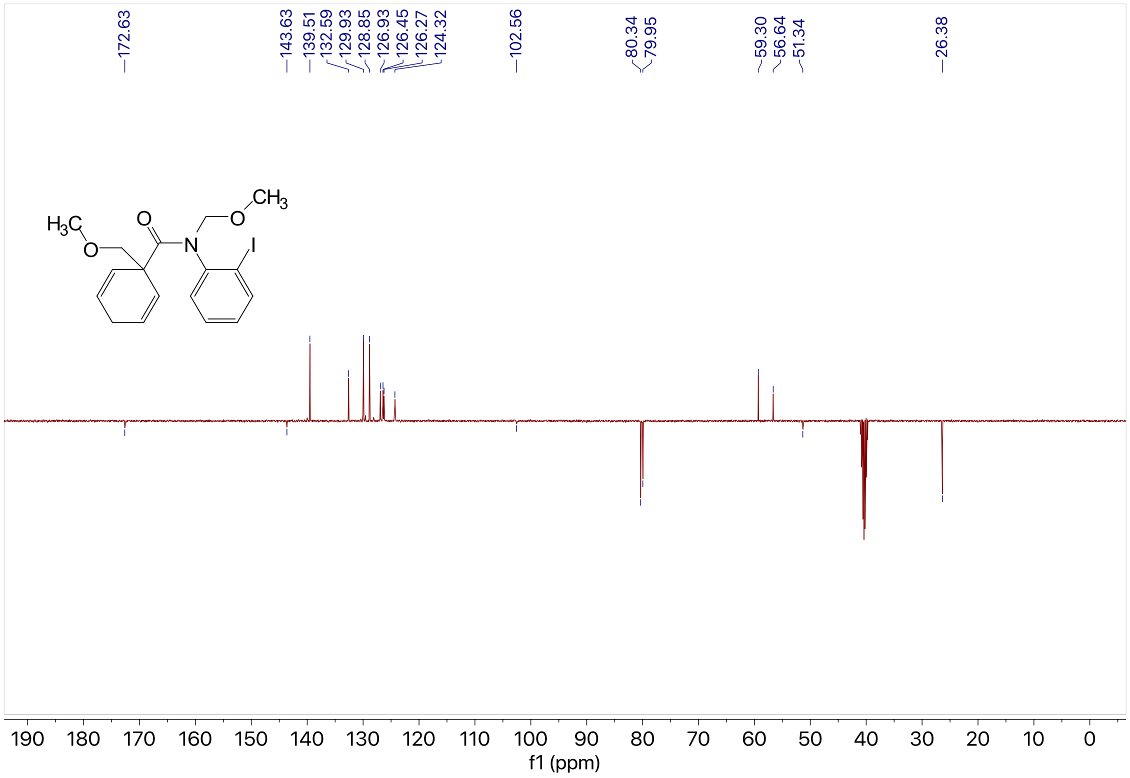

1-Benzyl-*N*-(2-bromophenyl)-*N*-(methoxymethyl)cyclohexa-2,5-diene-1-carboxamide (1h).

^{1}H *NMR (400 MHz),* **1h**

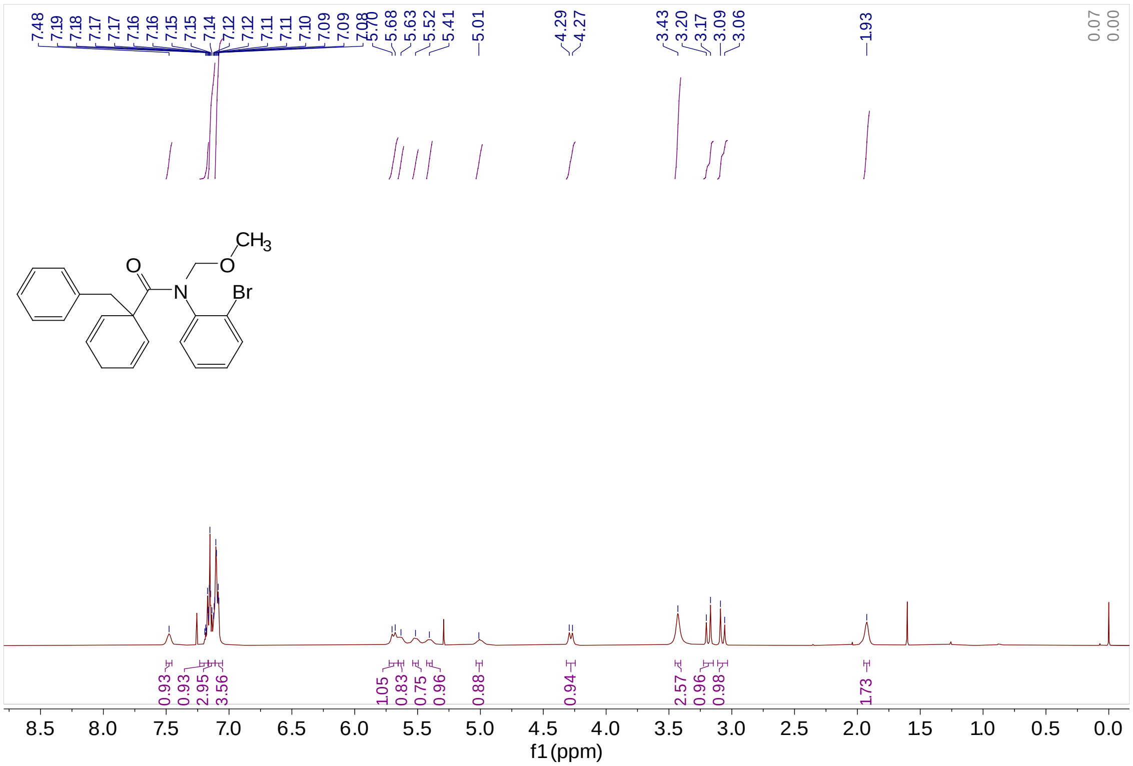

$^{13}C\{^{1}H\}$ *NMR (101 MHz, $CDCl_3$),* **1h**

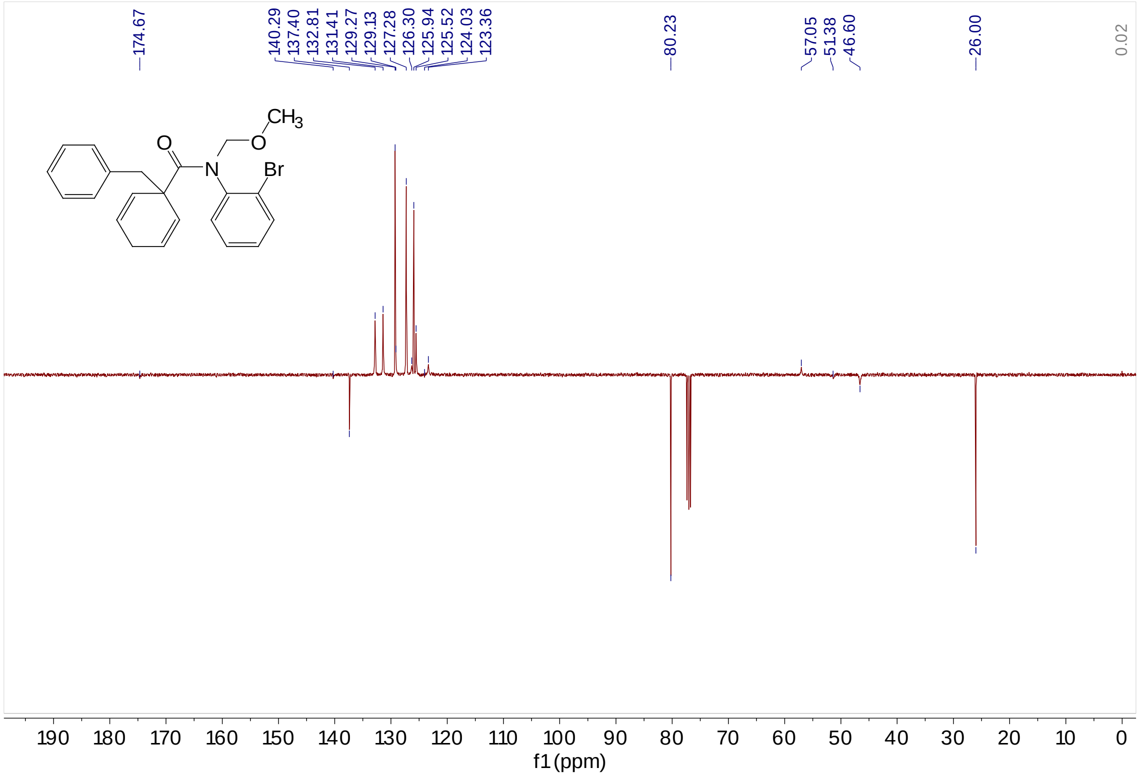

1-Benzyl-*N*-(2-iodophenyl)-*N*-(methoxymethyl)cyclohexa-2,5-diene-1-carboxamide (1h-I).

^{1}H NMR (400 MHz), **1h-I**

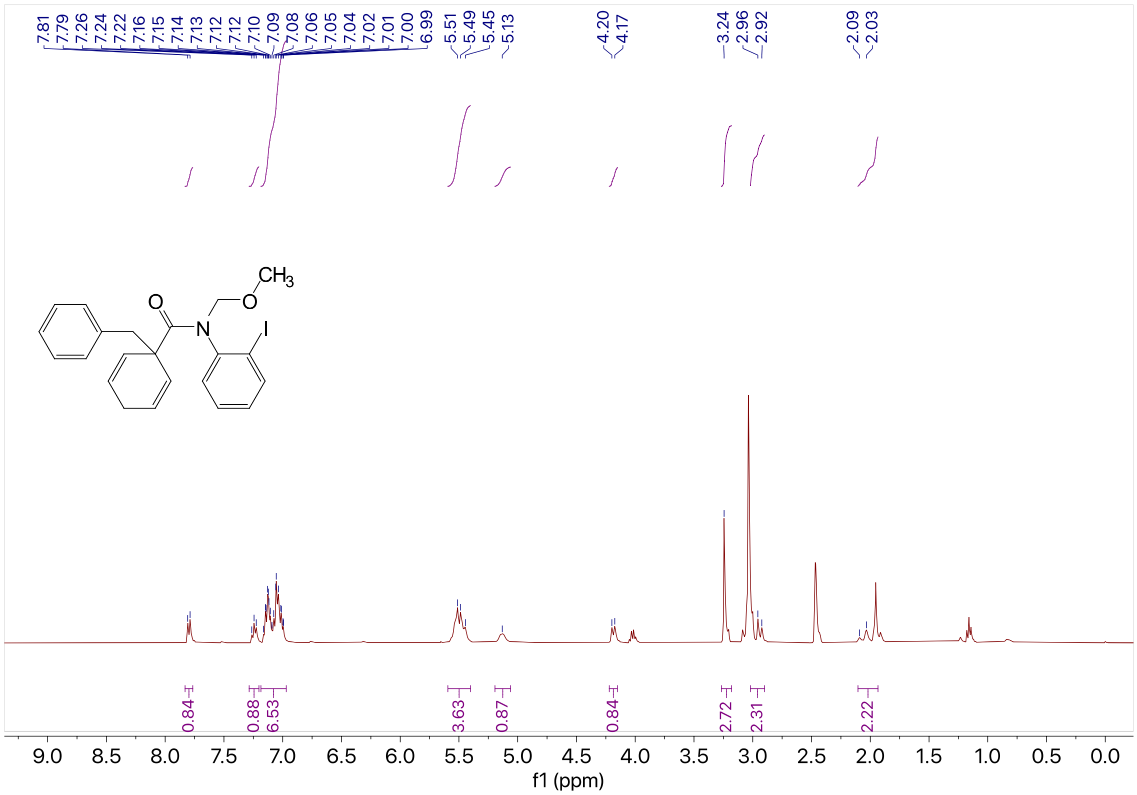

$^{13}C\{^{1}H\}$ NMR (101 MHz, $CDCl_3$), **1h-I**

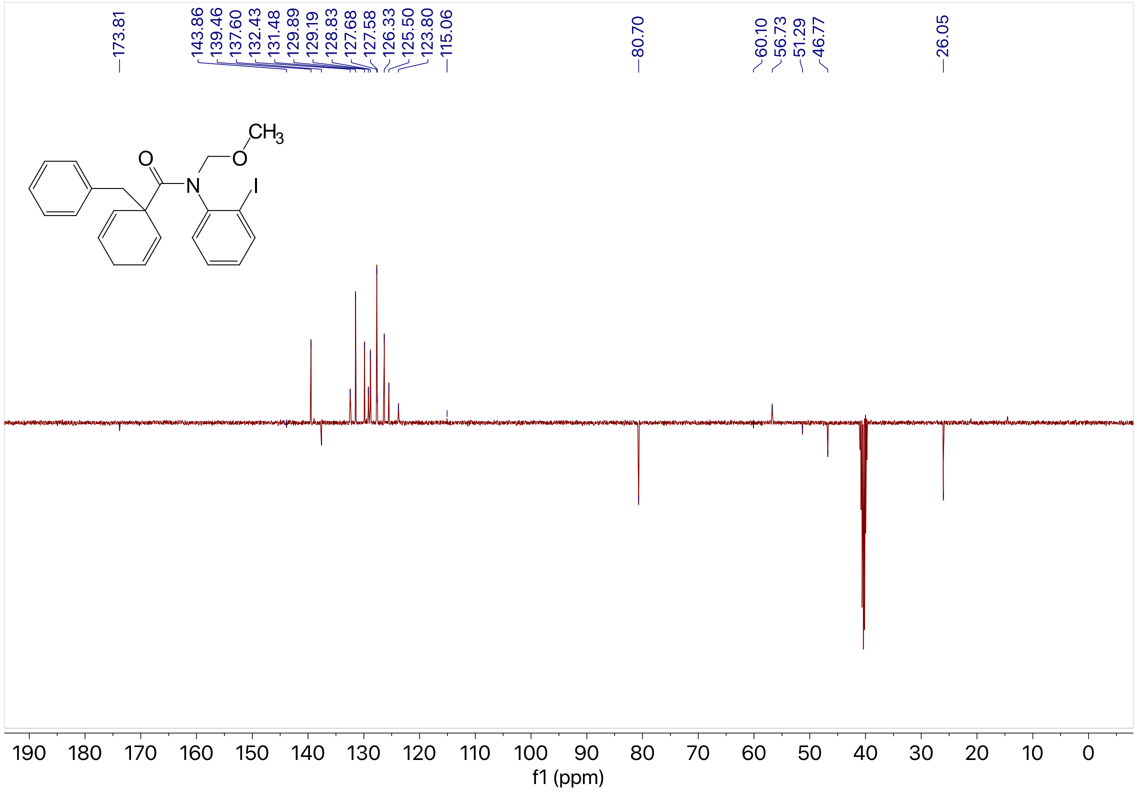

N-(2-Bromo-4-fluorophenyl)-1-isopropyl-N-(methoxymethyl)cyclohexa-2,5-diene-1-carboxamide (1i).

^{1}H NMR (400 MHz), **1i**

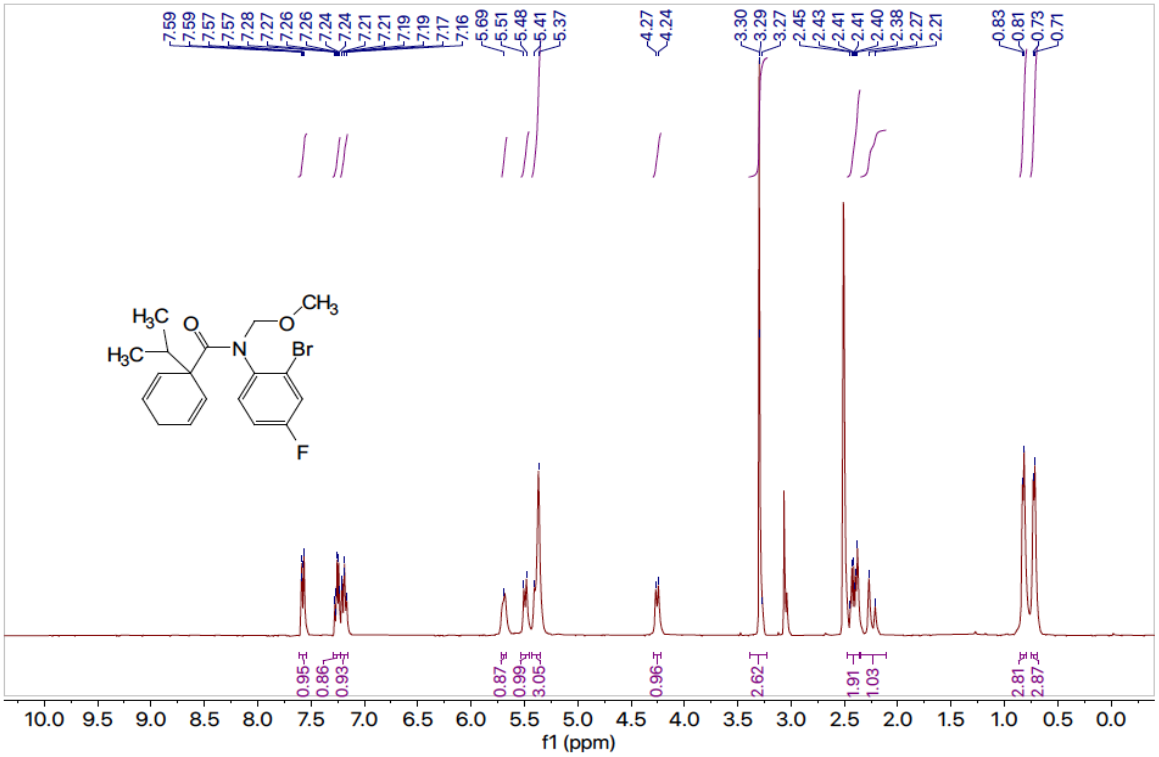

$^{13}C\{^{1}H\}$ NMR (101 MHz, $CDCl_3$), **1i**

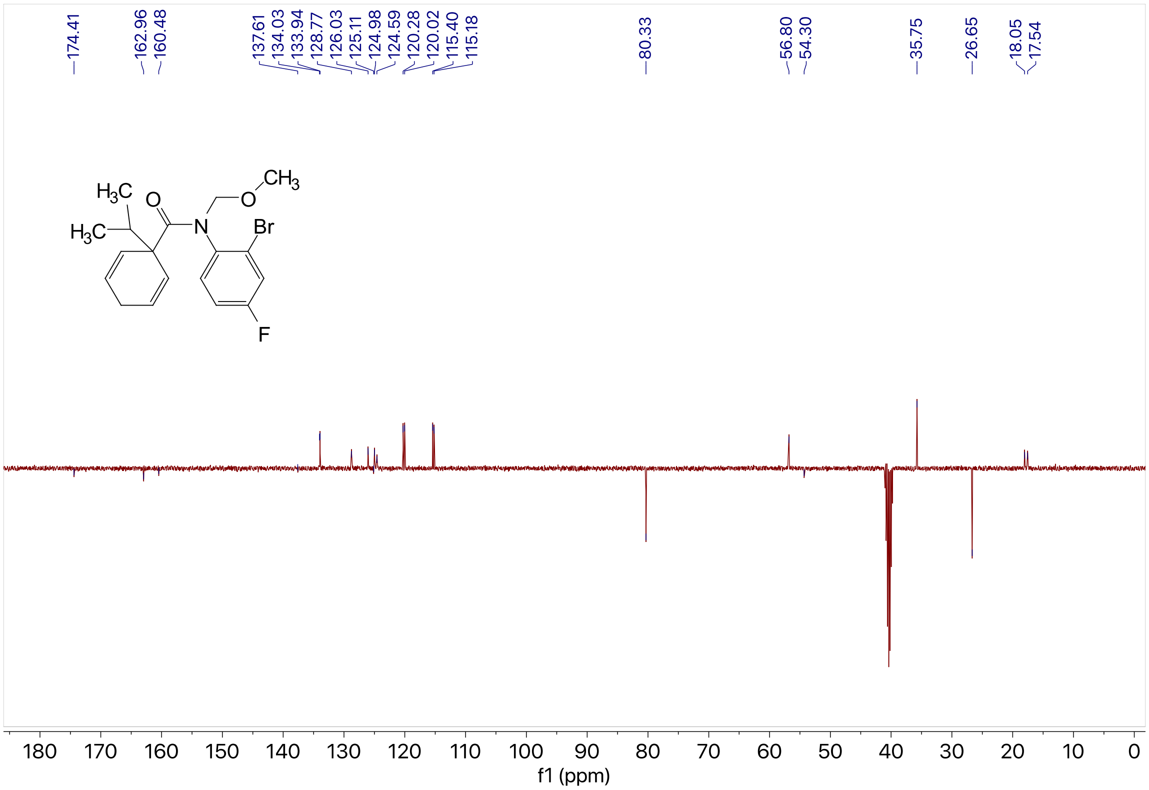

^{19}F NMR (376 MHz), **1i**

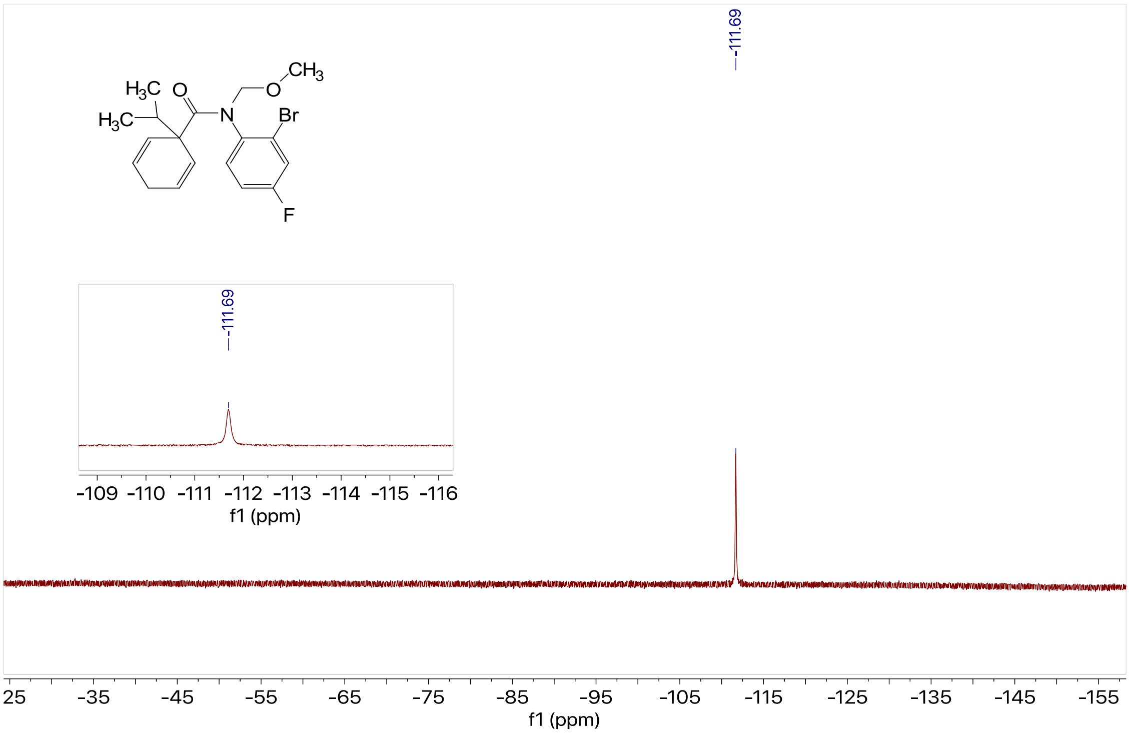

***N*-(4-Fluoro-2-iodophenyl)-1-isopropyl-*N*-(methoxymethyl)cyclohexa-2,5-diene-1-carboxamide (1i-I).**

^{1}H NMR (400 MHz), **1i-I**

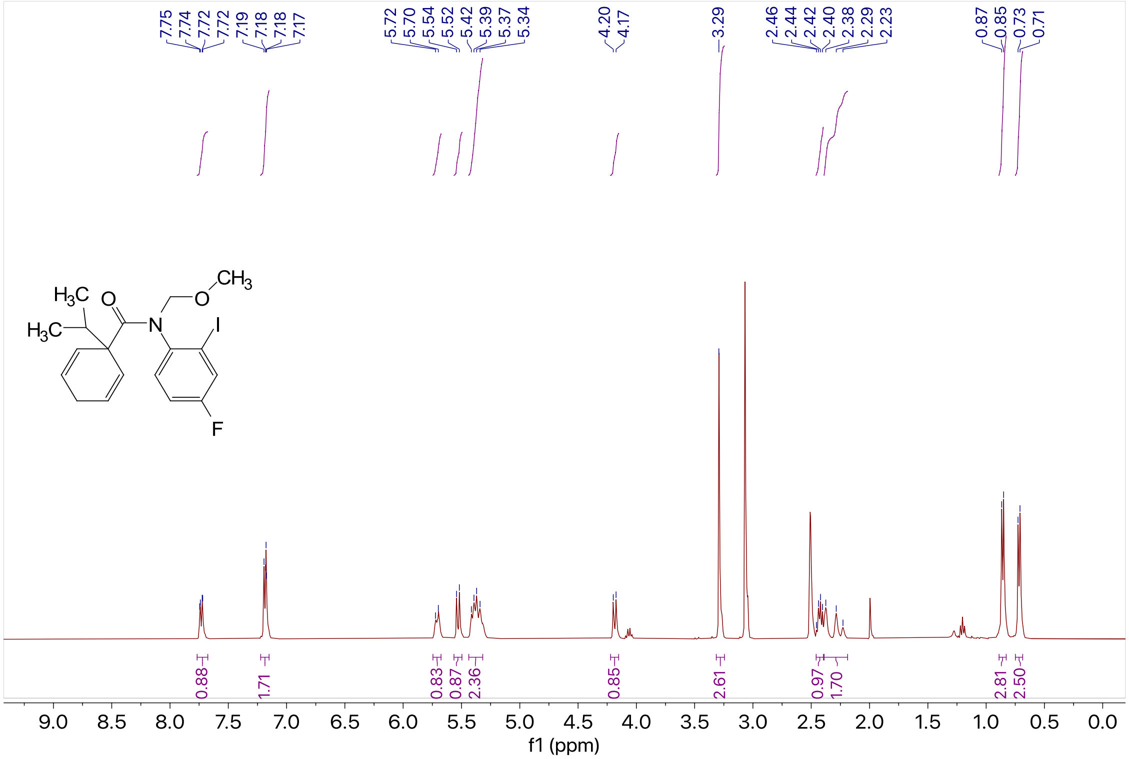

$^{13}C\{^{1}H\}$ *NMR (101 MHz, $CDCl_3$),* **1i-I**

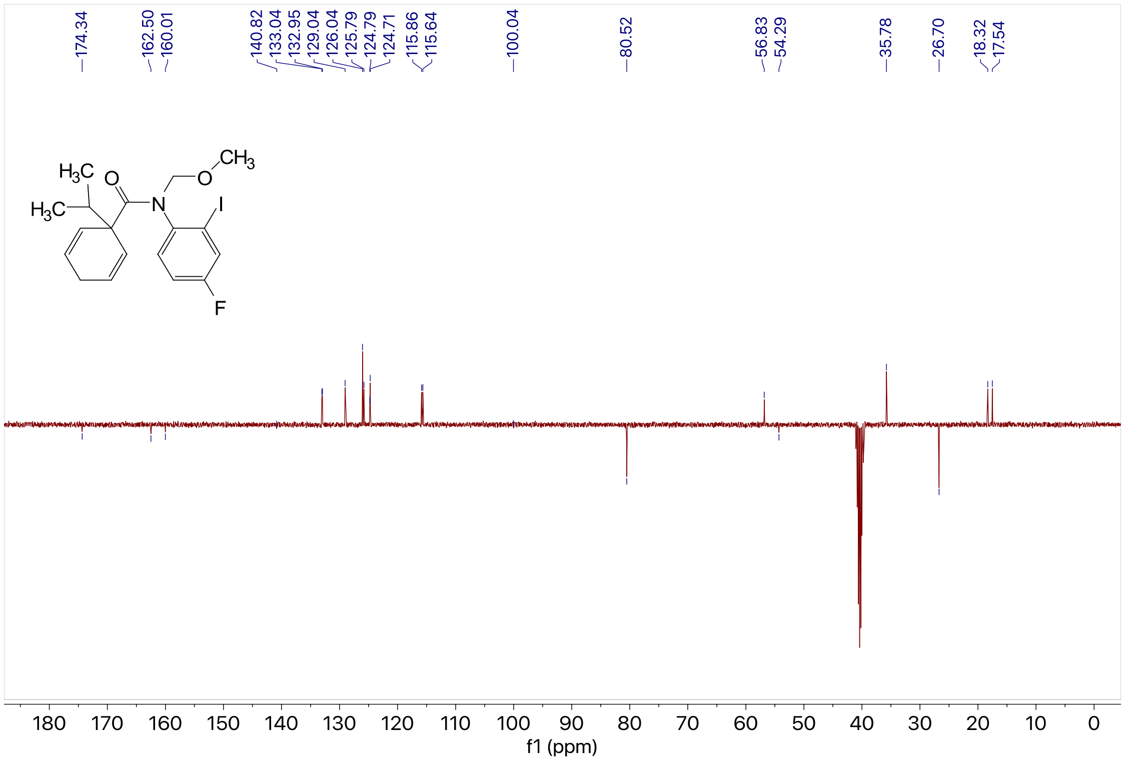

^{19}F *NMR (376 MHz),* **1i-I**

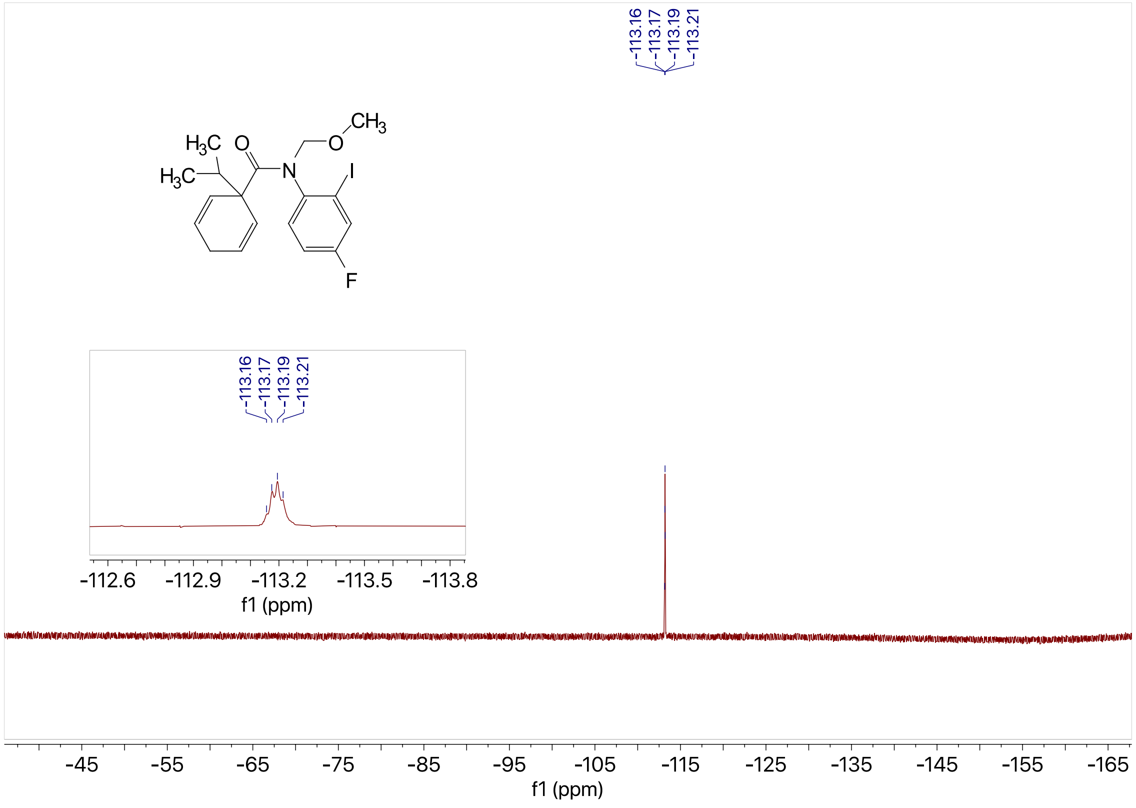

_N_-(2-Bromo-4-chlorophenyl)-1-isopropyl-_N_-(methoxymethyl)cyclohexa-2,5-diene-1-carboxamide (1j).

^{1}H NMR (400 MHz), **1j**

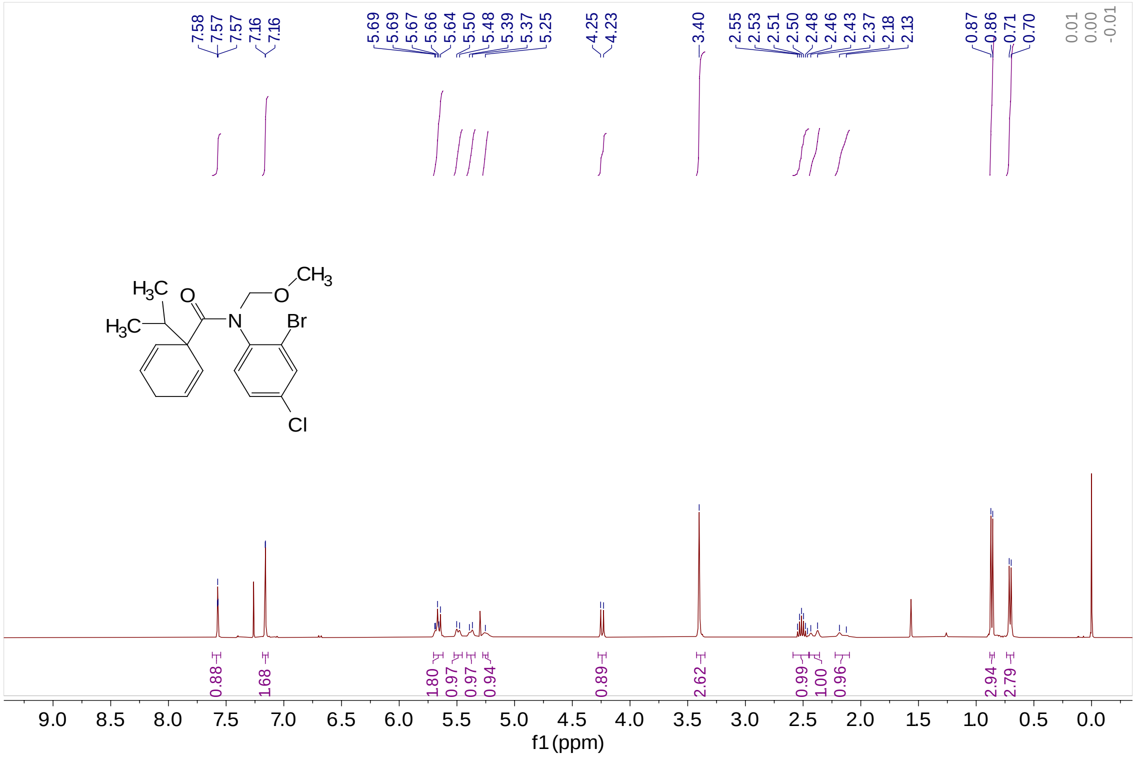

^{13}C{^{1}H} NMR (101 MHz, $CDCl_3$), **1j**

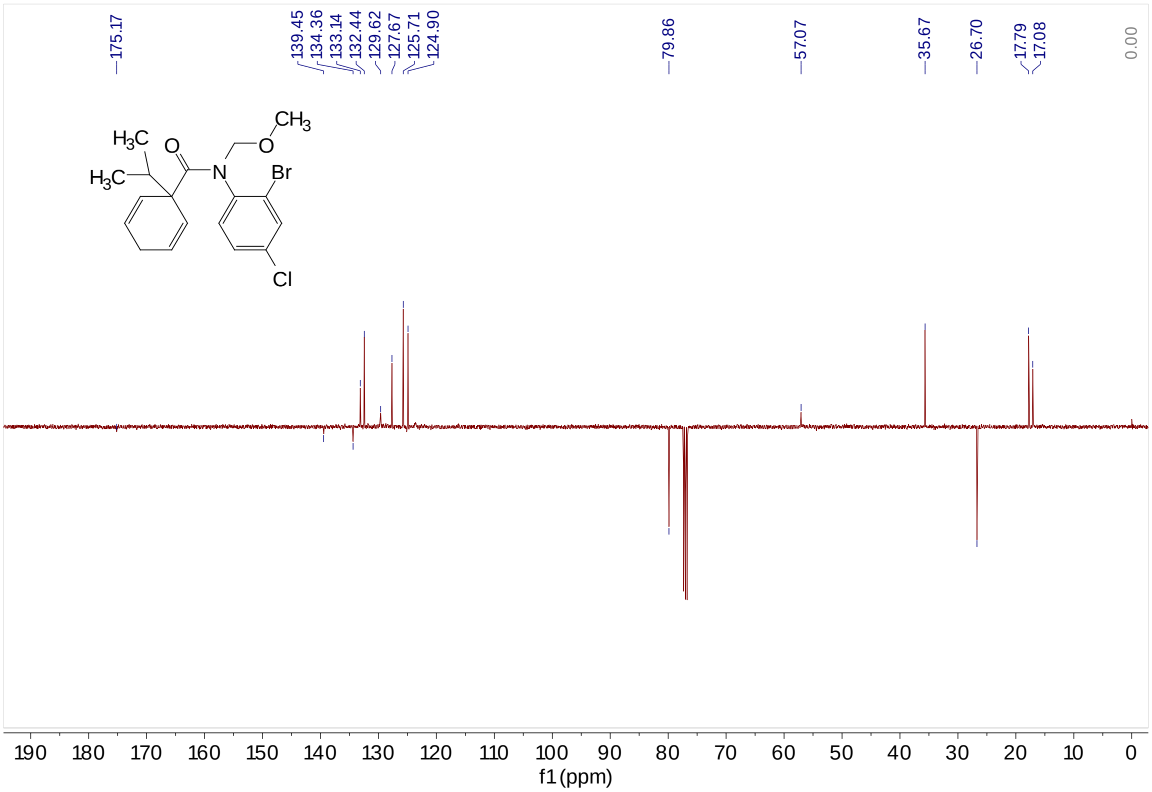

N-(4-Chloro-2-iodophenyl)-1-isopropyl-N-(methoxymethyl)cyclohexa-2,5-diene-1-carboxamide (1j-I).

^{1}H NMR (400 MHz), **1j-I**

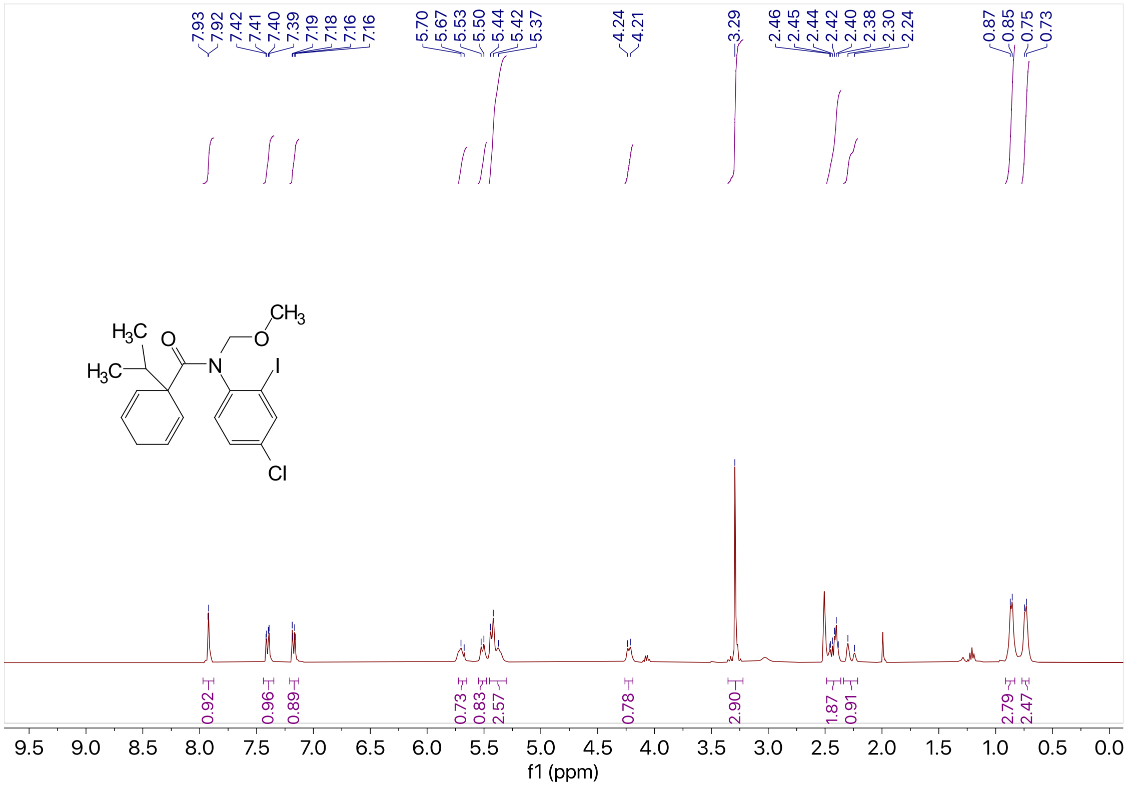

^{13}C{^{1}H} NMR (101 MHz, $CDCl_3$), **1j-I**

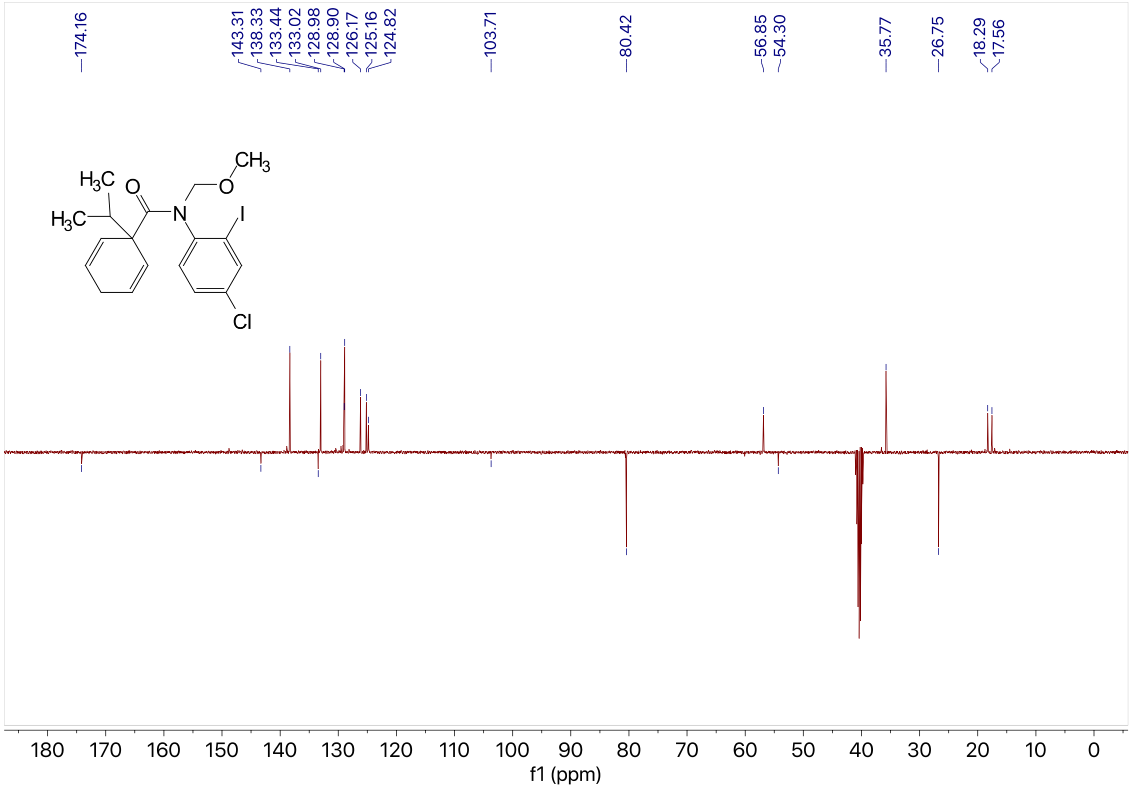

***N*-(2-Bromo-4-methylphenyl)-1-isopropyl-*N*-(methoxymethyl)cyclohexa-2,5-diene-1-carboxamide (1k).**

^{1}H NMR (400 MHz), **1k**

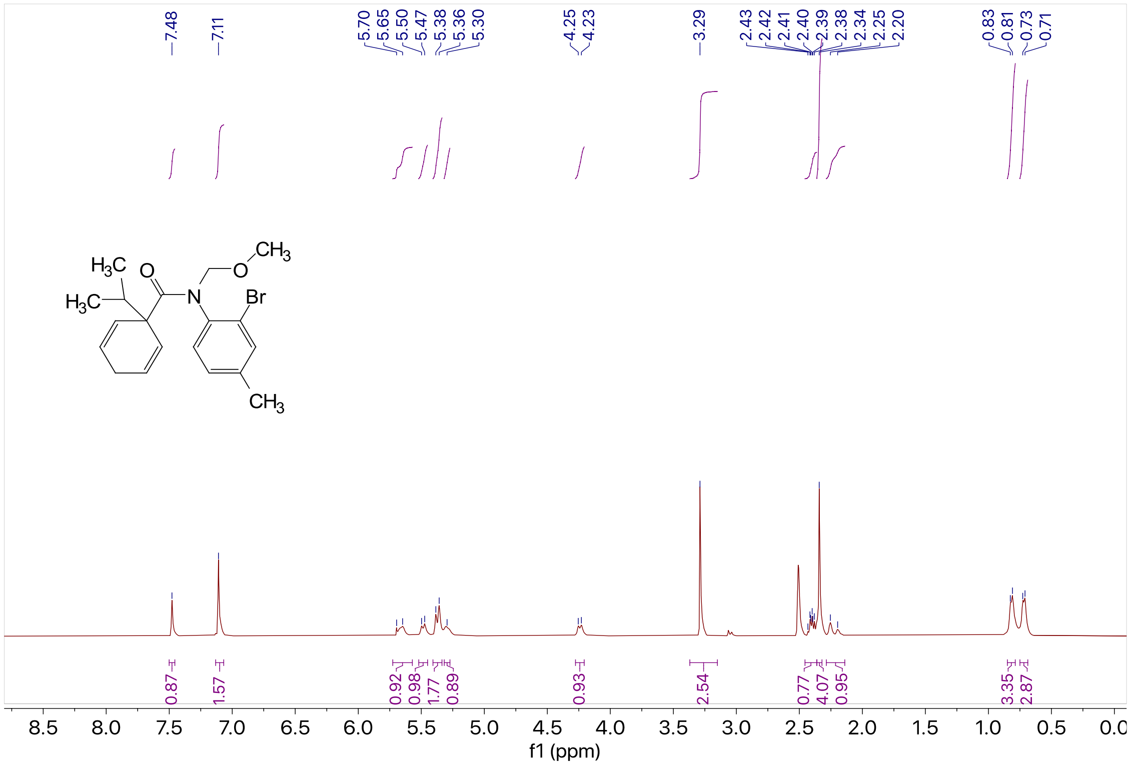

^{13}C{^{1}H} NMR (101 MHz, $CDCl_3$), **1k**

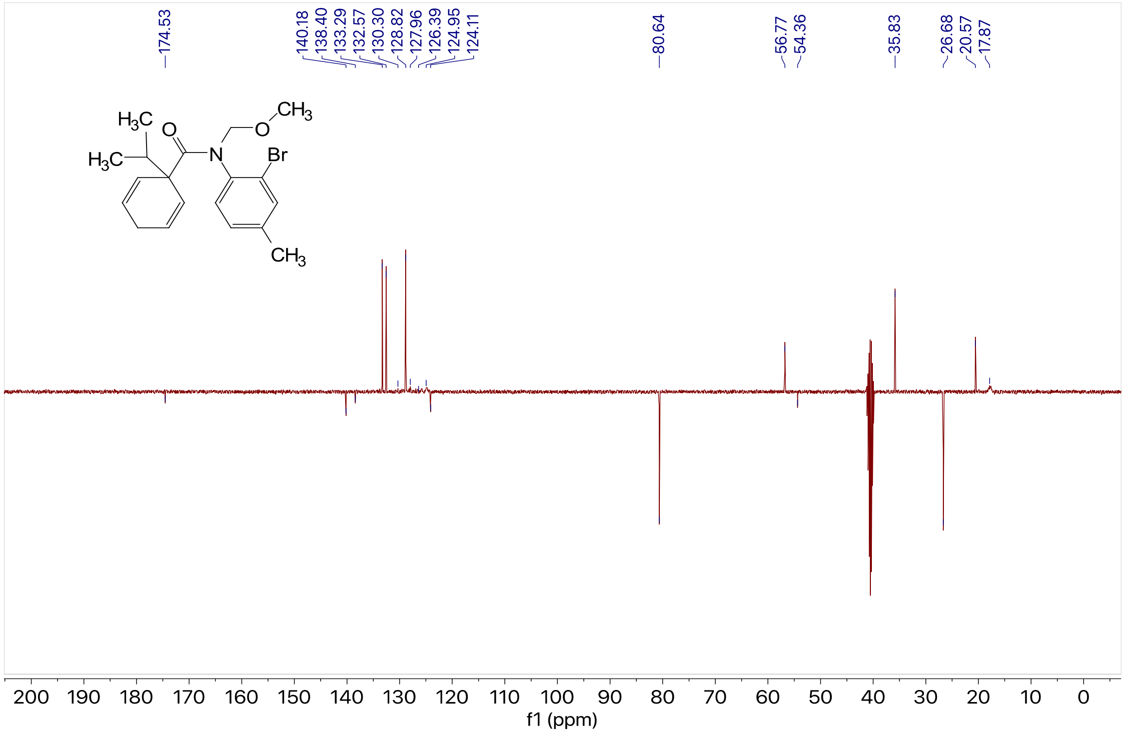

N-(2-Iodo-5-methylphenyl)-1-isopropyl-N-(methoxymethyl)cyclohexa-2,5-diene-1-carboxamide (1l-I).

^{1}H NMR (400 MHz), **1l-I**

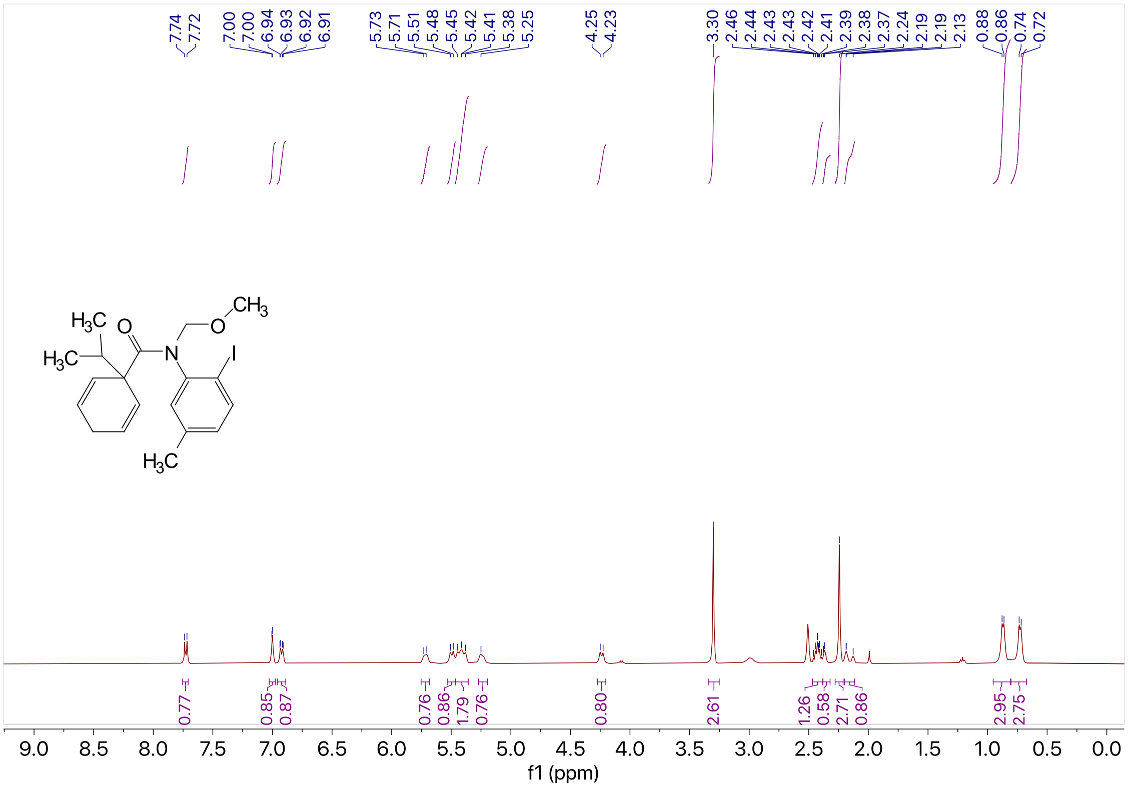

^{13}C{^{1}H} NMR (101 MHz, $CDCl_3$), **1l-I**

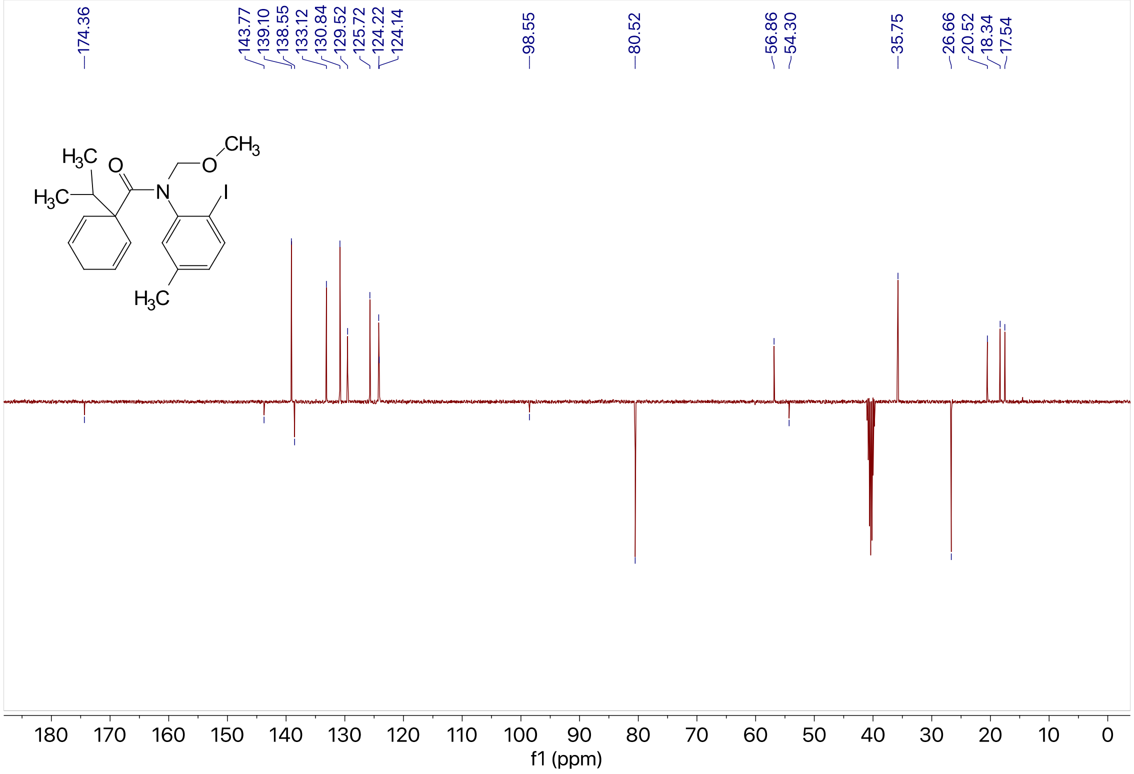

N-(2-Bromopyridin-3-yl)-N-(methoxymethyl)-1-methylcyclohexa-2,5-diene-1-carboxamide (1m).

^{1}H NMR (400 MHz), **1m**

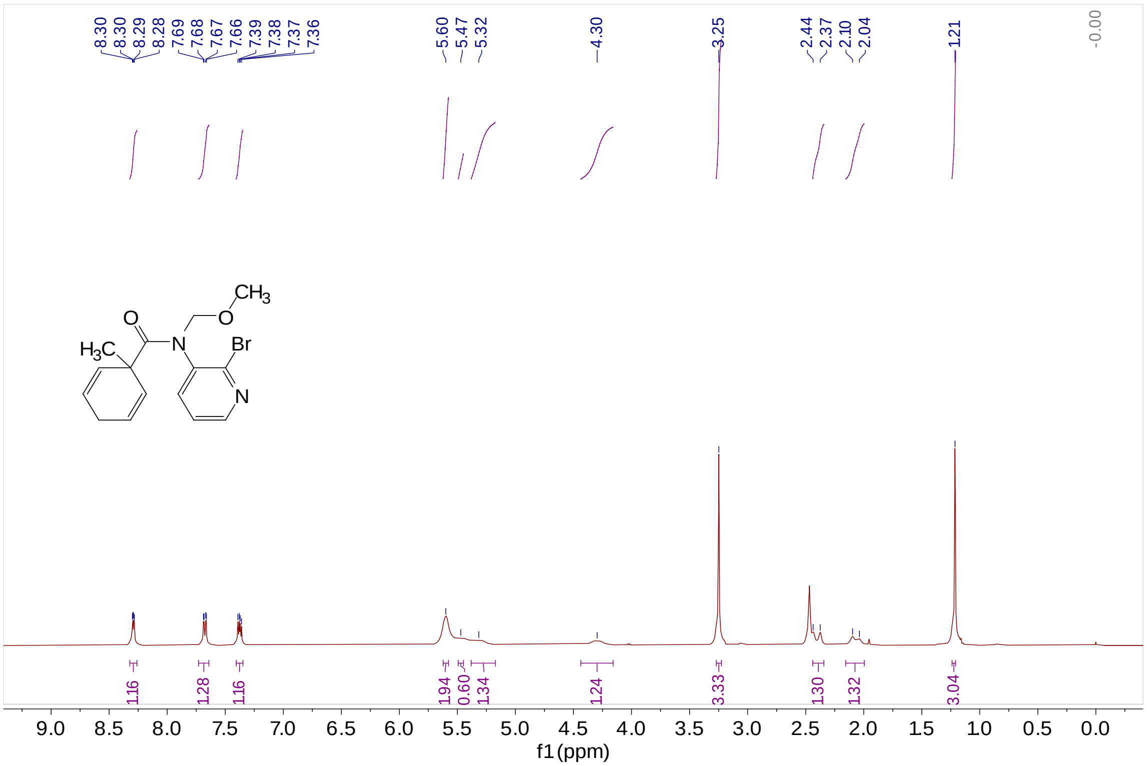

^{13}C{^{1}H} NMR (101 MHz, $CDCl_3$), **1m**

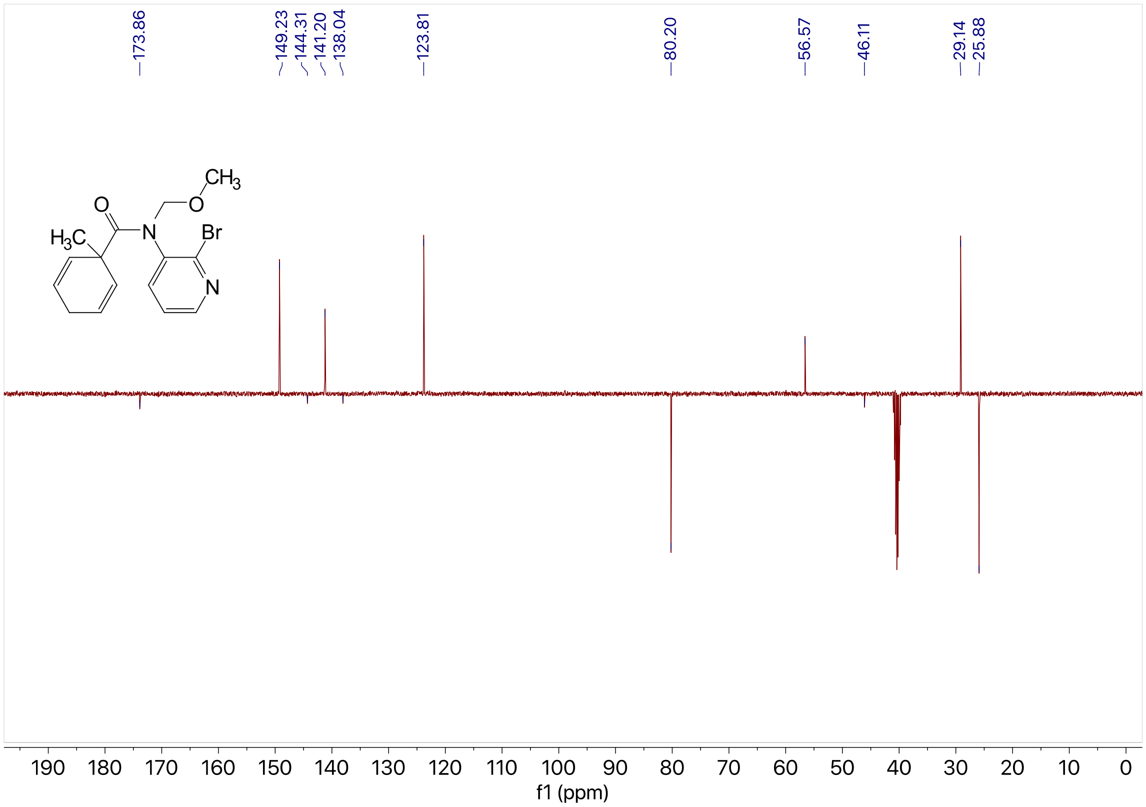

***N*-(3-Bromopyridin-2-yl)-*N*-(methoxymethyl)-1-methylcyclohexa-2,5-diene-1-carboxamide (1n).**

^{1}H NMR (400 MHz), **1n**

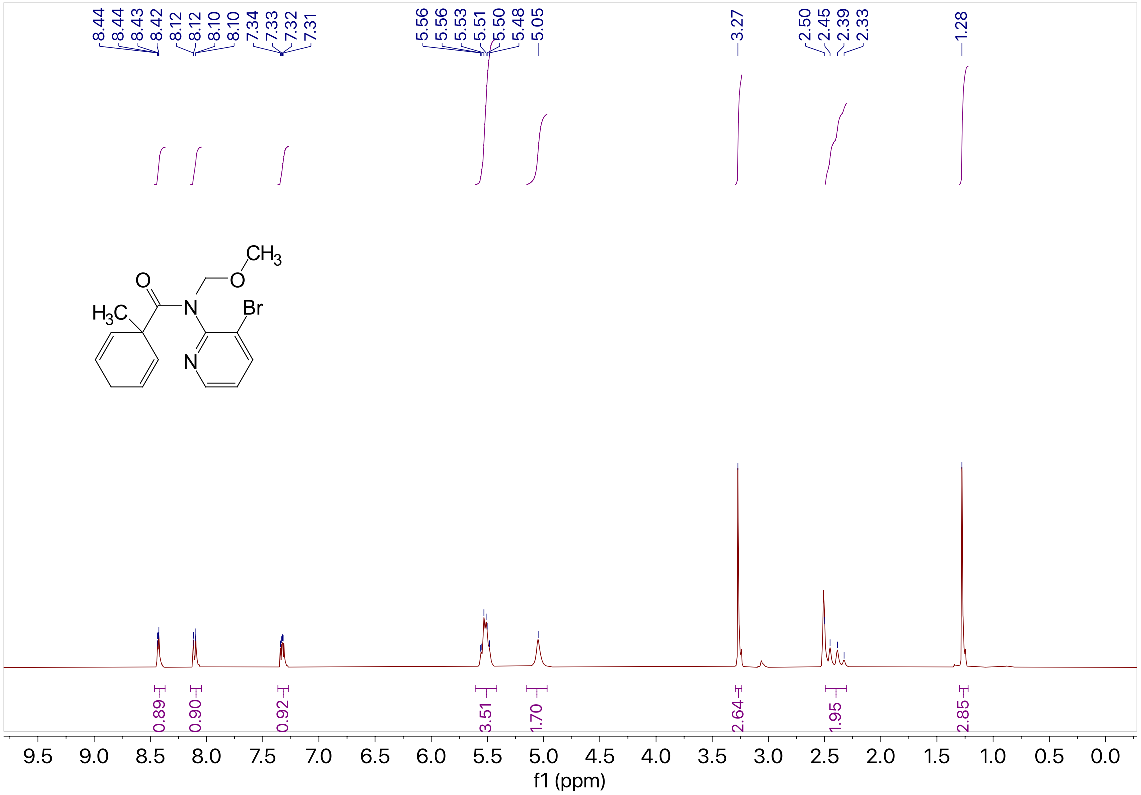

^{13}C{^{1}H} NMR (101 MHz, $CDCl_3$), **1n**

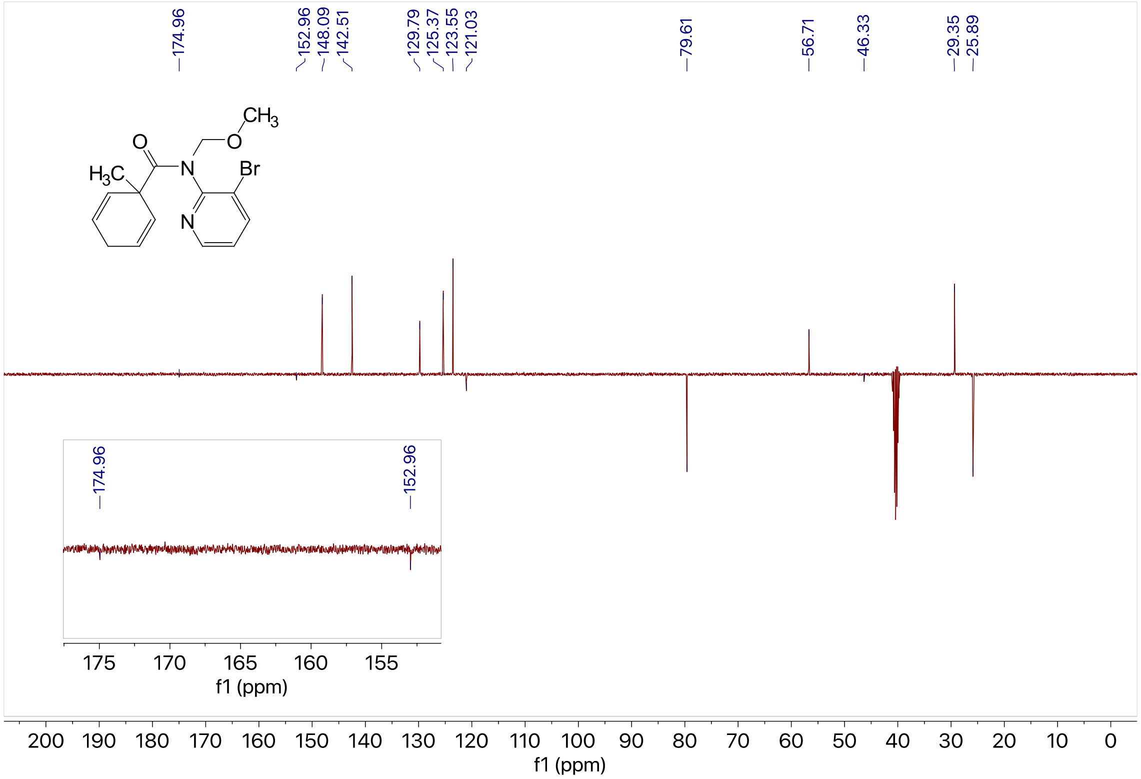

***N*-(3-Bromopyridin-2-yl)-1-ethyl-*N*-(methoxymethyl)cyclohexa-2,5-diene-1-carboxamide (1o).**

^{1}H NMR (400 MHz), **1o**

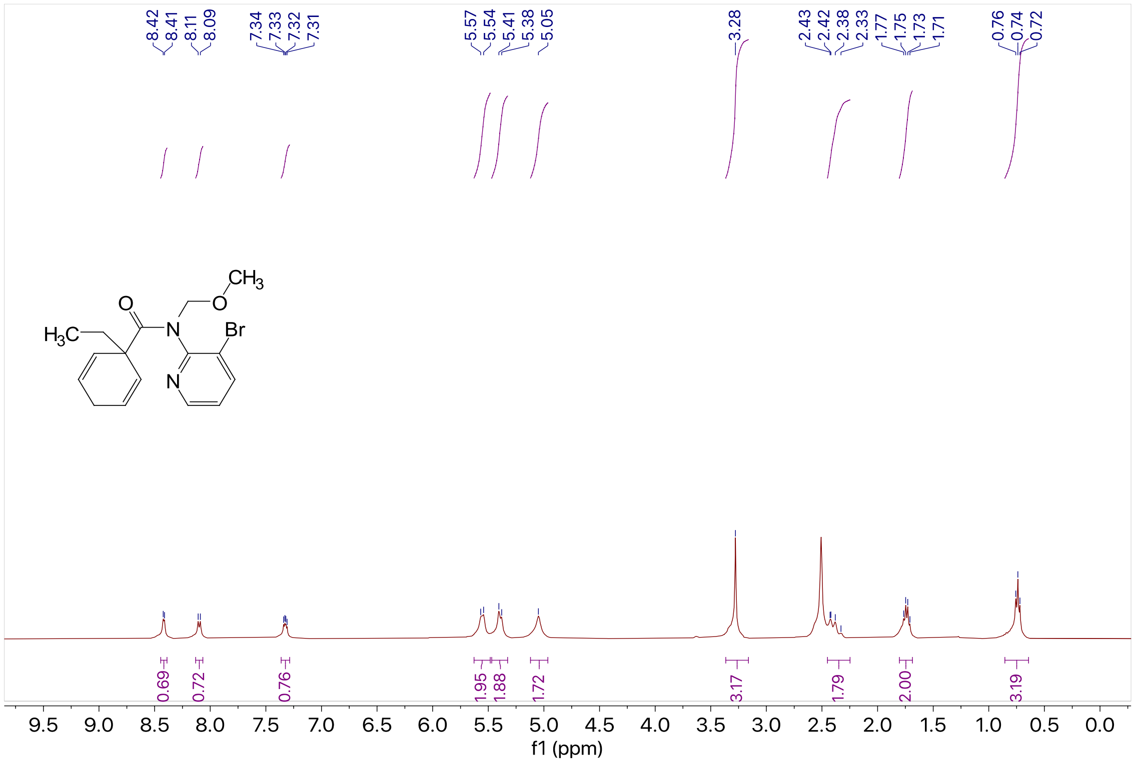

^{13}C{^{1}H} NMR (101 MHz, $CDCl_3$), **1o**

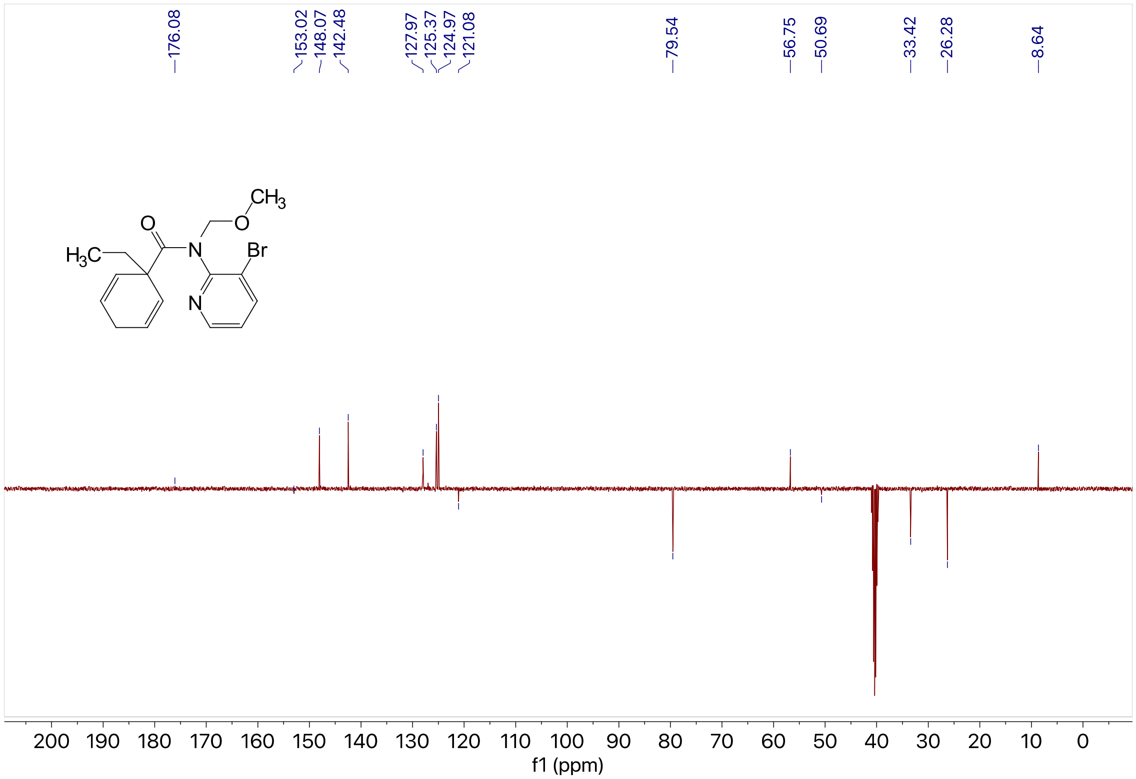

1-Ethyl-*N*-(2-iodopyridin-3-yl)cyclohexa-2,5-diene-1-carboxamide (1o-I).

^{1}H NMR (400 MHz), **1o-I**

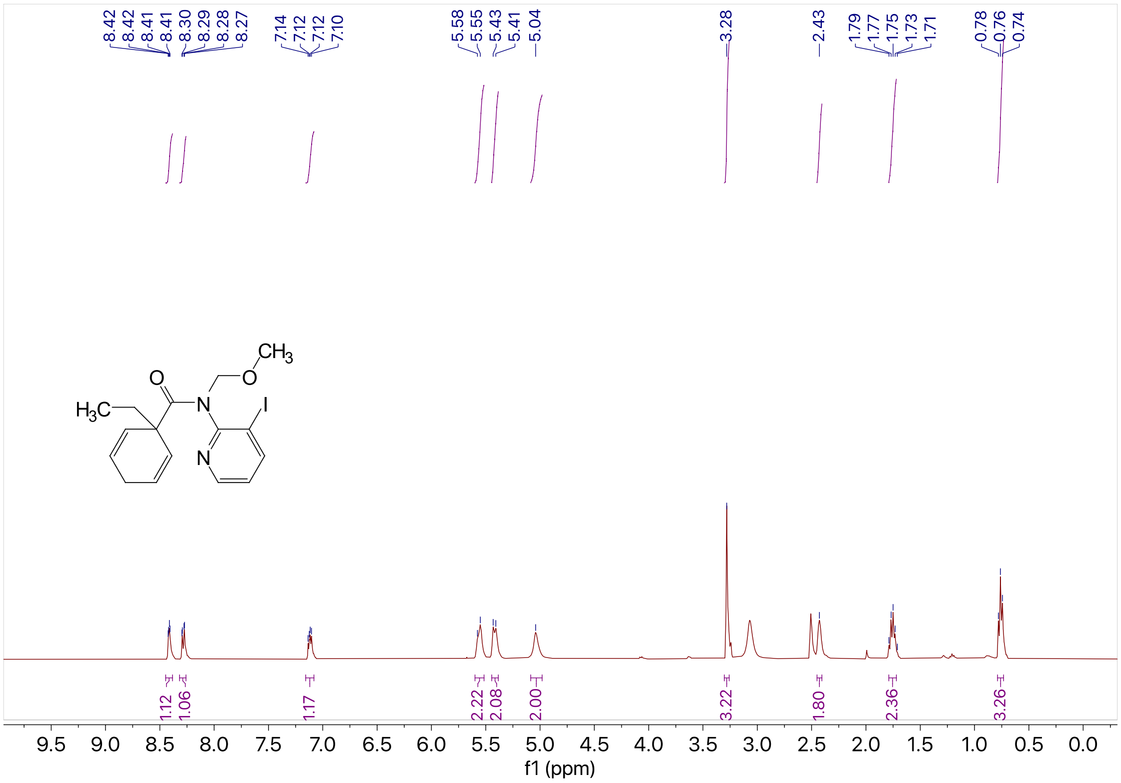

^{13}C{^{1}H} NMR (101 MHz, $CDCl_3$), **1o-I**

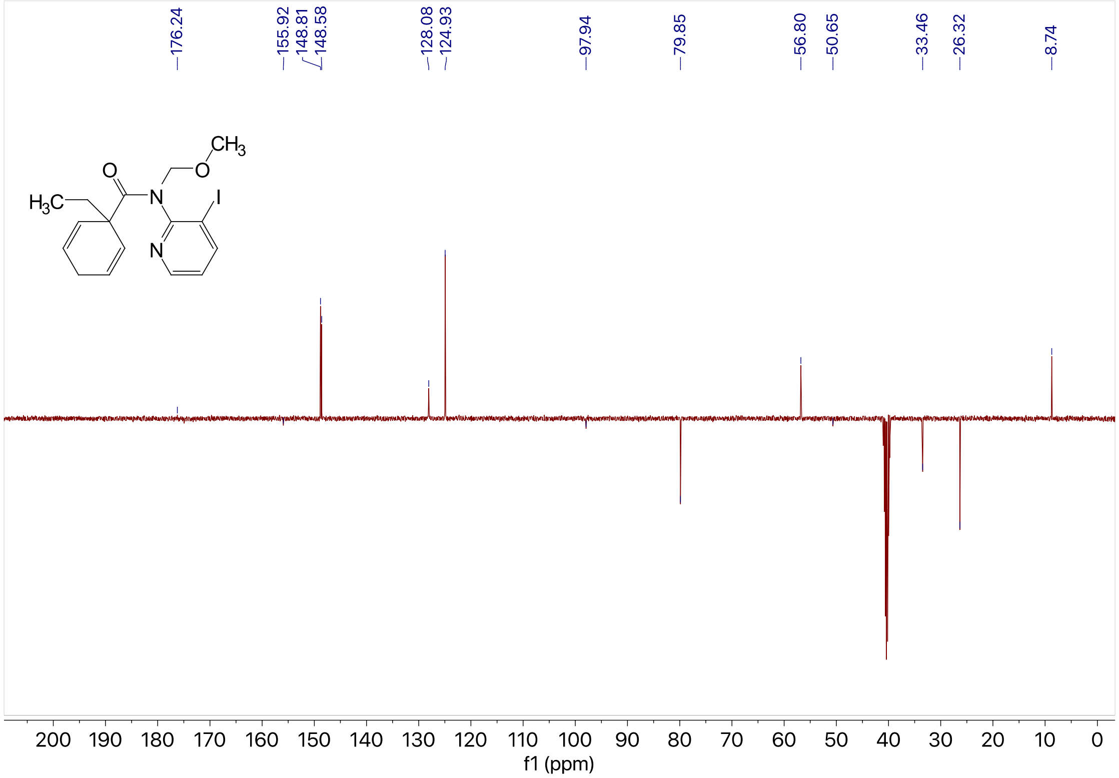

SI-Table 3: Chiral Ligand Screening.[a]

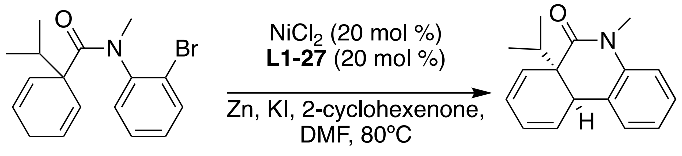

1d **2d**

Commercially available ligands (**L1-18**)

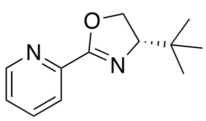

L1 *(2:1 e.r.)*

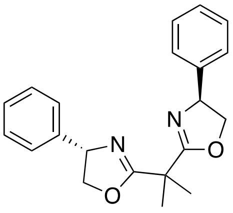

L2 *(1:1 e.r.)*

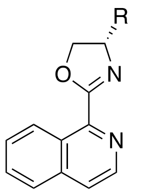

R = *t*Bu **L3** *(3:1 e.r.)*
R = *i*Pr **L4** *(1:1 e.r.)*

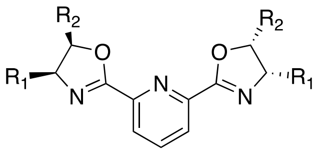

R_1 = *i*Pr, R_2 = H **L5** *(1:2 e.r.)*
R_1 = Ph, R_2 = H **L6** *(2:1 e.r.)*
R_1 = Bn, R_2 = H **L7** *(1:1 e.r.)*
R_1 = *t*Bu, R_2 = H **L8** *(1:1 e.r.)*
R_1 = Ph, R_2 = Ph **L9** *(1:1 e.r.)*

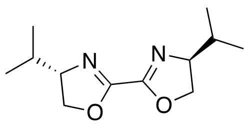

L10 *(1:1 e.r.)*

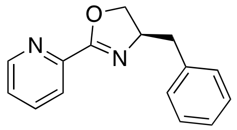

L11 *(1:1 e.r.)*

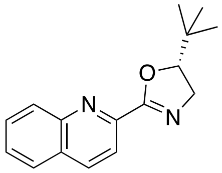

L12 *(1:1 e.r.)*

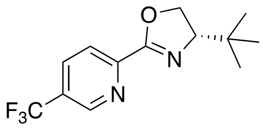

L13 *(1:3 e.r.)*

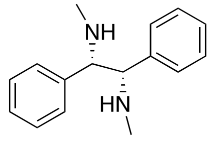

L14 *(n.d.)*

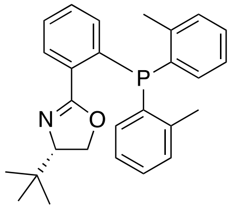

L15 *(n.d.)*

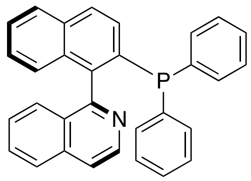

L16 *(1:1 e.r.)*

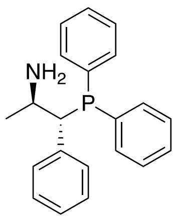

L17 *(n.d.)*

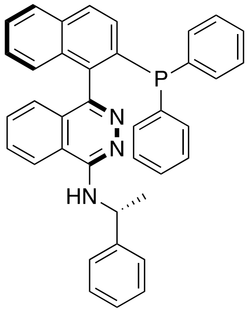

L18 *(n.d)*

Synthesized ligands (**L19-27**):

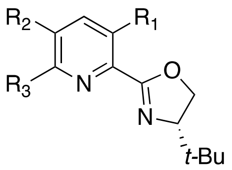

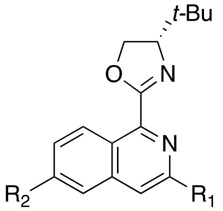

R_1 = CH_3, R_2, R_3 = H **L19** *(1:1 e.r.)*
R_1, R_2 = H, R_3 = OMe **L20** *(1:1 e.r.)*
R_1, R_2 = OMe, R_3 = H **L21** *(1:1 e.r.)*
R_1, R_2 = H, R_3 = F **L22** *(1:1 e.r.)*
R_1, R_2 = F, R_3 = H **L23** *(2:1 e.r.)*
R_1, R_2 = H, R_3 = CH_3 **L24** *(1:1 e.r.)*

R_1 = CH_3, R_2 = H **L25** *(1:1 e.r.)*
R_1 = H, R_2 = F **L26** *(7:1 e.r.)*
R_1 = H, R_2 = CH_3 **L27** *(9:1 e.r.)*[b]

---

[a]Unless otherwise noted, the reaction of aryl bromide **1d** (1 equiv.) was carried out with $NiCl_2$ (20 mol %), **L1-26** (20 mol %), Zn (3 equiv.), KI (1 equiv.), 2-cyclohexenone (2 equiv.), and DMF (12 mL/mmol) mmol) at 80 ˚C. [b]With NiI_2 (10 mol %), **L27** (15 mol %), Mn (3 equiv.), and 2-cyclohexenone (3 equiv.).

Chiral LC's, GCMS data, and NMR spectra for Mizoroki-Heck reaction products

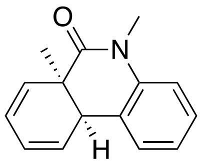

(6aR,10aR)-5,6a-Dimethyl-6a,10a-dihydrophenanthridin-6(5H)-one (2a).

Chiral LC for racemic **2a** sample

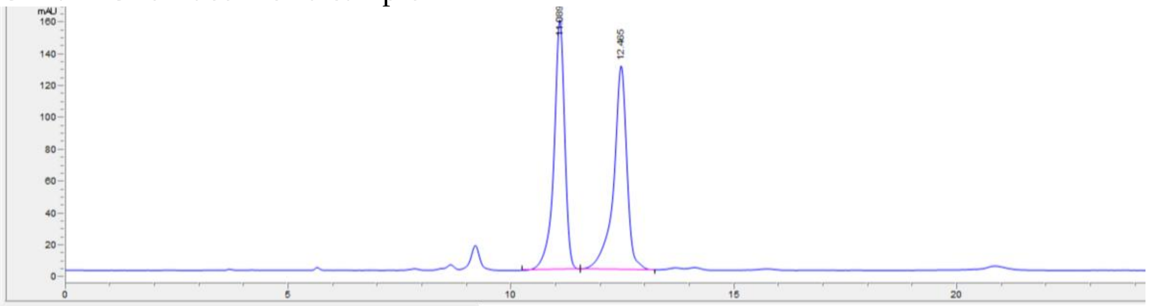

#	Time	Area	Height	Width	Area%	Symmetry
1	11.089	2641.9	157.2	0.252	49.978	1.228
2	12.465	2644.2	128.1	0.3029	50.022	1.31

Chiral LC for enantioselective **2a** reaction

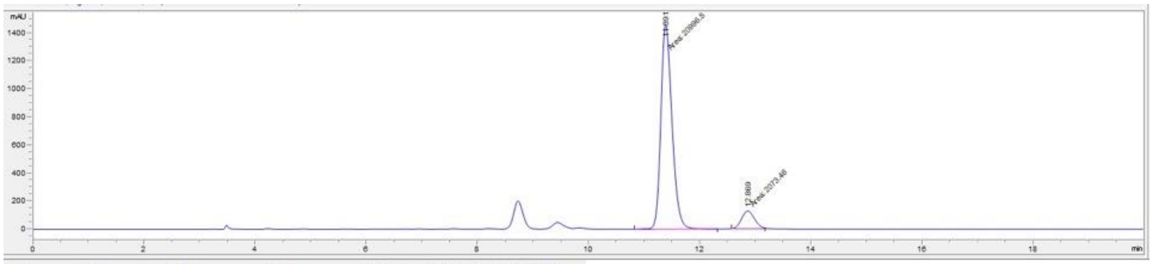

#	Time	Area	Height	Width	Area%	Symmetry
1	11.391	20996.5	1477.7	0.2368	91.012	0.733
2	12.869	2073.5	127.8	0.2704	8.988	0.872

GCMS data, **2a**

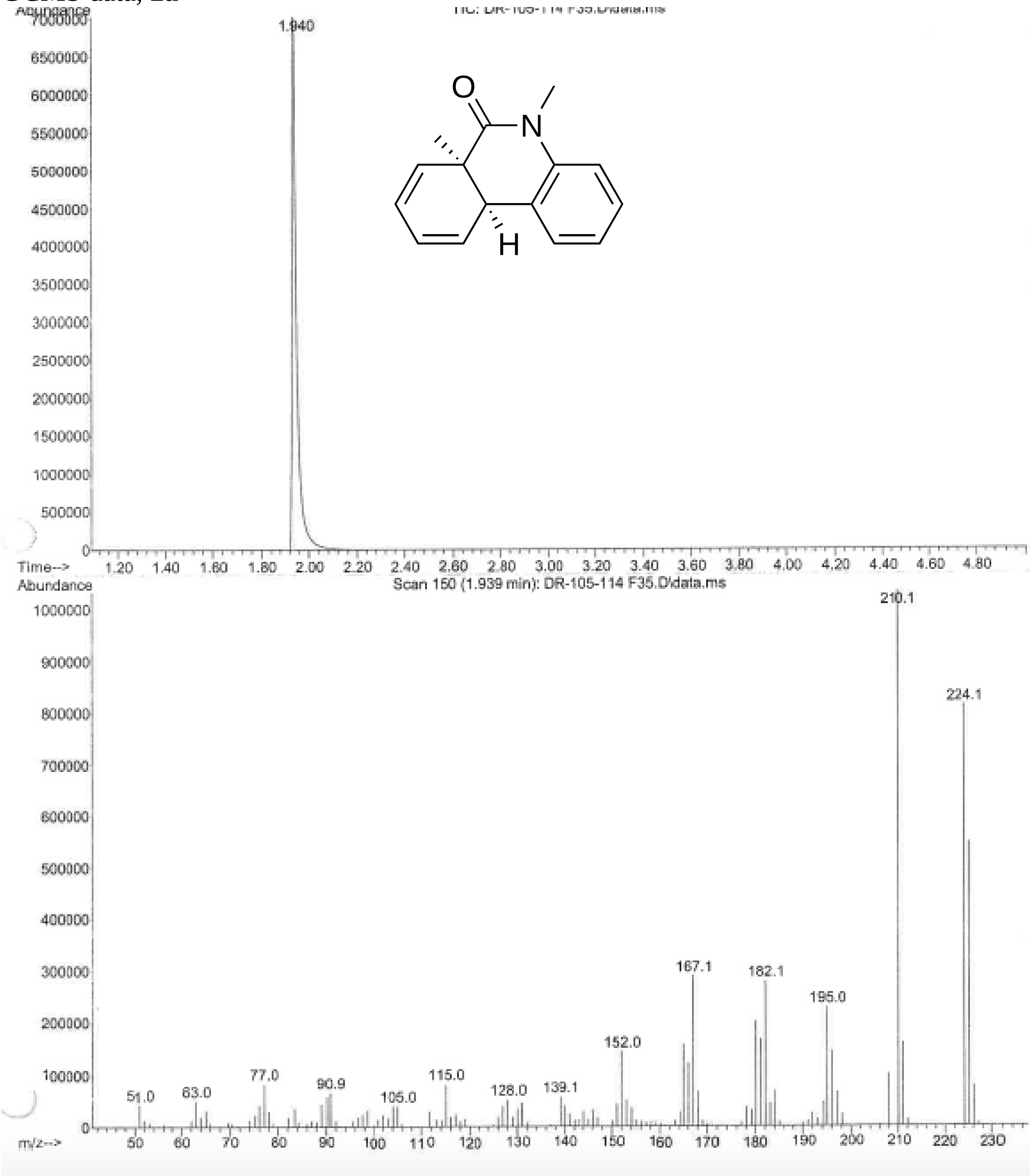

Abundance
TIC: DR-105-114 F35.D\data.ms
1.940
O
N
H
Time-->
Abundance
Scan 150 (1.939 min): DR-105-114 F35.D\data.ms
210.1
224.1
167.1
182.1
195.0
152.0
51.0
63.0
77.0
90.9
105.0
115.0
128.0
139.1
m/z-->

^{1}H NMR (400 MHz), **2a**

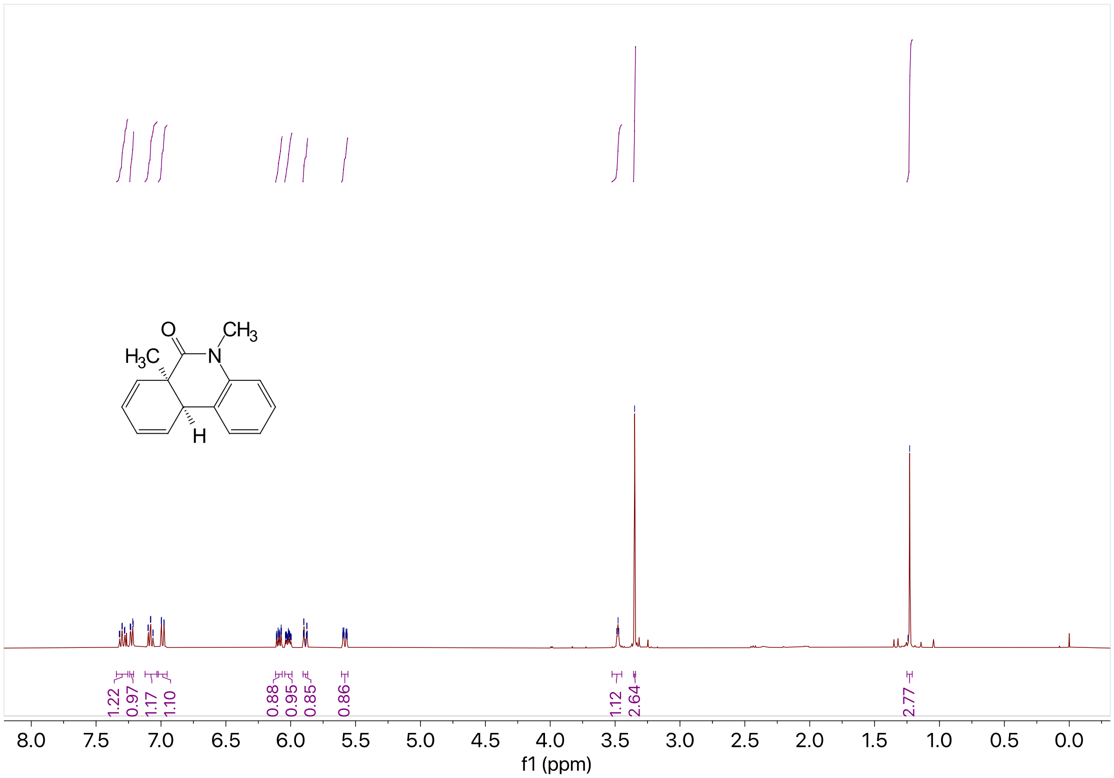

$^{13}C\{^{1}H\}$ NMR (101 MHz, $CDCl_3$), **2a**

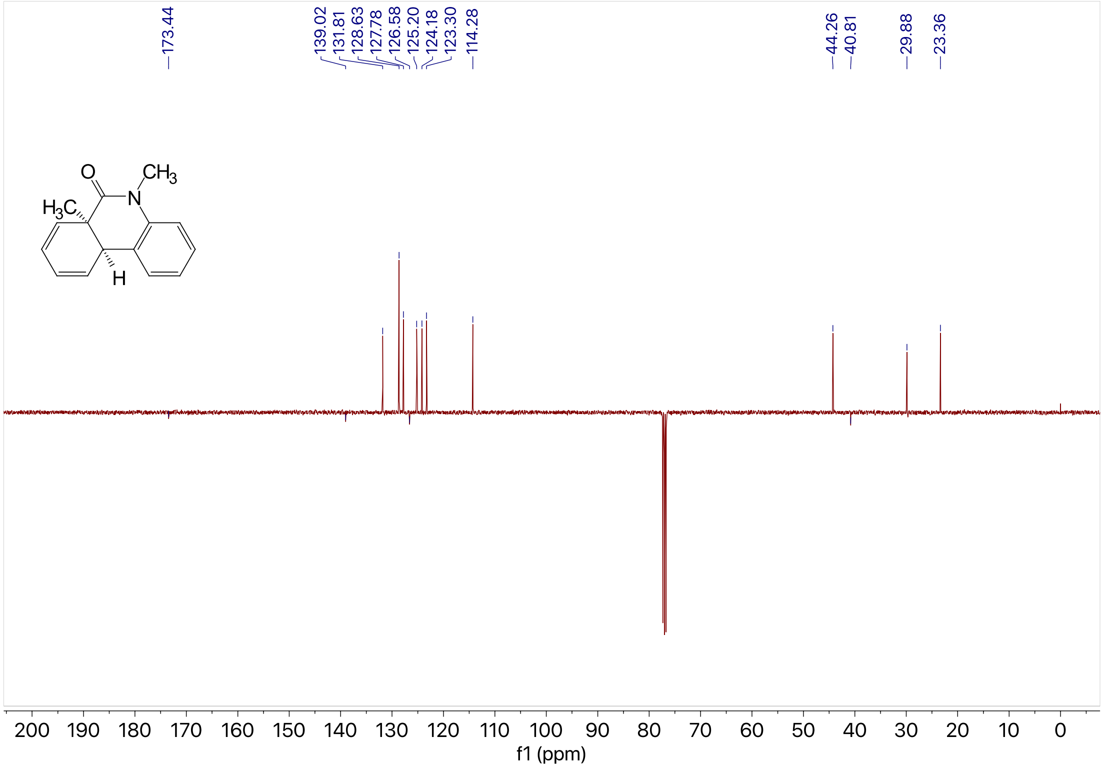

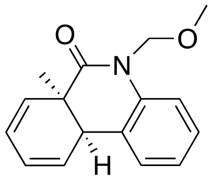

(6aR,10aR)-5-(Methoxymethyl)-6a-methyl-6a,10a-dihydrophenanthridin-6(5H)-one (2b).

Chiral LC for racemic **2b** sample

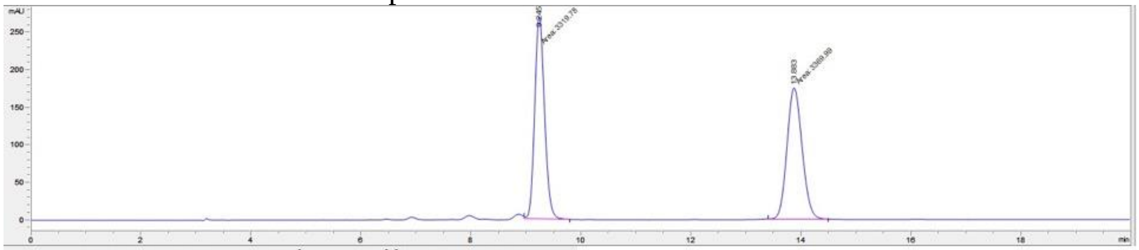

#	Time	Area	Height	Width	Area%	Symmetry
1	9.245	3319.8	268.6	0.206	49.625	0.843
2	13.883	3370	175.1	0.3209	50.375	0.874

Chiral LC for enantioselective **2b** reaction

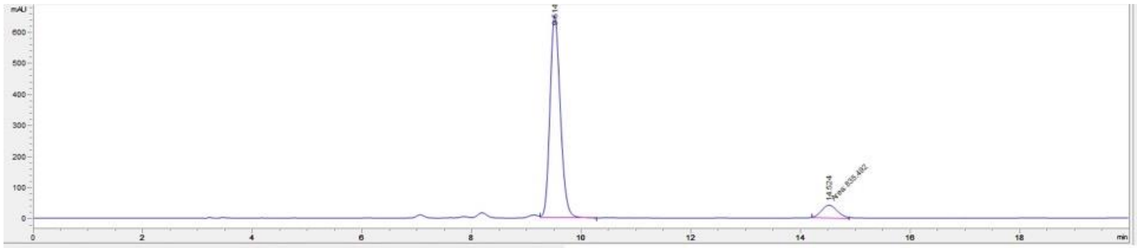

#	Time	Area	Height	Width	Area%	Symmetry
1	9.514	8599.8	651.5	0.204	91.145	0.78
2	14.524	835.5	41.3	0.337	8.855	0.857

GCMS data, **2b**

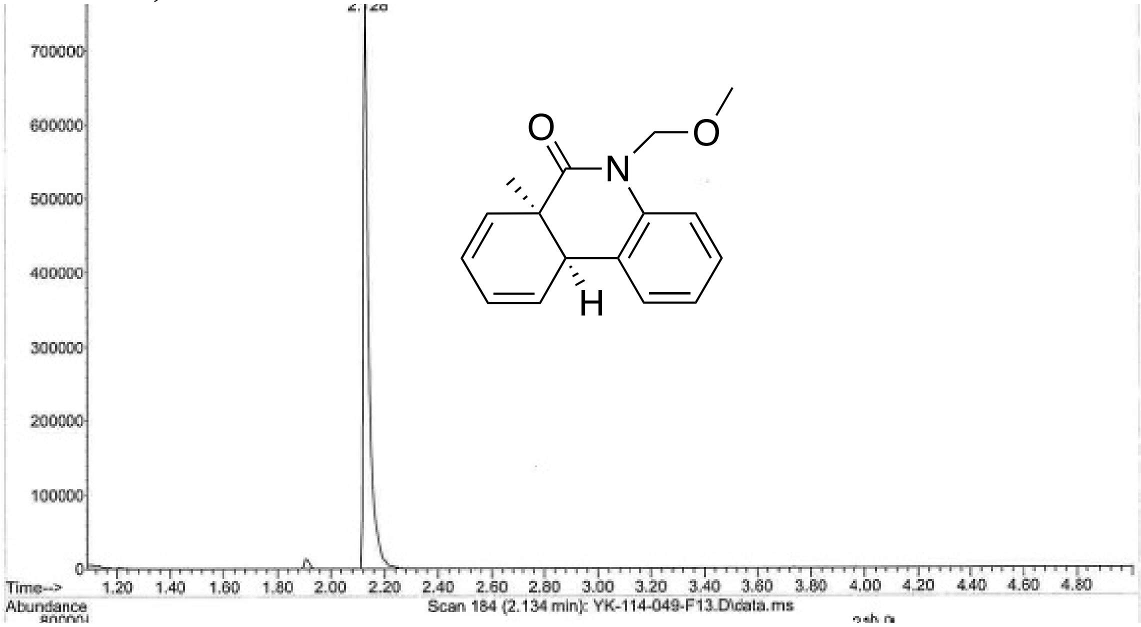

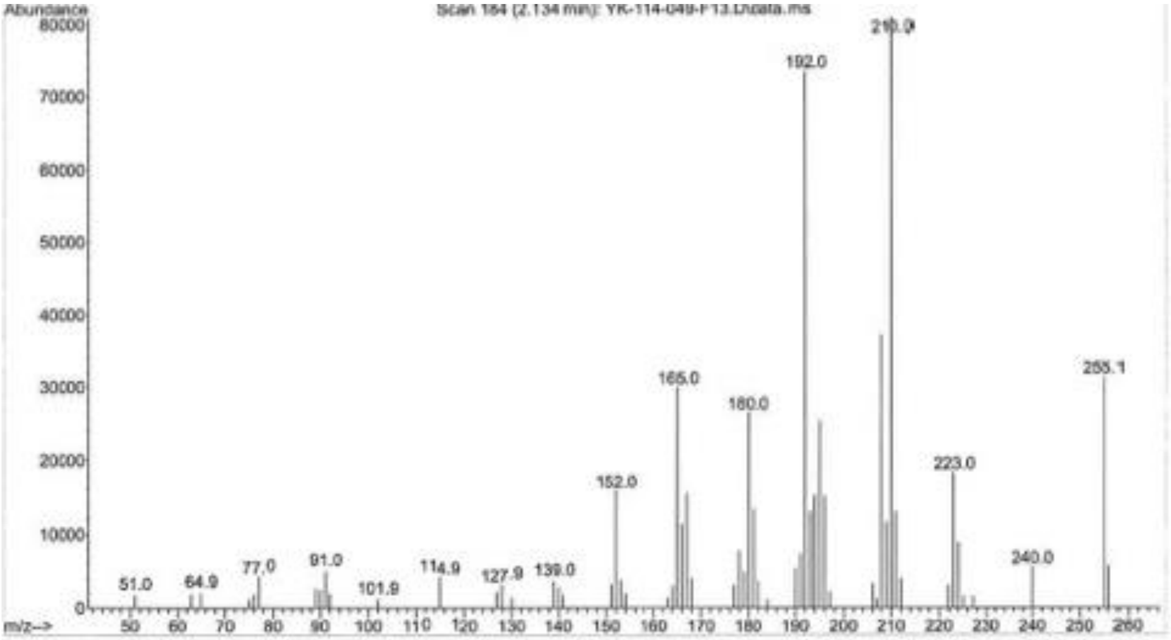

^{1}*H NMR (400 MHz)*, **2b**

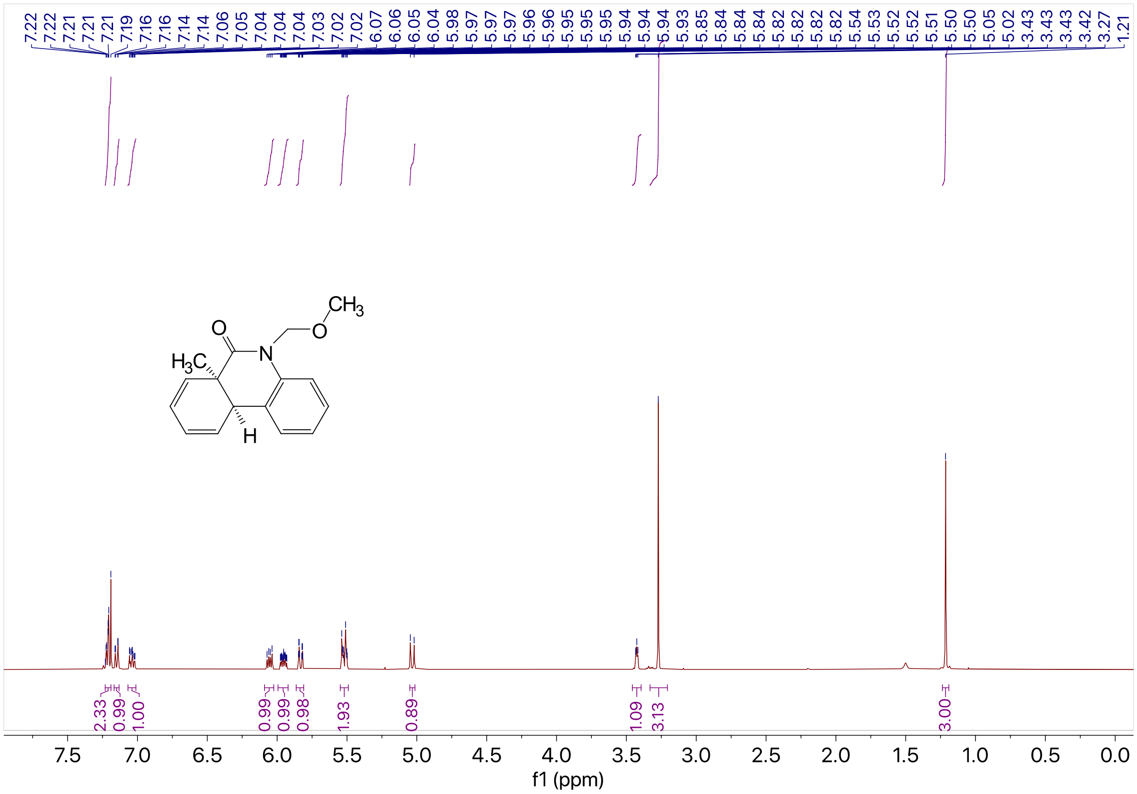

$^{13}C\{^{1}H\}$ *NMR (101 MHz, $CDCl_3$)*, **2b**

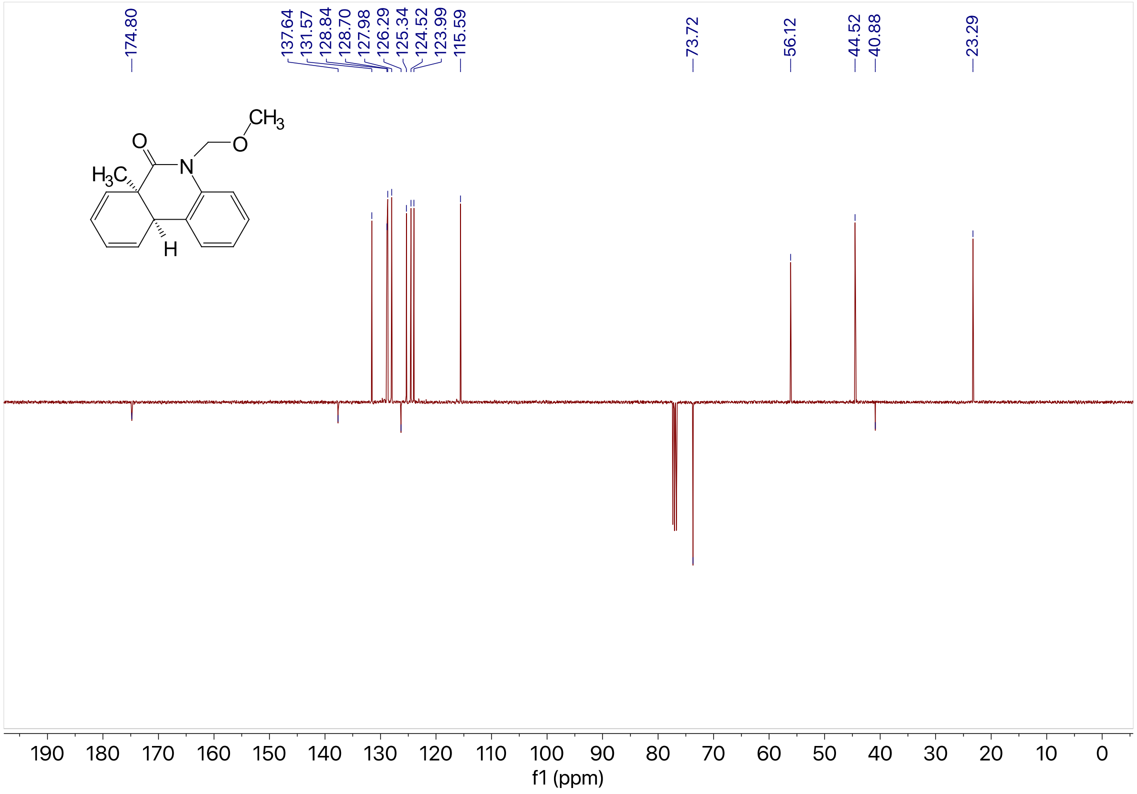

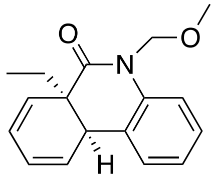

(6aR,10aR)-6a-Ethyl-5-(methoxymethyl)-6a,10a-dihydrophenanthridin-6(5H)-one (2c).

Chiral LC for racemic **2c** sample

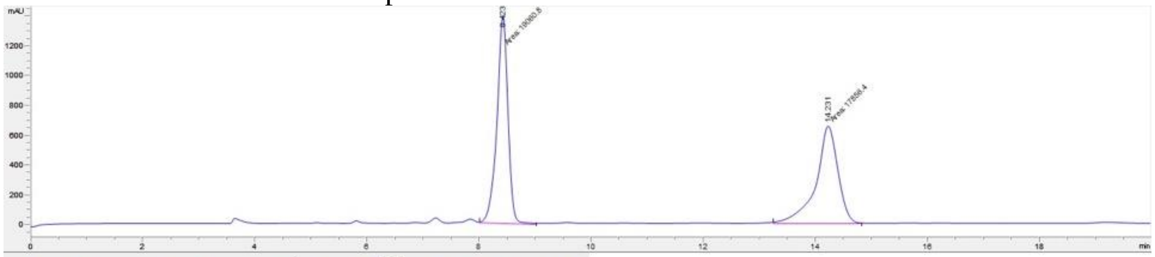

#	Time	Area	Height	Width	Area%	Symmetry
1	8.423	19060.8	1384	0.2295	51.631	1.13
2	14.231	17856.4	653.2	0.4556	48.369	1.25

Chiral LC for enantioselective **2c** reaction

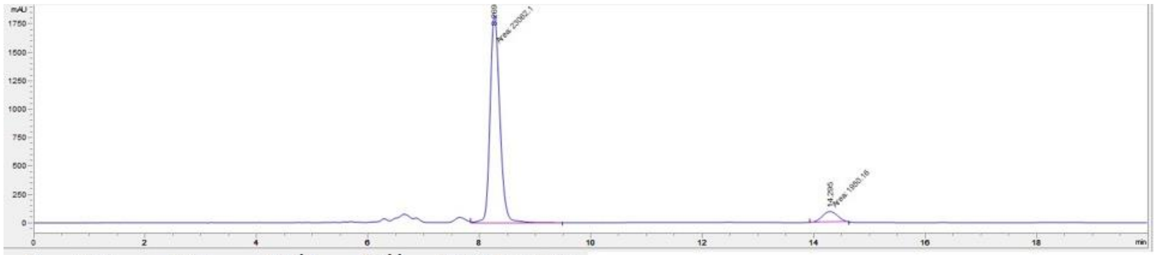

#	Time	Area	Height	Width	Area%	Symmetry
1	8.269	23062.1	1832.2	0.2098	92.203	0.747
2	14.295	1950.2	97.5	0.3335	7.797	0.992

Chiral LC for 1.0 mmol scale enantioselective **2c** reaction

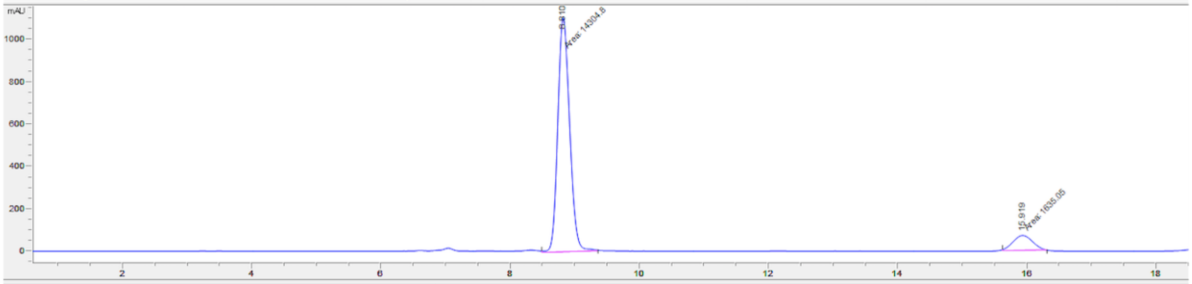

#	Time	Area	Height	Width	Area%	Symmetry
1	8.81	14304.8	1108.1	0.2152	89.742	0.801
2	15.919	1635.1	74.3	0.3665	10.258	0.951

GCMS data, **2c**

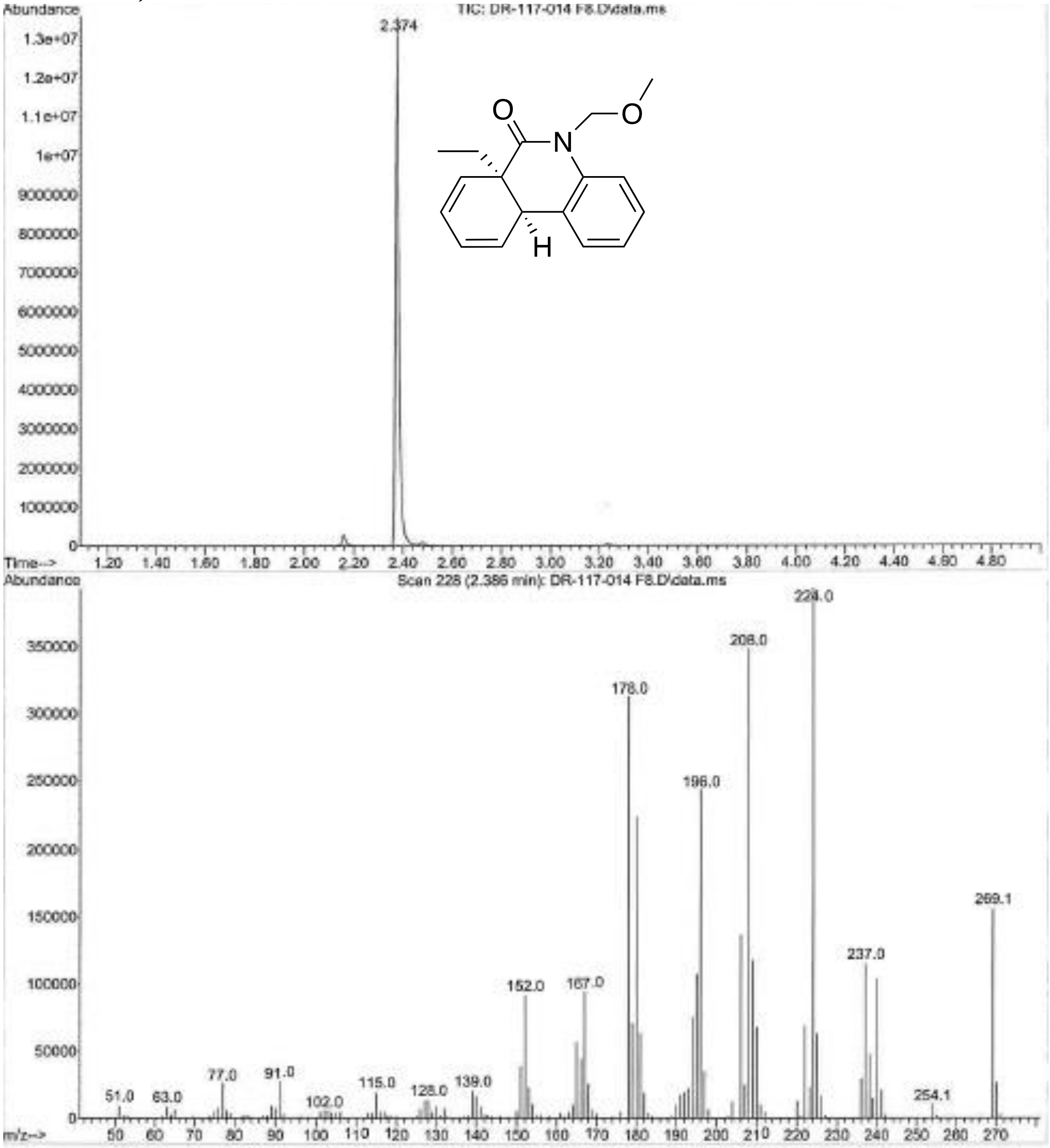

^{1}H *NMR (400 MHz),* **2c**

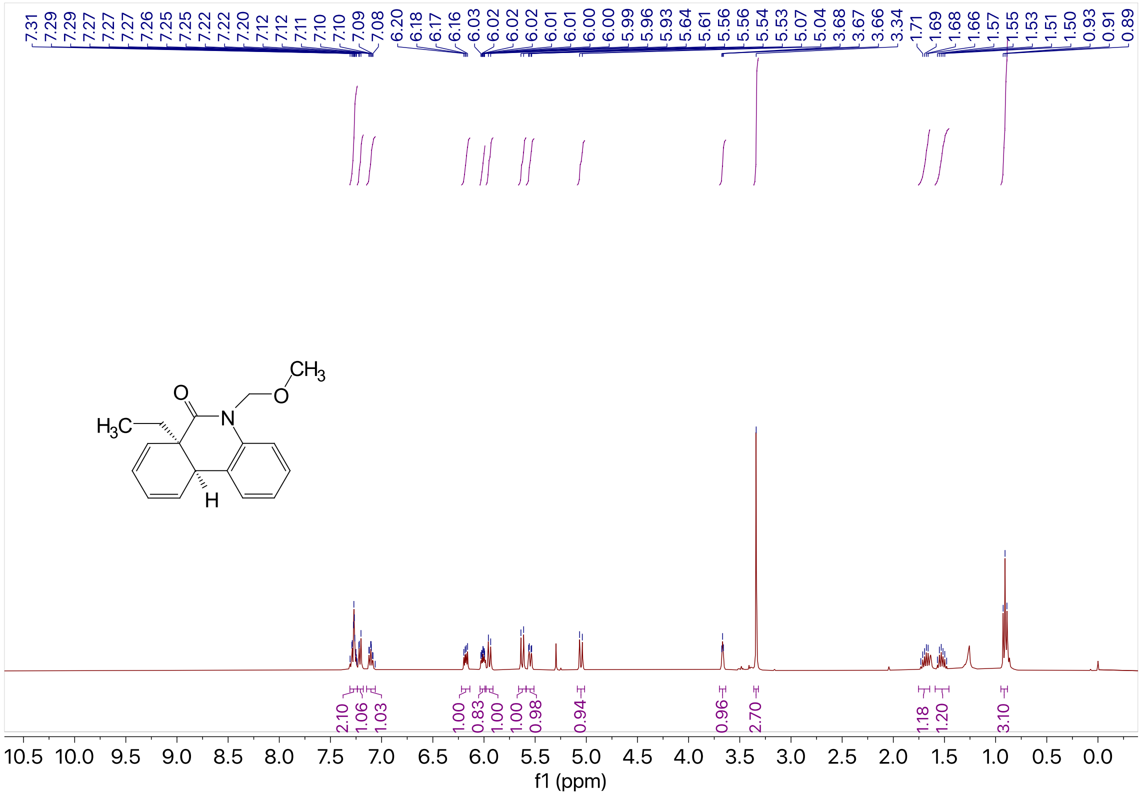

$^{13}C\{^{1}H\}$ *NMR (101 MHz, $CDCl_3$),* **2c**

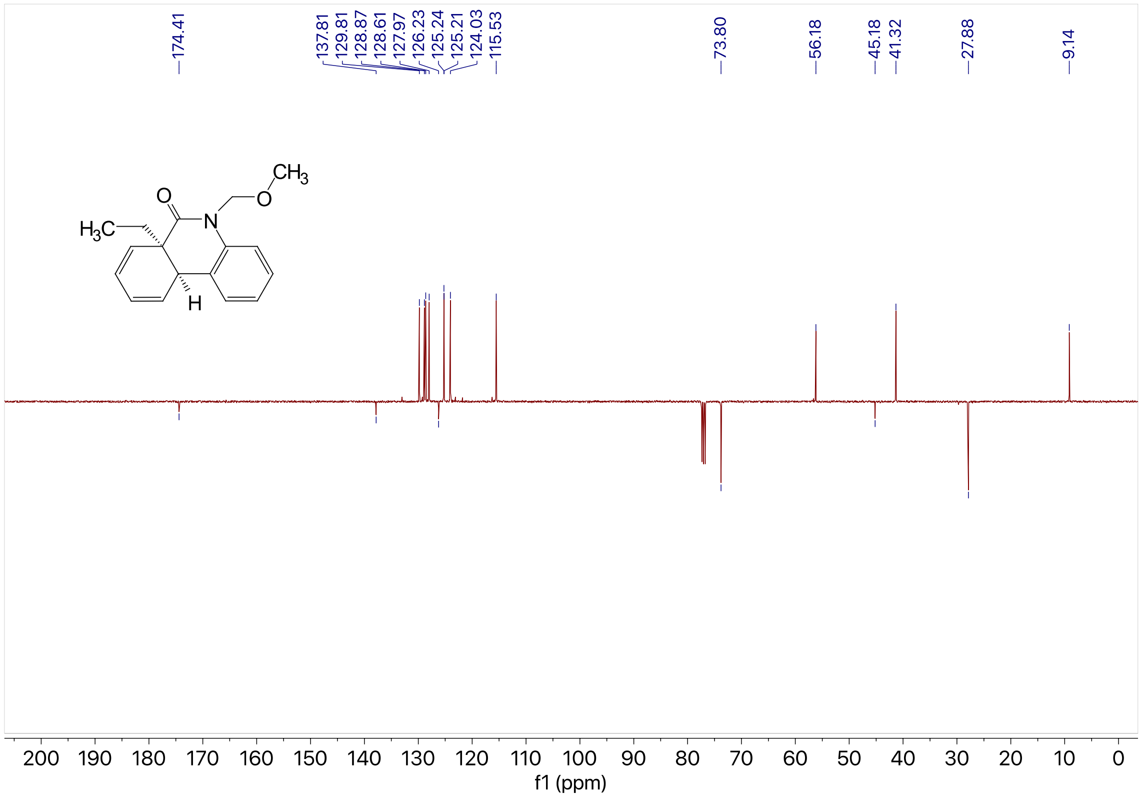

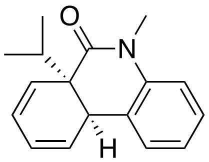

(6aR,10aR)-6a-Isopropyl-5-methyl-6a,10a-dihydrophenanthridin-6(5H)-one (2d).

Chiral LC for racemic **2d** sample

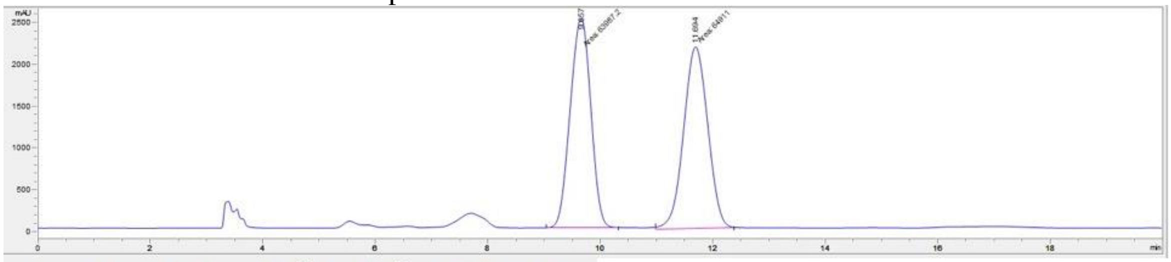

#	Time	Area	Height	Width	Area%	Symmetry
1	9.657	63987.2	2496.4	0.4272	49.642	1.083
2	11.694	64911	2165.8	0.4995	50.358	0.965

Chiral LC for enantioselective **2d** reaction

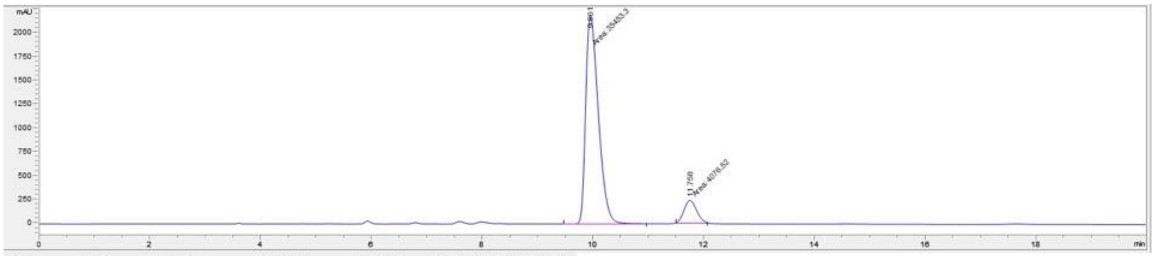

#	Time	Area	Height	Width	Area%	Symmetry
1	9.961	35453.3	2170	0.2723	89.687	0.572
2	11.758	4076.5	246.4	0.2757	10.313	0.845

GCMS data, **2d**

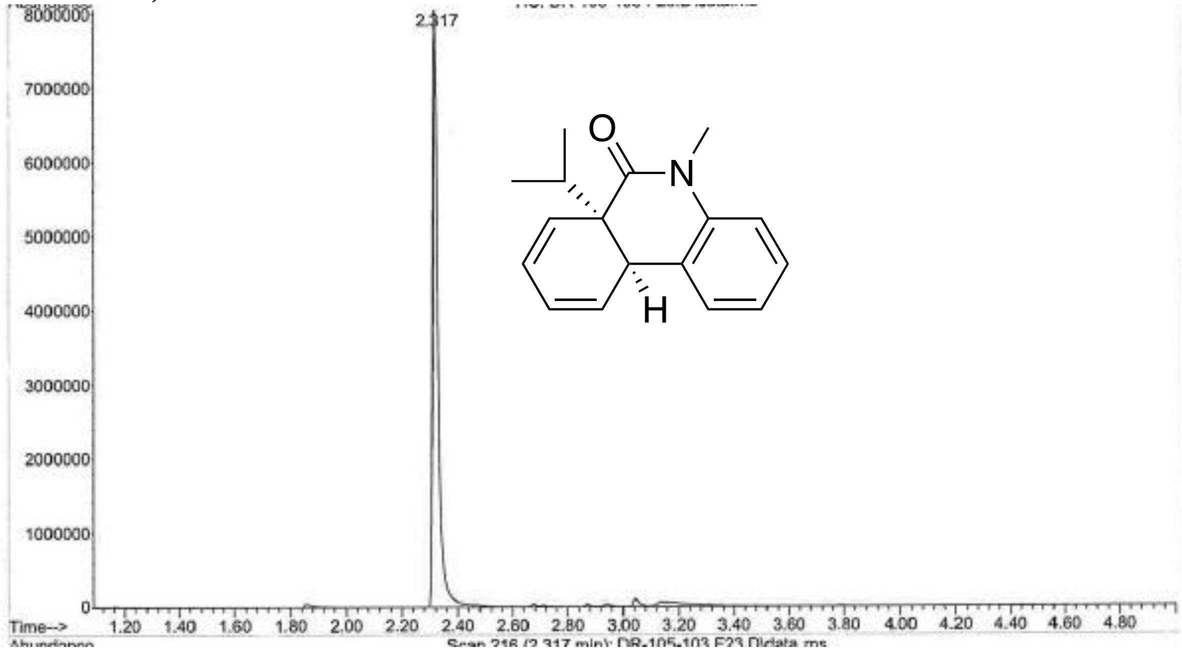

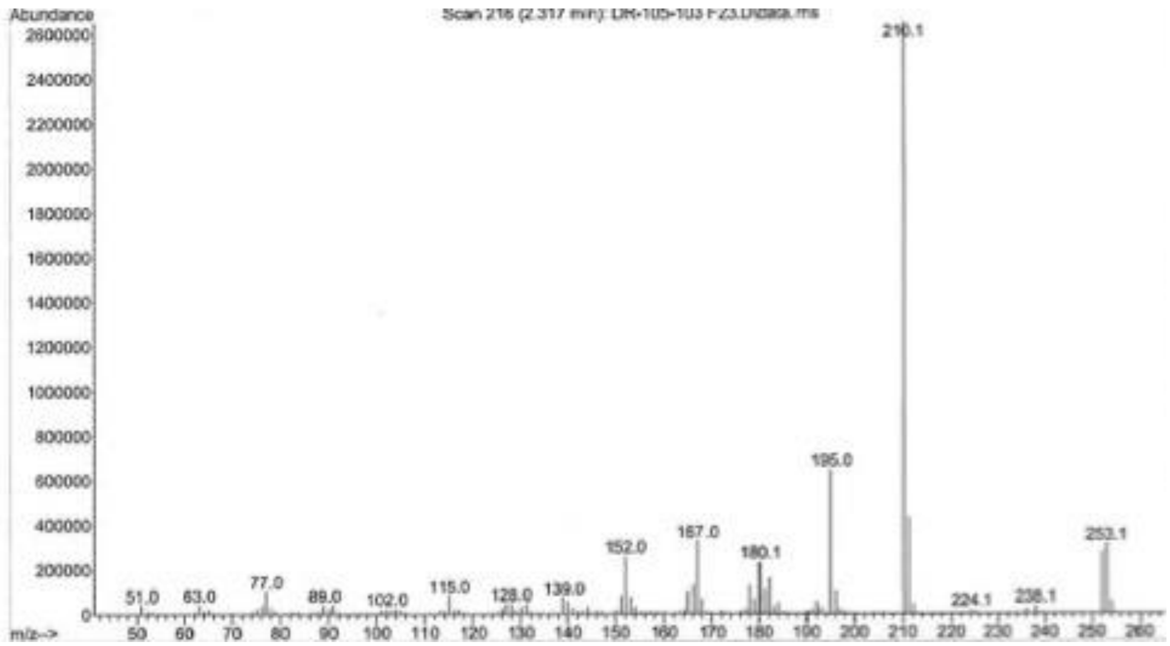

^1H *NMR (400 MHz)*, **2d**

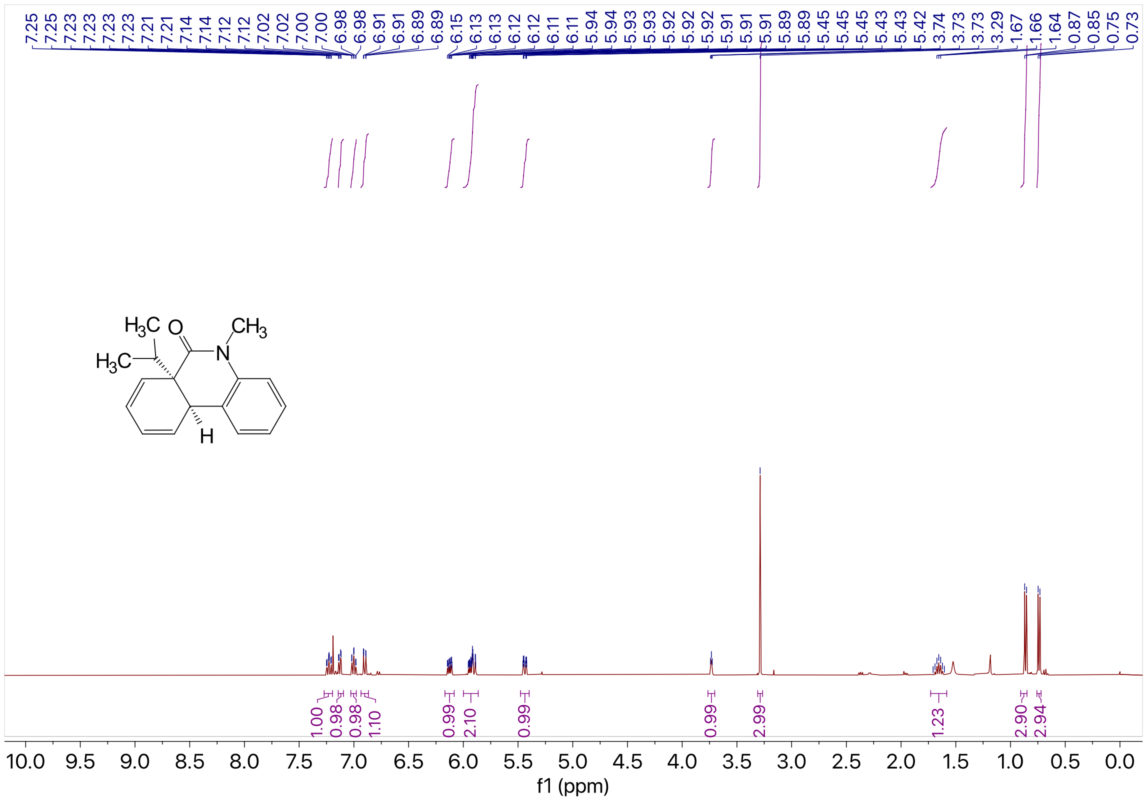

$^{13}C\{^{1}H\}$ NMR (101 MHz, $CDCl_3$), **2d**

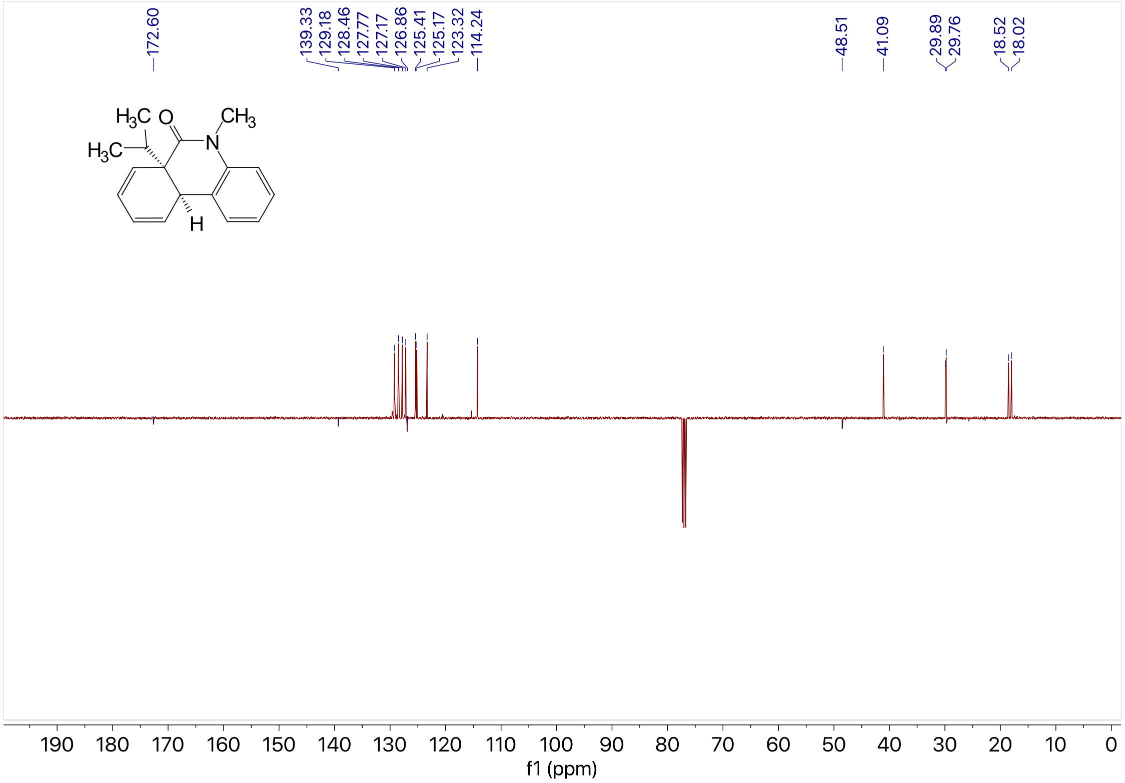

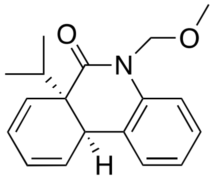

(6aR,10aR)-6a-Isopropyl-5-(methoxymethyl)-6a,10a-dihydrophenanthridin-6(5H)-one (2e).

Chiral LC for racemic **2e** sample

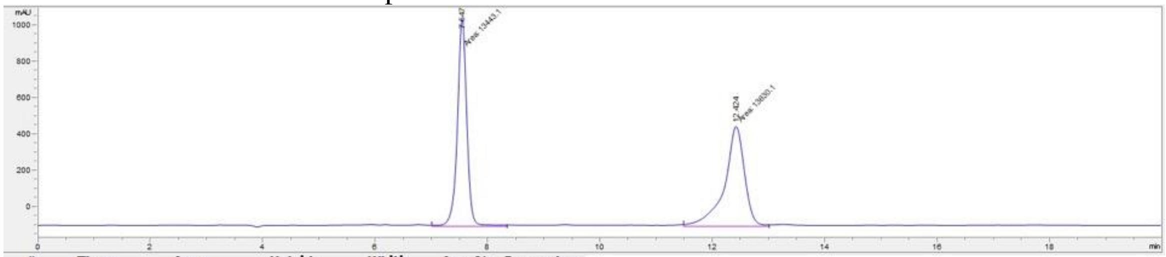

#	Time	Area	Height	Width	Area%	Symmetry
1	7.547	13443.1	1141.5	0.1963	49.655	0.994
2	12.424	13630.1	549.3	0.4136	50.345	1.357

Chiral LC for enantioselective **2e** reaction (at 80 ˚C)

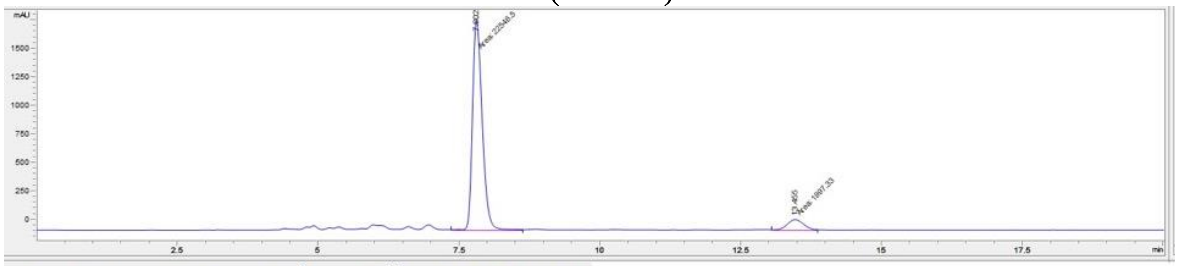

#	Time	Area	Height	Width	Area%	Symmetry
1	7.802	22546.5	1837.9	0.2045	91.862	0.671
2	13.455	1997.3	94.7	0.3517	8.138	0.913

Chiral LC for enantioselective **2e** reaction (at 60 ˚C)

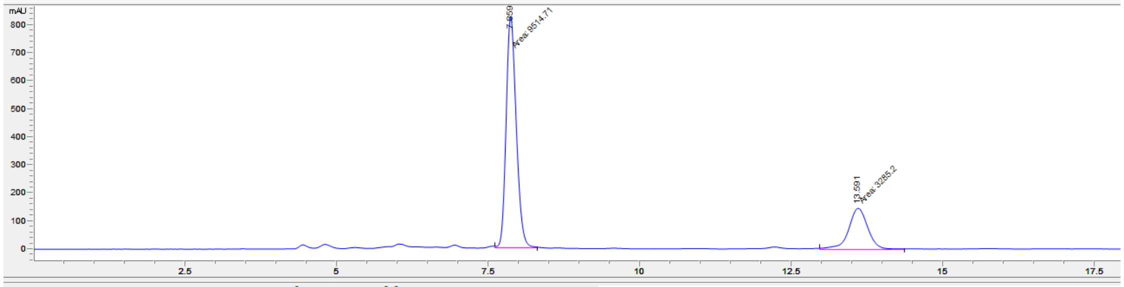

#	Time	Area	Height	Width	Area%	Symmetry
1	7.859	9514.7	821.5	0.193	74.334	0.776
2	13.591	3285.2	146.5	0.3737	25.666	0.969

GCMS data, **2e**

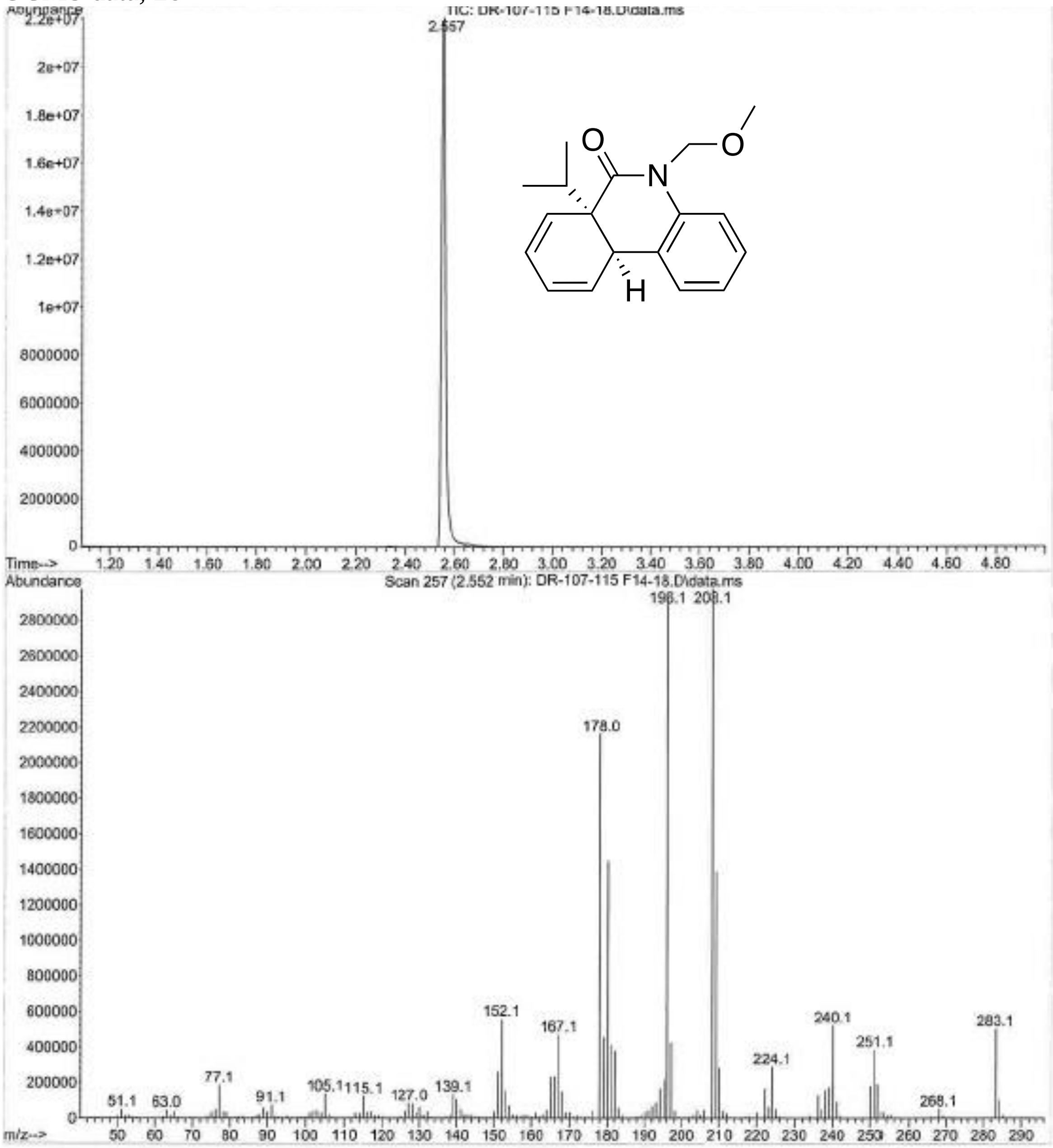

^{1}H NMR (400 MHz), **2e**

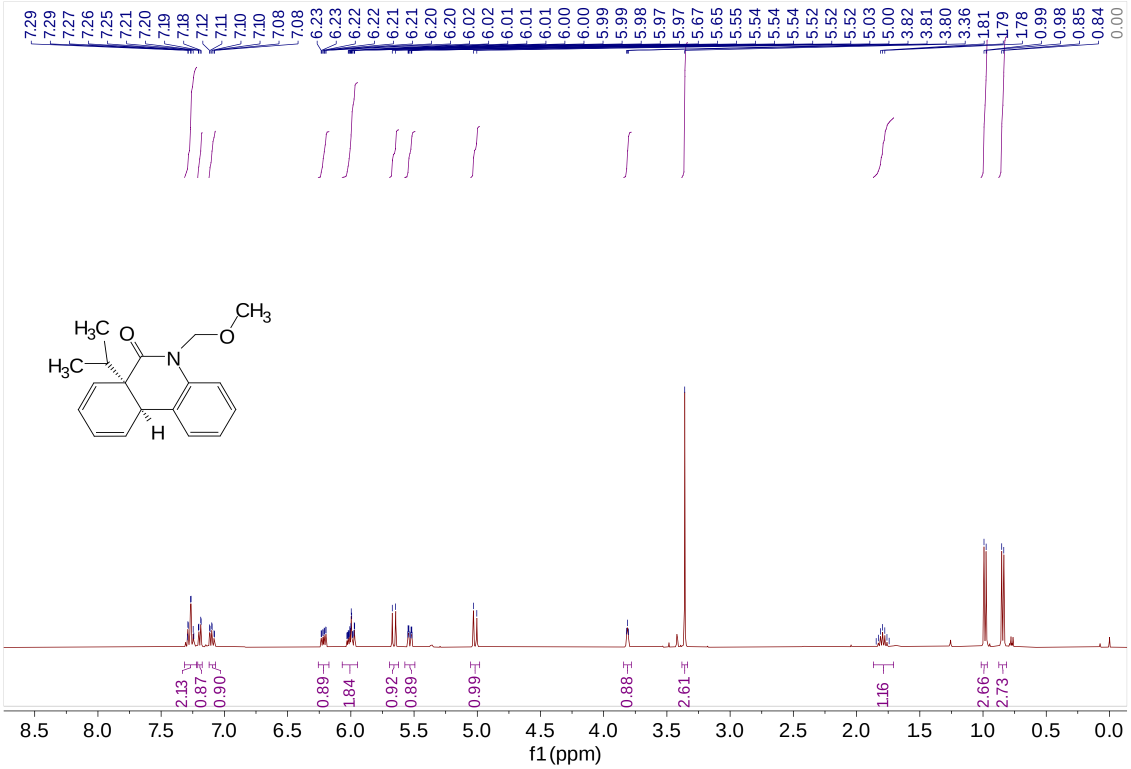

$^{13}C\{^{1}H\}$ NMR (101 MHz, $CDCl_3$), **2e**

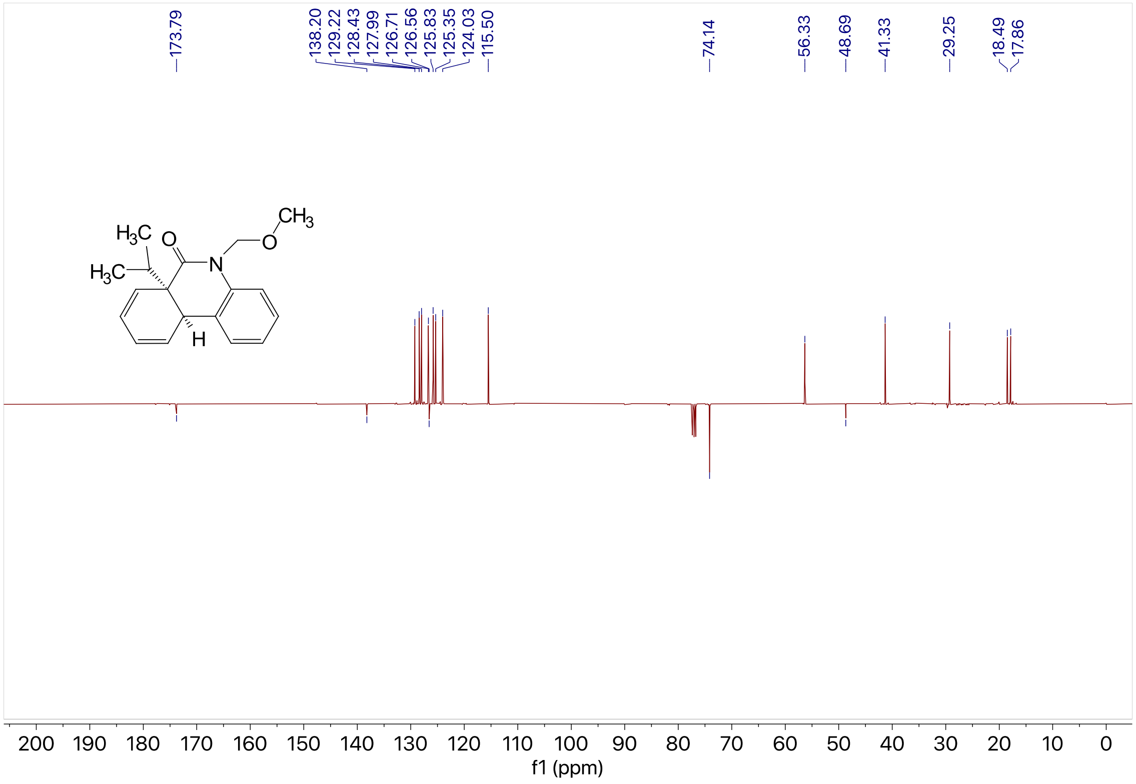

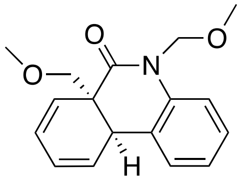

(6aR,10aR)-5,6a-Bis(methoxymethyl)-6a,10a-dihydrophenanthridin-6(5H)-one (2f).

Chiral LC for racemic **2f** sample

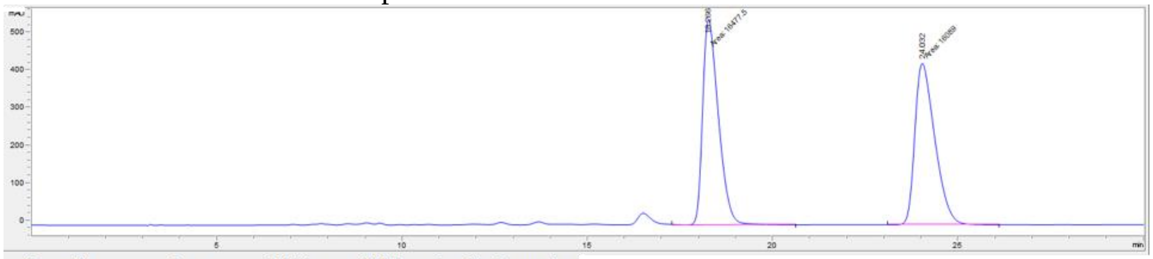

#	Time	Area	Height	Width	Area%	Symmetry
1	18.266	16477.5	547.6	0.5015	50.596	0.576
2	24.032	16089	429.5	0.6244	49.404	0.579

Chiral LC for enantioselective **2f** reaction

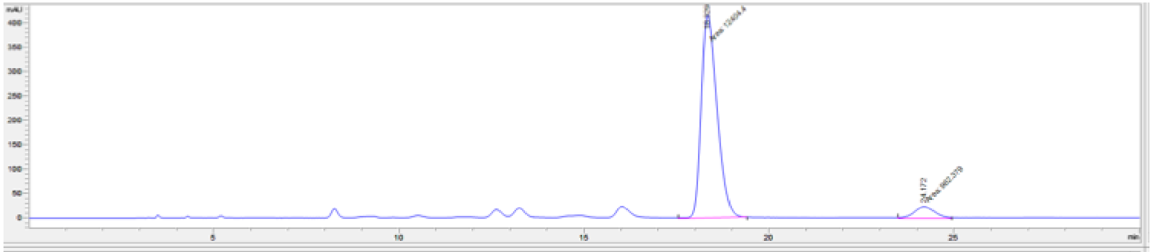

#	Time	Area	Height	Width	Area%	Symmetry
1	18.329	12404.4	416.4	0.4965	92.800	0.638
2	24.172	962.4	24	0.6672	7.200	0.861

GCMS data, **2f**

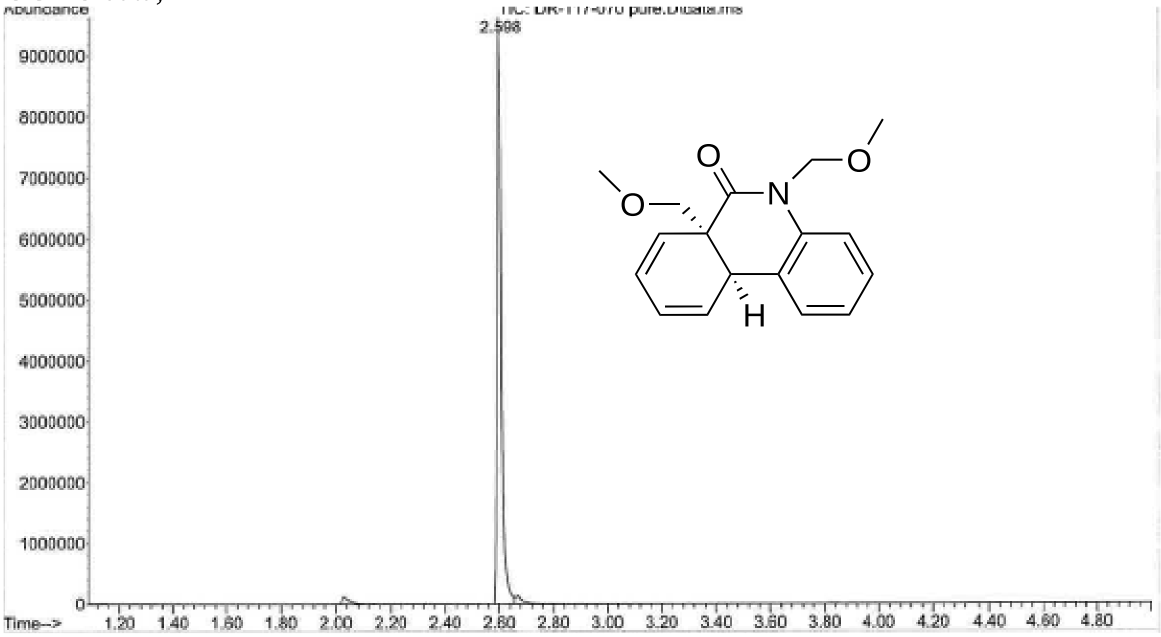

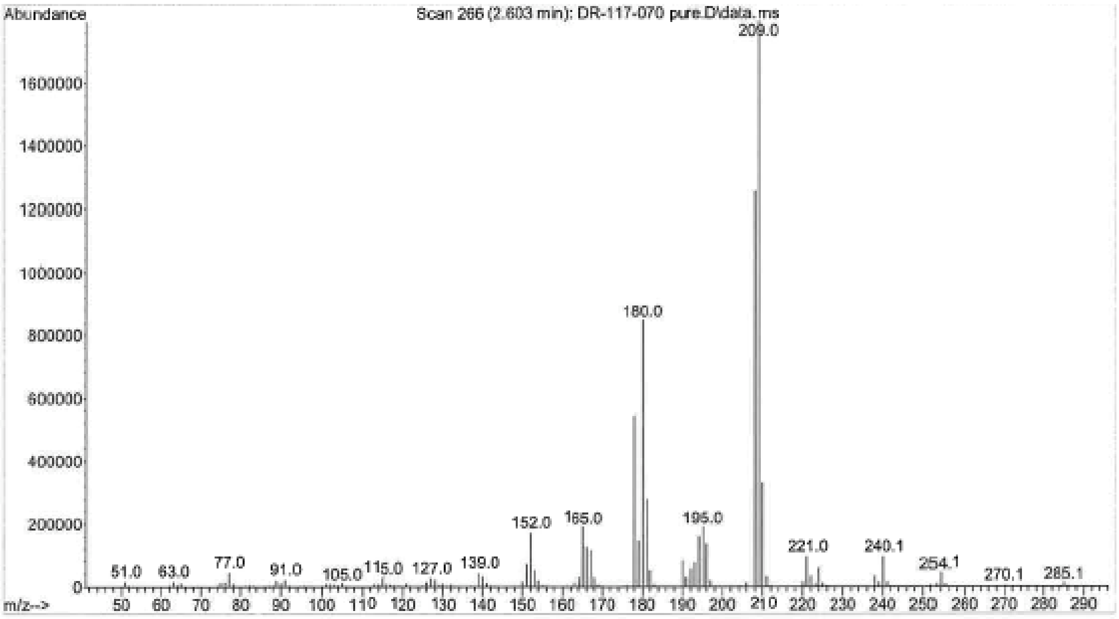

¹H NMR (400 MHz), **2f**

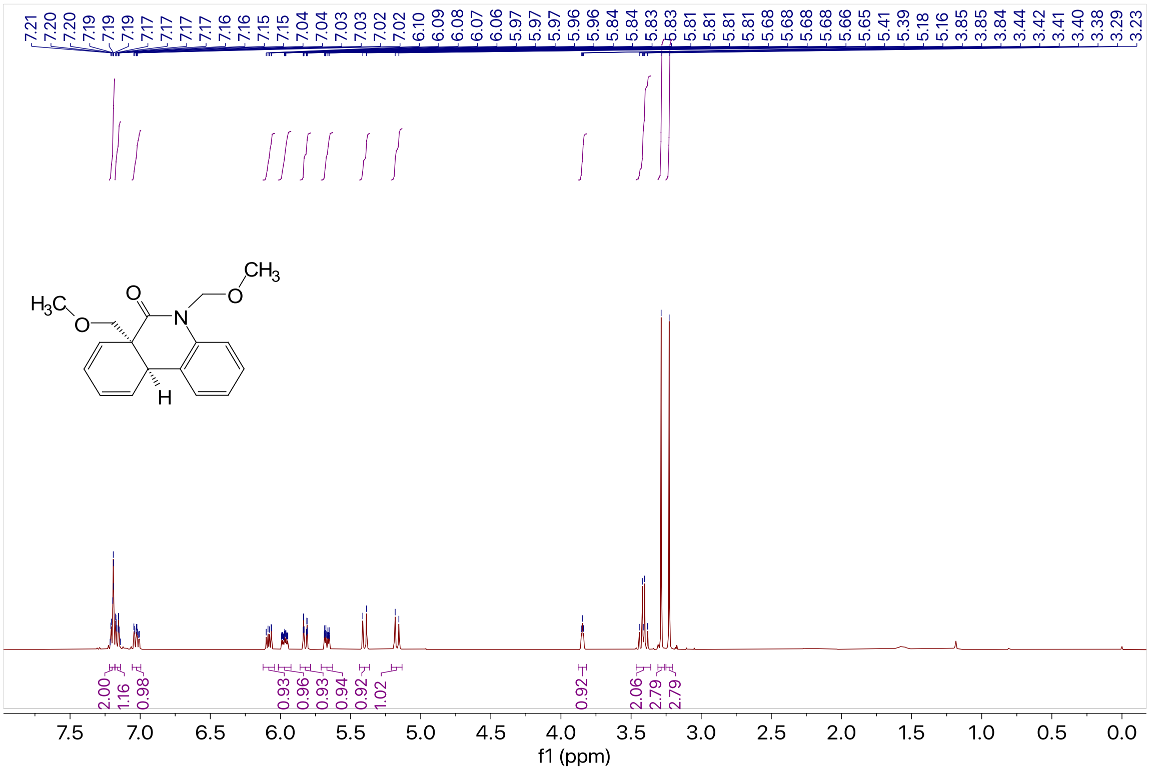

$^{13}C\{^{1}H\}$ *NMR (101 MHz, CDCl$_3$)*, **2f**

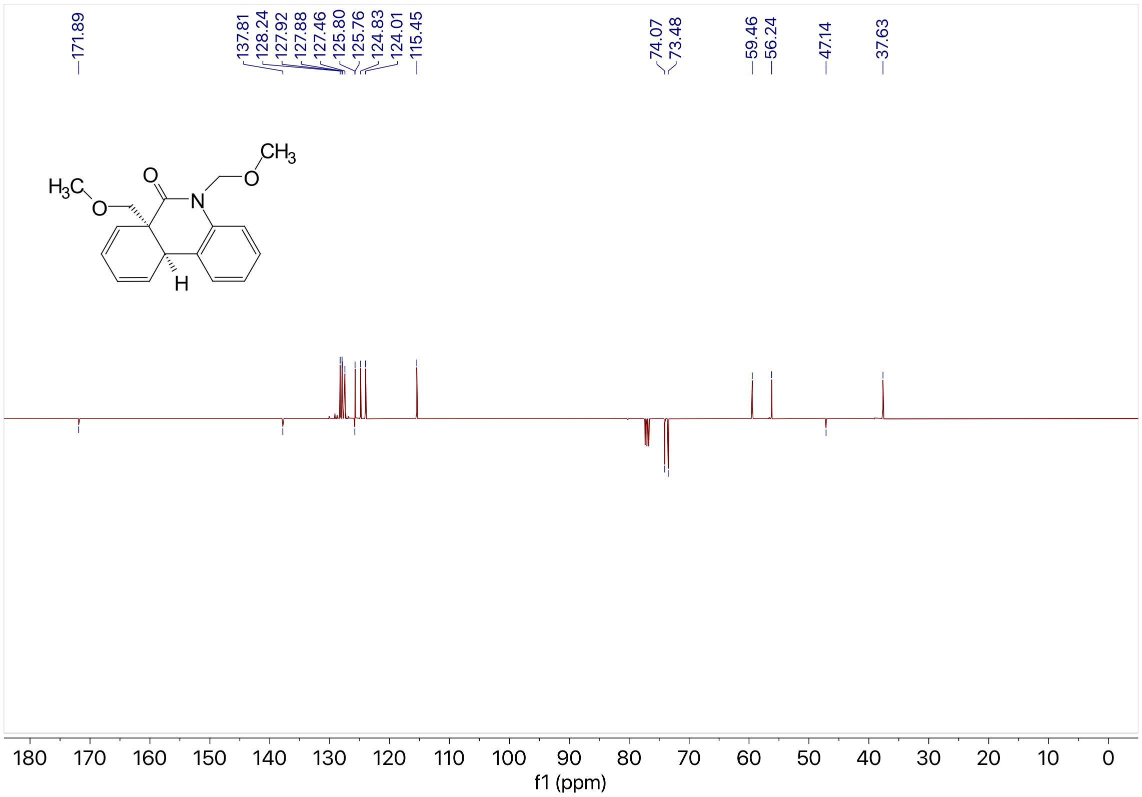

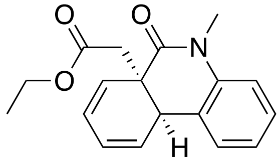

Ethyl 2-((6aR,10aR)-5-methyl-6-oxo-5,10a-dihydrophenanthridin-6a(6H)-yl)acetate (2g).

Chiral LC for racemic **2g** sample

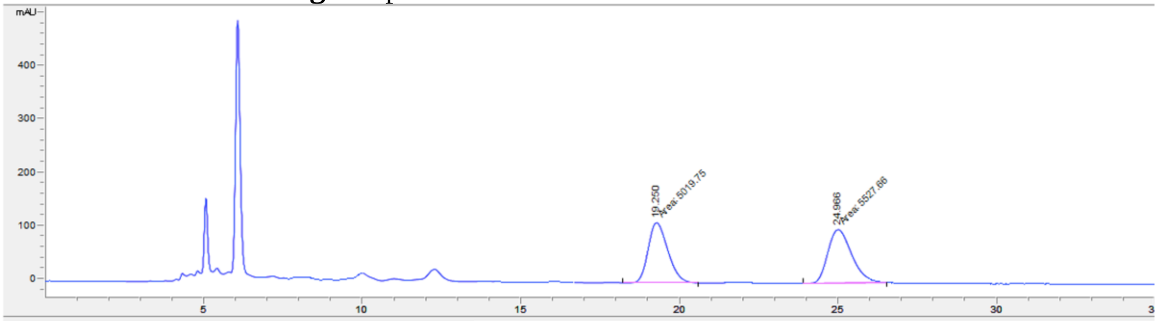

#	Time	Area	Height	Width	Area%	Symmetry
1	19.25	5019.8	114	0.7337	47.592	0.736
2	24.966	5527.7	102.2	0.9014	52.408	0.717

Chiral LC for enantioselective **2g** reaction

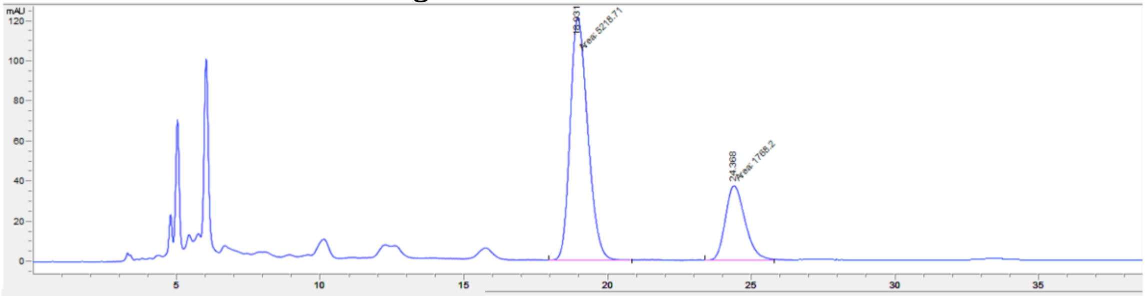

#	Time	Area	Height	Width	Area%	Symmetry
1	18.931	5218.7	121	0.7188	74.693	0.734
2	24.368	1768.2	37.1	0.7952	25.307	0.763

GCMS data, **2g**

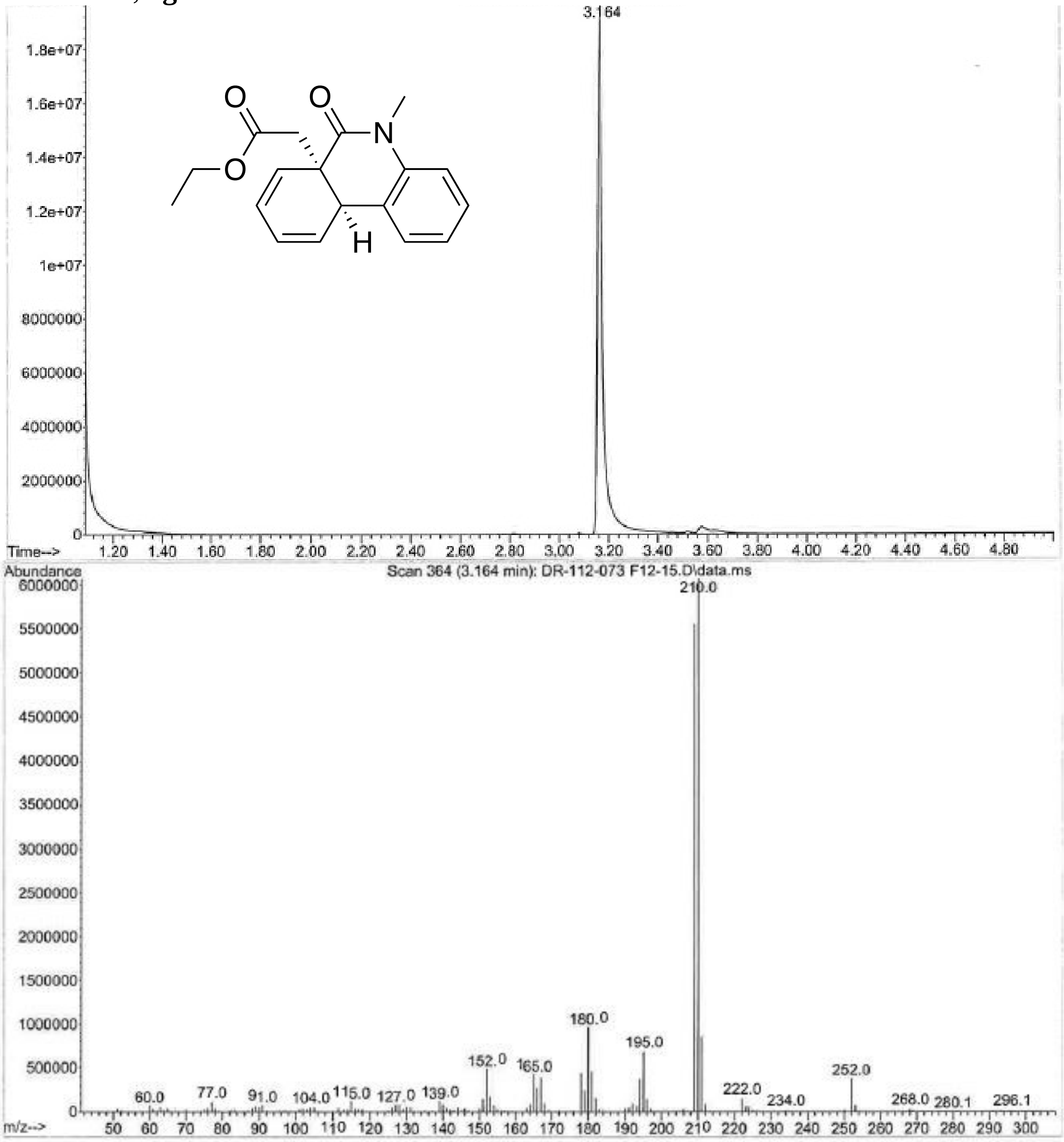

¹H NMR (400 MHz), **2g**

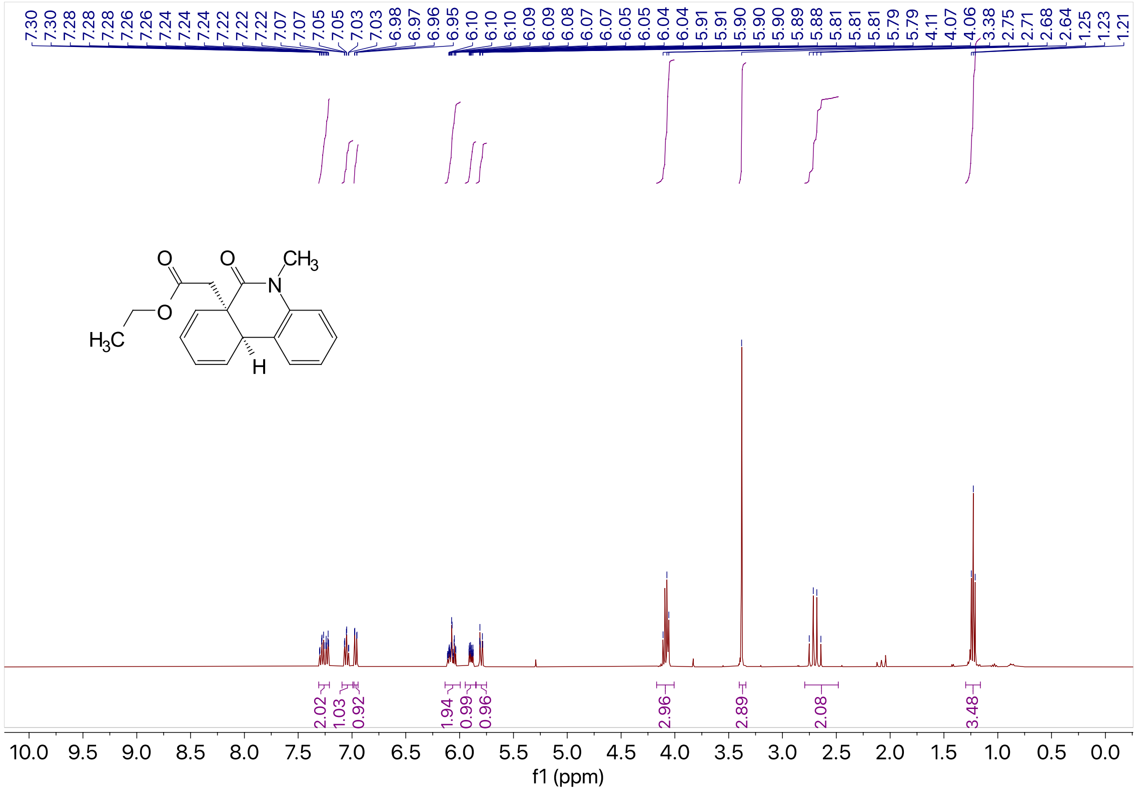

^{13}C{^{1}H} NMR (101 MHz, $CDCl_3$), **2g**

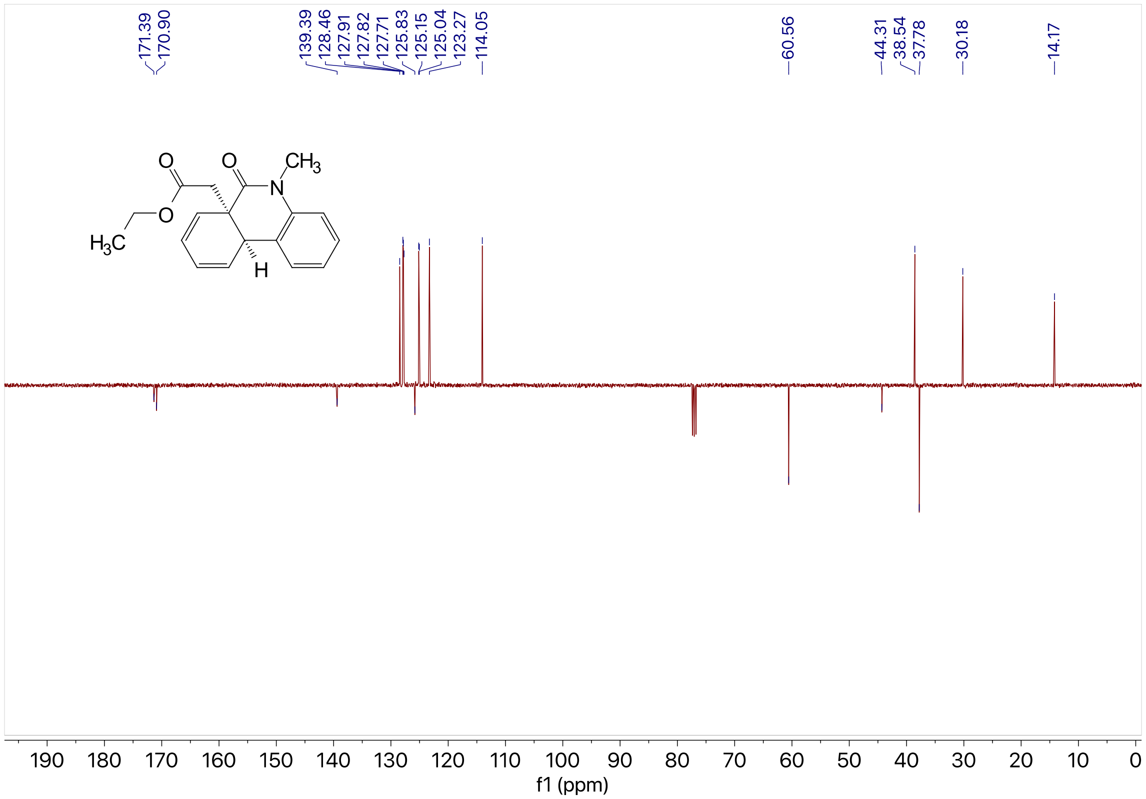

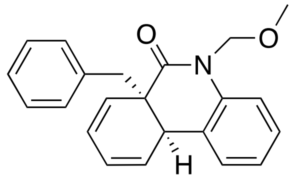

(6aR,10aR)-6a-Benzyl-5-(methoxymethyl)-6a,10a-dihydrophenanthridin-6(5H)-one (2h).

Chiral LC for racemic **2h** sample

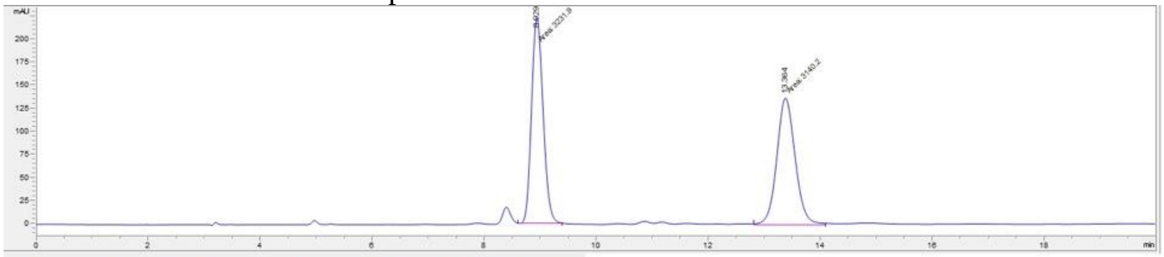

#	Time	Area	Height	Width	Area%	Symmetry
1	8.929	3231.9	223.7	0.2407	50.720	0.833
2	13.364	3140.2	137.6	0.3804	49.280	0.878

Chiral LC for enantioselective **2h** reaction

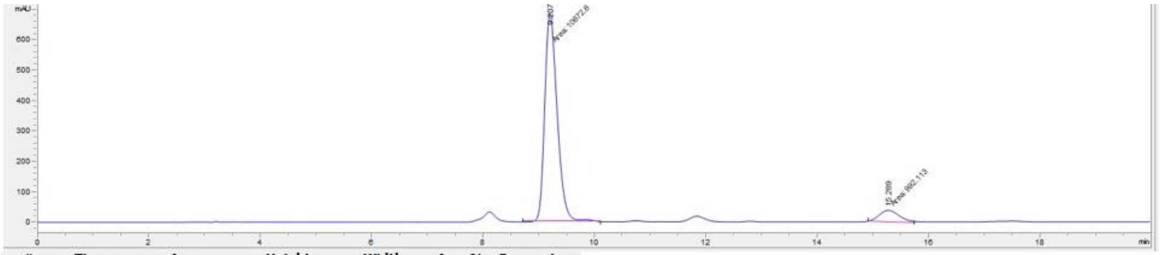

#	Time	Area	Height	Width	Area%	Symmetry
1	9.207	10672.6	680.1	0.2615	91.495	0.692
2	15.289	992.1	38.7	0.4271	8.505	0.814

GCMS data, **2h**

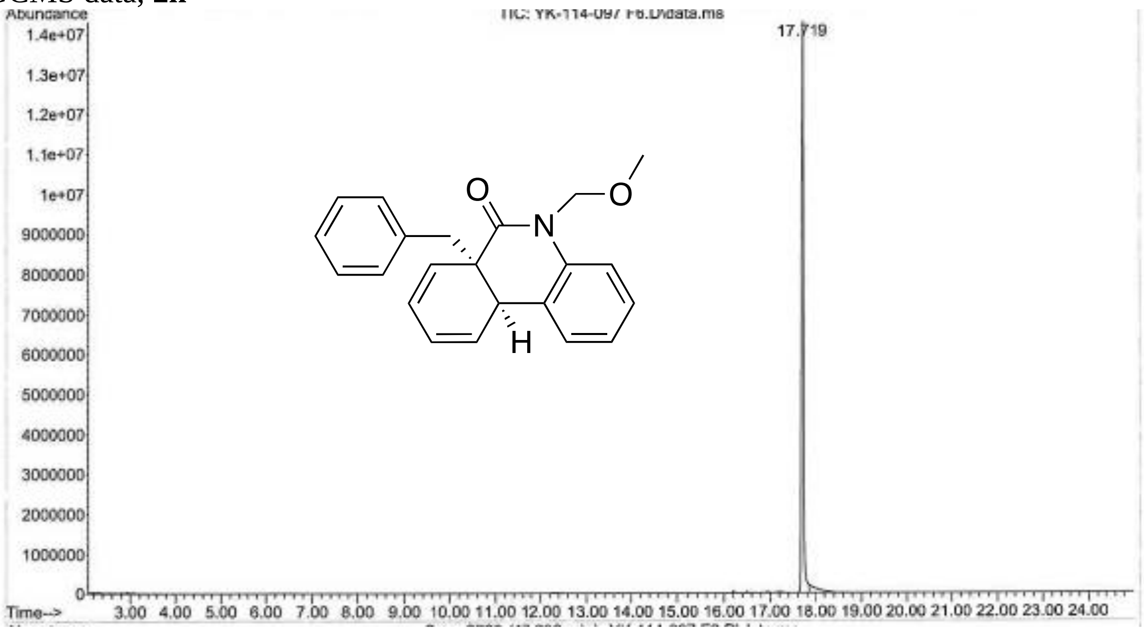

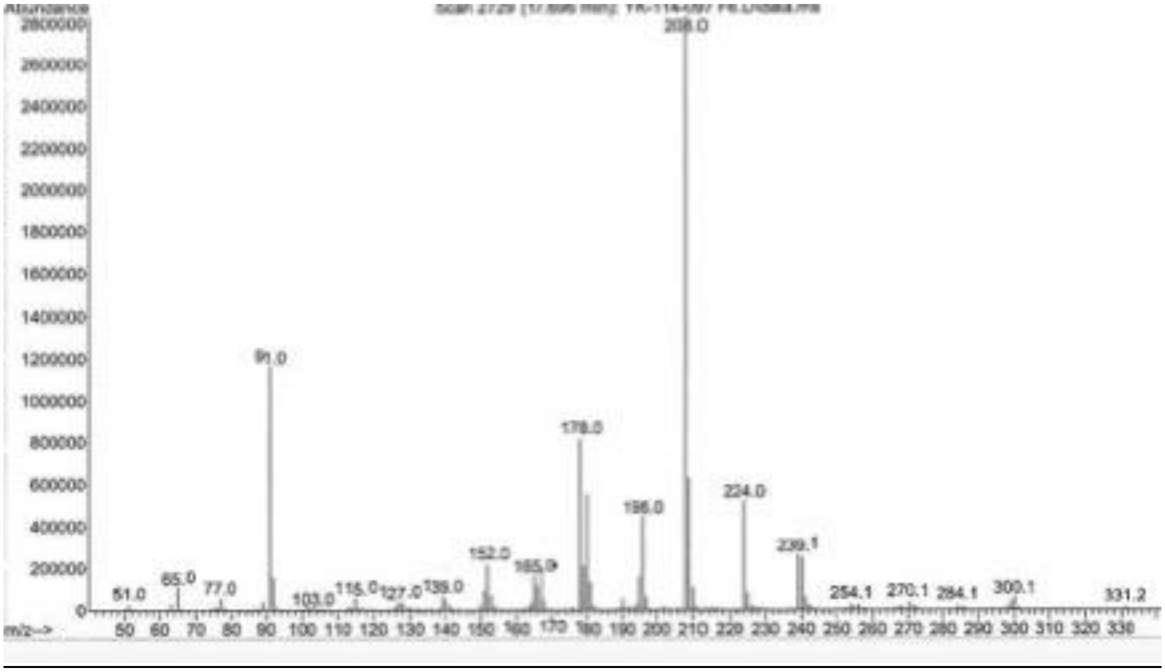

^{1}H NMR (400 MHz), **2h**

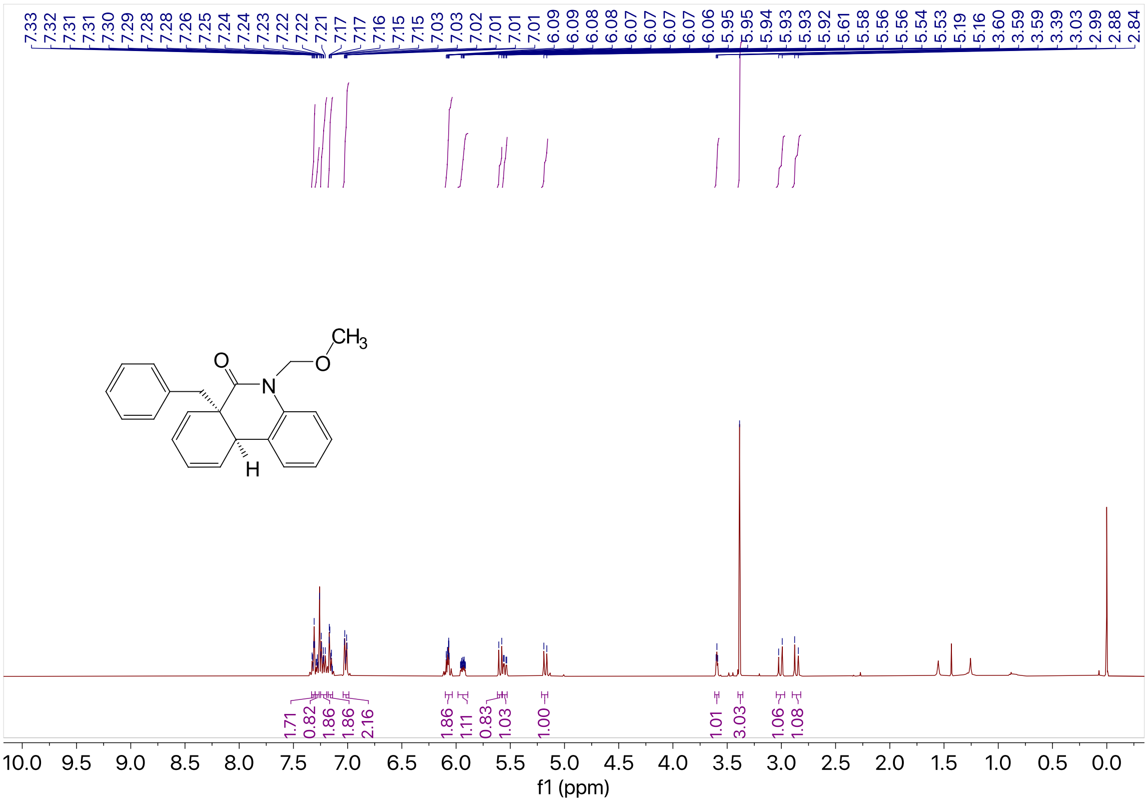

$^{13}C\{^{1}H\}$ *NMR (101 MHz, CDCl₃)*, **2h**

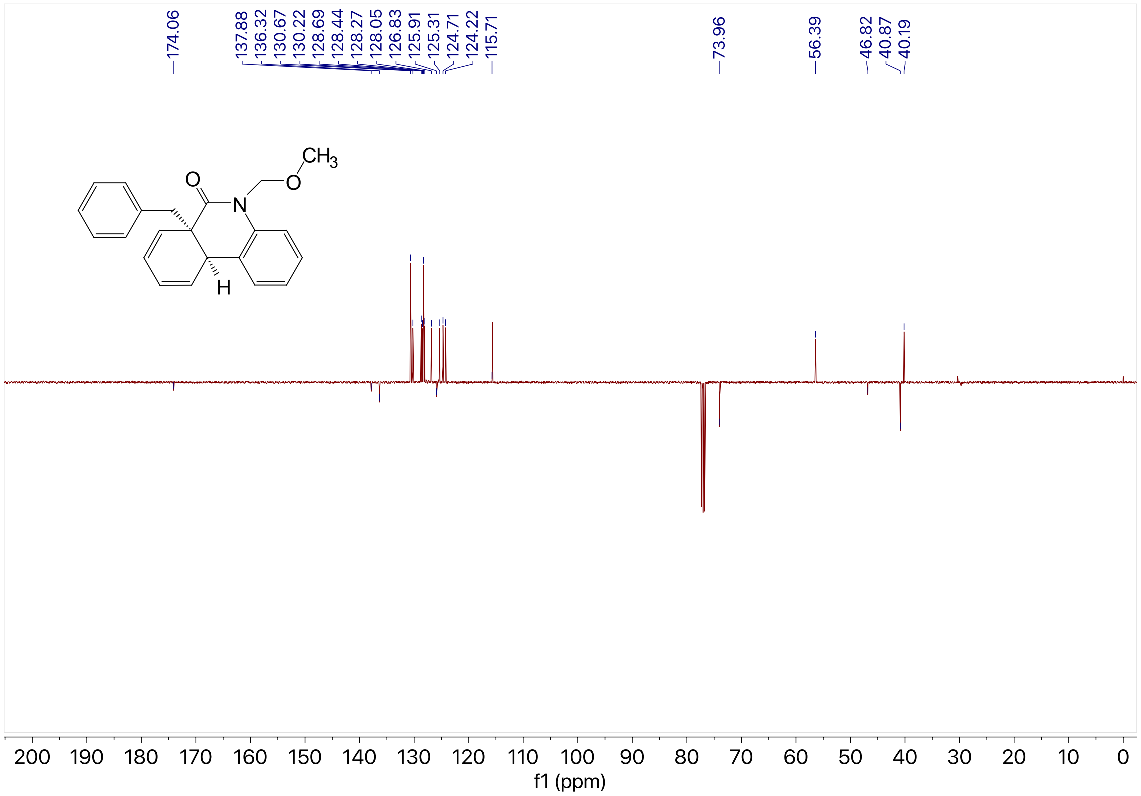

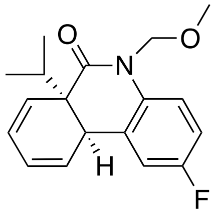

(6a*R*,10a*R*)-2-Fluoro-6a-isopropyl-5-(methoxymethyl)-6a,10a-dihydrophenanthridin-6(5*H*)-one (2i).

Chiral LC for racemic **2i** sample

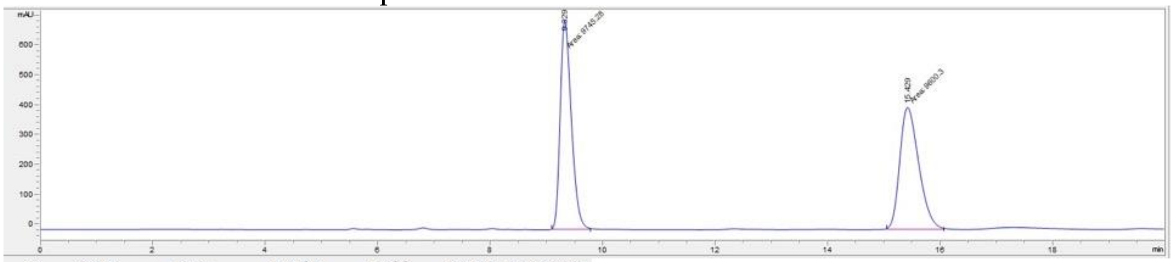

#	Time	Area	Height	Width	Area%	Symmetry
1	9.329	9745.3	701.2	0.2316	50.375	0.685
2	15.429	9600.3	408.4	0.3918	49.625	0.681

Chiral LC for enantioselective **2i** reaction

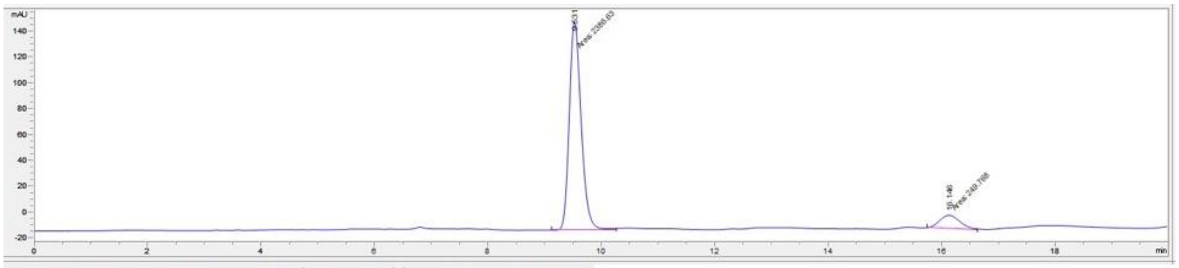

#	Time	Area	Height	Width	Area%	Symmetry
1	9.531	2386.6	162.2	0.2453	90.526	0.755
2	16.146	249.8	10.1	0.4121	9.474	0.772

GCMS data, **2i**

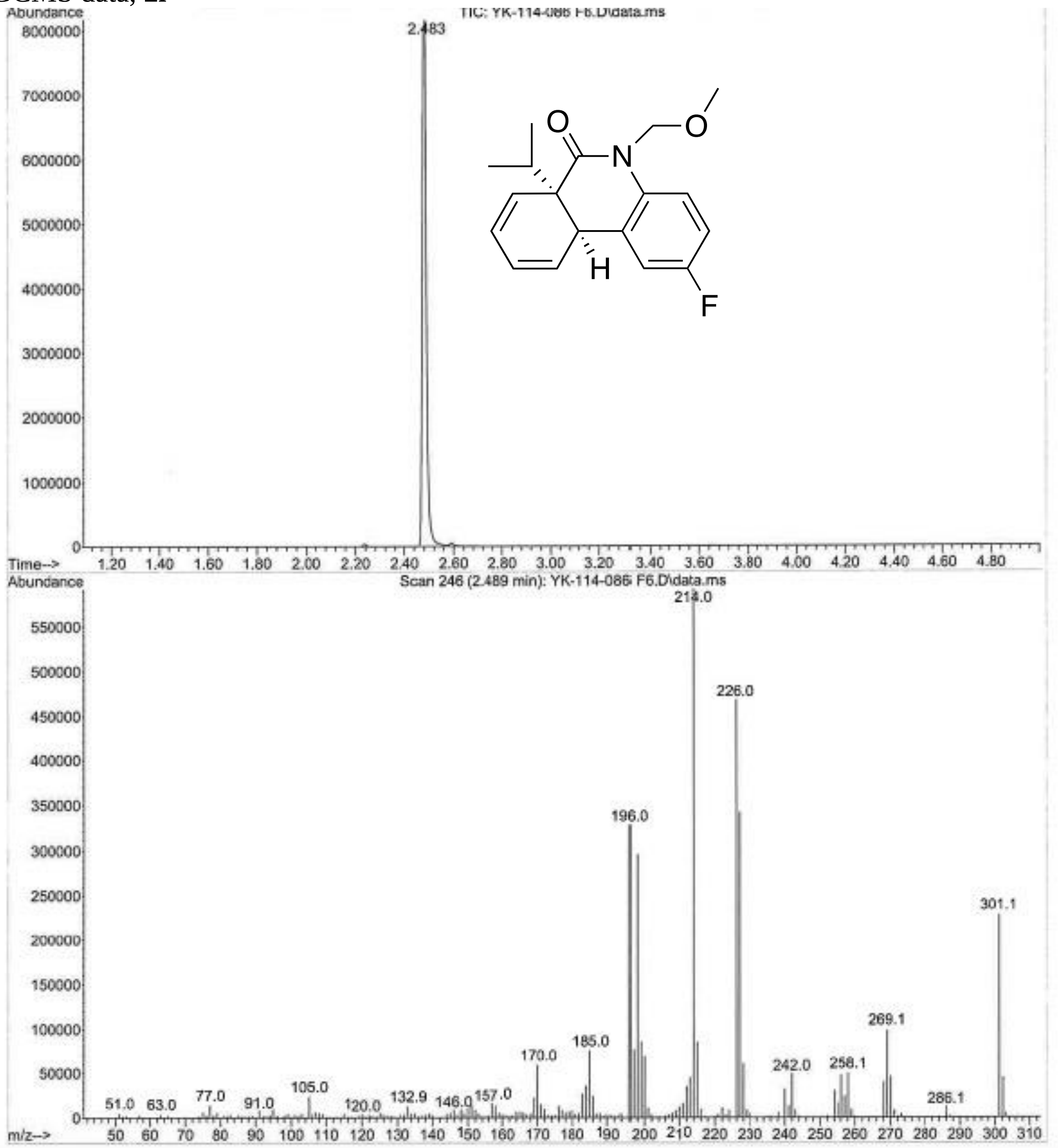

^{1}H NMR (400 MHz), **2i**

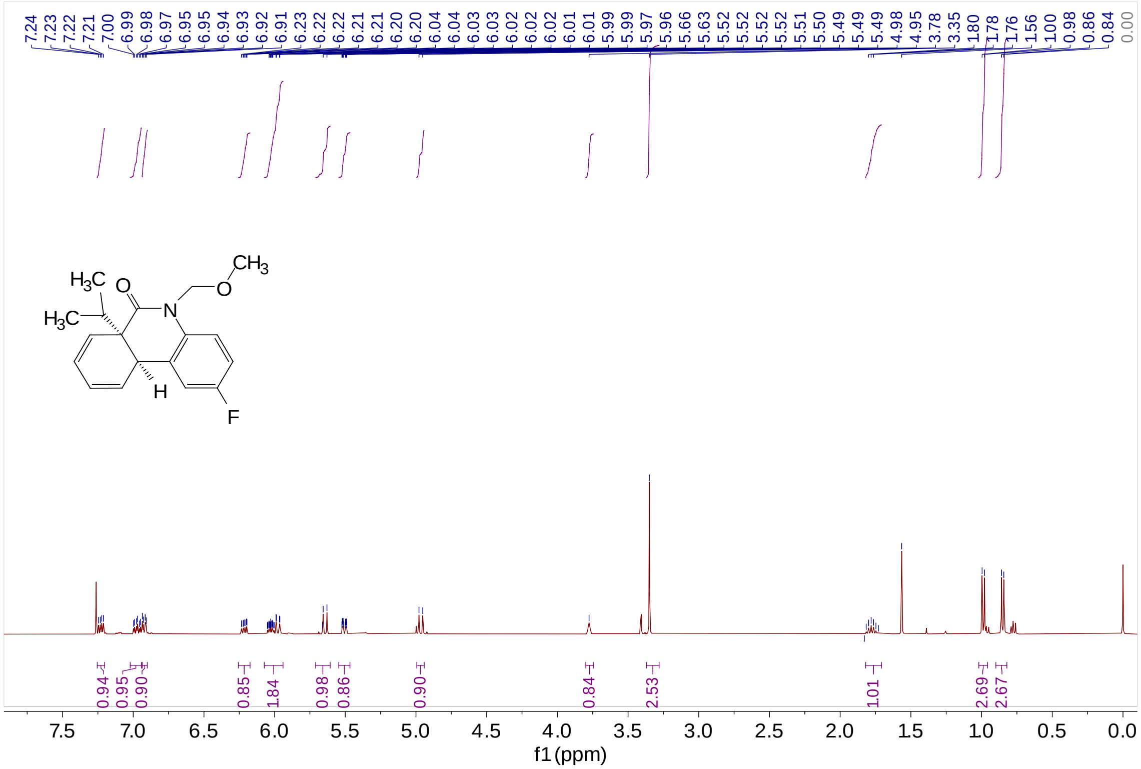

^{13}C{^{1}H} NMR (101 MHz, $CDCl_3$), **2i**

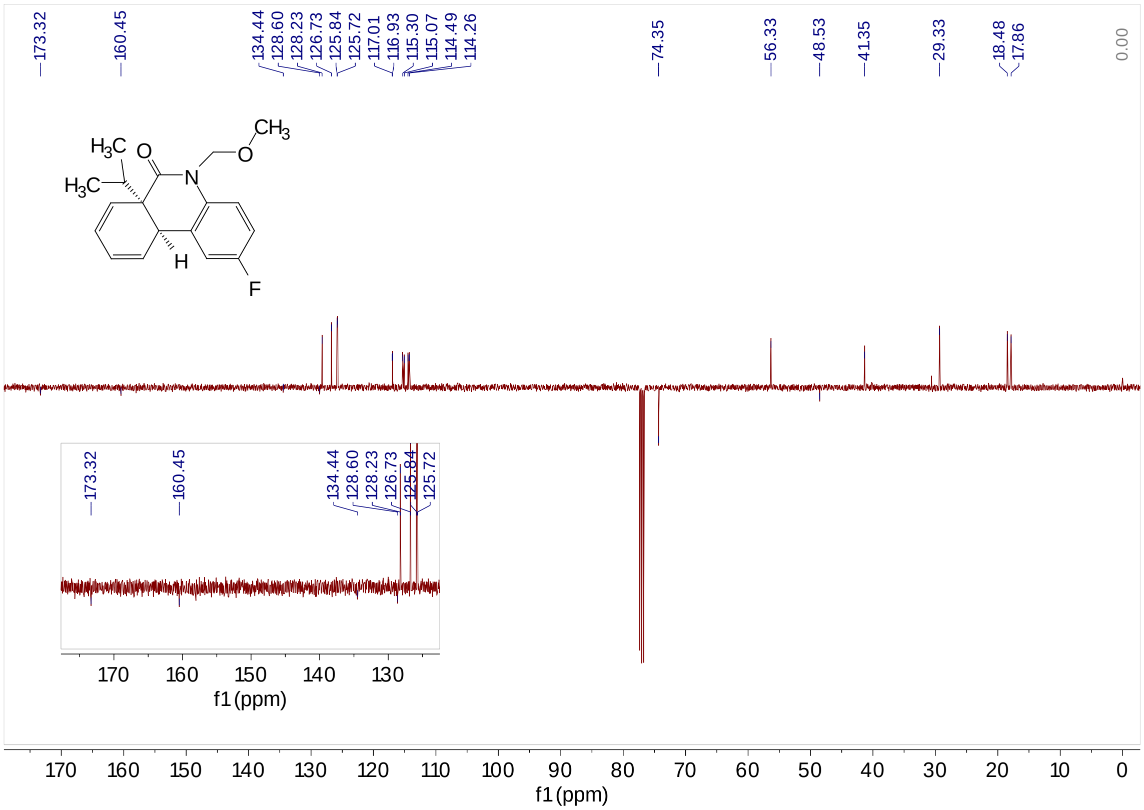

^{19}F NMR (376 MHz), **2i**

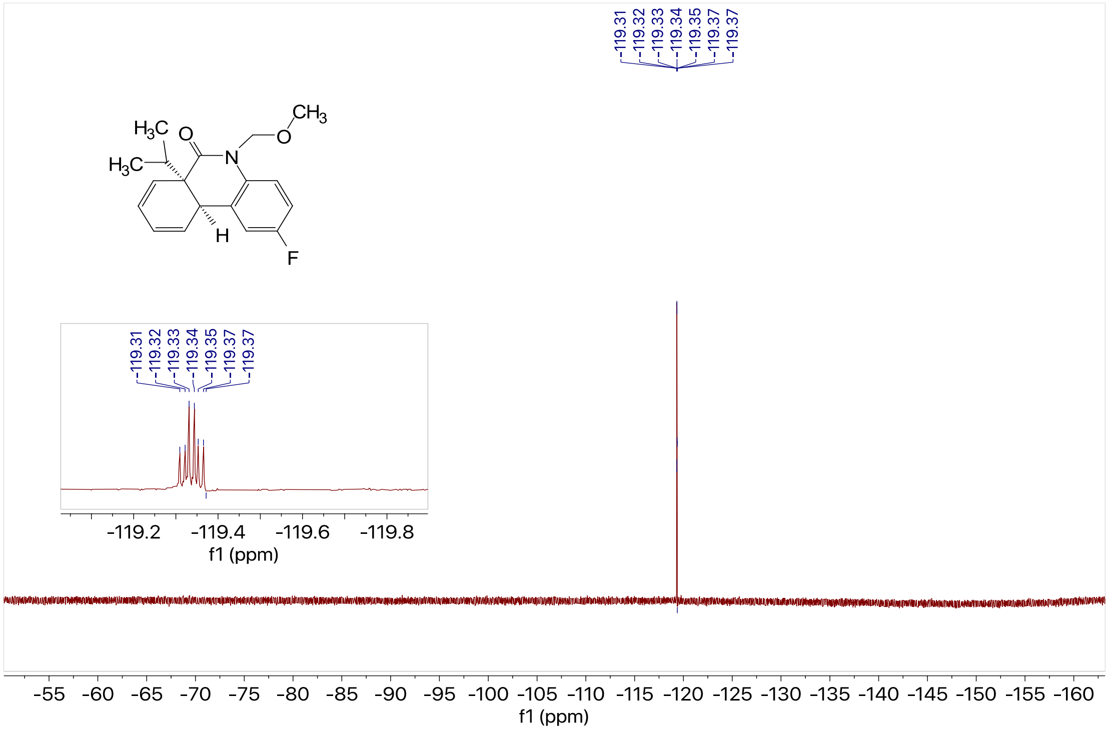

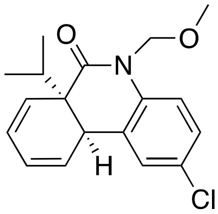

(6aR,10aR)-2-Chloro-6a-isopropyl-5-(methoxymethyl)-6a,10a-dihydrophenanthridin-6(5H)-one (2j).

Chiral LC for racemic **2j** sample

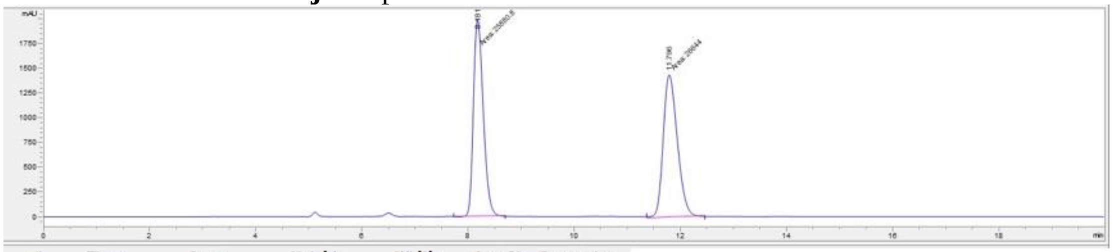

#	Time	Area	Height	Width	Area%	Symmetry
1	8.181	25880.8	1992.4	0.2165	49.274	0.704
2	11.796	26644	1439.7	0.3084	50.726	0.727

Chiral LC for enantioselective **2j** reaction

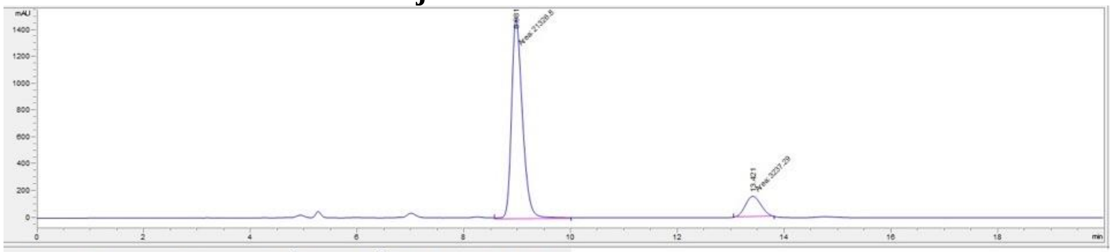

#	Time	Area	Height	Width	Area%	Symmetry
1	8.981	21326.8	1486.7	0.2391	86.821	0.675
2	13.421	3237.3	155.6	0.3467	13.179	0.894

GCMS data, **2j**

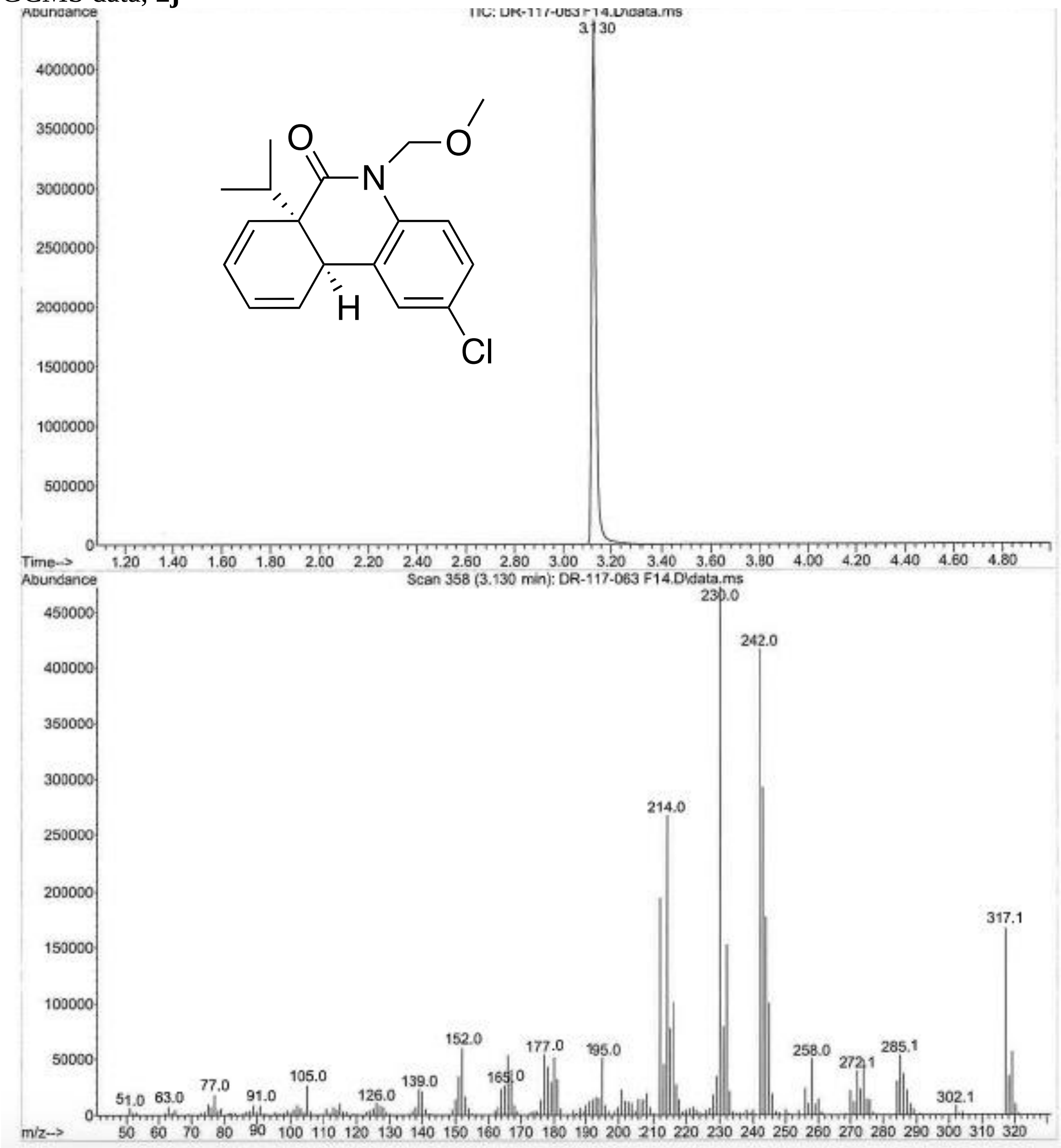

Abundance
TIC: DR-117-063 F14.D\data.ms
3.130
4000000
3500000
3000000
2500000
2000000
1500000
1000000
500000
0
Time-->
1.20 1.40 1.60 1.80 2.00 2.20 2.40 2.60 2.80 3.00 3.20 3.40 3.60 3.80 4.00 4.20 4.40 4.60 4.80
O
N
O
H
Cl
Abundance
Scan 358 (3.130 min): DR-117-063 F14.D\data.ms
230.0
242.0
214.0
317.1
152.0
177.0
195.0
165.0
258.0
272.1
285.1
302.1
139.0
126.0
105.0
91.0
77.0
63.0
51.0
450000
400000
350000
300000
250000
200000
150000
100000
50000
0
m/z-->
50 60 70 80 90 100 110 120 130 140 150 160 170 180 190 200 210 220 230 240 250 260 270 280 290 300 310 320

^{1}H *NMR (400 MHz)*, **2j**

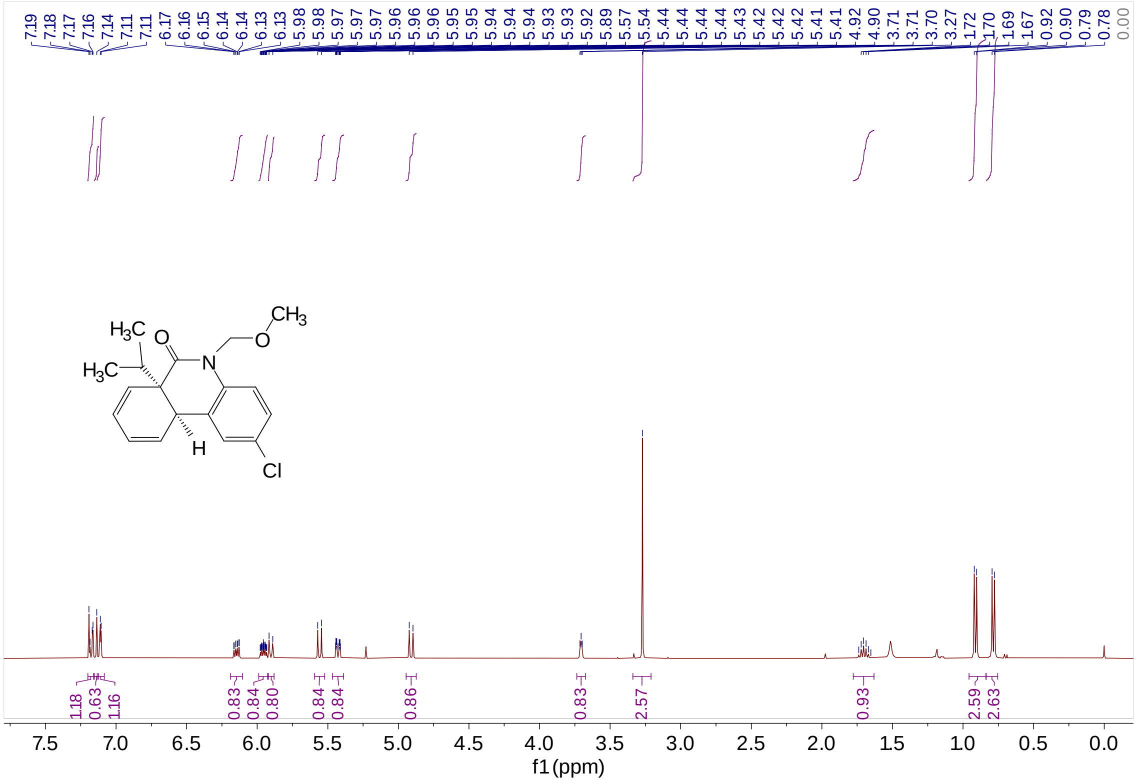

$^{13}C\{^{1}H\}$ *NMR (101 MHz, $CDCl_3$)*, **2j**

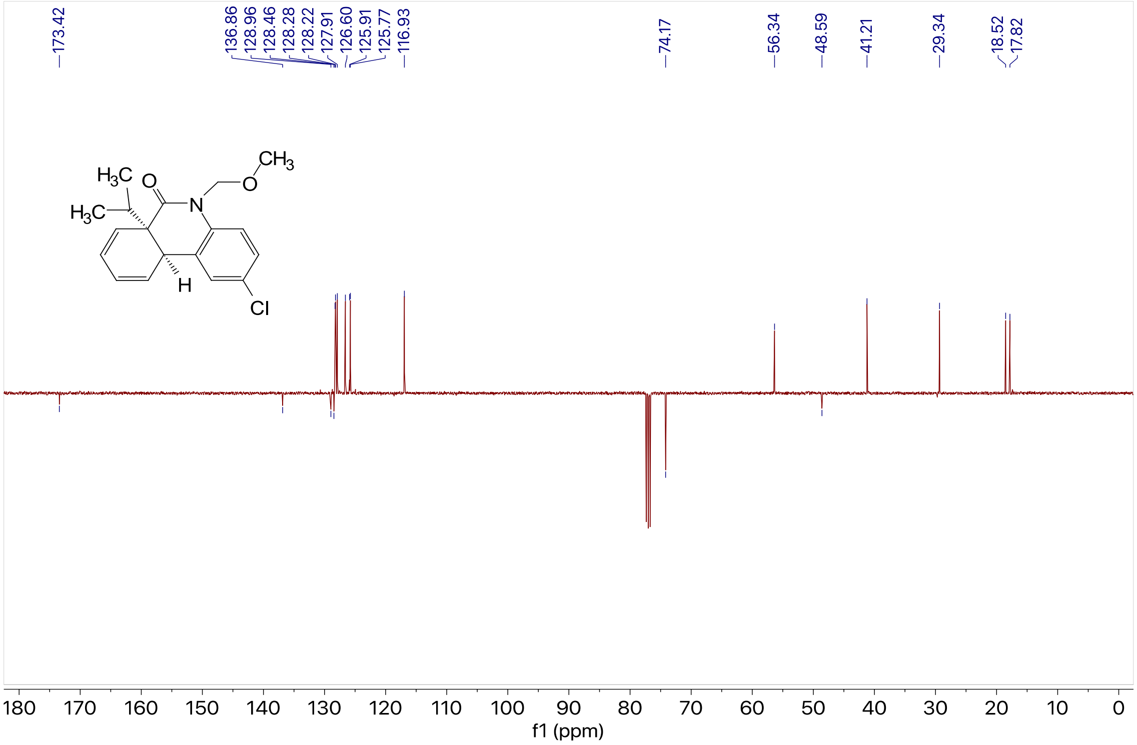

6a-Isopropyl-5-(methoxymethyl)-2-methyl-6a,10a-dihydrophenanthridin-6(5*H*)-one (2k).

^{1}H NMR (400 MHz), **2k**

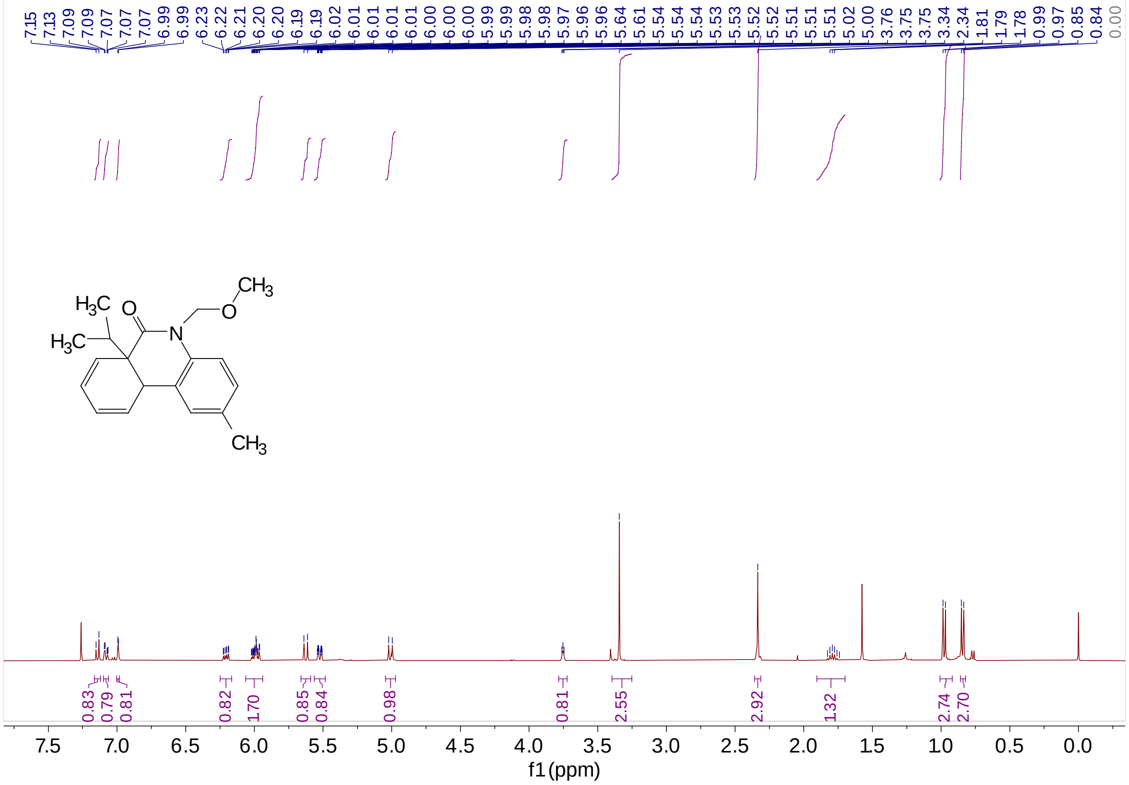

^{13}C{^{1}H} NMR (101 MHz, $CDCl_3$), **2k**

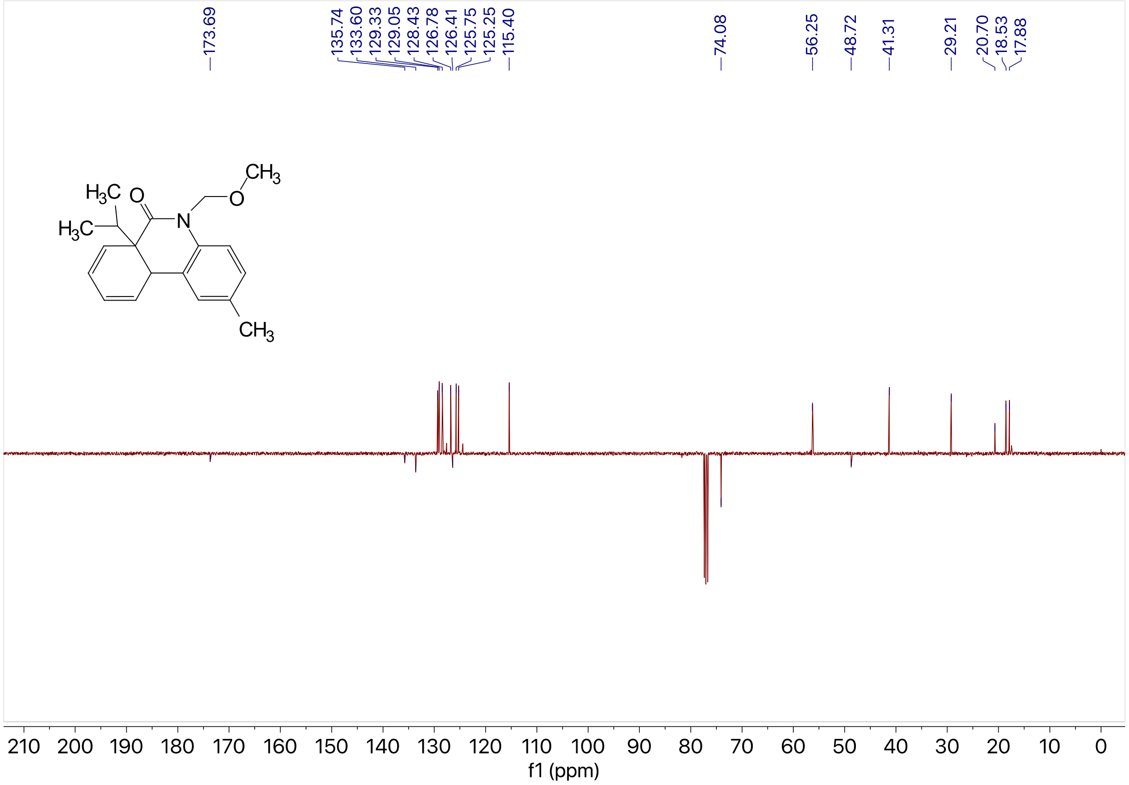

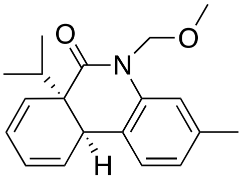

(6aR,10aR)-6a-Isopropyl-5-(methoxymethyl)-3-methyl-6a,10a-dihydrophenanthridin-6(5H)-one (2l).

Chiral LC for racemic **2l** sample

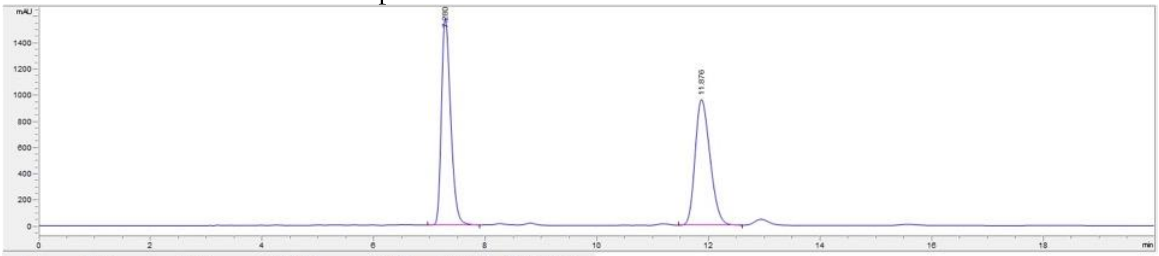

#	Time	Area	Height	Width	Area%	Symmetry
1	7.28	18318.5	1582.2	0.1771	50.433	0.661
2	11.876	18004.1	962.8	0.2902	49.567	0.718

Chiral LC for enantioselective **2l** reaction

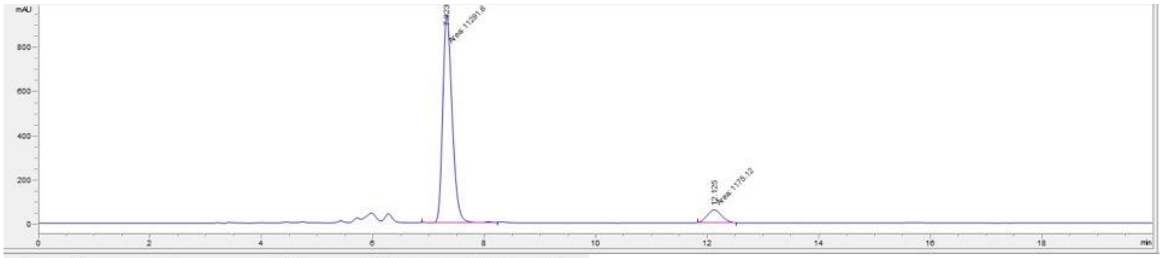

#	Time	Area	Height	Width	Area%	Symmetry
1	7.323	11291.6	938.3	0.2006	90.574	0.714
2	12.125	1175.1	60.6	0.323	9.426	0.891

GCMS data, **2l**

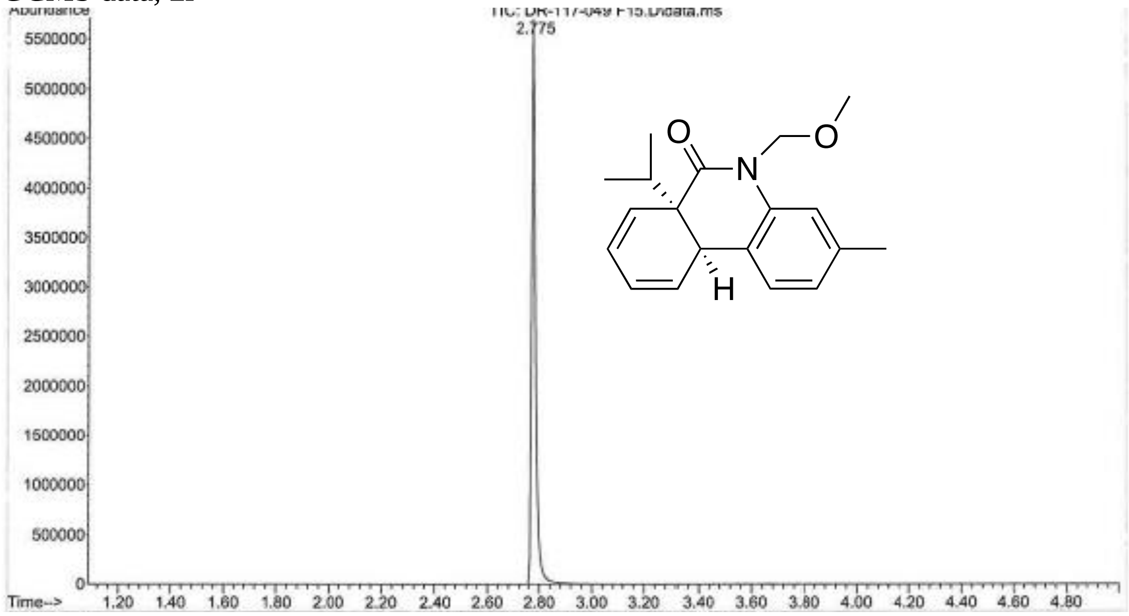

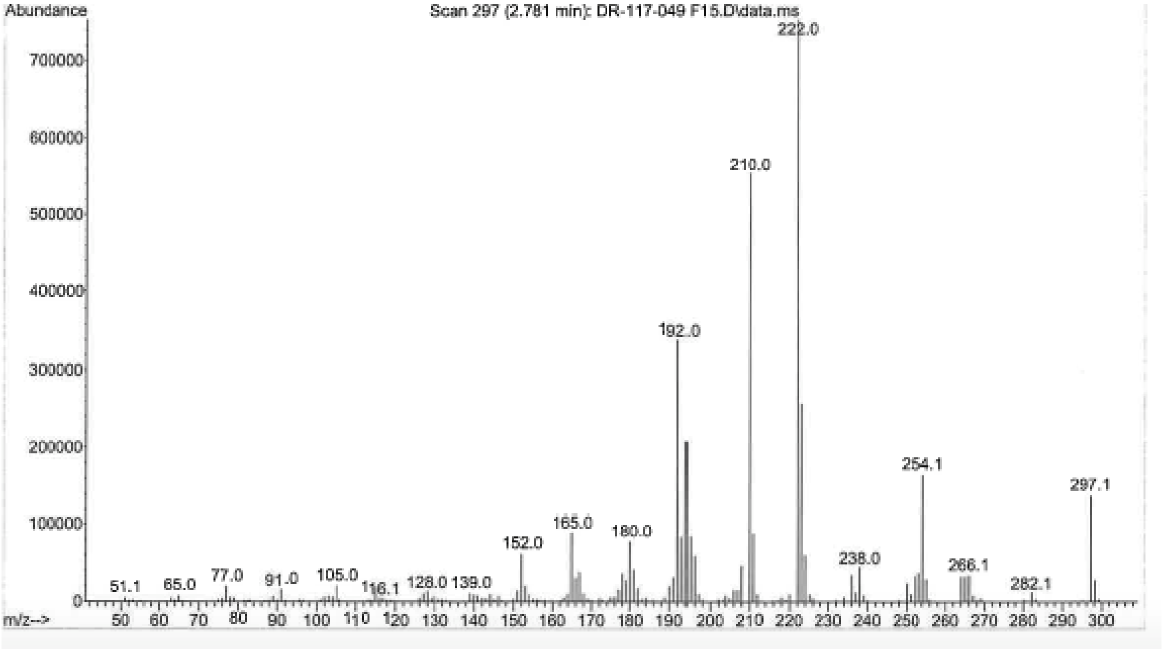

^{1}H NMR (400 MHz), **2l**

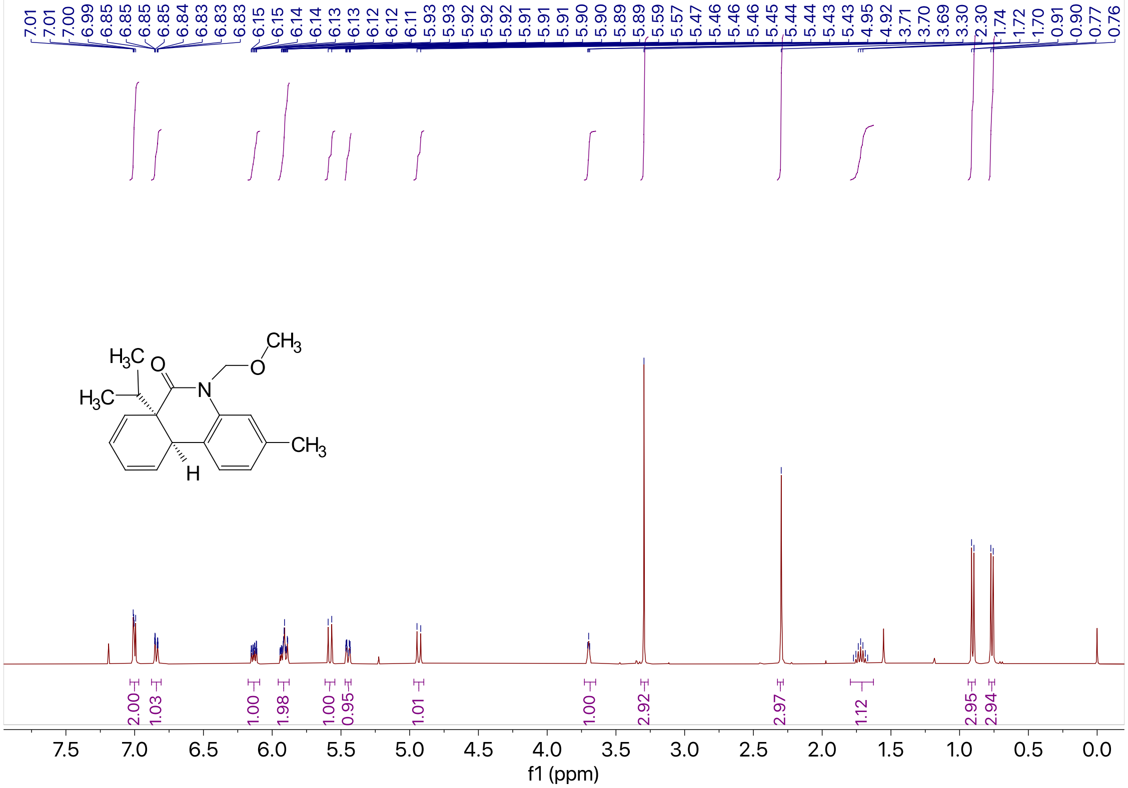

$^{13}C\{^{1}H\}$ NMR (101 MHz, $CDCl_3$), **2l**

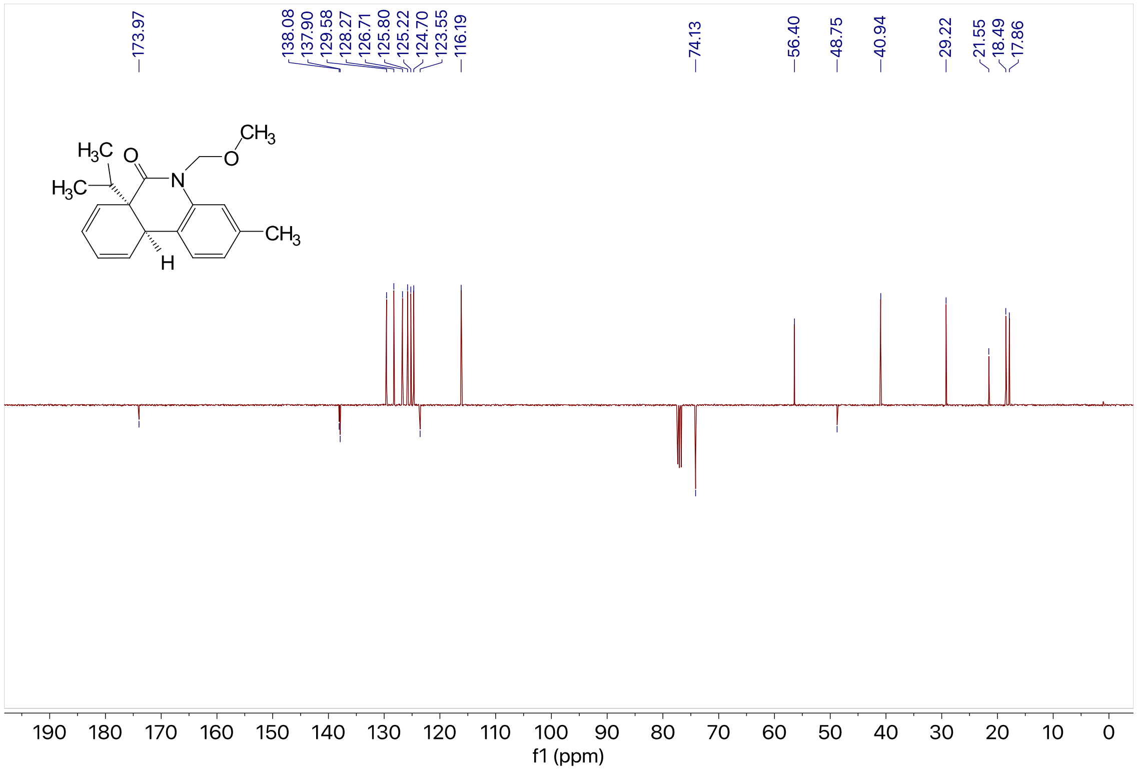

5-(Methoxymethyl)-6a-methyl-6a,9,10,10a-tetrahydrobenzo[*c*][1,5]naphthyridin-6(5*H*)-one (2m-2).

^{1}H NMR (400 MHz), **2m-2**

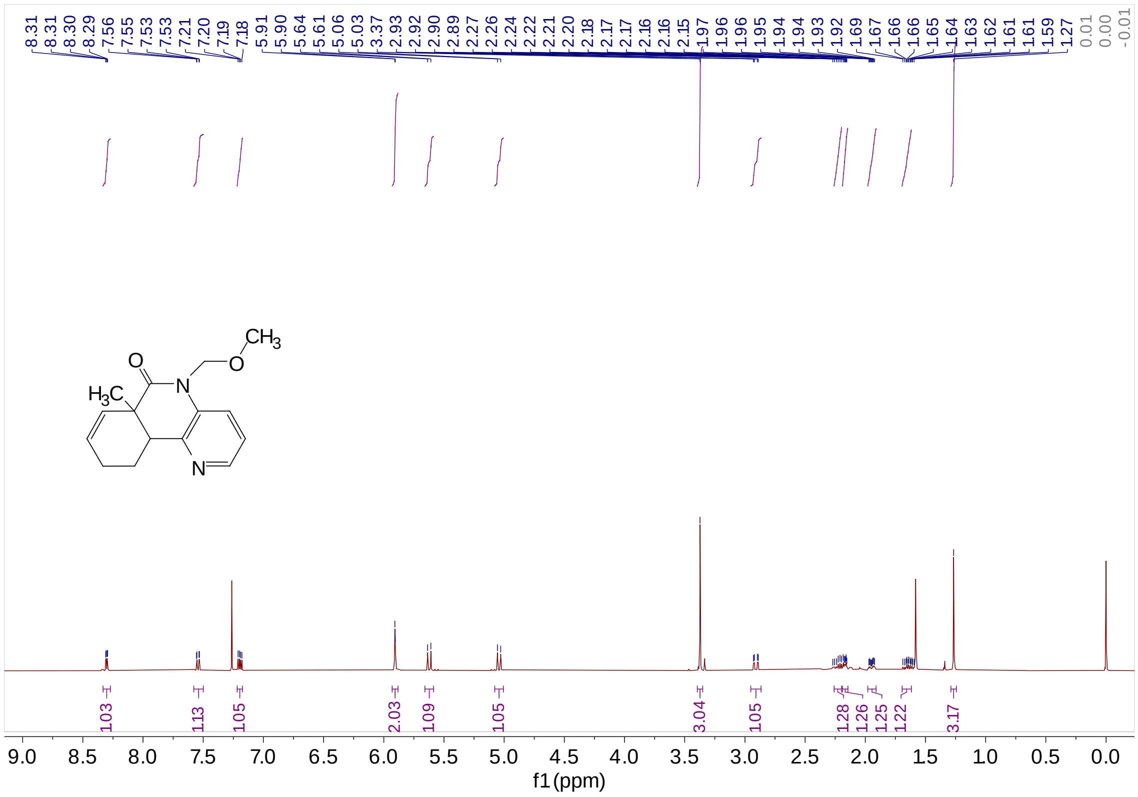

$^{13}C\{^1H\}$ *NMR (101 MHz, CDCl$_3$)*, **2m-2**

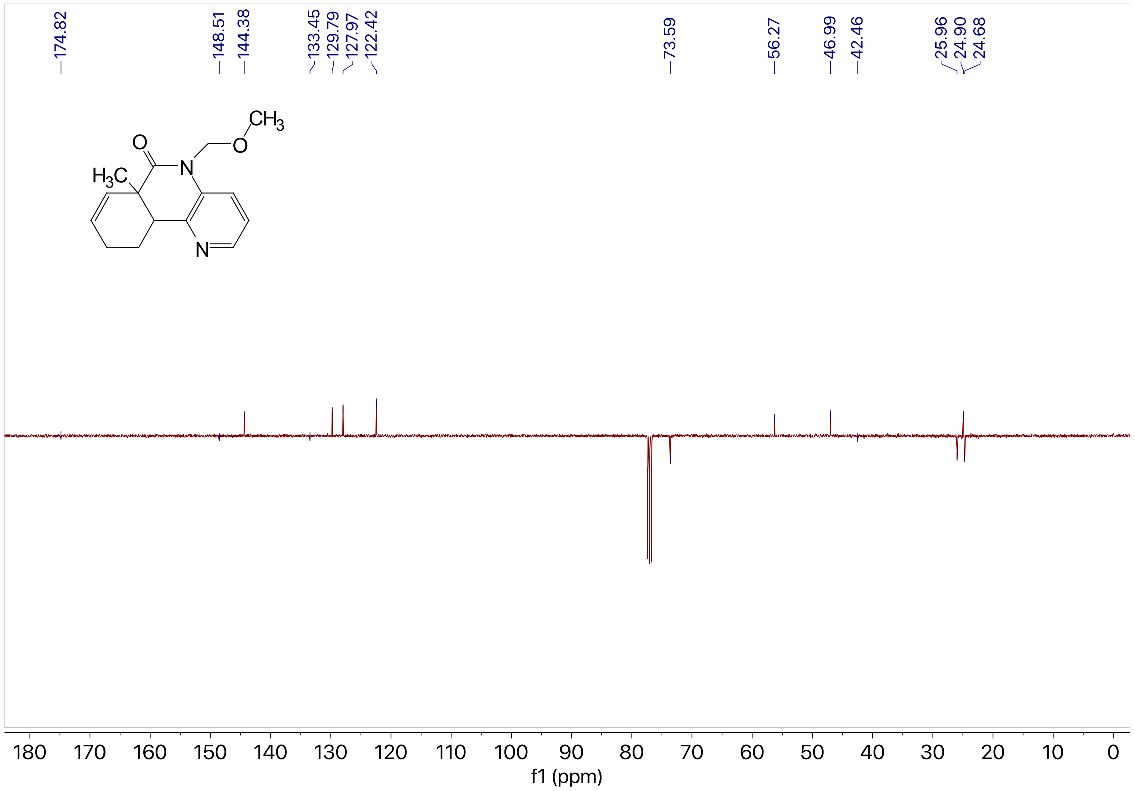

5-(Methoxymethyl)-6a-methyl-6a,10a-dihydrobenzo[*c*][1,8]naphthyridin-6(5*H*)-one (2n).

^1H *NMR (400 MHz)*, **2n**

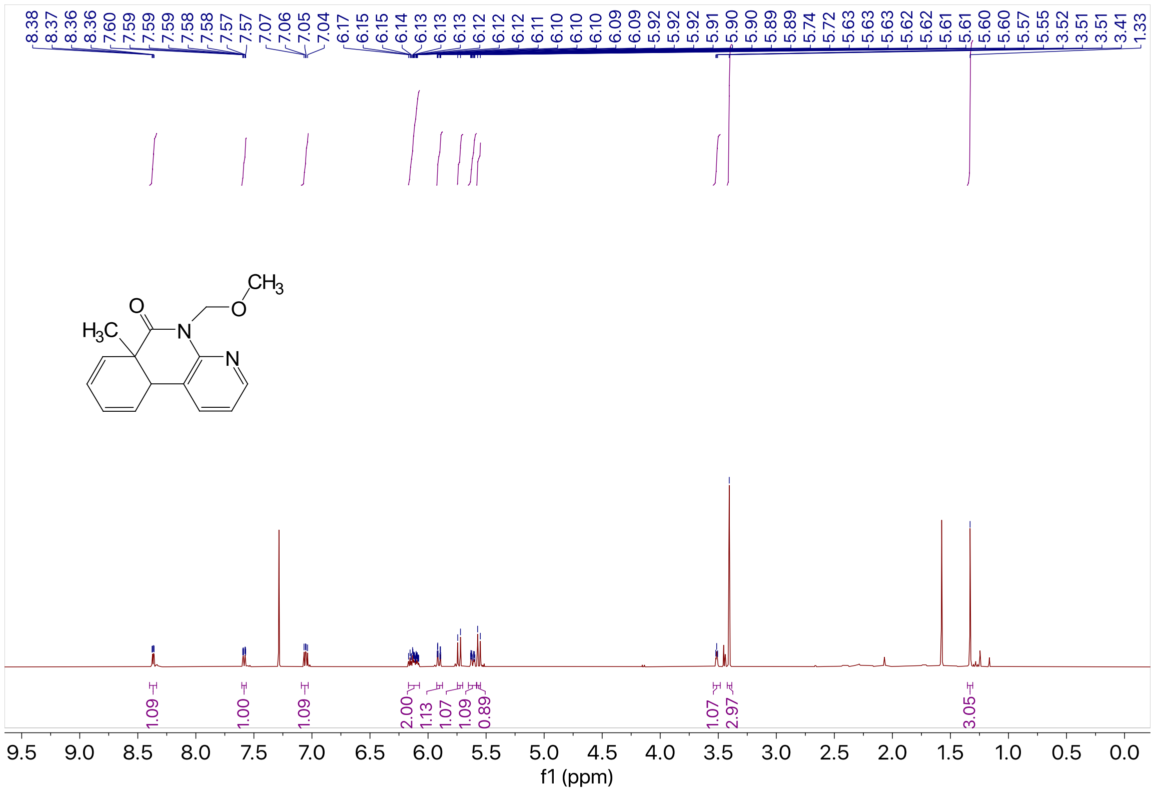

$^{13}C\{^{1}H\}$ *NMR (101 MHz, CDCl$_3$),* **2n**

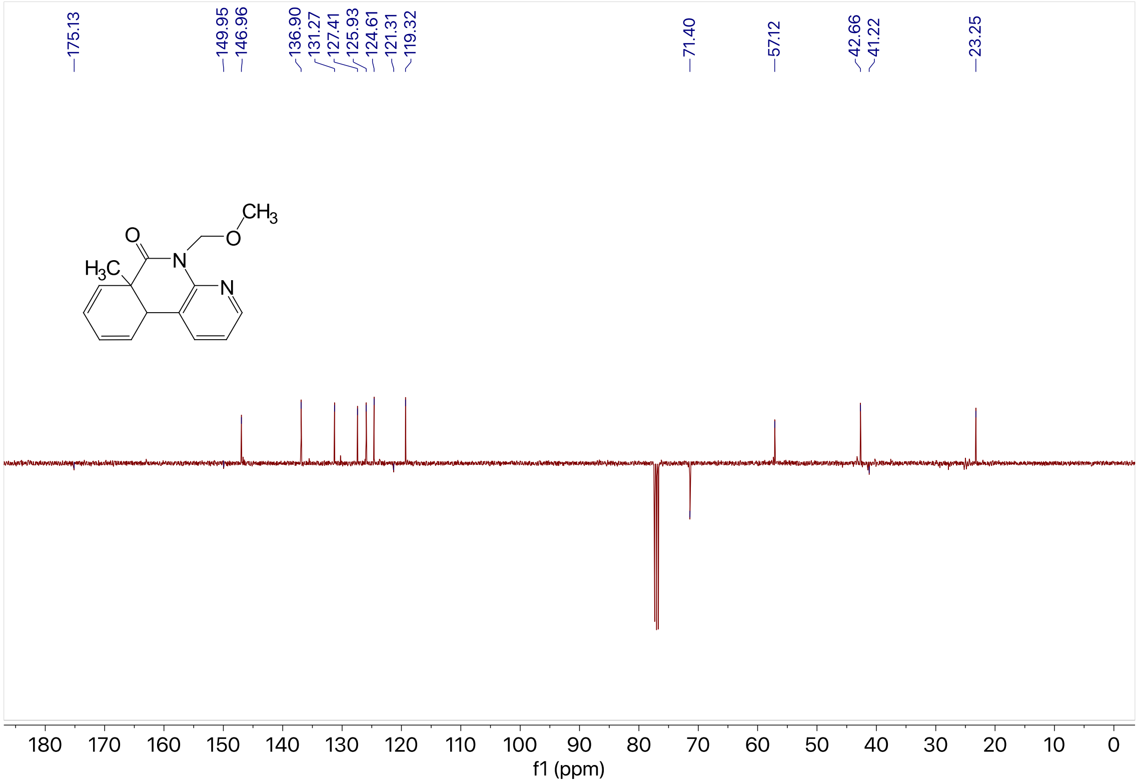

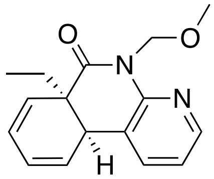

(6aR,10aR)-6a-Ethyl-5-(methoxymethyl)-6a,10a-dihydrobenzo[c][1,8]naphthyridin-6(5H)-one (2o).

Chiral LC for racemic **2o** sample

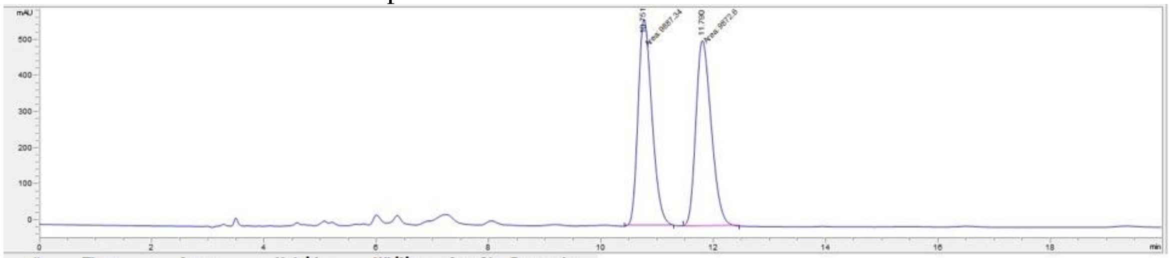

#	Time	Area	Height	Width	Area%	Symmetry
1	10.751	9887.3	573.2	0.2875	50.037	0.723
2	11.79	9872.6	516	0.3189	49.963	0.718

Chiral LC for enantioselective **2o** crude reaction (with **L26**)

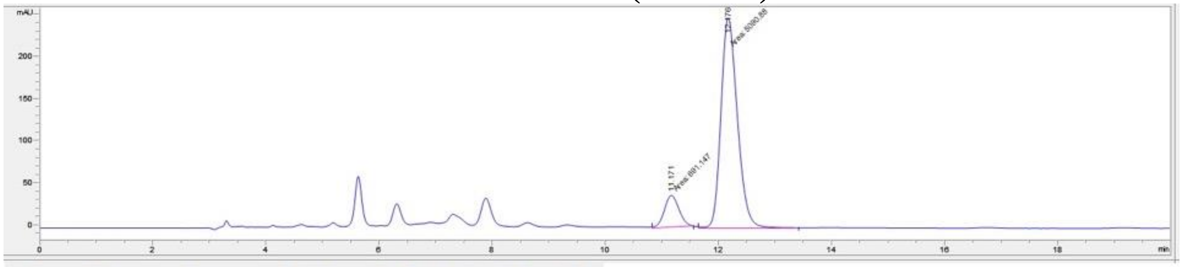

#	Time	Area	Height	Width	Area%	Symmetry
1	11.171	691.1	37.8	0.3044	11.953	0.897
2	12.176	5090.9	249.3	0.3403	88.047	0.757

Chiral LC for enantioselective **2o** reaction (with **L27**)

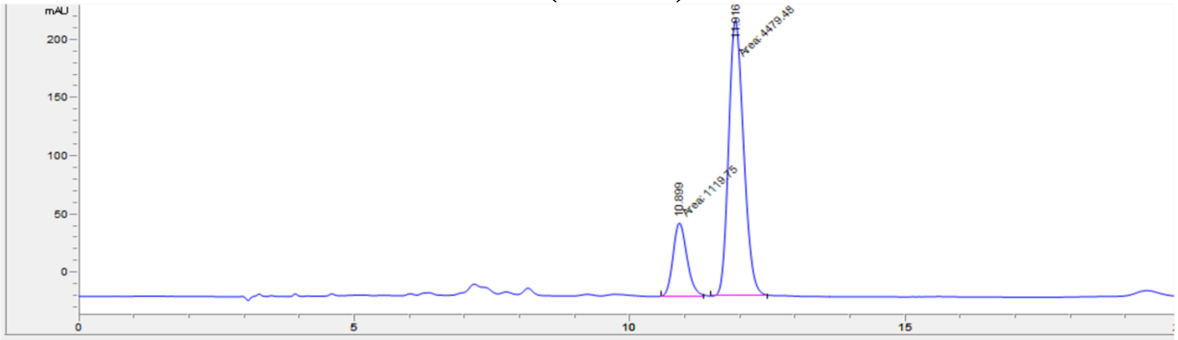

#	Time	Area	Height	Width	Area%	Symmetry
1	10.899	1119.7	63.3	0.2947	19.998	0.815
2	11.916	4479.5	237.1	0.3149	80.002	0.787

GCMS data, **2o**

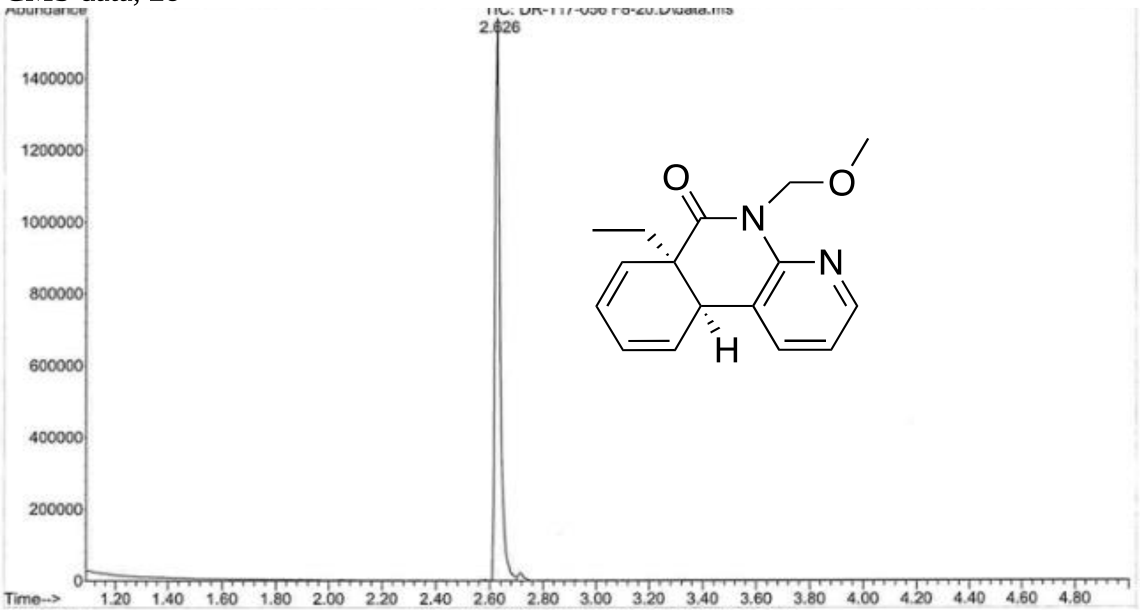

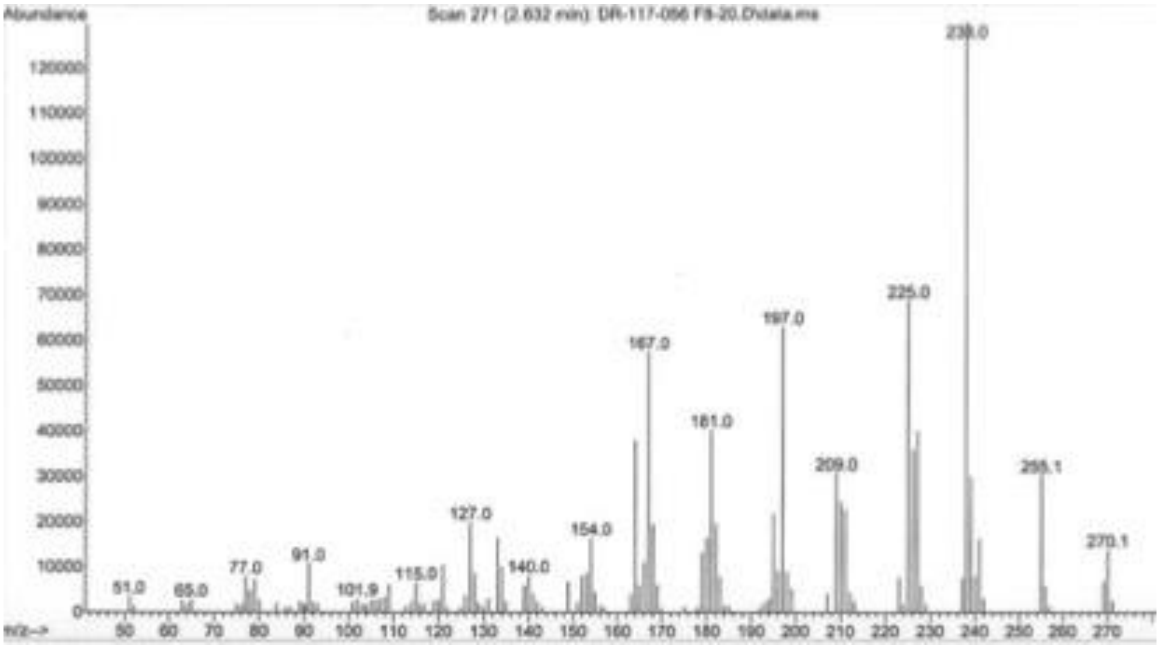

^{1}H NMR (400 MHz), **2o**

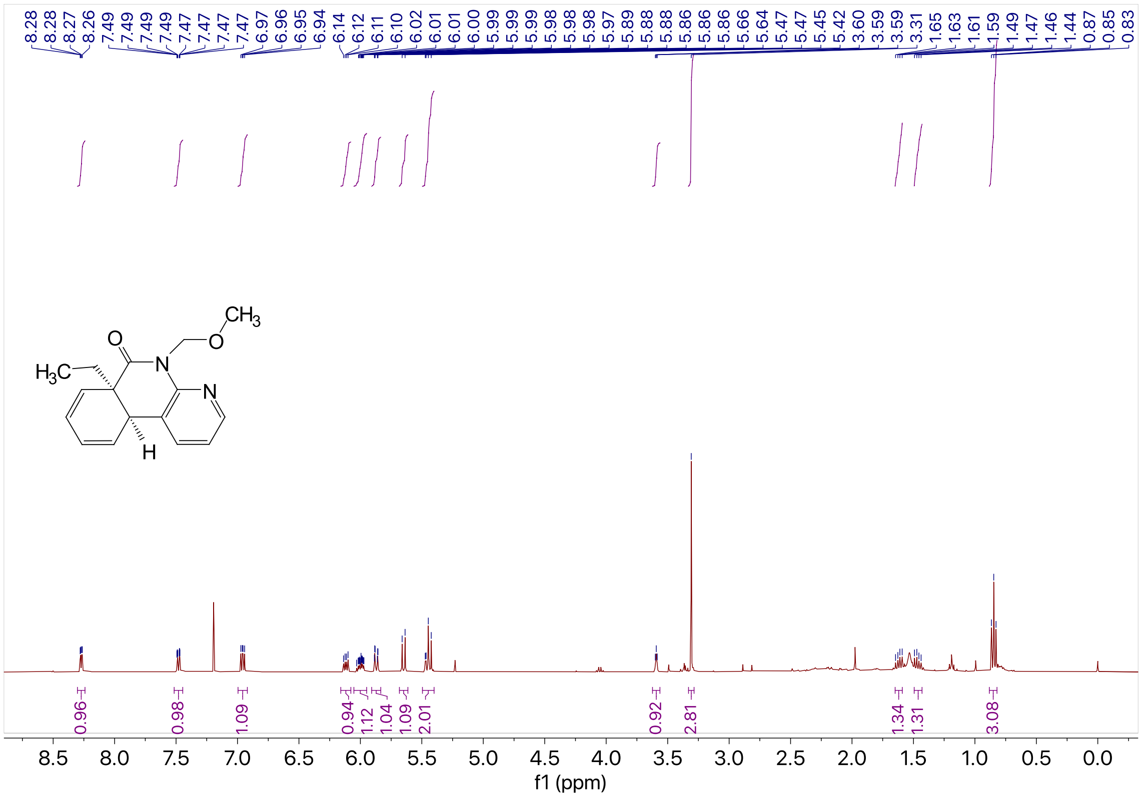

$^{13}C\{^{1}H\}$ *NMR (101 MHz, $CDCl_3$),* **2o**

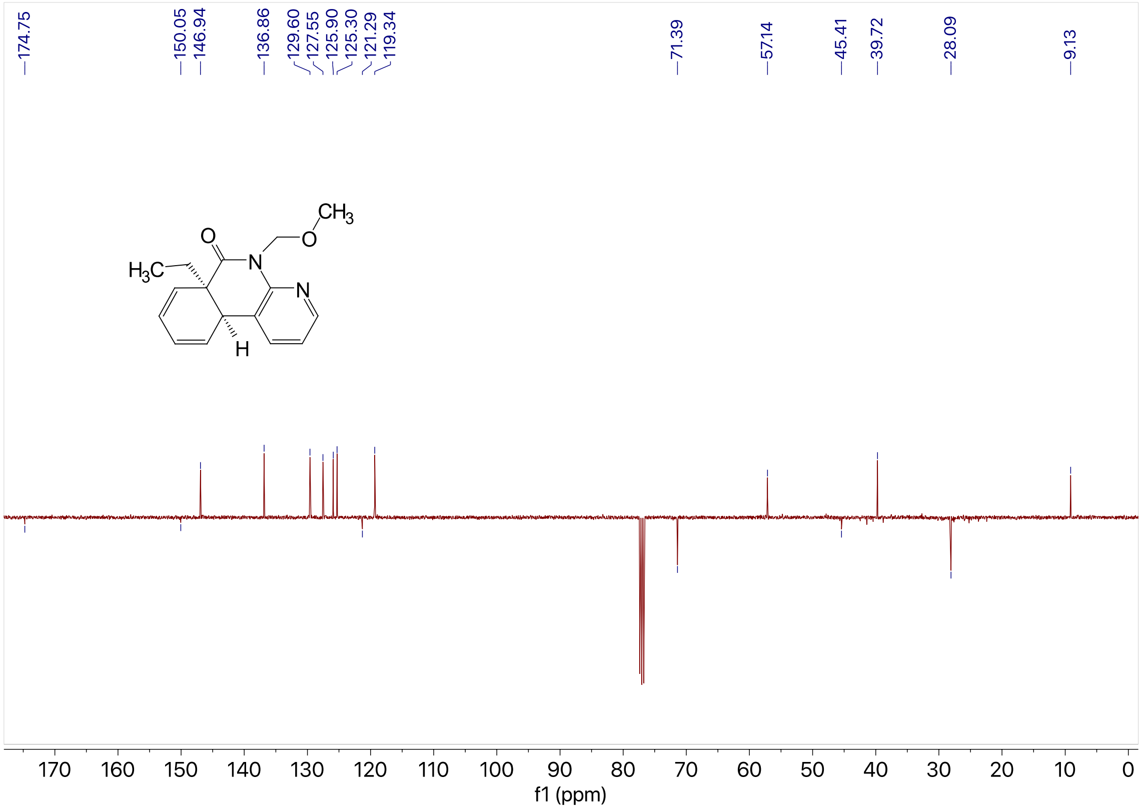

Ethyl 4b,9-dihydro-8a*H*-fluorene-8a-carboxylate (2p).

^{1}H *NMR (400 MHz),* **2p**

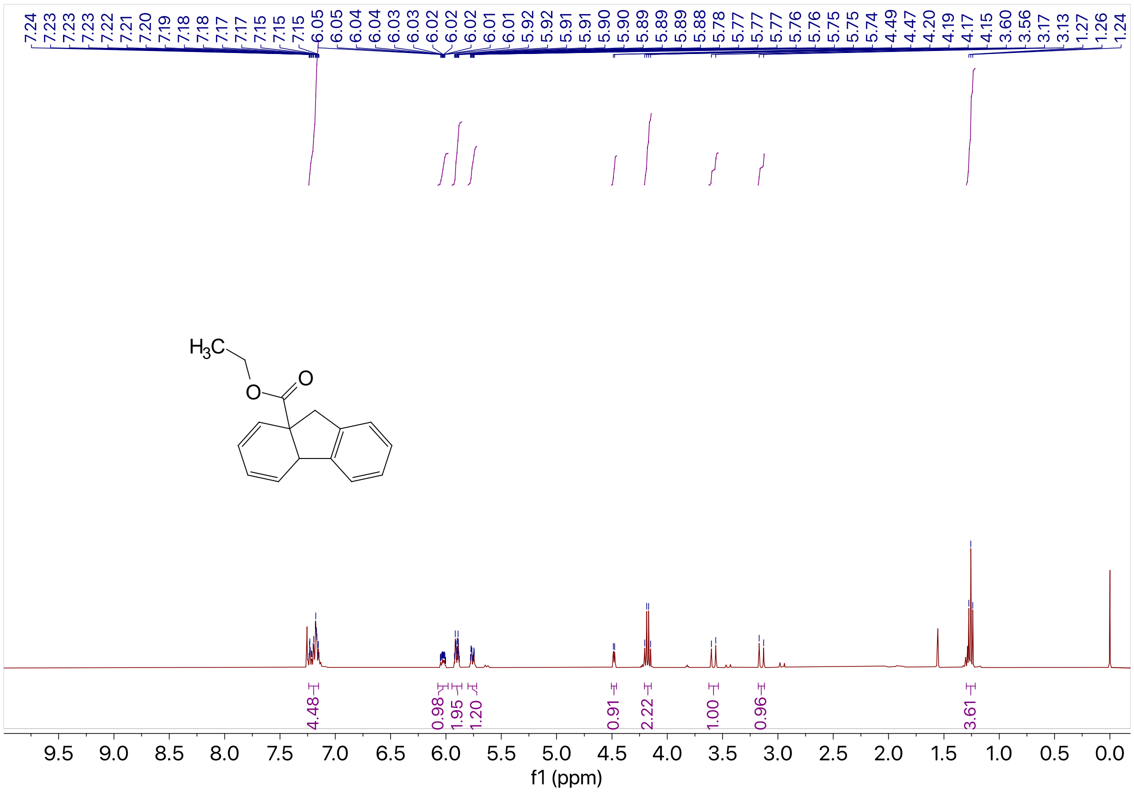

$^{13}C\{^{1}H\}$ NMR (101 MHz, $CDCl_3$), **2p**

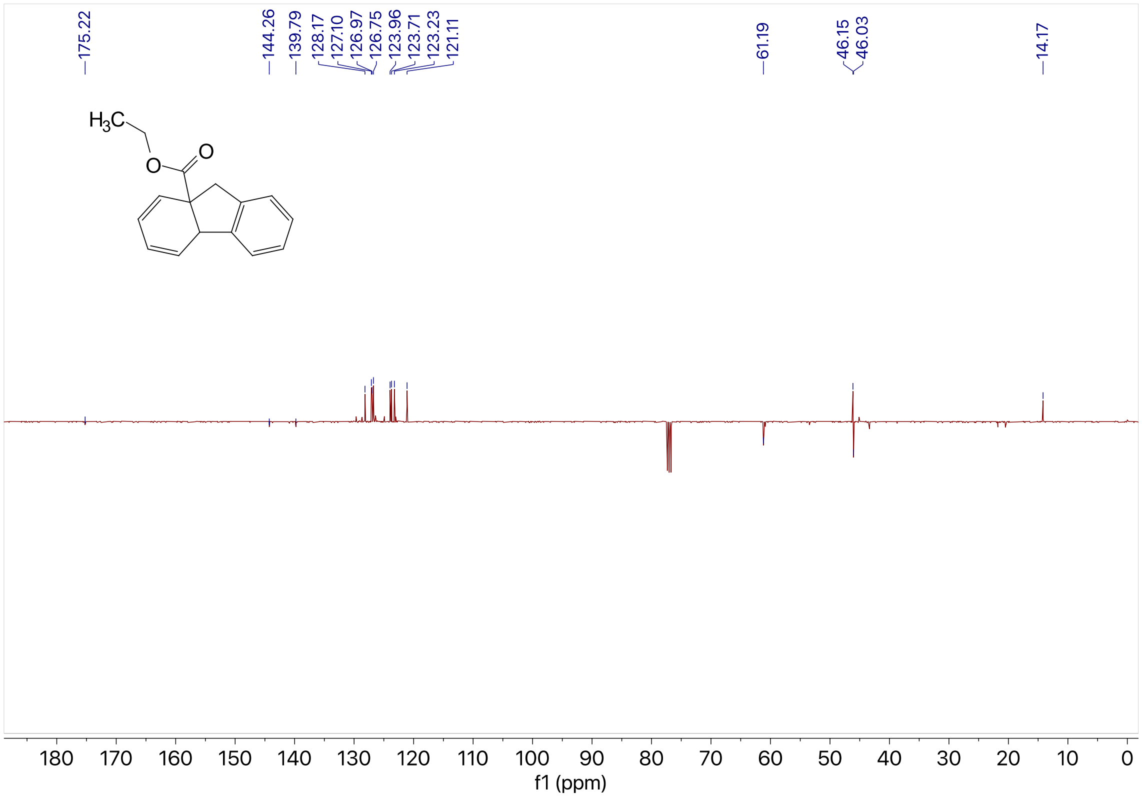

SI-Table 4: MOM Group Deprotection.

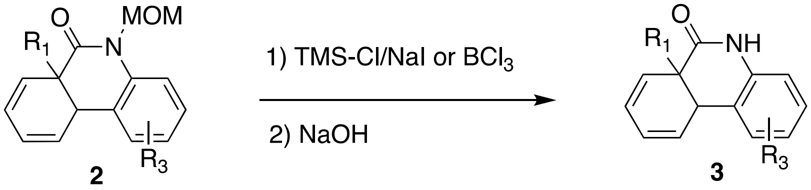

entry	R_1	R_3	yield (%)	compd
1	Et, **2c**	H	82	**3c**
2	*i*-Pr, **2e**	H	81	**3e**
3	MOM, **2f**	H	97	**3f**
4	*i*-Pr, **2i**	2-F	52	**3i**
5	*i*-Pr, **2j**	2-Cl	62	**3j**
6	*i*-Pr, **2k**	2-Me	85	**3k**
7	Me, **2m-2**	C_1=N	63	**3m-2**
8	Et, **2o**	C_4=N	79	**3o**

NMR spectra for MOM group deprotection products

(6aR,10aR)-6a-Ethyl-6a,10a-dihydrophenanthridin-6(5H)-one (3c).

^{1}H *NMR (400 MHz),* **3c**

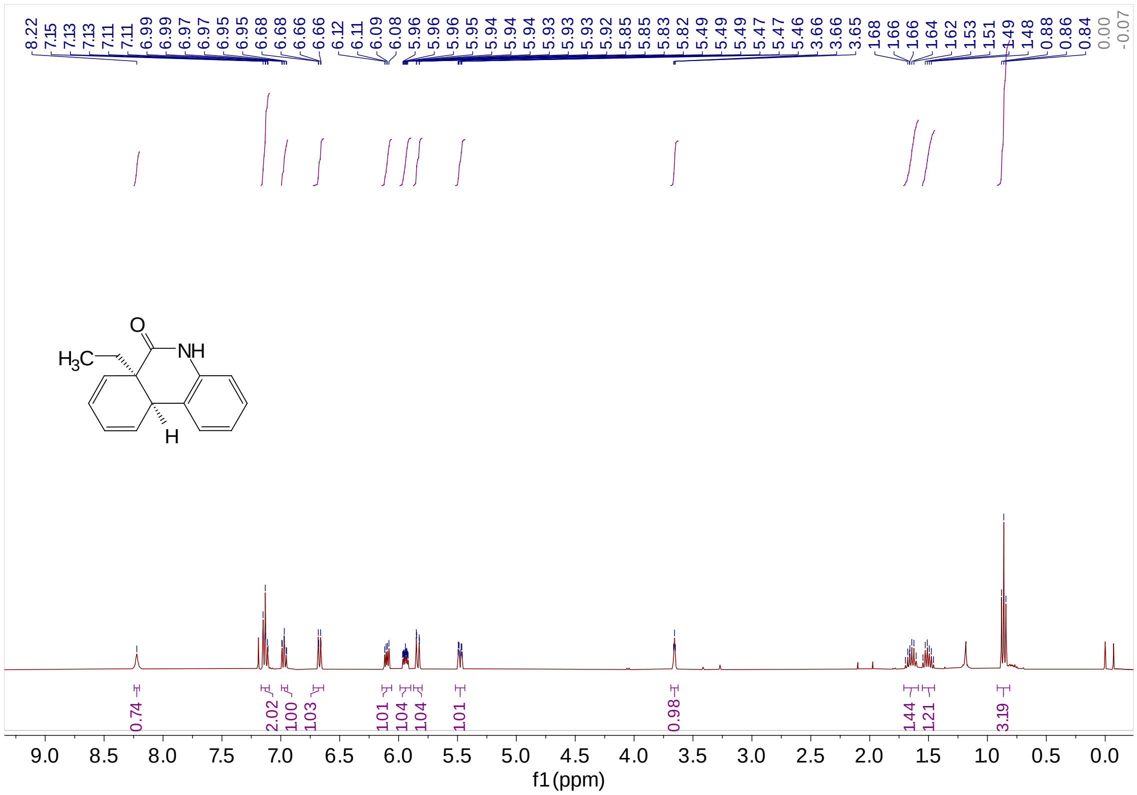

$^{13}C\{^{1}H\}$ *NMR (101 MHz, $CDCl_3$),* **3c**

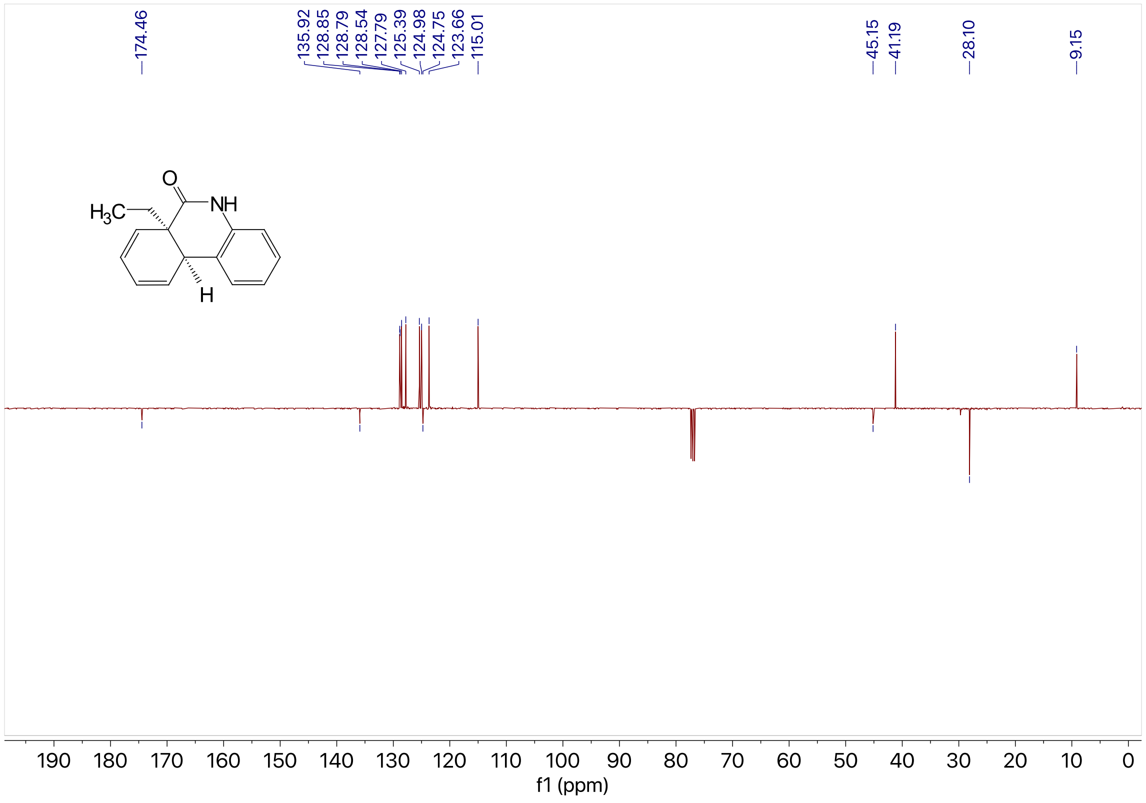

(6aR,10aR)-6a-Isopropyl-6a,10a-dihydrophenanthridin-6(5H)-one (3e).

^{1}H NMR (400 MHz), **3e**

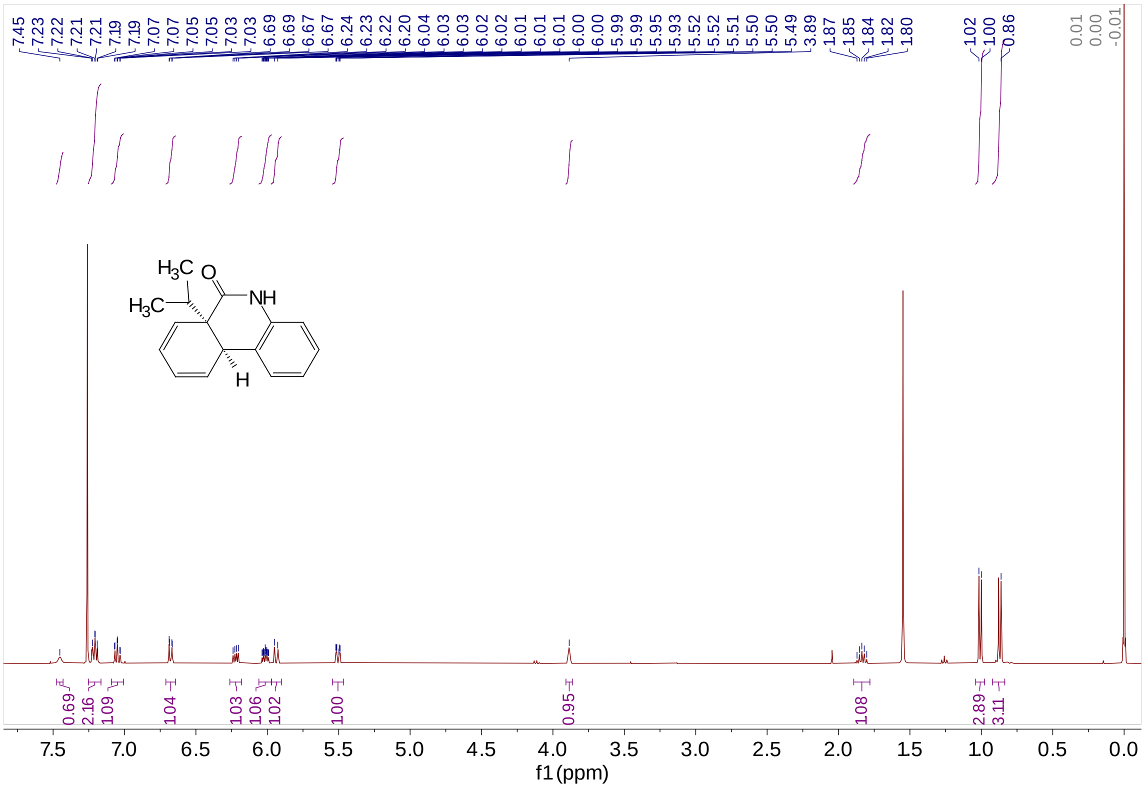

^{13}C{^{1}H} NMR (101 MHz, $CDCl_3$), **3e**

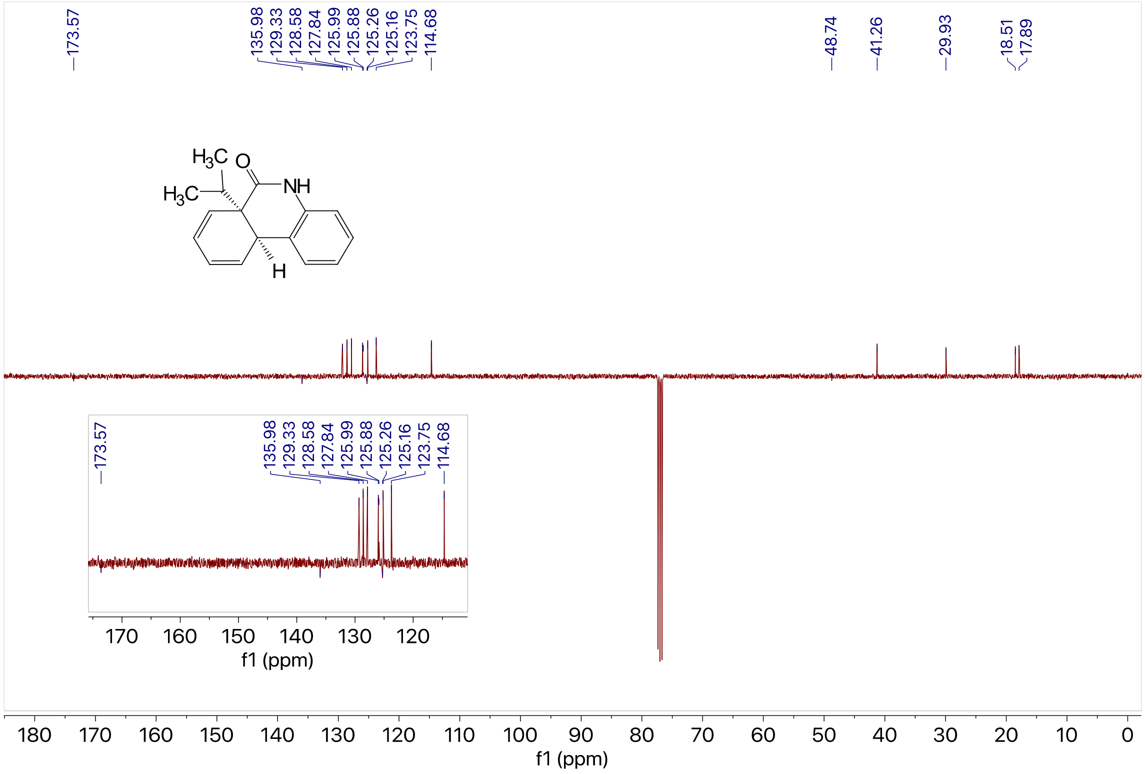

(6aR,10aR)-6a-(Methoxymethyl)-6a,10a-dihydrophenanthridin-6(5*H*)-one (3f).

^{1}H NMR (400 MHz), **3f**

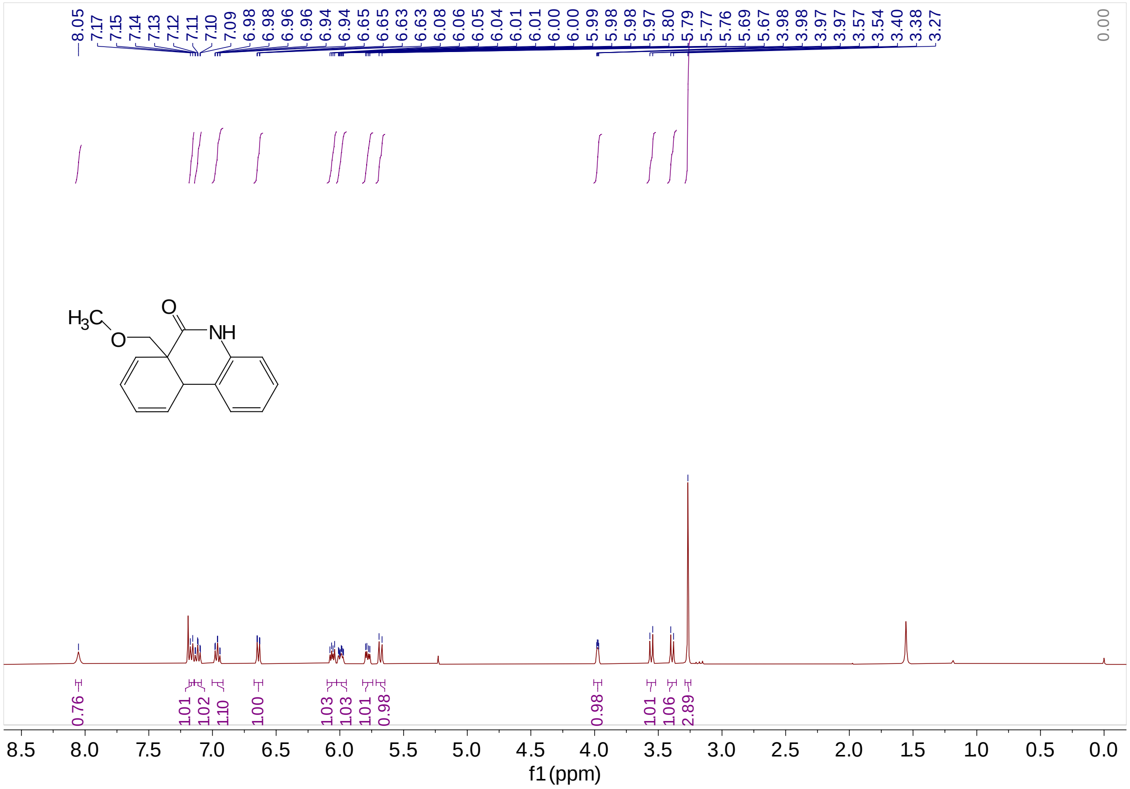

^{13}C{^{1}H} NMR (101 MHz, CDCl$_3$), **3f**

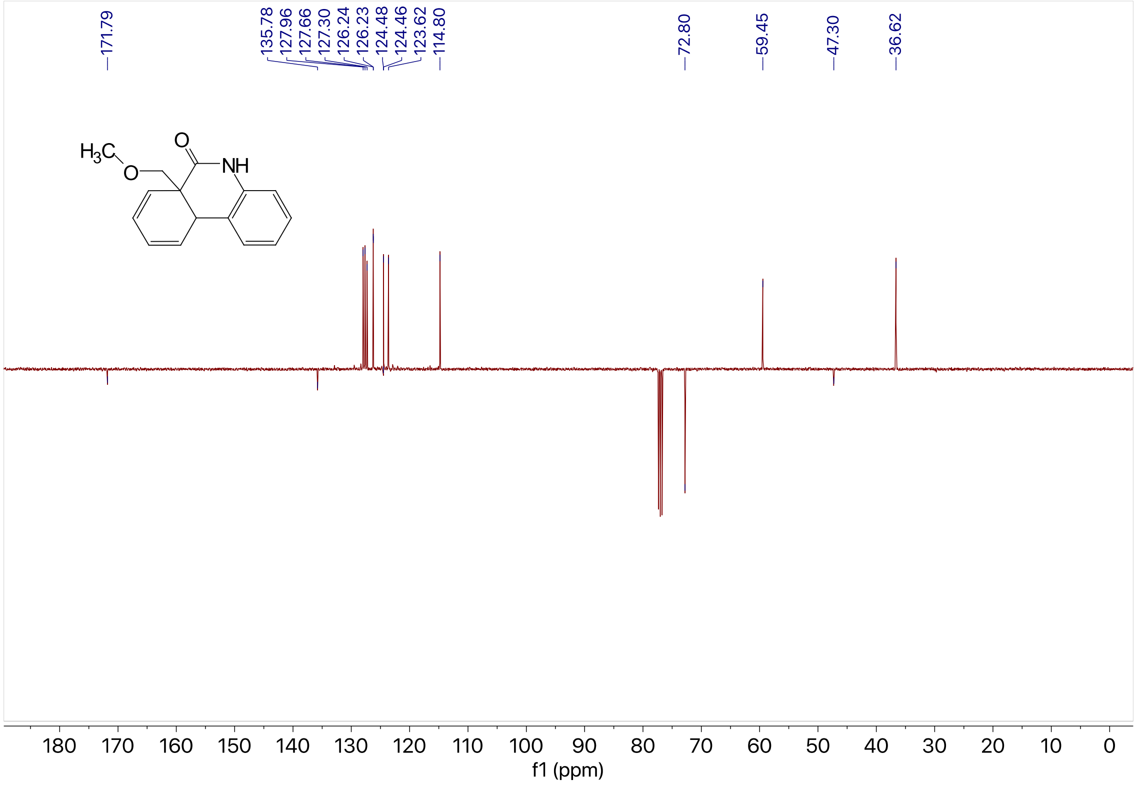

(6aR,10aR)-2-Fluoro-6a-isopropyl-6a,10a-dihydrophenanthridin-6(5H)-one (3i).

^{1}H NMR (400 MHz), **3i**

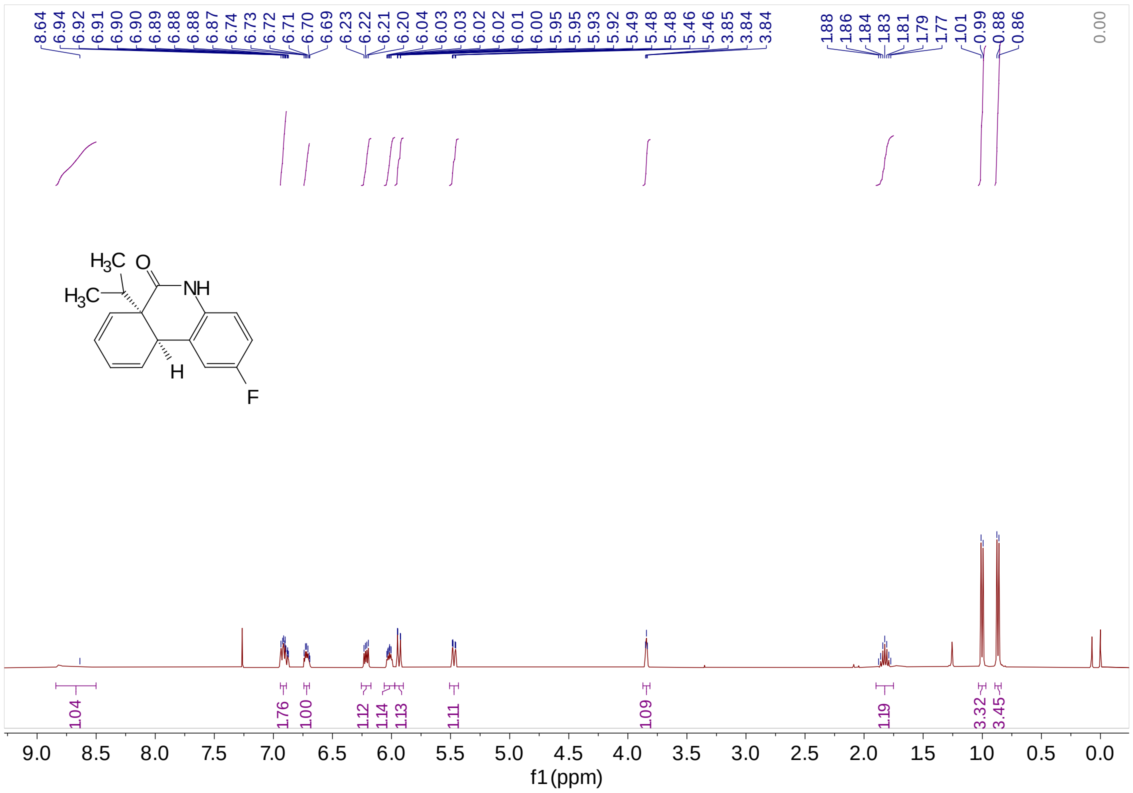

^{13}C{^{1}H} NMR (101 MHz, $CDCl_3$), **3i**

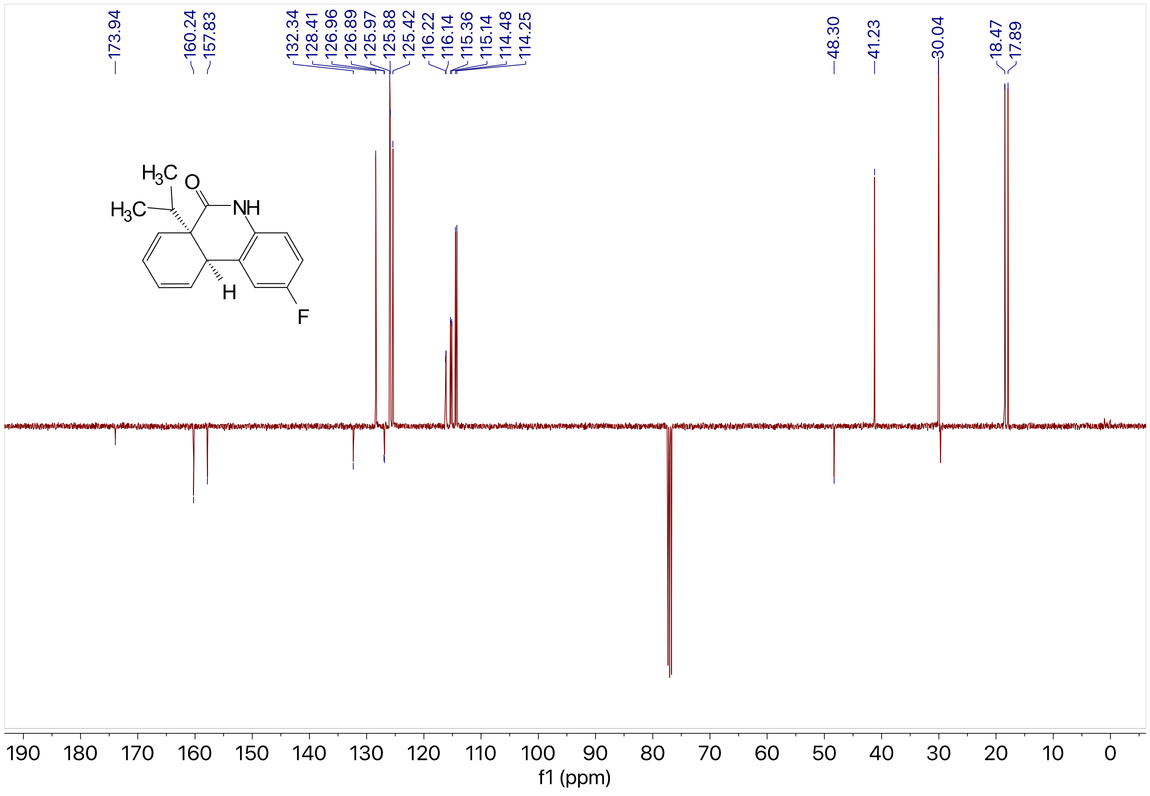

^{19}F NMR (376 MHz), **3i**

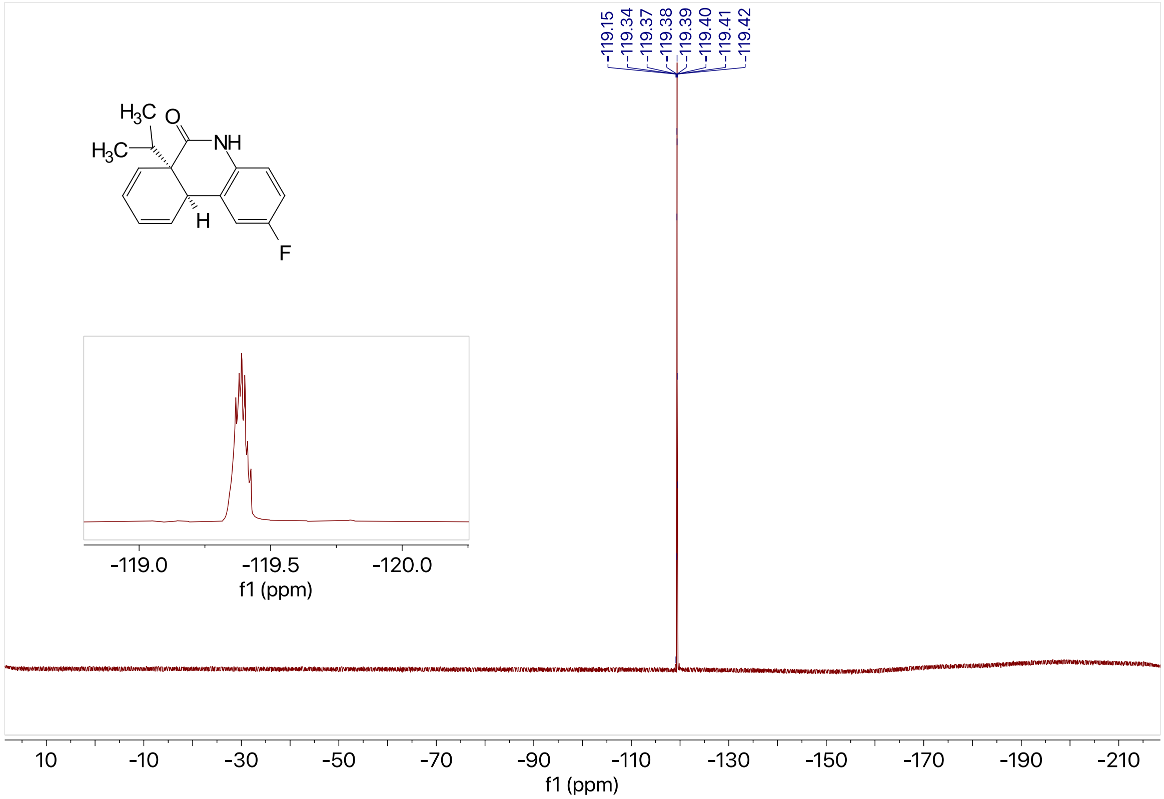

(6aR,10aR)-2-Chloro-6a-isopropyl-6a,10a-dihydrophenanthridin-6(5H)-oneone (3j).
^{1}H NMR (400 MHz), **3j**

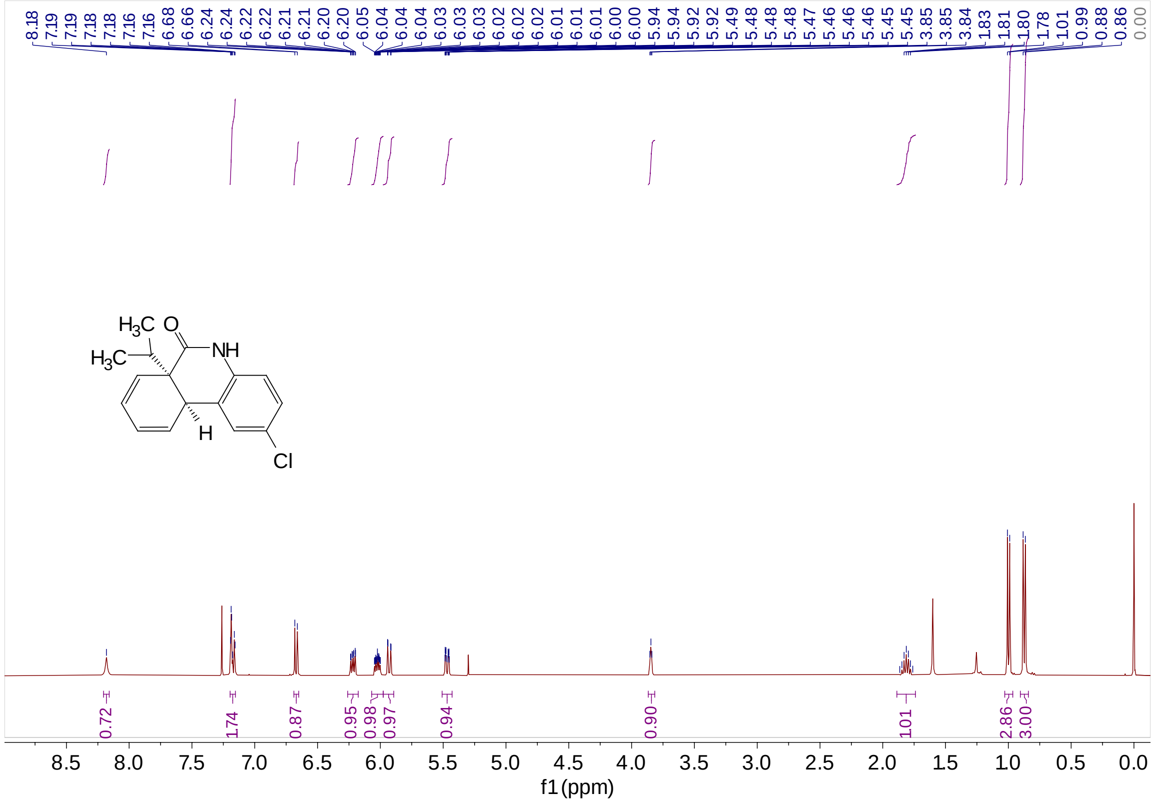

$^{13}C\{^{1}H\}$ NMR (101 MHz, $CDCl_3$), **3j**

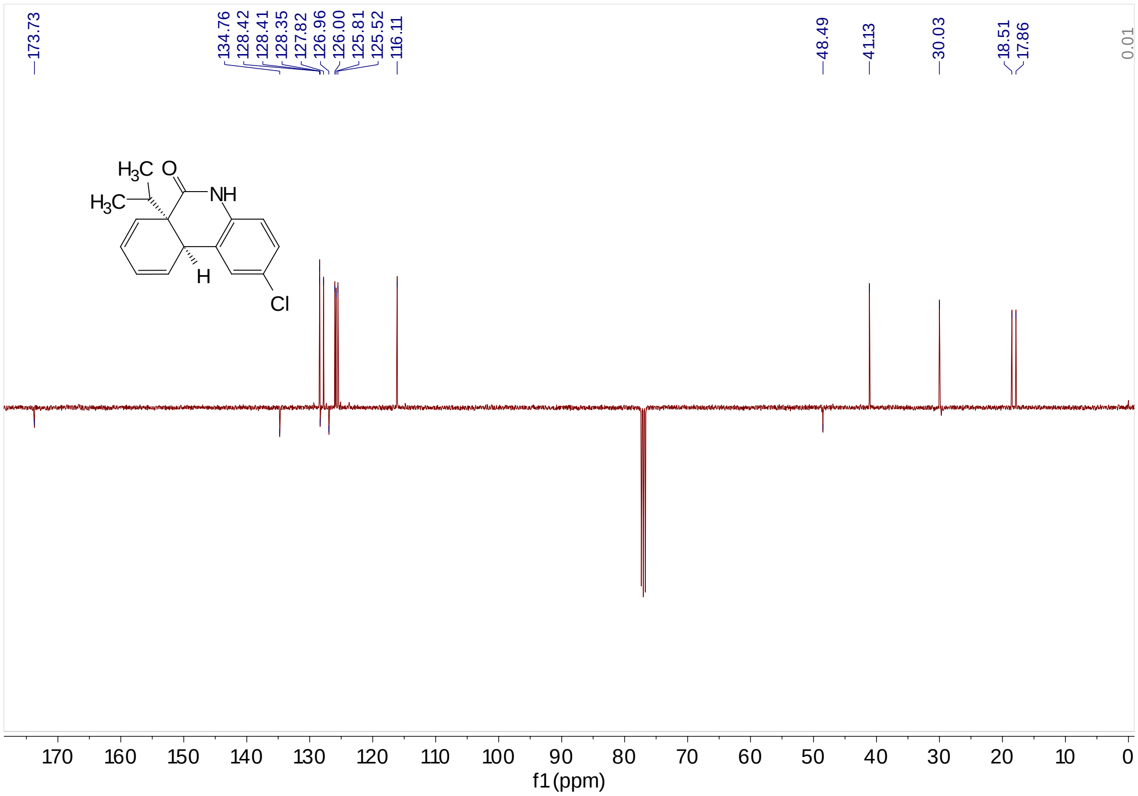

6a-Isopropyl-2-methyl-6a,10a-dihydrophenanthridin-6(5*H*)-one (3k).

^{1}H NMR (400 MHz), **3k**

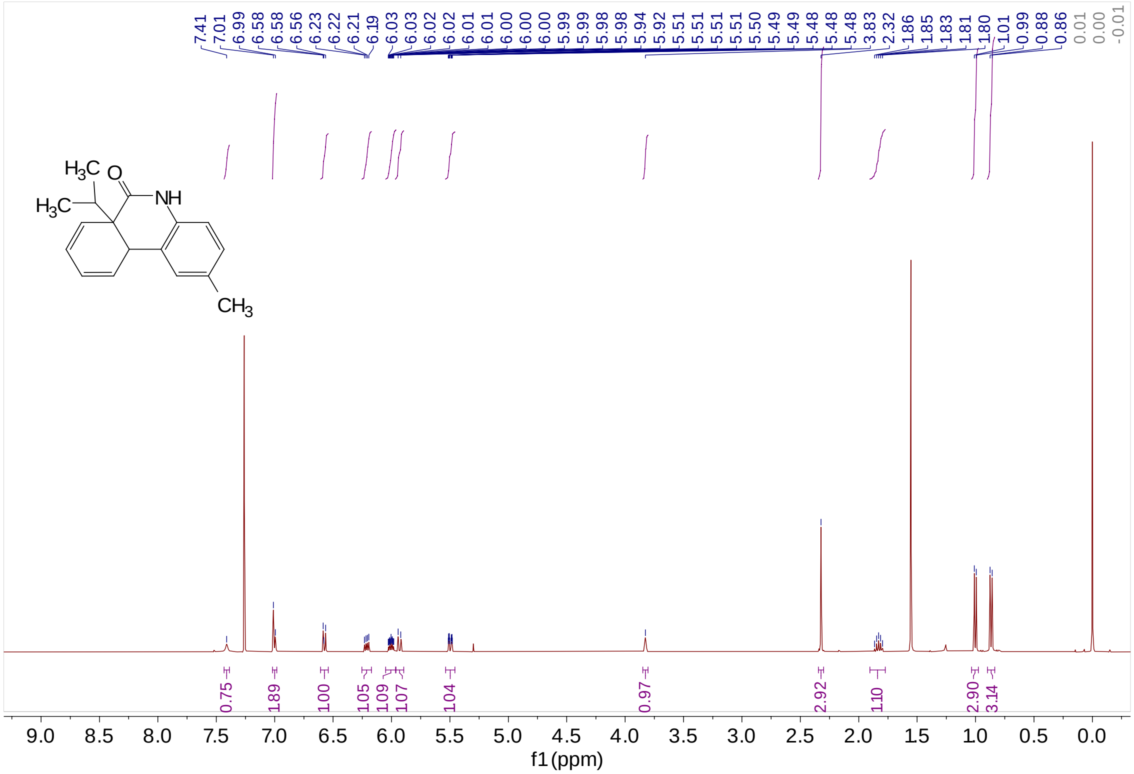

$^{13}C\{^{1}H\}$ NMR (101 MHz, $CDCl_3$), **3k**

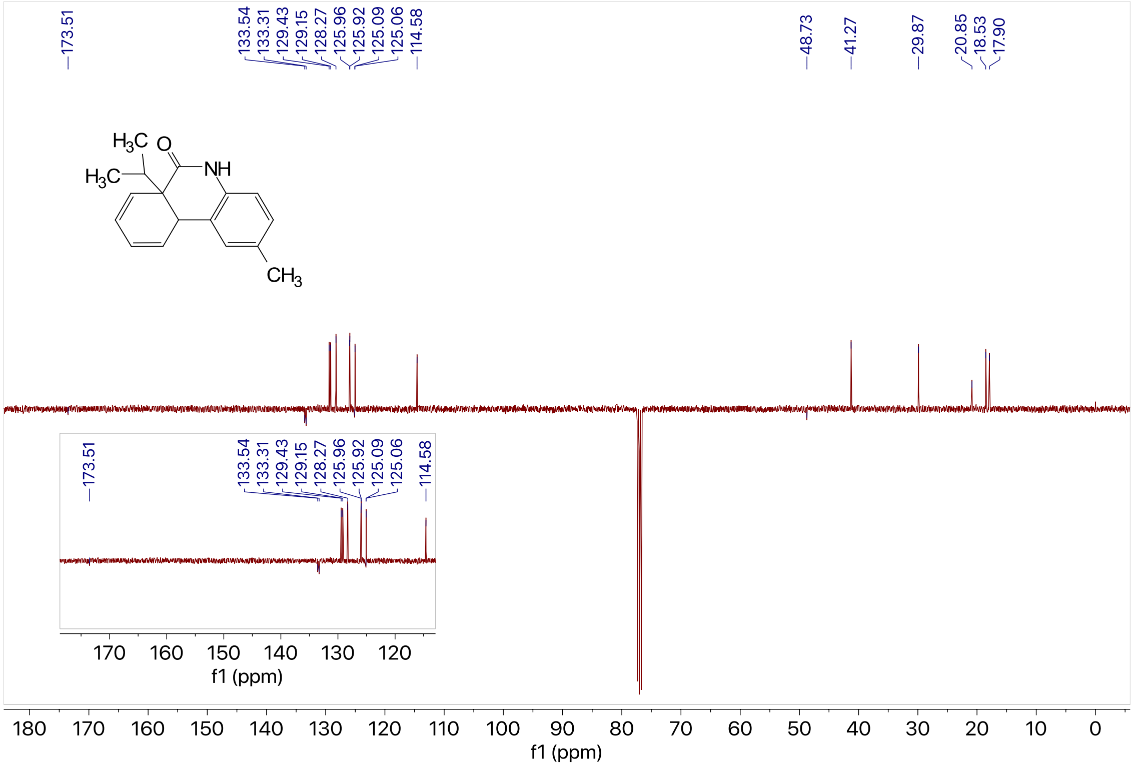

6a-Methyl-6a,9,10,10a-tetrahydrobenzo[*c*][1,5]naphthyridin-6(5*H*)-one (3m-2).

^{1}H NMR (400 MHz), **3m-2**

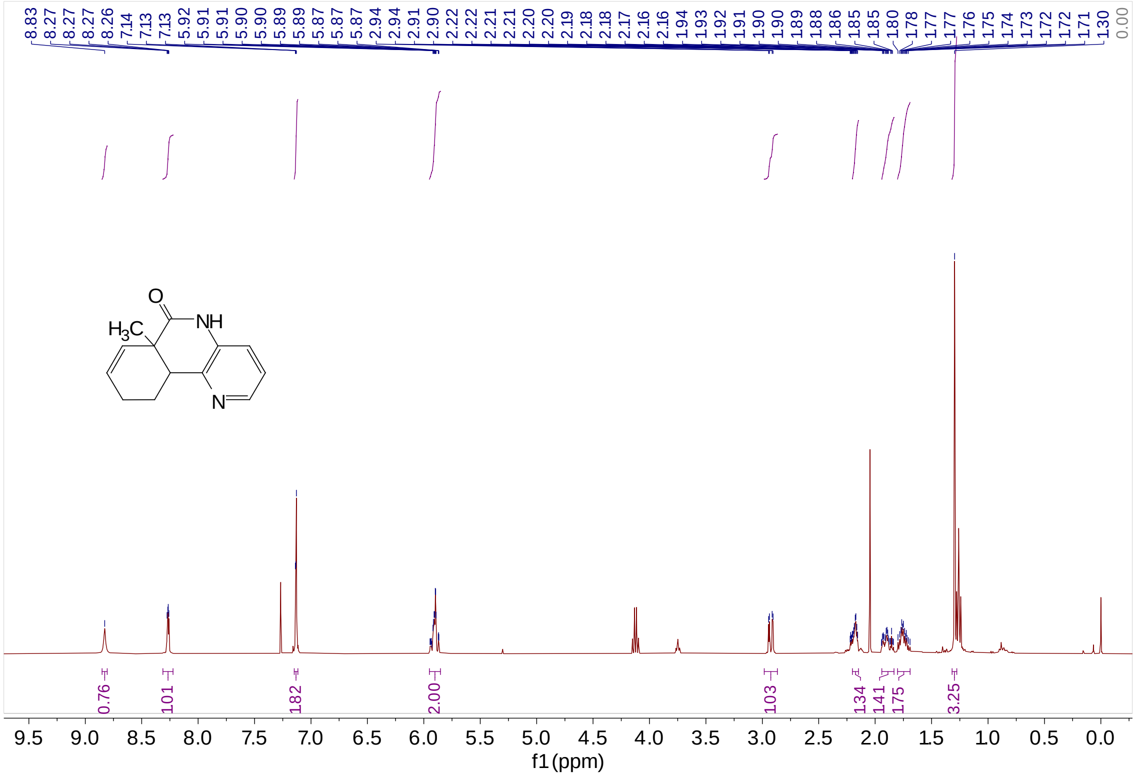

¹³C{¹H} NMR (101 MHz, CDCl₃), **3m-2**

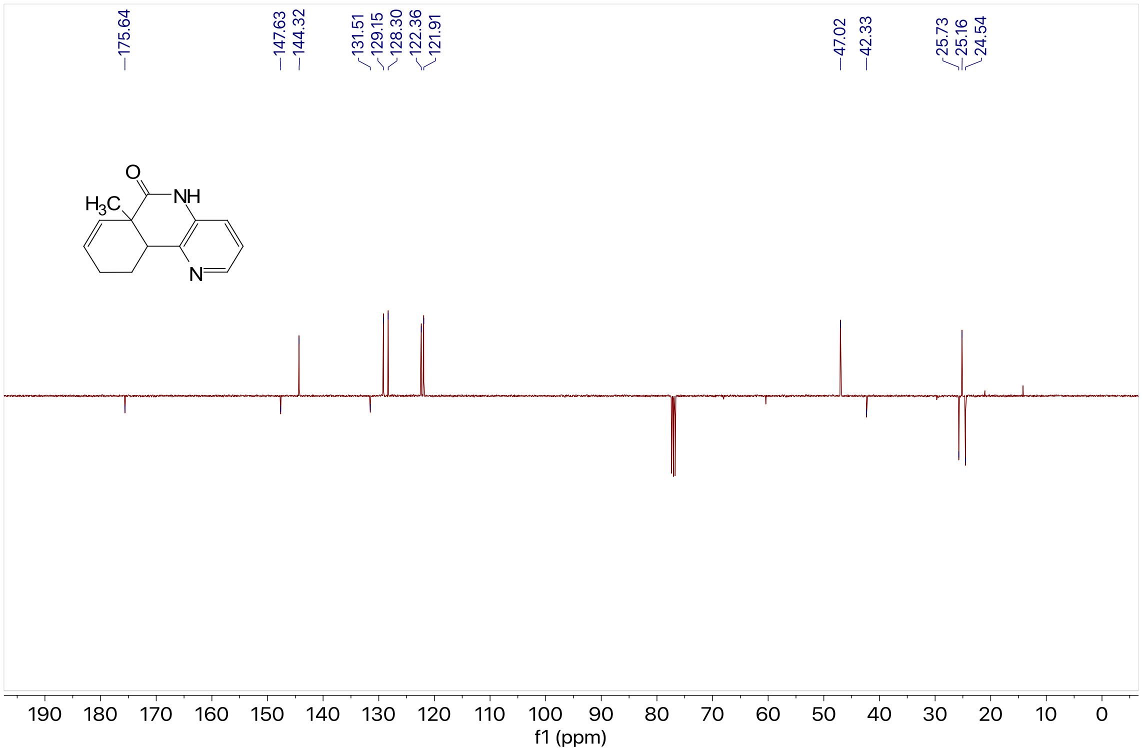

(6aR,10aR)-6a-Ethyl-6a,10a-dihydrobenzo[c][1,8]naphthyridin-6(5H)-one (3o).
¹H NMR (400 MHz), **3o**

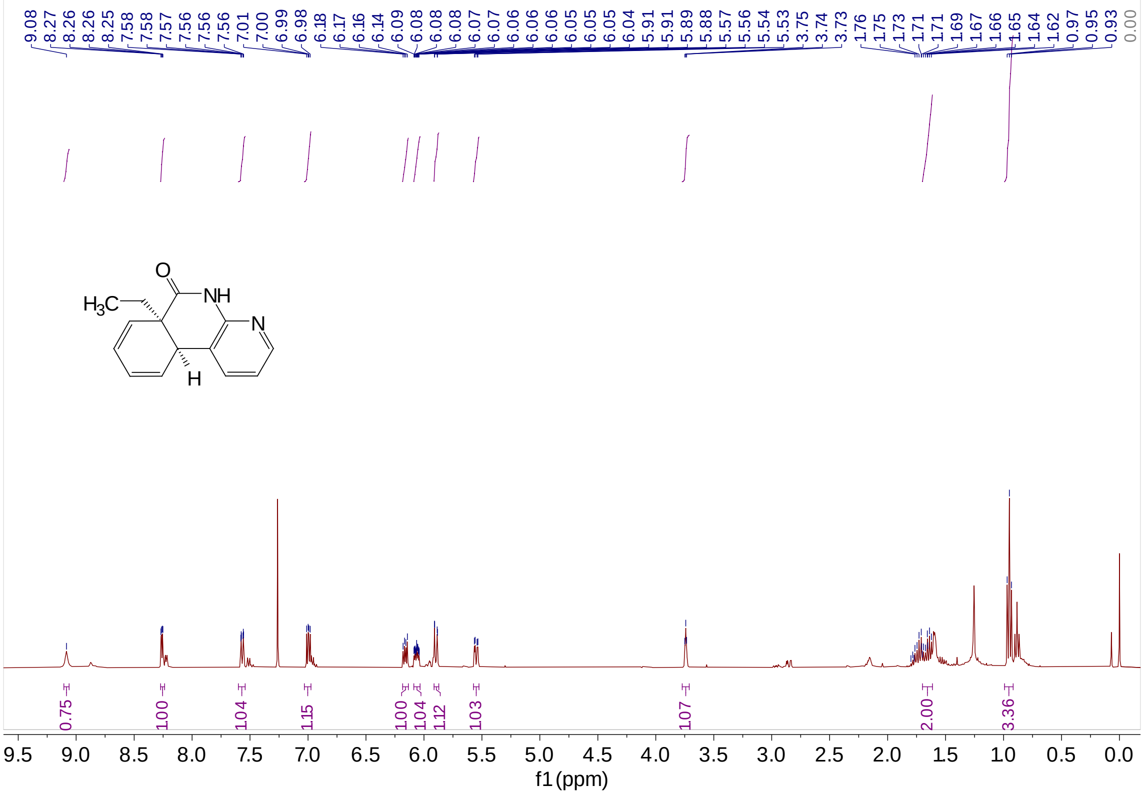

$^{13}C\{^{1}H\}$ *NMR (101 MHz, $CDCl_3$),* **3o**

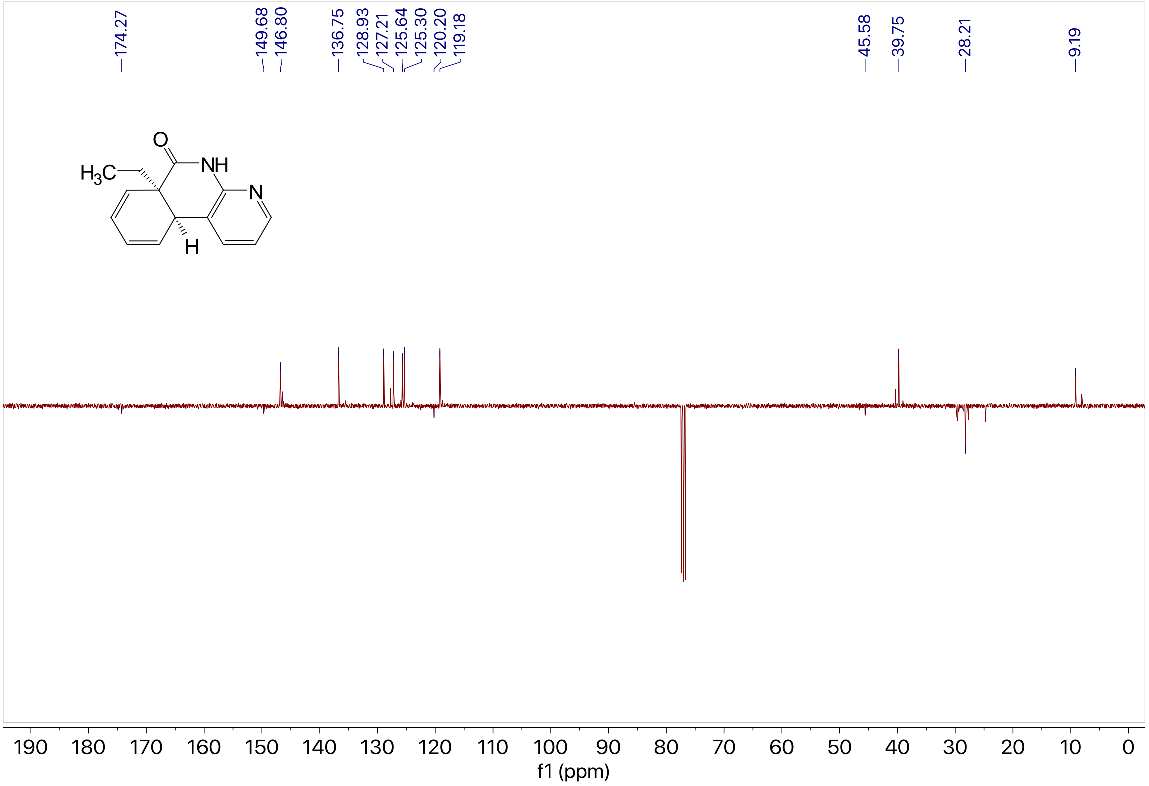

SI-Scheme 1. Synthesis of chiral *t*Bu-iQuinox ligands.

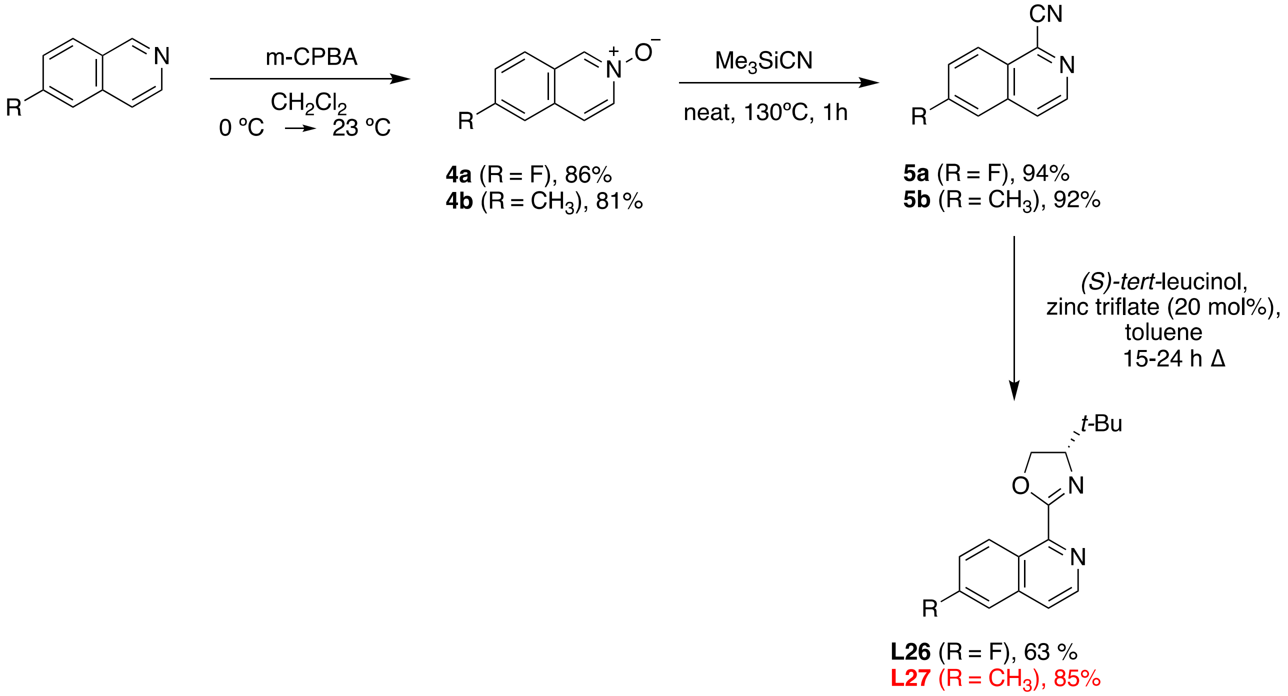

<u>**NMR spectra for isoquinoline *N*-oxides**</u>

6-fluoroisoquinoline 2-oxide (4a).

^{1}H NMR (400 MHz), **4a**

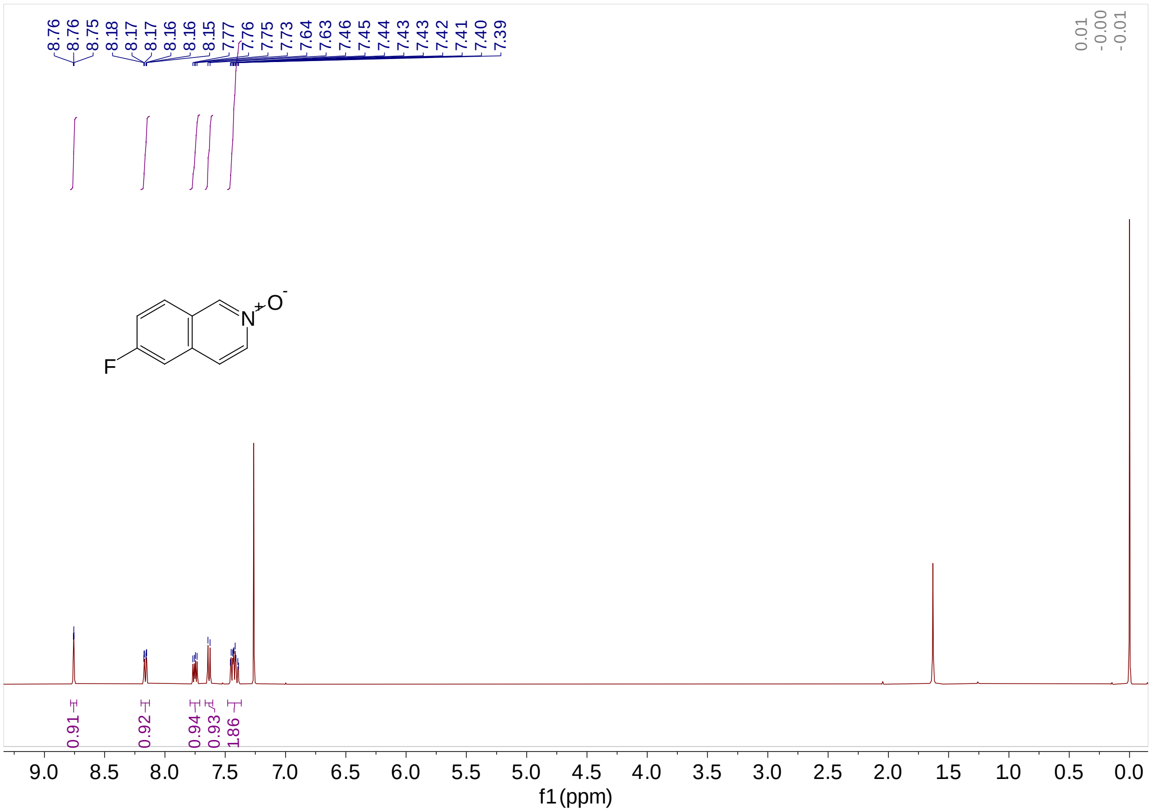

^{19}F NMR (376 MHz), **4a**

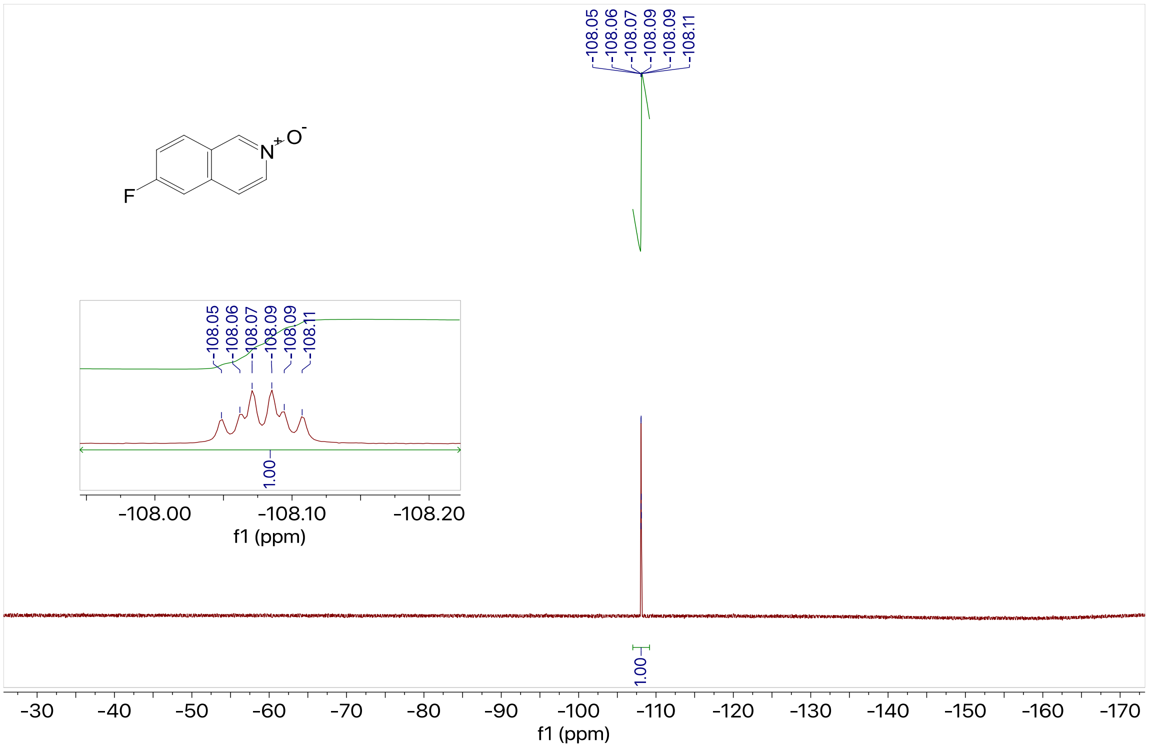

$^{13}C\{^{1}H\}$ NMR (101 MHz, $CDCl_3$), **4a**

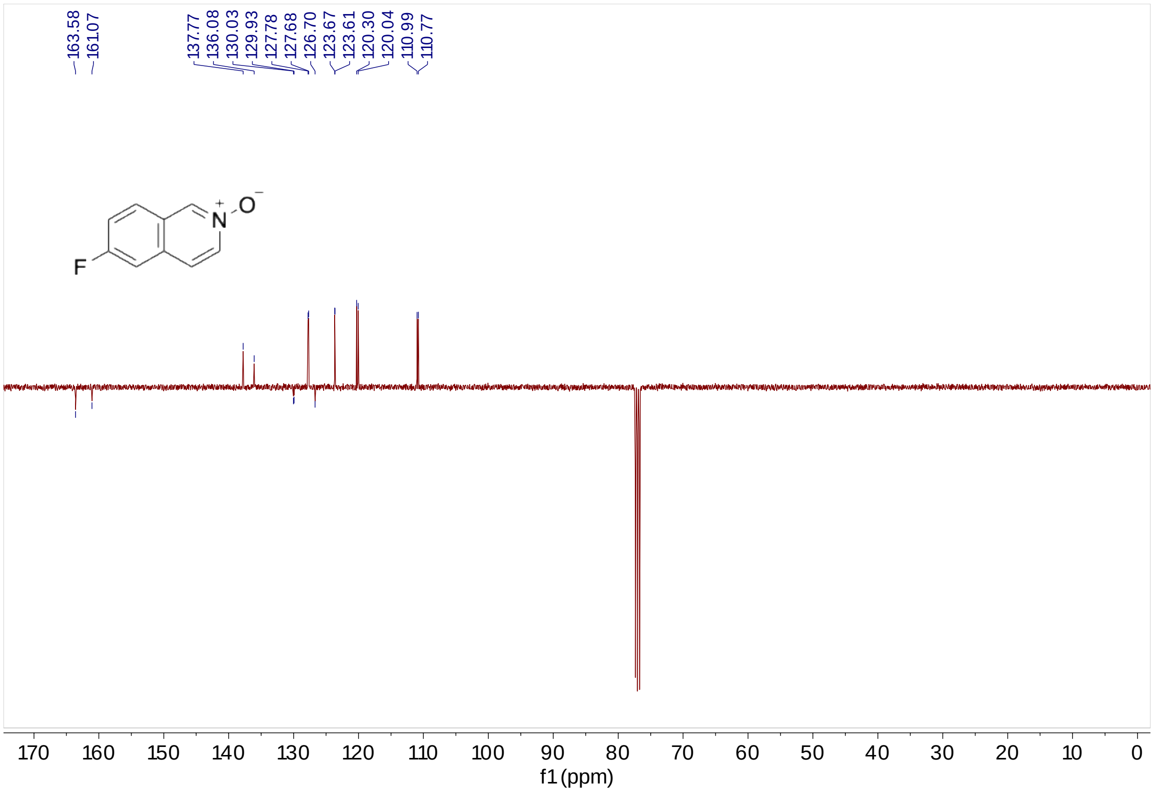

6-methylisoquinoline 2-oxide (4b).

^{1}H NMR (400 MHz), **4b**

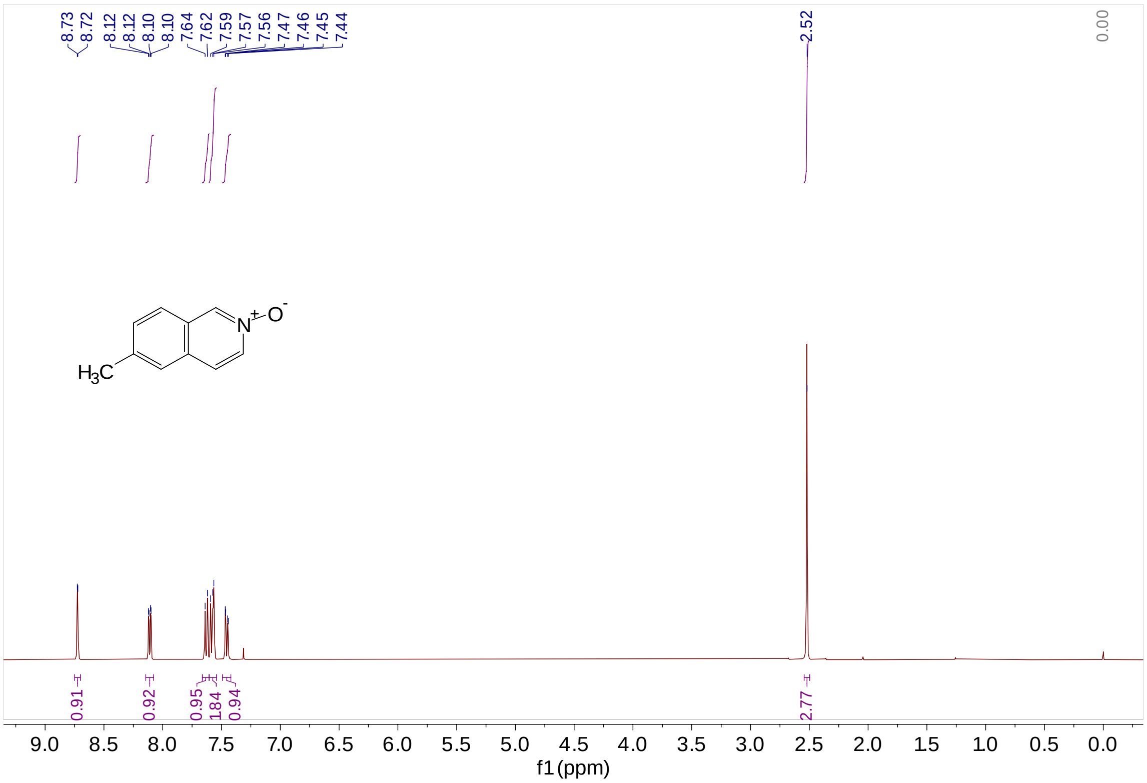

^{13}C{^{1}H} NMR (101 MHz, $CDCl_3$), **4b**

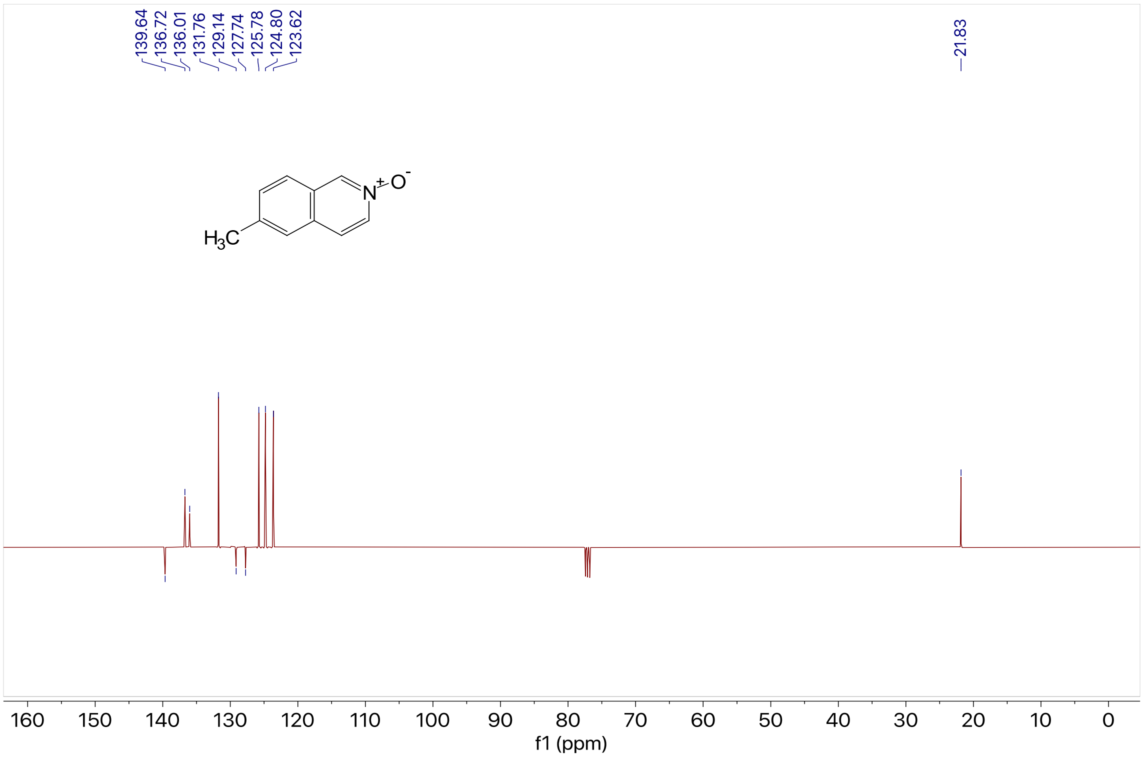

NMR spectra for isoquinoline carbonitrile products

6-fluoroisoquinoline-1-carbonitrile (5a).

^{1}H NMR (400 MHz), **5a**

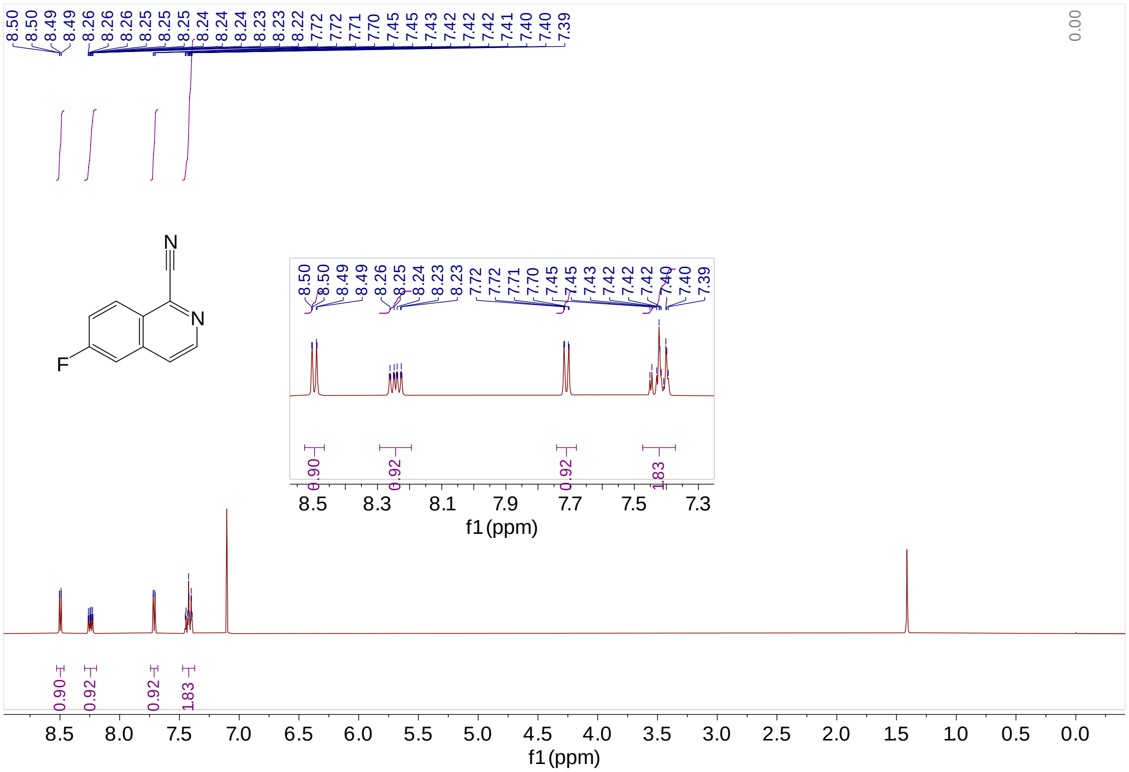

^{19}*F NMR (376 MHz)*, **5a**

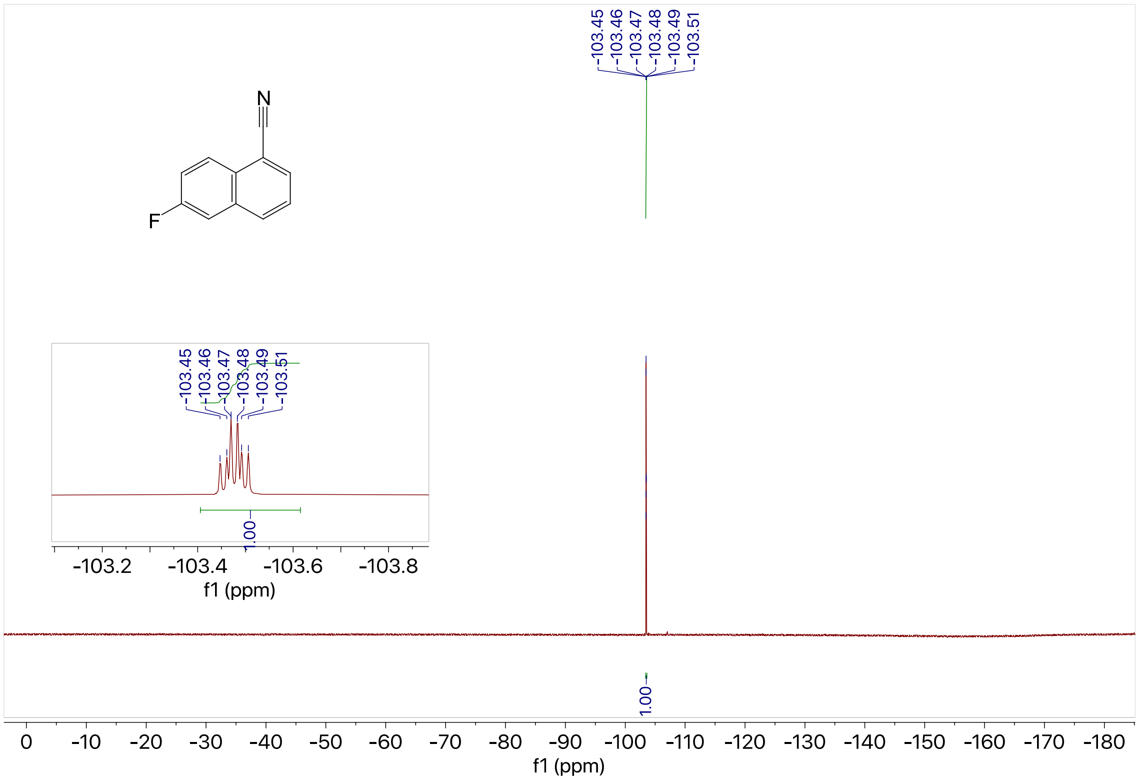

^{13}*C{^{1}H} NMR (101 MHz, CDCl$_3$)*, **5a**

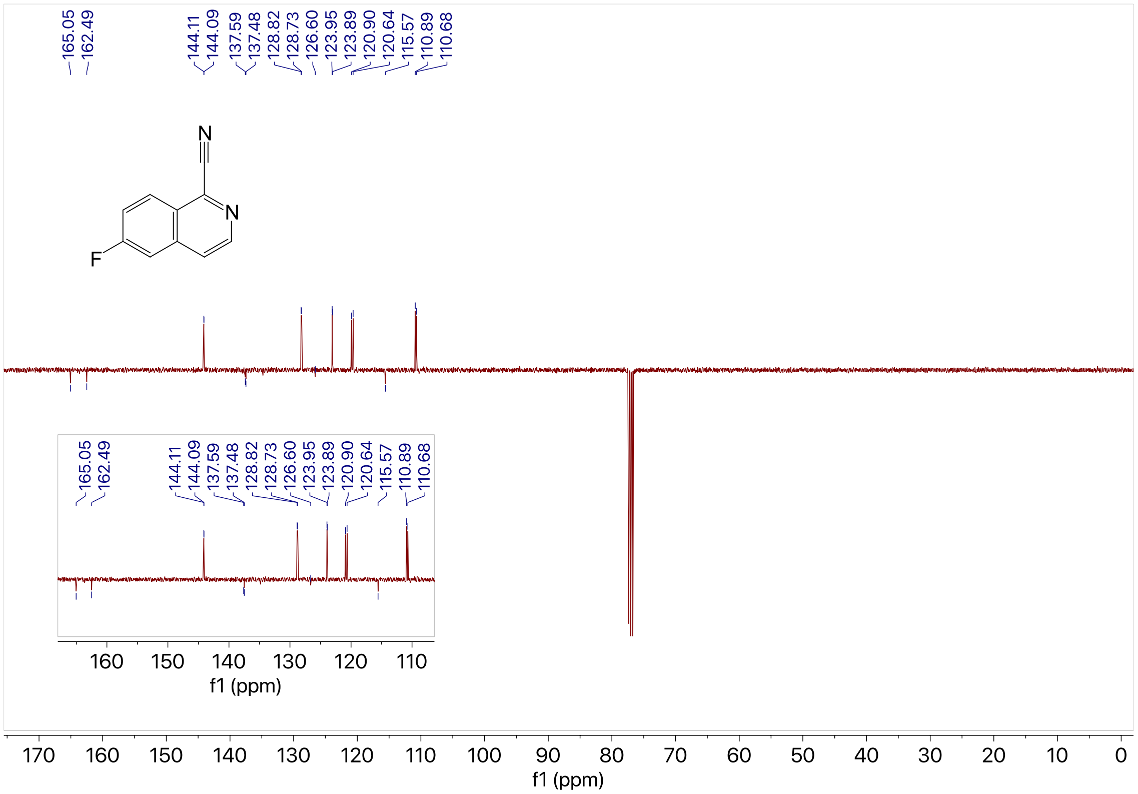

6-methylisoquinoline-1-carbonitrile (5b).

^{1}H NMR (400 MHz), **5b**

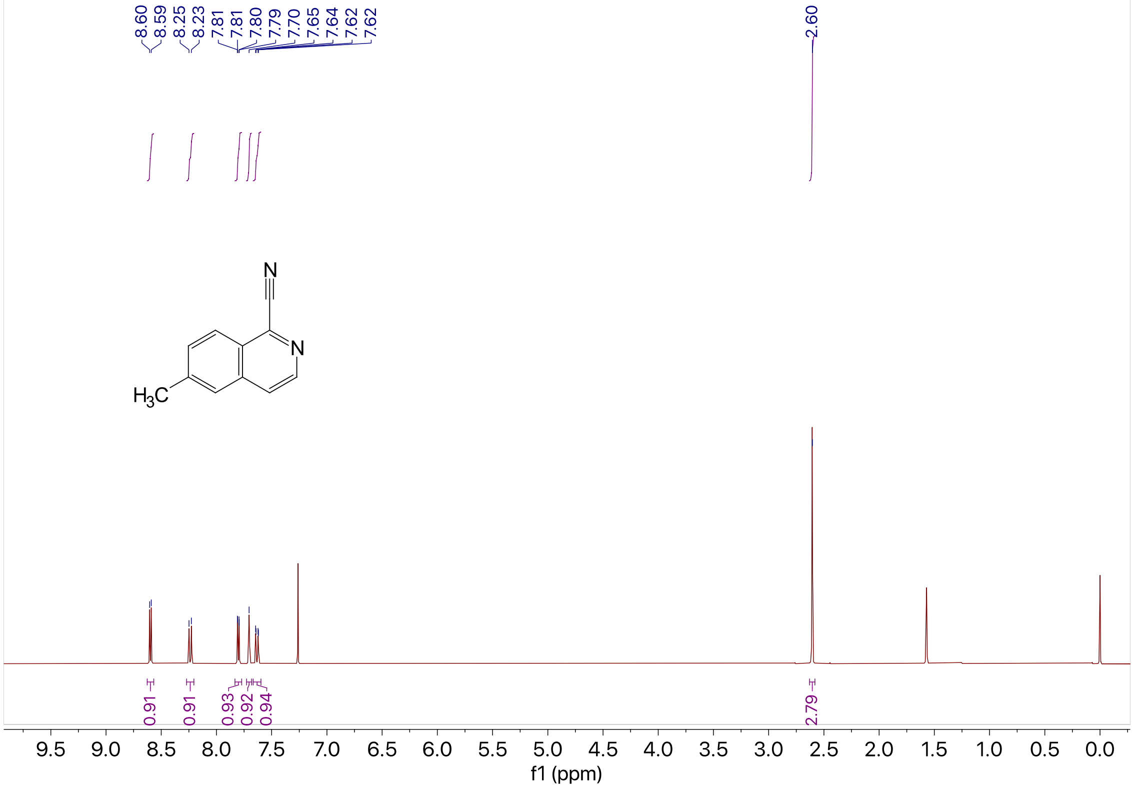

$^{13}C\{^{1}H\}$ NMR (101 MHz, $CDCl_3$), **5b**

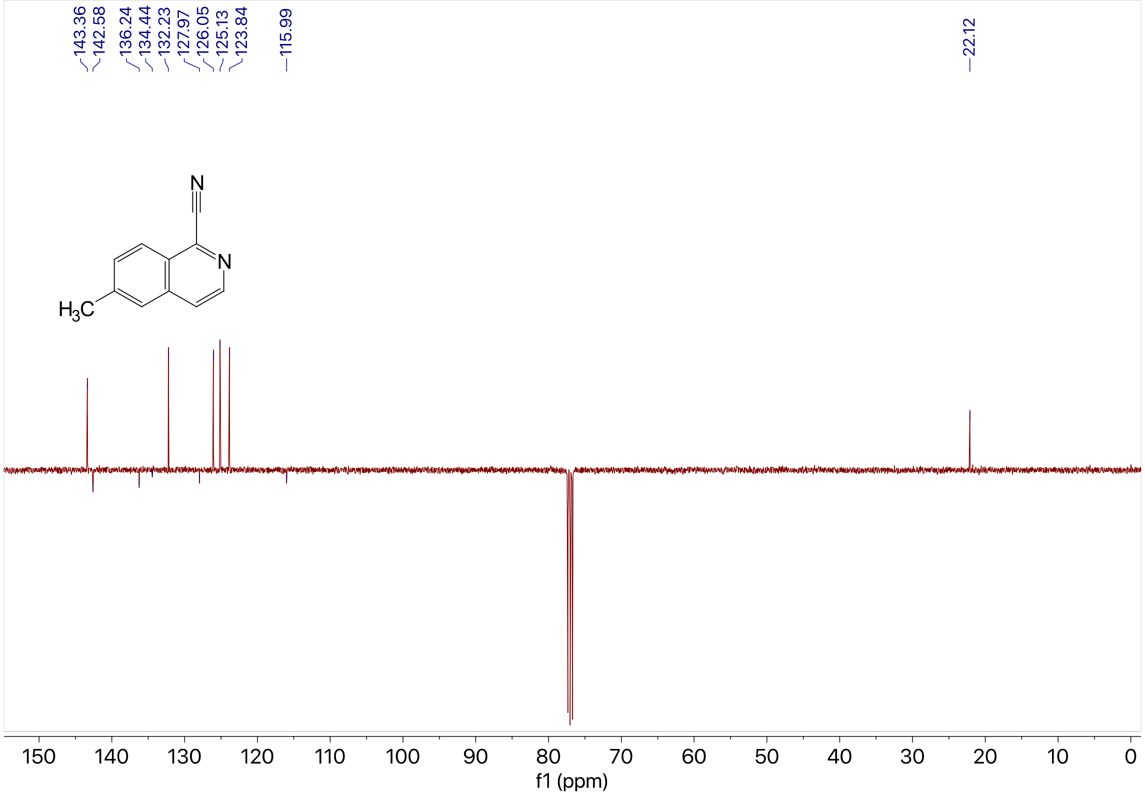

<u>NMR spectra for (*S*)-tert-Butyl *i*Quinox ligands</u>

(*S*)-4-(*tert*-Butyl)-2-(6-fluoroisoquinolin-1-yl)-4,5-dihydrooxazole (*t*Bu-^{6}F*i*Quinox, L26).

^{1}*H NMR (400 MHz)*, **L26**

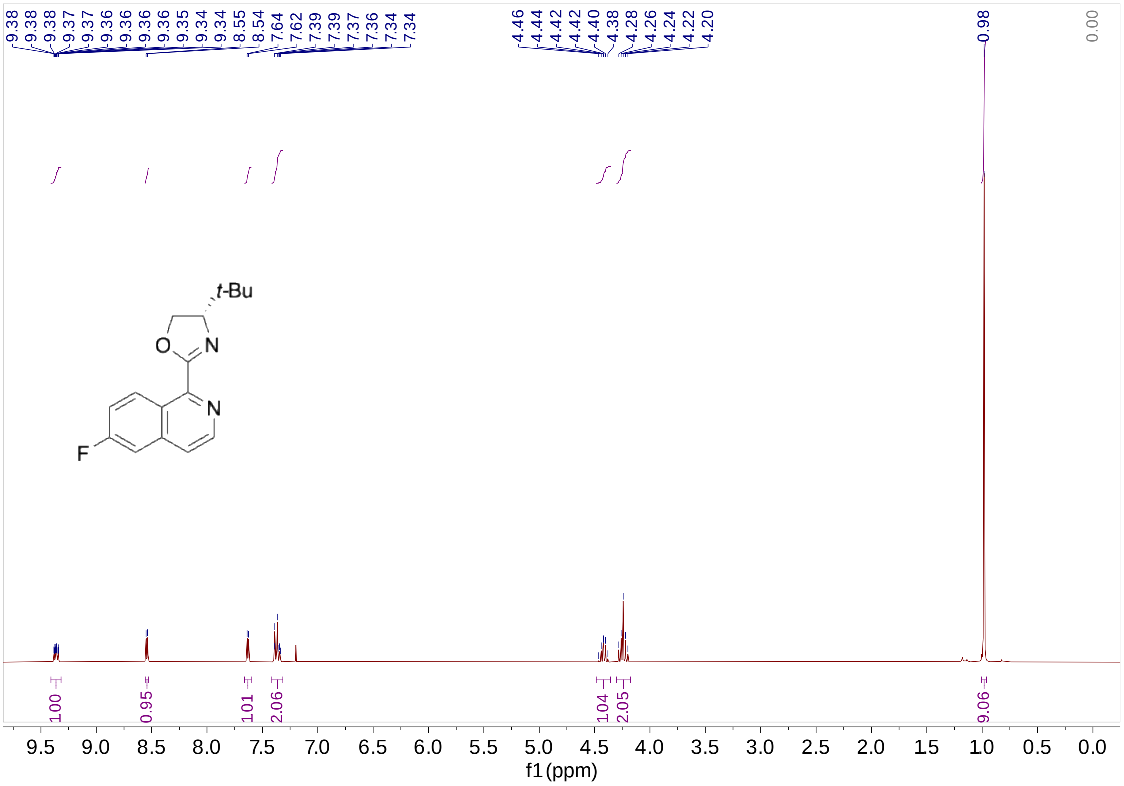

^{19}*F NMR (376 MHz)*, **L26**

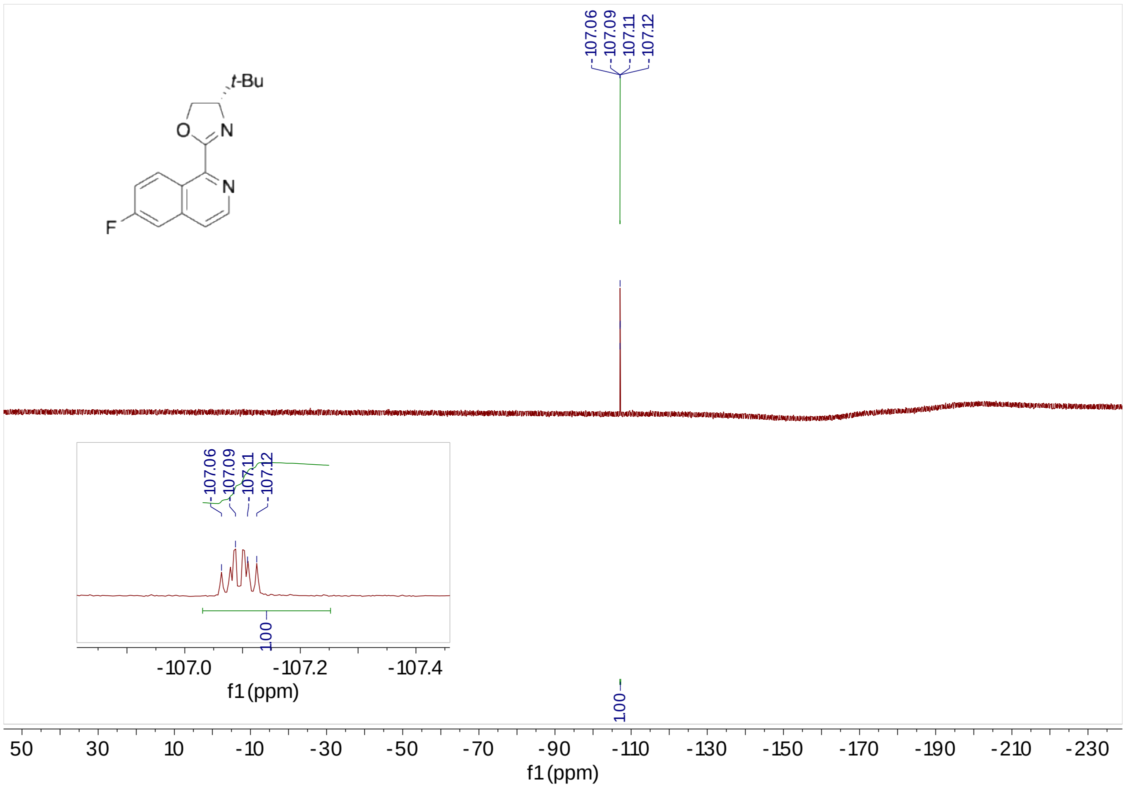

$^{13}C\{^{1}H\}$ *NMR (101 MHz, CDCl$_3$)*, **L26**

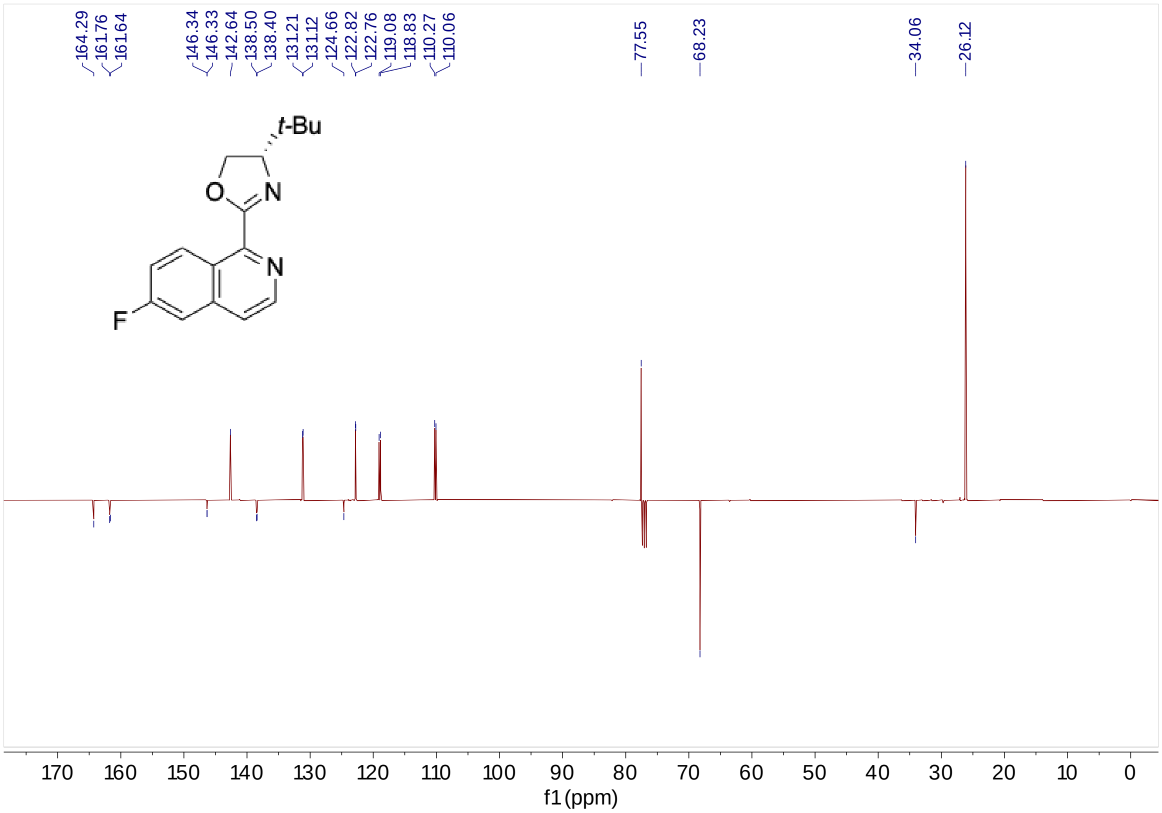

(*S*)-4-(*tert*-Butyl)-2-(6-methylisoquinolin-1-yl)-4,5-dihydrooxazole (*t*Bu-^{6}CH$_3$*i*Quinox, L27).

^{1}H *NMR (400 MHz)*, **L27**

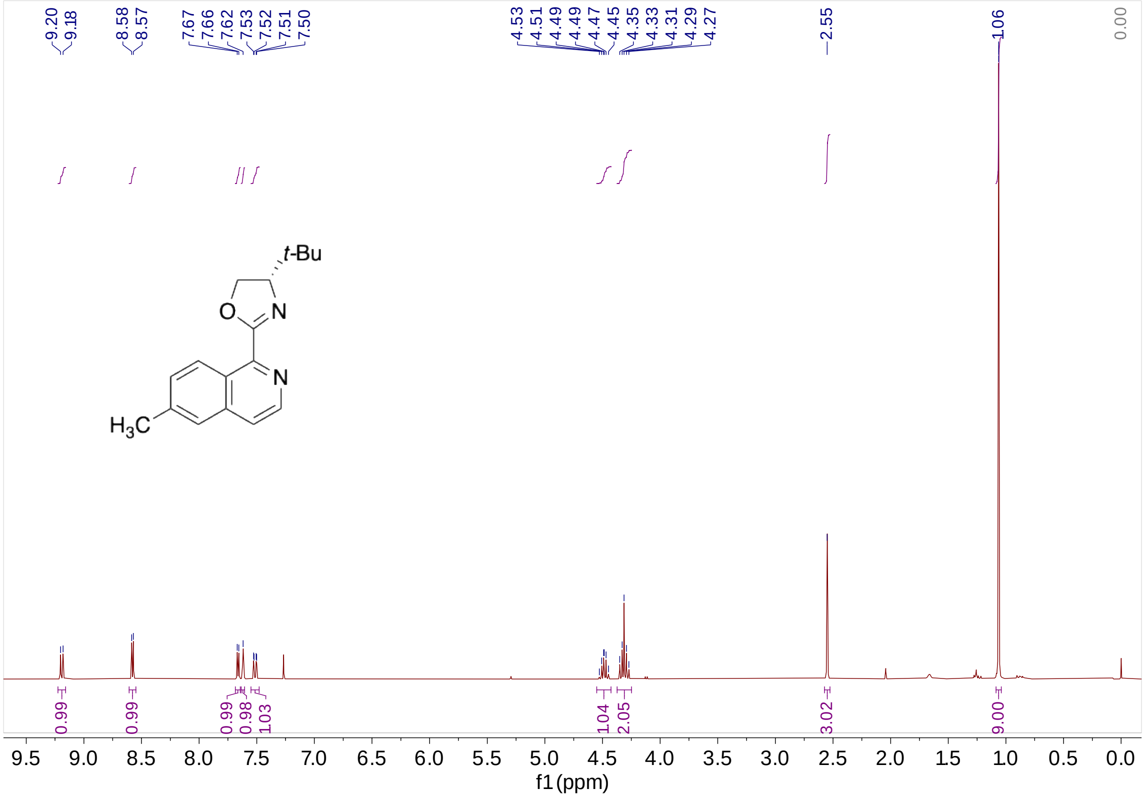

$^{13}C\{^{1}H\}$ *NMR (101 MHz, $CDCl_3$)*, **L27**

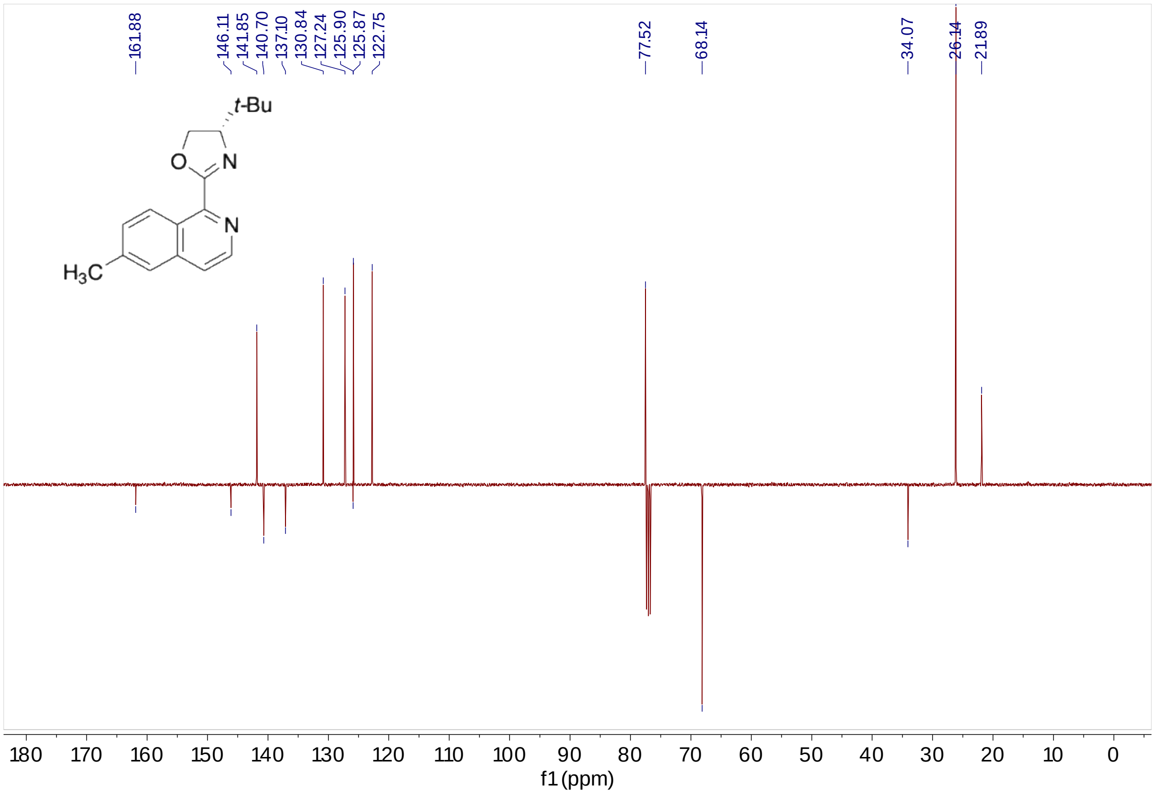

GCMS data and NMR spectra for Mechanistic Control Studies (Scheme 1).

GCMS data for the control experiment in the absence of Mn (eq 1)

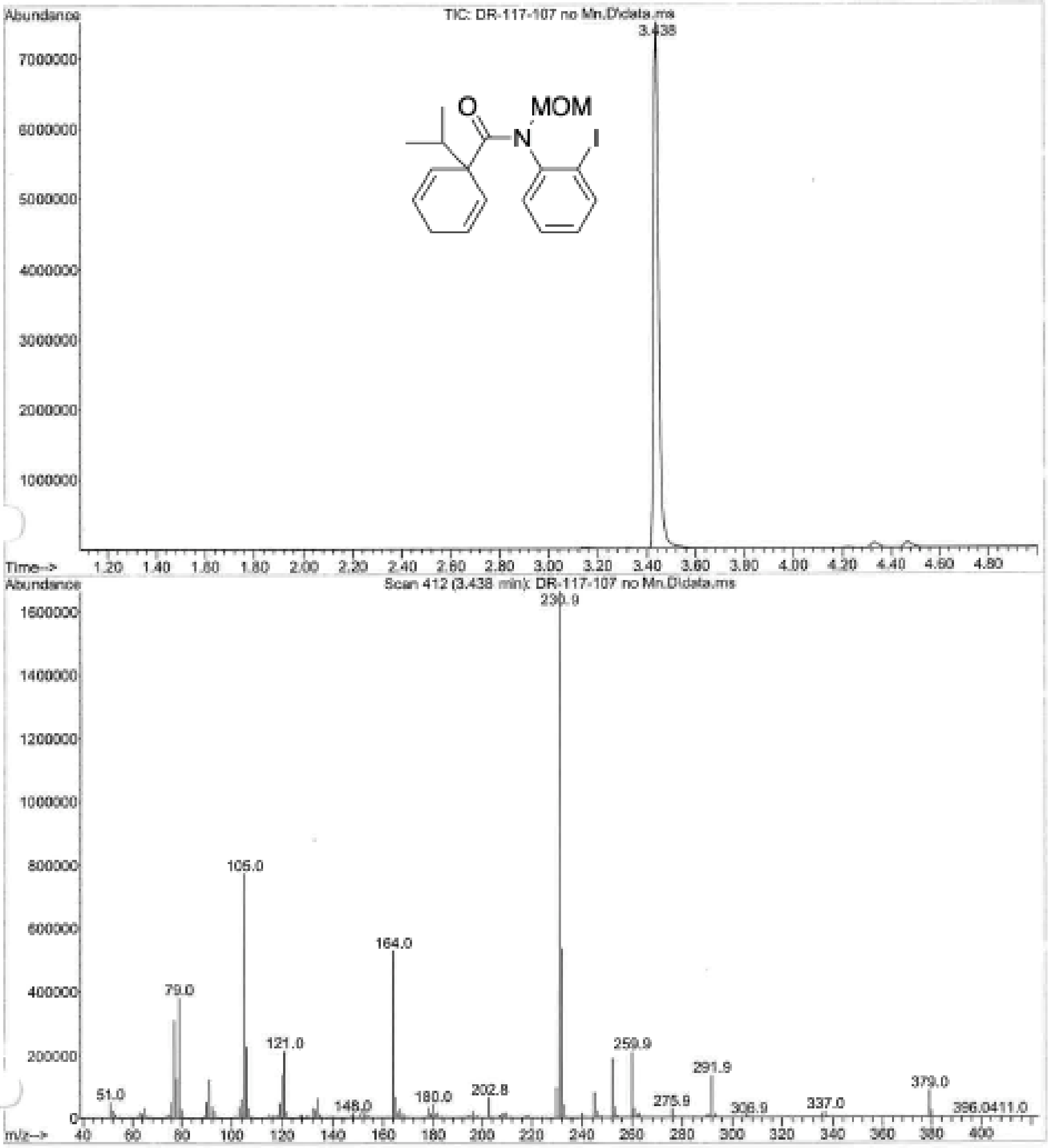

GCMS data for the control experiment using $Ni(COD)_2$ in the absence of Mn (eq 2)

peak #	R.T. min	first scan	max scan	last scan	PK TY	peak height	corr. area	corr. % max.	% of total
1	2.546	251	256	271	rBV	648018	790696	9.02%	8.277%
2	3.439	406	412	443	rBV	6805478	8762312	100.00%	91.723%

Sum of corrected areas: 9553008

AK1.M Fri Mar 10 14:55:31 2023

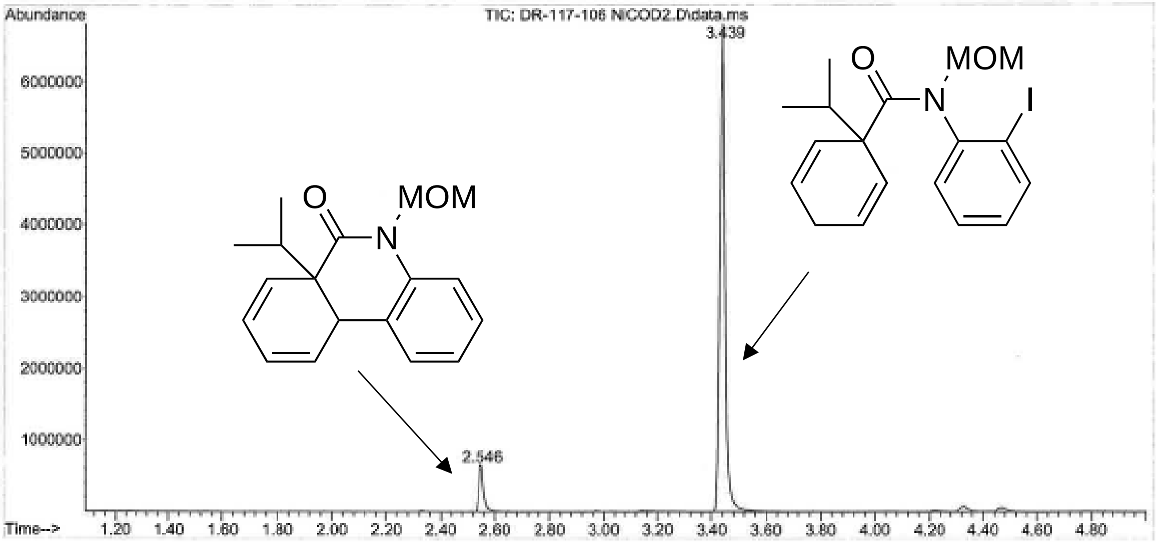

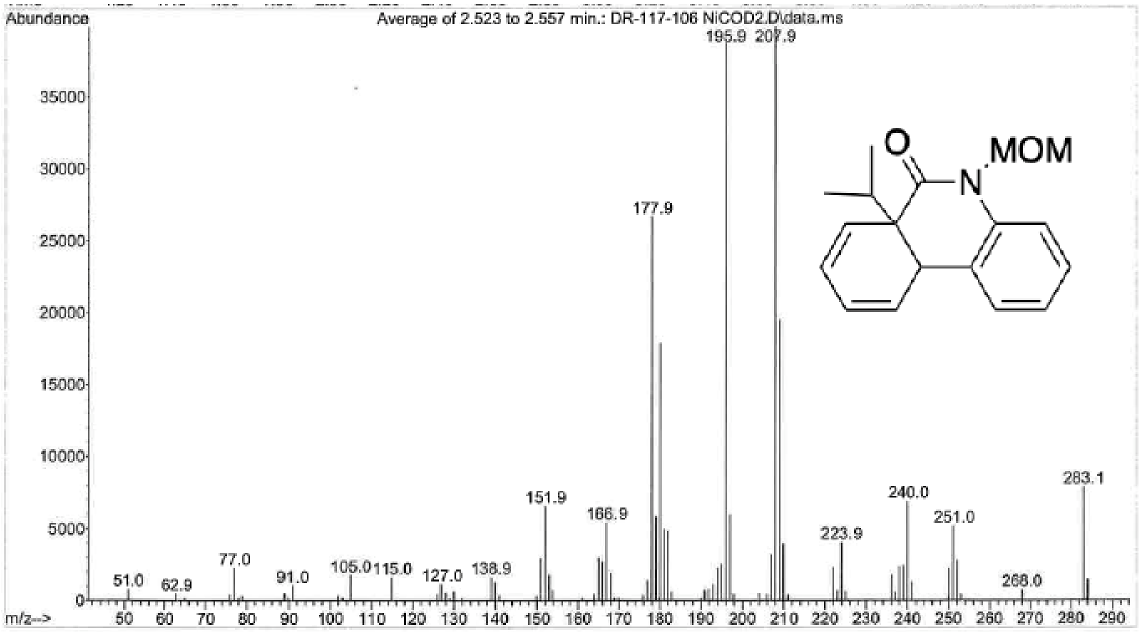

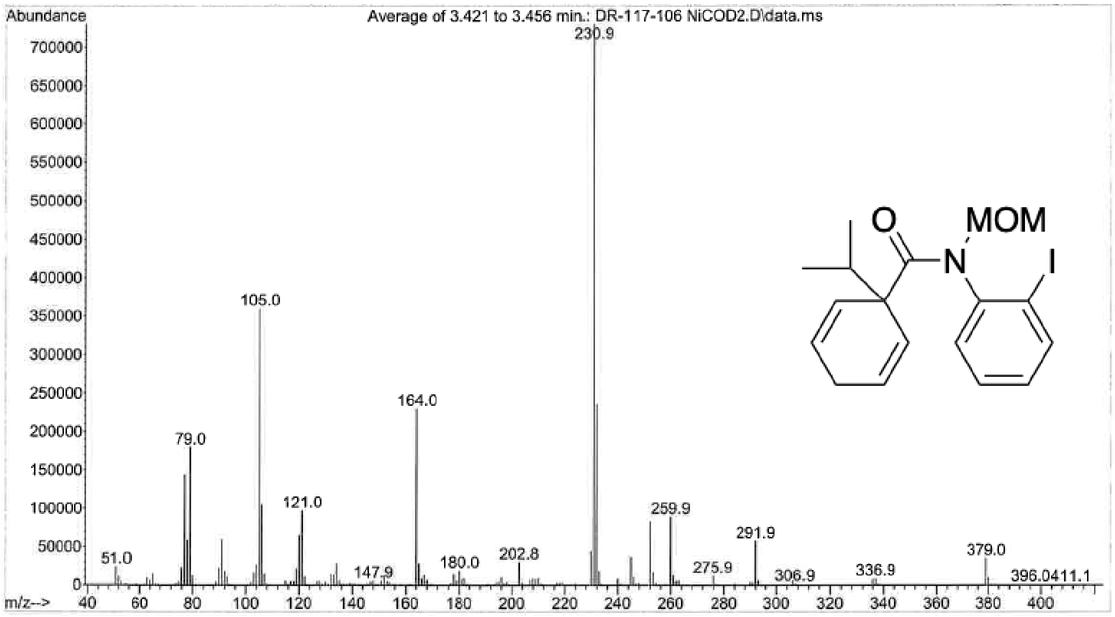

GCMS data for the control experiment using $Ni(COD)_2$ in the presence of Mn (eq 2)

peak #	R.T. min	first scan	max scan	last scan	PK TY	peak height	corr. area	corr. % max.	% of total
1	1.653	97	100	114	rVB	286010	482657	7.87%	6.146%
2	1.825	127	130	158	rVB	664557	1235721	20.14%	15.735%
3	2.546	250	256	270	rBV	5636612	6135189	100.00%	78.120%

Sum of corrected areas: 7853567

AK1.M Fri Mar 10 14:57:49 2023

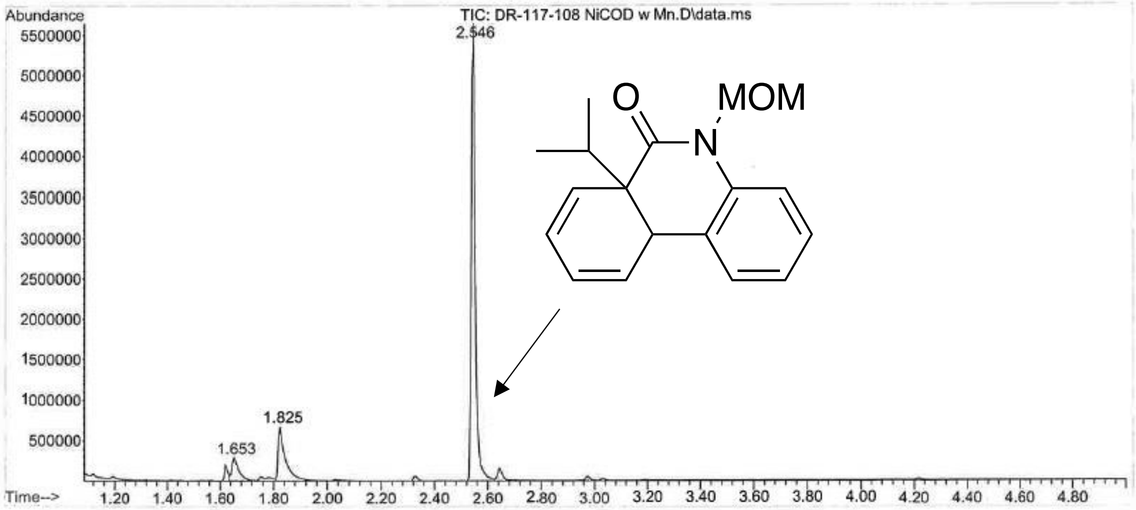

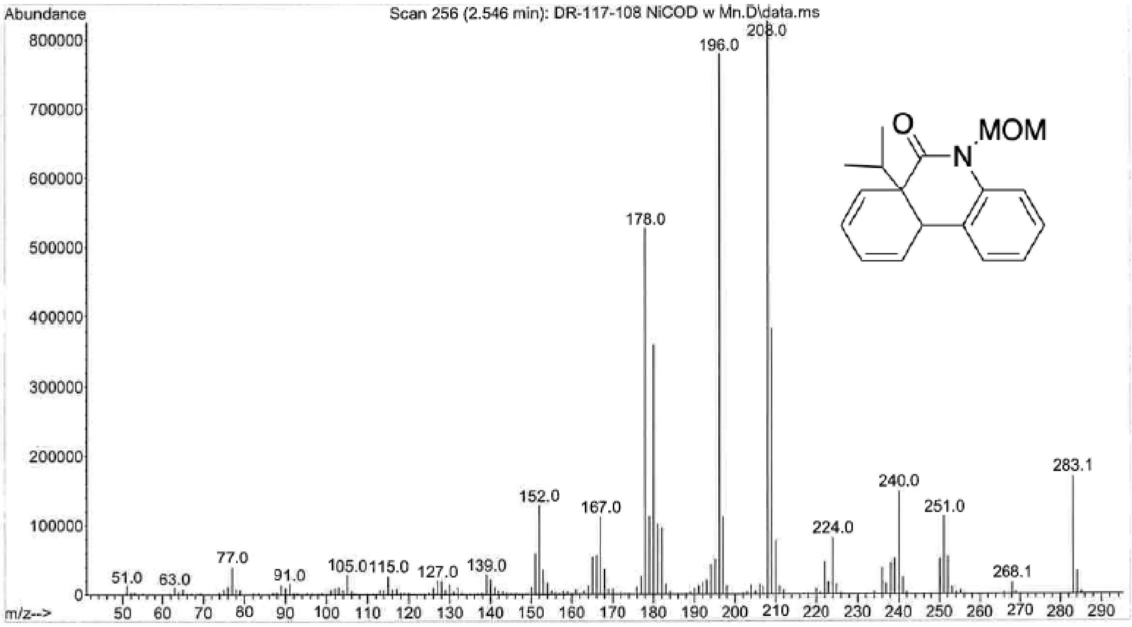

GCMS data for the control experiment using D_2O and Mn with the aryl bromide diene **1e** (eq 3-a)

1. GCMS of the starting material, **1e**

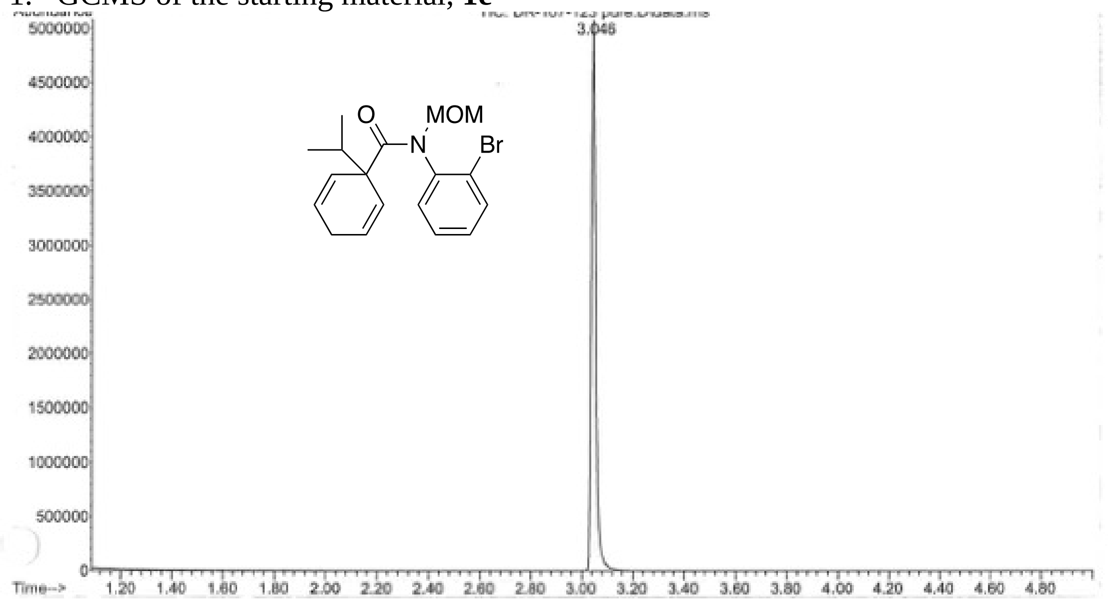

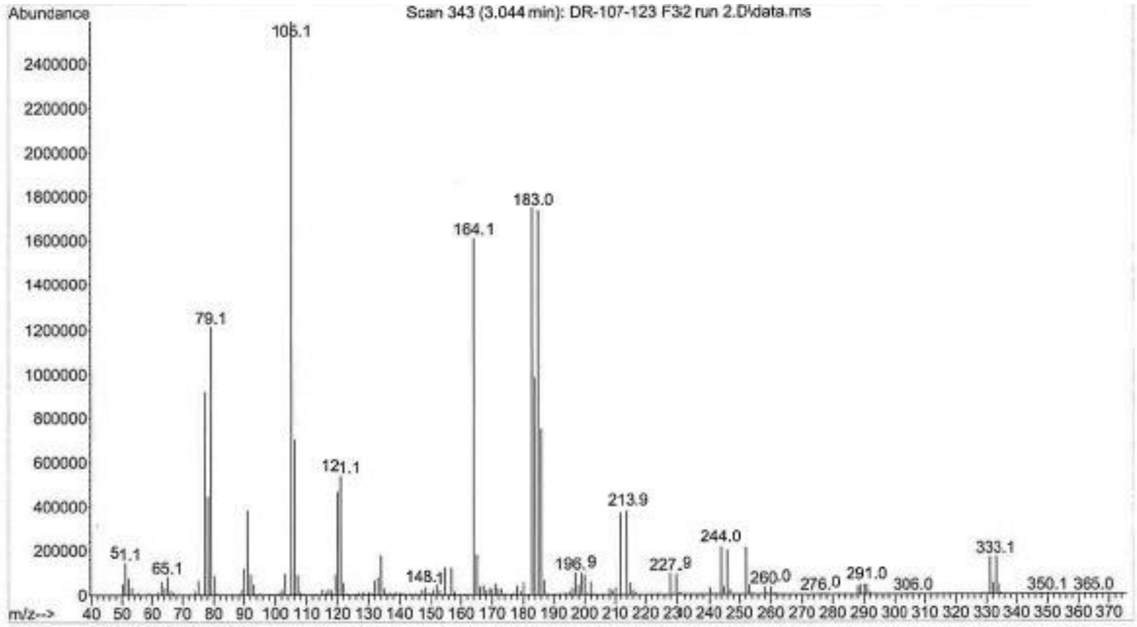

2. GCMS data for the control experiment with **1e** (eq 3-a)

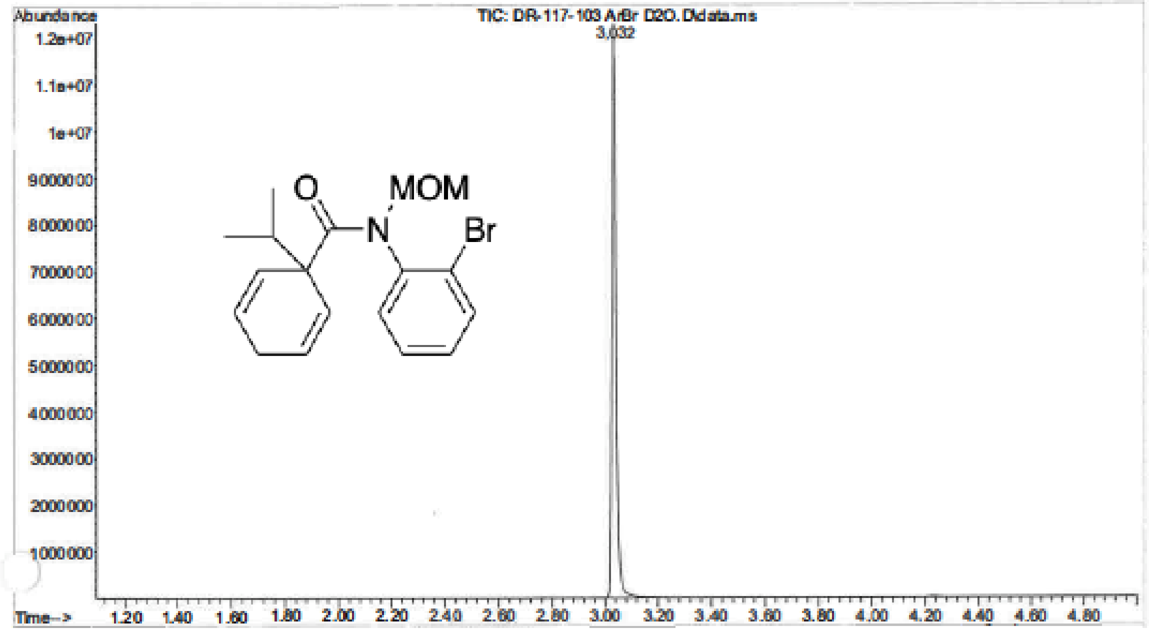

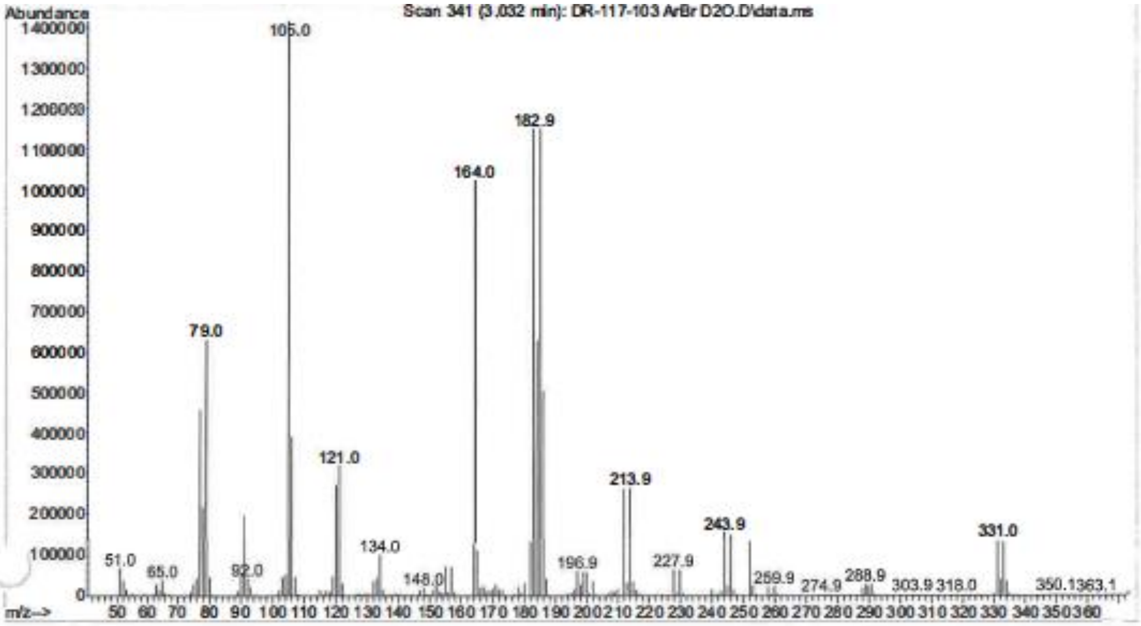

GCMS data for the control experiment using D_2O and Mn with the aryl iodide diene **1e-I** (eq 3-a)

1. GCMS of the starting material, **1e-I**

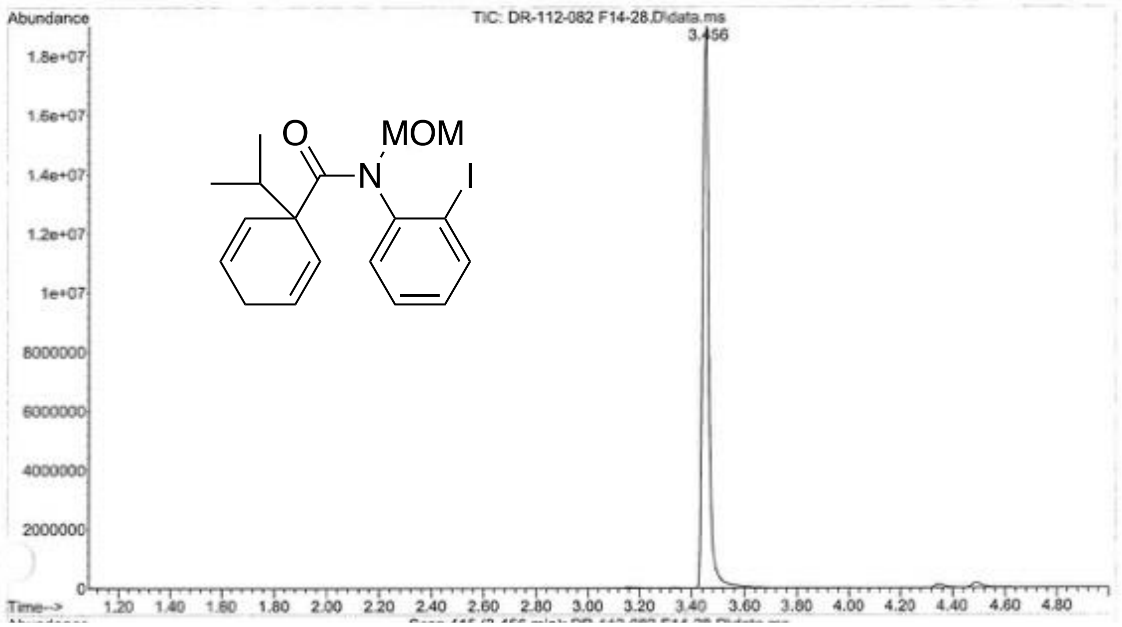

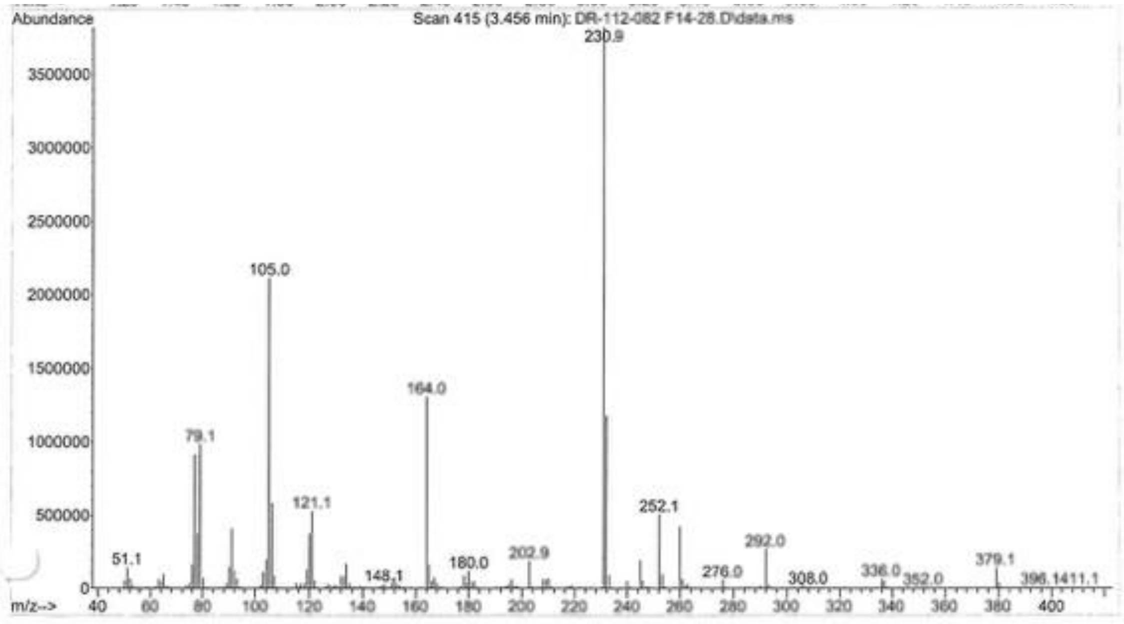

2. GCMS data for the crude Heck reaction with **1e-I** (non-deuterated reference)

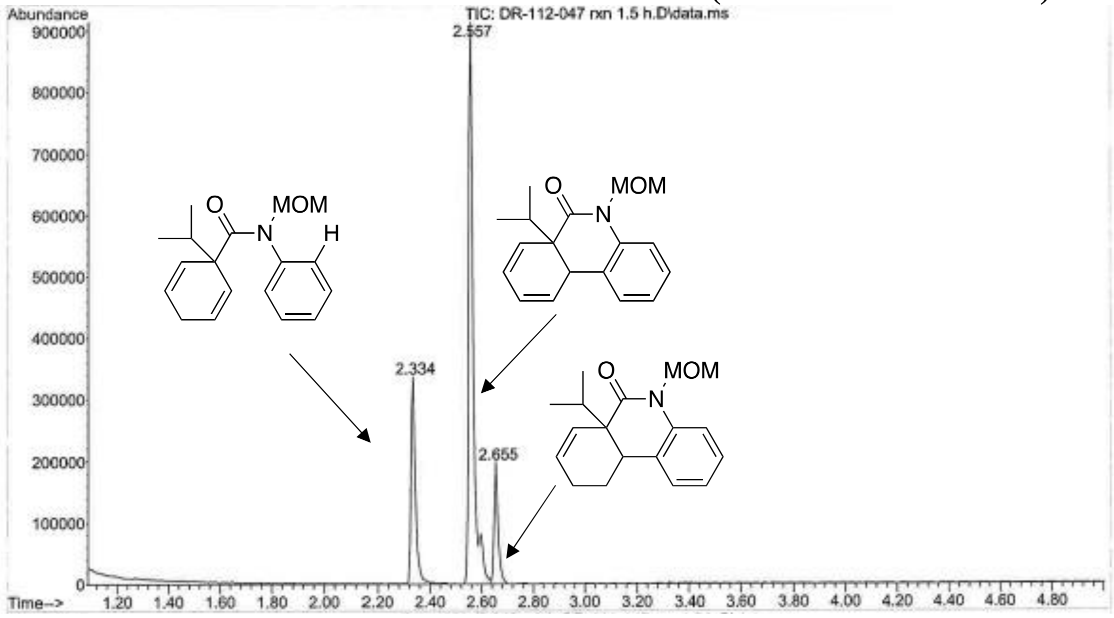

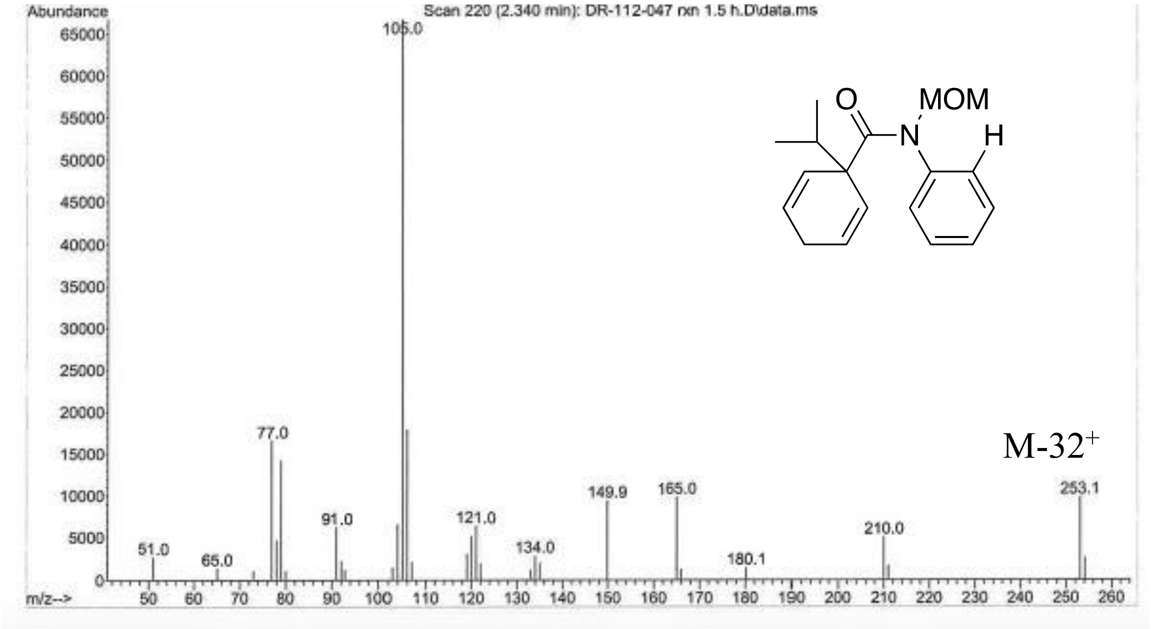

3. GCMS data for the control experiment with **1e-I** (eq 3-a)

peak #	R.T. min	first scan	max scan	last scan	PK TY	peak height	corr. area	corr. % max.	% of total
1	2.323	211	217	232	rBV	770405	891210	15.37%	13.322%
2	3.439	406	412	441	rBV	4039574	5798656	100.00%	86.678%

Sum of corrected areas: 6689866

AK1.M Fri Mar 10 15:05:49 2023

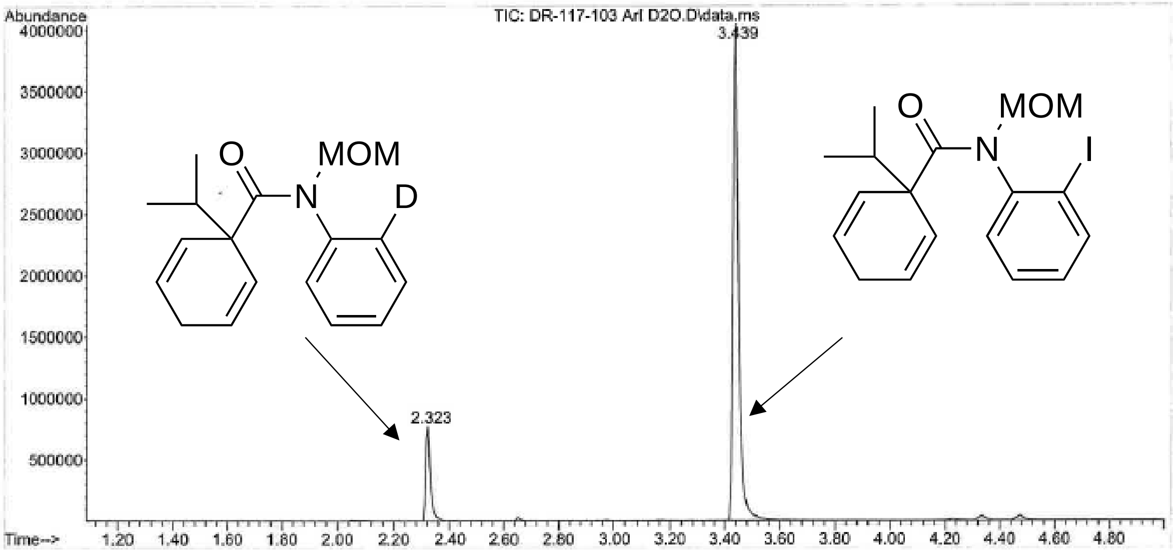

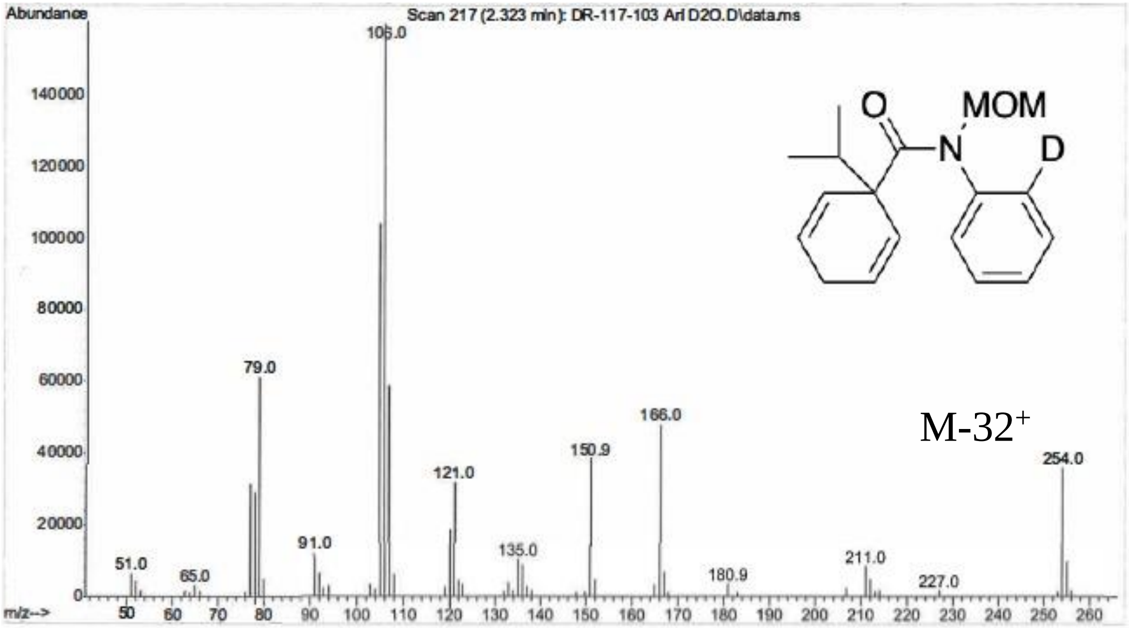
Abundance
Scan 217 (2.323 min): DR-117-103 ArI D2O.D\data.ms
106.0
79.0
91.0
51.0
65.0
121.0
135.0
150.9
166.0
180.9
211.0
227.0
254.0
M-32+
O
MOM
N
D
m/z-->
140000
120000
100000
80000
60000
40000
20000
0
50 60 70 80 90 100 110 120 130 140 150 160 170 180 190 200 210 220 230 240 250 260

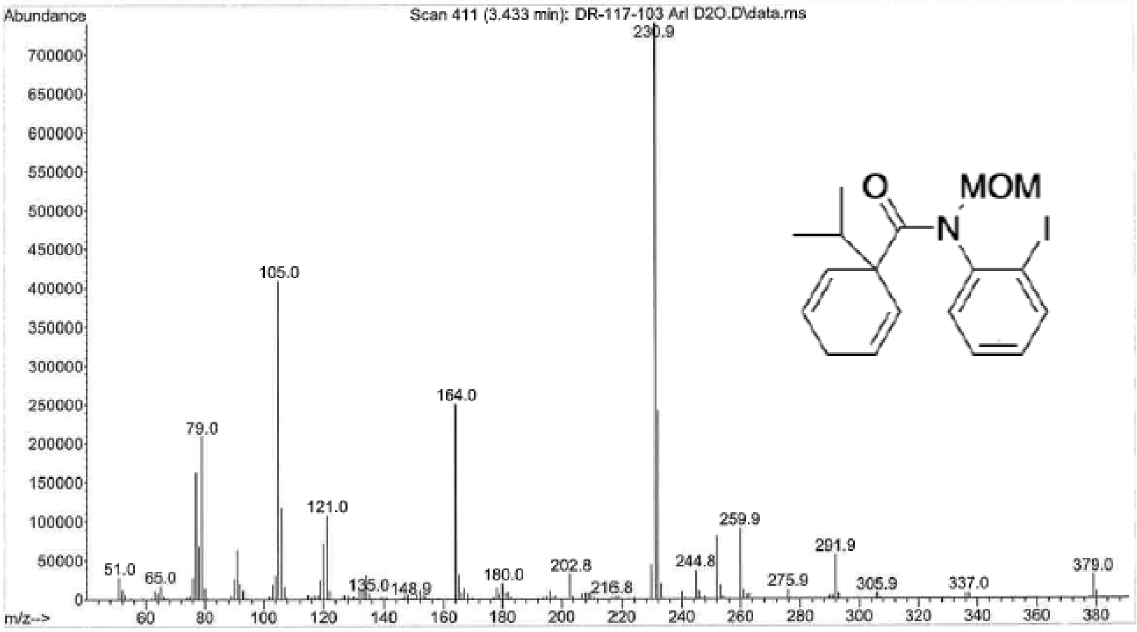
Abundance
Scan 411 (3.433 min): DR-117-103 ArI D2O.D\data.ms
230.9
105.0
164.0
79.0
121.0
259.9
291.9
51.0
65.0
135.0
148.9
180.0
202.8
216.8
244.8
275.9
305.9
337.0
379.0
O
MOM
N
I
m/z-->
700000
650000
600000
550000
500000
450000
400000
350000
300000
250000
200000
150000
100000
50000
0
60 80 100 120 140 160 180 200 220 240 260 280 300 320 340 360 380

GCMS data for the control experiment using D_2O and Zn with the aryl bromide diene **1d** (eq 3-b)

1. GCMS of the starting material, **1d**

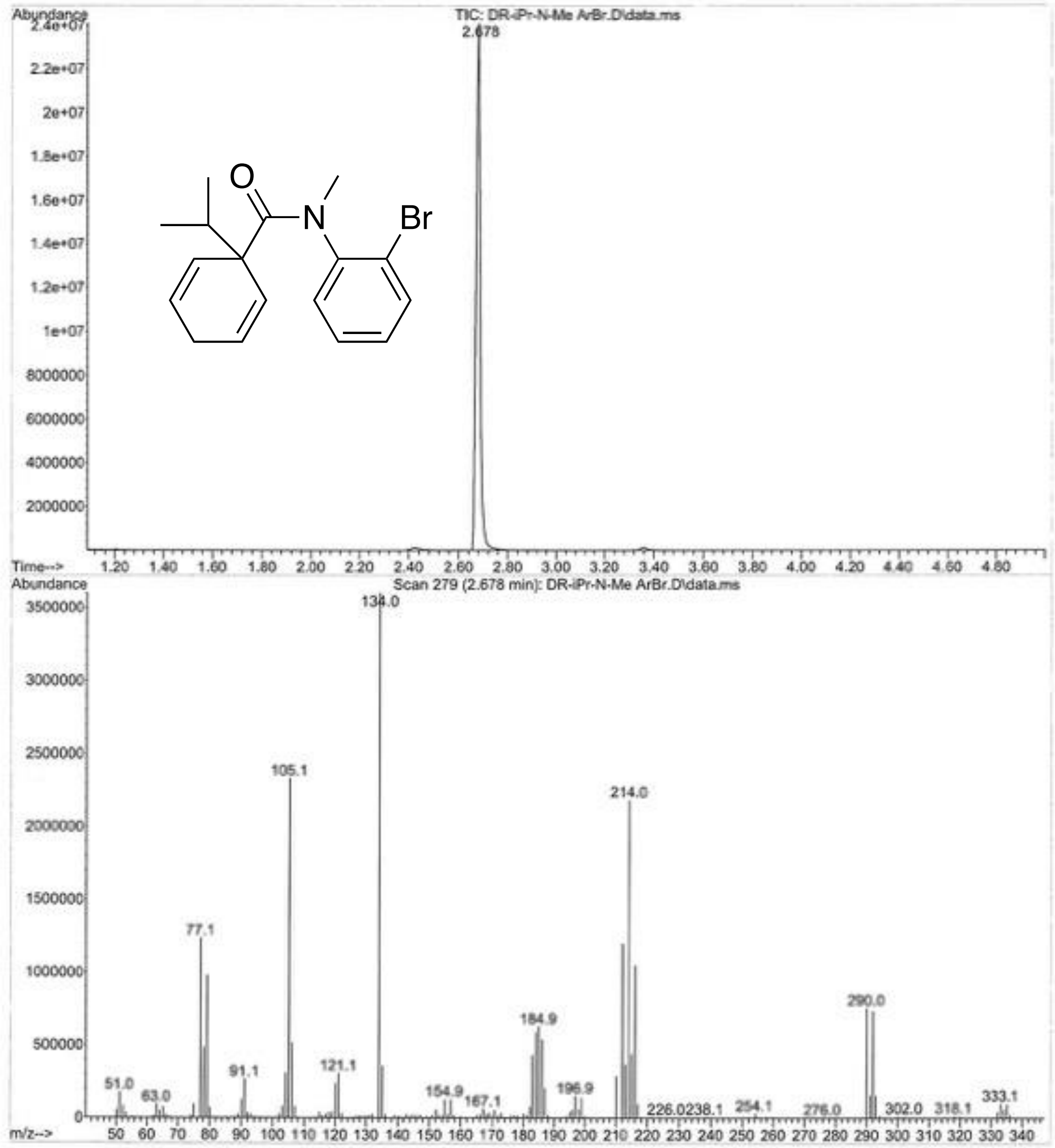

2. GCMS data for the control experiment with **1d** (eq 3-b)

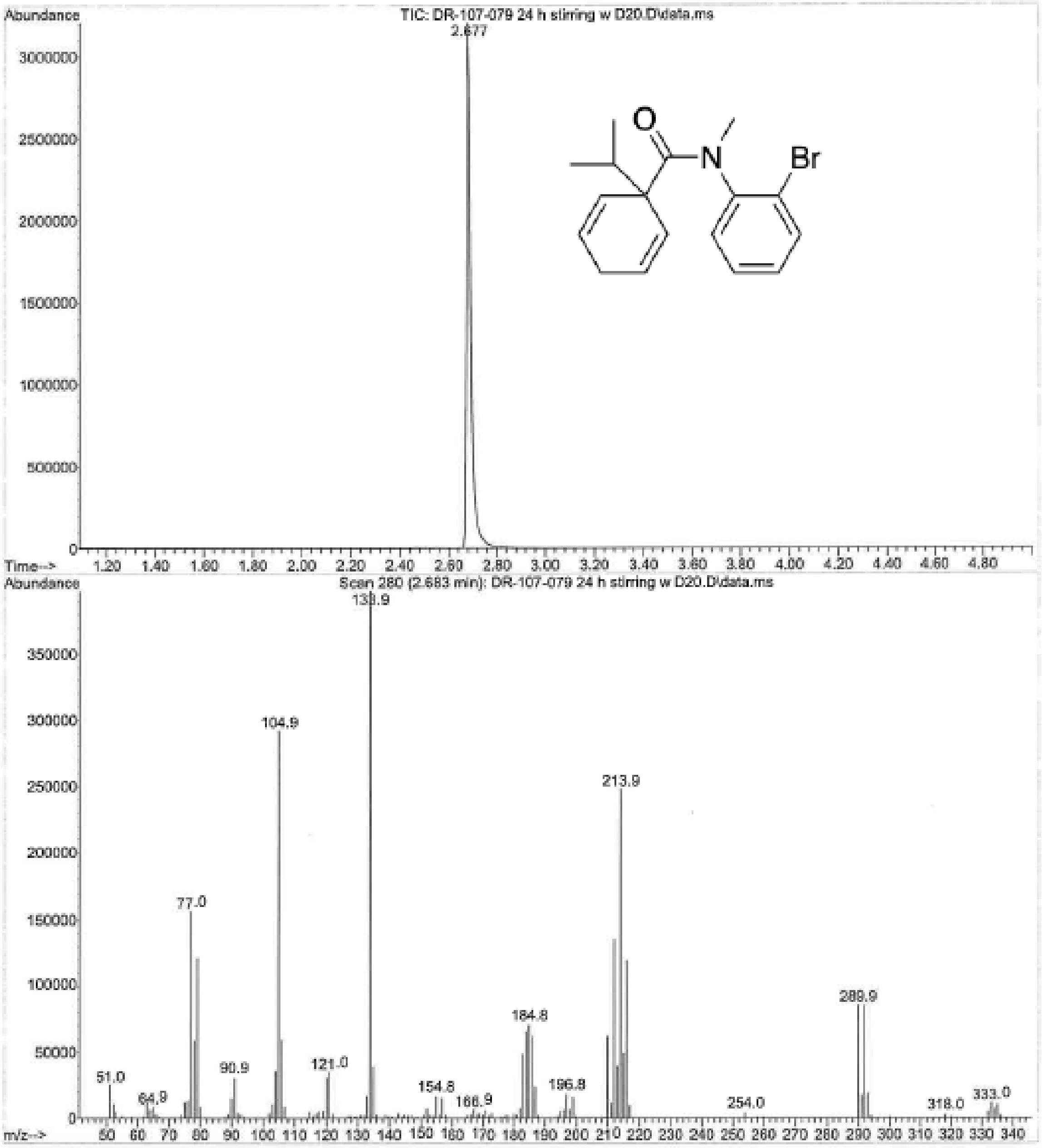

GCMS and NMR data for the control experiment using D_2O and Zn with the aryl iodide diene **1d-I** (eq 3-b)

1. GCMS of the starting material, **1d-I**

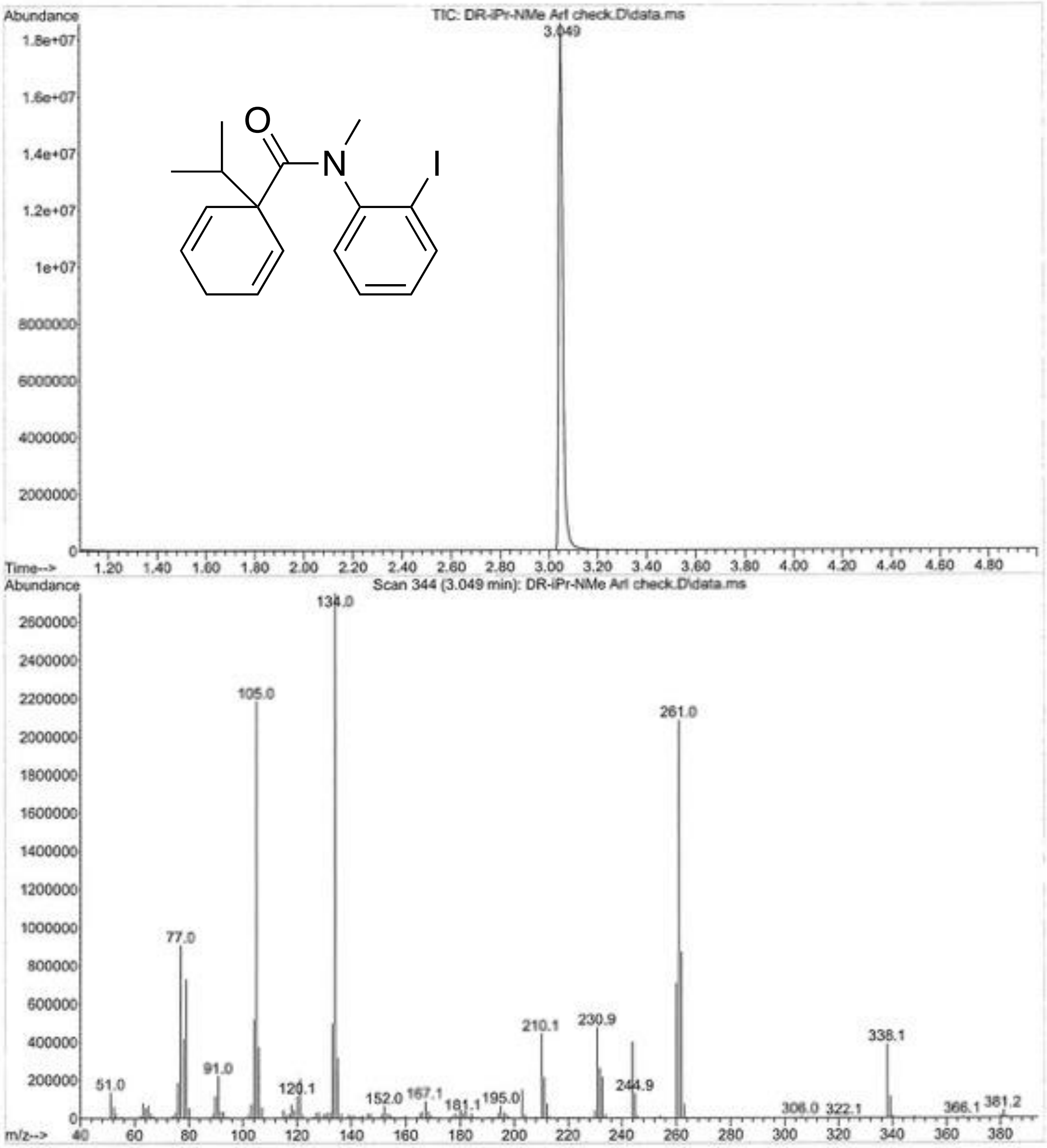

2. GCMS data for the Heck crude reaction with **1d-I** (non-deuterated reference)

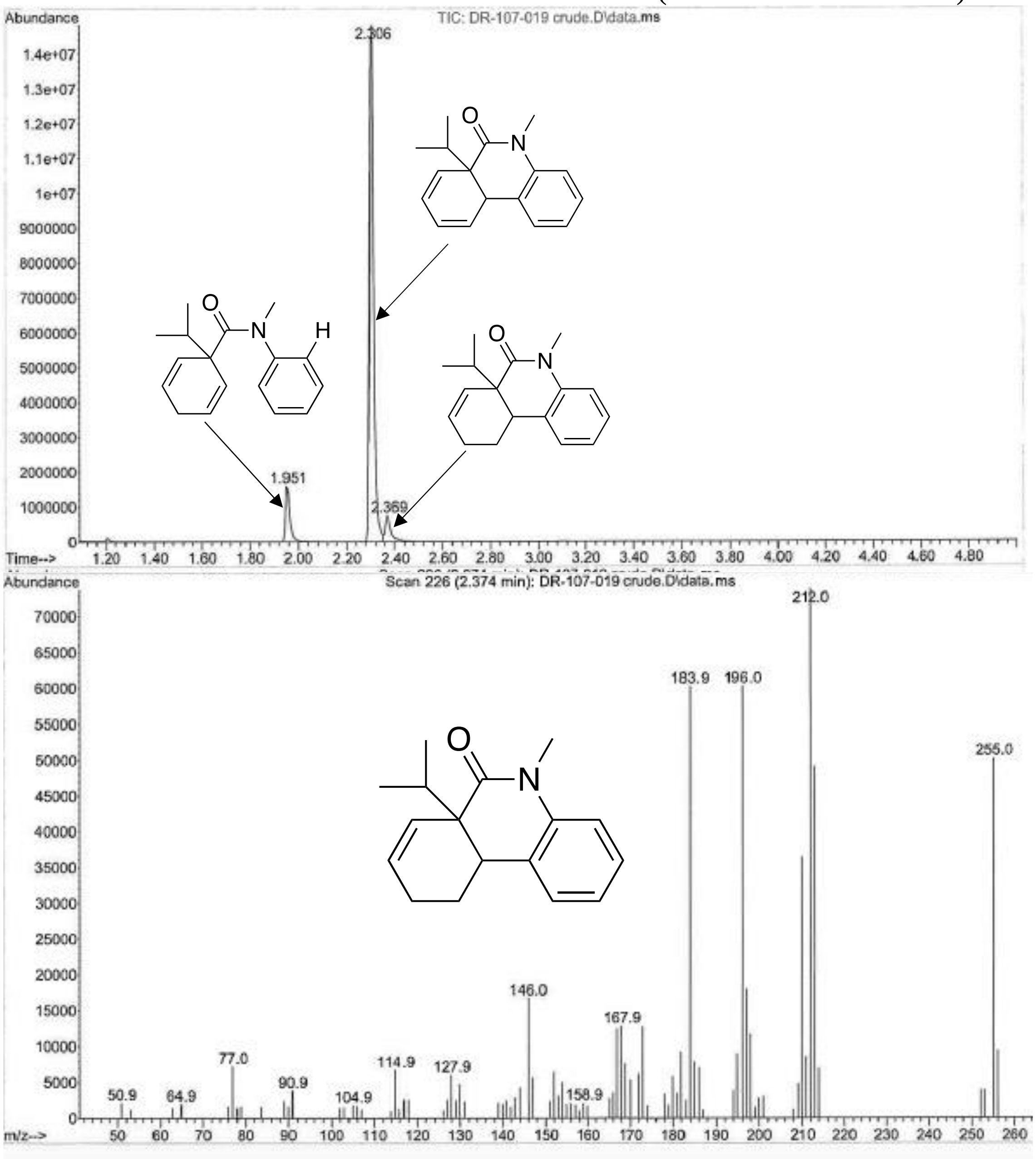

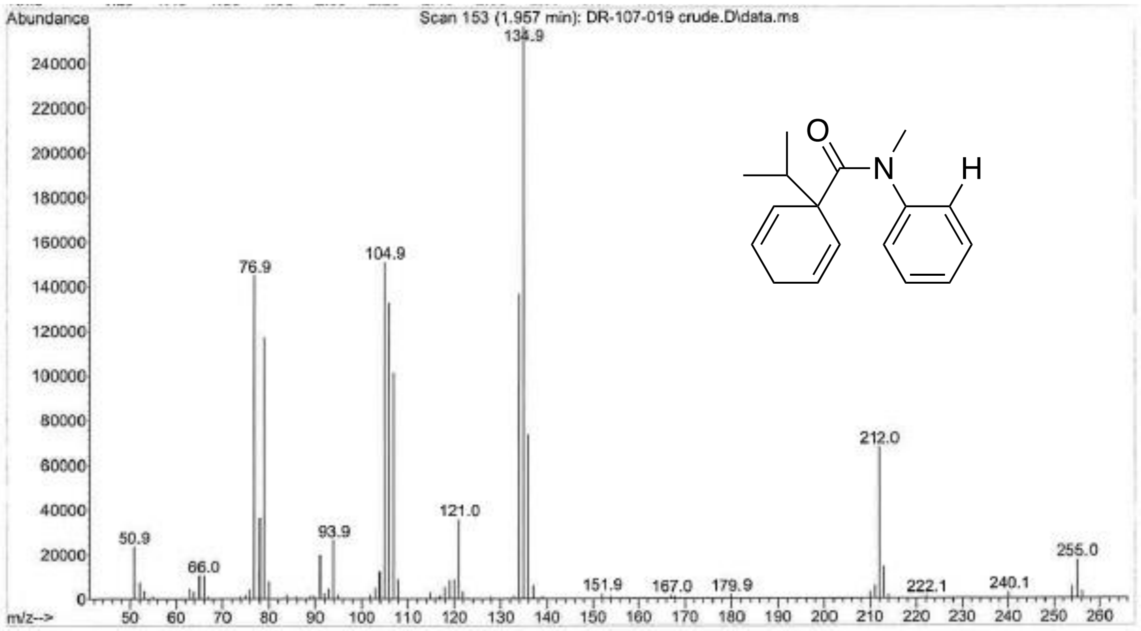

3. GCMS data for the control experiment with **1d-I** (eq 3-b)

peak #	R.T. min	first scan	max scan	last scan	PK TY	peak height	corr. area	corr. % max.	% of total
1	1.951	147	152	183	rBV	7055466	6868842	100.00%	81.943%
2	2.368	221	225	245	rBB	1454169	1513635	22.04%	18.057%

Sum of corrected areas: 8382477

AK1.M Wed Mar 15 10:01:10 2023

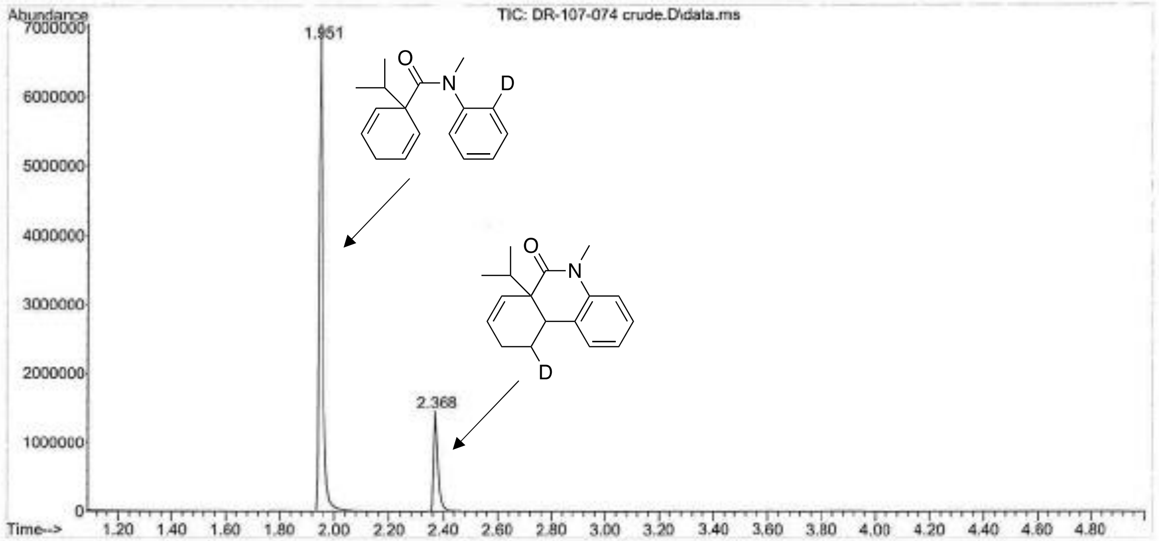

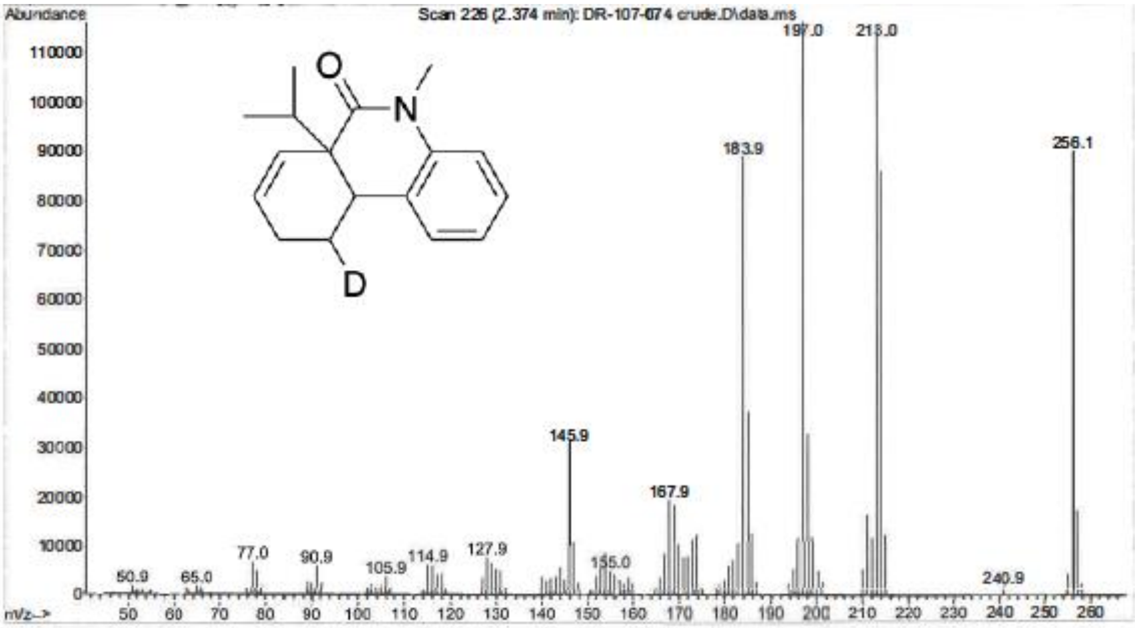
Scan 226 (2.374 min): DR-107-074 crude.D\data.ms
Abundance
m/z-->
197.0
213.0
183.9
256.1
145.9
167.9
77.0
90.9
105.9
114.9
127.9
155.0
50.9
65.0
240.9
D
O
N

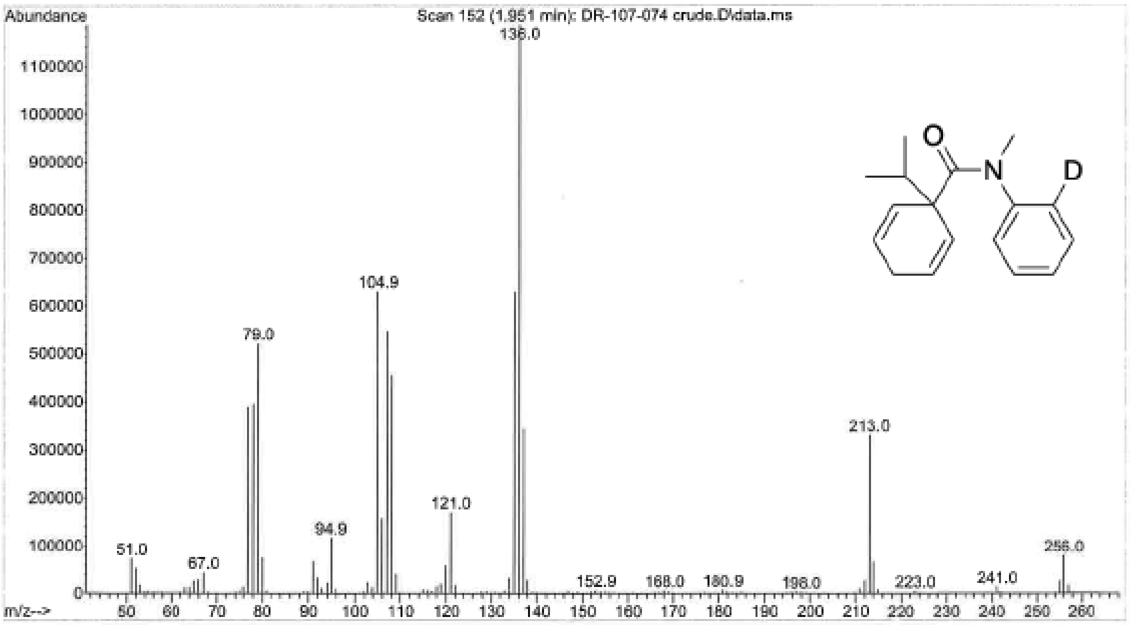
Scan 152 (1.951 min): DR-107-074 crude.D\data.ms
Abundance
m/z-->
136.0
104.9
79.0
213.0
121.0
94.9
51.0
67.0
152.9
168.0
180.9
198.0
223.0
241.0
256.0
O
N
D

^{1}H NMR (400 MHz), **2d-1**

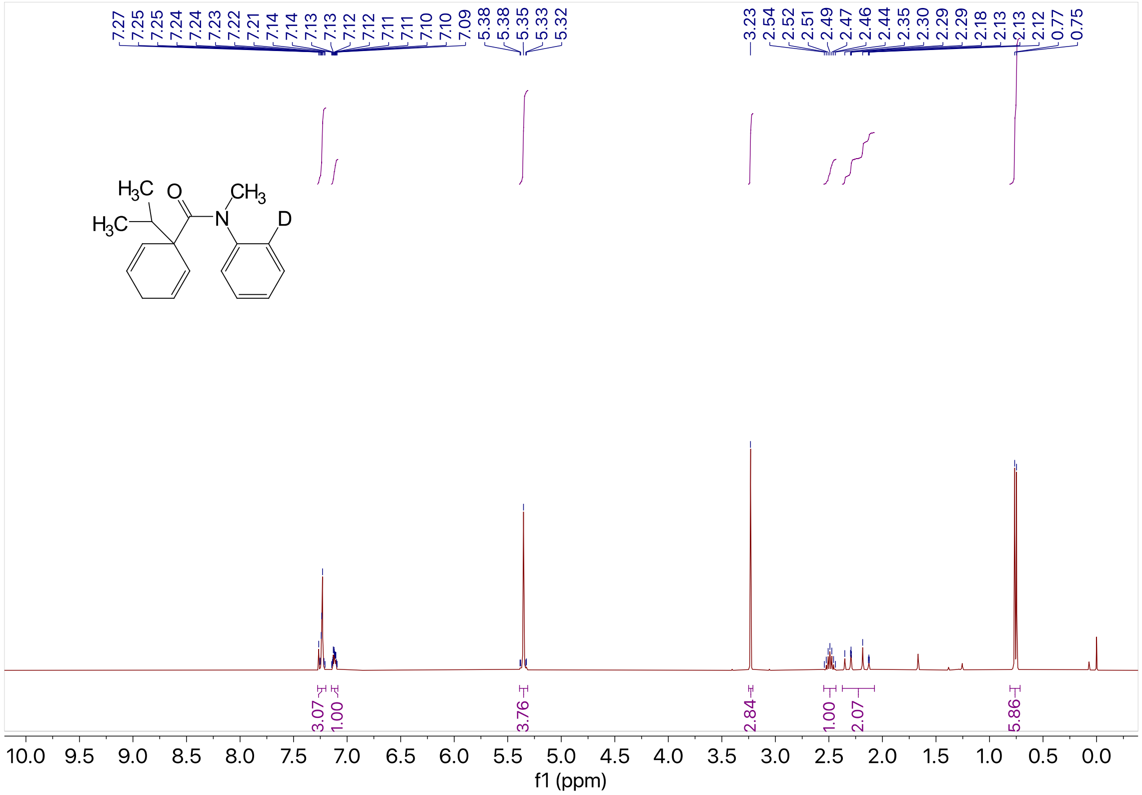

^{13}C{^{1}H} NMR (101 MHz, $CDCl_3$), **2d-1**

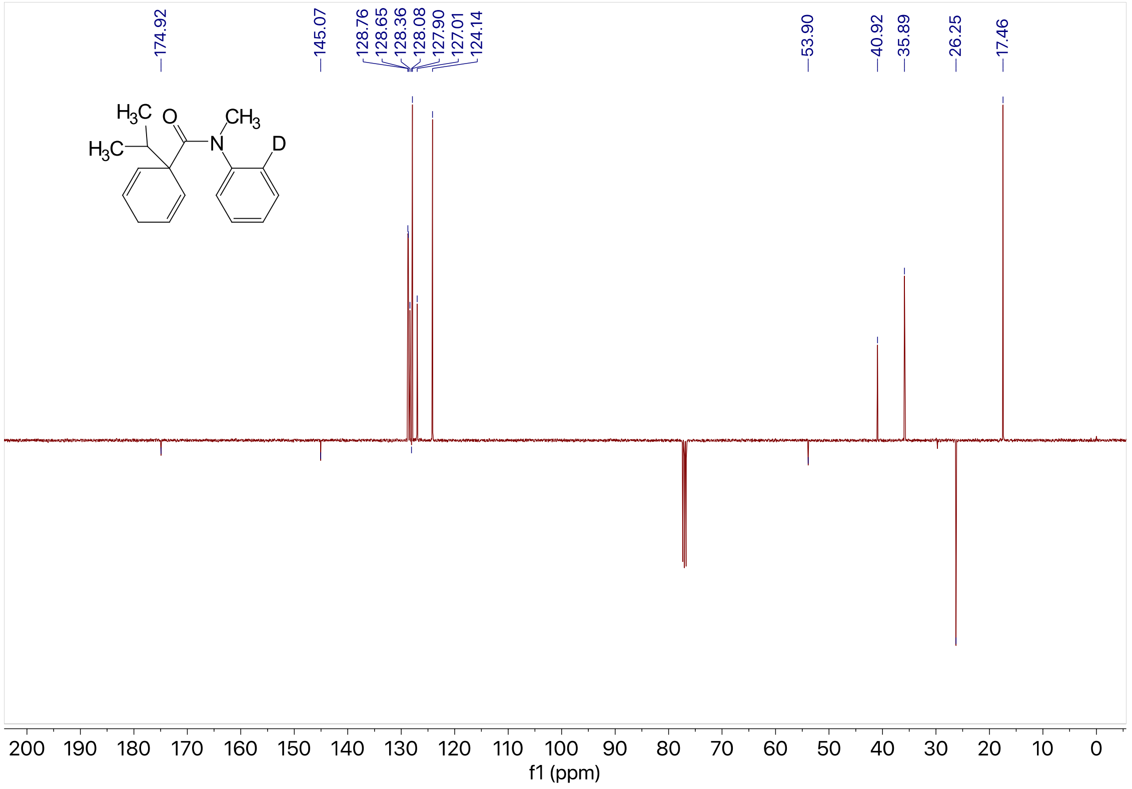

GCMS data for the control experiment using 2-cyclohexenone and Mn (eq 4)

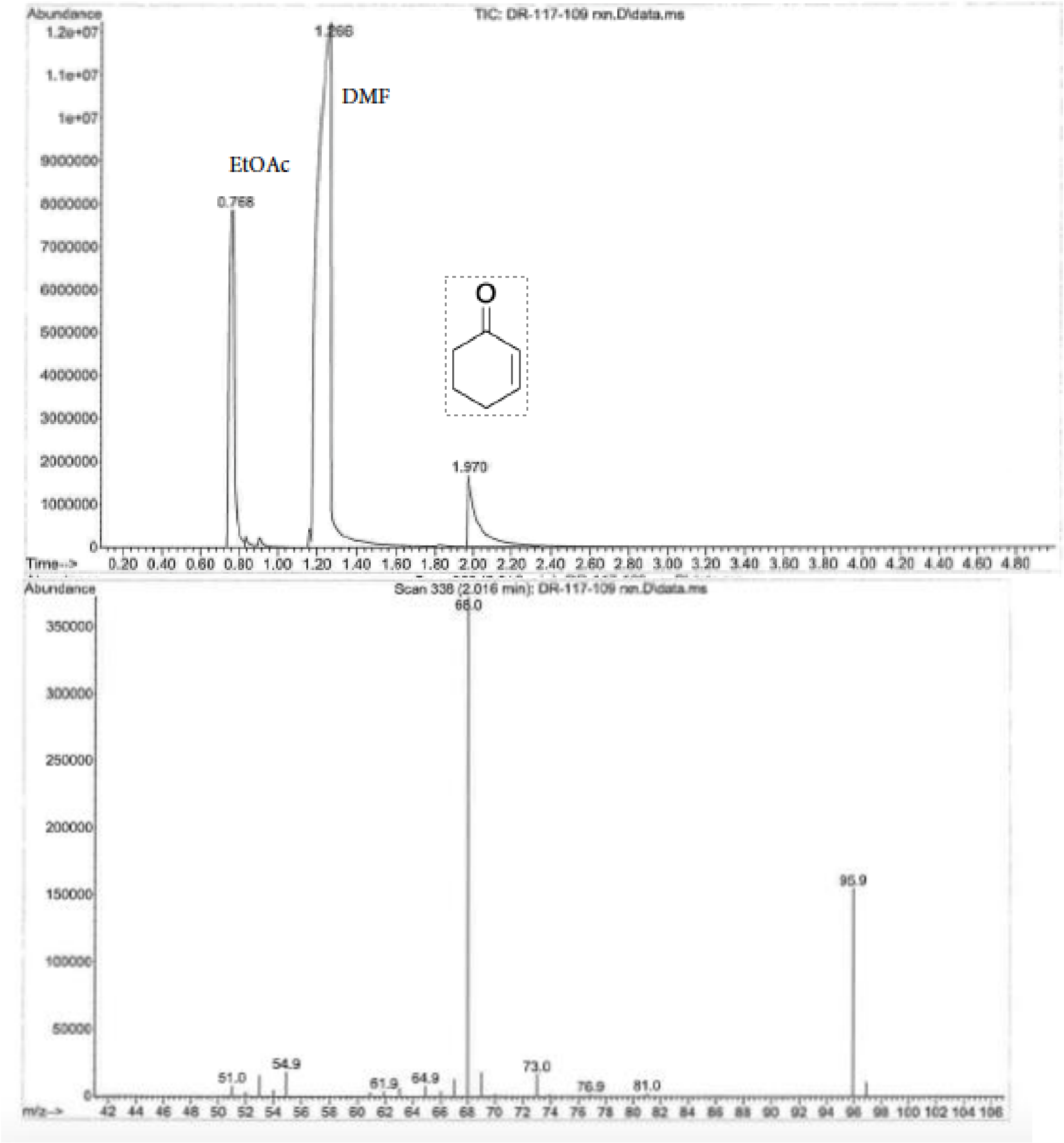

Sample Name: DR-2-cyclohexenone control
Misc Info :

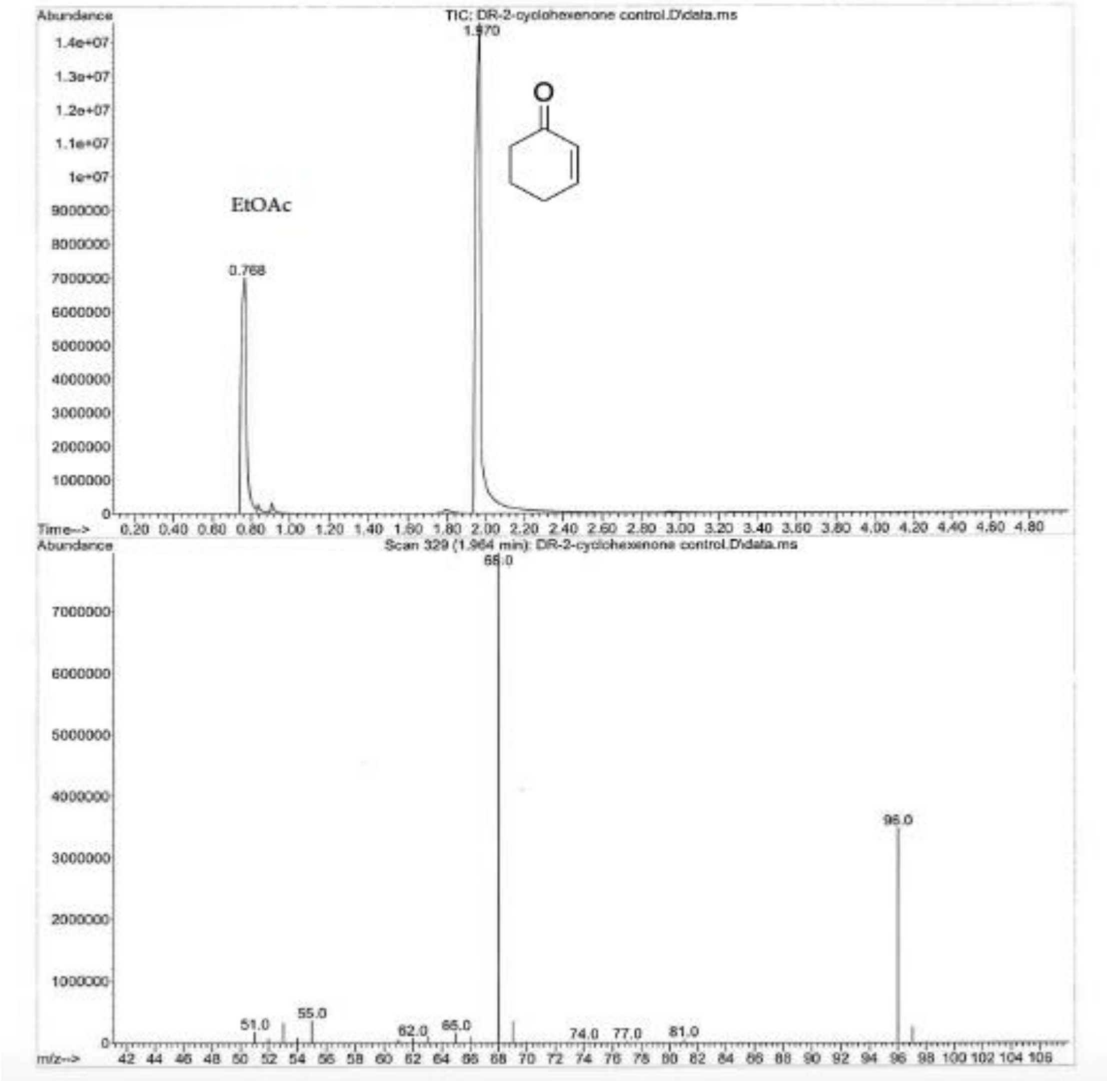

Sample Name: DR-cyclohexanone control
Misc Info :

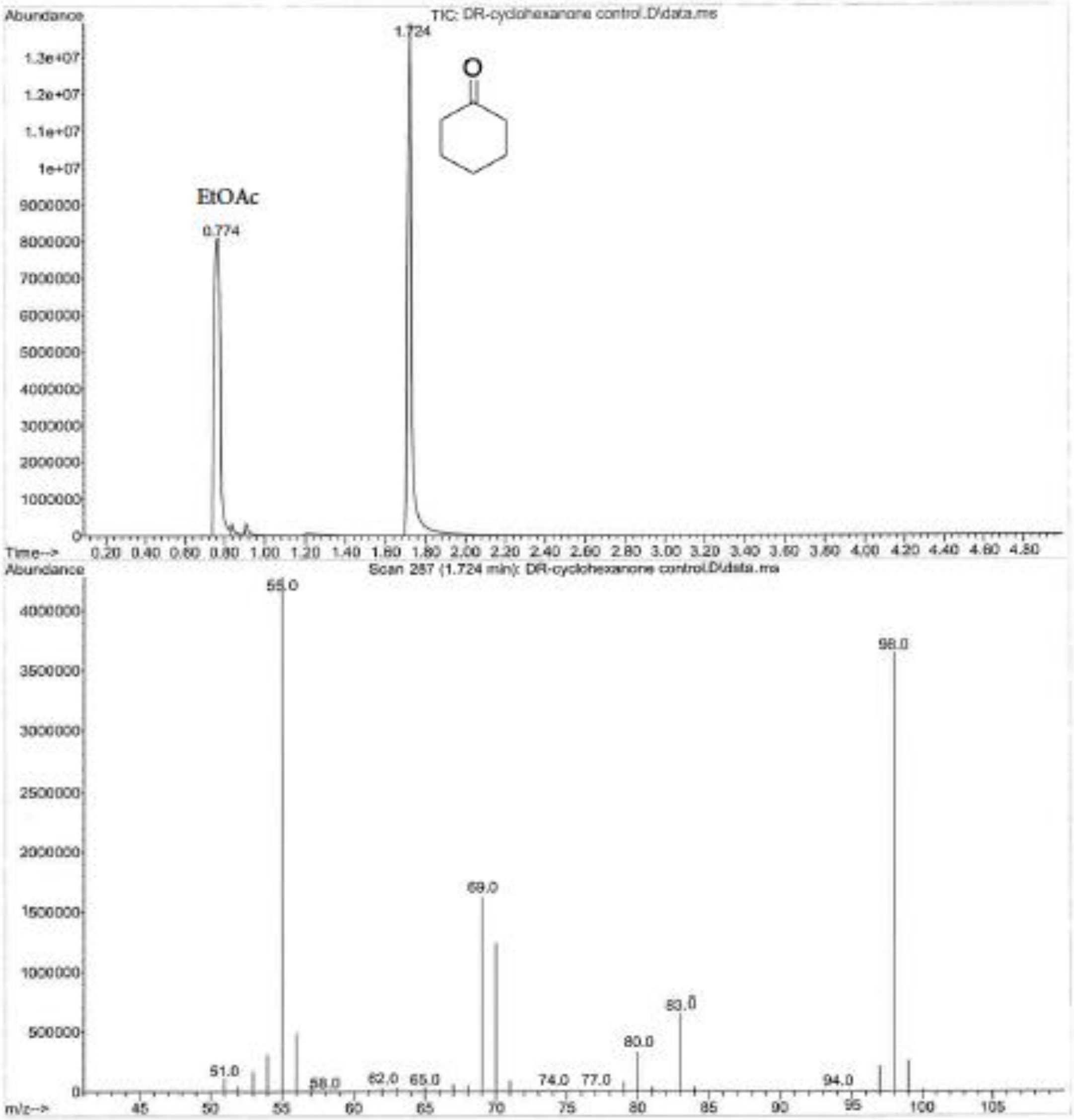

GCMS data for the control experiment using TEMPO (eq 5)

peak #	R.T. min	first scan	max scan	last scan	PK TY	peak height	corr. area	corr. % max.	% of total
1	2.020	159	164	182	rBV	149144	299156	6.93%	6.477%
2	2.546	250	256	271	rBV	3940128	4319310	100.00%	93.523%

Sum of corrected areas: 4618466

AK1.M Fri Mar 10 15:09:24 2023

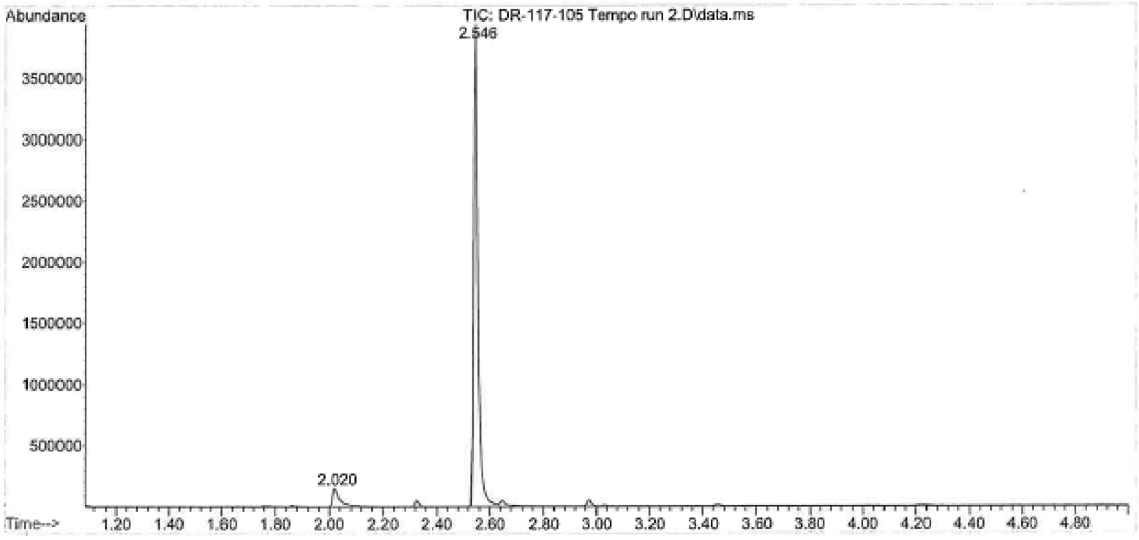

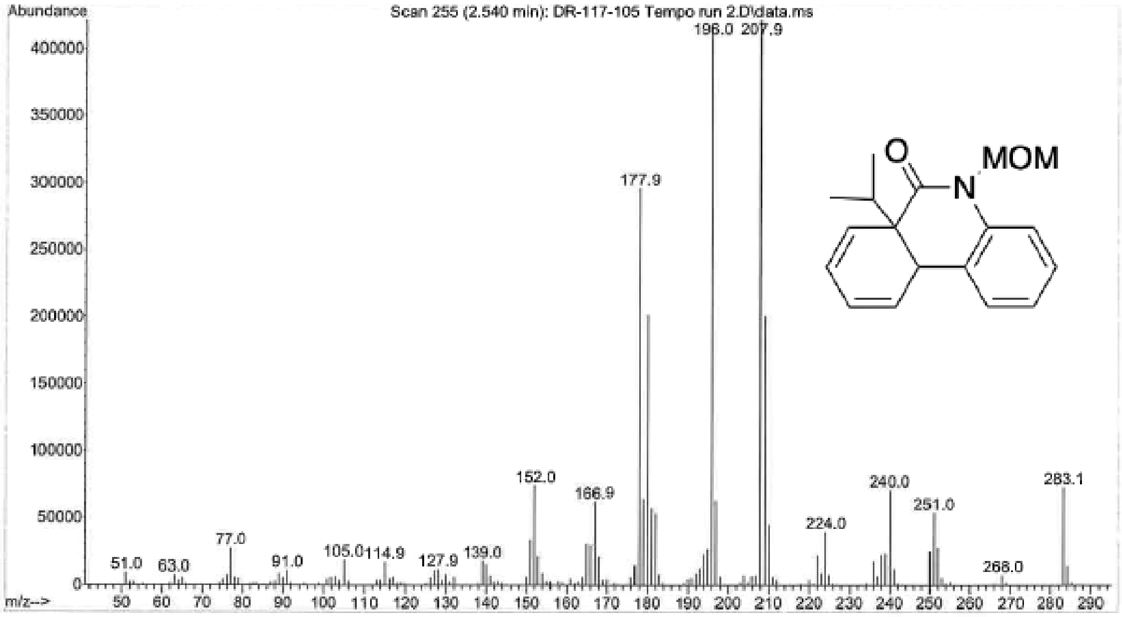

Computational Data

SI-Figure 1. Conformation Images and Relative Energy.

Conformational Changes:

"**R**" = parent configuration

"**A**" = Ni complexed to other **a**lkene of cyclohexadiene (compared to parent)

"**F**" = tBu-(6)CH3iQuinox ligand **f**lipped ~180 ° (compared to parent)

"**T**" = amide **t**wisted other way (compared to parent)

Numbers in parentheses are the Gibbs free energies (in kcal/mol) of the transition structures relative to the global minimum for intermediate B.

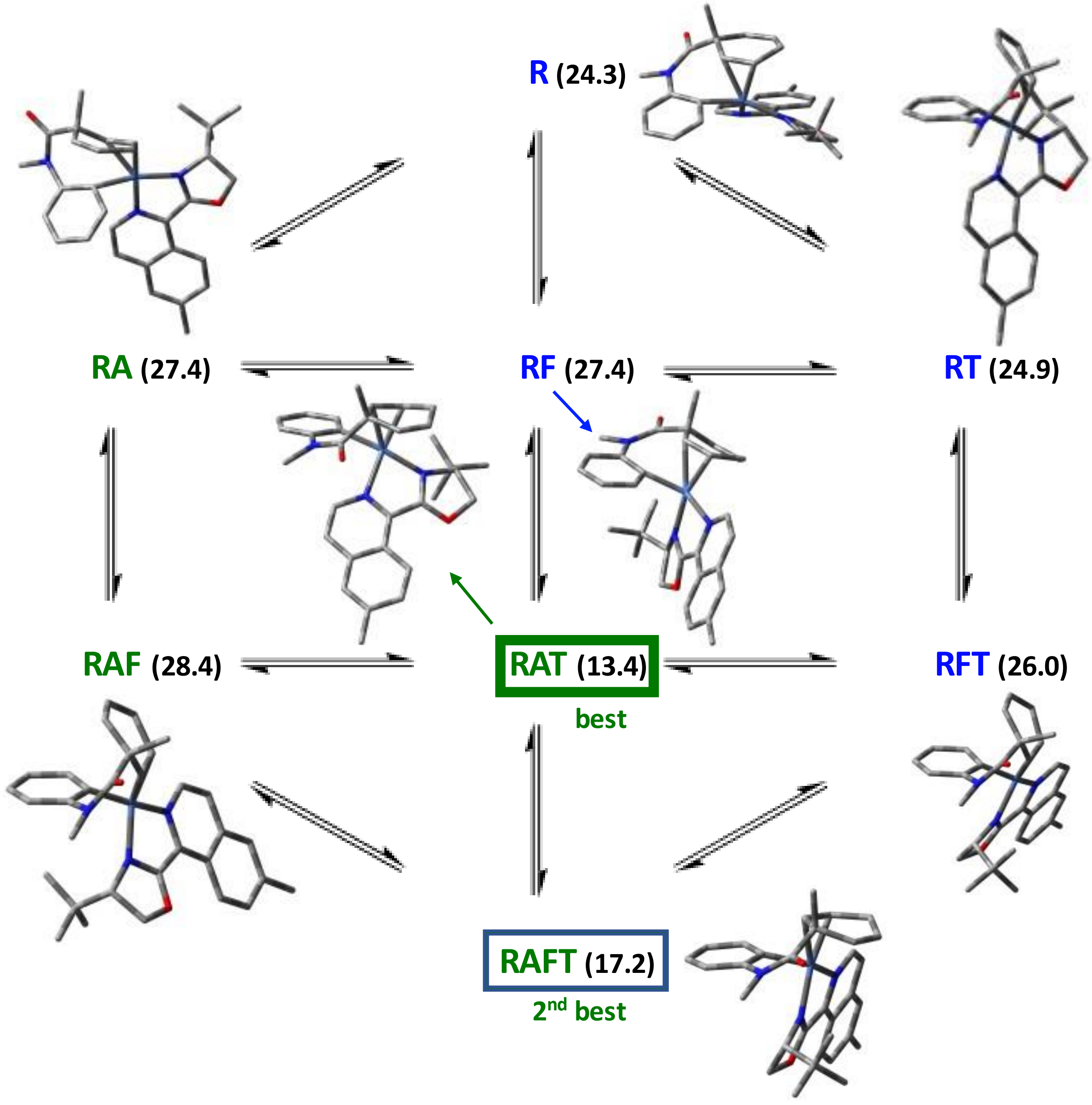

RA, RAF, RAT, and RAFT proceed to the observed **(S,S)** stereochemistry of product.

R, RF, RT, and RFT proceed to the unobserved **(R,R)** stereochemistry of product

SI-Table 5. Calculations.

```
Intermediate B of catalytic cycle for Ni-catalyzed Mizoroki-Heck reaction, bipy
ligand, R1=Me, R2=Me, R3=H

Species name:    intB
Full file name:  intB.log
Command line:    B3LYP/Def2SVP empiricaldispersion=GD3BJ freq geom=check
guess=read
Point group:     C1
NImag:           0

Thermochemistry:
Temperature:     298.150
Zero-point correction=                           0.438748 (Hartree/Particle)
Thermal correction to Energy=                    0.464328
Thermal correction to Enthalpy=                  0.465272
Thermal correction to Gibbs Free Energy=         0.383724
Sum of electronic and zero-point Energies=          -2713.418652
Sum of electronic and thermal Energies=             -2713.393071
Sum of electronic and thermal Enthalpies=           -2713.392127
Sum of electronic and thermal Free Energies=        -2713.473675

                        Standard orientation:

---------------------------------------------------------------------
Center     Atomic      Atomic             Coordinates (Angstroms)
Number     Number       Type             X           Y           Z
---------------------------------------------------------------------

     1          6           0       -0.973951   -1.273819    1.764039
     2          6           0       -0.813147   -1.945585    0.417461
     3          6           0        0.378069   -2.520761   -0.010500
     4          6           0        1.498124   -2.770010    0.970301
     5          6           0        1.456975   -1.820800    2.131125
     6          6           0        0.361050   -1.135778    2.470978
     7          6           0       -1.658675    0.129748    1.772262
     8          8           0       -1.475190    0.826815    2.754337
     9          7           0       -2.565556    0.514129    0.795559
    10          6           0       -1.913198   -2.169464    2.623799
    11          6           0       -2.649111    0.024919   -0.535609
    12          6           0       -1.513552   -0.449143   -1.218457
    13          6           0       -1.629178   -0.912101   -2.531617
    14          6           0       -2.846460   -0.826955   -3.217240
    15          6           0       -3.968612   -0.330348   -2.553089
    16          6           0       -3.874264    0.084384   -1.223176
    17          6           0       -3.451431    1.629970    1.148823
    18         28           0        0.292580   -0.507620   -0.665709
    19          7           0        2.268831   -0.368425   -0.763641
    20          7           0        0.451941    1.460432   -0.508782
    21          6           0        2.729296    0.810923   -0.286310
    22          6           0        1.675730    1.811624   -0.033401
    23          6           0        4.099458    1.064785   -0.180677
    24          6           0        1.901620    3.062484    0.542405
    25          6           0        5.003058    0.105371   -0.637667
    26          6           0        0.857834    3.987489    0.589359
    27          6           0        4.514035   -1.071962   -1.206433
    28          6           0       -0.369637    3.645390    0.024734
    29          6           0        3.135738   -1.272508   -1.238867
```

30	6	0	-0.530994	2.368973	-0.511567
31	1	0	-1.733662	-2.288087	-0.048110
32	1	0	0.321339	-3.185417	-0.880782
33	1	0	2.489118	-2.767341	0.496433
34	1	0	1.375722	-3.810899	1.333822
35	1	0	2.365994	-1.713892	2.729910
36	1	0	0.359670	-0.464236	3.329903
37	1	0	-1.492113	-3.181909	2.707302
38	1	0	-2.014542	-1.744442	3.631690
39	1	0	-2.912848	-2.236551	2.166796
40	1	0	-0.759445	-1.336355	-3.044567
41	1	0	-2.915033	-1.156526	-4.255948
42	1	0	-4.931899	-0.276658	-3.064195
43	1	0	-4.768182	0.442788	-0.712931
44	1	0	-3.535551	2.326300	0.302648
45	1	0	-4.459993	1.270245	1.406568
46	1	0	-3.028072	2.143847	2.016888
47	1	0	4.457581	2.012360	0.220349
48	1	0	2.881282	3.314085	0.947689
49	1	0	6.077596	0.287955	-0.573425
50	1	0	1.010850	4.968111	1.043997
51	1	0	5.184231	-1.830454	-1.612944
52	1	0	-1.202119	4.349623	0.000880
53	1	0	2.709776	-2.186153	-1.655858
54	1	0	-1.469611	2.058522	-0.966988

TS between Intermediates B and C of catalytic cycle for Ni-catalyzed Mizoroki-Heck reaction, bipy ligand, R1=Me, R2=Me, R3=H

Species name: intB_to_intC_ts
Full file name: intB_to_intC_ts.log
Command line: B3LYP/Def2SVP empiricaldispersion=GD3BJ freq geom=check guess=read
Point group: C1
NImag: 1

Thermochemistry:
Temperature: 298.150
Zero-point correction= 0.438642 (Hartree/Particle)
Thermal correction to Energy= 0.463347
Thermal correction to Enthalpy= 0.464292
Thermal correction to Gibbs Free Energy= 0.384821
Sum of electronic and zero-point Energies= -2713.418012
Sum of electronic and thermal Energies= -2713.393307
Sum of electronic and thermal Enthalpies= -2713.392363
Sum of electronic and thermal Free Energies= -2713.471834

Standard orientation:

Center Number	Atomic Number	Atomic Type	Coordinates (Angstroms) X	Y	Z
1	6	0	1.110913	-1.381711	-1.649350
2	6	0	0.905970	-1.929398	-0.244774

3	6	0	-0.346661	-2.446813	0.174825
4	6	0	-1.384295	-2.815348	-0.863686
5	6	0	-1.312555	-1.921531	-2.066169
6	6	0	-0.203750	-1.254721	-2.398820
7	6	0	1.845215	-0.015338	-1.773248
8	8	0	1.782258	0.552251	-2.847986
9	7	0	2.647896	0.485502	-0.757515
10	6	0	2.019876	-2.389341	-2.410968
11	6	0	2.625171	0.095557	0.601982
12	6	0	1.486461	-0.503385	1.176139
13	6	0	1.508333	-0.910938	2.516422
14	6	0	2.603632	-0.623199	3.335634
15	6	0	3.717750	0.007859	2.780319
16	6	0	3.735619	0.351878	1.426870
17	6	0	3.556074	1.572621	-1.134967
18	28	0	-0.358202	-0.491958	0.652821
19	7	0	-2.305594	-0.329957	0.717718
20	7	0	-0.489603	1.491373	0.389588
21	6	0	-2.764440	0.828281	0.186860
22	6	0	-1.713574	1.826163	-0.092234
23	6	0	-4.134197	1.065653	0.044101
24	6	0	-1.944738	3.060331	-0.702571
25	6	0	-5.040692	0.114047	0.511048
26	6	0	-0.902668	3.985456	-0.778605
27	6	0	-4.555191	-1.040752	1.126844
28	6	0	0.328227	3.660836	-0.210444
29	6	0	-3.176763	-1.226930	1.200610
30	6	0	0.491310	2.399936	0.362193
31	1	0	1.796780	-2.378797	0.190710
32	1	0	-0.317518	-3.086447	1.066513
33	1	0	-2.401490	-2.828765	-0.448455
34	1	0	-1.198795	-3.866126	-1.166857
35	1	0	-2.201896	-1.838273	-2.697415
36	1	0	-0.165874	-0.625589	-3.288744
37	1	0	1.560701	-3.388654	-2.399366
38	1	0	2.142420	-2.068680	-3.453963
39	1	0	3.014396	-2.450144	-1.941297
40	1	0	0.655826	-1.454007	2.935837
41	1	0	2.590860	-0.906562	4.389786
42	1	0	4.594832	0.217405	3.395827
43	1	0	4.631623	0.809813	1.010655
44	1	0	3.509725	2.381317	-0.391436
45	1	0	4.595730	1.216028	-1.200917
46	1	0	3.247349	1.948492	-2.114494
47	1	0	-4.490801	1.995315	-0.397962
48	1	0	-2.926064	3.300617	-1.110652
49	1	0	-6.114823	0.284845	0.415661
50	1	0	-1.059556	4.953462	-1.258342
51	1	0	-5.228257	-1.793446	1.539337
52	1	0	1.159244	4.367327	-0.209676
53	1	0	-2.753034	-2.122279	1.656619
54	1	0	1.431151	2.106280	0.826918

Intermediate C of catalytic cycle for Ni-catalyzed Mizoroki-Heck reaction, bipy ligand, R1=Me, R2=Me, R3=H

Species name: intC
Full file name: intC.log
Command line: B3LYP/Def2SVP empiricaldispersion=GD3BJ freq geom=check guess=read
Point group: C1
NImag: 0

Thermochemistry:
Temperature: 298.150
Zero-point correction= 0.441458 (Hartree/Particle)
Thermal correction to Energy= 0.466179
Thermal correction to Enthalpy= 0.467123
Thermal correction to Gibbs Free Energy= 0.387042
Sum of electronic and zero-point Energies= -2713.448896
Sum of electronic and thermal Energies= -2713.424175
Sum of electronic and thermal Enthalpies= -2713.423231
Sum of electronic and thermal Free Energies= -2713.503312

Standard orientation:

Center Number	Atomic Number	Atomic Type	Coordinates (Angstroms) X	Y	Z
1	6	0	2.539217	-1.520672	-0.574788
2	6	0	1.759363	-1.478753	0.773808
3	6	0	0.328126	-2.067627	0.662256
4	6	0	0.043674	-2.911639	-0.574894
5	6	0	0.428594	-2.084734	-1.774945
6	6	0	1.577305	-1.392702	-1.753026
7	6	0	3.569702	-0.398125	-0.768840
8	8	0	4.505925	-0.554087	-1.523248
9	7	0	3.341418	0.835297	-0.160427
10	6	0	3.312628	-2.854855	-0.680480
11	6	0	2.363612	1.040969	0.811740
12	6	0	1.578490	-0.044123	1.295018
13	6	0	0.705468	0.192004	2.387542
14	6	0	0.528070	1.469854	2.930463
15	6	0	1.255857	2.530203	2.399936
16	6	0	2.170744	2.320384	1.363891
17	6	0	4.244968	1.928840	-0.514288
18	28	0	-0.532890	-0.380444	0.306046
19	7	0	-2.405619	-0.921606	0.267540
20	7	0	-1.282179	1.336043	-0.446663
21	6	0	-3.253407	0.011673	-0.242040
22	6	0	-2.608022	1.258446	-0.707747
23	6	0	-4.632949	-0.209024	-0.278315
24	6	0	-3.279028	2.298370	-1.357491
25	6	0	-5.151869	-1.397625	0.233534
26	6	0	-2.562390	3.432859	-1.740677
27	6	0	-4.275104	-2.334387	0.781052
28	6	0	-1.196384	3.499455	-1.464257
29	6	0	-2.910584	-2.054866	0.779322
30	6	0	-0.593987	2.422669	-0.814639
31	1	0	2.350202	-2.039662	1.519670
32	1	0	0.016806	-2.557848	1.597505
33	1	0	-1.016163	-3.197816	-0.620826
34	1	0	0.602804	-3.866764	-0.559554

35	1	0	-0.222374	-2.055852	-2.653305
36	1	0	1.897930	-0.806672	-2.618329
37	1	0	2.634132	-3.710595	-0.581370
38	1	0	3.830989	-2.918344	-1.643694
39	1	0	4.067904	-2.919120	0.117500
40	1	0	0.207279	-0.657237	2.856963
41	1	0	-0.149810	1.619925	3.771973
42	1	0	1.138232	3.536727	2.807316
43	1	0	2.753428	3.164716	1.002730
44	1	0	3.692343	2.755618	-0.986957
45	1	0	4.764104	2.308038	0.378399
46	1	0	4.979780	1.533966	-1.221704
47	1	0	-5.299742	0.547310	-0.690609
48	1	0	-4.344830	2.225890	-1.571223
49	1	0	-6.227506	-1.582947	0.213952
50	1	0	-3.068154	4.254161	-2.252239
51	1	0	-4.634568	-3.271753	1.207681
52	1	0	-0.600505	4.368606	-1.746141
53	1	0	-2.195192	-2.760448	1.201208
54	1	0	0.468546	2.427513	-0.568229

Intermediate B of catalytic cycle for Ni-catalyzed Mizoroki-Heck reaction, R-tBu-(6)CH3iQuinox ligand, R1=Me, R2=Me, R3=H, R configuration for tBu, parent conformation (R)

Species name: intB_tBuMeQ_R
Full file name: intB_tBuMeQ_R.log
Command line: B3LYP/Def2SVP EmpiricalDispersion=GD3BJ freq geom=check guess=read
Point group: C1
NImag: 0

Thermochemistry:
Temperature: 298.150
Zero-point correction= 0.616965 (Hartree/Particle)
Thermal correction to Energy= 0.652707
Thermal correction to Enthalpy= 0.653651
Thermal correction to Gibbs Free Energy= 0.550153
Sum of electronic and zero-point Energies= -3062.231942
Sum of electronic and thermal Energies= -3062.196201
Sum of electronic and thermal Enthalpies= -3062.195257
Sum of electronic and thermal Free Energies= -3062.298754

Standard orientation:

Center Number	Atomic Number	Atomic Type	Coordinates (Angstroms) X	Y	Z
1	6	0	-3.710609	-0.540496	-0.743156
2	6	0	-3.092036	0.508052	0.169544
3	6	0	-2.547301	1.702901	-0.269401
4	6	0	-2.510788	2.042139	-1.739401
5	6	0	-3.444312	1.190786	-2.553414
6	6	0	-3.979557	0.052172	-2.112680
7	6	0	-2.843143	-1.818670	-0.986359
8	8	0	-2.719997	-2.235948	-2.127123
9	7	0	-2.353029	-2.536978	0.087693
10	6	0	-5.031007	-1.026570	-0.099953
11	6	0	-1.979381	-1.961438	1.327319
12	6	0	-1.386427	-0.691102	1.374729
13	6	0	-0.997119	-0.147924	2.601792
14	6	0	-1.125739	-0.889038	3.781958
15	6	0	-1.690380	-2.165331	3.734694
16	6	0	-2.120269	-2.694589	2.517988
17	6	0	-2.006167	-3.944126	-0.129714
18	28	0	-0.977158	0.436971	-0.089152
19	7	0	0.570600	1.874847	0.142963
20	6	0	1.585547	1.139255	0.458251
21	8	0	2.466568	1.635289	1.322927
22	6	0	1.894032	2.877762	1.818173
23	6	0	0.751324	3.179319	0.819043
24	6	0	1.059141	4.334729	-0.178059
25	6	0	-0.090984	4.454839	-1.185091
26	6	0	1.161831	5.641704	0.625978
27	6	0	2.368673	4.071781	-0.938333
28	7	0	0.560015	-0.688299	-0.552534
29	6	0	1.731372	-0.215759	-0.106196
30	6	0	2.951350	-0.926433	-0.251294

31	6	0	2.892124	-2.185792	-0.944194
32	6	0	1.640473	-2.612214	-1.449623
33	6	0	0.509184	-1.855454	-1.240017
34	6	0	4.215648	-0.470291	0.222494
35	6	0	5.339989	-1.235815	0.028670
36	6	0	5.295976	-2.493204	-0.647482
37	6	0	4.078355	-2.942619	-1.121959
38	6	0	6.560981	-3.282127	-0.826783
39	1	0	-3.383568	0.444818	1.216434
40	1	0	-2.425859	2.516440	0.451706
41	1	0	-1.480874	1.952020	-2.145188
42	1	0	-2.747255	3.111534	-1.865117
43	1	0	-3.667261	1.536454	-3.566666
44	1	0	-4.628393	-0.535954	-2.763713
45	1	0	-5.724275	-0.180660	0.019329
46	1	0	-5.514092	-1.780170	-0.738789
47	1	0	-4.845531	-1.473506	0.887987
48	1	0	-0.577353	0.860729	2.650681
49	1	0	-0.790614	-0.466050	4.731481
50	1	0	-1.808472	-2.749622	4.649353
51	1	0	-2.577225	-3.684650	2.489869
52	1	0	-0.982846	-4.142750	0.221517
53	1	0	-2.699848	-4.607869	0.409389
54	1	0	-2.078337	-4.155249	-1.201583
55	1	0	1.539168	2.688247	2.841914
56	1	0	2.690460	3.628823	1.843750
57	1	0	-0.177439	3.432911	1.352662
58	1	0	-0.146370	3.567329	-1.828039
59	1	0	-1.060224	4.578659	-0.676076
60	1	0	0.059505	5.331494	-1.832669
61	1	0	1.984016	5.623336	1.358245
62	1	0	1.350708	6.488297	-0.050851
63	1	0	0.226715	5.851354	1.170056
64	1	0	2.558857	4.887291	-1.652031
65	1	0	3.241641	4.017817	-0.269877
66	1	0	2.316220	3.135538	-1.515234
67	1	0	1.566429	-3.545410	-2.010786
68	1	0	-0.461101	-2.154126	-1.635624
69	1	0	4.283276	0.480492	0.746280
70	1	0	6.301770	-0.875358	0.401998
71	1	0	4.013825	-3.896294	-1.650681
72	1	0	7.309089	-2.696226	-1.385590
73	1	0	7.010360	-3.523184	0.150608
74	1	0	6.384962	-4.222110	-1.366932

Intermediate B of catalytic cycle for Ni-catalyzed Mizoroki-Heck reaction, R-tBu-(6)CH3iQuinox ligand, R1=Me, R2=Me, R3=H, R configuration for tBu, other alkene conformation (RA)

Species name: intB_tBuMeQ_RA
Full file name: intB_tBuMeQ_RA.log
Command line: B3LYP/Def2SVP EmpiricalDispersion=GD3BJ freq geom=check guess=read
Point group: C1
NImag: 0

Thermochemistry:
Temperature: 298.150
Zero-point correction= 0.617763 (Hartree/Particle)
Thermal correction to Energy= 0.653108
Thermal correction to Enthalpy= 0.654052
Thermal correction to Gibbs Free Energy= 0.552106
Sum of electronic and zero-point Energies= -3062.262337
Sum of electronic and thermal Energies= -3062.226993
Sum of electronic and thermal Enthalpies= -3062.226048
Sum of electronic and thermal Free Energies= -3062.327994

Standard orientation:

Center Number	Atomic Number	Atomic Type	Coordinates (Angstroms) X	Y	Z
1	6	0	3.658595	0.432726	0.195151
2	6	0	4.361350	-0.663267	-0.571728
3	6	0	4.119186	-0.953560	-1.852716
4	6	0	3.025246	-0.312841	-2.658392
5	6	0	2.180344	0.643021	-1.867442
6	6	0	2.495961	1.009472	-0.583093
7	6	0	3.062925	0.036974	1.580597
8	8	0	3.006457	0.871256	2.470717
9	7	0	2.565582	-1.229342	1.752321
10	6	0	4.672479	1.577091	0.436539
11	6	0	2.291878	-2.135728	0.678424
12	6	0	1.479805	-1.745704	-0.395182
13	6	0	1.222258	-2.667592	-1.415559
14	6	0	1.758229	-3.958651	-1.373137
15	6	0	2.558592	-4.343011	-0.292809
16	6	0	2.822040	-3.433495	0.729918
17	6	0	1.997997	-1.555946	3.060351
18	28	0	0.678522	-0.046637	-0.446875
19	7	0	-0.442363	1.626671	-0.539022
20	6	0	-1.682785	1.288827	-0.674505
21	8	0	-2.511679	2.208936	-1.146909
22	6	0	-1.674606	3.341558	-1.531696
23	6	0	-0.359750	3.088843	-0.765111
24	6	0	-0.243591	3.897884	0.569881
25	6	0	1.036161	3.522386	1.330302
26	6	0	-0.171460	5.391257	0.203520
27	6	0	-1.453948	3.634100	1.481201
28	7	0	-1.072219	-0.829570	0.063434
29	6	0	-2.122593	-0.054885	-0.262007
30	6	0	-3.470929	-0.480989	-0.131885
31	6	0	-3.689179	-1.794464	0.415476
32	6	0	-2.557599	-2.558638	0.777553
33	6	0	-1.286991	-2.062608	0.579954
34	6	0	-4.610726	0.297291	-0.491794
35	6	0	-5.874865	-0.210872	-0.317136
36	6	0	-6.106075	-1.512636	0.225151
37	6	0	-5.012786	-2.277626	0.581147
38	6	0	-7.513945	-2.006220	0.391731
39	1	0	5.163545	-1.180768	-0.038961
40	1	0	4.718428	-1.715389	-2.357919
41	1	0	2.402108	-1.081668	-3.145079

42	1	0	3.458012	0.265762	-3.499159
43	1	0	1.468013	1.250881	-2.436135
44	1	0	2.076828	1.915363	-0.152057
45	1	0	5.023072	1.970079	-0.528155
46	1	0	4.218435	2.380190	1.030289
47	1	0	5.540900	1.198106	0.995121
48	1	0	0.581027	-2.389003	-2.257550
49	1	0	1.543834	-4.665749	-2.177950
50	1	0	2.983621	-5.347974	-0.251265
51	1	0	3.464837	-3.713423	1.567608
52	1	0	0.917095	-1.330097	3.089102
53	1	0	2.139056	-2.624342	3.267820
54	1	0	2.495107	-0.954425	3.829300
55	1	0	-1.555770	3.302001	-2.624887
56	1	0	-2.204809	4.256495	-1.251166
57	1	0	0.514470	3.320919	-1.391202
58	1	0	1.060051	2.468520	1.638266
59	1	0	1.937864	3.735870	0.735466
60	1	0	1.112041	4.124373	2.247783
61	1	0	-1.087032	5.756635	-0.286404
62	1	0	-0.031871	5.992827	1.113801
63	1	0	0.679698	5.598389	-0.464992
64	1	0	-1.362494	4.231241	2.400367
65	1	0	-2.410719	3.906329	1.010609
66	1	0	-1.504385	2.577461	1.786169
67	1	0	-2.684474	-3.552130	1.211280
68	1	0	-0.401120	-2.643638	0.825828
69	1	0	-4.476257	1.292225	-0.907714
70	1	0	-6.736839	0.398268	-0.600508
71	1	0	-5.153787	-3.276520	0.999962
72	1	0	-8.086582	-1.332284	1.049893
73	1	0	-8.036759	-2.023606	-0.578677
74	1	0	-7.546293	-3.017106	0.819814

Intermediate B of catalytic cycle for Ni-catalyzed Mizoroki-Heck reaction, R-tBu-(6)CH3iQuinox ligand, R1=Me, R2=Me, R3=H, R configuration for tBu, logand flipped conformation (RF)

Species name: intB_tBuMeQ_RF
Full file name: intB_tBuMeQ_RF.log
Command line: B3LYP/Def2SVP EmpiricalDispersion=GD3BJ freq geom=check guess=read
Point group: C1
NImag: 0

Thermochemistry:
Temperature: 298.150
Zero-point correction= 0.618223 (Hartree/Particle)
Thermal correction to Energy= 0.653649
Thermal correction to Enthalpy= 0.654593
Thermal correction to Gibbs Free Energy= 0.552752
Sum of electronic and zero-point Energies= -3062.225028
Sum of electronic and thermal Energies= -3062.189602
Sum of electronic and thermal Enthalpies= -3062.188658
Sum of electronic and thermal Free Energies= -3062.290499

Standard orientation:

Center Number	Atomic Number	Atomic Type	Coordinates (Angstroms) X	Y	Z
1	6	0	-1.795508	-3.069549	-0.138639
2	6	0	-1.779366	-2.071016	-1.276735
3	6	0	-0.717608	-1.862360	-2.111387
4	6	0	0.411943	-2.846224	-2.212701
5	6	0	0.220332	-4.037379	-1.314998
6	6	0	-0.745071	-4.138718	-0.395491
7	6	0	-1.451942	-2.411726	1.246724
8	8	0	-0.653409	-2.973545	1.978289
9	7	0	-2.040083	-1.216385	1.606149
10	6	0	-3.190713	-3.724609	-0.028363
11	6	0	-3.001333	-0.495255	0.808024
12	6	0	-2.538695	0.154502	-0.346385
13	6	0	-3.483404	0.716049	-1.212346
14	6	0	-4.839375	0.731133	-0.869503
15	6	0	-5.273036	0.167804	0.335811
16	6	0	-4.351489	-0.463536	1.174038
17	6	0	-1.717674	-0.646156	2.914794
18	28	0	-0.662138	-0.131951	-0.438173
19	7	0	-0.001679	1.722321	0.007587
20	6	0	1.295941	1.724143	-0.064837
21	8	0	1.923736	2.882325	0.079059
22	6	0	0.897563	3.900238	0.127835
23	6	0	-0.383198	3.114899	0.448271
24	6	0	-1.661554	3.779510	-0.114845
25	6	0	-1.760594	3.528121	-1.625900
26	6	0	-2.879950	3.275008	0.678064
27	6	0	-1.574647	5.302028	0.133861
28	7	0	1.263002	-0.587555	-0.014889
29	6	0	2.073031	0.476956	-0.095975
30	6	0	3.490056	0.385199	-0.015206
31	6	0	4.037787	-0.912459	0.283728
32	6	0	3.136223	-1.979466	0.489381
33	6	0	1.781369	-1.791695	0.315933
34	6	0	4.397898	1.467658	-0.211545
35	6	0	5.752318	1.259275	-0.115818
36	6	0	6.308093	-0.020406	0.192478
37	6	0	5.443557	-1.079087	0.386463
38	6	0	7.797904	-0.176615	0.293991
39	1	0	-2.736123	-1.644206	-1.555431
40	1	0	-0.859156	-1.173756	-2.951786
41	1	0	1.402972	-2.386762	-2.058663
42	1	0	0.436083	-3.179438	-3.268007
43	1	0	0.930196	-4.860791	-1.437689
44	1	0	-0.811105	-5.030374	0.230929
45	1	0	-3.412430	-4.288873	-0.946343
46	1	0	-3.215765	-4.423635	0.820201
47	1	0	-3.975675	-2.969303	0.116059
48	1	0	-3.178234	1.153279	-2.163857
49	1	0	-5.563566	1.189101	-1.547543
50	1	0	-6.330284	0.192740	0.606873
51	1	0	-4.678376	-0.957170	2.092342
52	1	0	-1.461796	0.417380	2.796124

53	1	0	-2.574205	-0.726519	3.602866
54	1	0	-0.869058	-1.196042	3.336425
55	1	0	1.178419	4.634466	0.889892
56	1	0	0.867405	4.382244	-0.860183
57	1	0	-0.502804	3.044691	1.544240
58	1	0	-0.967945	4.064173	-2.171893
59	1	0	-1.663204	2.463171	-1.862691
60	1	0	-2.724877	3.885732	-2.015791
61	1	0	-2.861299	3.697141	1.695497
62	1	0	-3.817172	3.594220	0.199917
63	1	0	-2.903475	2.186920	0.766824
64	1	0	-2.533324	5.768624	-0.137494
65	1	0	-1.389748	5.529290	1.196110
66	1	0	-0.796758	5.793873	-0.469142
67	1	0	3.506439	-2.964398	0.779319
68	1	0	1.081012	-2.607853	0.472324
69	1	0	4.016380	2.456691	-0.448837
70	1	0	6.432325	2.098731	-0.281194
71	1	0	5.833671	-2.071921	0.621574
72	1	0	8.284813	0.110916	-0.652328
73	1	0	8.205493	0.485203	1.075792
74	1	0	8.086576	-1.209403	0.531352

Intermediate B of catalytic cycle for Ni-catalyzed Mizoroki-Heck reaction, R-tBu-(6)CH3iQuinox ligand, R1=Me, R2=Me, R3=H, R configuration for tBu, other twist conformation (RT)

Species name: intB_tBuMeQ_RT
Full file name: intB_tBuMeQ_RT.log
Command line: B3LYP/Def2SVP EmpiricalDispersion=GD3BJ freq geom=check guess=read
Point group: C1
NImag: 0

Thermochemistry:
Temperature: 298.150
Zero-point correction= 0.617771 (Hartree/Particle)
Thermal correction to Energy= 0.653087
Thermal correction to Enthalpy= 0.654032
Thermal correction to Gibbs Free Energy= 0.552119
Sum of electronic and zero-point Energies= -3062.263956
Sum of electronic and thermal Energies= -3062.228639
Sum of electronic and thermal Enthalpies= -3062.227695
Sum of electronic and thermal Free Energies= -3062.329607

Standard orientation:

Center Number	Atomic Number	Atomic Type	Coordinates (Angstroms) X	Y	Z
1	6	0	-2.826181	-0.386550	1.842291
2	6	0	-4.039291	-0.937053	1.129668
3	6	0	-4.628759	-0.347314	0.086694
4	6	0	-4.091277	0.881190	-0.588792

5	6	0	-2.792582	1.365654	-0.007924
6	6	0	-2.244421	0.808956	1.123119
7	6	0	-1.642626	-1.382759	2.033099
8	8	0	-0.897948	-1.264399	2.993115
9	7	0	-1.446813	-2.376330	1.107319
10	6	0	-3.262235	0.084288	3.251056
11	6	0	-1.963866	-2.340315	-0.224492
12	6	0	-1.828983	-1.184150	-1.007052
13	6	0	-2.311303	-1.189955	-2.318840
14	6	0	-2.909539	-2.335458	-2.859082
15	6	0	-3.036819	-3.485182	-2.074809
16	6	0	-2.568299	-3.485679	-0.760695
17	6	0	-0.371701	-3.322319	1.412863
18	28	0	-0.911904	0.340928	-0.419311
19	7	0	0.289994	1.861003	0.077860
20	6	0	1.470029	1.410109	0.348988
21	8	0	2.287601	2.220210	1.005394
22	6	0	1.494434	3.389970	1.370129
23	6	0	0.256002	3.288760	0.452224
24	6	0	0.298614	4.220078	-0.800027
25	6	0	-0.925971	3.954175	-1.687811
26	6	0	1.574043	3.975150	-1.622797
27	6	0	0.251247	5.677961	-0.312514
28	7	0	0.843977	-0.538472	-0.755365
29	6	0	1.863536	0.071365	-0.122579
30	6	0	3.156811	-0.502587	-0.003908
31	6	0	3.365819	-1.776355	-0.641365
32	6	0	2.280738	-2.356293	-1.335071
33	6	0	1.053060	-1.730398	-1.361148
34	6	0	4.249991	0.088548	0.695606
35	6	0	5.461661	-0.556025	0.750989
36	6	0	5.683907	-1.819139	0.120776
37	6	0	4.635880	-2.403641	-0.562722
38	6	0	7.033820	-2.468394	0.219062
39	1	0	-4.475438	-1.841456	1.562096
40	1	0	-5.541342	-0.778741	-0.332920
41	1	0	-3.999687	0.712405	-1.675059
42	1	0	-4.816933	1.713688	-0.498399
43	1	0	-2.454225	2.351347	-0.330635
44	1	0	-1.500542	1.359382	1.705311
45	1	0	-4.012510	0.882320	3.159889
46	1	0	-2.397586	0.440787	3.825240
47	1	0	-3.710769	-0.753522	3.804838
48	1	0	-2.210977	-0.299361	-2.948018
49	1	0	-3.271685	-2.328162	-3.889716
50	1	0	-3.510059	-4.380413	-2.483438
51	1	0	-2.685027	-4.372246	-0.133330
52	1	0	0.615091	-2.834119	1.348620
53	1	0	-0.410419	-4.151296	0.697464
54	1	0	-0.485038	-3.705041	2.435370
55	1	0	2.116450	4.276089	1.208662
56	1	0	1.250827	3.297890	2.438621
57	1	0	-0.665728	3.503778	1.013996
58	1	0	-1.864569	4.179013	-1.156596
59	1	0	-0.957151	2.910118	-2.033102
60	1	0	-0.894006	4.599632	-2.578009
61	1	0	2.492174	4.148909	-1.041487
62	1	0	1.601035	4.659542	-2.483599
63	1	0	1.605772	2.948883	-2.019947

64	1	0	0.218053	6.362654	-1.173051
65	1	0	1.134858	5.952335	0.283994
66	1	0	-0.645353	5.867319	0.299435
67	1	0	2.407660	-3.310503	-1.849518
68	1	0	0.197235	-2.168684	-1.869771
69	1	0	4.120250	1.047777	1.189485
70	1	0	6.287100	-0.090815	1.295546
71	1	0	4.771557	-3.368680	-1.056067
72	1	0	7.303444	-2.645387	1.273258
73	1	0	7.812474	-1.811966	-0.202898
74	1	0	7.066605	-3.429210	-0.311948

Intermediate B of catalytic cycle for Ni-catalyzed Mizoroki-Heck reaction, R-tBu-(6)CH3iQuinox ligand, R1=Me, R2=Me, R3=H, R configuration for tBu, other alkene, ligand flipped conformation (RAF)

Species name: intB_tBuMeQ_RAF
Full file name: intB_tBuMeQ_RAF.log
Command line: B3LYP/Def2SVP EmpiricalDispersion=GD3BJ freq geom=check guess=read
Point group: C1
NImag: 0

Thermochemistry:
Temperature: 298.150
Zero-point correction= 0.618013 (Hartree/Particle)
Thermal correction to Energy= 0.653214
Thermal correction to Enthalpy= 0.654158
Thermal correction to Gibbs Free Energy= 0.552839
Sum of electronic and zero-point Energies= -3062.261542
Sum of electronic and thermal Energies= -3062.226341
Sum of electronic and thermal Enthalpies= -3062.225396
Sum of electronic and thermal Free Energies= -3062.326715

Standard orientation:

Center Number	Atomic Number	Atomic Type	Coordinates (Angstroms) X	Y	Z
1	6	0	-1.942317	-2.711103	0.702820
2	6	0	-3.388210	-2.646707	0.266753
3	6	0	-3.780777	-2.528029	-1.003882
4	6	0	-2.835381	-2.319580	-2.152004
5	6	0	-1.399464	-2.177352	-1.730629
6	6	0	-0.994486	-2.400939	-0.434646
7	6	0	-1.552132	-1.758568	1.875310
8	8	0	-0.740067	-2.117005	2.712173
9	7	0	-2.112778	-0.506540	1.942264
10	6	0	-1.642273	-4.156728	1.167598
11	6	0	-2.898652	0.091229	0.910442
12	6	0	-2.400277	0.184622	-0.396631
13	6	0	-3.214367	0.730480	-1.390199
14	6	0	-4.495017	1.208869	-1.091087
15	6	0	-4.971279	1.145392	0.221106

16	6	0	-4.175998	0.582203	1.218400
17	6	0	-1.784760	0.269806	3.137519
18	28	0	-0.649354	-0.371556	-0.789368
19	7	0	0.064377	1.291366	-0.090643
20	6	0	1.278203	1.123964	0.330891
21	8	0	1.718416	1.976495	1.240312
22	6	0	0.564292	2.793219	1.619630
23	6	0	-0.422978	2.577431	0.456906
24	6	0	-0.396207	3.719411	-0.608023
25	6	0	-1.286828	3.347460	-1.799195
26	6	0	-0.961910	4.987175	0.055043
27	6	0	1.032339	3.979093	-1.114872
28	7	0	1.370906	-0.747081	-1.031882
29	6	0	2.103485	0.041753	-0.232805
30	6	0	3.498697	-0.133068	-0.030677
31	6	0	4.130757	-1.204456	-0.755362
32	6	0	3.325675	-2.000279	-1.602909
33	6	0	1.973910	-1.751419	-1.705049
34	6	0	4.305352	0.671797	0.825031
35	6	0	5.650107	0.415820	0.944343
36	6	0	6.291139	-0.642854	0.230957
37	6	0	5.522607	-1.430692	-0.604312
38	6	0	7.764838	-0.868217	0.406651
39	1	0	-4.131847	-2.782316	1.056490
40	1	0	-4.846345	-2.557569	-1.245593
41	1	0	-3.156138	-1.460971	-2.764636
42	1	0	-2.881185	-3.184795	-2.843076
43	1	0	-0.662152	-2.195185	-2.540026
44	1	0	0.047867	-2.638915	-0.210792
45	1	0	-1.802318	-4.853710	0.333052
46	1	0	-0.613157	-4.241658	1.539226
47	1	0	-2.317744	-4.434716	1.989619
48	1	0	-2.851306	0.806076	-2.418434
49	1	0	-5.113951	1.639600	-1.881611
50	1	0	-5.968665	1.517170	0.464677
51	1	0	-4.552933	0.489477	2.239517
52	1	0	-0.692250	0.352728	3.250215
53	1	0	-2.232794	1.266231	3.048621
54	1	0	-2.162348	-0.220670	4.047256
55	1	0	0.198405	2.402963	2.579653
56	1	0	0.914533	3.822089	1.744337
57	1	0	-1.452496	2.449518	0.814127
58	1	0	-0.940515	2.424494	-2.286168
59	1	0	-2.328927	3.198372	-1.487640
60	1	0	-1.265007	4.153972	-2.547502
61	1	0	-0.342958	5.340158	0.894656
62	1	0	-1.008219	5.806338	-0.677961
63	1	0	-1.983334	4.816958	0.430937
64	1	0	1.020788	4.794667	-1.853042
65	1	0	1.724160	4.278195	-0.313046
66	1	0	1.449106	3.093267	-1.619368
67	1	0	3.772427	-2.815355	-2.175019
68	1	0	1.338407	-2.368971	-2.341075
69	1	0	3.852658	1.485875	1.385297
70	1	0	6.254084	1.040982	1.606668
71	1	0	5.982115	-2.248145	-1.164407
72	1	0	8.332180	0.033983	0.124927
73	1	0	8.002045	-1.076875	1.462813
74	1	0	8.126862	-1.708258	-0.201158

Intermediate B of catalytic cycle for Ni-catalyzed Mizoroki-Heck reaction, R-tBu-(6)CH3iQuinox ligand, R1=Me, R2=Me, R3=H, R configuration for tBu, other alkene, other twist conformation (RAT)

Species name: intB_tBuMeQ_RAT
Full file name: intB_tBuMeQ_RAT.log
Command line: B3LYP/Def2SVP EmpiricalDispersion=GD3BJ freq geom=check guess=read
Point group: C1
NImag: 0

Thermochemistry:
Temperature: 298.150
Zero-point correction= 0.618125 (Hartree/Particle)
Thermal correction to Energy= 0.653504
Thermal correction to Enthalpy= 0.654448
Thermal correction to Gibbs Free Energy= 0.551800
Sum of electronic and zero-point Energies= -3062.246488
Sum of electronic and thermal Energies= -3062.211110
Sum of electronic and thermal Enthalpies= -3062.210166
Sum of electronic and thermal Free Energies= -3062.312814

Standard orientation:

Center Number	Atomic Number	Atomic Type	Coordinates (Angstroms) X	Y	Z
1	6	0	-1.788435	-1.394959	2.079341
2	6	0	-2.624782	-0.524842	1.165995
3	6	0	-2.691878	0.856803	1.270487
4	6	0	-2.178548	1.555513	2.501775
5	6	0	-1.147155	0.750626	3.234537
6	6	0	-0.960400	-0.556517	3.032954
7	6	0	-0.823636	-2.405847	1.379782
8	8	0	0.143204	-2.787908	2.015007
9	7	0	-1.139954	-2.983943	0.160143
10	6	0	-2.762364	-2.288367	2.900183
11	6	0	-1.975461	-2.437380	-0.851060
12	6	0	-2.071962	-1.045389	-1.051117
13	6	0	-2.880965	-0.550225	-2.077881
14	6	0	-3.533550	-1.417003	-2.963188
15	6	0	-3.421402	-2.794963	-2.778109
16	6	0	-2.654920	-3.301363	-1.726345
17	6	0	-0.435394	-4.231087	-0.158097
18	28	0	-1.160126	0.320785	-0.106116
19	7	0	-0.107437	1.956350	0.294214
20	6	0	1.159226	1.708306	0.267938
21	8	0	1.982774	2.720220	0.500994
22	6	0	1.141823	3.872662	0.795299
23	6	0	-0.272753	3.422989	0.364592
24	6	0	-0.789758	4.001852	-0.985221
25	6	0	-2.195275	3.440752	-1.247474
26	6	0	0.142390	3.615394	-2.143778

27	6	0	-0.878396	5.531177	-0.855878
28	7	0	0.637274	-0.299735	-0.762901
29	6	0	1.636453	0.397140	-0.191987
30	6	0	2.980734	-0.053398	-0.159888
31	6	0	3.269666	-1.292194	-0.830596
32	6	0	2.206595	-1.947106	-1.492291
33	6	0	0.927583	-1.437940	-1.430790
34	6	0	4.045888	0.625544	0.500121
35	6	0	5.311758	0.092536	0.489232
36	6	0	5.615208	-1.135025	-0.176663
37	6	0	4.593372	-1.801880	-0.824024
38	6	0	7.021072	-1.661623	-0.152171
39	1	0	-3.438464	-1.030690	0.653535
40	1	0	-3.486262	1.360936	0.710366
41	1	0	-1.813390	2.567593	2.289461
42	1	0	-3.057144	1.719097	3.159233
43	1	0	-0.542998	1.269395	3.984645
44	1	0	-0.204075	-1.107792	3.591892
45	1	0	-3.464314	-1.656800	3.464001
46	1	0	-2.196327	-2.906672	3.610615
47	1	0	-3.336532	-2.951839	2.234921
48	1	0	-2.999574	0.528633	-2.212568
49	1	0	-4.131022	-1.012892	-3.783021
50	1	0	-3.941555	-3.485348	-3.445122
51	1	0	-2.596961	-4.379993	-1.578450
52	1	0	-0.158407	-4.244596	-1.220669
53	1	0	-1.066889	-5.109475	0.050926
54	1	0	0.463250	-4.287468	0.463909
55	1	0	1.542366	4.724693	0.235451
56	1	0	1.219001	4.071420	1.873930
57	1	0	-1.003790	3.690576	1.137369
58	1	0	-2.896968	3.723089	-0.446640
59	1	0	-2.177159	2.343264	-1.311147
60	1	0	-2.594226	3.827276	-2.196985
61	1	0	1.176641	3.955651	-1.978832
62	1	0	-0.206605	4.076158	-3.079818
63	1	0	0.159188	2.525779	-2.298561
64	1	0	-1.326336	5.960771	-1.764343
65	1	0	0.109202	6.000985	-0.730220
66	1	0	-1.509076	5.826682	-0.001831
67	1	0	2.394593	-2.863638	-2.054263
68	1	0	0.093782	-1.919259	-1.936574
69	1	0	3.851931	1.562983	1.016234
70	1	0	6.117065	0.619925	1.006581
71	1	0	4.792809	-2.742960	-1.341413
72	1	0	7.353162	-1.833501	0.884912
73	1	0	7.718322	-0.930468	-0.593180
74	1	0	7.114879	-2.605807	-0.705348

Intermediate B of catalytic cycle for Ni-catalyzed Mizoroki-Heck reaction, R-tBu-(6)CH3iQuinox ligand, R1=Me, R2=Me, R3=H, R configuration for tBu, ligand flipped, other twist conformation (RFT)

Species name: intB_tBuMeQ_RFT
Full file name: intB_tBuMeQ_RFT.log

Command line: B3LYP/Def2SVP EmpiricalDispersion=GD3BJ freq geom=check guess=read
Point group: C1
NImag: 0

Thermochemistry:
Temperature: 298.150
Zero-point correction= 0.618402 (Hartree/Particle)
Thermal correction to Energy= 0.653463
Thermal correction to Enthalpy= 0.654407
Thermal correction to Gibbs Free Energy= 0.554037
Sum of electronic and zero-point Energies= -3062.260872
Sum of electronic and thermal Energies= -3062.225811
Sum of electronic and thermal Enthalpies= -3062.224867
Sum of electronic and thermal Free Energies= -3062.325237

Standard orientation:

Center Number	Atomic Number	Atomic Type	Coordinates (Angstroms) X	Y	Z
1	6	0	2.765159	-2.448128	0.579187
2	6	0	3.730780	-2.349543	-0.580802
3	6	0	3.365335	-2.380528	-1.863721
4	6	0	1.934126	-2.418058	-2.311594
5	6	0	0.944391	-2.327126	-1.185774
6	6	0	1.325605	-2.320288	0.137286
7	6	0	3.031019	-1.410911	1.703366
8	8	0	3.000446	-1.747344	2.874330
9	7	0	3.325257	-0.114905	1.354766
10	6	0	2.911104	-3.855796	1.208090
11	6	0	3.273734	0.411695	0.029755
12	6	0	2.121096	0.318468	-0.768265
13	6	0	2.158475	0.842092	-2.065964
14	6	0	3.298786	1.476087	-2.569916
15	6	0	4.431477	1.590545	-1.760893
16	6	0	4.415975	1.056595	-0.474282
17	6	0	3.817786	0.721453	2.454930
18	28	0	0.476364	-0.453276	-0.270400
19	7	0	-0.414166	1.281674	-0.280465
20	6	0	-1.693958	1.177658	-0.448706
21	8	0	-2.334736	2.257363	-0.865119
22	6	0	-1.303105	3.245347	-1.176484
23	6	0	-0.062977	2.714410	-0.434887
24	6	0	0.203277	3.405239	0.936266
25	6	0	1.397422	2.727111	1.612109
26	6	0	-1.023246	3.318049	1.858294
27	6	0	0.558738	4.876826	0.662795
28	7	0	-1.492932	-1.070824	0.081224
29	6	0	-2.378300	-0.095009	-0.165697
30	6	0	-3.786811	-0.282261	-0.109729
31	6	0	-4.254641	-1.596725	0.244191
32	6	0	-3.289606	-2.596727	0.500855
33	6	0	-1.946718	-2.302179	0.406064
34	6	0	-4.757111	0.730449	-0.365447
35	6	0	-6.097132	0.440233	-0.274250
36	6	0	-6.574785	-0.860019	0.074677

37	6	0	-5.647279	-1.852066	0.327449
38	6	0	-8.052707	-1.109177	0.157904
39	1	0	4.791019	-2.318455	-0.315993
40	1	0	4.127456	-2.363304	-2.647014
41	1	0	1.741100	-1.630334	-3.060399
42	1	0	1.725385	-3.365253	-2.847787
43	1	0	-0.094189	-2.539534	-1.454270
44	1	0	0.610150	-2.543721	0.933964
45	1	0	2.658124	-4.621772	0.461474
46	1	0	2.266295	-3.959816	2.090383
47	1	0	3.947731	-4.013669	1.537886
48	1	0	1.272853	0.772985	-2.706378
49	1	0	3.298325	1.881870	-3.584218
50	1	0	5.332369	2.080923	-2.135458
51	1	0	5.310654	1.113006	0.149164
52	1	0	3.069387	0.771964	3.257469
53	1	0	4.020854	1.728464	2.079959
54	1	0	4.735651	0.294988	2.886915
55	1	0	-1.664056	4.219937	-0.834061
56	1	0	-1.186614	3.255206	-2.270445
57	1	0	0.840452	2.797344	-1.051209
58	1	0	2.295303	2.802669	0.983912
59	1	0	1.205521	1.661894	1.796844
60	1	0	1.612300	3.207094	2.578495
61	1	0	-1.915788	3.795645	1.425706
62	1	0	-0.811146	3.828677	2.809391
63	1	0	-1.271826	2.273103	2.100775
64	1	0	0.827601	5.378175	1.604549
65	1	0	-0.277636	5.442946	0.224153
66	1	0	1.421590	4.958561	-0.017337
67	1	0	-3.604692	-3.604901	0.775823
68	1	0	-1.197814	-3.071700	0.594336
69	1	0	-4.434445	1.732855	-0.633472
70	1	0	-6.826600	1.229229	-0.473523
71	1	0	-5.977742	-2.857103	0.599084
72	1	0	-8.537754	-0.894458	-0.808466
73	1	0	-8.517404	-0.442323	0.902629
74	1	0	-8.278854	-2.147529	0.435090

Intermediate B of catalytic cycle for Ni-catalyzed Mizoroki-Heck reaction, R-tBu-(6)CH3iQuinox ligand, R1=Me, R2=Me, R3=H, R configuration for tBu, other alkene, ligand flipped, other twist conformation (RAFT)

Species name: intB_tBuMeQ_RAFT
Full file name: intB_tBuMeQ_RAFT.log
Command line: B3LYP/Def2SVP EmpiricalDispersion=GD3BJ freq geom=check guess=read
Point group: C1
NImag: 0

Thermochemistry:
Temperature: 298.150
Zero-point correction= 0.618776 (Hartree/Particle)
Thermal correction to Energy= 0.653821
Thermal correction to Enthalpy= 0.654766
Thermal correction to Gibbs Free Energy= 0.554605

Sum of electronic and zero-point Energies= -3062.246770
Sum of electronic and thermal Energies= -3062.211725
Sum of electronic and thermal Enthalpies= -3062.210781
Sum of electronic and thermal Free Energies= -3062.310942

Standard orientation:

Center Number	Atomic Number	Atomic Type	Coordinates (Angstroms) X	Y	Z
1	6	0	2.312762	-2.836714	-0.031748
2	6	0	2.078146	-2.008447	-1.277868
3	6	0	0.962843	-2.098017	-2.058105
4	6	0	-0.020495	-3.223111	-1.933533
5	6	0	0.373271	-4.217592	-0.878355
6	6	0	1.400299	-4.050836	-0.039859
7	6	0	1.983265	-2.041676	1.278052
8	8	0	1.221171	-2.534190	2.097779
9	7	0	2.601099	-0.841868	1.538715
10	6	0	3.791584	-3.277485	0.032720
11	6	0	3.233690	0.006503	0.563285
12	6	0	2.493546	0.456729	-0.545467
13	6	0	3.122176	1.276526	-1.485238
14	6	0	4.446114	1.696236	-1.298240
15	6	0	5.160126	1.276252	-0.172788
16	6	0	4.556618	0.420935	0.752967
17	6	0	2.385016	-0.307361	2.889102
18	28	0	0.722455	-0.198374	-0.555497
19	7	0	-0.096379	1.542656	-0.526322
20	6	0	-1.380142	1.450014	-0.698636
21	8	0	-2.004918	2.530756	-1.137105
22	6	0	-0.956077	3.495714	-1.464708
23	6	0	0.277371	2.963884	-0.706872
24	6	0	0.552394	3.685628	0.649038
25	6	0	1.698527	2.986845	1.390688
26	6	0	-0.697025	3.678706	1.545105
27	6	0	0.971922	5.130769	0.330671
28	7	0	-1.158452	-0.761000	-0.071188
29	6	0	-2.059773	0.197557	-0.336972
30	6	0	-3.454001	0.028224	-0.138572
31	6	0	-3.877617	-1.222303	0.435443
32	6	0	-2.884741	-2.170308	0.774879
33	6	0	-1.555169	-1.917523	0.505913
34	6	0	-4.444802	1.003735	-0.451548
35	6	0	-5.771337	0.735577	-0.215704
36	6	0	-6.209115	-0.501941	0.349594
37	6	0	-5.258939	-1.453350	0.665495
38	6	0	-7.674457	-0.729980	0.585281
39	1	0	2.924168	-1.439498	-1.652972
40	1	0	0.930830	-1.502910	-2.977403
41	1	0	-1.053895	-2.866877	-1.780988
42	1	0	-0.054984	-3.720660	-2.921974
43	1	0	-0.228820	-5.128707	-0.813891
44	1	0	1.620120	-4.812930	0.710393
45	1	0	4.030793	-3.916572	-0.830221
46	1	0	3.979684	-3.852937	0.950928
47	1	0	4.464620	-2.408317	0.021530

48	1	0	2.584023	1.599745	-2.381328
49	1	0	4.920431	2.348453	-2.035397
50	1	0	6.192296	1.598958	-0.023017
51	1	0	5.111562	0.066946	1.625164
52	1	0	1.331743	-0.020283	3.036204
53	1	0	3.023425	0.571844	3.026241
54	1	0	2.628252	-1.070223	3.640747
55	1	0	-1.302825	4.483350	-1.145688
56	1	0	-0.834083	3.480399	-2.557990
57	1	0	1.183069	3.026819	-1.321439
58	1	0	2.620694	2.966389	0.793532
59	1	0	1.432672	1.952487	1.636454
60	1	0	1.910718	3.517166	2.331456
61	1	0	-1.556911	4.188667	1.084814
62	1	0	-0.479822	4.198872	2.489859
63	1	0	-1.000612	2.652003	1.803094
64	1	0	1.214913	5.664873	1.261328
65	1	0	0.177520	5.703890	-0.172541
66	1	0	1.867829	5.152439	-0.310102
67	1	0	-3.166093	-3.109199	1.255097
68	1	0	-0.770723	-2.626441	0.772574
69	1	0	-4.146155	1.956144	-0.883683
70	1	0	-6.520861	1.490455	-0.466198
71	1	0	-5.561498	-2.406935	1.104053
72	1	0	-8.236983	-0.652903	-0.359673
73	1	0	-8.083362	0.037386	1.262943
74	1	0	-7.870275	-1.717598	1.024099

TS between Intermediates B and C of catalytic cycle for Ni-catalyzed Mizoroki-Heck reaction, R-tBu-(6)CH3iQuinox ligand, R1=Me, R2=Me, R3=H, R configuration for tBu, parent conformation (R)

Species name: intB_to_intC_ts_tBuMeQ_R
Full file name: intB_to_intC_ts_tBuMeQ_R.log
Command line: B3LYP/Def2SVP empiricaldispersion=GD3BJ freq geom=check guess=read
Point group: C1
NImag: 1

Thermochemistry:
Temperature: 298.150
Zero-point correction= 0.617670 (Hartree/Particle)
Thermal correction to Energy= 0.652311
Thermal correction to Enthalpy= 0.653255
Thermal correction to Gibbs Free Energy= 0.552715
Sum of electronic and zero-point Energies= -3062.227568
Sum of electronic and thermal Energies= -3062.192926
Sum of electronic and thermal Enthalpies= -3062.191982
Sum of electronic and thermal Free Energies= -3062.292522

Standard orientation:

Center Number	Atomic Number	Atomic Type	Coordinates (Angstroms) X	Y	Z

1	6	0	0.530836	-2.011929	1.959066
2	6	0	1.750288	-1.247757	1.468300
3	6	0	1.909894	0.134701	1.732967
4	6	0	0.934365	0.860145	2.635907
5	6	0	-0.414377	0.204961	2.669225
6	6	0	-0.601493	-1.075132	2.342072
7	6	0	-0.083487	-3.093521	1.032370
8	8	0	-1.124407	-3.602647	1.410667
9	7	0	0.530715	-3.534494	-0.128314
10	6	0	0.979580	-2.787214	3.233467
11	6	0	1.618900	-2.930556	-0.803118
12	6	0	1.980714	-1.582437	-0.619545
13	6	0	3.126558	-1.071597	-1.248012
14	6	0	3.838976	-1.830021	-2.179503
15	6	0	3.446508	-3.147630	-2.414754
16	6	0	2.363813	-3.692945	-1.725461
17	6	0	-0.072125	-4.717933	-0.752383
18	28	0	1.122167	0.035172	-0.102847
19	7	0	0.661340	1.951084	-0.293334
20	6	0	-0.612604	2.069438	-0.483797
21	8	0	-1.084290	3.292357	-0.705607
22	6	0	0.050934	4.193449	-0.565343
23	6	0	1.265190	3.257599	-0.690858
24	6	0	2.575757	3.744421	-0.034685
25	6	0	2.483472	3.920800	1.486803
26	6	0	3.695871	2.748527	-0.369042
27	6	0	2.906902	5.106410	-0.678669
28	7	0	-0.705602	-0.189899	-0.999927
29	6	0	-1.452944	0.874286	-0.661217
30	6	0	-2.867343	0.833143	-0.560028
31	6	0	-3.505186	-0.419532	-0.860467
32	6	0	-2.688305	-1.495441	-1.280158
33	6	0	-1.319730	-1.342961	-1.342249
34	6	0	-3.679664	1.929773	-0.152705
35	6	0	-5.041194	1.775867	-0.048565
36	6	0	-5.690521	0.537844	-0.343428
37	6	0	-4.914767	-0.532929	-0.743757
38	6	0	-7.183196	0.435412	-0.211913
39	1	0	2.663367	-1.841302	1.430556
40	1	0	2.936658	0.515413	1.732669
41	1	0	0.833334	1.912249	2.343494
42	1	0	1.363521	0.885728	3.658627
43	1	0	-1.265969	0.807510	2.998482
44	1	0	-1.586945	-1.538526	2.401138
45	1	0	1.390090	-2.083754	3.972099
46	1	0	0.118679	-3.306083	3.673990
47	1	0	1.751781	-3.532733	2.985730
48	1	0	3.470507	-0.059431	-1.018089
49	1	0	4.702456	-1.401584	-2.691816
50	1	0	4.000791	-3.771987	-3.118213
51	1	0	2.108658	-4.736519	-1.898273
52	1	0	-0.210083	-4.547687	-1.830112
53	1	0	0.562173	-5.606636	-0.613447
54	1	0	-1.038077	-4.897565	-0.273121
55	1	0	-0.021101	4.947452	-1.355857
56	1	0	-0.024394	4.676407	0.420847
57	1	0	1.502055	3.136318	-1.764524
58	1	0	1.626146	4.540151	1.791740

59	1	0	2.411705	2.957883	2.004453
60	1	0	3.390439	4.422689	1.855654
61	1	0	3.824911	2.639190	-1.457493
62	1	0	4.653278	3.089608	0.052211
63	1	0	3.475798	1.758830	0.050788
64	1	0	3.897760	5.448994	-0.345891
65	1	0	2.928841	5.041267	-1.778379
66	1	0	2.181195	5.884689	-0.394158
67	1	0	-3.139868	-2.450278	-1.554545
68	1	0	-0.669965	-2.146757	-1.677168
69	1	0	-3.217733	2.888892	0.073354
70	1	0	-5.652069	2.624555	0.269276
71	1	0	-5.381766	-1.492641	-0.976227
72	1	0	-7.500514	0.668732	0.817709
73	1	0	-7.683037	1.162879	-0.872396
74	1	0	-7.548628	-0.568729	-0.465958

TS between Intermediates B and C of catalytic cycle for Ni-catalyzed Mizoroki-Heck reaction, R-tBu-(6)CH3iQuinox ligand, R1=Me, R2=Me, R3=H, R configuration for tBu, other alkene conformation (RA)

Species name: intB_to_intC_ts_tBuMeQ_RA
Full file name: intB_to_intC_ts_tBuMeQ_RA.log
Command line: B3LYP/Def2SVP empiricaldispersion=GD3BJ freq geom=check guess=read
Point group: C1
NImag: 1

Thermochemistry:
Temperature: 298.150
Zero-point correction= 0.617176 (Hartree/Particle)
Thermal correction to Energy= 0.651562
Thermal correction to Enthalpy= 0.652506
Thermal correction to Gibbs Free Energy= 0.552899
Sum of electronic and zero-point Energies= -3062.222641
Sum of electronic and thermal Energies= -3062.188255
Sum of electronic and thermal Enthalpies= -3062.187311
Sum of electronic and thermal Free Energies= -3062.286918

Standard orientation:

Center Number	Atomic Number	Atomic Type	Coordinates (Angstroms) X	Y	Z
1	6	0	-4.024883	-0.003179	-0.234372
2	6	0	-4.472824	-0.336434	1.173510
3	6	0	-3.934670	0.276442	2.234129
4	6	0	-2.842263	1.318671	2.160524
5	6	0	-2.165392	1.437361	0.802794
6	6	0	-2.598709	0.524799	-0.208151
7	6	0	-4.140165	-1.154812	-1.254306
8	8	0	-5.086046	-1.251347	-2.006718
9	7	0	-3.069367	-2.041655	-1.337625
10	6	0	-4.906234	1.151606	-0.773907

11	6	0	-2.265572	-2.263637	-0.196824
12	6	0	-1.715298	-1.183467	0.513928
13	6	0	-1.196194	-1.415627	1.802028
14	6	0	-1.024384	-2.713910	2.286768
15	6	0	-1.458885	-3.795162	1.517298
16	6	0	-2.109128	-3.569070	0.304629
17	6	0	-3.180220	-3.083666	-2.355156
18	28	0	-0.568889	0.396408	0.257574
19	7	0	0.742448	1.809734	0.386355
20	6	0	1.957919	1.362015	0.409865
21	8	0	2.905823	2.227437	0.744224
22	6	0	2.213258	3.445693	1.150977
23	6	0	0.808112	3.279685	0.538032
24	6	0	0.618664	4.027899	-0.822286
25	6	0	-0.750070	3.692686	-1.432091
26	6	0	0.677152	5.539364	-0.541738
27	6	0	1.712773	3.633114	-1.828694
28	7	0	1.088242	-0.675141	-0.259707
29	6	0	2.228800	-0.035933	0.041422
30	6	0	3.508015	-0.652786	-0.025187
31	6	0	3.543538	-2.024699	-0.457779
32	6	0	2.317638	-2.656481	-0.770091
33	6	0	1.129855	-1.966853	-0.649857
34	6	0	4.740356	-0.015148	0.299799
35	6	0	5.923302	-0.706738	0.194896
36	6	0	5.974706	-2.067469	-0.236213
37	6	0	4.788366	-2.699436	-0.553543
38	6	0	7.301007	-2.765383	-0.331259
39	1	0	-5.292559	-1.049129	1.294283
40	1	0	-4.314242	0.045513	3.233848
41	1	0	-2.108470	1.138625	2.967907
42	1	0	-3.280997	2.301650	2.415736
43	1	0	-1.900635	2.446533	0.491119
44	1	0	-2.262987	0.746320	-1.232183
45	1	0	-4.850009	2.012429	-0.094354
46	1	0	-4.586413	1.458928	-1.780979
47	1	0	-5.947518	0.813596	-0.845147
48	1	0	-0.875792	-0.580877	2.431392
49	1	0	-0.569737	-2.875000	3.266265
50	1	0	-1.343936	-4.816511	1.886178
51	1	0	-2.534925	-4.410208	-0.242261
52	1	0	-2.187931	-3.518286	-2.532982
53	1	0	-3.883945	-3.880975	-2.063928
54	1	0	-3.560918	-2.632099	-3.278328
55	1	0	2.205005	3.465568	2.251035
56	1	0	2.786144	4.297099	0.770958
57	1	0	0.029341	3.614537	1.237370
58	1	0	-0.851217	2.614600	-1.620545
59	1	0	-1.576734	4.017131	-0.781749
60	1	0	-0.868937	4.212888	-2.394025
61	1	0	1.657792	5.861226	-0.158873
62	1	0	0.492864	6.101907	-1.469093
63	1	0	-0.089954	5.840962	0.189464
64	1	0	1.571908	4.188930	-2.767480
65	1	0	2.726543	3.859464	-1.466400
66	1	0	1.665620	2.561173	-2.075772
67	1	0	2.308692	-3.696123	-1.102419
68	1	0	0.169904	-2.438628	-0.854809
69	1	0	4.741425	1.020542	0.630050

70	1	0	6.859212	-0.202255	0.447935
71	1	0	4.792485	-3.740076	-0.885756
72	1	0	7.969524	-2.236941	-1.030600
73	1	0	7.807120	-2.774465	0.648041
74	1	0	7.194252	-3.803400	-0.674077

TS between Intermediates B and C of catalytic cycle for Ni-catalyzed Mizoroki-Heck reaction, R-tBu-(6)CH3iQuinox ligand, R1=Me, R2=Me, R3=H, R configuration for tBu, flipped ligand conformation (RF)

Species name: intB_to_intC_ts_tBuMeQ_RF
Full file name: intB_to_intC_ts_tBuMeQ_RF.log
Command line: B3LYP/Def2SVP empiricaldispersion=GD3BJ freq geom=check guess=read
Point group: C1
NImag: 1

Thermochemistry:
Temperature: 298.150
Zero-point correction= 0.618083 (Hartree/Particle)
Thermal correction to Energy= 0.652585
Thermal correction to Enthalpy= 0.653529
Thermal correction to Gibbs Free Energy= 0.554238
Sum of electronic and zero-point Energies= -3062.223794
Sum of electronic and thermal Energies= -3062.189292
Sum of electronic and thermal Enthalpies= -3062.188348
Sum of electronic and thermal Free Energies= -3062.287639

Standard orientation:

Center Number	Atomic Number	Atomic Type	Coordinates (Angstroms) X	Y	Z
1	6	0	3.167804	-1.832210	-0.543385
2	6	0	1.941636	-2.169989	0.270898
3	6	0	0.791888	-2.701144	-0.335389
4	6	0	0.752914	-2.993212	-1.818748
5	6	0	1.724076	-2.128295	-2.575558
6	6	0	2.805627	-1.593217	-1.999594
7	6	0	4.037001	-0.637072	-0.087229
8	8	0	5.039585	-0.421584	-0.745436
9	7	0	3.734772	0.144859	1.013249
10	6	0	4.100051	-3.077542	-0.459218
11	6	0	2.578663	0.088165	1.829505
12	6	0	1.331995	-0.405055	1.397219
13	6	0	0.275711	-0.507750	2.319615
14	6	0	0.379049	0.002996	3.613816
15	6	0	1.594667	0.549837	4.025199
16	6	0	2.678755	0.570270	3.152522
17	6	0	4.753789	1.143848	1.359798
18	28	0	0.346961	-0.768837	-0.228457
19	7	0	-0.248702	1.115075	-0.754602
20	6	0	-1.537469	1.187281	-0.733232
21	8	0	-2.100868	2.330796	-1.114018

22	6	0	-1.011475	3.267943	-1.327099
23	6	0	0.222481	2.358925	-1.434815
24	6	0	1.545772	2.972321	-0.945273
25	6	0	1.533313	3.175163	0.575492
26	6	0	2.666073	2.019665	-1.374946
27	6	0	1.740810	4.326438	-1.651349
28	7	0	-1.667085	-1.130860	-0.557473
29	6	0	-2.371445	0.006728	-0.452694
30	6	0	-3.762919	0.042880	-0.153297
31	6	0	-4.432475	-1.219869	-0.000334
32	6	0	-3.664081	-2.396649	-0.156950
33	6	0	-2.314606	-2.313213	-0.414943
34	6	0	-4.527556	1.232987	0.022193
35	6	0	-5.867093	1.156258	0.320176
36	6	0	-6.546144	-0.091643	0.461330
37	6	0	-5.817854	-1.254700	0.299739
38	6	0	-8.013322	-0.106551	0.780041
39	1	0	2.139212	-2.471448	1.298384
40	1	0	0.139449	-3.296077	0.310739
41	1	0	-0.265597	-2.874305	-2.221136
42	1	0	0.999631	-4.063180	-1.978284
43	1	0	1.544792	-1.973376	-3.643077
44	1	0	3.525765	-1.011528	-2.577109
45	1	0	3.571430	-3.968962	-0.825596
46	1	0	4.990732	-2.906359	-1.076269
47	1	0	4.421159	-3.255555	0.579155
48	1	0	-0.664698	-0.983844	2.026359
49	1	0	-0.473027	-0.048833	4.294489
50	1	0	1.715048	0.935162	5.039627
51	1	0	3.629677	0.954925	3.514743
52	1	0	4.267365	2.086643	1.638295
53	1	0	5.383698	0.800713	2.195330
54	1	0	5.394530	1.291876	0.486564
55	1	0	-1.229296	3.841099	-2.234484
56	1	0	-0.978007	3.942506	-0.458508
57	1	0	0.371198	2.075852	-2.493000
58	1	0	0.754940	3.888843	0.887736
59	1	0	1.362140	2.232481	1.108898
60	1	0	2.497087	3.588593	0.908516
61	1	0	2.744678	1.977945	-2.472279
62	1	0	3.640865	2.334965	-0.980013
63	1	0	2.460241	1.006112	-1.018602
64	1	0	2.735693	4.731836	-1.413169
65	1	0	1.679314	4.223859	-2.746910
66	1	0	0.999797	5.076141	-1.331784
67	1	0	-4.138703	-3.375785	-0.069657
68	1	0	-1.720052	-3.216921	-0.532596
69	1	0	-4.048750	2.202767	-0.083684
70	1	0	-6.436031	2.079810	0.453451
71	1	0	-6.304418	-2.226926	0.406015
72	1	0	-8.211343	0.421507	1.727209
73	1	0	-8.586036	0.417571	-0.002635
74	1	0	-8.403841	-1.129462	0.866535

TS between Intermediates B and C of catalytic cycle for Ni-catalyzed Mizoroki-Heck reaction, R-tBu-(6)CH3iQuinox ligand, R1=Me, R2=Me, R3=H, R configuration for tBu, other twist conformation (RT)

Species name: intB_to_intC_ts_tBuMeQ_RT
Full file name: intB_to_intC_ts_tBuMeQ_RT.log
Command line: B3LYP/Def2SVP empiricaldispersion=GD3BJ freq geom=check guess=read
Point group: C1
NImag: 1

Thermochemistry:
Temperature: 298.150
Zero-point correction= 0.616990 (Hartree/Particle)
Thermal correction to Energy= 0.651531
Thermal correction to Enthalpy= 0.652475
Thermal correction to Gibbs Free Energy= 0.552563
Sum of electronic and zero-point Energies= -3062.226549
Sum of electronic and thermal Energies= -3062.192008
Sum of electronic and thermal Enthalpies= -3062.191064
Sum of electronic and thermal Free Energies= -3062.290976

Standard orientation:

Center Number	Atomic Number	Atomic Type	Coordinates (Angstroms) X	Y	Z
1	6	0	3.662531	-0.495957	-1.292024
2	6	0	4.633229	-0.033945	-0.226035
3	6	0	4.461975	1.133542	0.403491
4	6	0	3.309057	2.080775	0.165449
5	6	0	2.166669	1.507807	-0.661616
6	6	0	2.299171	0.136524	-1.056592
7	6	0	3.518139	-2.025891	-1.424961
8	8	0	4.103352	-2.651337	-2.283546
9	7	0	2.629816	-2.660687	-0.562200
10	6	0	4.154298	0.023273	-2.666235
11	6	0	2.317054	-2.073182	0.682374
12	6	0	1.921641	-0.726816	0.761240
13	6	0	1.877239	-0.101590	2.020474
14	6	0	2.022581	-0.842883	3.197071
15	6	0	2.309081	-2.206072	3.115171
16	6	0	2.491535	-2.807089	1.868851
17	6	0	2.485306	-4.103784	-0.734324
18	28	0	0.587923	0.518641	0.044878
19	7	0	-0.782974	1.801224	-0.420715
20	6	0	-1.943421	1.254786	-0.598629
21	8	0	-2.895807	2.022138	-1.110906
22	6	0	-2.260609	3.284782	-1.465102
23	6	0	-0.909484	3.243621	-0.715894
24	6	0	-0.852999	4.118961	0.572332
25	6	0	0.519660	3.950814	1.236162
26	6	0	-1.951987	3.705807	1.563706
27	6	0	-1.027860	5.591201	0.163006
28	7	0	-1.026920	-0.686967	0.267745
29	6	0	-2.161857	-0.143995	-0.199632
30	6	0	-3.398211	-0.844722	-0.245833

31	6	0	-3.398918	-2.192181	0.259415
32	6	0	-2.186007	-2.713941	0.765094
33	6	0	-1.039616	-1.947783	0.751956
34	6	0	-4.621204	-0.312208	-0.747769
35	6	0	-5.760756	-1.080333	-0.744901
36	6	0	-5.776456	-2.419018	-0.247197
37	6	0	-4.599607	-2.948483	0.244821
38	6	0	-7.056244	-3.204194	-0.271776
39	1	0	5.514625	-0.651045	-0.034222
40	1	0	5.209097	1.461783	1.132251
41	1	0	2.956439	2.465081	1.138336
42	1	0	3.695960	2.980179	-0.349346
43	1	0	1.699097	2.193351	-1.373433
44	1	0	1.595139	-0.207989	-1.828867
45	1	0	4.256294	1.116293	-2.636623
46	1	0	3.458542	-0.258603	-3.470709
47	1	0	5.128471	-0.424377	-2.899084
48	1	0	1.701752	0.974165	2.104017
49	1	0	1.934347	-0.349368	4.166841
50	1	0	2.443935	-2.796637	4.023668
51	1	0	2.804975	-3.849590	1.815455
52	1	0	2.452408	-4.325723	-1.806967
53	1	0	1.553518	-4.430591	-0.254519
54	1	0	3.333786	-4.663609	-0.307198
55	1	0	-2.931813	4.092143	-1.154976
56	1	0	-2.148575	3.299619	-2.559066
57	1	0	-0.088543	3.555227	-1.377934
58	1	0	1.331265	4.261601	0.561051
59	1	0	0.695117	2.904877	1.520611
60	1	0	0.582564	4.568256	2.144511
61	1	0	-2.961859	3.793669	1.134140
62	1	0	-1.919705	4.353552	2.452352
63	1	0	-1.812623	2.669100	1.906900
64	1	0	-0.914698	6.241597	1.043141
65	1	0	-2.021220	5.797423	-0.264151
66	1	0	-0.268478	5.895096	-0.575443
67	1	0	-2.155020	-3.726990	1.170498
68	1	0	-0.094227	-2.324325	1.139269
69	1	0	-4.649226	0.703065	-1.134955
70	1	0	-6.689238	-0.656458	-1.135677
71	1	0	-4.577164	-3.968824	0.634315
72	1	0	-7.435434	-3.297830	-1.302656
73	1	0	-7.839018	-2.690710	0.310319
74	1	0	-6.925741	-4.213715	0.140794

TS between Intermediates B and C of catalytic cycle for Ni-catalyzed Mizoroki-Heck reaction, R-tBu-(6)CH3iQuinox ligand, R1=Me, R2=Me, R3=H, R configuration for tBu, other alkene, flipped ligand conformation (RAF)

Species name: intB_to_intC_ts_tBuMeQ_RAF
Full file name: intB_to_intC_ts_tBuMeQ_RAF.log
Command line: B3LYP/Def2SVP empiricaldispersion=GD3BJ freq geom=check guess=read
Point group: C1
NImag: 1

Thermochemistry:
Temperature: 298.150
Zero-point correction= 0.616835 (Hartree/Particle)
Thermal correction to Energy= 0.651343
Thermal correction to Enthalpy= 0.652287
Thermal correction to Gibbs Free Energy= 0.552539
Sum of electronic and zero-point Energies= -3062.221671
Sum of electronic and thermal Energies= -3062.187163
Sum of electronic and thermal Enthalpies= -3062.186219
Sum of electronic and thermal Free Energies= -3062.285968

Standard orientation:

Center Number	Atomic Number	Atomic Type	Coordinates (Angstroms) X	Y	Z
1	6	0	-3.115959	-1.957971	-1.153843
2	6	0	-3.840702	-2.587241	0.018379
3	6	0	-3.170136	-3.224384	0.985205
4	6	0	-1.665552	-3.369483	1.038067
5	6	0	-0.905004	-2.475108	0.069563
6	6	0	-1.697756	-1.591271	-0.738529
7	6	0	-3.808959	-0.726913	-1.777644
8	8	0	-4.468193	-0.805137	-2.792414
9	7	0	-3.584613	0.501643	-1.163935
10	6	0	-2.987864	-3.012149	-2.280777
11	6	0	-3.248840	0.526862	0.209476
12	6	0	-2.170960	-0.232964	0.689206
13	6	0	-2.033710	-0.414911	2.077779
14	6	0	-2.837458	0.286517	2.979877
15	6	0	-3.855561	1.107621	2.494282
16	6	0	-4.077649	1.201629	1.120513
17	6	0	-4.247504	1.655419	-1.765466
18	28	0	-0.270188	-0.615560	0.396301
19	7	0	0.428073	1.170595	-0.164829
20	6	0	1.647730	1.027273	-0.565661
21	8	0	2.128110	1.947773	-1.395068
22	6	0	0.989128	2.790260	-1.752508
23	6	0	-0.044991	2.483202	-0.648012
24	6	0	-0.090919	3.546317	0.492938
25	6	0	-1.000061	3.047627	1.621071
26	6	0	-0.680020	4.839045	-0.095747
27	6	0	1.311933	3.814680	1.062277
28	7	0	1.633370	-1.030683	0.504727
29	6	0	2.428177	-0.131989	-0.100171
30	6	0	3.835372	-0.291221	-0.203119
31	6	0	4.405138	-1.467427	0.398251
32	6	0	3.532631	-2.375340	1.045162
33	6	0	2.177164	-2.134085	1.072886
34	6	0	4.708930	0.633763	-0.844576
35	6	0	6.060730	0.389254	-0.884113
36	6	0	6.640964	-0.775465	-0.295498
37	6	0	5.805884	-1.679136	0.333857
38	6	0	8.125917	-0.983609	-0.373720
39	1	0	-4.933299	-2.562001	0.018938
40	1	0	-3.728708	-3.710117	1.790697
41	1	0	-1.324179	-3.228197	2.080683

42	1	0	-1.410426	-4.422215	0.813642
43	1	0	-0.025718	-2.924410	-0.402311
44	1	0	-1.174967	-1.135736	-1.592232
45	1	0	-2.478242	-3.907080	-1.899689
46	1	0	-2.429880	-2.610907	-3.140035
47	1	0	-3.987907	-3.290103	-2.636431
48	1	0	-1.282357	-1.101728	2.477989
49	1	0	-2.683637	0.166120	4.053942
50	1	0	-4.510496	1.641745	3.185561
51	1	0	-4.924215	1.779639	0.749329
52	1	0	-4.124678	1.603557	-2.853582
53	1	0	-3.788627	2.574391	-1.377119
54	1	0	-5.330245	1.672558	-1.557590
55	1	0	0.658619	2.480778	-2.755341
56	1	0	1.337197	3.827490	-1.783479
57	1	0	-1.056604	2.366845	-1.062031
58	1	0	-0.618560	2.119933	2.067366
59	1	0	-2.016953	2.850375	1.258300
60	1	0	-1.067067	3.806097	2.415478
61	1	0	-0.057208	5.261083	-0.900189
62	1	0	-0.763207	5.607705	0.687029
63	1	0	-1.689656	4.666463	-0.502536
64	1	0	1.247590	4.561674	1.867406
65	1	0	2.011872	4.208726	0.310054
66	1	0	1.749786	2.904152	1.500123
67	1	0	3.933002	-3.272166	1.521298
68	1	0	1.482788	-2.823216	1.553213
69	1	0	4.300143	1.530446	-1.304588
70	1	0	6.717829	1.107413	-1.380908
71	1	0	6.219504	-2.578070	0.796524
72	1	0	8.661714	-0.144227	0.099117
73	1	0	8.458000	-1.022256	-1.424198
74	1	0	8.436335	-1.913856	0.120815

TS between Intermediates B and C of catalytic cycle for Ni-catalyzed Mizoroki-Heck reaction, R-tBu-(6)CH3iQuinox ligand, R1=Me, R2=Me, R3=H, R configuration for tBu, other alkene, other twist conformation (RAT)

Species name: intB_to_intC_ts_tBuMeQ_RAT
Full file name: intB_to_intC_ts_tBuMeQ_RAT.log
Command line: B3LYP/Def2SVP empiricaldispersion=GD3BJ freq geom=check guess=read
Point group: C1
NImag: 1

Thermochemistry:
Temperature: 298.150
Zero-point correction= 0.617940 (Hartree/Particle)
Thermal correction to Energy= 0.652449
Thermal correction to Enthalpy= 0.653394
Thermal correction to Gibbs Free Energy= 0.552807
Sum of electronic and zero-point Energies= -3062.245339
Sum of electronic and thermal Energies= -3062.210829
Sum of electronic and thermal Enthalpies= -3062.209885
Sum of electronic and thermal Free Energies= -3062.310472

Standard orientation:

Center Number	Atomic Number	Atomic Type	Coordinates (Angstroms) X	Y	Z
1	6	0	-1.951232	-1.403192	1.996715
2	6	0	-2.705665	-0.541979	0.990656
3	6	0	-2.692227	0.877602	1.067639
4	6	0	-2.311861	1.548129	2.365976
5	6	0	-1.316133	0.747075	3.149344
6	6	0	-1.136296	-0.562817	2.962458
7	6	0	-0.999236	-2.484420	1.406621
8	8	0	-0.132987	-2.934205	2.133061
9	7	0	-1.222310	-3.029942	0.149845
10	6	0	-3.005204	-2.208547	2.808940
11	6	0	-1.964328	-2.432877	-0.893800
12	6	0	-2.163419	-1.036581	-0.941999
13	6	0	-2.923550	-0.479216	-1.979999
14	6	0	-3.389909	-1.268812	-3.034850
15	6	0	-3.161600	-2.645312	-3.005993
16	6	0	-2.468506	-3.224569	-1.940610
17	6	0	-0.552503	-4.302330	-0.129352
18	28	0	-1.150793	0.374039	-0.103146
19	7	0	-0.066101	1.952927	0.270308
20	6	0	1.195867	1.676674	0.300338
21	8	0	2.024953	2.666330	0.599695
22	6	0	1.192420	3.824249	0.896296
23	6	0	-0.209175	3.417999	0.387330
24	6	0	-0.651529	4.062235	-0.959469
25	6	0	-2.049545	3.537737	-1.319195
26	6	0	0.332782	3.712286	-2.085974
27	6	0	-0.724804	5.585688	-0.765366
28	7	0	0.665069	-0.334511	-0.715865
29	6	0	1.666322	0.358185	-0.147658
30	6	0	3.008162	-0.103012	-0.105551
31	6	0	3.288214	-1.349821	-0.765730
32	6	0	2.221846	-2.002476	-1.424936
33	6	0	0.947287	-1.479979	-1.371606
34	6	0	4.078442	0.571175	0.550909
35	6	0	5.339878	0.027079	0.546866
36	6	0	5.634197	-1.208167	-0.108208
37	6	0	4.607699	-1.870952	-0.752132
38	6	0	7.035403	-1.747043	-0.076607
39	1	0	-3.615603	-1.004762	0.612532
40	1	0	-3.473578	1.386293	0.490437
41	1	0	-1.953854	2.573226	2.205889
42	1	0	-3.238490	1.674534	2.962854
43	1	0	-0.734585	1.264366	3.918160
44	1	0	-0.411753	-1.122836	3.554223
45	1	0	-3.713256	-1.517076	3.288954
46	1	0	-2.508978	-2.801125	3.589360
47	1	0	-3.566247	-2.891225	2.151357
48	1	0	-3.149813	0.590130	-1.977094
49	1	0	-3.941027	-0.812600	-3.859399
50	1	0	-3.541916	-3.283392	-3.806204
51	1	0	-2.333009	-4.305033	-1.922394
52	1	0	-0.079465	-4.273748	-1.120917

53	1	0	-1.267032	-5.140081	-0.103990
54	1	0	0.209112	-4.459886	0.639761
55	1	0	1.633085	4.687014	0.385403
56	1	0	1.224907	3.983990	1.983676
57	1	0	-0.969788	3.665983	1.137753
58	1	0	-2.783769	3.780198	-0.534570
59	1	0	-2.039860	2.446909	-1.451124
60	1	0	-2.398749	3.990235	-2.259193
61	1	0	1.360350	4.037202	-1.859635
62	1	0	0.032005	4.210910	-3.019387
63	1	0	0.349096	2.629062	-2.280953
64	1	0	-1.114504	6.063147	-1.676776
65	1	0	0.260566	6.032996	-0.563206
66	1	0	-1.398476	5.852964	0.064823
67	1	0	2.405291	-2.925032	-1.978679
68	1	0	0.112169	-1.958048	-1.879426
69	1	0	3.891824	1.514056	1.059412
70	1	0	6.148570	0.551946	1.061546
71	1	0	4.799857	-2.817859	-1.261683
72	1	0	7.364331	-1.913030	0.962434
73	1	0	7.739977	-1.026124	-0.522775
74	1	0	7.121691	-2.696788	-0.621482

TS between Intermediates B and C of catalytic cycle for Ni-catalyzed Mizoroki-Heck reaction, R-tBu-(6)CH3iQuinox ligand, R1=Me, R2=Me, R3=H, R configuration for tBu, flipped ligand, other twist conformation (RFT)

Species name: intB_to_intC_ts_tBuMeQ_RFT
Full file name: intB_to_intC_ts_tBuMeQ_RFT.log
Command line: B3LYP/Def2SVP empiricaldispersion=GD3BJ freq geom=check guess=read
Point group: C1
NImag: 1

Thermochemistry:
Temperature: 298.150
Zero-point correction= 0.616992 (Hartree/Particle)
Thermal correction to Energy= 0.651463
Thermal correction to Enthalpy= 0.652407
Thermal correction to Gibbs Free Energy= 0.552804
Sum of electronic and zero-point Energies= -3062.225757
Sum of electronic and thermal Energies= -3062.191287
Sum of electronic and thermal Enthalpies= -3062.190343
Sum of electronic and thermal Free Energies= -3062.289946

Standard orientation:

Center Number	Atomic Number	Atomic Type	Coordinates (Angstroms) X	Y	Z
1	6	0	-3.537053	-1.702135	-0.790928
2	6	0	-3.732854	-2.624279	0.395415
3	6	0	-2.754852	-3.436848	0.810869
4	6	0	-1.381738	-3.513295	0.184348

5	6	0	-1.050675	-2.363052	-0.755487
6	6	0	-2.062627	-1.366479	-0.944636
7	6	0	-4.386076	-0.414368	-0.756143
8	8	0	-5.425175	-0.321396	-1.374225
9	7	0	-3.889972	0.672563	-0.039323
10	6	0	-3.944450	-2.463058	-2.078705
11	6	0	-2.987147	0.464523	1.026080
12	6	0	-1.870524	-0.373244	0.878791
13	6	0	-1.200448	-0.822475	2.032785
14	6	0	-1.474623	-0.268505	3.285433
15	6	0	-2.492783	0.679411	3.404796
16	6	0	-3.265782	1.011755	2.293097
17	6	0	-4.719575	1.875253	-0.055731
18	28	0	-0.259550	-0.650413	-0.197962
19	7	0	0.440642	1.175542	0.026790
20	6	0	1.714484	1.177545	0.216650
21	8	0	2.252679	2.303491	0.675050
22	6	0	1.126257	3.180165	0.997253
23	6	0	-0.061321	2.538644	0.246371
24	6	0	-0.444710	3.232089	-1.096675
25	6	0	-1.416285	2.333923	-1.876833
26	6	0	0.793994	3.489065	-1.970117
27	6	0	-1.136275	4.560428	-0.752016
28	7	0	1.661410	-1.064085	-0.407863
29	6	0	2.479846	-0.049737	-0.066870
30	6	0	3.892626	-0.180787	-0.006612
31	6	0	4.446165	-1.469885	-0.325470
32	6	0	3.551008	-2.509307	-0.668441
33	6	0	2.193600	-2.275904	-0.695232
34	6	0	4.788885	0.872249	0.340188
35	6	0	6.143549	0.643568	0.364282
36	6	0	6.706992	-0.631152	0.050870
37	6	0	5.850946	-1.661545	-0.287252
38	6	0	8.196528	-0.814059	0.095496
39	1	0	-4.720341	-2.652227	0.862926
40	1	0	-2.947590	-4.124773	1.639316
41	1	0	-0.620383	-3.615481	0.980333
42	1	0	-1.310291	-4.462841	-0.379265
43	1	0	-0.443739	-2.617108	-1.631600
44	1	0	-1.912965	-0.681778	-1.790470
45	1	0	-3.358082	-3.387302	-2.166536
46	1	0	-3.786404	-1.843038	-2.973720
47	1	0	-5.011228	-2.714552	-2.033115
48	1	0	-0.426476	-1.592071	1.962407
49	1	0	-0.910126	-0.594526	4.161326
50	1	0	-2.729327	1.116945	4.376908
51	1	0	-4.125016	1.670515	2.416315
52	1	0	-5.090933	2.029486	-1.074830
53	1	0	-4.112526	2.735151	0.255017
54	1	0	-5.595723	1.785973	0.607329
55	1	0	1.387508	4.192566	0.672563
56	1	0	1.003676	3.160471	2.090024
57	1	0	-0.955491	2.488034	0.883528
58	1	0	-2.272727	2.024375	-1.264541
59	1	0	-0.911064	1.424988	-2.234858
60	1	0	-1.801748	2.869990	-2.756963
61	1	0	1.508974	4.183634	-1.504275
62	1	0	0.487178	3.935991	-2.927584
63	1	0	1.323449	2.552317	-2.205066

64	1	0	-1.419744	5.095642	-1.670557
65	1	0	-0.483118	5.230960	-0.170979
66	1	0	-2.053160	4.390264	-0.165033
67	1	0	3.933435	-3.501622	-0.914184
68	1	0	1.491613	-3.067307	-0.952325
69	1	0	4.395842	1.856222	0.583839
70	1	0	6.816959	1.462043	0.630703
71	1	0	6.249648	-2.648226	-0.533615
72	1	0	8.586108	-0.587001	1.101445
73	1	0	8.694143	-0.121064	-0.602659
74	1	0	8.491997	-1.839006	-0.166142

TS between Intermediates B and C of catalytic cycle for Ni-catalyzed Mizoroki-Heck reaction, R-tBu-(6)CH3iQuinox ligand, R1=Me, R2=Me, R3=H, R configuration for tBu, other alkene, flipped ligand, other twist conformation (RAFT)

Species name: intB_to_intC_ts_tBuMeQ_RAFT
Full file name: intB_to_intC_ts_tBuMeQ_RAFT.log
Command line: B3LYP/Def2SVP empiricaldispersion=GD3BJ freq geom=check guess=read
Point group: C1
NImag: 1

Thermochemistry:
Temperature: 298.150
Zero-point correction= 0.617926 (Hartree/Particle)
Thermal correction to Energy= 0.652404
Thermal correction to Enthalpy= 0.653349
Thermal correction to Gibbs Free Energy= 0.553792
Sum of electronic and zero-point Energies= -3062.239074
Sum of electronic and thermal Energies= -3062.204596
Sum of electronic and thermal Enthalpies= -3062.203652
Sum of electronic and thermal Free Energies= -3062.303208

Standard orientation:

Center Number	Atomic Number	Atomic Type	Coordinates (Angstroms) X	Y	Z
1	6	0	-3.257617	-1.675398	-0.614734
2	6	0	-2.150453	-2.115814	0.327317
3	6	0	-0.976967	-2.762315	-0.137270
4	6	0	-0.824462	-3.152373	-1.592095
5	6	0	-1.730672	-2.372772	-2.497434
6	6	0	-2.800394	-1.702615	-2.063500
7	6	0	-3.915711	-0.281999	-0.405845
8	8	0	-4.778285	0.032296	-1.206205
9	7	0	-3.573371	0.589546	0.614682
10	6	0	-4.410455	-2.706685	-0.447205
11	6	0	-2.694538	0.326234	1.691951
12	6	0	-1.558888	-0.483632	1.545561
13	6	0	-0.770653	-0.793725	2.664960
14	6	0	-1.019496	-0.203526	3.905263
15	6	0	-2.114374	0.652982	4.041542

16	6	0	-2.951232	0.899386	2.953968
17	6	0	-4.363021	1.823821	0.670109
18	28	0	-0.415301	-0.881551	0.063578
19	7	0	0.111005	1.004510	-0.119158
20	6	0	1.385609	1.152205	0.012679
21	8	0	1.825618	2.384125	0.241427
22	6	0	0.631114	3.202791	0.441063
23	6	0	-0.494150	2.348584	-0.175720
24	6	0	-0.878203	2.749274	-1.634728
25	6	0	-1.884503	1.739908	-2.199668
26	6	0	0.359977	2.780410	-2.546110
27	6	0	-1.535006	4.138858	-1.586635
28	7	0	1.568905	-1.150897	-0.280207
29	6	0	2.272772	-0.019818	-0.089761
30	6	0	3.691665	0.009074	0.003534
31	6	0	4.380990	-1.249062	-0.104337
32	6	0	3.603304	-2.415985	-0.280380
33	6	0	2.230697	-2.329331	-0.355622
34	6	0	4.471437	1.188035	0.190988
35	6	0	5.841411	1.105780	0.260355
36	6	0	6.537631	-0.136494	0.152831
37	6	0	5.796577	-1.288429	-0.026590
38	6	0	8.036538	-0.156481	0.236100
39	1	0	-2.508790	-2.421282	1.309320
40	1	0	-0.467793	-3.406199	0.589909
41	1	0	0.217933	-3.051920	-1.934265
42	1	0	-1.045816	-4.234743	-1.690746
43	1	0	-1.507075	-2.389337	-3.567982
44	1	0	-3.454762	-1.178366	-2.761108
45	1	0	-4.034563	-3.721484	-0.641676
46	1	0	-5.214962	-2.480274	-1.158239
47	1	0	-4.823523	-2.669536	0.573478
48	1	0	0.062703	-1.496167	2.568423
49	1	0	-0.374284	-0.424863	4.757833
50	1	0	-2.341219	1.109826	5.006919
51	1	0	-3.828188	1.529157	3.095852
52	1	0	-3.738257	2.640371	1.054754
53	1	0	-5.248114	1.713195	1.316751
54	1	0	-4.715272	2.057505	-0.338816
55	1	0	0.802609	4.166079	-0.049381
56	1	0	0.515922	3.349609	1.525216
57	1	0	-1.395485	2.364600	0.449661
58	1	0	-2.831716	1.759733	-1.648984
59	1	0	-1.492760	0.714014	-2.170716
60	1	0	-2.112164	1.980832	-3.248844
61	1	0	1.130234	3.485438	-2.198899
62	1	0	0.066152	3.092832	-3.559089
63	1	0	0.818007	1.783077	-2.635368
64	1	0	-1.876093	4.429263	-2.591548
65	1	0	-0.844237	4.923209	-1.239572
66	1	0	-2.415076	4.140443	-0.923836
67	1	0	4.088470	-3.390651	-0.358200
68	1	0	1.625021	-3.223665	-0.486169
69	1	0	3.978946	2.152942	0.275271
70	1	0	6.422366	2.020678	0.401266
71	1	0	6.296720	-2.255745	-0.112455
72	1	0	8.378202	0.262165	1.196959
73	1	0	8.478058	0.467612	-0.558162
74	1	0	8.439233	-1.173756	0.139761

Intermediate C of catalytic cycle for Ni-catalyzed Mizoroki-Heck reaction, R-tBu-(6)CH3iQuinox ligand, R1=Me, R2=Me, R3=H, R configuration for tBu, parent conformation (R)

Species name: intC_tBuMeQ_R
Full file name: intC_tBuMeQ_R.log
Command line: B3LYP/Def2SVP EmpiricalDispersion=GD3BJ freq geom=check guess=read
Point group: C1
NImag: 0

Thermochemistry:
Temperature: 298.150
Zero-point correction= 0.620880 (Hartree/Particle)
Thermal correction to Energy= 0.655016
Thermal correction to Enthalpy= 0.655960
Thermal correction to Gibbs Free Energy= 0.557380
Sum of electronic and zero-point Energies= -3062.301075
Sum of electronic and thermal Energies= -3062.266939
Sum of electronic and thermal Enthalpies= -3062.265995
Sum of electronic and thermal Free Energies= -3062.364575

Standard orientation:

Center Number	Atomic Number	Atomic Type	Coordinates (Angstroms) X	Y	Z
1	6	0	-1.340228	-2.665197	0.125732
2	6	0	-2.589659	-1.740447	0.149325
3	6	0	-2.597930	-0.885124	-1.121799
4	6	0	-2.807540	-1.731621	-2.364425
5	6	0	-1.440682	-2.375166	-2.443735
6	6	0	-0.785540	-2.736863	-1.293308
7	6	0	-0.137574	-2.164699	0.967717
8	8	0	0.966813	-2.646574	0.767554
9	7	0	-0.337283	-1.199890	1.929046
10	6	0	-1.672422	-4.083173	0.641033
11	6	0	-1.597516	-0.626352	2.213835
12	6	0	-2.706697	-0.897881	1.392582
13	6	0	-3.936302	-0.303752	1.698510
14	6	0	-4.086391	0.561325	2.782647
15	6	0	-2.981059	0.827397	3.592505
16	6	0	-1.748426	0.234136	3.317158
17	6	0	0.824188	-0.794003	2.716023
18	28	0	-0.730166	-0.611586	-1.473510
19	7	0	-0.583209	1.087609	-0.626225
20	6	0	0.604071	1.285691	-0.150786
21	8	0	0.739185	2.278347	0.715670
22	6	0	-0.604902	2.806522	0.956579
23	6	0	-1.466971	2.170132	-0.158434
24	6	0	-1.866057	3.137970	-1.312106
25	6	0	-0.618836	3.767860	-1.952061
26	6	0	-2.647754	2.367531	-2.387069

27	6	0	-2.774879	4.229254	-0.722364
28	7	0	1.294257	-0.475090	-1.490163
29	6	0	1.716982	0.423955	-0.588600
30	6	0	3.061868	0.495730	-0.140735
31	6	0	3.985413	-0.450501	-0.709322
32	6	0	3.500577	-1.362649	-1.674049
33	6	0	2.168097	-1.348959	-2.027795
34	6	0	3.550014	1.417754	0.829877
35	6	0	4.871060	1.386955	1.206779
36	6	0	5.799356	0.452333	0.653598
37	6	0	5.342152	-0.443824	-0.292267
38	6	0	7.230286	0.471378	1.108819
39	1	0	-3.481319	-2.394073	0.116302
40	1	0	-3.217671	0.012260	-1.041465
41	1	0	-3.001896	-1.118486	-3.256349
42	1	0	-3.629310	-2.472475	-2.290780
43	1	0	-0.976158	-2.598505	-3.410267
44	1	0	0.183959	-3.233326	-1.360557
45	1	0	-2.440068	-4.544576	0.002222
46	1	0	-0.776508	-4.718914	0.640800
47	1	0	-2.063867	-4.029388	1.668755
48	1	0	-4.795370	-0.526860	1.060095
49	1	0	-5.056950	1.010784	3.000269
50	1	0	-3.075433	1.488814	4.456372
51	1	0	-0.907792	0.439645	3.976558
52	1	0	0.955504	0.297072	2.669779
53	1	0	0.713316	-1.094843	3.768894
54	1	0	1.703022	-1.289397	2.293939
55	1	0	-0.906335	2.481788	1.961060
56	1	0	-0.538215	3.898526	0.917466
57	1	0	-2.377824	1.730479	0.269641
58	1	0	-0.012706	4.333688	-1.227648
59	1	0	0.023606	3.003203	-2.415421
60	1	0	-0.915954	4.471185	-2.744149
61	1	0	-3.578964	1.943736	-1.981311
62	1	0	-2.921825	3.043634	-3.210595
63	1	0	-2.050471	1.546051	-2.809261
64	1	0	-3.133798	4.895585	-1.520975
65	1	0	-3.657164	3.789796	-0.229876
66	1	0	-2.252816	4.859553	0.014269
67	1	0	4.178722	-2.084343	-2.132658
68	1	0	1.765989	-2.051385	-2.759294
69	1	0	2.873621	2.148048	1.267250
70	1	0	5.229378	2.101028	1.952590
71	1	0	6.026694	-1.170127	-0.736139
72	1	0	7.688518	1.454349	0.911157
73	1	0	7.295224	0.302068	2.196198
74	1	0	7.830985	-0.297032	0.603819

Intermediate C of catalytic cycle for Ni-catalyzed Mizoroki-Heck reaction, R-tBu-(6)CH3iQuinox ligand, R1=Me, R2=Me, R3=H, R configuration for tBu, other alkene conformation (RA)

Species name: intC_tBuMeQ_RA
Full file name: intC_tBuMeQ_RA.log

Command line: B3LYP/Def2SVP EmpiricalDispersion=GD3BJ freq geom=check guess=read
Point group: C1
NImag: 0

Thermochemistry:
Temperature: 298.150
Zero-point correction= 0.622226 (Hartree/Particle)
Thermal correction to Energy= 0.656552
Thermal correction to Enthalpy= 0.657496
Thermal correction to Gibbs Free Energy= 0.557411
Sum of electronic and zero-point Energies= -3062.265900
Sum of electronic and thermal Energies= -3062.231573
Sum of electronic and thermal Enthalpies= -3062.230629
Sum of electronic and thermal Free Energies= -3062.330714

Standard orientation:

Center Number	Atomic Number	Atomic Type	Coordinates (Angstroms) X	Y	Z
1	6	0	-4.150800	0.661323	0.373501
2	6	0	-4.383017	0.461488	1.859968
3	6	0	-3.387997	0.408337	2.752319
4	6	0	-1.919686	0.485847	2.408838
5	6	0	-1.656769	0.767108	0.922586
6	6	0	-2.730544	0.173443	0.007867
7	6	0	-5.228762	-0.118668	-0.407494
8	8	0	-6.322299	0.368482	-0.605371
9	7	0	-4.921621	-1.401446	-0.864286
10	6	0	-4.336415	2.151001	0.022685
11	6	0	-3.741220	-2.070084	-0.517196
12	6	0	-2.658578	-1.329844	-0.000824
13	6	0	-1.509762	-2.013937	0.404296
14	6	0	-1.374249	-3.401535	0.267573
15	6	0	-2.432421	-4.115361	-0.291055
16	6	0	-3.608102	-3.461704	-0.670935
17	6	0	-5.970653	-2.125222	-1.576679
18	28	0	-0.047018	-0.077315	0.324065
19	7	0	1.023037	1.477391	0.322764
20	6	0	2.294104	1.221177	0.374033
21	8	0	3.096532	2.249681	0.596238
22	6	0	2.226333	3.391429	0.871879
23	6	0	0.856775	2.946126	0.319310
24	6	0	0.528225	3.497151	-1.105175
25	6	0	-0.781984	2.884311	-1.619744
26	6	0	0.353389	5.021014	-0.992011
27	6	0	1.653414	3.163029	-2.098764
28	7	0	1.735097	-0.972212	-0.106276
29	6	0	2.770136	-0.150322	0.129648
30	6	0	4.124503	-0.574760	0.084385
31	6	0	4.360279	-1.954458	-0.254880
32	6	0	3.241423	-2.778125	-0.518213
33	6	0	1.964348	-2.262996	-0.429669
34	6	0	5.249587	0.259291	0.346896
35	6	0	6.520984	-0.256811	0.274208
36	6	0	6.771419	-1.622542	-0.062832

37	6	0	5.690831	-2.443361	-0.320676
38	6	0	8.185853	-2.122040	-0.126578
39	1	0	-5.424130	0.416665	2.192751
40	1	0	-3.630179	0.292491	3.814029
41	1	0	-1.454272	-0.455086	2.758575
42	1	0	-1.442044	1.269609	3.022147
43	1	0	-1.606614	1.847669	0.761197
44	1	0	-2.539547	0.506496	-1.029172
45	1	0	-3.633507	2.767492	0.598842
46	1	0	-4.172110	2.323786	-1.052668
47	1	0	-5.358133	2.468802	0.261830
48	1	0	-0.761872	-1.523020	1.074472
49	1	0	-0.485694	-3.917090	0.635273
50	1	0	-2.362820	-5.198803	-0.406646
51	1	0	-4.429242	-4.050452	-1.075262
52	1	0	-5.558613	-2.590984	-2.483456
53	1	0	-6.419512	-2.906488	-0.942075
54	1	0	-6.749715	-1.406220	-1.847109
55	1	0	2.224822	3.542759	1.961330
56	1	0	2.660583	4.265692	0.377252
57	1	0	0.045726	3.227185	1.004400
58	1	0	-0.715252	1.788590	-1.679795
59	1	0	-1.634021	3.142048	-0.975012
60	1	0	-1.001808	3.264803	-2.628244
61	1	0	1.277464	5.531601	-0.679417
62	1	0	0.067654	5.440242	-1.968193
63	1	0	-0.440078	5.280011	-0.272844
64	1	0	1.412954	3.582403	-3.086833
65	1	0	2.626444	3.579488	-1.797707
66	1	0	1.767027	2.075322	-2.227315
67	1	0	3.386756	-3.824151	-0.794337
68	1	0	1.083784	-2.873484	-0.630312
69	1	0	5.097831	1.304352	0.605750
70	1	0	7.373134	0.395731	0.480165
71	1	0	5.848271	-3.492368	-0.581383
72	1	0	8.766609	-1.550780	-0.869302
73	1	0	8.689902	-1.986837	0.844450
74	1	0	8.233384	-3.185914	-0.395183

Intermediate C of catalytic cycle for Ni-catalyzed Mizoroki-Heck reaction, R-tBu-(6)CH3iQuinox ligand, R1=Me, R2=Me, R3=H, R configuration for tBu, flipped ligand conformation (RF)

```
Species name:    intC_tBuMeQ_RF
Full file name:  intC_tBuMeQ_RF.log
Command line:    B3LYP/Def2SVP EmpiricalDispersion=GD3BJ freq geom=check
guess=read
Point group:     C1
NImag:           0

Thermochemistry:
Temperature:     298.150
Zero-point correction=                          0.620585 (Hartree/Particle)
Thermal correction to Energy=                   0.654940
Thermal correction to Enthalpy=                 0.655884
Thermal correction to Gibbs Free Energy=        0.555995
```

Sum of electronic and zero-point Energies= -3062.293347
Sum of electronic and thermal Energies= -3062.258993
Sum of electronic and thermal Enthalpies= -3062.258049
Sum of electronic and thermal Free Energies= -3062.357938

Standard orientation:

Center Number	Atomic Number	Atomic Type	Coordinates (Angstroms) X	Y	Z
1	6	0	-2.932172	-1.189246	0.290834
2	6	0	-3.245188	-0.154199	-0.811067
3	6	0	-2.097574	-0.212512	-1.828063
4	6	0	-2.219775	-1.474419	-2.669022
5	6	0	-1.794988	-2.496675	-1.635778
6	6	0	-2.081386	-2.303290	-0.305917
7	6	0	-2.072840	-0.599974	1.437387
8	8	0	-1.372005	-1.340001	2.113599
9	7	0	-2.149141	0.749312	1.691203
10	6	0	-4.207777	-1.771144	0.932687
11	6	0	-2.967622	1.636111	0.953662
12	6	0	-3.526640	1.219182	-0.267800
13	6	0	-4.307425	2.119497	-0.999708
14	6	0	-4.534813	3.421245	-0.550704
15	6	0	-3.975569	3.827546	0.661711
16	6	0	-3.199076	2.943720	1.412845
17	6	0	-1.358631	1.270894	2.801616
18	28	0	-0.534479	-0.956331	-0.957552
19	7	0	1.244856	-1.804723	-0.522613
20	6	0	2.160739	-0.900115	-0.617713
21	8	0	3.413966	-1.307808	-0.774270
22	6	0	3.344800	-2.749900	-0.974065
23	6	0	1.923952	-3.121304	-0.487825
24	6	0	1.893133	-3.795109	0.917638
25	6	0	0.444017	-4.078367	1.332159
26	6	0	2.649156	-5.131689	0.814140
27	6	0	2.544976	-2.888609	1.973651
28	7	0	0.475043	0.696649	-0.762159
29	6	0	1.795411	0.528185	-0.582582
30	6	0	2.687722	1.607208	-0.348756
31	6	0	2.117028	2.927033	-0.291380
32	6	0	0.720979	3.055606	-0.476576
33	6	0	-0.055239	1.942934	-0.712744
34	6	0	4.092806	1.468815	-0.149318
35	6	0	4.868068	2.578870	0.082837
36	6	0	4.316442	3.895636	0.135748
37	6	0	2.955676	4.045214	-0.050282
38	6	0	5.219988	5.067318	0.390519
39	1	0	-4.164638	-0.488569	-1.324968
40	1	0	-1.931518	0.718006	-2.385132
41	1	0	-1.533035	-1.481412	-3.529048
42	1	0	-3.236450	-1.663586	-3.069099
43	1	0	-1.286275	-3.419946	-1.931920
44	1	0	-1.770065	-3.064701	0.406597
45	1	0	-4.803467	-2.301387	0.174455
46	1	0	-3.954350	-2.472517	1.740238
47	1	0	-4.825248	-0.962259	1.352329

48	1	0	-4.743241	1.785453	-1.945095
49	1	0	-5.148359	4.107420	-1.137272
50	1	0	-4.149094	4.838106	1.037570
51	1	0	-2.785231	3.278632	2.361910
52	1	0	-0.753881	2.125361	2.462585
53	1	0	-2.004408	1.597172	3.631317
54	1	0	-0.707156	0.467273	3.156763
55	1	0	3.501557	-2.937522	-2.046565
56	1	0	4.158334	-3.203951	-0.400064
57	1	0	1.428293	-3.795815	-1.201728
58	1	0	-0.105472	-3.151869	1.539477
59	1	0	-0.086046	-4.655956	0.556653
60	1	0	0.431517	-4.680563	2.253300
61	1	0	3.714323	-5.005403	0.566047
62	1	0	2.604140	-5.660821	1.777522
63	1	0	2.197553	-5.788547	0.052997
64	1	0	2.526115	-3.385124	2.955223
65	1	0	3.597591	-2.659028	1.745539
66	1	0	1.991940	-1.942609	2.076483
67	1	0	0.249180	4.038962	-0.436753
68	1	0	-1.128203	2.026574	-0.864905
69	1	0	4.547603	0.481991	-0.185153
70	1	0	5.943506	2.455502	0.232925
71	1	0	2.502231	5.038310	-0.013077
72	1	0	5.746149	4.951035	1.352217
73	1	0	5.995297	5.136557	-0.390152
74	1	0	4.666160	6.015544	0.411779

Intermediate C of catalytic cycle for Ni-catalyzed Mizoroki-Heck reaction, R-tBu-(6)CH3iQuinox ligand, R1=Me, R2=Me, R3=H, R configuration for tBu, other alkene conformation (RA)

Species name: intC_tBuMeQ_RT
Full file name: intC_tBuMeQ_RT.log
Command line: B3LYP/Def2SVP EmpiricalDispersion=GD3BJ freq geom=check guess=read
Point group: C1
NImag: 0

Thermochemistry:
Temperature: 298.150
Zero-point correction= 0.619747 (Hartree/Particle)
Thermal correction to Energy= 0.654403
Thermal correction to Enthalpy= 0.655347
Thermal correction to Gibbs Free Energy= 0.553707
Sum of electronic and zero-point Energies= -3062.264780
Sum of electronic and thermal Energies= -3062.230124
Sum of electronic and thermal Enthalpies= -3062.229180
Sum of electronic and thermal Free Energies= -3062.330820

Standard orientation:

Center Number	Atomic Number	Atomic Type	Coordinates (Angstroms) X	Y	Z

1	6	0	-3.839657	0.279171	1.171673
2	6	0	-4.735883	0.923137	0.131100
3	6	0	-4.256892	1.640436	-0.890833
4	6	0	-2.794413	1.886315	-1.168525
5	6	0	-1.849993	1.290137	-0.113480
6	6	0	-2.443653	0.042589	0.553509
7	6	0	-4.478541	-1.054004	1.612722
8	8	0	-5.282828	-1.088188	2.520006
9	7	0	-4.132715	-2.215836	0.916801
10	6	0	-3.749894	1.181243	2.415725
11	6	0	-3.295947	-2.208809	-0.204404
12	6	0	-2.488726	-1.080667	-0.449738
13	6	0	-1.688202	-1.054209	-1.594197
14	6	0	-1.636291	-2.126168	-2.495669
15	6	0	-2.411323	-3.251407	-2.224691
16	6	0	-3.236497	-3.294709	-1.095756
17	6	0	-4.807824	-3.452601	1.298614
18	28	0	-0.213694	0.437668	-0.690810
19	7	0	0.962764	1.627317	0.195096
20	6	0	2.095315	1.088109	0.523877
21	8	0	2.903180	1.816493	1.277877
22	6	0	2.154484	3.016872	1.637899
23	6	0	0.995567	3.043849	0.619975
24	6	0	1.212856	4.014933	-0.581497
25	6	0	0.026605	3.904646	-1.547234
26	6	0	2.512269	3.682735	-1.332570
27	6	0	1.269340	5.450473	-0.031241
28	7	0	1.457688	-0.736963	-0.750286
29	6	0	2.439687	-0.252806	0.027688
30	6	0	3.638807	-0.963436	0.303895
31	6	0	3.777762	-2.261745	-0.302518
32	6	0	2.718821	-2.734871	-1.109838
33	6	0	1.591933	-1.962724	-1.302051
34	6	0	4.700133	-0.487132	1.127591
35	6	0	5.819986	-1.258800	1.323756
36	6	0	5.973198	-2.547321	0.725840
37	6	0	4.952712	-3.023878	-0.073315
38	6	0	7.223740	-3.338286	0.980337
39	1	0	-5.815300	0.819897	0.275258
40	1	0	-4.960394	2.102879	-1.591288
41	1	0	-2.584329	1.516406	-2.188709
42	1	0	-2.632788	2.972988	-1.239485
43	1	0	-1.614335	2.042499	0.648937
44	1	0	-1.782938	-0.276370	1.382637
45	1	0	-3.346921	2.165336	2.140818
46	1	0	-3.102245	0.727171	3.182653
47	1	0	-4.744469	1.319325	2.856312
48	1	0	-1.259434	-0.086160	-1.967578
49	1	0	-1.038967	-2.061574	-3.407050
50	1	0	-2.403871	-4.100420	-2.911259
51	1	0	-3.850766	-4.177354	-0.927262
52	1	0	-5.311074	-3.277936	2.254373
53	1	0	-4.075867	-4.266550	1.403923
54	1	0	-5.563383	-3.742331	0.550466
55	1	0	2.841428	3.866429	1.574527
56	1	0	1.815862	2.890238	2.676705
57	1	0	0.047751	3.302542	1.111369
58	1	0	-0.912532	4.193403	-1.053261

59	1	0	-0.090590	2.880442	-1.929116
60	1	0	0.173237	4.575403	-2.406780
61	1	0	3.400919	3.734641	-0.685034
62	1	0	2.664436	4.401456	-2.151340
63	1	0	2.471342	2.679039	-1.783145
64	1	0	1.337709	6.167634	-0.862734
65	1	0	2.142948	5.623839	0.615751
66	1	0	0.362655	5.694907	0.545234
67	1	0	2.788811	-3.716135	-1.582825
68	1	0	0.755035	-2.316743	-1.901638
69	1	0	4.621654	0.489154	1.598888
70	1	0	6.624309	-0.877572	1.957912
71	1	0	5.036936	-4.005691	-0.544635
72	1	0	7.353400	-3.524211	2.059166
73	1	0	8.112406	-2.779149	0.644331
74	1	0	7.208937	-4.306663	0.462357

Intermediate C of catalytic cycle for Ni-catalyzed Mizoroki-Heck reaction, R-tBu-(6)CH3iQuinox ligand, R1=Me, R2=Me, R3=H, R configuration for tBu, other alkene, flipped ligand conformation (RAF)

Species name: intC_tBuMeQ_RAF
Full file name: intC_tBuMeQ_RAF.log
Command line: B3LYP/Def2SVP EmpiricalDispersion=GD3BJ freq geom=check guess=read
Point group: C1
NImag: 0

Thermochemistry:
Temperature: 298.150
Zero-point correction= 0.621605 (Hartree/Particle)
Thermal correction to Energy= 0.656175
Thermal correction to Enthalpy= 0.657119
Thermal correction to Gibbs Free Energy= 0.555566
Sum of electronic and zero-point Energies= -3062.263879
Sum of electronic and thermal Energies= -3062.229309
Sum of electronic and thermal Enthalpies= -3062.228365
Sum of electronic and thermal Free Energies= -3062.329918

Standard orientation:

Center Number	Atomic Number	Atomic Type	Coordinates (Angstroms) X	Y	Z
1	6	0	-3.584842	-1.882343	0.785892
2	6	0	-4.248324	-1.100288	1.904911
3	6	0	-3.592116	-0.213504	2.661648
4	6	0	-2.135810	0.154329	2.494636
5	6	0	-1.421428	-0.664919	1.407192
6	6	0	-2.360713	-1.091212	0.279244
7	6	0	-4.609566	-2.107457	-0.345889
8	8	0	-5.393261	-3.032550	-0.295666
9	7	0	-4.619023	-1.216065	-1.420677
10	6	0	-3.174228	-3.275201	1.302483

11	6	0	-3.862041	-0.037253	-1.437714
12	6	0	-2.790624	0.100062	-0.534062
13	6	0	-2.092655	1.310408	-0.497225
14	6	0	-2.364858	2.359465	-1.382475
15	6	0	-3.387693	2.187858	-2.313707
16	6	0	-4.141852	1.011180	-2.332916
17	6	0	-5.609787	-1.444227	-2.468272
18	28	0	-0.105425	0.417177	0.528875
19	7	0	1.241568	1.639106	-0.245264
20	6	0	2.368936	1.047769	-0.443192
21	8	0	3.346879	1.776703	-0.966944
22	6	0	2.762840	3.074997	-1.289502
23	6	0	1.407525	3.070372	-0.542209
24	6	0	1.356855	3.939173	0.750067
25	6	0	0.008652	3.722376	1.454375
26	6	0	1.478380	5.414737	0.337136
27	6	0	2.490295	3.556963	1.714732
28	7	0	1.383294	-0.848779	0.498193
29	6	0	2.512856	-0.369191	-0.067500
30	6	0	3.673142	-1.161940	-0.275835
31	6	0	3.612663	-2.539508	0.135467
32	6	0	2.407082	-3.004211	0.704565
33	6	0	1.337212	-2.151477	0.865809
34	6	0	4.884737	-0.689106	-0.861861
35	6	0	5.953046	-1.538720	-1.017090
36	6	0	5.906306	-2.907643	-0.610456
37	6	0	4.739625	-3.381860	-0.043512
38	6	0	7.110572	-3.781478	-0.808827
39	1	0	-5.297688	-1.329761	2.111858
40	1	0	-4.126209	0.295170	3.471098
41	1	0	-2.094559	1.246365	2.316737
42	1	0	-1.614801	0.018819	3.457763
43	1	0	-0.980939	-1.549444	1.879101
44	1	0	-1.804904	-1.757823	-0.408395
45	1	0	-2.492921	-3.178937	2.158758
46	1	0	-2.677801	-3.855311	0.507756
47	1	0	-4.061010	-3.835292	1.622171
48	1	0	-1.470314	1.583400	0.387278
49	1	0	-1.817044	3.300731	-1.321642
50	1	0	-3.627969	2.987450	-3.017232
51	1	0	-4.957930	0.921208	-3.047316
52	1	0	-6.013700	-2.452198	-2.334303
53	1	0	-5.139282	-1.356759	-3.458236
54	1	0	-6.439251	-0.721902	-2.398644
55	1	0	2.652424	3.121252	-2.382281
56	1	0	3.466742	3.846261	-0.958892
57	1	0	0.594585	3.394506	-1.208293
58	1	0	-0.088254	2.688098	1.820643
59	1	0	-0.841428	3.938611	0.789069
60	1	0	-0.077020	4.384740	2.328359
61	1	0	2.437355	5.632236	-0.158510
62	1	0	1.416962	6.064012	1.223180
63	1	0	0.667091	5.706632	-0.348945
64	1	0	2.439119	4.179068	2.620650
65	1	0	3.487929	3.707237	1.274851
66	1	0	2.408351	2.506269	2.034051
67	1	0	2.315556	-4.044490	1.021956
68	1	0	0.405763	-2.504728	1.300631
69	1	0	4.961925	0.346051	-1.183009

70	1	0	6.873500	-1.158029	-1.466770
71	1	0	4.667900	-4.423374	0.277686
72	1	0	7.980779	-3.371448	-0.270375
73	1	0	7.387433	-3.825172	-1.874998
74	1	0	6.936597	-4.806390	-0.454614

Intermediate C of catalytic cycle for Ni-catalyzed Mizoroki-Heck reaction, R-tBu-(6)CH3iQuinox ligand, R1=Me, R2=Me, R3=H, R configuration for tBu, other alkene, other twist conformation (RAT)

Species name: intC_tBuMeQ_RAT
Full file name: intC_tBuMeQ_RAT.log
Command line: B3LYP/Def2SVP EmpiricalDispersion=GD3BJ freq geom=check guess=read
Point group: C1
NImag: 0

Thermochemistry:
Temperature: 298.150
Zero-point correction= 0.619616 (Hartree/Particle)
Thermal correction to Energy= 0.654325
Thermal correction to Enthalpy= 0.655269
Thermal correction to Gibbs Free Energy= 0.554033
Sum of electronic and zero-point Energies= -3062.276242
Sum of electronic and thermal Energies= -3062.241533
Sum of electronic and thermal Enthalpies= -3062.240589
Sum of electronic and thermal Free Energies= -3062.341824

Standard orientation:

Center Number	Atomic Number	Atomic Type	Coordinates (Angstroms) X	Y	Z
1	6	0	3.311894	-0.583644	-1.512923
2	6	0	3.155764	0.490381	-0.398908
3	6	0	2.143452	1.596543	-0.742711
4	6	0	1.697722	1.674284	-2.193372
5	6	0	1.269091	0.297906	-2.630411
6	6	0	2.003344	-0.764699	-2.275689
7	6	0	3.696525	-1.981542	-1.005507
8	8	0	4.233301	-2.772435	-1.751709
9	7	0	3.329790	-2.360041	0.287425
10	6	0	4.422730	-0.146970	-2.495312
11	6	0	2.766442	-1.489100	1.215418
12	6	0	2.602713	-0.106264	0.897830
13	6	0	2.150708	0.774309	1.915437
14	6	0	1.746623	0.294749	3.174800
15	6	0	1.861616	-1.060701	3.442602
16	6	0	2.387105	-1.942925	2.486030
17	6	0	3.599346	-3.743331	0.675672
18	28	0	0.739383	0.677082	0.193446
19	7	0	-0.764060	1.783688	-0.346349
20	6	0	-1.873168	1.125296	-0.449462
21	8	0	-2.925742	1.792304	-0.906446

22	6	0	-2.437493	3.106589	-1.300387
23	6	0	-1.060527	3.209701	-0.610145
24	6	0	-1.044994	4.078626	0.683884
25	6	0	0.370565	4.079630	1.274855
26	6	0	-2.030841	3.529964	1.727067
27	6	0	-1.419916	5.520024	0.298771
28	7	0	-0.780794	-0.681117	0.513441
29	6	0	-1.941277	-0.283966	-0.031081
30	6	0	-3.072069	-1.137067	-0.168305
31	6	0	-2.931388	-2.484881	0.315565
32	6	0	-1.693276	-2.859436	0.884822
33	6	0	-0.660348	-1.949539	0.960213
34	6	0	-4.317887	-0.758908	-0.748888
35	6	0	-5.345330	-1.667681	-0.833583
36	6	0	-5.220198	-3.007097	-0.354240
37	6	0	-4.019172	-3.390074	0.209483
38	6	0	-6.382358	-3.950323	-0.475604
39	1	0	4.157854	0.907991	-0.191584
40	1	0	2.419053	2.574105	-0.326034
41	1	0	0.884414	2.400511	-2.314652
42	1	0	2.517149	2.046481	-2.838938
43	1	0	0.369330	0.172624	-3.239396
44	1	0	1.734421	-1.772430	-2.602362
45	1	0	4.198175	0.837508	-2.923944
46	1	0	4.520620	-0.877024	-3.306546
47	1	0	5.389860	-0.084062	-1.973500
48	1	0	2.269072	1.848549	1.783809
49	1	0	1.399797	0.991413	3.939435
50	1	0	1.573549	-1.453960	4.420024
51	1	0	2.506118	-2.990352	2.753429
52	1	0	2.662214	-4.271707	0.910070
53	1	0	4.259904	-3.777035	1.554632
54	1	0	4.090389	-4.234640	-0.169078
55	1	0	-3.173602	3.846336	-0.970238
56	1	0	-2.371468	3.117287	-2.398469
57	1	0	-0.309520	3.619212	-1.299294
58	1	0	1.106450	4.471766	0.555185
59	1	0	0.672941	3.064516	1.556513
60	1	0	0.408505	4.712585	2.173919
61	1	0	-3.064312	3.490958	1.349653
62	1	0	-2.033855	4.174873	2.618412
63	1	0	-1.745015	2.518020	2.053277
64	1	0	-1.332871	6.176749	1.177162
65	1	0	-2.454425	5.607560	-0.066729
66	1	0	-0.747410	5.915068	-0.479740
67	1	0	-1.552836	-3.871981	1.267859
68	1	0	0.299022	-2.212509	1.399545
69	1	0	-4.453941	0.252002	-1.123542
70	1	0	-6.292861	-1.358842	-1.282244
71	1	0	-3.888452	-4.407517	0.585088
72	1	0	-6.678869	-4.068860	-1.530723
73	1	0	-7.261334	-3.555678	0.060032
74	1	0	-6.148200	-4.943608	-0.069431

Intermediate C of catalytic cycle for Ni-catalyzed Mizoroki-Heck reaction, R-tBu-(6)CH3iQuinox ligand, R1=Me, R2=Me, R3=H, R configuration for tBu, flipped ligand, other twist conformation (RFT)

Species name: intC_tBuMeQ_RFT
Full file name: intC_tBuMeQ_RFT.log
Command line: B3LYP/Def2SVP EmpiricalDispersion=GD3BJ freq geom=check guess=read
Point group: C1
NImag: 0

Thermochemistry:
Temperature: 298.150
Zero-point correction= 0.621186 (Hartree/Particle)
Thermal correction to Energy= 0.655706
Thermal correction to Enthalpy= 0.656651
Thermal correction to Gibbs Free Energy= 0.556032
Sum of electronic and zero-point Energies= -3062.267944
Sum of electronic and thermal Energies= -3062.233423
Sum of electronic and thermal Enthalpies= -3062.232479
Sum of electronic and thermal Free Energies= -3062.333098

Standard orientation:

Center Number	Atomic Number	Atomic Type	Coordinates (Angstroms) X	Y	Z
1	6	0	3.447389	-2.159408	0.665936
2	6	0	3.616011	-3.066783	-0.537651
3	6	0	2.638359	-3.290432	-1.422806
4	6	0	1.276550	-2.639340	-1.384042
5	6	0	1.044364	-1.776342	-0.138795
6	6	0	2.335708	-1.125760	0.373754
7	6	0	4.791753	-1.463986	0.967513
8	8	0	5.658004	-2.043385	1.588224
9	7	0	4.989722	-0.158733	0.509520
10	6	0	3.100969	-3.008471	1.905955
11	6	0	4.118935	0.471065	-0.388134
12	6	0	2.821872	-0.051488	-0.566415
13	6	0	1.985935	0.532053	-1.521255
14	6	0	2.359234	1.667883	-2.251053
15	6	0	3.623318	2.208317	-2.028807
16	6	0	4.501948	1.610237	-1.118041
17	6	0	6.270049	0.469402	0.822046
18	28	0	-0.009425	-0.196834	-0.376494
19	7	0	-0.998146	1.507341	-0.392123
20	6	0	-2.268997	1.318685	-0.288855
21	8	0	-3.039945	2.398765	-0.300300
22	6	0	-2.152332	3.529097	-0.566245
23	6	0	-0.735535	2.954132	-0.328451
24	6	0	-0.073053	3.359431	1.023250
25	6	0	1.256039	2.606500	1.180705
26	6	0	-0.988061	3.015736	2.209095
27	6	0	0.206232	4.870296	0.981304
28	7	0	-1.764803	-0.948191	-0.064071
29	6	0	-2.776410	-0.054902	-0.124385
30	6	0	-4.143336	-0.428341	-0.032708

31	6	0	-4.433856	-1.828188	0.130933
32	6	0	-3.345495	-2.727525	0.182684
33	6	0	-2.052147	-2.264105	0.082437
34	6	0	-5.237683	0.484103	-0.092202
35	6	0	-6.527136	0.020669	0.008234
36	6	0	-6.829468	-1.365880	0.173636
37	6	0	-5.780241	-2.262504	0.231324
38	6	0	-8.261173	-1.804911	0.278302
39	1	0	4.572487	-3.589670	-0.628012
40	1	0	2.818755	-3.983505	-2.251203
41	1	0	1.163310	-2.061399	-2.320083
42	1	0	0.500076	-3.421467	-1.452548
43	1	0	0.609851	-2.377097	0.672609
44	1	0	2.123094	-0.624345	1.338318
45	1	0	2.176498	-3.575131	1.732893
46	1	0	2.970126	-2.369252	2.793787
47	1	0	3.912947	-3.714904	2.115427
48	1	0	1.063800	0.006085	-1.852022
49	1	0	1.687486	2.093334	-2.998693
50	1	0	3.952263	3.087389	-2.586764
51	1	0	5.495856	2.035187	-0.991323
52	1	0	6.753281	-0.123010	1.604663
53	1	0	6.108446	1.498087	1.175257
54	1	0	6.931452	0.490731	-0.059262
55	1	0	-2.438919	4.340637	0.110996
56	1	0	-2.323423	3.839744	-1.607017
57	1	0	-0.050629	3.233260	-1.142130
58	1	0	1.939137	2.800238	0.341353
59	1	0	1.091850	1.519832	1.237601
60	1	0	1.757781	2.913592	2.110403
61	1	0	-1.959768	3.530446	2.159270
62	1	0	-0.509191	3.318190	3.152234
63	1	0	-1.173952	1.931992	2.270682
64	1	0	0.708175	5.188657	1.907169
65	1	0	-0.715886	5.465689	0.891416
66	1	0	0.864551	5.130375	0.137037
67	1	0	-3.524385	-3.797428	0.303367
68	1	0	-1.205703	-2.945510	0.120757
69	1	0	-5.048272	1.547234	-0.215560
70	1	0	-7.353653	0.734105	-0.039089
71	1	0	-5.975658	-3.329901	0.356084
72	1	0	-8.824374	-1.511751	-0.622889
73	1	0	-8.755132	-1.317783	1.135046
74	1	0	-8.348692	-2.892696	0.401830

Intermediate C of catalytic cycle for Ni-catalyzed Mizoroki-Heck reaction, R-tBu-(6)CH3iQuinox ligand, R1=Me, R2=Me, R3=H, R configuration for tBu, other alkene, flipped ligand, other twist conformation (RAFT)

Species name: intC_tBuMeQ_RAFT
Full file name: intC_tBuMeQ_RAFT.log
Command line: B3LYP/Def2SVP EmpiricalDispersion=GD3BJ freq geom=check guess=read
Point group: C1
NImag: 0

Thermochemistry:
Temperature: 298.150
Zero-point correction= 0.619874 (Hartree/Particle)
Thermal correction to Energy= 0.654354
Thermal correction to Enthalpy= 0.655298
Thermal correction to Gibbs Free Energy= 0.555911
Sum of electronic and zero-point Energies= -3062.278333
Sum of electronic and thermal Energies= -3062.243852
Sum of electronic and thermal Enthalpies= -3062.242908
Sum of electronic and thermal Free Energies= -3062.342295

Standard orientation:

Center Number	Atomic Number	Atomic Type	Coordinates (Angstroms) X	Y	Z
1	6	0	-3.421014	-1.854695	-0.589099
2	6	0	-2.409541	-2.050533	0.572495
3	6	0	-0.998708	-2.461636	0.117219
4	6	0	-0.820881	-2.853417	-1.341622
5	6	0	-1.534661	-1.867861	-2.224827
6	6	0	-2.728438	-1.384731	-1.862363
7	6	0	-4.511892	-0.812022	-0.305593
8	8	0	-5.578873	-0.871973	-0.877581
9	7	0	-4.212386	0.265298	0.534199
10	6	0	-4.128908	-3.197246	-0.876858
11	6	0	-3.062609	0.338176	1.313963
12	6	0	-2.147647	-0.756596	1.346983
13	6	0	-1.106871	-0.744919	2.317649
14	6	0	-0.884794	0.377149	3.134870
15	6	0	-1.729448	1.471676	3.013592
16	6	0	-2.816322	1.452333	2.128254
17	6	0	-5.225140	1.312609	0.653961
18	28	0	-0.301956	-0.682024	0.275652
19	7	0	0.362620	1.210353	0.105764
20	6	0	1.651078	1.240590	0.057774
21	8	0	2.230719	2.437157	0.045483
22	6	0	1.164604	3.410381	0.228669
23	6	0	-0.127024	2.598502	-0.018435
24	6	0	-0.811542	2.856949	-1.393821
25	6	0	-2.097416	2.026448	-1.460277
26	6	0	0.119011	2.460248	-2.550049
27	6	0	-1.179500	4.346933	-1.489647
28	7	0	1.620568	-1.088615	0.022267
29	6	0	2.426127	-0.011078	0.022580
30	6	0	3.847394	-0.104179	0.017146
31	6	0	4.417852	-1.424556	0.026627
32	6	0	3.534062	-2.527448	0.056973
33	6	0	2.172881	-2.325122	0.059376
34	6	0	4.738808	1.008630	0.000622
35	6	0	6.098335	0.806353	-0.010780
36	6	0	6.675868	-0.499022	-0.004050
37	6	0	5.826459	-1.588231	0.015353
38	6	0	8.169563	-0.651004	-0.018031
39	1	0	-2.841726	-2.792993	1.266342
40	1	0	-0.536466	-3.181039	0.808467
41	1	0	0.243924	-2.910222	-1.608754

42	1	0	-1.216940	-3.873667	-1.521548
43	1	0	-1.088126	-1.577256	-3.179964
44	1	0	-3.283549	-0.709821	-2.517910
45	1	0	-3.390351	-3.974480	-1.113581
46	1	0	-4.820341	-3.092204	-1.720641
47	1	0	-4.707952	-3.522322	0.000961
48	1	0	-0.567792	-1.667336	2.537192
49	1	0	-0.085471	0.364307	3.877331
50	1	0	-1.579813	2.351641	3.643693
51	1	0	-3.487928	2.307336	2.106426
52	1	0	-4.830480	2.279848	0.307393
53	1	0	-5.555486	1.412818	1.698428
54	1	0	-6.074647	1.024516	0.028258
55	1	0	1.335892	4.226619	-0.480780
56	1	0	1.242931	3.794613	1.256219
57	1	0	-0.865055	2.785023	0.773008
58	1	0	-2.821415	2.364345	-0.706216
59	1	0	-1.894751	0.963667	-1.283732
60	1	0	-2.569599	2.121395	-2.449706
61	1	0	1.085045	2.988005	-2.510817
62	1	0	-0.349113	2.707740	-3.514461
63	1	0	0.317787	1.377690	-2.546090
64	1	0	-1.778905	4.526871	-2.394721
65	1	0	-0.296677	5.001135	-1.553793
66	1	0	-1.781120	4.667276	-0.623353
67	1	0	3.928798	-3.544831	0.079353
68	1	0	1.483889	-3.166174	0.092255
69	1	0	4.340297	2.019021	-0.007609
70	1	0	6.763872	1.673116	-0.026835
71	1	0	6.232996	-2.602109	0.022337
72	1	0	8.619615	-0.152513	0.856147
73	1	0	8.600449	-0.173986	-0.913613
74	1	0	8.474467	-1.706140	-0.008686

Mizoroki-Heck Cyclization

Ni-Catalyzed

B3LYP/Def2SVP EmpiricalDispersion=GD3BJ optimization

B3LYP/Def2TZVPP EmpiricalDispersion=GD3BJ single-point

(thermodynamic corrections from the optimization at left)

Resulting stereochemistry in intC

1st = cabon bearing R1 (CH3 in this case) adjacent to C=O

2nd = next carbon

R1=R2=CH3; R3=H

**L=bpy**	**E (elec)**	**H**	**G**	**ΔE**	**ΔH**	**ΔG**	**E (elec)**	**H**	**G**	**ΔE**	**ΔH**	**ΔG**			
intB	-2713.85740	-2713.39213	-2713.47368	0.0	0.0	0.0	-2715.37357	-2714.90830	-2714.98985	0.0	0.0	0.0			
intB_to_intC_ts	-2713.85665	-2713.39236	-2713.47183	0.5	-0.1	1.2	-2715.37164	-2714.90734	-2714.98682	1.2	0.6	1.9			
intC	-2713.89035	-2713.42323	-2713.50331	-20.7	-19.5	-18.6	-2715.40473	-2714.93760	-2715.01768	-19.6	-18.4	-17.5			

L=tBu-(6)CH3iQuinox

Nomenclature of stereoisomers:

R configuration of cabon bearing tBu group.

"R" = parent configuration

"A" = Ni complexed to other alkene of cyclohexadiene (compared to parent)

"F" = tBu-(6)CH3iQuinox ligand flipped ~180° (compared to parent)

"T" = amide twisted other way (compared to parent)

	E (elec)	H	G	ΔE	ΔH	ΔG	E (elec)	H	G	ΔE	ΔH	ΔG			
intB_tBuMeQ_R	-3062.84891	-3062.19526	-3062.29875	20.6	20.4	19.4	-3064.75092	-3064.09727	-3064.20077	20.7	20.5	19.5			
intB_to_intC_ts_tBuMeQ_R	-3062.84524	-3062.19198	-3062.29252	22.9	22.4	23.3	-3064.74585	-3064.09259	-3064.19313	23.9	23.4	24.3			
intC_tBuMeQ_R	-3062.92196	-3062.26600	-3062.36458	-25.2	-24.0	-21.9	-3064.81819	-3064.16223	-3064.26081	-21.5	-20.3	-18.2	R	R	
intB_tBuMeQ_RA	-3062.88010	-3062.22605	-3062.32799	1.0	1.0	1.0	-3064.78137	-3064.12732	-3064.22926	1.6	1.6	1.6			
intB_to_intC_ts_tBuMeQ_RA	-3062.83982	-3062.18731	-3062.28692	26.3	25.3	26.8	-3064.74103	-3064.08852	-3064.18813	26.9	25.9	27.4			
intC_tBuMeQ_RA	-3062.88813	-3062.23063	-3062.33071	-4.0	-1.8	-0.7	-3064.78922	-3064.13173	-3064.23181	-3.3	-1.2	0.0	S	S	
intB_tBuMeQ_RF	-3062.84325	-3062.18866	-3062.29050	24.1	24.5	24.5	-3064.74667	-3064.09208	-3064.19392	23.4	23.7	23.8			
intB_to_intC_ts_tBuMeQ_RF	-3062.84188	-3062.18835	-3062.28764	25.0	24.7	26.3	-3064.74229	-3064.08876	-3064.18805	26.1	25.8	27.4			
intC_tBuMeQ_RF	-3062.91393	-3062.25805	-3062.35794	-20.2	-19.0	-17.8	-3064.81135	-3064.15546	-3064.25535	-17.2	-16.1	-14.8	R	R	
intB_tBuMeQ_RT	-3062.88173	-3062.22770	-3062.32961	0.0	0.0	0.0	-3064.78390	-3064.12987	-3064.23178	0.0	0.0	0.0			Lowest energy intermediate B
intB_to_intC_ts_tBuMeQ_RT	-3062.84354	-3062.19106	-3062.29098	24.0	23.0	24.2	-3064.74458	-3064.09211	-3064.19202	24.7	23.7	24.9			
intC_tBuMeQ_RT	-3062.88453	-3062.22918	-3062.33082	-1.8	-0.9	-0.8	-3064.78534	-3064.12999	-3064.23163	-0.9	-0.1	0.1	R	R	
intB_tBuMeQ_RAF	-3062.87955	-3062.22540	-3062.32672	1.4	1.4	1.8	-3064.78126	-3064.12710	-3064.22842	1.7	1.7	2.1			
intB_to_intC_ts_tBuMeQ_RAF	-3062.83851	-3062.18622	-3062.28597	27.1	26.0	27.4	-3064.73901	-3064.08673	-3064.18648	28.2	27.1	28.4			
intC_tBuMeQ_RAF	-3062.88548	-3062.22837	-3062.32992	-2.4	-0.4	-0.2	-3064.78644	-3064.12932	-3064.23087	-1.6	0.3	0.6	S	S	
intB_tBuMeQ_RAT	-3062.86461	-3062.21017	-3062.31281	10.7	11.0	10.5	-3064.76580	-3064.11136	-3064.21400	11.4	11.6	11.2			
intB_to_intC_ts_tBuMeQ_RAT	-3062.86328	-3062.20989	-3062.31047	11.6	11.2	12.0	-3064.76323	-3064.10984	-3064.21043	13.0	12.6	13.4			Lowest energy pathway
intC_tBuMeQ_RAT	-3062.89586	-3062.24059	-3062.34182	-8.9	-8.1	-7.7	-3064.79386	-3064.13860	-3064.23983	-6.3	-5.5	-5.1	S	S	
intB_tBuMeQ_RFT	-3062.87927	-3062.22487	-3062.32524	1.5	1.8	2.7	-3064.78087	-3064.12647	-3064.22684	1.9	2.1	3.1			
intB_to_intC_ts_tBuMeQ_RFT	-3062.84275	-3062.19034	-3062.28995	24.5	23.4	24.9	-3064.74323	-3064.09082	-3064.19042	25.5	24.5	26.0			
intC_tBuMeQ_RFT	-3062.88913	-3062.23248	-3062.33310	-4.6	-3.0	-2.2	-3064.79050	-3064.13385	-3064.23447	-4.1	-2.5	-1.7	R	R	
intB_tBuMeQ_RAFT	-3062.86555	-3062.21078	-3062.31094	10.2	10.6	11.7	-3064.76850	-3064.11373	-3064.21390	9.7	10.1	11.2			
intB_to_intC_ts_tBuMeQ_RAFT	-3062.85700	-3062.20365	-3062.30321	15.5	15.1	16.6	-3064.75809	-3064.10474	-3064.20430	16.2	15.8	17.2			Second lowest energy pathway
intC_tBuMeQ_RAFT	-3062.89821	-3062.24291	-3062.34230	-10.3	-9.5	-8.0	-3064.79610	-3064.14080	-3064.24019	-7.7	-6.9	-5.3	S	S	

Energy Conversion 627.51
