# Supplementary material for: Ni-Catalyzed Enantioselective Intramolecular Mizoroki–Heck Reaction for the Synthesis of Phenanthridinone Derivatives
Source: J Org Chem. 2023 Jun 15;88(13):8203–26. doi: 10.1021/acs.joc.3c00202 (PMC10337041; doi:10.1021/acs.joc.3c00202)
Supplement: Supplementary file 2 — jo3c00202_si_002.zip [file jo3c00202_si_002.zip › 2d-1 Deuterated side product/1H deuterated side product 2d-1/1/pdata/1/email_DR-107-074-076-078 combined F7 cosy_1_1.pdf]

DR-107-074-076-078 combined F7 cosy
PROTON CDCl3 {D:\nmrusers\malachowski} BM 3

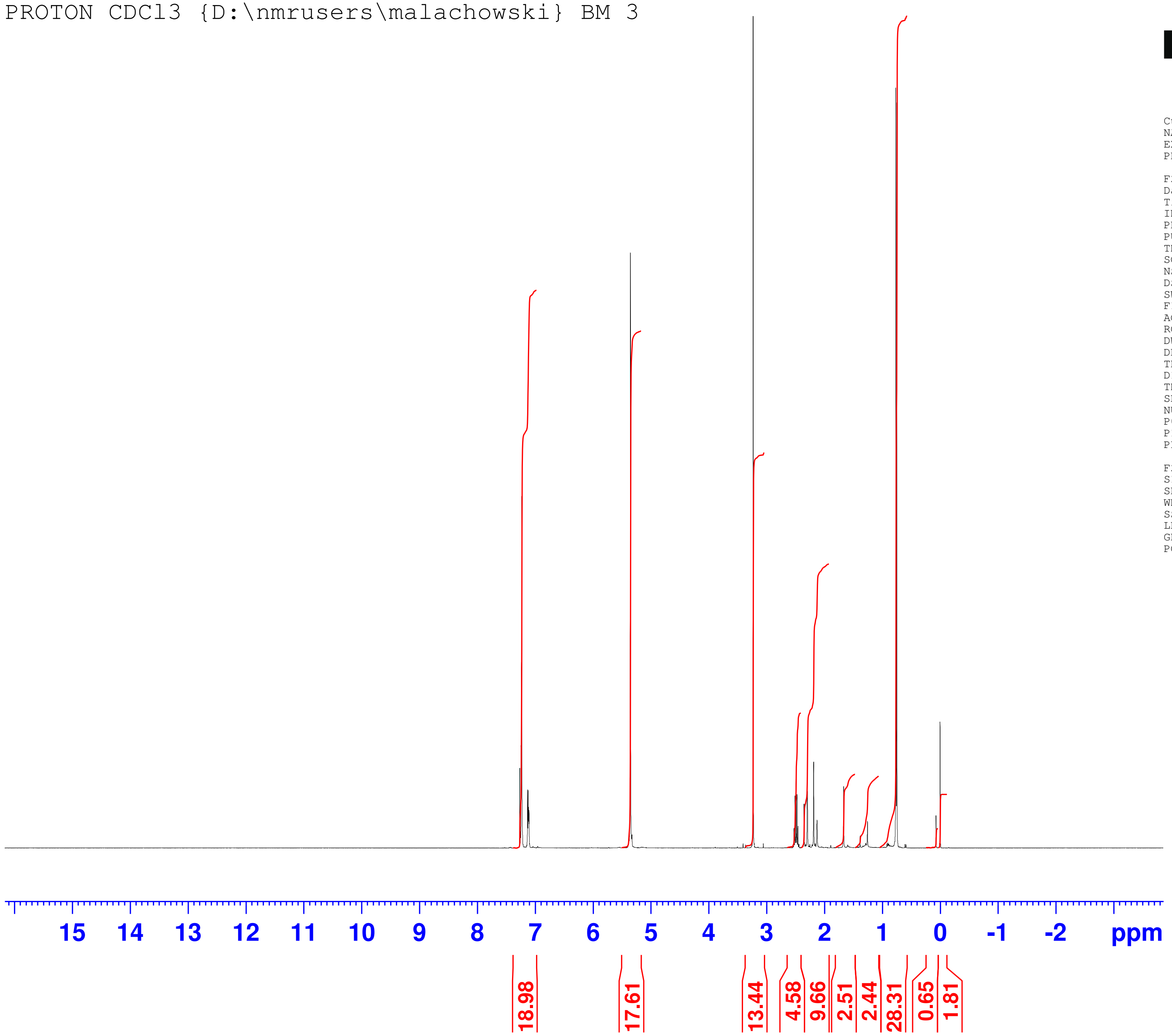

Current Data Parameters
NAME DR-107-074-076-078 combined F7 cosy
EXPNO 1
PROCNO 1

F2 - Acquisition Parameters
Date_ 20210106
Time 11.07 h
INSTRUM spect
PROBHD Z104450_0352 (
PULPROG zg30
TD 65536
SOLVENT CDCl3
NS 16
DS 2
SWH 8012.820 Hz
FIDRES 0.244532 Hz
AQ 4.0894465 sec
RG 203
DW 62.400 usec
DE 17.30 usec
TE 298.0 K
D1 1.00000000 sec
TD0 1
SFO1 400.1524709 MHz
NUC1 1H
P0 4.25 usec
P1 12.75 usec
PLW1 12.14200020 W

F2 - Processing parameters
SI 65536
SF 400.1500081 MHz
WDW EM
SSB 0
LB 0.30 Hz
GB 0
PC 1.00
