# Supplementary material for: Ni-Catalyzed Enantioselective Intramolecular Mizoroki–Heck Reaction for the Synthesis of Phenanthridinone Derivatives
Source: J Org Chem. 2023 Jun 15;88(13):8203–26. doi: 10.1021/acs.joc.3c00202 (PMC10337041; doi:10.1021/acs.joc.3c00202)
Supplement: Supplementary file 2 — jo3c00202_si_002.zip [file jo3c00202_si_002.zip › 2d-1 Deuterated side product/1H deuterated side product 2d-1/2/pdata/1/email_DR-107-074-076-078 combined F7 cosy_2_1.pdf]

DR-107-074-076-078 combined F7 cosy
COSYGPSW CDCl3 {D:\nmrusers\malachowski} BM 3

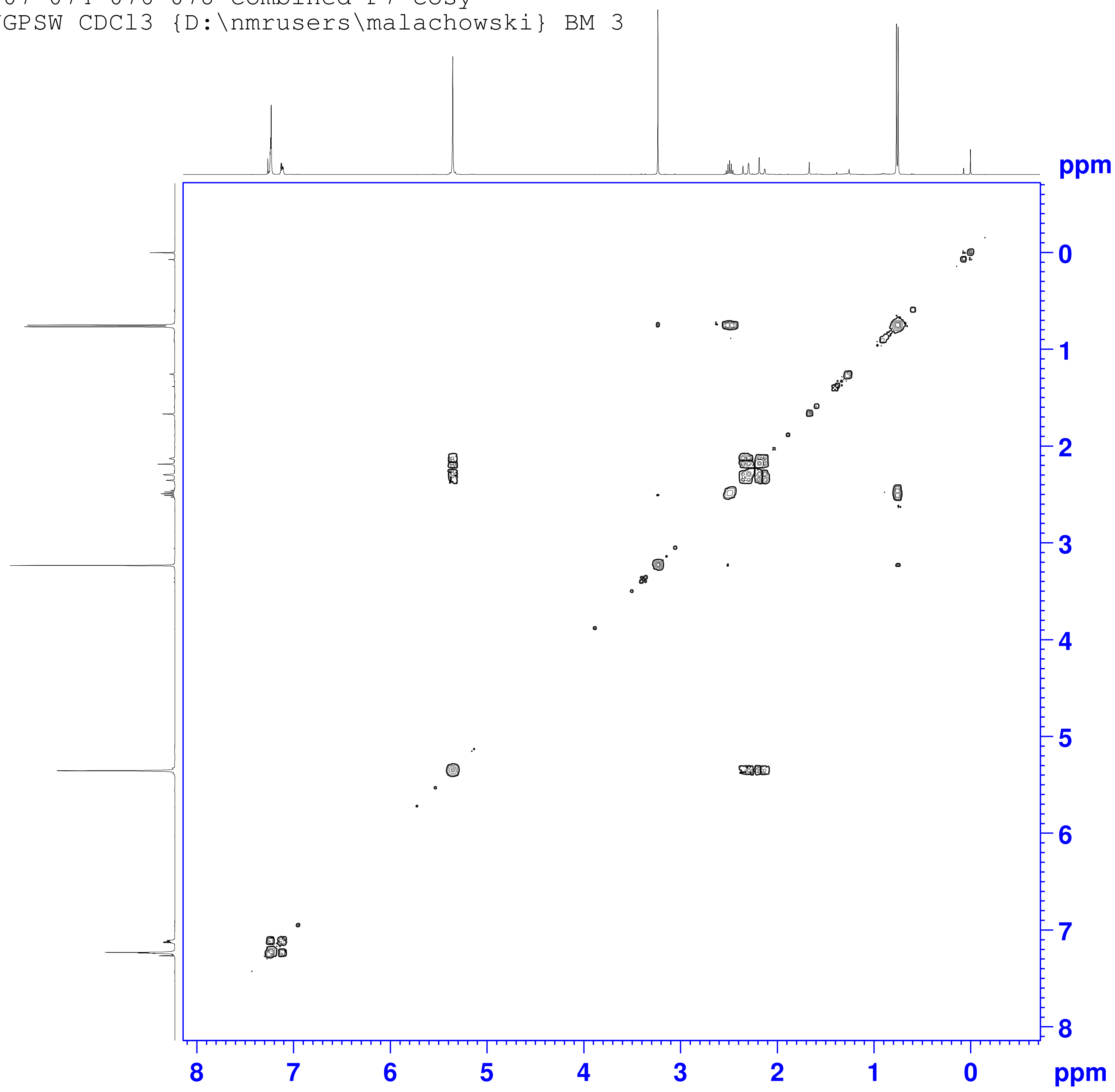

Current Data Parameters

NAME	DR-107-074-076-078 combined F7 cosy	
EXPNO	2	
PROCNO	1	

F2 - Acquisition Parameters

Date_	20210106	
Time	11.11	h
INSTRUM	spect	
PROBHD	Z104450_0352 (	
PULPROG	cosygpppqf	
TD	2048	
SOLVENT	CDCl3	
NS	1	
DS	16	
SWH	3546.099	Hz
FIDRES	3.462988	Hz
AQ	0.2887680	sec
RG	64	
DW	141.000	usec
DE	6.50	usec
TE	298.0	K
D0	0.00000300	sec
D1	1.90824997	sec
D11	0.03000000	sec
D12	0.00002000	sec
D13	0.00000400	sec
D16	0.00020000	sec
IN0	0.00028200	sec
TDav	1	
SFO1	400.1514931	MHz
NUC1	1H	
P0	12.75	usec
P1	12.75	usec
P17	2500.00	usec
PLW1	12.14200020	W
PLW10	2.19309998	W
GPNAM[1]	SMSQ10.100	
GPZ1	10.00	%
P16	1000.00	usec

F1 - Acquisition parameters

TD	128	
SFO1	400.1515	MHz
FIDRES	55.407803	Hz
SW	8.862	ppm
FnMODE	QF	

F2 - Processing parameters

SI	1024	
SF	400.1500081	MHz
WDW	QSINE	
SSB	0	
LB	0	Hz
GB	0	
PC	1.40	

F1 - Processing parameters

SI	1024	
MC2	QF	
SF	400.1500081	MHz
WDW	QSINE	
SSB	0	
LB	0	Hz
GB	0	
