# Supplementary figures and images for: Ni-Catalyzed Enantioselective Intramolecular Mizoroki–Heck Reaction for the Synthesis of Phenanthridinone Derivatives
Source: J Org Chem. 2023 Jun 15;88(13):8203–26. doi: 10.1021/acs.joc.3c00202 (PMC10337041; doi:10.1021/acs.joc.3c00202)

DR-112-057 DMSO 350 K  
PROTON DMSO {D:\nmrusers\malachowski} BM 5

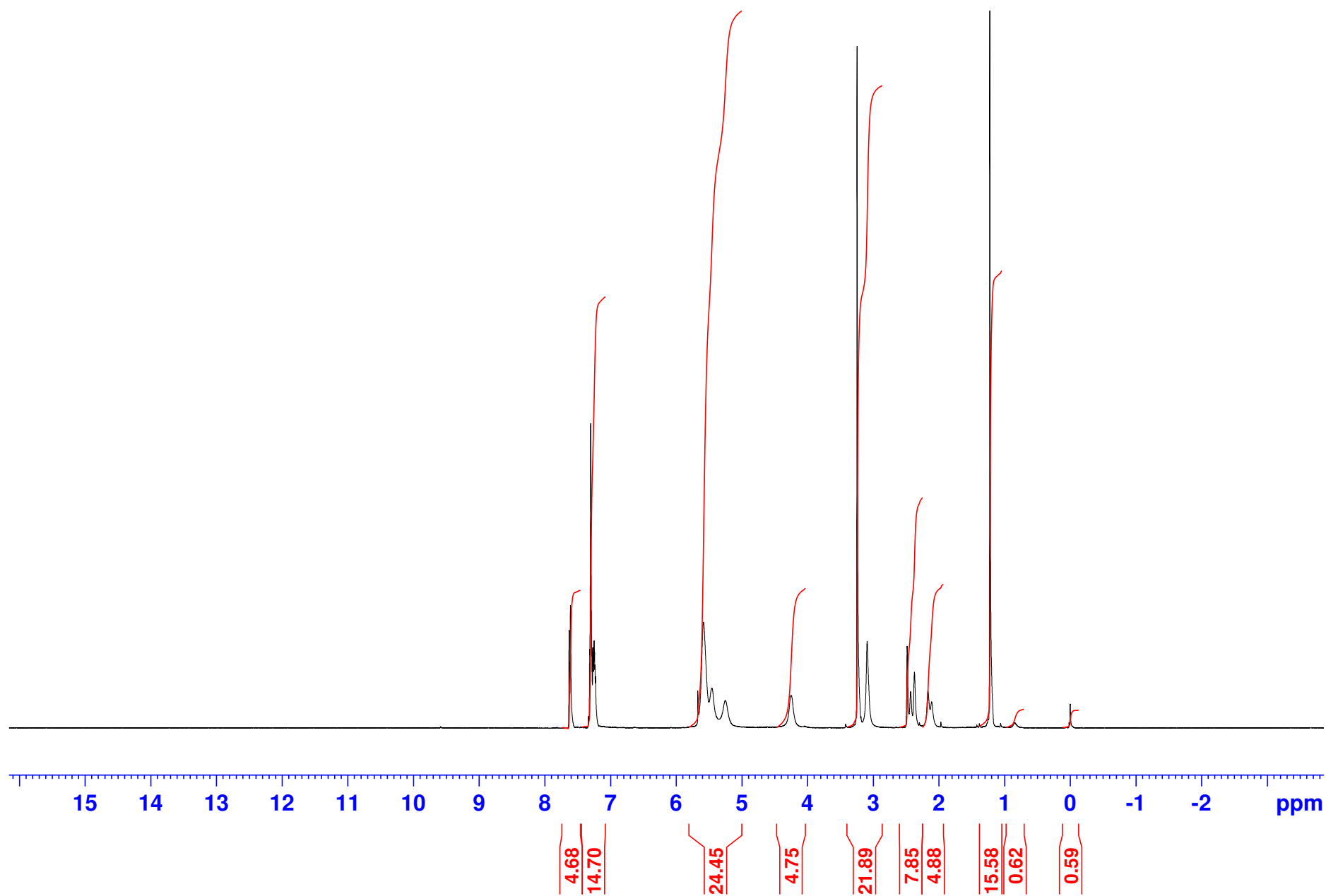

Supplement: Supplementary file 2 — jo3c00202_si_002.zip [file jo3c00202_si_002.zip › Amides (N-MOM)/1b Me-NMOM-ArBr/1H Me-NMOM-ArBr 1b/1/pdata/1/email_DR-112-057 DMSO 350 K_1_1.pdf]

YK-114-054

PROTON DMSO {D:\nmrusers\malachowski} BM 4

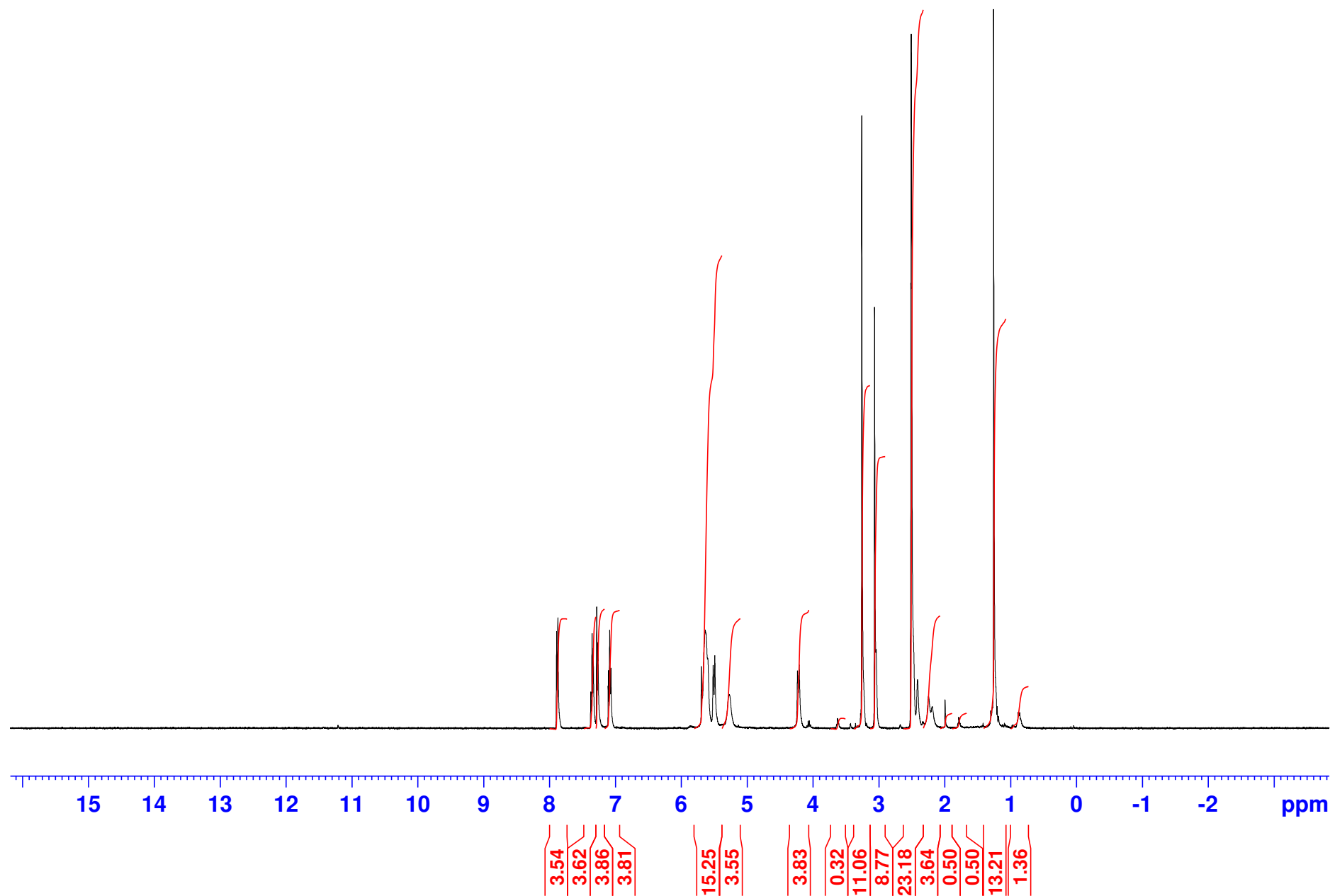

Supplement: Supplementary file 2 — jo3c00202_si_002.zip [file jo3c00202_si_002.zip › Amides (N-MOM)/1b-I Me-NMOM-ArI/1H Me-NMOM-ArI 1b-I/1/pdata/1/email_YK-114-054_1_1.pdf]
